# Profiling the proteome-wide selectivity of diverse electrophiles

**DOI:** 10.1038/s41557-025-01902-z

**Published:** 2025-10-30

**Authors:** Patrick R. A. Zanon, Fengchao Yu, Patricia Z. Musacchio, Lisa Lewald, Michael Zollo, Kristina Krauskopf, Dario Mrdović, Patrick Raunft, Thomas E. Maher, Marko Cigler, Christopher J. Chang, Kathrin Lang, F. Dean Toste, Alexey I. Nesvizhskii, Stephan M. Hacker

**Affiliations:** 1 Department of Chemistry, Technical University of Munich, Garching, Germany; 2 Department of Molecular Physiology, Leiden Institute of Chemistry, Leiden University, Leiden, the Netherlands; 3 Department of Pathology, University of Michigan, Ann Arbor, MI USA; 4 Department of Chemistry, University of California, Berkeley, CA USA; 5 Department of Chemistry, Group of Synthetic Biochemistry, Technical University of Munich, Garching, Germany; 6 Department of Molecular and Cell Biology, University of California, Berkeley, CA USA; 7 Department of Chemistry, Princeton University, Princeton, NJ USA; 8 Department of Chemistry and Applied Biosciences, ETH Zurich, Zurich, Switzerland; 9 Gilbert S. Omenn Department of Computational Medicine and Bioinformatics, University of Michigan, Ann Arbor, MI USA

**Keywords:** Proteomics, Target identification, Screening, Mass spectrometry

## Abstract

Covalent inhibitors that do not rely on hijacking enzymatic activity have mainly been limited to those targeting cysteine residues. The development of such cysteine-directed covalent inhibitors has greatly profited from the use of competitive residue-specific proteomics to determine their proteome-wide selectivity. Several probes have been developed to monitor other amino acids using this technology, and many more electrophiles exist to modify proteins. Nevertheless, there has been a lack of direct, proteome-wide comparisons of the selectivity of diverse electrophiles. Here we developed an unbiased workflow to analyse electrophile selectivity proteome-wide and used it to directly compare 56 alkyne probes containing diverse reactive groups. In this way, we verified and identified probes to monitor a total of nine different amino acids, as well as the protein amino terminus, across the proteome.

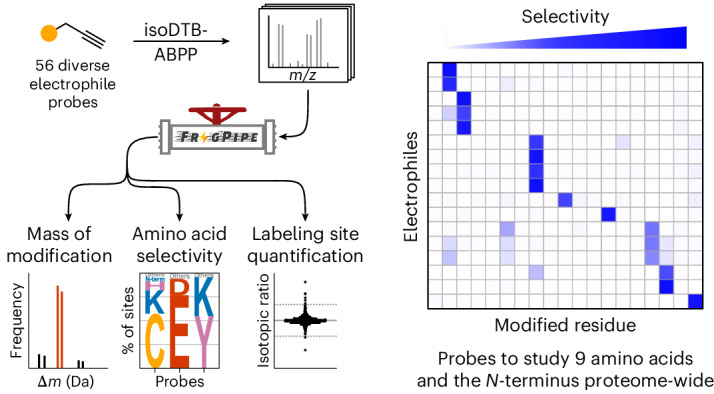

## Abstract (web summary)

Covalent inhibitors are powerful entities in drug discovery. Now the amino acid selectivity and reactivity of a diverse electrophile library have been assessed proteome-wide using an unbiased workflow. This comparative analysis and the probes described could help guide the discovery and design of covalent ligands targeting residues beyond cysteine.

## Main

Covalent inhibitors are powerful entities in drug discovery with key advantages including increased binding affinity to the target, the potential to generate selectivity among closely related proteins and improved pharmacodynamic properties^1^. Nevertheless, careful optimization of the reactivity and selectivity of these inhibitors is essential to avoid toxicity and possible immunogenic reactions^1^.

**Fig. 1:**
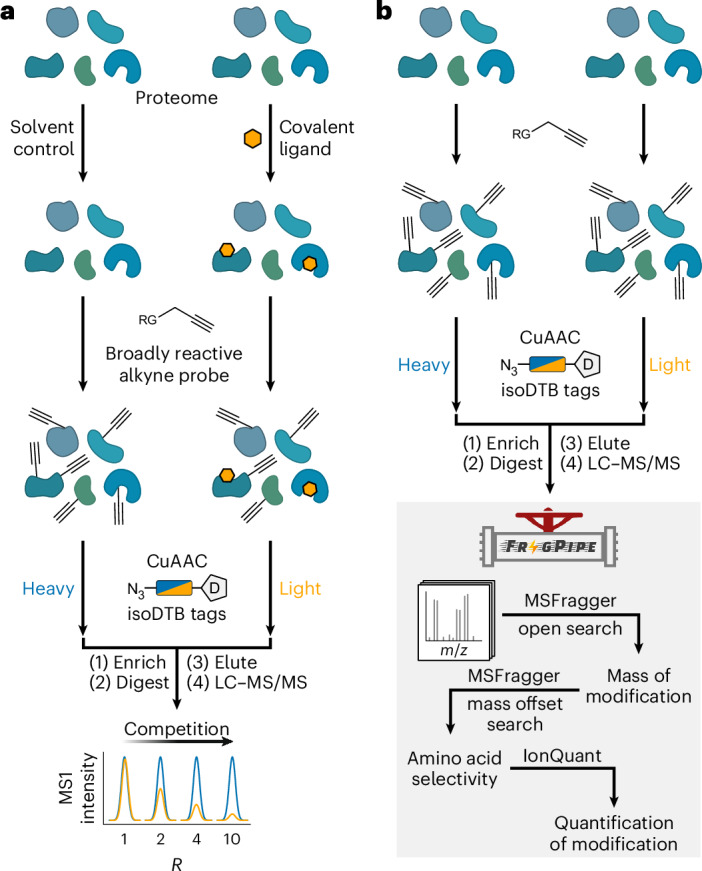
Workflows for competitive isoDTB-ABPP and unbiased analysis of electrophile selectivity. **a**, Workflow for competitive, residue-specific chemoproteomic experiments using the isoDTB-ABPP workflow^5^. **b**, Unbiased workflow to comprehensively investigate electrophile reactivity in the proteome using the MSFragger-based FragPipe computational platform^31,32^. RG, reactive group. D, desthiobiotin; CuAAC, copper-catalyzed azide-alkyne cycloaddition.

Competitive residue-specific proteomics provides essential tools for assessment of the proteome-wide selectivity of covalent inhibitors targeting cysteines based on the isotopic tandem orthogonal proteolysis-activity-based protein profiling platform^2–4^. Our isotopically labelled desthiobiotin azide (isoDTB) tags enable a streamlined experimental workflow (Fig. 1a) in which two samples of a proteome of interest are treated with a covalent ligand or with the corresponding solvent as a control^5^. Next, a broadly reactive alkyne probe is applied that labels many residues with alkynes. Residues already engaged by the ligand are blocked from this reactivity. In the next step, isotopically differentiated isoDTB tags are attached using copper-catalysed azide–alkyne cycloaddition^6^ to differentiate the proteins originating from the compound-treated and vehicle-treated samples. Then, the two samples are mixed, enriched and proteolytically digested, and the modified peptides are eluted, before being identified and quantified using liquid chromatography coupled to tandem mass spectrometry (LC–MS/MS). Peptides containing residues that are engaged by the covalent ligand will show high ratios (*R*) between the two samples (*R* ≫ 1), whereas unaffected peptides will have ratios close to 1. In this way, target engagement and selectivity of covalent inhibitors can be investigated proteome-wide in a quantitative fashion^5^.

Covalent inhibitors that do not rely on hijacking enzyme activity have so far almost exclusively targeted cysteine residues^7^. However, as cysteine is a very rare amino acid, many binding pockets contain no suitable cysteine for covalent engagement^8^. Furthermore, various other nucleophilic residues (for example, lysines^9,10^, histidines^11,12^, and aspartates and glutamates^13^) represent targets of interest, as they are key to the mechanisms of action of many enzymes and can be posttranslationally modified (for example, by ubiquitination^14^, acylation^15^, methylation^16^ or phosphorylation^17^). Many reactive groups have been developed that target residues other than cysteines^18^, but global investigation of their targets and amino acid selectivity remains a challenge. Use of alkyne, azide or (desthio)biotin derivatives enables such investigations using direct enrichment^19^, but making these derivatives can be synthetically challenging, and this method requires the resulting modification to be stable at all residues. By contrast, use of a broadly reactive alkyne probe enables competitive residue-specific proteomics of unmodified covalent inhibitors regardless of the stability of their adducts. Use of such probes has been reported for cysteines^2,3,20,21^, lysines^22,23^, aspartates and glutamates^8,24^, methionines^25,26^ and tryptophans^27,28^, as well as tyrosines^29,30^. However, various different strategies have been used for the affinity tags, isotopic labelling, mass spectrometric instrumentation and data analysis in different studies, making it impossible to directly compare the reactivity and selectivity of the reported probes. To address this challenge, we directly compared a large variety of electrophiles and established their amino acid selectivity using the isoDTB activity‐based protein profiling (isoDTB-ABPP) workflow^5^. We enhanced and developed features for the MSFragger-based^31,32^ FragPipe computational platform so that proteome-wide electrophile selectivity could be studied in a completely unbiased fashion (Fig. 1b). Using this modified platform, we verified or identified probes to study nine different amino acids, as well as the protein amino terminus, proteome-wide. This set of probes will enable competitive profiling of covalent inhibitors against a variety of reactive amino acids, thereby guiding covalent ligand development. As we were most interested in antibacterial applications^5^, we performed the main part of our analysis in the lysate of *Staphylococcus aureus* SH1000 (ref. ^33^).

## Results and discussion

### Unbiased analysis of proteome-wide electrophile selectivity

We tailored the MSFragger-based FragPipe computational platform for competitive residue-specific proteomics^31,32^. FragPipe’s ultrafast fragment-ion indexing method is especially powerful for the complex data analyses needed to identify and localize modifications on peptides in an unbiased fashion. To validate additional features, we used a published dataset^5^, in which 1 mM iodoacetamide alkyne (**IA-alkyne**) was used in a noncompetitive isoDTB-ABPP workflow (Fig. 1b).

First, we optimized FragPipe’s open search to investigate which masses of modification were found on the detected peptides. In this workflow, the peptide is identified on the basis of the MS2 level, and the MS1 mass information is used to deduce the mass of the respective modification. Owing to the complex underlying data analysis, the open search^31,32,34,35^ implements extensive data filtration^34,36–38^, deisotoping^39^, mass calibration^32^ and summation of mass shifts^35^ to produce the final output. MSFragger efficiently handles the resulting large search space and enables discovery of unknown modifications.

**Fig. 2:**
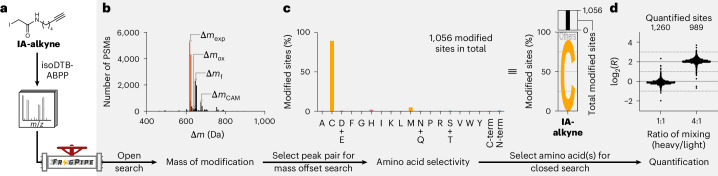
An unbiased workflow to study electrophile selectivity using the MSFragger-based FragPipe computational platform. **a**, Labelling of the proteome of *S. aureus* SH1000 with 1 mM **IA‑alkyne** and analysis using the isoDTB-ABPP workflow^5^ (Fig. 1b) resulted in generation of MS data for analysis of proteome-wide reactivity and selectivity. **b**, Through analysis with an open search in MSFragger^31,32^, the masses of modification that occurred proteome-wide were assigned. The peaks highlighted in red are the expected modifications (Δ*m*_exp_) resulting from alkylation and modification with the light and heavy isoDTB tags, respectively. Further modifications of the alkylated peptides by oxidation (Δ*m*_ox_), formylation (Δ*m*_f_) or carbamidomethylation on a second cysteine (Δ*m*_CAM_) were also detected. **c**, One peak pair (Δ*m*_exp_) was selected for an MSFragger mass offset search to localize this modification to the modified amino acid(s), allowing selectivity to be assessed across all proteinogenic amino acids. The bar graph represents the fraction of all modified sites that were modified at the indicated amino acid. The same data are also presented in a letter plot, in which the size of the letter is scaled by the fraction of all modified sites that were modified at the indicated amino acid. All amino acids that were modified in fewer than 5% of cases are summarized as ‘Others’. The total number of modified sites is given as a bar graph on top of the letter plot. **d**, One amino acid (cysteine) was selected for quantification at the selected masses (Δ*m*_exp_) using MSFragger closed search and the IonQuant quantification module^42^. Here, two datasets were analysed, in which the heavy and light samples were mixed at a ratio of 1:1 and 4:1, respectively. Grey solid lines indicate the expected values of log_2_(*R*) = 0 and log_2_(*R*) = 2. Grey dashed lines indicate the respective preferred window of quantification (−1 < log_2_(*R*) < 1 for the 1:1 ratio; 1 < log_2_(*R*) < 3 for the 4:1 ratio). The total number of quantified sites is given at the top of the plot. The mass spectrometric experiments used for this analysis were performed as part of an earlier study^5^. All data are based on technical duplicates. C-term, C-terminal modification; N-term, N-terminal modification; PSM, peptide spectrum match. Source data

In addition to the expected modification (Δ*m*_exp_) by alkylation with **IA-alkyne**, we detected formylation (Δ*m*_f_) of the modified peptides; this can occur during elution, redissolving or storage of peptides in solutions containing formic acid^40^ (Fig. 2a,b). Further, we identified smaller peaks corresponding to oxidation of the formed thioether (Δ*m*_ox_) or carbamidomethylation at a second cysteine (Δ*m*_CAM_). The detection of these minor modifications verified that MSFragger could identify different probe modifications proteome-wide in an unbiased fashion. As the highest deviation of all these masses of modification from the expected value was 0.0044 Da (6.9 ppm), the molecular formula could be directly deduced from the mass spectrometry (MS) data for unknown modifications.

Next, we performed a mass offset search, which involves searching at the mass with an indicated offset from the mass of the unmodified peptide^31,32^. The modification is computationally localized to an amino acid residue or a stretch of amino acids without previous specification of which amino acids might be modified^32^. Thus, FragPipe allows analysis of selectivity towards all amino acid residues and protein termini simultaneously. As expected, the offset corresponding to alkylation with **IA-alkyne** (Δ*m*_exp_) showed high cysteine selectivity (89%; Fig. 2c).

Finally, the relative intensities of the light and heavy channels need to be accurately quantified. Previously, complex in-house software^3,41^ or workarounds in existing software^5^ often had to be used. Therefore, we extended FragPipe’s IonQuant^42^ to allow relative quantification of isotopically labelled modified peptides. Using a mass offset search, we quantified 1,896 modified peptides, with 99% in the preferred quantification window of −1 < log_2_(*R*) < 1 (Supplementary Fig. 1). We also used a closed search, in which the potentially modified amino acid(s) are specified before the search and one modification with the probe per peptide is allowed (Supplementary Discussion), to quantify 1,260 cysteines (>99% in the preferred window; Fig. 2d). The total number of modified sites, which is used for selectivity analysis based on a mass offset search (here 1,056 total modified sites; Fig. 2c), and the number of quantified residues in a closed search (here 1,260 cysteines) or mass offset search (here 1,896 peptides; Supplementary Fig. 1) is expected to differ because of differences in data analysis and filtering.

We also analysed a published dataset^5^ in which the heavy and light samples were mixed at a ratio of 4:1. Using a closed search, we quantified 989 cysteines, with 98% in the preferred quantification window of 1 < log_2_(*R*) < 3 (Fig. 2d). The high quality of this quantification data was comparable with that obtained by data evaluation with MaxQuant^43^ using our previously described workaround^5^ or pFind 3 (ref. ^44^) using a custom script for downstream analysis^41^ (Supplementary Fig. 2). Importantly, our automated FragPipe workflow simplifies the data analysis and allows analysis for probes that are not selective for a certain amino acid type.

Overall, the optimized FragPipe computational platform enables completely unbiased analysis of residue-specific proteomic data obtained with various probes, including identification of the mass of the modification, its amino acid selectivity and its use for quantitative applications. While this manuscript was under consideration, the pChem computational platform^45^ was reported; this platform produced similar results using our benchmarking data (Supplementary Discussion).

Having our unbiased analysis workflow at hand, we applied a standardized sample preparation protocol for all probes throughout this project to directly compare the proteome-wide reactivity and selectivity of diverse alkyne-containing probes (Supplementary Discussion). Two identical samples of *S. aureus* lysate were treated with 100 µM of the respective probe, modified with 100 µM of the light or heavy isoDTB tag, mixed at a ratio of 1:1 and analysed. A concise overview of the reactions of all electrophiles used in this study with proteinogenic amino acids is given in Supplementary Table 1.

### Diverse chemistries allow monitoring of cysteines

**Fig. 3:**
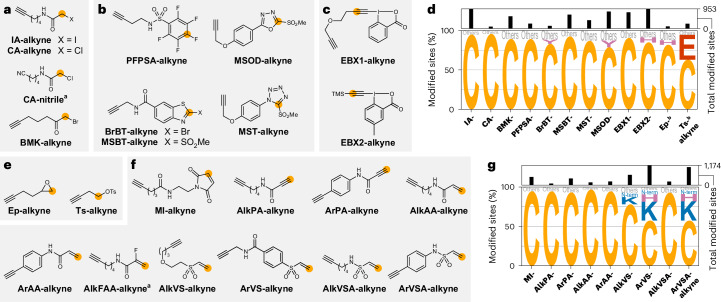
Amino acid selectivity of electrophiles targeting cysteines. **a**–**c**,**e**,**f**, Structures of alkyne probes containing α-halocarbonyl (**a**), S_N_Ar (**b**), hypervalent iodine (**c**), S_N_2 (**e**) or Michael acceptor (**f**) electrophiles that were investigated for their proteome-wide amino acid selectivity. Orange circles indicate the initial site of electrophilic reactivity. **d**,**g**, Amino acid selectivity of probes targeting cysteines (**d**) and reacting as Michael acceptors (**g**) upon treatment of the proteome of *S. aureus* SH1000 at a probe concentration of 100 µM. The data are presented as letter plots, in which the size of each letter is scaled by the fraction of all modified sites that were modified at the indicated amino acid. All amino acids that were modified in fewer than 5% of cases are summarized as ‘Others’. The total numbers of modified sites are given as a bar graph on top of the letter plot. ^a^No clear mass of modification was detected, and therefore no analysis of the amino acid selectivity was possible. ^b^Data for the indicated probe at 1 mM are shown. All data are based on technical duplicates. Source data

Using the standard conditions for **IA-alkyne**^3^ (Fig. 3a), we detected 95% selectivity for cysteines and quantified 1,197 cysteines (Fig. 3d and Supplementary Figs. 3 and 4). Notably, using the full workflow allowed us to study many more sites than was possible in an attempt to monitor modifications without enrichment (Supplementary Discussion and Supplementary Fig. 5). An increase in the concentration to 1 mM resulted in 86% selectivity for cysteines. Chloroacetamide **CA-alkyne**^3^ and α-bromomethyl ketone **BMK-alkyne**^46^ (Fig. 3a) also demonstrated high selectivity for cysteines (96% and 89%, respectively) and allowed quantification of 230 and 976 cysteines, respectively (Fig. 3d and Supplementary Figs. 3 and 4). A chloroacetamide negative control lacking the alkyne (**CA-nitrile**; Fig. 3a) did not yield any clear modification (Supplementary Fig. 3).

Nucleophilic aromatic substitution (**PFPSA-alkyne**^47^, **BrBT-alkyne**, **MSBT-alkyne**, **MST-alkyne** and **MSOD-alkyne**^20^; Fig. 3b) showed high cysteine selectivity (70–92%) and allowed quantification of 362–1,061 cysteines (Fig. 3d and Supplementary Figs. 6 and 7). We also investigated hypervalent iodine reagents that have been described for labelling of cysteines in the proteome^21^. Whereas **EBX1-alkyne**^48,49^ introduced different modifications to cysteine, **EBX2-alkyne**^50^ selectively led to the minimal modification with an ethynyl group (85%, 1,251 cysteines; Fig. 3c,d and Supplementary Figs. 8–11). Finally, nucleophilic substitution at unactivated sp^3^-carbon centres (**Ep-alkyne** and **Ts-alkyne**; Fig. 3e) showed a clear preference for cysteines, although substantial modification of glutamates was also found for **Ts-alkyne** (Fig. 3d and Supplementary Figs. 12 and 13).

In aggregate, these probes quantified 1,941 cysteines, covering 37% of the 5,268 cysteines encoded in the *S. aureus* genome. **IA-alkyne** can still be considered the gold standard for monitoring of cysteine residues with residue-specific proteomics. Nevertheless, we verified the existence of many other complementary probes that could further increase coverage, with **EBX2-alkyne** and **MSBT-alkyne** being especially powerful (Supplementary Fig. 14).

### Michael acceptors preferentially react with cysteines

Michael acceptors are mainstays of the design of covalent inhibitors (Fig. 3f). For a maleimide probe (**MI-alkyne**, after hydrolysis of the resulting succinimide (Supplementary Fig. 15)) and propiolamides (**AlkPA-alkyne** and **ArPA-alkyne**), we detected high selectivity (93–95%) and quantified a total of 283–752 cysteines (Fig. 3g and Supplementary Figs. 16 and 17). We also observed the expected modification and strong proteomic labelling (160–1,174 localized sites; Fig. 3f,g and Supplementary Figs. 18–21) for all acceptor-substituted terminal alkenes except for **AlkFAA-alkyne**. Whereas **AlkAA-alkyne** labelled cysteine residues with 97% selectivity, fewer than 60% of labelled sites were cysteines for **ArVSA-alkyne** and **ArVS-alkyne** (>20% lysines, ~9% histidines, ~5% protein N-termini). Notably, N-terminal labelling occurred preferentially on prolines, when the initial methionine was removed^51^ (Supplementary Fig. 21). Although Michael acceptors can be designed to also target other amino acid residues such as lysines^52^, cysteines as the major modification sites need to be monitored carefully.

### Studying lysine residues proteome-wide

**Fig. 4:**
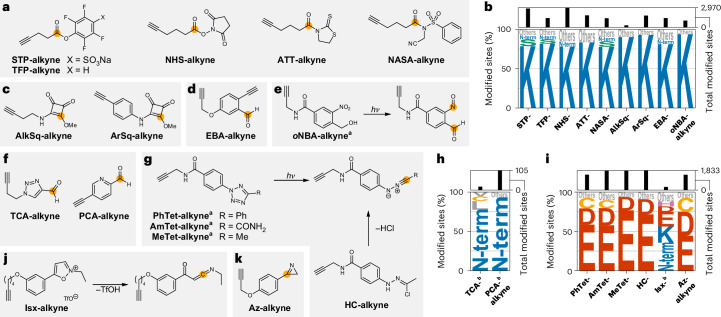
Amino acid selectivity of electrophiles targeting lysines, protein N-termini, aspartates and glutamates. **a**,**c**–**g**,**j**,**k** Structures of alkyne probes containing or producing activated ester (**a**), squaric acid ester (**c**), ethynylbenzaldehyde (**d**), nitrosobenzaldehyde (**e**), heteroaryl aldehyde (**f**), nitrilimine (**g**), ketoketenimine (**j**) or azirine (**k**) electrophiles that were investigated with respect to their proteome-wide amino acid selectivity. Orange circles indicate the initial site of electrophilic reactivity. For probes ***o*NBA-alkyne**, **PhTet-alkyne**, **AmTet-alkyne**, **MeTet-alkyne**, **HC-alkyne** and **Isx-alkyne**, the reactions leading to the reactive intermediate are also shown. **b**,**h**,**i**, Amino acid selectivity of probes targeting lysines (**b**), N-termini (**h**) or carboxylic acid residues (**i**) upon treatment of the proteome of *S. aureus* SH1000 at 100 µM probe concentration. The data are represented as letter plots, in which the size of each letter is scaled by the fraction of all modified sites that were modified at the indicated amino acid. All amino acids that were modified in fewer than 5% of cases are summarized as ‘Others’. The total numbers of modified sites are given as a bar graph on top of the letter plot. ^a^Labelling was performed using UV activation at 280–315 nm (**PhTet-alkyne**, **AmTet-alkyne** and **MeTet-alkyne**) or 365 nm (***o*NBA-alkyne**) for 10 min. ^b^Data for the indicated probe at 1 mM are shown. All data are based on technical duplicates. Source data

The activated ester probe **STP-alkyne** (Fig. 4a) allows monitoring of many lysine residues in the proteome^22^. We verified its selectivity (78%; Fig. 4b and Supplementary Figs. 22 and 23) and quantified 3,277 lysines. The remaining peptides were mainly labelled at serines (9%), threonines (2%) or N-termini (5%). This selectivity was retained even at a probe concentration of 1 mM. Four additional acylation reagents (**TFP-alkyne**^22^, **NHS-alkyne**^23^, **ATT-alkyne**^53^ and **NASA-alkyne**^54^; Fig. 4a) displayed similar selectivity and allowed quantification of 2,145–4,404 lysines (Fig. 4b and Supplementary Figs. 22 and 24). **STP-alkyne** also quantified 428 serines, 165 threonines and 152 protein N-termini.

Squaric acid derivatives also react with amines under physiological conditions^55,56^. Both **AlkSq-alkyne** and **ArSq-alkyne** demonstrated very high selectivity for lysines (93% and 90%, respectively; Fig. 4b,c and Supplementary Figs. 25 and 26). Therefore, **ArSq-alkyne** is a promising broadly reactive alkyne probe for lysines (2,990 quantified lysines), whereas structures like **AlkSq-alkyne** show potential for use in covalent inhibitor design owing to their tempered reactivity (1,339 quantified lysines)^56^. Another reactivity of lysines is the formation of imines with aldehydes. Previously, 2-ethynyl-benzaldehyde-based probes including **EBA-alkyne** (Fig. 4d) were shown to form imines that cyclized to stable isoquinolinium salts^57^ (Supplementary Fig. 27). **EBA-alkyne** exhibited high lysine selectivity and good proteomic coverage (81%, 3,796 quantified lysines; Fig. 4b and Supplementary Figs. 28 and 29). Similarly, the nitrosobenzaldehyde formed by irradiation of ***o*NBA-alkyne** reacted irreversibly with lysines in cells^58^ (Fig. 4e and Supplementary Fig. 30). We detected highly selective modification of lysine by ***o*NBA-alkyne**, which was retained even at 1 mM probe concentration (93%; 1,456 lysines; Fig. 4b and Supplementary Figs. 31 and 32). Importantly, several of these probes also quantified a substantial number of protein N-termini (243 for **AlkSq-alkyne**, 252 for **ArSq-alkyne**, 253 for **EBA-alkyne** and 35 for ***o*NBA-alkyne**).

Virtually none (0.05–0.68%) of the modifications with any of the lysine-directed probes occurred next to a proteolysis site, which indicates that the modified lysines are not recognized as cleavage sites by trypsin. As this could affect the detectability of some sequences^59^, the use of complementary proteases to increase lysine coverage may be particularly useful. Taking the data of all lysine-directed probes together, we quantified 9,129 lysines, covering 15% of the 62,166 lysines encoded in the genome of *S. aureus*. Although **STP-alkyne** remains the reagent of choice to study lysines for standard applications, **ArSq-alkyne**, **EBA-alkyne** and ***o*NBA-alkyne** also displayed high selectivity using complementary chemistries (Supplementary Fig. 33).

### Global monitoring of N-termini of proteins

Although several lysine-directed probes allow monitoring of protein N-termini, selective chemistry is highly desirable. Carboxaldehydes of electron-poor heteroaromatics have previously been used to modify proteins (**TCA-alkyne**^60^ and **PCA-alkyne**^61^; Fig. 4f). Although we were not able to detect more than a few sites for **TCA-alkyne** in the proteome, **PCA-alkyne** showed high selectivity for the protein N-terminus (93%) and allowed quantification of 167 protein N-termini at 1 mM probe concentration (Fig. 4h and Supplementary Figs. 34–36). The coverage certainly needs to be improved; however, **PCA-alkyne** is a suitable starting point for selective monitoring of the protein N-terminus proteome-wide. Combining the data for **PCA-alkyne** (1 mM), **STP-alkyne**, **ArSq-alkyne** and **EBA-alkyne**, we were able to quantify 464 protein N-termini in 412 proteins (some proteins were detected with and without clipping of the N-terminal methionine), covering 14% of the 2,959 proteins encoded in the *S. aureus* genome.

### Monitoring aspartates and glutamates across the proteome

Previously, we developed 2,5-disubstituted tetrazoles (Fig. 4g) to globally study aspartates and glutamates^8^. We verified the expected modification by formation of a nitrilimine upon light irradiation and the subsequent reactivity leading to a diacylated hydrazine (**PhTet-alkyne**, **AmTet-alkyne** and **MeTet-alkyne**; Fig. 4g and Supplementary Figs. 37 and 38). This modification showed a strong preference for aspartates and glutamates (79–94%), with **MeTet-alkyne** quantifying 2,192 aspartates and glutamates (Fig. 4i, Supplementary Figs. 38–40 and Supplementary Discussion). Hydrazonoyl chlorides liberate the same nitrilimine species without irradiation^62^, and we obtained exquisite reactivity and selectivity with **HC-alkyne** (91%, 2,450 aspartates and glutamates; Fig. 4g,i and Supplementary Figs. 37, 41 and 42).

Isoxazolium salts such as **Isx-alkyne** can modify glutamates in proteins^63^, but we also detected substantial labelling at lysines and the protein N-terminus (Fig. 4i,j and Supplementary Figs. 43 and 44). The use of 2*H*-azirines to target aspartates and glutamates has been described (**Az-alkyne**^24^; Fig. 4k and Supplementary Fig. 45). Using our workflow, we found that besides the expected reactivity (Δ*m*_exp_), these probes showed a modification with a mass of Δ*m*_exp_ + 1 Da (Supplementary Figs. 46 and 47). We further analysed their combined selectivity (Fig. 4i and Supplementary Figs. 46 and 47) and found that 75% of all modifications were at aspartates and glutamates and 18% at cysteines. Therefore, **Az-alkyne** is a valuable probe for study of aspartates and glutamates, but care must be taken to account for cysteine off-targets.

Notably, **MeTet-alkyne** and **HC-alkyne** showed strong preferences for labelling glutamates (77% and 71%, respectively) over aspartates (17% and 19%, respectively), whereas **Az-alkyne** demonstrated a smaller difference (44% for glutamates versus 30% for aspartates), indicating that it reacted more readily with the more sterically hindered aspartate. Taking all carboxylic-acid-directed probes together, we quantified 7,811 aspartates and glutamates, corresponding to 7.8% of the 100,780 aspartates and glutamates encoded in the *S. aureus* genome. Specifically, **MeTet-alkyne**, **HC-alkyne** and **Az-alkyne** constitute a set of complementary probes that allow deep profiling of these amino acids (Supplementary Fig. 48). Considering that no probes exist to selectively monitor protein carboxyl termini proteome-wide, it is also noteworthy that all carboxylic-acid-directed probes together quantified 179 protein C-termini, with **MeTet-alkyne** and **HC-alkyne** at 1 mM being the most promising (101 and 109 quantified protein C-termini, respectively).

### Residue-specific proteomics at tyrosines

**Fig. 5:**
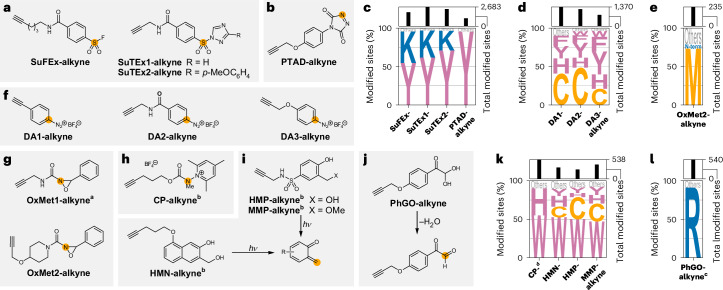
Amino acid selectivity of electrophiles targeting tyrosines, aromatic amino acids, methionines, tryptophans, histidines and arginines. **a**,**b**,**f**–**j**, Structures of alkyne probes containing or producing sulfur (VI) exchange (**a**), triazolinedione (**b**), diazonium (**f**), oxaziridine (**g**), carbamoyl (**h**), *o*-quinone methide (**i**) or glyoxal (**j**) electrophiles that were investigated with respect to their proteome-wide amino acid selectivity. Orange circles indicate the initial site of electrophilic reactivity. For probes **HMN-alkyne**, **HMP-alkyne**, **MMP-alkyne** and **PhGO-alkyne**, the reactions leading to the reactive intermediate are also shown. **c**–**e**,**k**,**l**, Amino acid selectivity of the probes targeting tyrosines (**c**), aromatic residues (**d**), methionines (**e**), tryptophans and histidines (**k**) or arginines (**l**) upon treatment of the proteome of *S. aureus* SH1000 at 100 µM probe concentration. The data are represented as letter plots, in which the size of each letter is scaled by the fraction of all modified sites that were modified at the indicated amino acid. All amino acids that were modified in fewer than 5% of cases are summarized as ‘Others’. The total numbers of modified sites are given as a bar graph on top of the letter plot. ^a^No clear mass of modification was detected and therefore no analysis of the amino acid selectivity was possible. ^b^Labelling was performed using UV activation at 280–315 nm (**CP-alkyne**, **HMP-alkyne** and **MMP-alkyne**) or 365 nm (**HMN-alkyne**) for 10 min. ^c^Data for the indicated probe at 1 mM are shown. ^d^Labelling was performed in degassed lysate under argon. All data are based on technical duplicates. Source data

Tyrosines offer a unique opportunity for various selective chemistries through reactions with the hydroxyl group, as well as with the electron-rich aromatic system. Sulfonylation of the hydroxyl group using sulfur–fluoride (**SuFEx-alkyne**)^64,65^ and sulfur–triazole (**SuTEx1-alkyne** and **SuTEx2-alkyne**)^29,30^ exchange chemistry has been established for proteome-wide approaches (Fig. 5a). We verified the tyrosine reactivity of these probes (55–71%; Fig. 5c and Supplementary Figs. 49 and 50) and found lysine residues to be the most prominent off-targets (26–41%). Consistent with the findings of a previous study in human proteomes, **SuTEx2-alkyne** showed the highest tyrosine selectivity and allowed quantification of 2,653 tyrosines in bacterial lysates.

Reagents including **PTAD-alkyne** have been established for labelling of the aromatic system of tyrosines^66^ (Fig. 5b and Supplementary Fig. 51). We identified the expected adduct that showed high selectivity for tyrosines (95%; Fig. 5c and Supplementary Figs. 52 and 53). We also detected a modification that corresponded to fragmentation of **PTAD-alkyne** to the isocyanate and subsequent reactivity, which showed some selectivity for lysines and protein N-termini and could be strongly reduced through addition of excess primary amine^67^ (Supplementary Figs. 52 and 53). Given the exquisite selectivity of the expected modification, it would be interesting to optimize the stability of these reagents to further reduce this side reactivity.

Through combination of the data for all tyrosine probes, we quantified 3,968 tyrosines covering 12% of the 32,172 tyrosines encoded in the *S. aureus* genome. Although **SuTEx2-alkyne** is currently the probe of choice for tyrosines, reagents such as **PTAD-alkyne** should enable the development of optimized complementary probes in the future (Supplementary Fig. 54).

### Diazonium salts perform arylation chemistry

During our investigation of tyrosine-directed chemistries, we also considered aryl diazonium salts that have been shown to lead to azo coupling on tyrosines^68^ (Fig. 5f and Supplementary Fig. 55). Proteome-wide, we detected only minor azo coupling and almost exclusively arylation, corresponding to a formal loss of molecular nitrogen^69^ (Supplementary Figs. 55 and 56 and Supplementary Discussion). Strikingly, next to modifications on cysteines^70^, this led to up to 75% of all modifications being localized to aromatic amino acids for **DA3-alkyne** (1,218 total, 19% phenylalanines, 19% histidines, 8% tryptophans, 30% tyrosines; Fig. 5d and Supplementary Figs. 56 and 57).

### A tool for global monitoring of methionine residues

Hypervalent iodine reagents to monitor methionines have been described but require an additional reaction step to give a stable modification^26^. Therefore, we focused on oxaziridines^25^ (Supplementary Fig. 58). **OxMet1-alkyne** did not result in detection of the expected modification (Fig. 5g and Supplementary Fig. 59). Implementing previously described design principles for more stable methionine modification^71^, we synthesized **OxMet2-alkyne** (Fig. 5g), which led to the detection of a high number of modified peptides, with a preference for methionine modification (73%, 1,838 quantified methionines, 8.5% of 21,677 encoded in the *S. aureus* genome; Fig. 5e and Supplementary Figs. 59 and 60). Thus, **OxMet2-alkyne** could be used as a tailored reagent for proteome-wide monitoring of methionines.

### Proteome-wide monitoring of tryptophans and histidines

*N*-carbamoylpyridinium salts such as **CP-alkyne** (Fig. 5h) can photochemically label tryptophans in proteins through photoinduced electron transfer^27,28^ (Supplementary Fig. 61). Upon irradiation under protective gas, **CP-alkyne** showed the expected mass of modification in the proteome, with almost complete selectivity for tryptophans (55%) and histidines (35%; Fig. 5k and Supplementary Figs. 62 and 63), allowing quantification of 467 tryptophans and 797 histidines. This selectivity was retained when the reaction was run open to air or with 1 mM **CP-alkyne** (Supplementary Figs. 62 and 63). The reactivity with histidines was especially noteworthy, as we were unsuccessful in detecting the expected modification with reported histidine-selective thiophosphorodichloridates (**TPAC-alkyne**; Supplementary Fig. 64), probably owing to instability of the conjugate^72^. The fraction of >35% for histidine labelling with **CP-alkyne** was the highest we detected for any probe, making it the probe of choice to study histidines proteome-wide. Taking all conditions for **CP-alkyne** together, we quantified 1,697 histidines, covering 9.1% of the 18,746 histidines in the *S. aureus* genome (Supplementary Fig. 65).

Simultaneously, we investigated UV-activatable *o*-quinone methide precursors for tryptophan labelling through a formal [4 + 2]-cycloaddition (**HMN-alkyne**, **HMP-alkyne** and **MMP-alkyne**; Fig. 5i and Supplementary Fig. 66). We observed a preference for tryptophans (47–53%; Fig. 5k and Supplementary Figs. 67 and 68), with cysteines (14–29%), histidines (5–13%) and tyrosines (7–10%) being the main off-targets. Therefore, **HMN-alkyne** and **MMP-alkyne** could be used as complementary probes to study tryptophans proteome-wide. Through combination of the data for all probes, we quantified a total of 701 tryptophans covering 11% of the 6,183 tryptophans encoded in the *S. aureus* genome (Supplementary Fig. 65). While this manuscript was under consideration, *N*-sulfonyl oxaziridines were demonstrated to have strong potential to further expand this probe selection^73^.

### Monitoring arginines across the whole proteome

Although arginine has been targeted with glyoxal-based reagents for bioconjugation^74^ and cross-linking^75^, no method was available to globally monitor arginines with residue-specific proteomics. Therefore, we synthesized **PhGO-alkyne** based on the known reactivity of phenylglyoxals with arginines that eventually produces a stable imidazole derivative (Fig. 5j and Supplementary Fig. 69). We detected only minor modification of the proteome, with the expected modification accompanied by an oxidation product^76^ (Supplementary Fig. 70). At an increased concentration of 1 mM **PhGO-alkyne**, however, we detected many modifications of the proteome (1,544 arginines were quantified, 5.4% of the 28,550 arginines encoded in the *S. aureus* genome), with the expected mass and high arginine selectivity (91%), alongside some oxidation (Fig. 5l and Supplementary Figs. 70 and 71). Almost none of the modified arginines were at the position of proteolytic cleavage (<0.2%), indicating that modified arginines are not recognized by trypsin as cleavage sites and that complementary proteases may help to increase coverage.

### Probes to study nine amino acids and the protein N-terminus

**Fig. 6:**
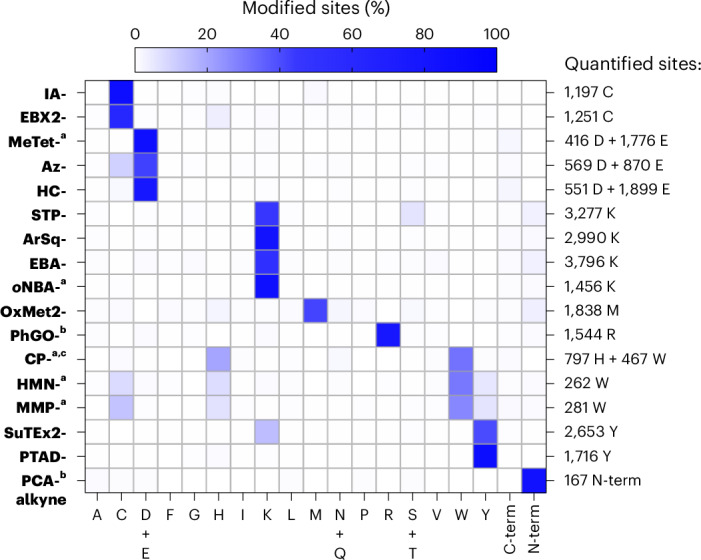
A set of 17 electrophilic probes that enables study of nine different amino acid residues and the protein N-terminus. The heatmap shows the selectivities of a selection of the probes that allow study of diverse residues in the proteome of *S. aureus*. The colour is scaled by the fraction of all modified sites that were modified at the indicated amino acid. ^a^Labelling was performed using UV-activation at 280–315 nm (**MeTet-alkyne**, **CP-alkyne** and **MMP-alkyne**) or 365 nm (***o*NBA-alkyne** and **HMN-alkyne**) for 10 min. ^b^Data for the indicated probe at 1 mM are shown. ^c^Labelling was performed in degassed lysate under argon. All data are based on technical duplicates. Source data

Through screening of 56 alkyne probes, we identified a set of 17 probes that we currently consider to be ideal for profiling nine different amino acids and the protein N-terminus proteome-wide (Fig. 6). In total, we were able to quantify 20,558 different sites in the proteome using our probe selection. These sites covered 1,399 of the 2,959 proteins encoded in the *S. aureus* genome (47%) and 85% of the annotated essential proteins (301 of 353)^77^. Thus, our probe selection enables us to gain very deep insights into the bacterial proteome.

To show that the applications of our probe selection are not restricted to bacterial systems, we also applied the probes in lysates of the human cancer cell line MDA-MB-231. We reproduced similar selectivities to those seen in the bacterial system for all probes (Supplementary Figs. 72–80) except **PCA-alkyne**^78,79^ (Supplementary Discussion).

## Conclusions

We report a universal workflow that can be used to study the reactivity and amino acid selectivity of electrophilic probes in a proteome-wide setup. By extending and developing components of the MSFragger-based FragPipe computational platform^31,32^, we were able to identify masses of modification, amino acid selectivity and relative abundance in an unbiased fashion. As well as studying all probes at 100 µM, we studied 14 of them at 1 mM to compare the effects on selectivity and coverage (Supplementary Discussion).

Although our selection of 56 alkyne probes was certainly not comprehensive, we covered most chemistries that had more broadly been applied for protein labelling and did not require additional reagents. In this way, we verified and identified tailored probes to study nine different amino acids and the protein N-terminus proteome-wide. Within our set of probes, we verified the selectivity of **IA-alkyne** and **EBX2-alkyne** for cysteines^2,3^, **STP-alkyne** for lysines^22^, **SuTEx2-alkyne** for tyrosines^29^, **CP-alkyne** for tryptophans and **PCA-alkyne** for protein N-termini, as well as **MeTet-alkyne**^8^ and **Az-alkyne**^24^ for aspartates and glutamates. For several of these amino acids, we identified additional probes based on complementary chemistries, including **ArSq-alkyne**, **EBA-alkyne** and ***o*NBA-alkyne** for lysines; **HC-alkyne** for aspartates and glutamates; **PTAD-alkyne** for tyrosines; and **HMN-alkyne** and **MMP-alkyne** for tryptophans. In addition, we developed a tailored probe for methionines (**OxMet2-alkyne**) and identified probes to study histidines (**CP-alkyne**) and arginines (**PhGO-alkyne**) in a residue-specific fashion across the proteome.

The selectivities and reactivities of the studied probes (Supplementary Tables 9 and 10) represent a valuable guide for the selection of suitable electrophiles for various applications in protein labelling and ligand monitoring. It is now possible to directly compare the amino acid selectivity of all these different chemotypes. Furthermore, we are convinced that the number of quantified sites can be used as a measure to estimate the reactivity of a certain chemotype in the whole proteome and thus to choose candidate electrophiles for different applications that require various levels of reactivity.

To investigate whether the selectivities were reproduced in a cellular environment, we performed a preliminary experiment in which we treated *S. aureus* cells with our panel of 17 probes. Strikingly, 15 of the 17 probes also allowed monitoring of sites in this setup with very similar overall selectivity (Supplementary Figs. 81–85, Supplementary Table 11 and Supplementary Discussion).

Important residues for which no selective broadly reactive alkyne probes could be established here included serines and threonines, as well as protein C-termini. Neither the fluorophosphonate **FP-alkyne**^80^ nor the phosphorus–sulfur incorporation chemistry of **PSI-alkyne**^81,82^ yielded (stable) modifications at serine or threonine (Supplementary Figs. 86 and 87). In our view, **STP-alkyne** is the best probe for monitoring of at least some serines and threonines in a residue-specific fashion. For protein C-termini, the aspartate- and glutamate-directed probes allowed us to quantify 179 protein C-termini, but increased coverage and selective probes are surely desirable.

We stress that the reported selectivities only refer to the modifications that were stable enough to survive the entire isoDTB-ABPP workflow. Some probes are likely to produce additional, more labile modifications that we did not detect directly but that will be important to consider for covalent inhibitor design. To obtain more complete information about the initial selectivity of covalent inhibitors, an important application of our probe selection will be to competitively study target engagement for any covalently reactive protein ligand regardless of its amino acid selectivity and adduct stability.

These studies have enabled the profiling of established, tailored and previously unreported probes for residue-specific proteomics, which now allow monitoring of a total of nine different amino acids and the protein N-terminus. We expect our workflow and probe selection to be instrumental in the identification of selectively reactive groups for discovery and design of covalent ligands targeting diverse amino acids, thereby advancing the development of covalent inhibitors for the many protein binding sites that lack a suitable cysteine residue for engagement with the currently dominant acrylamide-based chemistry.

## Methods

### Cultivation and lysis of bacterial and human cells

*S. aureus* SH1000 (ref. ^33^) was a gift from S. J. Foster at the Krebs Institute, Department of Molecular Biology and Biotechnology, University of Sheffield. Overnight cultures were inoculated with 5 µl of a glycerol stock into 5 ml of B medium (10 g l^−1^ peptone, 5 g l^−1^ NaCl, 5 g l^−1^ yeast extract, 1 g l^−1^ K_2_HPO_4_) and grown overnight (200 rpm, 37 °C). B medium was inoculated 1:100 with an overnight culture and incubated (200 rpm, 37 °C) until 1 h after it reached the stationary phase (optical density ~6). Cells were collected by centrifugation (10 min, 8,000*g*, 4 °C), and pellets of 100 ml initial culture were pooled and washed twice with phosphate-buffered saline (PBS; 10 mM Na_2_HPO_4_, 1.8 mM KH_2_PO_4_, 140 mM NaCl, 2.7 mM KCl; pH = 7.4) before either immediate use or storage at −80 °C. The bacterial pellets were resuspended in 5 ml PBS and transferred into 7-ml tubes containing 0.1 mm ceramic beads (Peqlab, 91-PCS-CK01L). Cells were lysed in a Precellys 24 bead mill (3 × 30 s, 6,500 rpm) while cooling with an airflow that had been precooled with liquid nitrogen. The suspension was transferred into a microcentrifuge tube and centrifuged (30 min, 20,000*g*, 4 °C). The supernatants of several samples were pooled and filtered through a 0.45-µm filter. The protein concentration of the lysate was determined using a bicinchoninic acid assay (typical concentrations were between ~2 mg ml^−1^ and ~3 mg ml^−1^), and the concentration was adjusted to 1 mg ml^−1^ with PBS. The lysates were used immediately for all MS experiments.

MDA-MB-231 cells (ATCC, catalogue no. HTB-26) were cultured in Dulbecco’s modified Eagle medium supplemented with 10% fetal bovine serum and 2 mM glutamine at 37 °C with 5% CO_2_. The cells were routinely tested for mycoplasma contamination. For preparation of lysates, cells were grown to confluence, scraped into PBS, collected by centrifugation (5 min, 800*g*) and washed with PBS. Pellets from 12 15-cm dishes were pooled. The pellets were either used immediately or stored at −80 °C. The pellets were resuspended in 5 ml PBS. Cells were lysed using sonication, and the suspension was transferred into a microcentrifuge tube and centrifuged (30 min, 20,000*g*, 4 °C). The supernatants of several samples were pooled. The protein concentration of the lysate was determined using a bicinchoninic acid assay (typical concentrations were between ~2 mg ml^−1^ and ~3 mg ml^−1^), and the concentration was adjusted to 1 mg ml^−1^ with PBS. The lysates were used immediately for all MS experiments.

### isoDTB-ABPP experiments with constitutively active probes in lysate

Two technical replicates were prepared separately as distinct samples starting from the same lysate. Two samples of 1.00 ml freshly prepared lysate of the indicated cells were incubated with 20 µl of the respective probe—5 or 50 mM stock in dimethyl sulfoxide (DMSO) (for **HC-**, **SuFEx-**, **SuTEx1-**, **SuTEx2-**, **PTAD-**, **DA1-**, **DA2-**, **DA3-**, **OxMet1-**, **OxMet2-** and **TPAC-alkyne**, dimethylformamide was used as the solvent instead of DMSO) at a final concentration of 100 µM or 1 mM, respectively—for 1 h at room temperature (for one control experiment with **PTAD-alkyne**, Tris (200 mM final concentration, pH = 7.4) was added to both samples before incubation). One sample was clicked to the heavy and one to the light isoDTB tag by addition of 120 µl of a solution consisting of 60 µl TBTA ligand (0.9 mg ml^−1^ in 4:1 ^*t*^BuOH/DMSO), 20 µl CuSO_4_⋅5H_2_O (12.5 mg ml^−1^ in H_2_O), 20 µl TCEP (13 mg ml^−1^ in H_2_O, freshly prepared) and 20 µl of the respective isoDTB tag (5 mM in DMSO). After incubation of the samples (1 h at room temperature), the light- and heavy-labelled samples were combined in 8 ml cold acetone to precipitate all proteins. Precipitates were stored at −20 °C overnight.

### isoDTB-ABPP experiments with photoactivated probes in lysate

Two technical replicates were prepared separately as distinct samples starting from the same lysate. Two samples of 1.20 ml freshly prepared lysate of the indicated cells were incubated with 24 µl of the respective photoprobe (5 or 50 mM stock in DMSO at a final concentration of 100 µM or 1 mM, respectively) for 30 min at room temperature. The samples were transferred to a six-well plate and irradiated for 10 min with either a Luzchem LZC-UVB lamp (280–315 nm; **CP-**, **HMP-**, **MMP-**, **PhTet-**, **AmTet-** and **MeTet-alkyne**) or a Philips TL-D BLB 18 W lamp (365 nm; ***o*NBA-** and **HMN-alkyne**). During irradiation, the six-well plate was placed on an ice pack, which had been precooled to 4 °C, for cooling. Then, 1.00 ml of each sample was transferred to a microcentrifuge tube, and one was clicked to the heavy and the other to the light isoDTB tag by addition of 120 µl of a solution consisting of 60 µl TBTA ligand (0.9 mg ml^−1^ in 4:1 ^*t*^BuOH/DMSO), 20 µl CuSO_4_⋅5H_2_O (12.5 mg ml^−1^ in H_2_O), 20 µl TCEP (13 mg ml^−1^ in H_2_O, freshly prepared) and 20 µl of the respective isoDTB tag (5 mM in DMSO). After incubation of the samples at room temperature for 1 h, the light- and heavy-labelled samples were combined in 8 ml cold acetone to precipitate all proteins. Precipitates were stored at −20 °C overnight.

### isoDTB-ABPP experiments with constitutively active probes in situ

Two technical replicates were prepared separately as distinct samples. *S. aureus* SH1000 pellets were freshly prepared and washed as described under ‘Cultivation and lysis of bacterial and human cells’. The bacterial pellets were resuspended in PBS to give a final optical density at a wavelength of 600 nm of 40. Two samples of 1.00 ml freshly prepared bacterial suspension were incubated with 20 µl of the respective probe—5 or 50 mM stock in DMSO (for **HC-**, **SuTEx2-**, **PTAD-** and **OxMet2-alkyne**, dimethylformamide was used as the solvent instead of DMSO) at a final concentration of 100 µM or 1 mM, respectively—for 1 h at 37 °C with shaking at 200 rpm. Cells were collected by centrifugation (10 min, 8,000*g*, 4 °C) and washed twice with 1 ml PBS before storage at −80 °C. The pellets were thawed and resuspended in 1 ml PBS with 5 µl 10 mg ml^−1^ lysostaphin (recombinant from *Staphylococcus simulans*, dissolved in 20 mM sodium acetate, pH = 4.5). The samples were incubated with shaking at 200 rpm for 1 hour at 37 °C. Then, 20 µl 20% sodium dodecyl sulfate in PBS was added, and the samples were sonicated. Samples were then centrifuged at 21,100*g* for 30 min at room temperature, and 900 µl of the supernatant was taken for the further experiments. One sample was clicked to the heavy and one to the light isoDTB tag by addition of 108 µl of a solution consisting of 54 µl TBTA ligand (0.9 mg ml^−1^ in 4:1 ^*t*^BuOH/DMSO), 18 µl CuSO_4_⋅5H_2_O (12.5 mg ml^−1^ in H_2_O), 18 µl TCEP (13 mg ml^−1^ in H_2_O, freshly prepared) and 18 µl of the respective isoDTB tag (5 mM in DMSO). After incubation of the samples (1 h at room temperature), the light- and heavy-labelled samples were combined into 8 ml cold acetone to precipitate all proteins. Precipitates were stored at −20 °C overnight.

### isoDTB-ABPP experiments with photoactivated probes in situ

Two technical replicates were prepared separately as distinct samples. *S. aureus* SH1000 pellets were freshly prepared and washed as described in ‘Cultivation and lysis of bacterial and human cells’. The bacterial pellets were resuspended in PBS to give a final optical density at a wavelength of 600 nm of 40. Two samples of 1.30 ml freshly prepared bacterial suspension were incubated with 26 µl of the respective probe (5 mM stock in DMSO with a final concentration of 100 µM) for 30 min at 37 °C with shaking at 200 rpm. Then, 1.20 ml of each sample was transferred to a six-well plate and irradiated for 10 min with either a Luzchem LZC-UVB lamp (280–315 nm; **CP-**, **MMP-** and **PhTet-alkyne**) or a Philips TL-D BLB 18 W lamp (365 nm; ***o*NBA-** and **HMN-alkyne**). During irradiation, the six-well plate was placed on an ice pack precooled to 4 °C for cooling. Next, 1.00 ml of each sample was transferred to a microcentrifuge tube. The cells were collected by centrifugation (10 min, 8,000*g*, 4 °C) and washed twice with 1 ml PBS before storage at −80 °C. The pellets were thawed and resuspended in 1 ml PBS with 5 µl 10 mg ml^−1^ lysostaphin (recombinant from *S. simulans*, dissolved in 20 mM sodium acetate, pH = 4.5). The samples were incubated with shaking at 200 rpm for 1 hour at 37 °C. Then, 20 µl 20% sodium dodecyl sulfate in PBS was added, and the samples were sonicated. The samples were centrifuged at 21,100*g* for 30 min at room temperature, and 900 µl of the supernatant was taken for the further experiments. One sample was clicked to the heavy and one to the light isoDTB tag by addition of 108 µl of a solution consisting of 54 µl TBTA ligand (0.9 mg ml^−1^ in 4:1 ^*t*^BuOH/DMSO), 18 µl CuSO_4_⋅5H_2_O (12.5 mg ml^−1^ in H_2_O), 18 µl TCEP (13 mg ml^−1^ in H_2_O, freshly prepared) and 18 µl of the respective isoDTB tag (5 mM in DMSO). After incubation of the samples (1 h at room temperature), the light- and heavy-labelled samples were combined in 8 ml cold acetone to precipitate all proteins. Precipitates were stored at −20 °C overnight.

### isoDTB-ABPP MS sample preparation

For all isoDTB-ABPP experiments, protein precipitates were centrifuged (3,500*g*, for 10 min at room temperature) and the supernatant was removed. The precipitates were resuspended in 1 ml cold MeOH by sonification and centrifuged (10 min, 21,100*g*, 4 °C). The supernatant was removed, and the washing step with MeOH was repeated once. The pellets were dissolved in 300 µl urea (8 M in 0.1 M aqueous triethylammonium bicarbonate (TEAB)) by sonification. Then, 900 µl TEAB (0.1 M in H_2_O) was added, and the solution was added to 1.2 ml of washed high-capacity streptavidin agarose beads (50 µl initial slurry, Fisher Scientific, catalogue no. 10733315) in NP40 substitute (0.2% in PBS). The samples were rotated for 1 h at room temperature to ensure binding to the beads.

The beads were centrifuged (1 min, 1,000*g* at room temperature) and the supernatant was removed. The beads were resuspended in 600 µl NP40 substitute (0.1% in PBS) and transferred to a centrifuge column (Fisher Scientific, catalogue no. 11894131). Beads were washed with 2 × 600 µl NP40 substitute (0.1% in PBS), 3 × 600 µl PBS and 3 × 600 µl H_2_O. The beads were resuspended in 600 µl urea (8 M in 0.1 M aqueous TEAB), transferred to a Protein LoBind tube (Eppendorf) and centrifuged (1 min, 1,000*g*). The supernatant was removed, and the beads were resuspended in 300 µl urea (8 M in 0.1 M aqueous TEAB) and incubated sequentially with 15 µl dithiothreitol (31 mg ml^−1^ in H_2_O) for 45 min (200 rpm, 37 °C), 15 µl iodoacetamide (74 mg ml^−1^ in H_2_O) for 30 min (200 rpm, room temperature) and 15 µl dithiothreitol (31 mg ml^−1^ in H_2_O) for 30 min (200 rpm, room temperature). The samples were then diluted with 900 µl TEAB (0.1 M in H_2_O) and centrifuged (1 min, 1,000*g*). After removal of the supernatant, the beads were resuspended in 200 µl urea (2 M in 0.1 M aqueous TEAB) and incubated with 4 µl trypsin (0.5 mg ml^−1^; Promega, V5113) overnight at 200 rpm and 37 °C.

The samples were diluted by addition of 400 µl NP40 substitute (0.1% in PBS) and transferred to a centrifuge column (Fisher Scientific, catalogue no. 11894131). Beads were washed with 3 × 600 µl NP40 substitute (0.1% in PBS), 3 × 800 µl PBS and 3 × 800 µl H_2_O. Peptides were eluted into Protein LoBind tubes with 1 × 200 µl and 2 × 100 µl formic acid (0.1% in 50% aqueous MeCN) or trifluoroacetic acid (TFA; 0.1% in 50% aqueous MeCN; used as an alternative to formic acid to avoid formylation of peptides^40^), followed by a final centrifugation (3 min, 3,000*g*). The solvent was removed in a rotating vacuum concentrator (~5 h, 30 °C), and the resulting residue was dissolved in 30 µl formic acid (1% in H_2_O) or TFA (0.1% in H_2_O) by sonification for 5 min. After washing filters (Merck, UVC30GVNB) with the resuspension buffer, the samples were loaded and filtered through them by centrifugation (3 min, 17,000*g*). The samples were then transferred to MS sample vials and stored at −20 °C until measurement.

### Sample analysis by LC–MS/MS

We analysed 5 µl of each sample using a Q Exactive Plus mass spectrometer (Thermo Fisher) coupled to an Ultimate 3000 nano HPLC system (Dionex). Samples were loaded on an Acclaim C18 PepMap100 trap column (75 µm ID × 2 cm, Acclaim, PN 164535) and washed with 0.1% TFA. The subsequent separation was carried out on an AURORA series AUR2-25075C18A column (75 µm ID × 25 cm, serial no. IO257504282) with a flow rate of 400 nl min^−1^ using buffer A (0.1% formic acid in water) and buffer B (0.1% formic acid in acetonitrile). The column was heated to 40 °C. Analysis started with washing in 5% buffer B for 7 min followed by a gradient from 5% to 40% buffer B over 105 min, a further increase to 60% buffer B in 10 min and final increase to 90% buffer B in 10 min. The concentration of buffer B was maintained at 90% for 10 min, then decreased to 5% in 0.1 min and held at 5% for another 9.9 min. The Q Exactive Plus mass spectrometer was operated in TOP10 data-dependent mode. In the orbitrap, full MS scans were collected in a scan range of 300–1,500 *m*/*z* at a resolution of 70,000 and an automatic gain control (AGC) target of 3 × 10^6^ with 80 ms maximum injection time. The most intense peaks were selected for MS2 measurement with a minimum AGC target of 1 × 10^3^ and isotope exclusion and dynamic exclusion (exclusion duration: 60 s) enabled. Peaks with unassigned charge or a charge of +1 were excluded. Peptide match was ‘preferred’. MS2 spectra were collected at a resolution of 17,500, aiming for an AGC target of 1 × 10^5^ with a maximum injection time of 100 ms. Isolation was conducted in the quadrupole using a window of 1.6 *m*/*z*. Fragments were generated using higher-energy collisional dissociation (normalized collision energy: 27%) and finally detected in the orbitrap. Proteomics data from the LC–MS/MS analyses were collected using Thermo Scientific Xcalibur (v.4.1).

### Further experimental methods

All synthetic procedures, specialized sample preparation protocols that were used for individual samples only, and complete data analysis procedures can be found in Supplementary Information.

### Reporting summary

Further information on research design is available in the Nature Portfolio Reporting Summary linked to this article.

## Online content

Any methods, additional references, Nature Portfolio reporting summaries, source data, extended data, supplementary information, acknowledgements, peer review information; details of author contributions and competing interests; and statements of data and code availability are available at 10.1038/s41557-025-01902-z.

## Supplementary information

Supplementary Information Supplementary Discussion, Figs. 1–87, Tables 6–11, synthetic experimental procedures, Methods and NMR spectra.

Reporting Summary

Supplementary Table 1 Overview of the chemical mechanisms of the electrophiles used.

Supplementary Table 2 Overview of electrophile reactivity. A summary of the key information on masses of modification, amino acid selectivity and quantification for all probes.

Supplementary Table 3 Mass of modification data for all probes. Masses of modification were determined using an open search in MSFragger-based FragPipe.

Supplementary Table 4 Amino acid selectivity data for all probes. Amino acid selectivity was determined using a Mass offset search in MSFragger-based FragPipe.

Supplementary Table 5 Quantification using all probes. Quantification was mainly performed using MSFragger closed search and IonQuant labelling-based quantification. Individual datasets are also included that were quantified using MSFragger mass offset search and IonQuant labelling-based quantification or using MaxQuant or pFind 3.

## Source data

Source Data Fig. 2 Numerical data to reproduce the plots in Fig. 2.

Source Data Fig. 3 Numerical data to reproduce the plots in Fig. 3.

Source Data Fig. 4 Numerical data to reproduce the plots in Fig. 4.

Source Data Fig. 5 Numerical data to reproduce the plots in Fig. 5.

Source Data Fig. 6 Numerical data to reproduce the plots in Fig. 6.

## Data Availability

The mass spectrometric data for all proteomic analyses, which comprise all the raw data needed to reproduce our findings, have been deposited to the ProteomeXchange Consortium (http://proteomecentral.proteomexchange.org) via the PRIDE partner repository^83^ with dataset identifiers PXD024454 and PXD065811, where they are freely available. Source data are provided with this paper.

## Code availability

Code for the MSFragger-based FragPipe analyses is available at https://fragpipe.nesvilab.org/. A custom script for downstream analysis of the pFind 3 data is available at https://github.com/morpheusliu/Post-processing-program-for-pFind3-results.
