# Supplementary material for: Profiling the proteome-wide selectivity of diverse electrophiles
Source: Nat Chem. 2025 Oct 30;17(11):1712–21. doi: 10.1038/s41557-025-01902-z (PMC12580327; doi:10.1038/s41557-025-01902-z)
Supplement: Supplementary file 1 — Supplementary Discussion, Figs. 1–87, Tables 6–11, synthetic experimental procedures, Methods and NMR spectra. [file 41557_2025_1902_MOESM1_ESM.pdf]

nature chemistry

Supplementary information https://doi.org/10.1038/s41557-025-01902-z

Profiling the proteome-wide selectivity of diverse electrophiles

In the format provided by the authors and unedited

Table of Contents

Supplementary Discussion **2**
Data analysis allowing multiple modifications of a single peptide 2
Comparison to data analysis using pChem 2
Reactivity of alkyne handle 2
Tag- and enrichment-free workflow 2
Prolonged irradiation with MeTet-alkyne 2
Arylation chemistry with diazonium salts 2
Comparison of quantified sites at low and high concentrations 3
Use of the probes in cells 3
Supplementary Figures **4**
Supplementary Tables **66**
Experimental Procedures **80**
General remarks to synthetic procedures 80
Synthetic procedures 81
Synthesis of the isoDTB tags 105
Control experiment for the role of CuAAC conditions in labelling with DA1-alkyne 105
isoDTB-ABPP Experiments with CP-alkyne under inert gas 105
Chemoproteomic experiment without enrichment 105
isoDTB-ABPP Data Analysis **105**
Adding labelling-based quantification support on the MS1-level to IonQuant 105
General setup of analysis software 106
Analysis of the mass of modifications with FragPipe 106
Analysis of amino acid selectivity with FragPipe 106
Quantification with FragPipe 108
Quantification with MaxQuant 108
Quantification with pFind 3 109
Analysis of peptides with multiple probe modifications using FragPipe 110
Data analysis with pChem 110
Generation of sequence logos with pLogo 111
Analysis of protein essentiality 111
Data availability 111
NMR Spectra **113**
References **182**

Supplementary Discussion

Data analysis allowing multiple modifications of a single peptide

In our analyses, we usually only include peptides with one modification with the respective probe clicked to the heavy or light isoDTB tag, respectively. By including up to two modifications with **IA-alkyne** clicked to the isoDTB tag as well as one with the unclicked probe in a Closed Search, we also checked in our benchmark data, if we detect peptides, in which more than one residue is labelled with **IA-alkyne**. In this way, we detected 15,075 peptide spectrum matches (PSMs) with exactly one modification with **IA-alkyne**, which is clicked to the isoDTB tag. We could only detect very few PSMs (122) with two residues modified with **IA-alkyne**, in which both are clicked to the isoDTB tag. We did identify several PSMs (1201), in which one residue is modified with **IA-alkyne** clicked to the isoDTB tag and one only with **IA-alkyne**. In this way, while adding the possibility of multiple modified residues could help to increase coverage, to not overcomplicate the data analysis and to allow high confidence localization of the modification, we only included single modification with the probe in all further analysis.

Comparison to data analysis using pChem

When we analysed our benchmark data with the pChem computational platform,[1] the expected masses of modification for reaction with the probe and for additional modification by formylation were detected in agreement with our data. As the selectivity is reported by pChem on the PSM level (92% cysteine selectivity) and we report on the site level (89%), the selectivity values are not directly comparable. If we analyse our data at the PSM level, we also detect 92% selectivity for cysteines. Therefore, both tools are complementary in assessing electrophile selectivity.

Reactivity of alkyne handle

It is noteworthy that all our probes contain terminal alkynes that could react with catalytic cysteines *e.g.* of deubiquitinating enzymes.[2] Nevertheless, these alkynes show a very low reactivity towards free thiols. Therefore, the reactivity of the probes investigated here should be clearly dominated by the reactivity of the tested electrophiles. Furthermore, such modification would destroy the CuAAC handle and avoid enrichment and detection.

Tag- and enrichment-free workflow

We tested whether our analysis can also be done directly with probe-labelled lysate without attaching isoDTB tags and without performing enrichment on the example of **IA-alkyne** (Supplementary Fig. 5). While we were able to detect the expected mass of modification and high cysteine selectivity (83%), the data was dominated by unmodified peptides (<1% of identified spectra had the modification and ~80% were unmodified or carbamidomethylated peptides). In this way, we were only able to localise the modification on 95 sites. Therefore, while modifications with electrophiles can be found and localised without enrichment, the enrichment step is essential to obtain high coverage.

Prolonged irradiation with MeTet-alkyne

When prolonging the photoreaction with **MeTet-alkyne**, we observed a decrease in the expected modification and an increasing additional modification that could be assigned as arylation, which localised to all aromatic amino acids and to cysteines (Supplementary Fig. 38 and 40). While this chemistry might, therefore, also be interesting to monitor aromatic amino acids, care must be taken to not overextend irradiation times when monitoring aspartates and glutamates with **MeTet-alkyne**.

Arylation chemistry with diazonium salts

Aryl radicals could be formed from diazonium ions through physiological reductants such as NADH, ascorbate and glutathione (Supplementary Fig. 55).[3, 4] The reaction of such radicals has been described with cysteine[5] as well as tyrosine and tryptophan.[6]

For the example of **DA1-alkyne**, we also performed a control experiment, in which we removed excess probe before the CuAAC reaction. Also in this case, we observed the same mass of modification, selectivity and number of quantified residues. This indicates that the reactivity occurs independent of the reagents used in the CuAAC reaction.

Coverage for PCA-alkyne

PCA-alkyne is validated in this study to be a valuable starting point to selectively modify protein *N*-termini. While highly specialised methods for *N*-terminomics allow higher coverage of up to thousands of peptide *N*-termini, they

also only detect few protein *N*-termini.[7, 8] In combination with the high degree of *N*-terminal modification through *e.g.* acetylation,[9] we did not detect these rare events in the human proteome.

Comparison of quantified sites at low and high concentrations

We have studied 14 of our probes at two different concentrations (100 μM and 1 mM) in *S. aureus* lysate. Some of the probes, especially those who label less strongly, show a strong increase in quantified sites from 100 μM to 1 mM (*e.g.* 41 *vs.* 284 quantified cysteines for **Ep-alkyne** and 409 *vs.* 1544 quantified arginines for **PhGO-alkyne**). Nevertheless, many others show a more modest increase. On the one hand, especially for cysteine-directed probes like **IA-alkyne** (1197 *vs.* 1316 quantified cysteines), this might point to the fact that the detectable sites are already largely covered. This is corroborated by the observation that the overlap of the quantified sites for the cysteine-directed probes is high (Supplementary Fig. 14). On the other hand, across all data sets the maximum number of quantified sites in one experiment is in the range of 5000 sites for any of our probes. This suggests that our workflow with the used instrumentation cannot detect much more sites than this. Therefore, probes like **STP-alkyne** that already quantify many sites at 100 μM will not show a strong increase at 1 mM as the number of sites is already largely saturated (4022 *vs.* 4990 quantified sites).

Use of the probes in cells

We monitored eight of the nine amino acids as well as the protein *N*-terminus also with probe treatment in cells. Especially, **MeTet-**, **Az-**, **EBA-**, **ArSq-**, **SuTEx2-** and **PTAD-alkyne** also performed very well, making these chemotypes very promising for studies in cells. While for cellular engagement studies of irreversible covalent inhibitors the cells can be lysed before probe addition, using these probes in cells will be especially useful to identify the cellular targets for competitors that give unstable or reversible adducts and for whom cells cannot be lysed before treatment with the probe without the risk of altering the engaged residues upon lysis. While these initial results are very promising, cellular toxicity and probe uptake need to be carefully evaluated for such applications.

Supplementary Figures

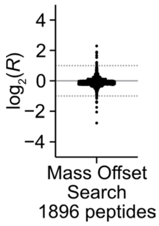

Supplementary Figure 1 | Quantification of a Mass Offset Search. Labelling of the proteome of *S. aureus* SH1000 with 1 mM **IA-alkyne** and analysis using the isoDTB-ABPP workflow[10] results in MS data that was analysed using MSFragger[11, 12] Mass Offset Search and IonQuant[13] labelling-based quantification. The mass offsets correspond to the expected modification by alkylation (Δm_{exp}, Fig. 2). Here, the dataset was analysed, in which the heavy and light samples were mixed at a ratio of 1:1. The grey, solid line indicates the expected value of $\log_2(R) = 0$ The grey, dashed lines indicate the preferred window of quantification ($-1 < \log_2(R) < 1$). The mass spectrometric experiments used for this analysis were performed in an earlier study.[10] All data is based on technical duplicates.

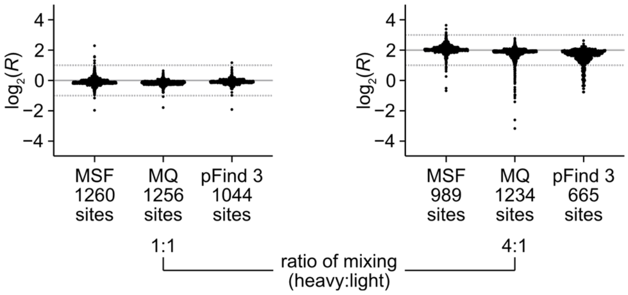

Supplementary Figure 2 | Comparison of different quantification tools. Labelling of the proteome of *S. aureus* SH1000 with 1 mM **IA-alkyne** and analysis using the isoDTB-ABPP workflow[10] results in MS data that was analysed by quantification of the expected modification by alkylation on cysteine (Δm_{exp}, Fig. 2). MSFragger (MSF)[11, 12] Closed Search and IonQuant[13] labelling-based quantification were used. MaxQuant (MQ)[14] was applied using a described workaround[10] for quantification. pFind 3[15] was applied using a custom script[16] for downstream data analysis. Here, two datasets were analysed, in which the heavy and light samples were mixed at a ratio of 1:1 (left panel) and 4:1 (right panel), respectively. The grey, solid lines indicate the expected values of $\log_2(R) = 0$ and $\log_2(R) = 2$. The grey, dashed lines indicate the respective preferred window of quantification ($-1 < \log_2(R) < 1$ for the 1:1 ratio, $1 < \log_2(R) < 3$ for the 4:1 ratio). The mass spectrometric experiments used for this analysis were performed in an earlier study.[10] All data is based on technical duplicates.

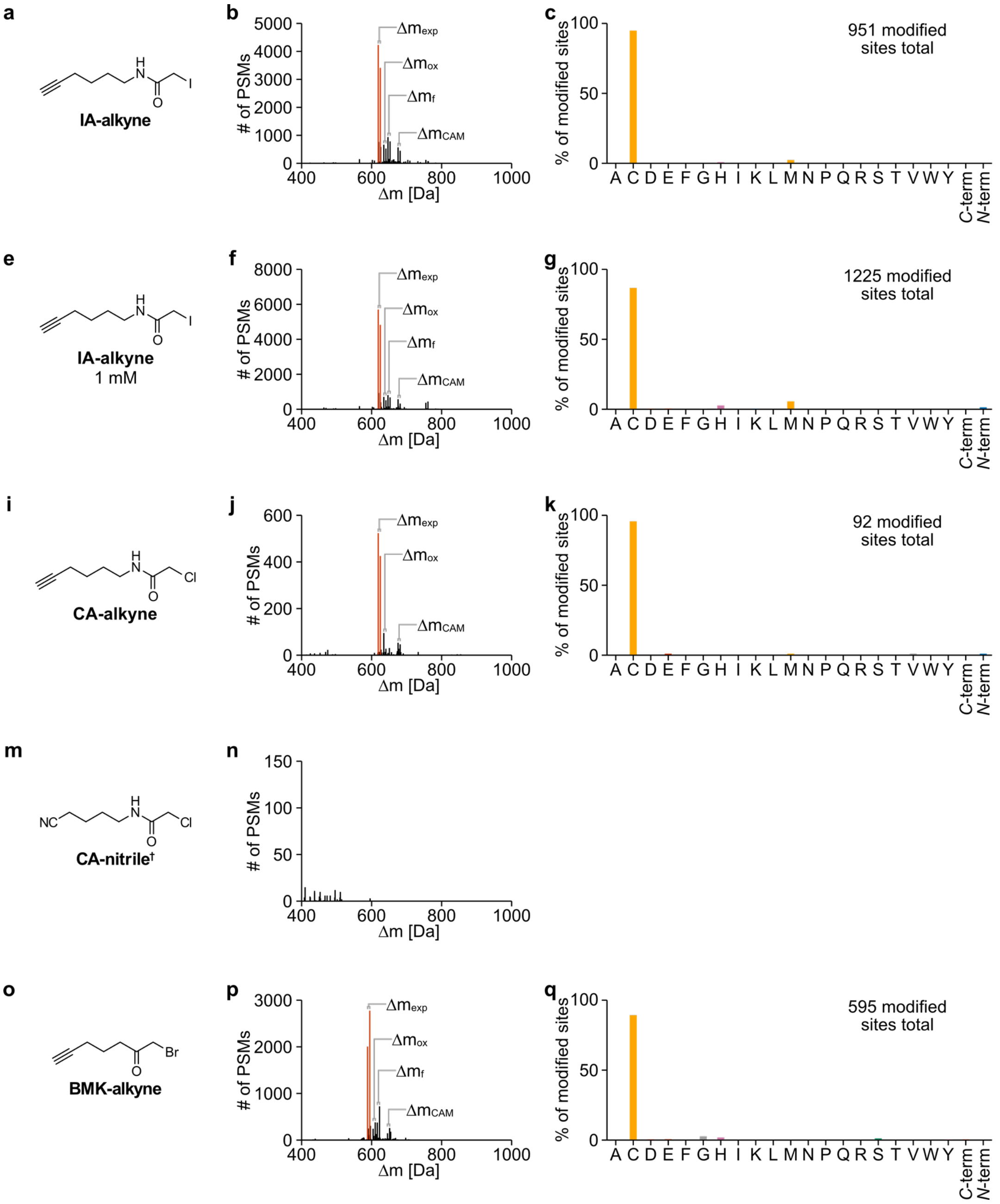

Supplementary Figure 3 | Masses of modification, amino acid selectivity and quantification for α-halocarbonyl probes. **a**,**e**,**i**,**m**,**o**, Structures of the probes that were used for treatment of the proteome of *S. aureus* SH1000 at 100 μM (**a**,**i**,**m**,**o**) or 1 mM (**e**) probe concentration. **b**,**f**,**j**,**n**,**p**, Masses of modification determined through analysis with an Open Search in MSFragger[11, 12]-based FragPipe. The peaks highlighted in red are the masses that have been selected for further analysis within the same row. The expected masses are labelled as Δm_{exp}. Further modification of the modified peptides by oxidation (Δm_{ox}), formylation (Δm_{f}) or carbamidomethylation on a second cysteine (Δm_{CAM}) is also indicated if the respective peaks were detected. **c**,**g**,**k**,**q**, One peak pair (indicated in red in the same row) is selected for a Mass Offset Search[11] that localizes this modification to the modified amino acid(s). In this way, selectivity is assessed across all proteinogenic amino acids. The bar graph represents the fraction of all modified sites that is modified at the indicated amino acid. *C*-term = *C*-terminal modification. *N*-term = *N*-terminal modification. **d**,**h**,**l**,**r**, Specific amino acid(s) are selected for quantification at the selected masses (indicated in red in the same row) using a Closed Search[11] and the IonQuant[13] feature. The heavy and light samples were mixed at a ratio of 1:1. The grey, solid line indicates the expected values of $\log_2(R) = 0$. The grey, dashed lines indicate the preferred window of quantification ($-1 < \log_2(R) < 1$). All data is based on technical duplicates. †: No clear mass of modification was detected and therefore no analysis of the amino acid selectivity was possible.

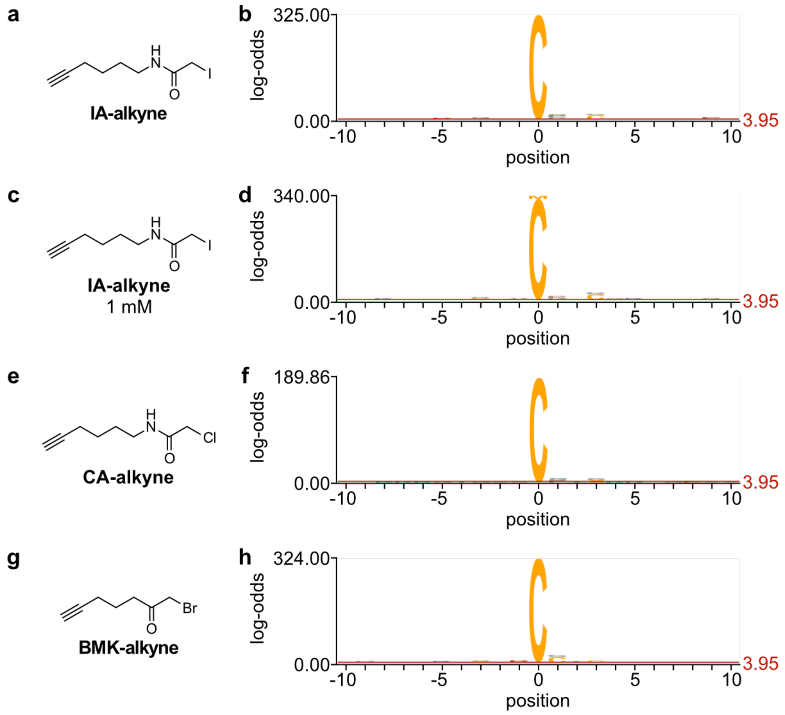

Supplementary Figure 4 | Sequence logos around sites modified by IA-, CA-, and BMK-alkyne. a,**c**,**e**,**g**, Structures of the probes that were used for treatment of the proteome of *S. aureus* SH1000 at 100 μM (**a**,**e**,**g**) or 1 mM (**c**) probe concentration. **b**,**d**,**f**,**h**, Sequence logo for all modified sequences determined using pLogo[17] based on the modified sites identified in the Mass Offset Searches. Position 0 indicates the modified site. The y-axis shows the log-odds of the binomial probability (log-odds) for the shown amino acids. The red line indicates the cut-off of $p < 0.05$. X indicates a residue that is outside of the sequence of the protein (before the *N*-terminus for positions −10 to −1 or after the *C*-terminus for positions 1 to 10) All data is based on technical duplicates.

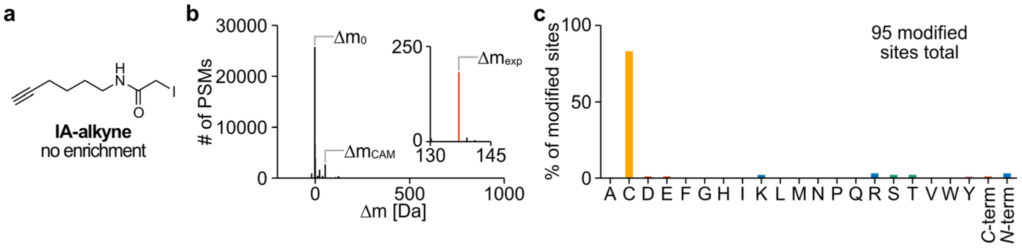

Supplementary Figure 5 | Masses of modification and amino acid selectivity for IA-alkyne without clicking to isoDTB tags and without enrichment. a, Structure of the probe that was used for treatment of *S. aureus* SH1000 at 100 μM. **b**, Masses of modification determined through analysis with an Open Search in MSFragger[11, 12]-based FragPipe. The peak highlighted in red is the mass that has been selected for selectivity analysis. The expected mass is labelled as Δm_{exp}. Modification by carbamidomethylation (Δm_{CAM}) and unmodified peptides (Δm_0)are also indicated. **c**, One peak (indicated in red) is selected for a Mass Offset Search[11] that localizes this modification to the modified amino acid(s). In this way, selectivity is assessed across all proteinogenic amino acids. The bar graph represents the fraction of all modified sites that is modified at the indicated amino acid. *C*-term = *C*-terminal modification. *N*-term = *N*-terminal modification. All data is based on technical duplicates.

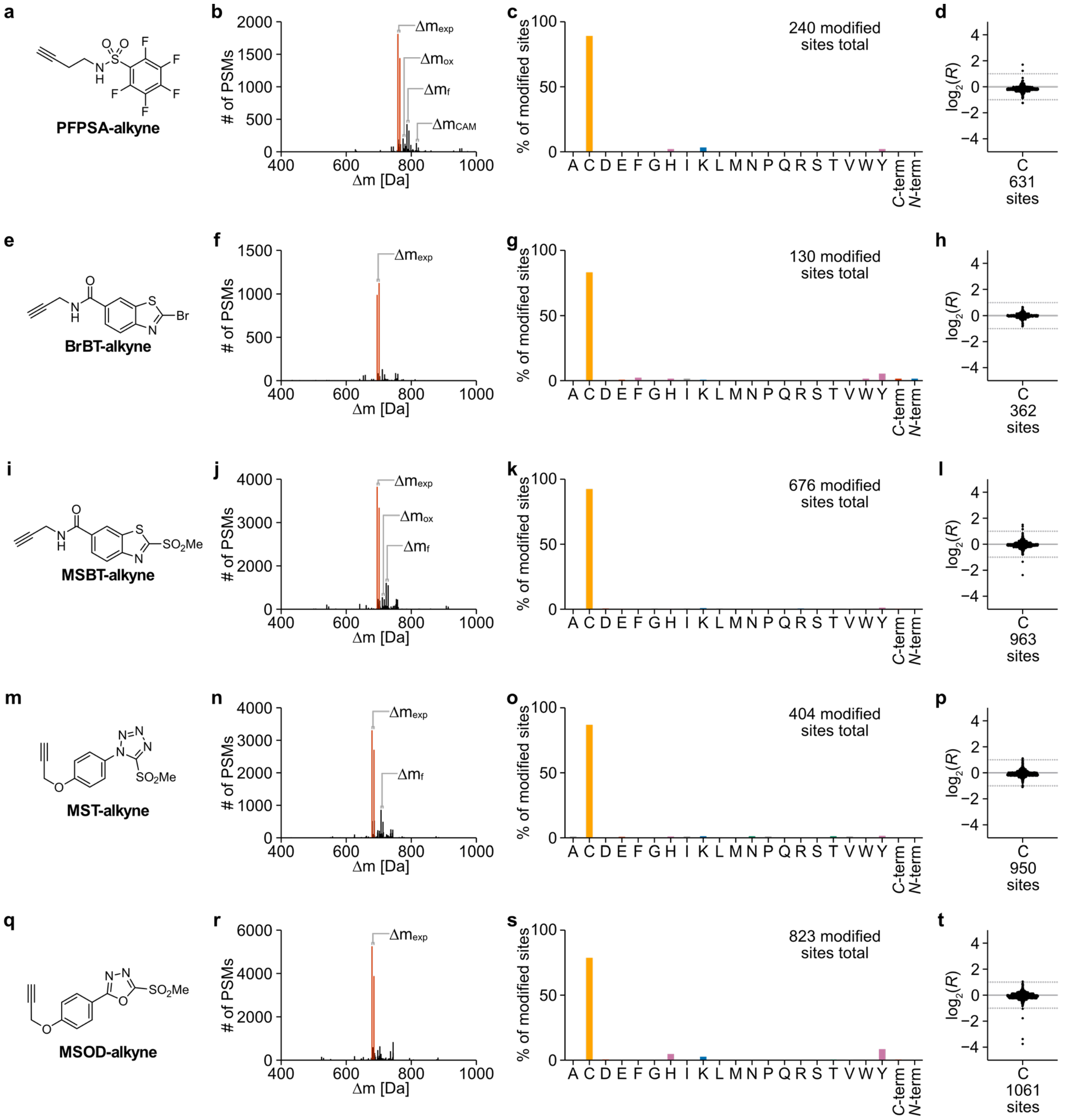

Supplementary Figure 6 | Masses of modification, amino acid selectivity and quantification for probes reacting through nucleophilic aromatic substitution. **a**,**e**,**i**,**m**,**q**, Structures of the probes that were used for treatment of the proteome of *S. aureus* SH1000 at 100 μM probe concentration. **b**,**f**,**j**,**n**,**r**, Masses of modification determined through analysis with an Open Search in MSFragger[11, 12]-based FragPipe. The peaks highlighted in red are the masses that have been selected for further analysis within the same row. The expected masses are labelled as Δm_{exp}. Further modification of the modified peptides by oxidation (Δm_{ox}), formylation (Δm_{f}) or carbamidomethylation on a second cysteine (Δm_{CAM}) is also indicated if the respective peaks were detected. **c**,**g**,**k**,**o**,**s**, One peak pair (indicated in red in the same row) is selected for a Mass Offset Search[11] that localizes this modification to the modified amino acid(s). In this way, selectivity is assessed across all proteinogenic amino acids. The bar graph represents the fraction of all modified sites that is modified at the indicated amino acid. *C*-term = *C*-terminal modification. *N*-term = N-terminal modification. **d**,**h**,**l**,**p**,**t**, Specific amino acid(s) are selected for quantification at the selected masses (indicated in red in the same row) using a Closed Search[11] and the IonQuant[13] feature. The heavy and light samples were mixed at a ratio of 1:1. The grey, solid line indicates the expected values of $\log_2(R) = 0$. The grey, dashed lines indicate the preferred window of quantification ($-1 < \log_2(R) < 1$). All data is based on technical duplicates.

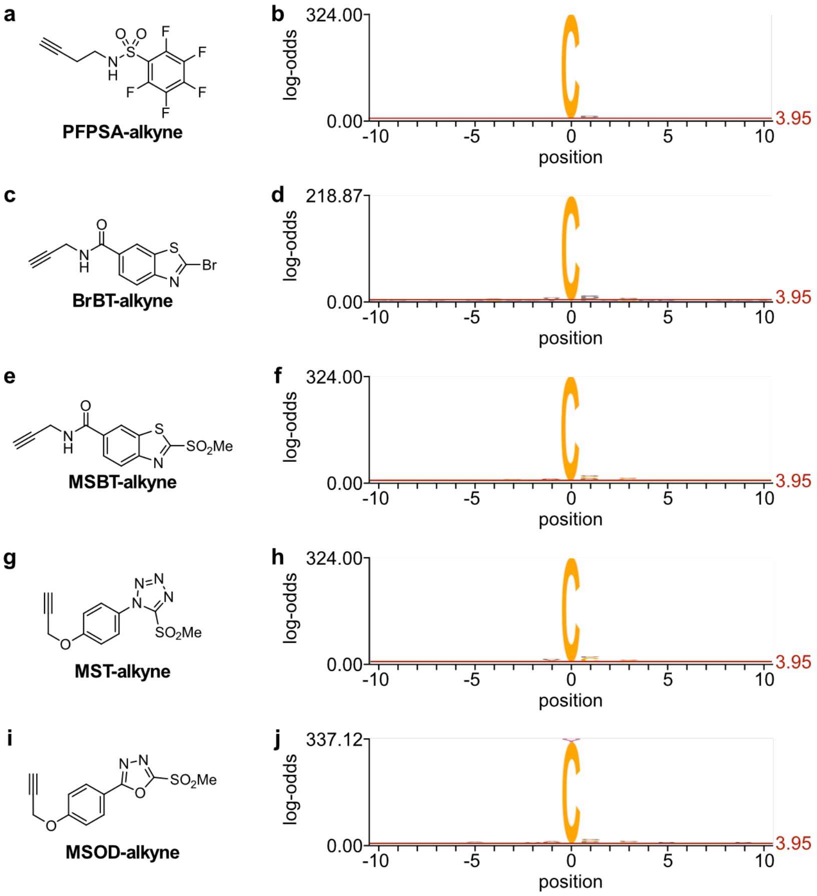

Supplementary Figure 7 | Sequence logos around sites modified by PFPSA-, BrBT-, MSBT-, MST-, and MSOD-alkyne. a,**c**,**e**,**g**,**i**, Structures of the probes that were used for treatment of the proteome of *S. aureus* SH1000 at 100 μM probe concentration. **b**,**d**,**f**,**h**,**j**, Sequence logo for all modified sequences determined using pLogo[17] based on the modified sites identified in the Mass Offset Searches. Position 0 indicates the modified site. The y-axis shows the log-odds of the binomial probability (log-odds) for the shown amino acids. The red line indicates the cut-off of $p < 0.05$. X indicates a residue that is outside of the sequence of the protein (before the *N*-terminus for positions −10 to −1 or after the *C*-terminus for positions 1 to 10) All data is based on technical duplicates.

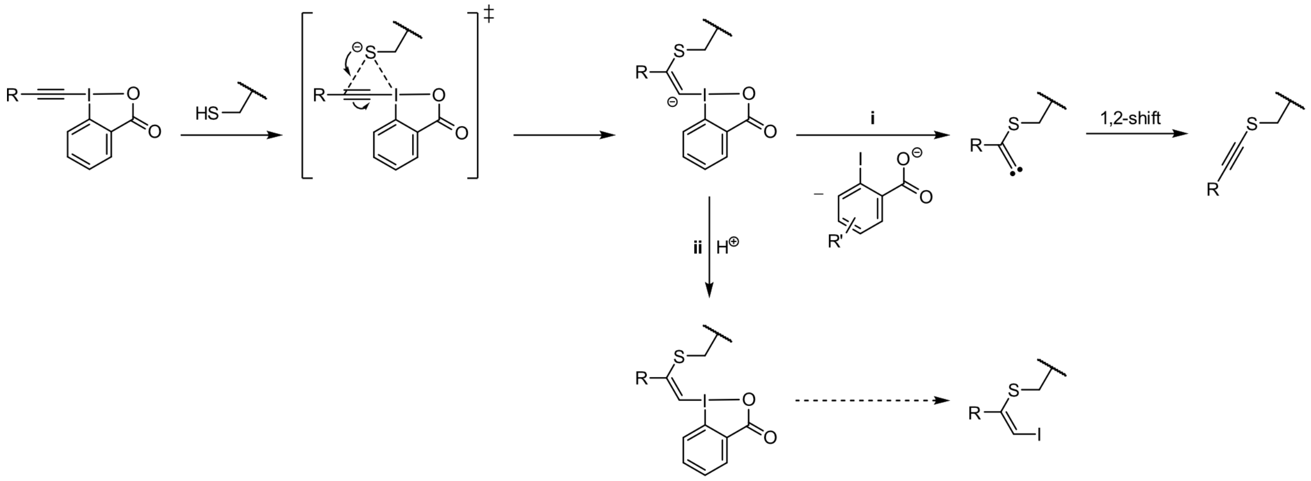

Supplementary Figure 8 | Mechanism of cysteine-labelling with hypervalent iodine reagent EBX1-alkyne as described by Frei *et al.*[18] After addition of cysteine, the vinylic carbanion can either undergo α-elimination followed by a 1,2-shift (**i**) to result in formal alkynylation or get protonated (**ii**) to produce a vinylbenziodoxolone (VBX),[19] which could potentially further react to the corresponding vinyl iodide.[20, 21]

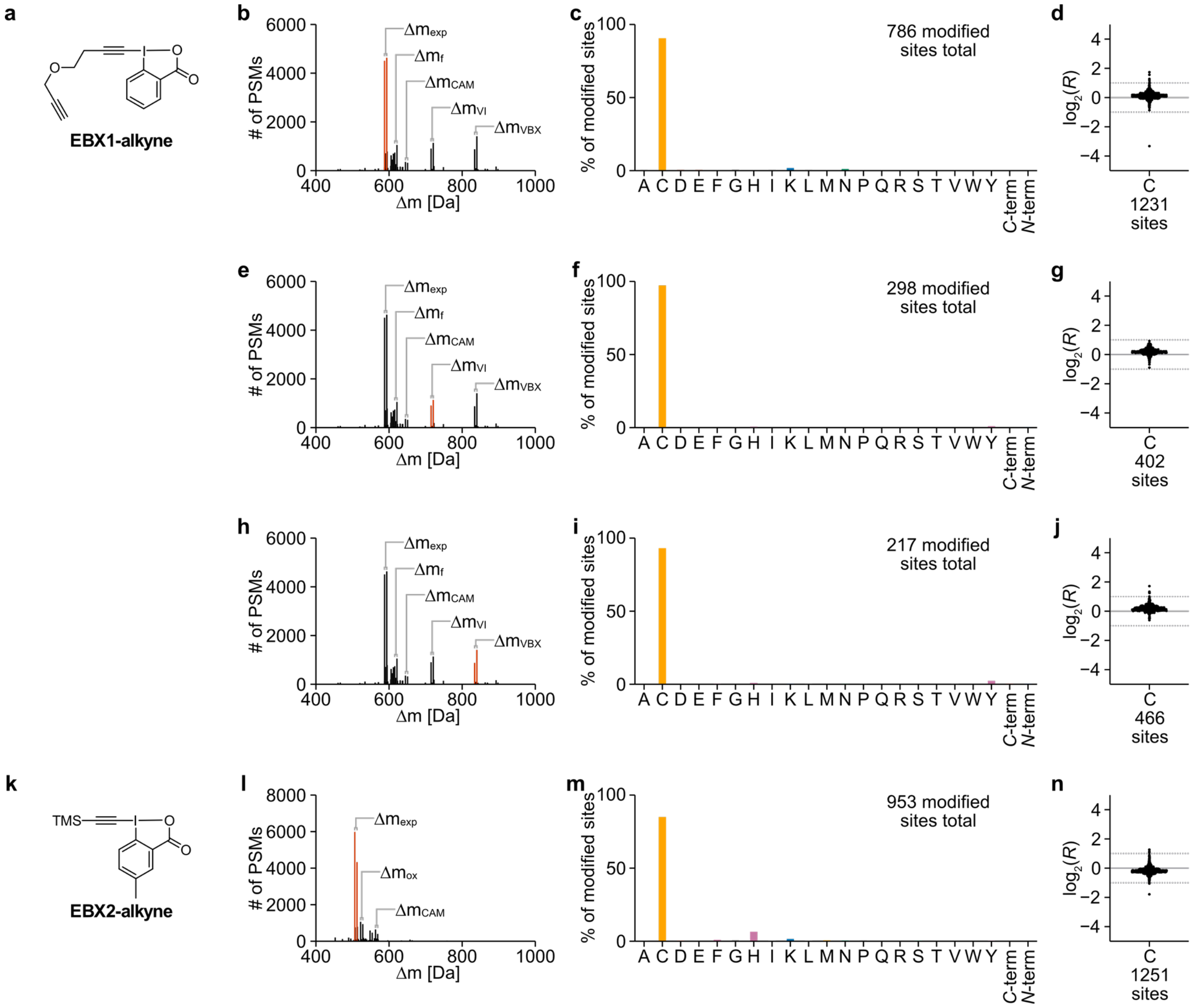

Supplementary Figure 9 | Masses of modification, amino acid selectivity and quantification for probes containing hypervalent iodine. **a**,**k**, Structures of the probes that were used for treatment of the proteome of *S. aureus* SH1000 at 100 μM probe concentration. **b**,**e**,**h**,**l**, Masses of modification determined through analysis with an Open Search in MSFragger[11, 12]-based FragPipe. The peaks highlighted in red are the masses that have been selected for further analysis within the same row. The expected masses are labelled as Δm_{exp}. The additionally identified reactivities resulting in VBX modification (Δm_{VBX}) and vinyl iodide formation (Δm_{VI}) are also highlighted. Further modification of the modified peptides by oxidation (Δm_{ox}), formylation (Δm_{f}) or carbamidomethylation on a second cysteine (Δm_{CAM}) is also indicated if the respective peaks were detected. **c**,**f**,**i**,**m**, One peak pair (indicated in red in the same row) is selected for a Mass Offset Search[11] that localizes this modification to the modified amino acid(s). In this way, selectivity is assessed across all proteinogenic amino acids. The bar graph represents the fraction of all modified sites that is modified at the indicated amino acid. *C*-term = *C*-terminal modification. *N*-term = *N*-terminal modification. **d**,**g**,**j**,**n**, Specific amino acid(s) are selected for quantification at the selected masses (indicated in red in the same row) using a Closed Search[11] and the IonQuant[13] feature. The heavy and light samples were mixed at a ratio of 1:1. The grey, solid line indicates the expected values of $\log_2(R) = 0$. The grey, dashed lines indicate the preferred window of quantification ($-1 < \log_2(R) < 1$). All data is based on technical duplicates.

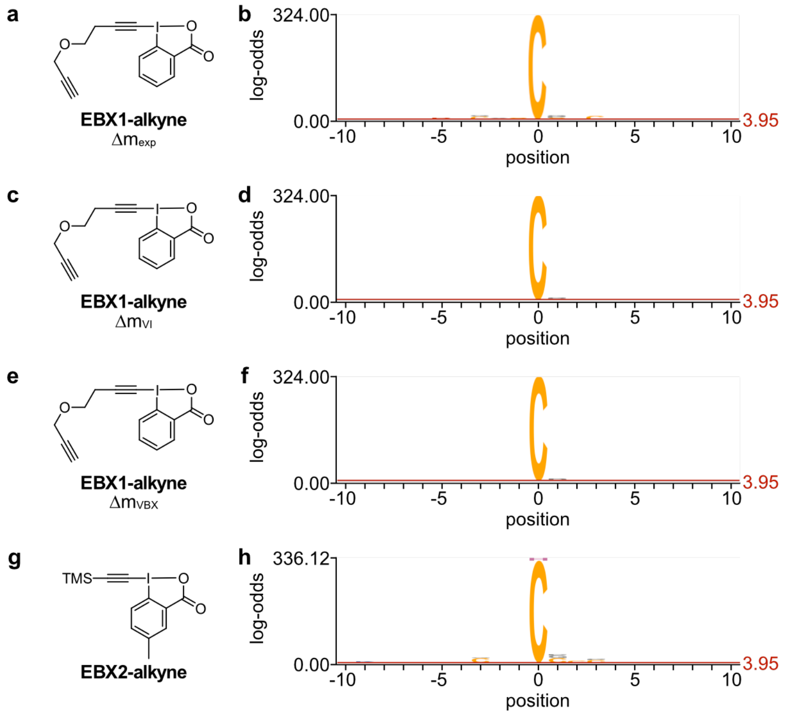

Supplementary Figure 10 | Sequence logos around sites modified by EBX1- and EBX2-alkyne. a,**d**,**g**,**j**, Structures of the probes that were used for treatment of the proteome of *S. aureus* SH1000 at 100 μM probe concentration. For **EBX1-alkyne**, vinyliodide (**c**) and VBX (**e**) were detected as additional modifications. **b**,**d**,**f**,**h**, Sequence logo for all modified sequences determined using pLogo[17] based on the modified sites identified in the Mass Offset Searches. Position 0 indicates the modified site. The y-axis shows the log-odds of the binomial probability (log-odds) for the shown amino acids. The red line indicates the cut-off of $p < 0.05$. X indicates a residue that is outside of the sequence of the protein (before the *N*-terminus for positions −10 to −1 or after the *C*-terminus for positions 1 to 10) All data is based on technical duplicates.

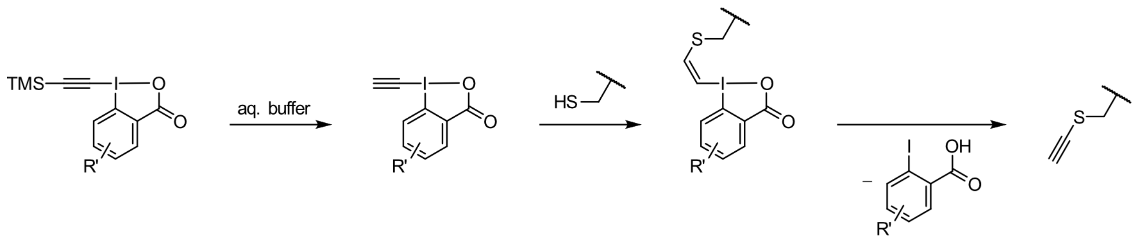

Supplementary Figure 11 | Mechanism of cysteine-labelling with hypervalent iodine reagent EBX2-alkyne as described by Tessier *et al.*[22]

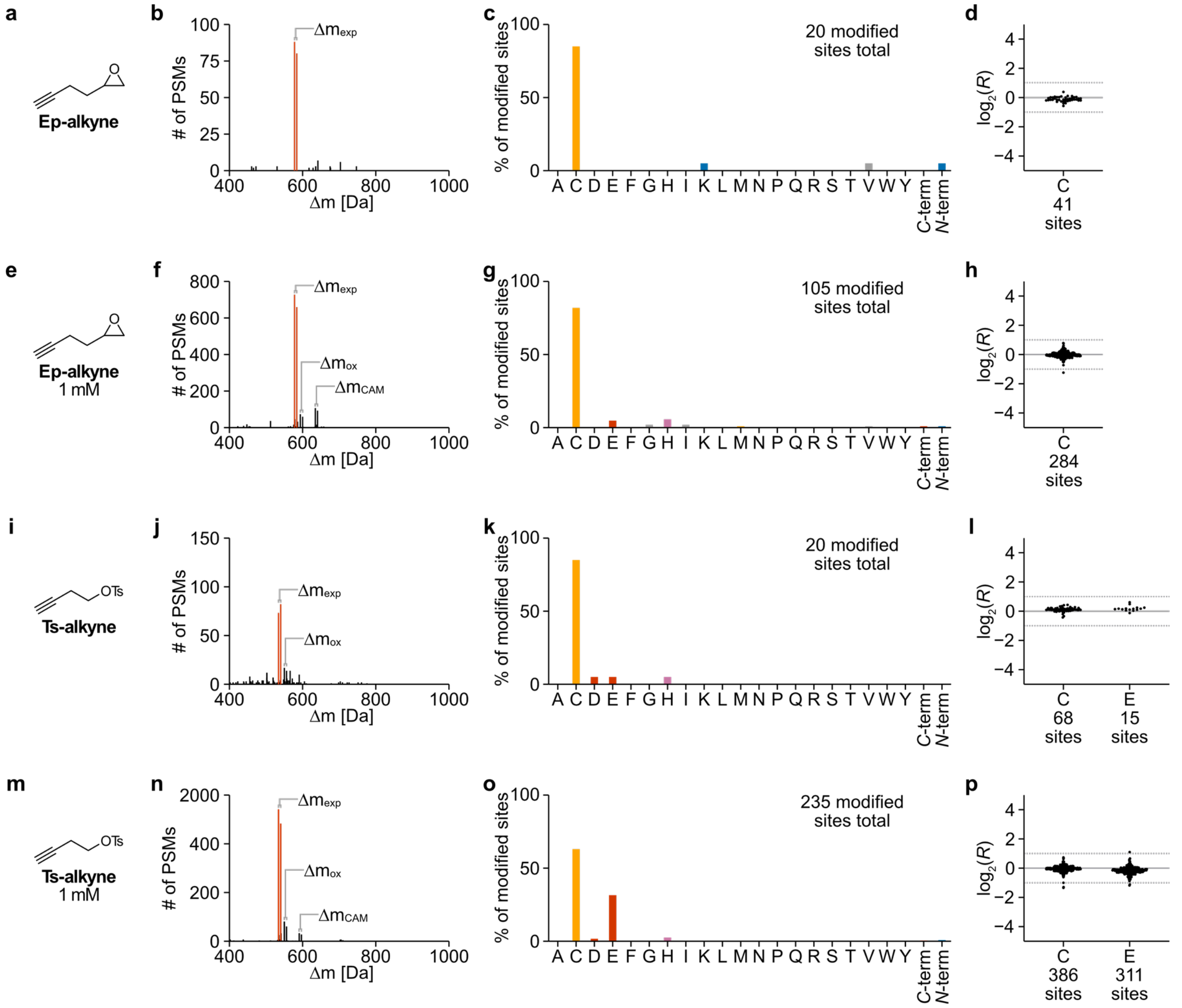

Supplementary Figure 12 | Masses of modification, amino acid selectivity and quantification for probes leading to nucleophilic substitution reactions at unactivated sp^3-carbon centres. **a**,**e**,**i**,**m**, Structures of the probes that were used for treatment of the proteome of *S. aureus* SH1000 at 100 μM (**a**,**i**) or 1 mM (**e**,**m**) probe concentration . **b**,**f**,**j**,**n**, Masses of modification determined through analysis with an Open Search in MSFragger[11, 12]-based FragPipe. The peaks highlighted in red are the masses that have been selected for further analysis within the same row. The expected masses are labelled as Δm_{exp}. Further modification of the modified peptides by oxidation (Δm_{ox}) or carbamidomethylation on a second cysteine (Δm_{CAM}) is also indicated if the respective peaks were detected. **c**,**g**,**k**,**o**, One peak pair (indicated in red in the same row) is selected for a Mass Offset Search[11] that localizes this modification to the modified amino acid(s). In this way, selectivity is assessed across all proteinogenic amino acids. The bar graph represents the fraction of all modified sites that is modified at the indicated amino acid. *C*-term = *C*-terminal modification. *N*-term = *N*-terminal modification. **d**,**h**,**l**,**p**, Specific amino acid(s) are selected for quantification at the selected masses (indicated in red in the same row) using a Closed Search[11] and the IonQuant[13] feature. The heavy and light samples were mixed at a ratio of 1:1. The grey, solid line indicates the expected values of $\log_2(R) = 0$. The grey, dashed lines indicate the preferred window of quantification ($-1 < \log_2(R) < 1$). All data is based on technical duplicates.

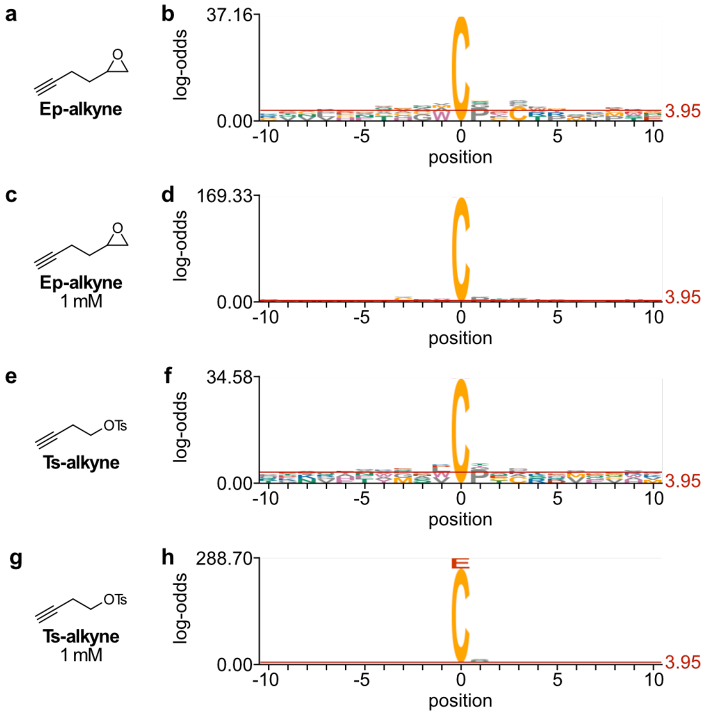

Supplementary Figure 13 | Sequence logos around sites modified by Ep-, and Ts-alkyne. a,**c**,**e**,**g**, Structures of the probes that were used for treatment of the proteome of *S. aureus* SH1000 at 100 μM (**a**,**e**) or 1 mM (**c**,**g**) probe concentration. **b**,**d**,**f**,**h**, Sequence logo for all modified sequences determined using pLogo[17] based on the modified sites identified in the Mass Offset Searches. Position 0 indicates the modified site. The y-axis shows the log-odds of the binomial probability (log-odds) for the shown amino acids. The red line indicates the cut-off of $p < 0.05$. X indicates a residue that is outside of the sequence of the protein (before the *N*-terminus for positions −10 to −1 or after the *C*-terminus for positions 1 to 10) All data is based on technical duplicates.

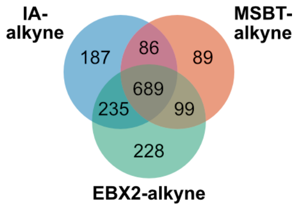

Supplementary Figure 14 | Venn-diagram of the quantified cysteines with IA-, MSBT- and EBX2-alkyne. Data is based on treatment of the proteome of *S. aureus* SH1000 at 100 μM probe concentration. All data is based on technical duplicates.

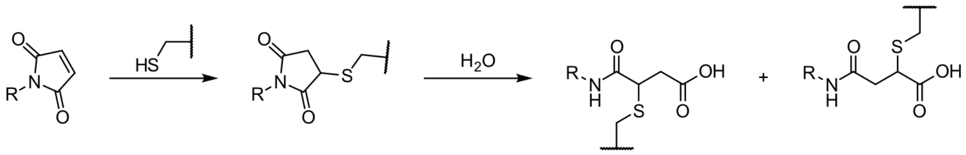

Supplementary Figure 15 | Mechanism of cysteine-labelling with maleimides (MI-alkyne) and hydrolysis of the formed adduct.[23]

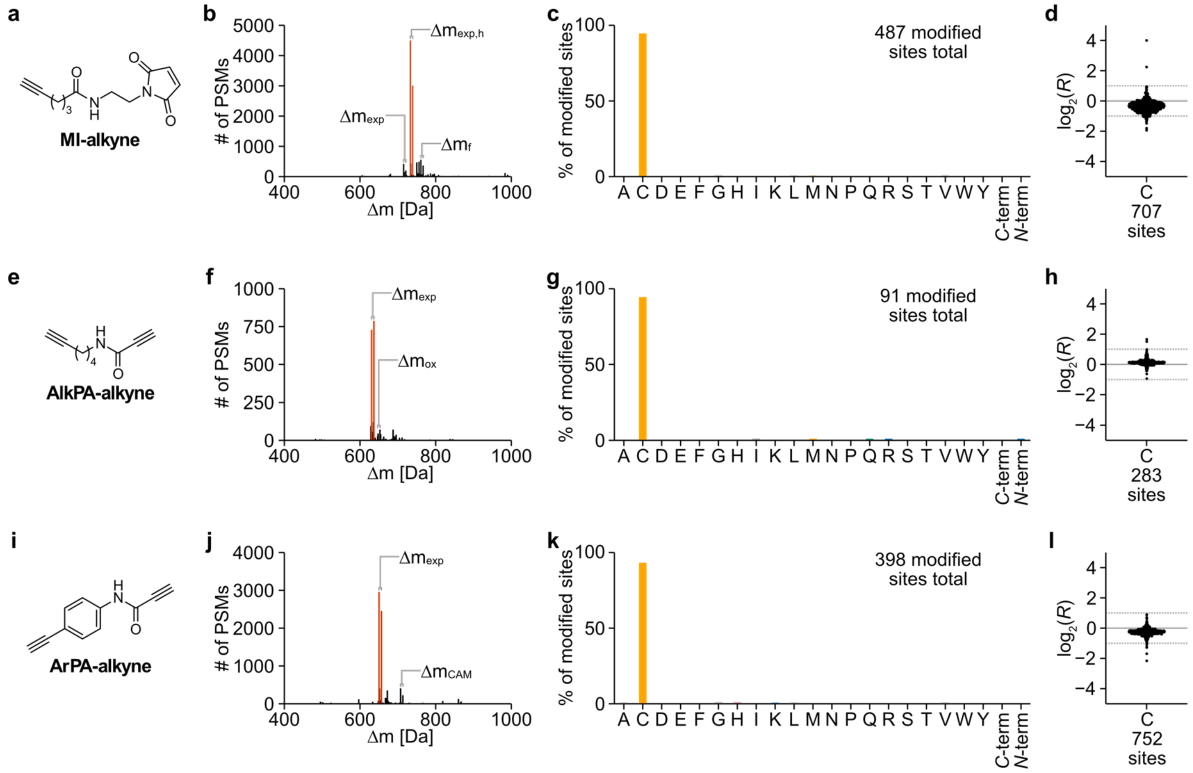

Supplementary Figure 16 | Masses of modification, amino acid selectivity and quantification for probes containing a maleimide or propiolamide reactive group. a,**e**,**i**, Structures of the probes that were used for treatment of the proteome of *S. aureus* SH1000 at 100 μM probe concentration. **b**,**f**,**j**, Masses of modification determined through analysis with an Open Search in MSFragger[11, 12]-based FragPipe. The peaks highlighted in red are the masses that have been selected for further analysis within the same row. The expected masses are labelled as Δm_{exp}. For **MI-alkyne**, the expected mass after hydrolysis of the initially formed succinimide is also highlighted ($\Delta m_{exp,h}$). Further modification of the modified peptides by oxidation (Δm_{ox}), formylation (Δm_{f}) or carbamidomethylation on a second cysteine (Δm_{CAM}) is also indicated if the respective peaks were detected. **c**,**g**,**k**, One peak pair (indicated in red in the same row) is selected for a Mass Offset Search[11] that localizes this modification to the modified amino acid(s). In this way, selectivity is assessed across all proteinogenic amino acids. The bar graph represents the fraction of all modified sites that is modified at the indicated amino acid. *C*-term = *C*-terminal modification. *N*-term = *N*-terminal modification. **d**,**h**,**l**, Specific amino acid(s) are selected for quantification at the selected masses (indicated in red in the same row) using a Closed Search[11] and the IonQuant[13] feature. The heavy and light samples were mixed at a ratio of 1:1. The grey, solid line indicates the expected values of $\log_2(R) = 0$. The grey, dashed lines indicate the preferred window of quantification ($-1 < \log_2(R) < 1$). All data is based on technical duplicates.

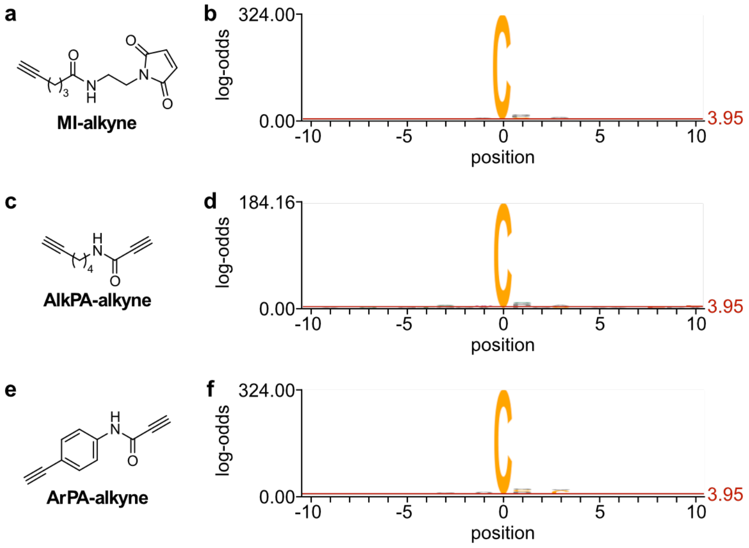

Supplementary Figure 17 | Sequence logos around sites modified by MI-, AlkPA-, and ArPA-alkyne. **a**,**c**,**e**, Structures of the probes that were used for treatment of the proteome of *S. aureus* SH1000 at 100 μM probe concentration. **b**,**d**,**f**, Sequence logo for all modified sequences determined using pLogo[17] based on the modified sites identified in the Mass Offset Searches. Position 0 indicates the modified site. The y-axis shows the log-odds of the binomial probability (log-odds) for the shown amino acids. The red line indicates the cut-off of $p < 0.05$. X indicates a residue that is outside of the sequence of the protein (before the *N*-terminus for positions −10 to −1 or after the *C*-terminus for positions 1 to 10) All data is based on technical duplicates.

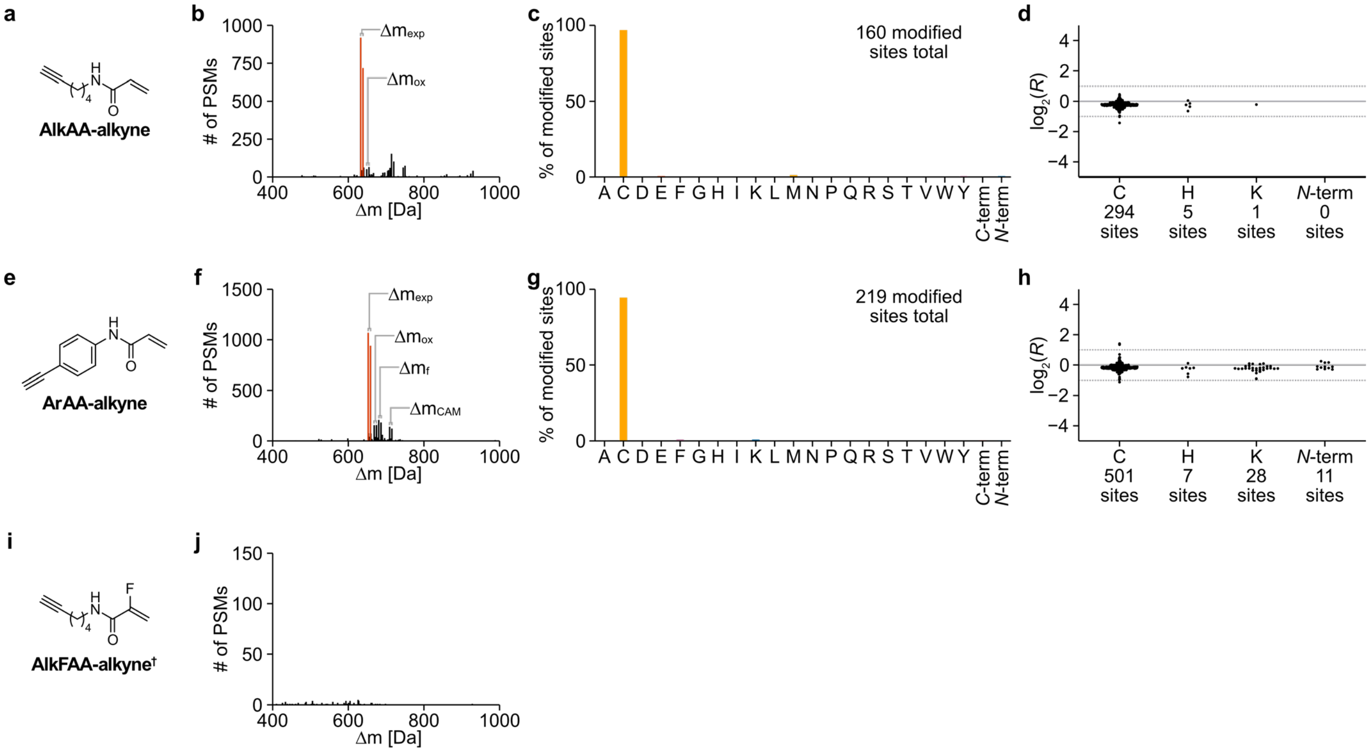

Supplementary Figure 18 | Masses of modification, amino acid selectivity and quantification for acrylamide probes. **a**,**e**,**i**, Structures of the probes that were used for treatment of the proteome of *S. aureus* SH1000 at 100 μM probe concentration. **b**,**f**,**j**, Masses of modification determined through analysis with an Open Search in MSFragger[11, 12]-based FragPipe. The peaks highlighted in red are the masses that have been selected for further analysis within the same row. The expected masses are labelled as Δm_{exp}. Further modification of the modified peptides by oxidation (Δm_{ox}), formylation (Δm_{f}) or carbamidomethylation on a second cysteine (Δm_{CAM}) is also indicated if the respective peaks were detected. **c**,**g**, One peak pair (indicated in red in the same row) is selected for a Mass Offset Search[11] that localizes this modification to the modified amino acid(s). In this way, selectivity is assessed across all proteinogenic amino acids. The bar graph represents the fraction of all modified sites that is modified at the indicated amino acid. *C*-term = *C*-terminal modification. *N*-term = *N*-terminal modification. **d**,**h**, Specific amino acid(s) are selected for quantification at the selected masses (indicated in red in the same row) using a Closed Search[11] and the IonQuant[13] feature. The heavy and light samples were mixed at a ratio of 1:1. The grey, solid line indicates the expected values of $\log_2(R) = 0$. The grey, dashed lines indicate the preferred window of quantification ($-1 < \log_2(R) < 1$). All data is based on technical duplicates. †: No clear mass of modification was detected and therefore no analysis of the amino acid selectivity was possible.

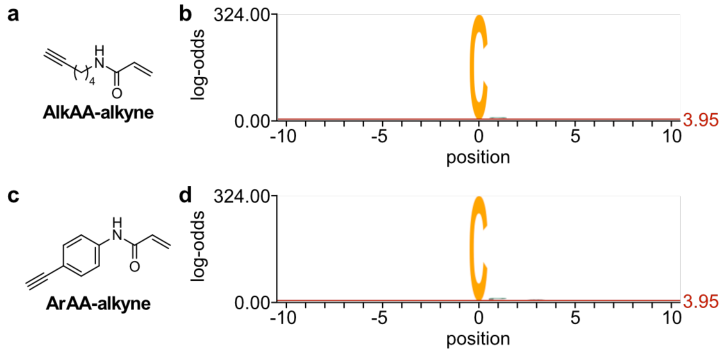

Supplementary Figure 19 | Sequence logos around sites modified by AlkAA- and ArAA-alkyne. a,**c**, Structures of the probes that were used for treatment of the proteome of *S. aureus* SH1000 at 100 μM probe concentration. **b**,**d**, Sequence logo for all modified sequences determined using pLogo[17] based on the modified sites identified in the Mass Offset Searches. Position 0 indicates the modified site. The y-axis shows the log-odds of the binomial probability (log-odds) for the shown amino acids. The red line indicates the cut-off of $p < 0.05$. X indicates a residue that is outside of the sequence of the protein (before the *N*-terminus for positions −10 to −1 or after the *C*-terminus for positions 1 to 10) All data is based on technical duplicates.

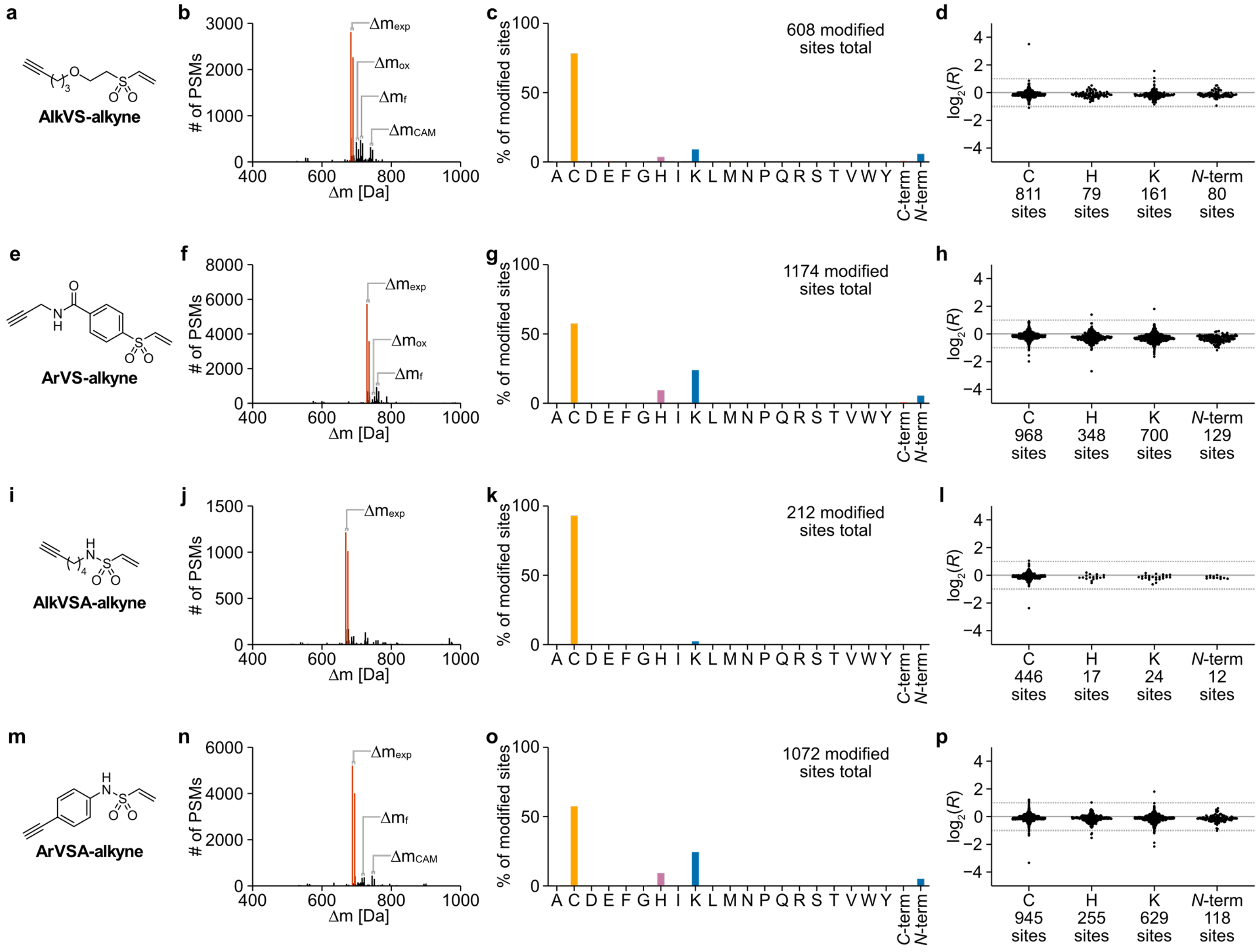

Supplementary Figure 20 | Masses of modification, amino acid selectivity and quantification for probes containing other acceptor-substituted terminal alkenes. **a**,**e**,**i**,**m**, Structures of the probes that were used for treatment of the proteome of *S. aureus* SH1000 at 100 µM probe concentration. **b**,**f**,**j**,**n**, Masses of modification determined through analysis with an Open Search in MSFragger[11, 12]-based FragPipe. The peaks highlighted in red are the masses that have been selected for further analysis within the same row. The expected masses are labelled as Δm_{exp}. Further modification of the modified peptides by oxidation (Δm_{ox}), formylation (Δm_{f}) or carbamidomethylation on a second cysteine (Δm_{CAM}) is also indicated if the respective peaks were detected. **c**,**g**,**k**,**o**, One peak pair (indicated in red in the same row) is selected for a Mass Offset Search[11] that localizes this modification to the modified amino acid(s). In this way, selectivity is assessed across all proteinogenic amino acids. The bar graph represents the fraction of all modified sites that is modified at the indicated amino acid. *C*-term = *C*-terminal modification. *N*-term = *N*-terminal modification. **d**,**h**,**l**,**p**, Specific amino acid(s) are selected for quantification at the selected masses (indicated in red in the same row) using a Closed Search[11] and the IonQuant[13] feature. The heavy and light samples were mixed at a ratio of 1:1. The grey, solid line indicates the expected values of $\log_2(R) = 0$. The grey, dashed lines indicate the preferred window of quantification ($-1 < \log_2(R) < 1$). All data is based on technical duplicates.

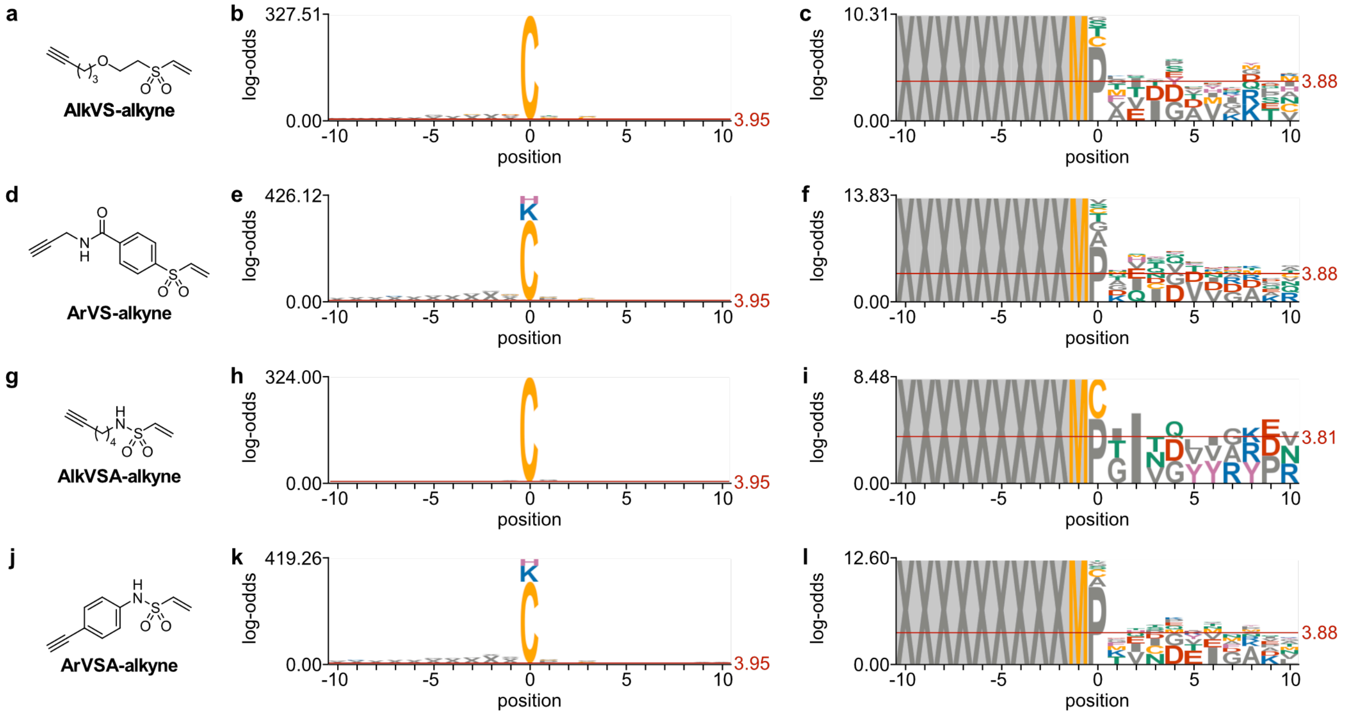

Supplementary Figure 21 | Sequence logos around sites modified by AlkVS-, ArVS-, AlkVSA-, and ArVSA-alkyne. a,**d**,**g**,**j**, Structures of the probes that were used for treatment of the proteome of *S. aureus* SH1000 at 100 μM probe concentration. **b**,**c**,**e**,**f**,**h**,**i**,**k**,**l** Sequence logos determined using pLogo[17] based on the modified sites identified in the Mass Offset Searches. Either the sequence logos for all modified sequences (**b**,**e**,**h**,**k**) or the sequence logos for a subset of sites that are modified at the protein *N*-terminus after clipping of the initial methionine (**c**,**f**,**i**,**l**) are shown. Position 0 indicates the modified site. The y-axis shows the log-odds of the binomial probability (log-odds) for the shown amino acids. The red line indicates the cut-off of $p < 0.05$. X indicates a residue that is outside of the sequence of the protein (before the *N*-terminus for positions −10 to −1 or after the *C*-terminus for positions 1 to 10) All data is based on technical duplicates.

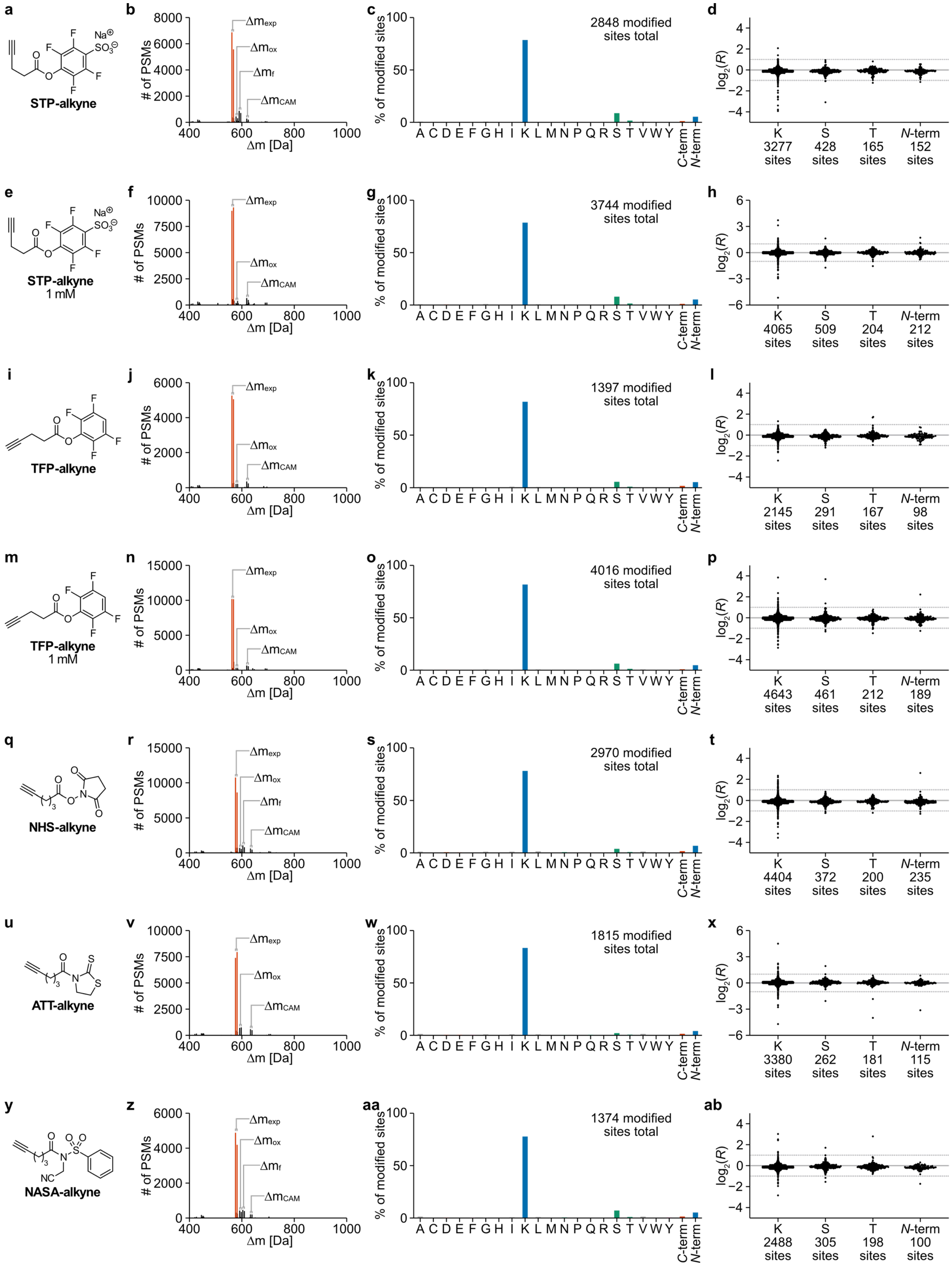

Supplementary Figure 22 | Masses of modification, amino acid selectivity and quantification for probes that are acylation reagents. a,**e**,**i**,**m**,**q**,**u**,**y**, Structures of the probes that were used for treatment of the proteome of *S. aureus* SH1000 at 100 μM (**a**,**i**,**q**,**u**,**y**) or 1 mM (**e**,**m**) probe concentration. **b**,**f**,**j**,**n**,**r**,**v**,**z**, Masses of modification

determined through analysis with an Open Search in MSFragger[11, 12]-based FragPipe. The peaks highlighted in red are the masses that have been selected for further analysis within the same row. The expected masses are labelled as Δm_{exp}. Further modification of the modified peptides by oxidation (Δm_{ox}), formylation (Δm_{f}) or carbamidomethylation on a second cysteine (Δm_{CAM}) is also indicated if the respective peaks were detected. **c**,**g**,**k**,**o**,**s**,**w**,**aa**, One peak pair (indicated in red in the same row) is selected for a Mass Offset Search[11] that localizes this modification to the modified amino acid(s). In this way, selectivity is assessed across all proteinogenic amino acids. The bar graph represents the fraction of all modified sites that is modified at the indicated amino acid. *C*-term = *C*-terminal modification. *N*-term = *N*-terminal modification. **d**,**h**,**l**,**p**,**t**,**x**,**ab**, Specific amino acid(s) are selected for quantification at the selected masses (indicated in red in the same row) using a Closed Search[11] and the IonQuant[13] feature. The heavy and light samples were mixed at a ratio of 1:1. The grey, solid line indicates the expected values of $\log_2(R) = 0$. The grey, dashed lines indicate the preferred window of quantification ($-1 < \log_2(R) < 1$). All data is based on technical duplicates.

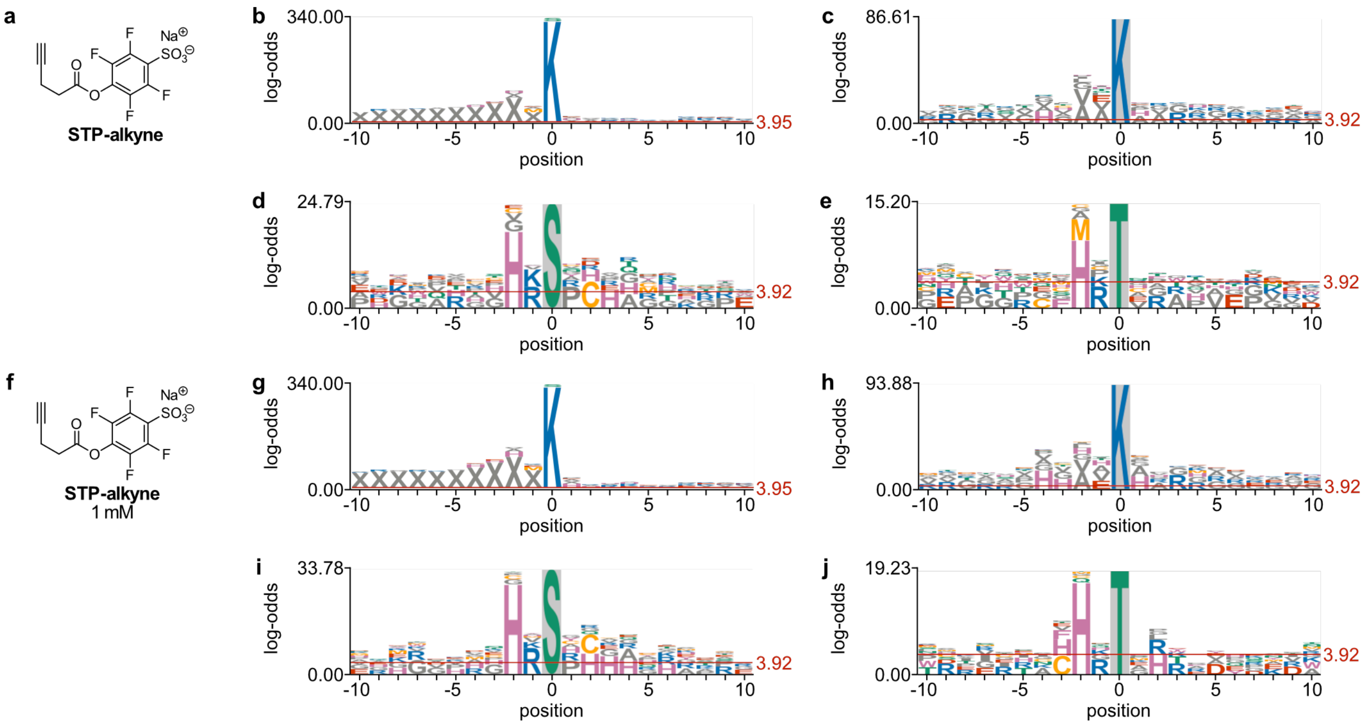

Supplementary Figure 23 | Sequence logos around sites modified by STP-alkyne. a,**f**, Structure of the probe that was used for treatment of the proteome of *S. aureus* SH1000 at 100 μM (**a**) or 1 mM (**f**) probe concentration. **b**-**e**,**g**-**j**, Sequence logos determined using pLogo[17] based on the modified sites identified in the Mass Offset Searches. Either the sequence logos for all modified sequences (**b**,**g**) or the sequence logos for a subset of sites that are modified at lysine (**b**,**g**), serine (**d**,**i**) or threonine (**e**,**j**) are shown. Position 0 indicates the modified site. The y-axis shows the log-odds of the binomial probability (log-odds) for the shown amino acids. The red line indicates the cut-off of $p < 0.05$. X indicates a residue that is outside of the sequence of the protein (before the *N*-terminus for positions −10 to −1 or after the *C*-terminus for positions 1 to 10) All data is based on technical duplicates.

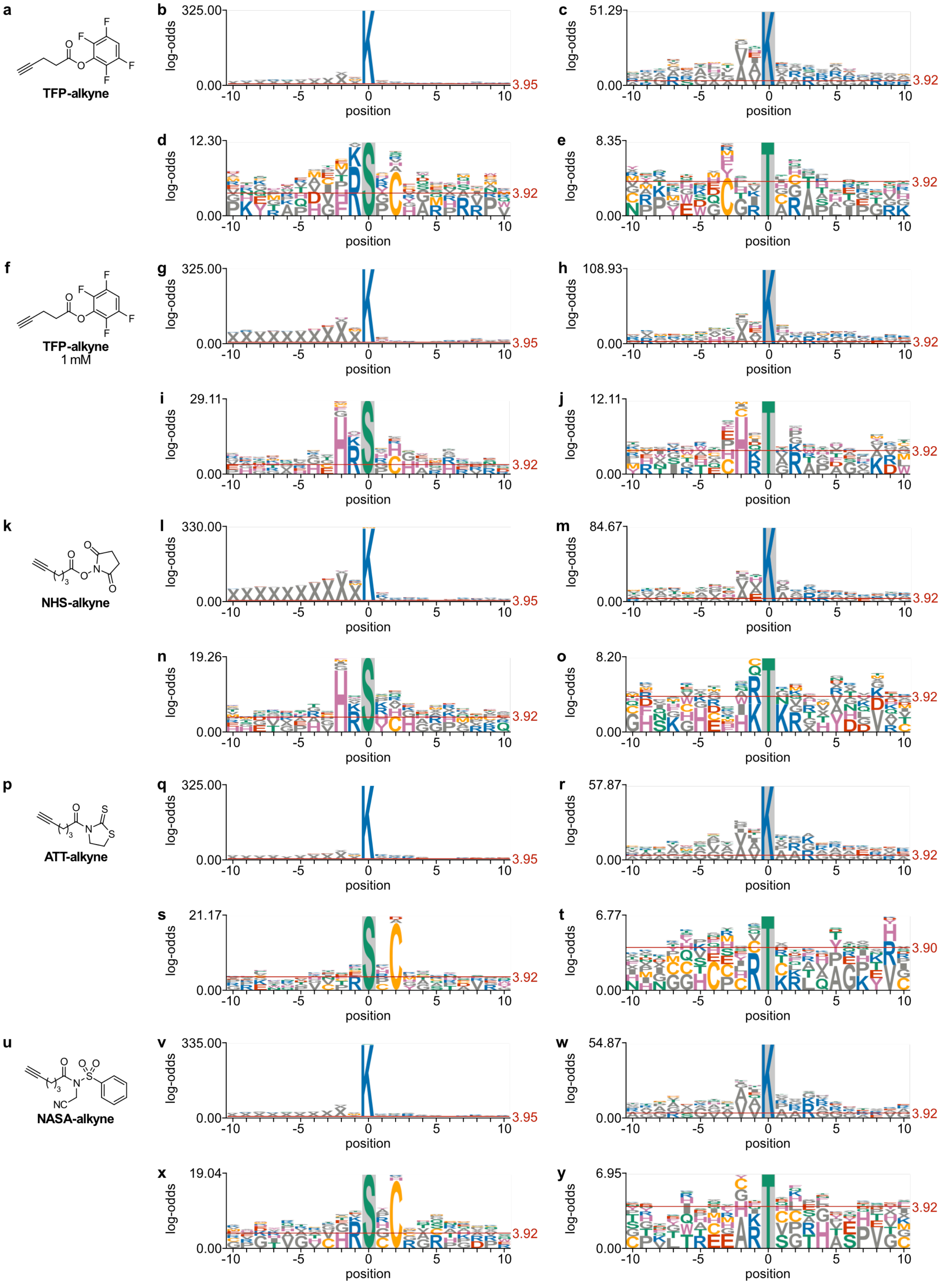

Supplementary Figure 24 | Sequence logos around sites modified by TFP-, NHS-, ATT- and NASA-alkyne. **a**,**f**,**k**,**p**,**u**, Structures of the probes that were used for treatment of the proteome of *S. aureus* SH1000 at 100 μM (**a**,**k**,**p**,**u**) or 1 mM (**f**) probe concentration. **b-e**,**g-j**,**l-o**,**q-t**,**v-y**, Sequence logos determined using pLogo[17] based on

the modified sites identified in the Mass Offset Searches. Either the sequence logos for all modified sequences (**b**,**g**,**l**,**q**,**v**) or the sequence logos for a subset of sites that are modified at lysine (**c**,**h**,**m**,**r**,**w**), serine (**d**,**i**,**n**,**s**,**x**) or threonine (**e**,**j**,**o**,**t**,**y**) are shown. Position 0 indicates the modified site. The y-axis shows the log-odds of the binomial probability (log-odds) for the shown amino acids. The red line indicates the cut-off of $p < 0.05$. X indicates a residue that is outside of the sequence of the protein (before the *N*-terminus for positions −10 to −1 or after the *C*-terminus for positions 1 to 10) All data is based on technical duplicates.

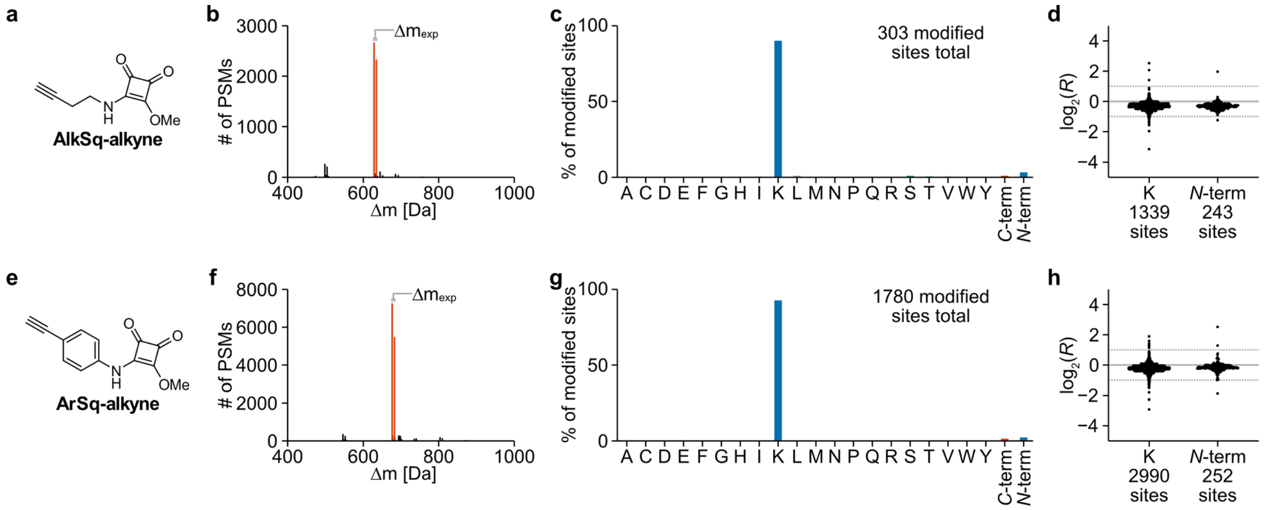

Supplementary Figure 25 | Masses of modification, amino acid selectivity and quantification for squaric acid monoamide probes. a,**e**, Structures of the probes that were used for treatment of the proteome of *S. aureus* SH1000 at 100 µM probe concentration. **b**,**f**, Masses of modification determined through analysis with an Open Search in MSFragger[11, 12]-based FragPipe. The peaks highlighted in red are the masses that have been selected for further analysis within the same row. The expected masses are labelled as Δm_{exp}. **c**,**g**, One peak pair (indicated in red in the same row) is selected for a Mass Offset Search[11] that localizes this modification to the modified amino acid(s). In this way, selectivity is assessed across all proteinogenic amino acids. The bar graph represents the fraction of all modified sites that is modified at the indicated amino acid. *C*-term = *C*-terminal modification. *N*-term = *N*-terminal modification. **d**,**h**, Specific amino acid(s) are selected for quantification at the selected masses (indicated in red in the same row) using a Closed Search[11] and the IonQuant[13] feature. The heavy and light samples were mixed at a ratio of 1:1. The grey, solid line indicates the expected values of $\log_2(R) = 0$. The grey, dashed lines indicate the preferred window of quantification ($-1 < \log_2(R) < 1$). All data is based on technical duplicates.

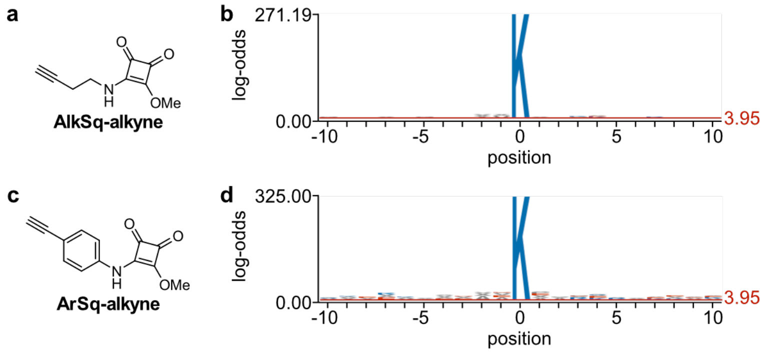

Supplementary Figure 26 | Sequence logos around sites modified by AlkSq- and ArSq-alkyne. a,**c**, Structures of the probes that were used for treatment of the proteome of *S. aureus* SH1000 at 100 µM probe concentration. **b**,**d**, Sequence logo for all modified sequences determined using pLogo[17] based on the modified sites identified in the Mass Offset Searches. Position 0 indicates the modified site. The y-axis shows the log-odds of the binomial probability (log-odds) for the shown amino acids. The red line indicates the cut-off of $p < 0.05$. X indicates a residue that is outside of the sequence of the protein (before the *N*-terminus for positions −10 to −1 or after the *C*-terminus for positions 1 to 10) All data is based on technical duplicates.

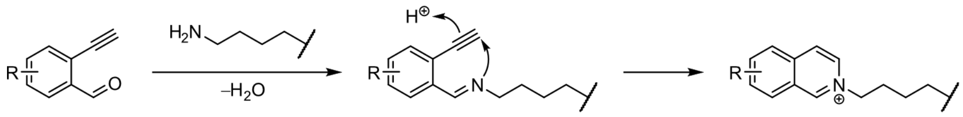

Supplementary Figure 27 | Mechanism of lysine-labelling with 2-ethynylbenzaldehydes (EBA-alkyne) through a 6-endo-*dig* cyclisation of the formed imine as described by Deng *et al.*[24]

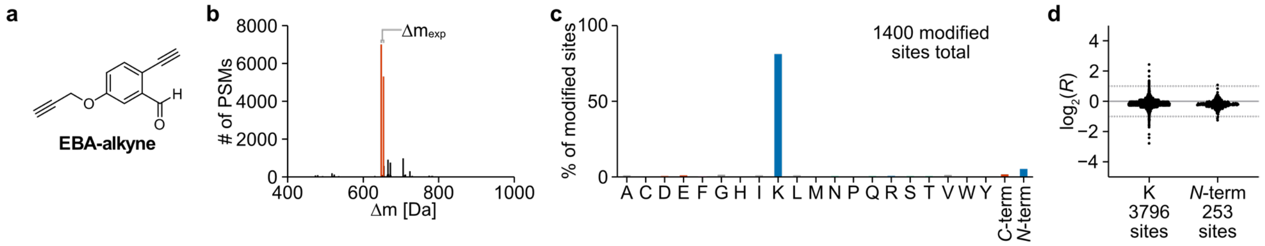

Supplementary Figure 28 | Masses of modification, amino acid selectivity and quantification for EBA-alkyne. **a**, Structure of the probe that was used for treatment of the proteome of *S. aureus* SH1000 at 100 μM probe concentration. **b**, Masses of modification determined through analysis with an Open Search in MSFragger[11, 12]-based FragPipe. The peaks highlighted in red are the masses that have been selected for further analysis. The expected masses are labelled as Δm_{exp}. **c**, One peak pair (indicated in red) is selected for a Mass Offset Search[11] that localizes this modification to the modified amino acid(s). In this way, selectivity is assessed across all proteinogenic amino acids. The bar graph represents the fraction of all modified sites that is modified at the indicated amino acid. *C*-term = *C*-terminal modification. *N*-term = *N*-terminal modification. **d**, Specific amino acid(s) are selected for quantification at the selected masses (indicated in red in the same row) using a Closed Search[11] and the IonQuant[13] feature. The heavy and light samples were mixed at a ratio of 1:1. The grey, solid line indicates the expected values of $\log_2(R) = 0$. The grey, dashed lines indicate the preferred window of quantification ($-1 < \log_2(R) < 1$). All data is based on technical duplicates.

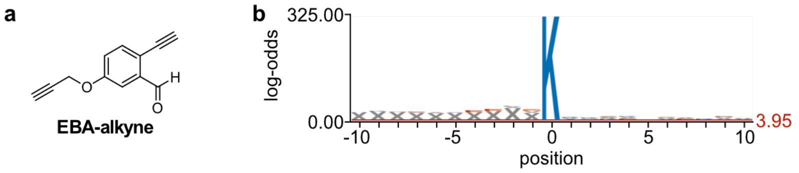

Supplementary Figure 29 | Sequence logos around sites modified by EBA-alkyne. **a**, Structure of the probe for treatment of the proteome of *S. aureus* SH1000 lysate at 100 μM probe concentration. **b**, Sequence logo for all modified sequences determined using pLogo[17] based on the modified sites identified in the Mass Offset Searches. Position 0 indicates the modified site. The y-axis shows the log-odds of the binomial probability (log-odds) for the shown amino acids. The red line indicates the cut-off of $p < 0.05$. X indicates a residue that is outside of the sequence of the protein (before the *N*-terminus for positions −10 to −1 or after the *C*-terminus for positions 1 to 10) All data is based on technical duplicates.

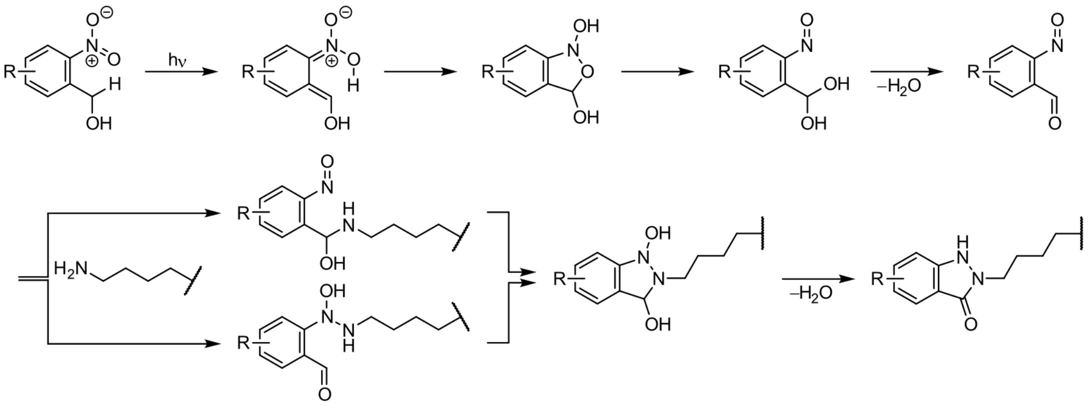

Supplementary Figure 30 | Mechanism of the photoinduced labelling of lysine with *ortho*-nitrobenzylalcohols (*o*NBA-alkyne) as described by Zhu *et al.*[25]

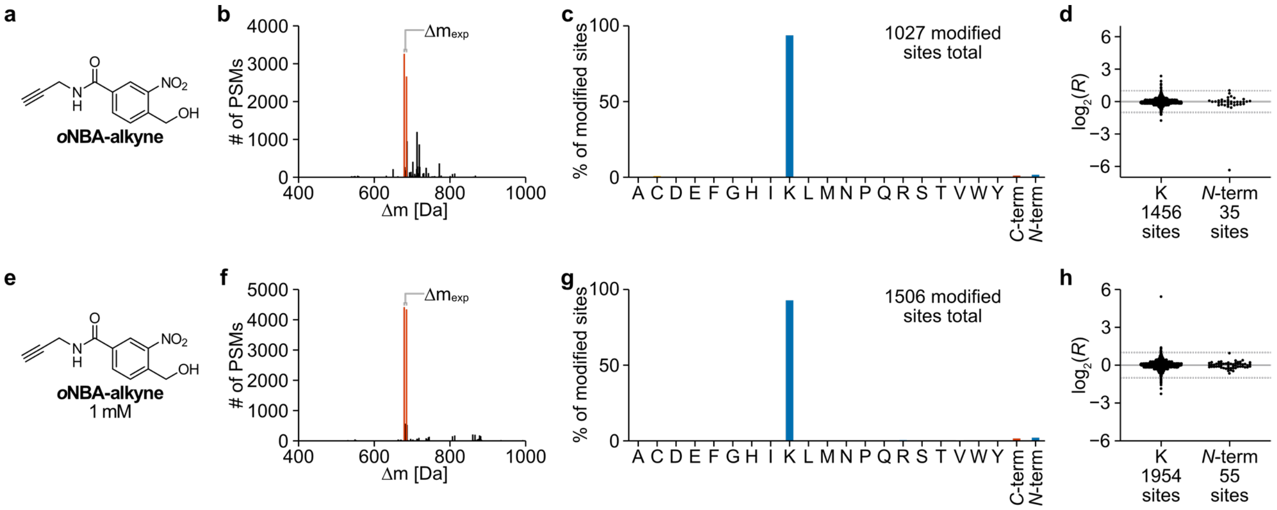

Supplementary Figure 31 | Masses of modification, amino acid selectivity and quantification for oNBA-alkyne. a,**e**, Structure of the probe that was used for treatment of the proteome of *S. aureus* SH1000 at 100 μM (**a**) or 1 mM (**e**) probe concentration. The samples were irradiated for 10 min at 365 nm after 30 min of treatment with the probe. **b**,**f**, Masses of modification determined through analysis with an Open Search in MSFragger[11, 12]-based FragPipe. The peaks highlighted in red are the masses that have been selected for further analysis within the same row. The expected masses are labelled as Δm_{exp}. **c**,**g**, One peak pair (indicated in red in the same row) is selected for a Mass Offset Search[11] that localizes this modification to the modified amino acid(s). In this way, selectivity is assessed across all proteinogenic amino acids. The bar graph represents the fraction of all modified sites that is modified at the indicated amino acid. *C*-term = *C*-terminal modification. *N*-term = *N*-terminal modification. **d**,**h**, Specific amino acid(s) are selected for quantification at the selected masses (indicated in red in the same row) using a Closed Search[11] and the IonQuant[13] feature. The heavy and light samples were mixed at a ratio of 1:1. The grey, solid line indicates the expected values of $\log_2(R) = 0$. The grey, dashed lines indicate the preferred window of quantification ($-1 < \log_2(R) < 1$). All data is based on technical duplicates.

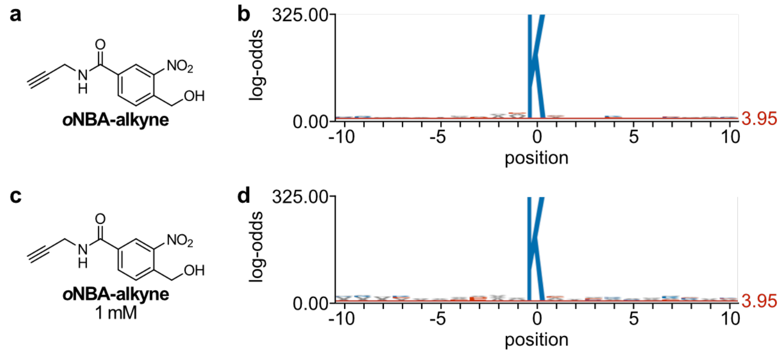

Supplementary Figure 32 | Sequence logos around sites modified by *o*NBA-alkyne. a,**c**, Structure of the probe that was used for treatment of the proteome of *S. aureus* SH1000 at 100 μM (**a**) or 1 mM (**c**) probe concentration. The samples were irradiated for 10 min at 365 nm after 30 min of treatment with the probe. **b**,**d**, Sequence logo for all modified sequences determined using pLogo[17] based on the modified sites identified in the Mass Offset Searches. Position 0 indicates the modified site. The y-axis shows the log-odds of the binomial probability (log-odds) for the shown amino acids. The red line indicates the cut-off of $p < 0.05$. X indicates a residue that is outside of the sequence of the protein (before the *N*-terminus for positions −10 to −1 or after the *C*-terminus for positions 1 to 10) All data is based on technical duplicates.

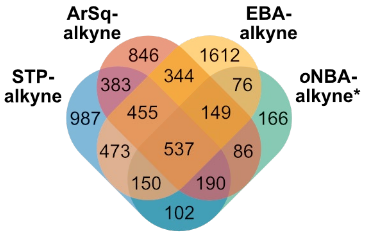

Supplementary Figure 33 | Venn-diagram of the quantified lysines with STP-, ArSq-, EBA- and *o*NBA-alkyne. Data is based on treatment of the proteome of *S. aureus* SH1000 at 100 μM probe concentration. All data is based on technical duplicates. *: The samples were irradiated for 10 min at 365 nm after 30 min of treatment with the probe.

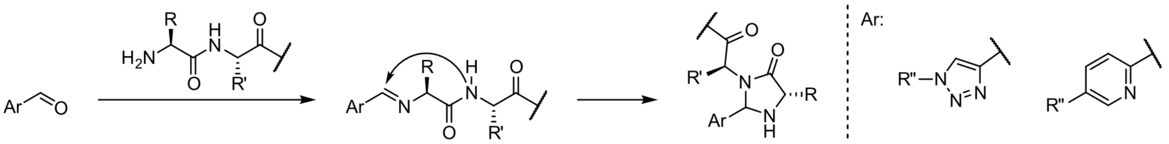

Supplementary Figure 34 | Mechanism of *N*-terminal labelling with heteroaromatic aldehydes (TCA- and PCA-alkyne) as described by Onoda *et al.*[26] and MacDonald *et al.*,[27] respectively.

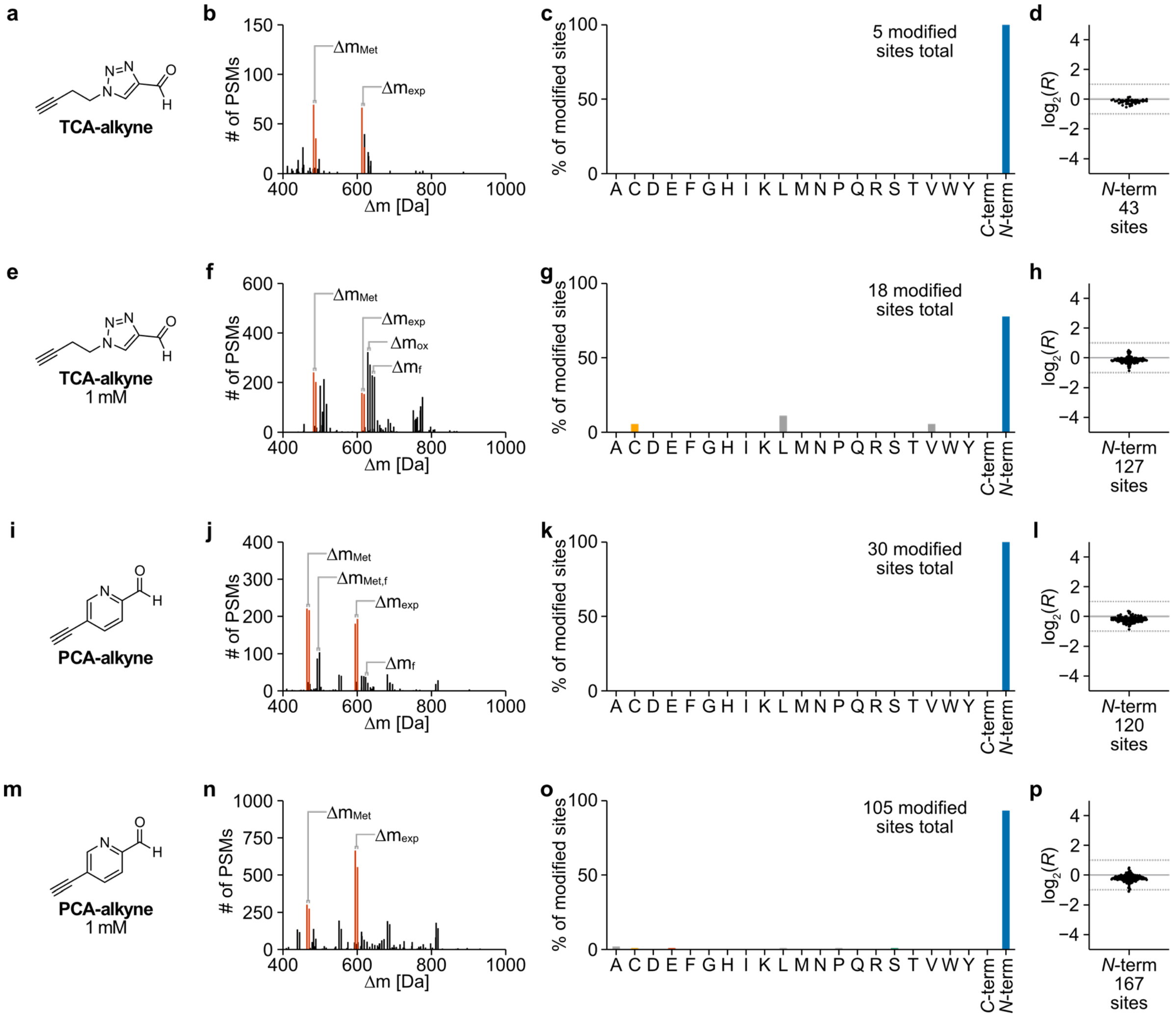

Supplementary Figure 35 | Masses of modification, amino acid selectivity and quantification for TCA- and PCA-alkyne. a,**e**,**i**,**m**, Structures of the probes that were used for treatment of the proteome of *S. aureus* SH1000 at 100 µM (**a**,**i**) or 1 mM (**e**,**m**) probe concentration. **b**,**f**,**j**,**n**, Masses of modification determined through analysis with an Open Search in MSFragger[11, 12]-based FragPipe. The peaks highlighted in red are the masses that have been selected for further analysis within the same row. The expected masses are labelled as Δm_{exp}. Further modification of the modified peptides by oxidation (Δm_{ox}) or formylation (Δm_{f}) is also indicated if the respective peaks were detected. Additionally, the peaks are indicated, in which the initial methionine is cleaved off the peptide and the new *N*-terminus is labelled with the expected modification (Δm_{Met}), as well as those, for which this modification is further formylated ($\Delta m_{Met,f}$). **c**,**g**,**k**,**q**, One peak pair (indicated in red in the same row) is selected for a Mass Offset Search[11] that localizes this modification to the modified amino acid(s). In this way, selectivity is assessed across all proteinogenic amino acids. The bar graph represents the fraction of all modified sites that is modified at the indicated amino acid. *C*-term = *C*-terminal modification. *N*-term = *N*-terminal modification. **d**,**h**,**l**,**r**, Specific amino acid(s) are selected for quantification at the selected masses (indicated in red in the same row) using a Closed Search[11] and the IonQuant[13] feature. The heavy and light samples were mixed at a ratio of 1:1. The grey, solid line indicates the expected values of $\log_2(R) = 0$. The grey, dashed lines indicate the preferred window of quantification ($-1 < \log_2(R) < 1$). All data is based on technical duplicates.

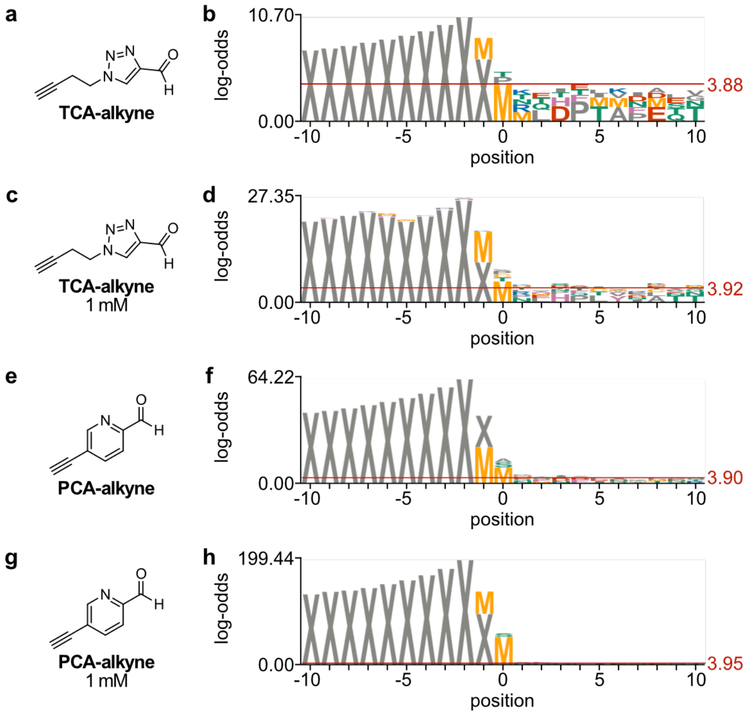

Supplementary Figure 36 | Sequence logos around sites modified by TCA- and PCA-alkyne. a,**c**,**e**,**g**, Structures of the probes that were used for treatment of the proteome of *S. aureus* SH1000 at 100 μM (**a**,**e**) or 1 mM (**c**,**g**) probe concentration. **b**,**d**,**f**,**h**, Sequence logo for all modified sequences determined using pLogo[17] based on the modified sites identified in the Mass Offset Searches. Position 0 indicates the modified site. The y-axis shows the log-odds of the binomial probability (log-odds) for the shown amino acids. The red line indicates the cut-off of $p < 0.05$. X indicates a residue that is outside of the sequence of the protein (before the *N*-terminus for positions −10 to −1 or after the *C*-terminus for positions 1 to 10) All data is based on technical duplicates.

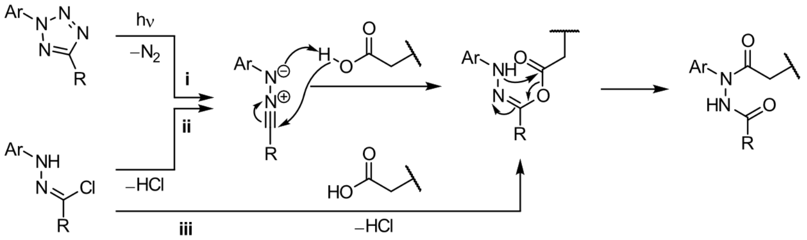

Supplementary Figure 37 | Mechanism of labelling of carboxylic acids with *in situ* generated nitrilimines. These reactive intermediates can be generated from 2,5-disubstituted tetrazoles (**PhTet-**, **AmTet-** and **MeTet-alkyne**) *via* photolysis (**i**) as described by Meier and Heimgartner[28] or from hydrazonoyl chlorides (**HC-alkyne**) *via* elimination of HCl (**ii**) as described by Hegarty *et al.*[29] For hydrazonoyl chlorides, an addition-elimination mechanism (**iii**) is also in principle possible. The stable product is formed from the initial adduct through an *O*,*N*-acyl shift.

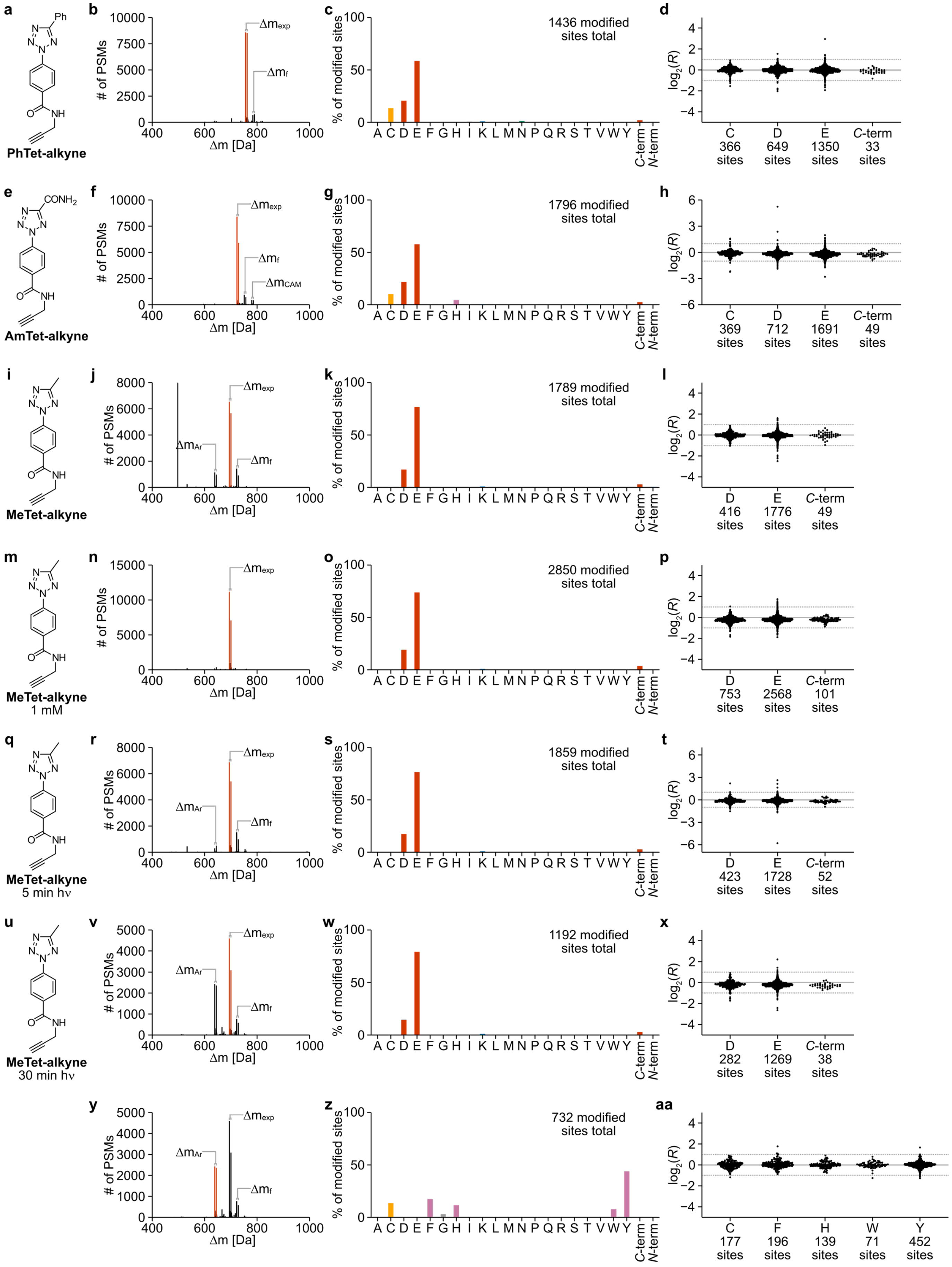

Supplementary Figure 38 | Masses of modification, amino acid selectivity and quantification for probes based on 2,5-disubstituted tetrazoles. a,**e**,**i**,**m**,**q**,**u**, Structures of the probes that were used for treatment of the proteome of *S. aureus* SH1000 at 100 μM (**a**,**e**,**i**,**q**,**u**) or 1 mM (**m**) probe concentration. The samples were irradiated

for 5 min (**q**), 10 min (**a**,**e**,**i**,**m)** or 30 min (**u**) at 280-315 nm after 30 min of treatment with the probe. **b**,**f**,**j**,**n**,**r**,**v**,**y**, Masses of modification determined through analysis with an Open Search in MSFragger[11, 12]-based FragPipe. The peaks highlighted in red are the masses that have been selected for further analysis within the same row. The expected masses are labelled as Δm_{exp}. Further modification of the modified peptides by formylation (Δm_f) or carbamidomethylation on a second cysteine (Δm_{CAM}) is also indicated if the respective peaks were detected. The additionally identified modification by arylation is additionally highlighted as Δm_{Ar}. **c**,**g**,**k**,**o**,**s**,**w**,**z**, One peak pair (indicated in red in the same row) is selected for a Mass Offset Search[11] that localizes this modification to the modified amino acid(s). In this way, selectivity is assessed across all proteinogenic amino acids. The bar graph represents the fraction of all modified sites that is modified at the indicated amino acid. *C*-term = *C*-terminal modification. *N*-term = *N*-terminal modification. **d**,**h**,**l**,**p**,**t**,**x**,**aa**, Specific amino acid(s) are selected for quantification at the selected masses (indicated in red in the same row) using a Closed Search[11] and the IonQuant[13] feature. The heavy and light samples were mixed at a ratio of 1:1. The grey, solid line indicates the expected values of $\log_2(R) = 0$. The grey, dashed lines indicate the preferred window of quantification ($-1 < \log_2(R) < 1$). All data is based on technical duplicates.

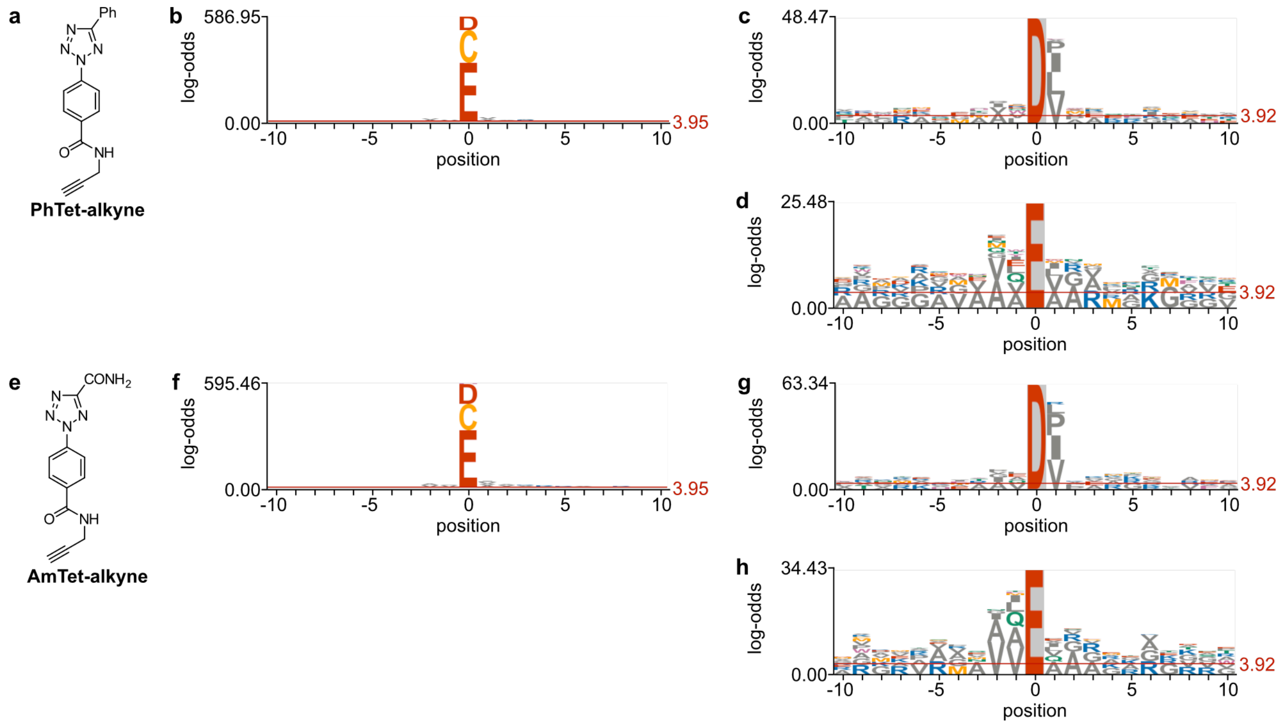

Supplementary Figure 39 | Sequence logos around sites modified by PhTet- and AmTet-alkyne. **a**,**e**, Structures of the probes that were used for treatment of the proteome of *S. aureus* SH1000 at 100 µM probe concentration. The samples were irradiated for 10 min at 280-315 nm after 30 min of treatment with the probe. **b**-**d**,**f**-**h**, Sequence logos determined using pLogo[17] based on the modified sites identified in the Mass Offset Searches. Either the sequence logos for all modified sequences (**b**,**f**) or the sequence logos for a subset of sites that are modified at aspartate (**c**,**g**) or glutamate (**d**,**h**) are shown. Position 0 indicates the modified site. The y-axis shows the log-odds of the binomial probability (log-odds) for the shown amino acids. The red line indicates the cut-off of $p < 0.05$. X indicates a residue that is outside of the sequence of the protein (before the *N*-terminus for positions −10 to −1 or after the *C*-terminus for positions 1 to 10) All data is based on technical duplicates.

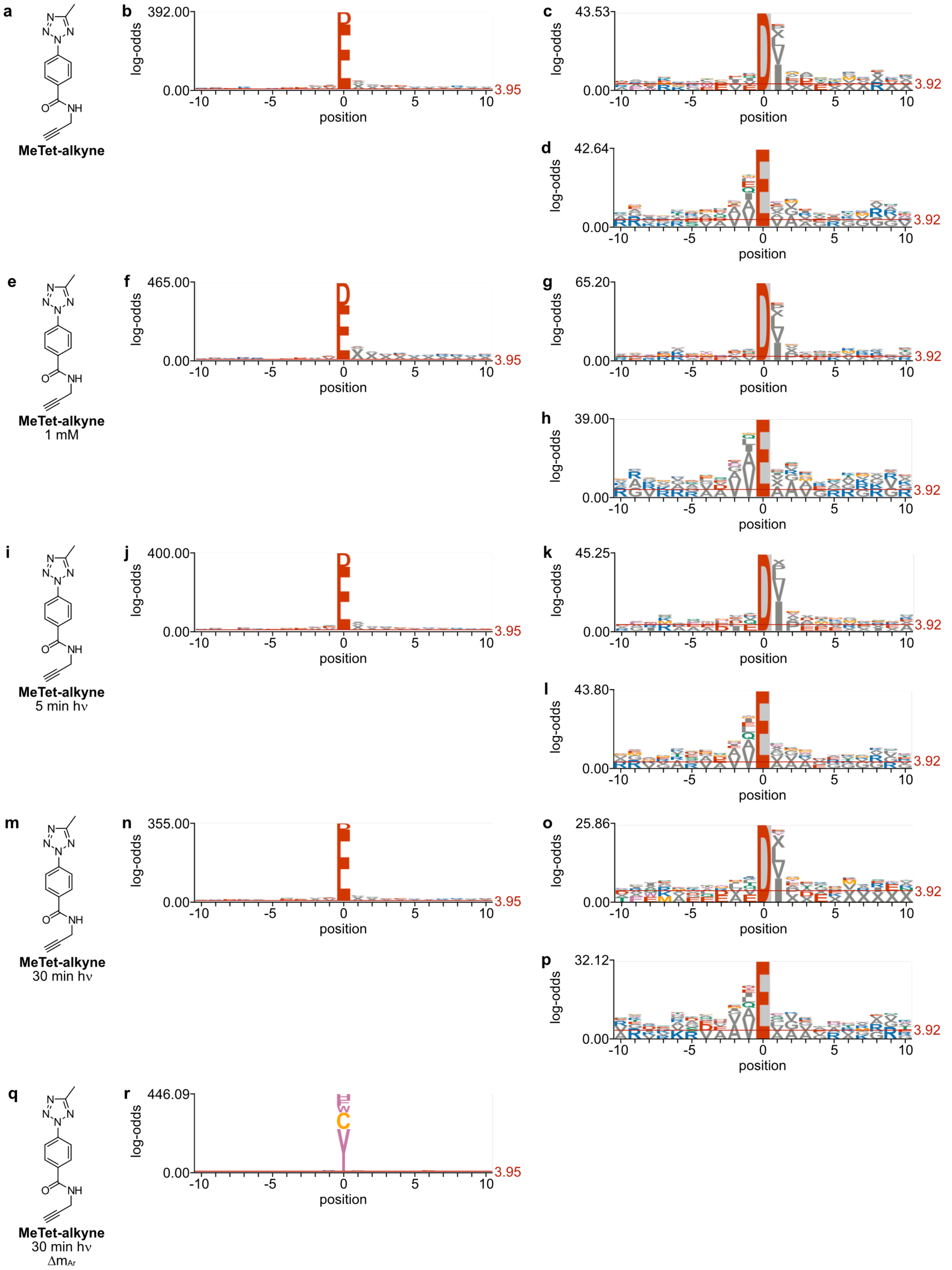

Supplementary Figure 40 | Sequence logos around sites modified by MeTet-alkyne. a,**e**,**i**,**m**,**q**, Structures of the probe that was used for treatment of the proteome of *S. aureus* SH1000 at 100 μM (**a**,**i**,**m**,**q**) or 1 mM (**e**) probe concentration. The samples were irradiated for 5 min (**i**), 10 min (**a**,**e**) or 30 min (**m**,**q**) at 280-315 nm after 30 min of treatment with the probe. For **MeTet-alkyne**, arylation (**q**) was detected as an additional modification. **b-d**,**f-h**,**j-l**,**n-p**,**r** Sequence logos determined using pLogo[17] based on the modified sites identified in the Mass Offset

Searches. Either the sequence logos for all modified sequences (**b**,**f**,**j**,**n**,**r**) or the sequence logos for a subset of sites that are modified at aspartate (**c**,**g**,**k**,**o**) or glutamate (**d**,**h**,**l**,**p**) are shown. Position 0 indicates the modified site. The y-axis shows the log-odds of the binomial probability (log-odds) for the shown amino acids. The red line indicates the cut-off of $p < 0.05$. X indicates a residue that is outside of the sequence of the protein (before the *N*-terminus for positions −10 to −1 or after the *C*-terminus for positions 1 to 10) All data is based on technical duplicates.

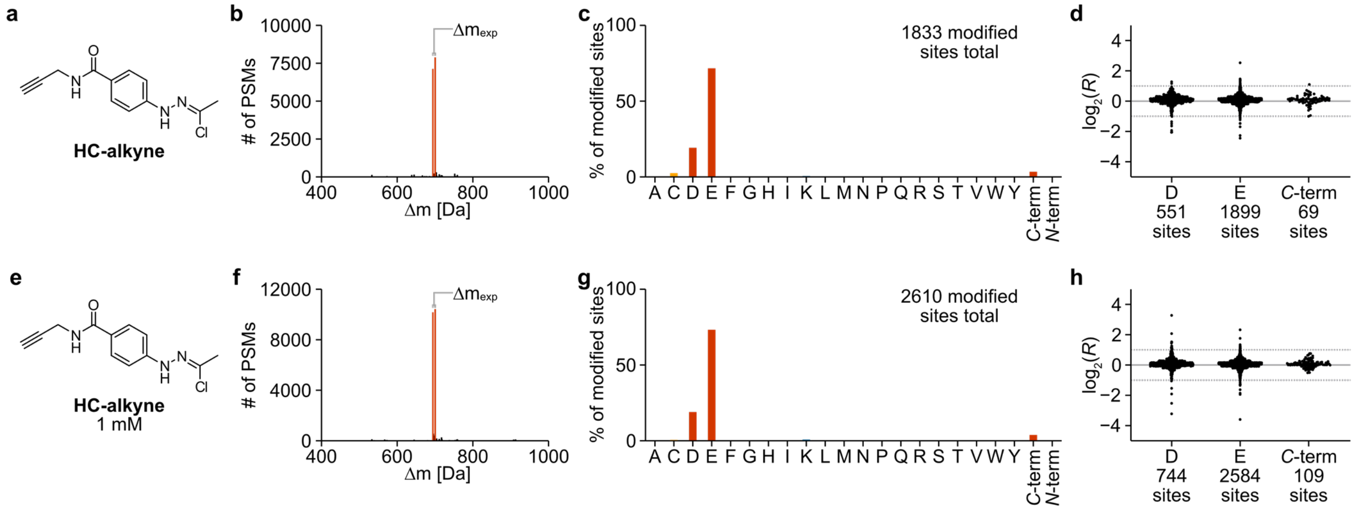

Supplementary Figure 41 | Masses of modification, amino acid selectivity and quantification for HC-alkyne. **a**,**e**, Structure of the probe that was used for treatment of the proteome of *S. aureus* SH1000 at 100 μM (**a**) or 1 mM (**e**) probe concentration. **b**,**f**,**j**,**n**,**p**, Masses of modification determined through analysis with an Open Search in MSFragger[11, 12]-based FragPipe. The peaks highlighted in red are the masses that have been selected for further analysis within the same row. The expected masses are labelled as Δm_{exp}. **c**,**g**, One peak pair (indicated in red in the same row) is selected for a Mass Offset Search[11] that localizes this modification to the modified amino acid(s). In this way, selectivity is assessed across all proteinogenic amino acids. The bar graph represents the fraction of all modified sites that is modified at the indicated amino acid. *C*-term = *C*-terminal modification. *N*-term = *N*-terminal modification. **d**,**h**, Specific amino acid(s) are selected for quantification at the selected masses (indicated in red in the same row) using a Closed Search[11] and the IonQuant[13] feature. The heavy and light samples were mixed at a ratio of 1:1. The grey, solid line indicates the expected values of $\log_2(R) = 0$. The grey, dashed lines indicate the preferred window of quantification ($-1 < \log_2(R) < 1$). All data is based on technical duplicates.

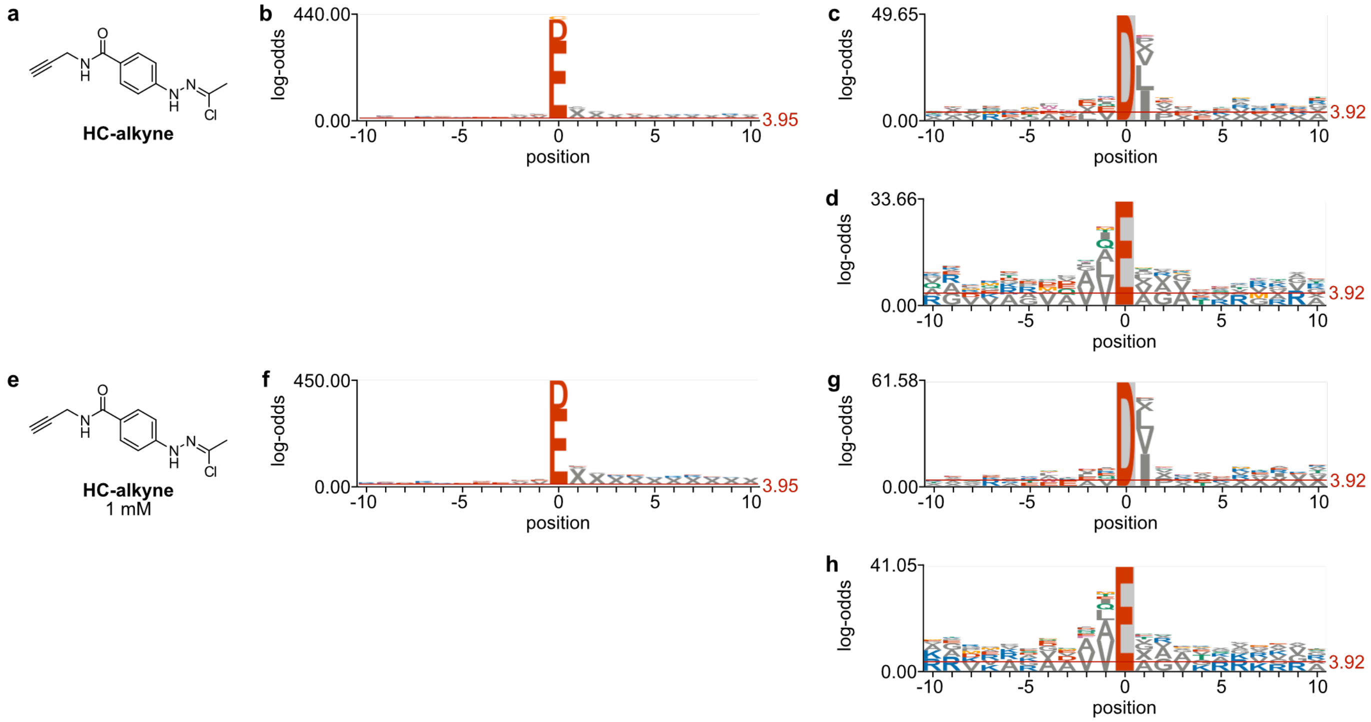

Supplementary Figure 42 | Sequence logos around sites modified by HC-alkyne. a,**e**, Structure of the probe that was used for treatment of the proteome of *S. aureus* SH1000 at 100 μM (**a**) or 1 mM (**e**) probe concentration. **b**-**d**,**f**-**h**, Sequence logos determined using pLogo[17] based on the modified sites identified in the Mass Offset Searches. Either the sequence logos for all modified sequences (**b**,**f**) or the sequence logos for a subset of sites that are modified at aspartate (**c**,**d**) or glutamate (**g**,**h**) are shown. Position 0 indicates the modified site. The y-axis shows the log-odds of the binomial probability (log-odds) for the shown amino acids. The red line indicates the cut-off of $p < 0.05$. X indicates a residue that is outside of the sequence of the protein (before the *N*-terminus for positions −10 to −1 or after the *C*-terminus for positions 1 to 10) All data is based on technical duplicates.

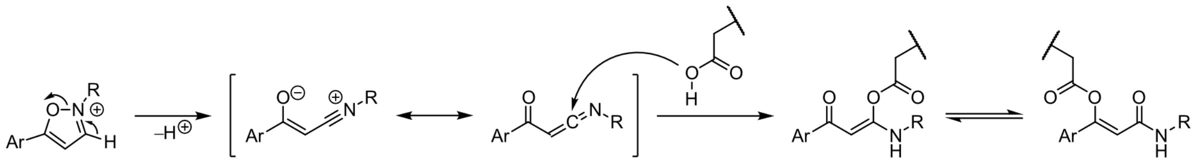

Supplementary Figure 43 | Mechanism of labelling of aspartate and glutamate with isoxazolium salt Isx-alkyne as described by Woodward and Olofson.[30]

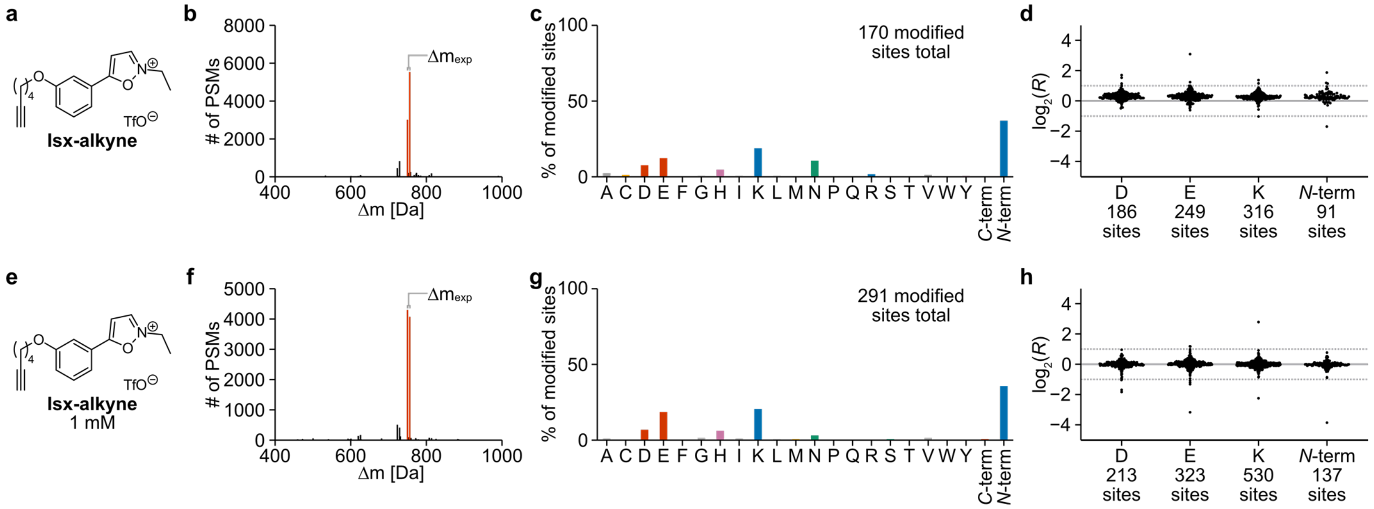

Supplementary Figure 44 | Masses of modification, amino acid selectivity and quantification for Isx-alkyne. **a**,**e**, Structure of the probe that was used for treatment of the proteome of *S. aureus* SH1000 at 100 μM (**a**) or 1 mM (**e**) probe concentration. **b**,**f**, Masses of modification determined through analysis with an Open Search in MSFragger[11, 12]-based FragPipe. The peaks highlighted in red are the masses that have been selected for further analysis within the same row. The expected masses are labelled as Δm_{exp}. **c**,**g**, One peak pair (indicated in red in the same row) is selected for a Mass Offset Search[11] that localizes this modification to the modified amino acid(s). In this way, selectivity is assessed across all proteinogenic amino acids. The bar graph represents the fraction of all modified sites that is modified at the indicated amino acid. *C*-term = *C*-terminal modification. *N*-term = *N*-terminal modification. **d**,**h**, Specific amino acid(s) are selected for quantification at the selected masses (indicated in red in the same row) using a Closed Search[11] and the IonQuant[13] feature. The heavy and light samples were mixed at a ratio of 1:1. The grey, solid line indicates the expected values of $\log_2(R) = 0$. The grey, dashed lines indicate the preferred window of quantification ($-1 < \log_2(R) < 1$). All data is based on technical duplicates.

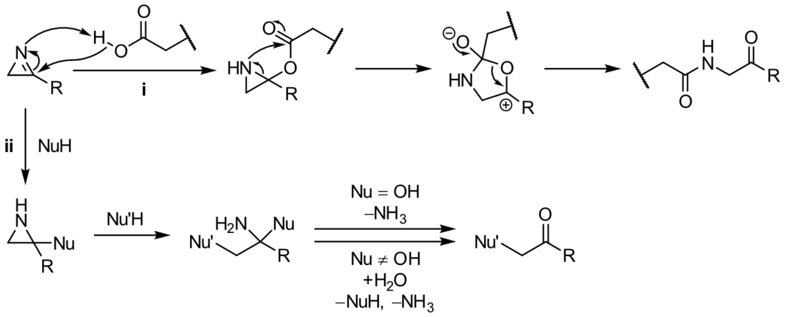

Supplementary Figure 45 | Mechanism of labelling of aspartate and glutamate with 2*H*-azirines (Az-alkyne) as described by Black and Doyle (i).[31] Furthermore, a speculative reaction mechanism with nucleophiles leading to a modification that is 1 Da heavier than the expected adduct is shown (ii).

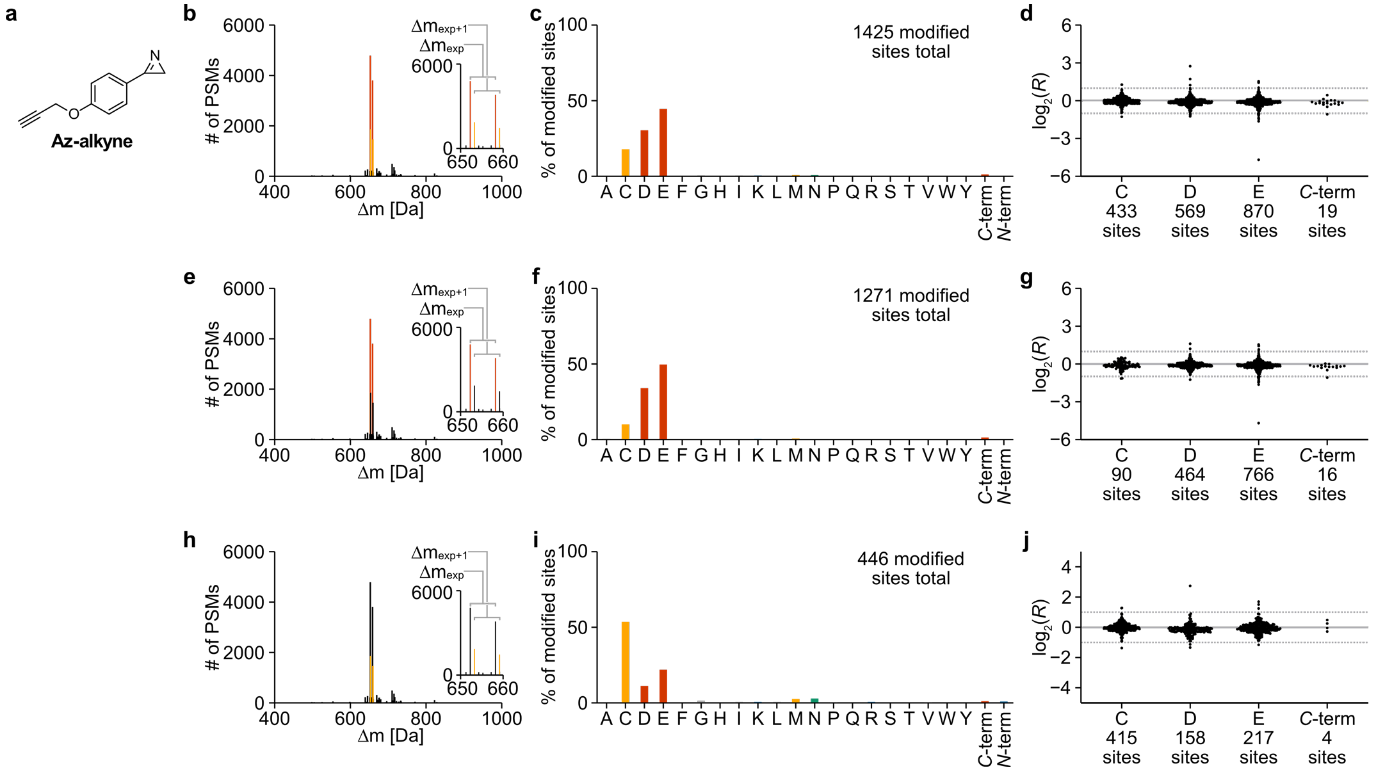

Supplementary Figure 46 | Masses of modification, amino acid selectivity and quantification for Az-alkyne. **a**, Structure of the probe that was used for treatment of the proteome of *S. aureus* SH1000 at 100 µM probe concentration. **b**,**e**,**h**, Masses of modification determined through analysis with an Open Search in MSFragger[11, 12]-based FragPipe. The peaks highlighted in red or yellow are the masses that have been selected for further analysis within the same row. The expected masses are labelled as Δm_{exp}. The expected mass +1 Da is labelled as Δm_{exp+1}. **c**,**f**,**i**, One peak pair (indicated in red or yellow in the same row, **e**,**h**) or both peak pairs (indicated in red and yellow in the same row, **b**) are selected for a Mass Offset Search[11] that localizes these modification(s) to the modified amino acid(s). In this way, selectivity is assessed across all proteinogenic amino acids. The bar graph represents the fraction of all modified sites that is modified at the indicated amino acid. *C*-term = *C*-terminal modification. *N*-term = *N*-terminal modification. **d**,**g**,**j**, Specific amino acid(s) are selected for quantification at the selected masses (indicated in red or yellow in the same row, **e**,**h**, or indicated in red and yellow in the same row, **b**) using a Closed Search[11] and the IonQuant[13] feature. The heavy and light samples were mixed at a ratio of 1:1. The grey, solid line indicates the expected values of $\log_2(R) = 0$. The grey, dashed lines indicate the preferred window of quantification ($-1 < \log_2(R) < 1$). All data is based on technical duplicates.

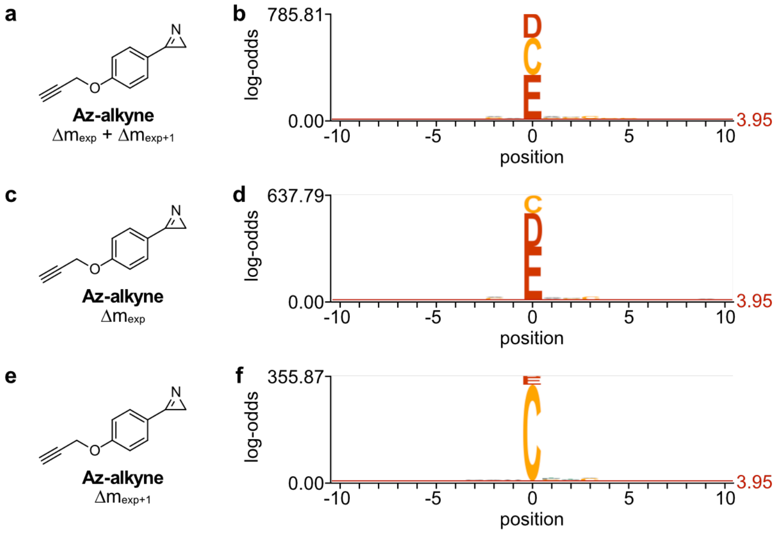

Supplementary Figure 47 | Sequence logos around sites modified by Az-alkyne. a,**c**,**e**, Structure of the probe that was used for treatment of the proteome of *S. aureus* SH1000 at 100 µM probe concentration. As computationally differentiating the modifications Δm_{exp} and Δm_{exp+1} was not fully possible, they were analysed together (**a**) or in separate (**c**,**e**) runs. **b**,**d**,**f**, Sequence logo for all modified sequences determined using pLogo[17] based on the modified sites identified in the Mass Offset Searches. Position 0 indicates the modified site. The y-axis shows the log-odds of the binomial probability (log-odds) for the shown amino acids. The red line indicates the cut-off of $p < 0.05$. X indicates a residue that is outside of the sequence of the protein (before the *N*-terminus for positions −10 to −1 or after the *C*-terminus for positions 1 to 10) All data is based on technical duplicates.

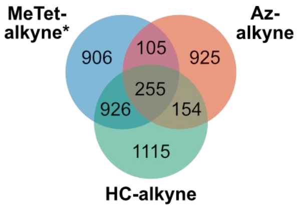

Supplementary Figure 48 | Venn-diagram of the quantified aspartates and glutamates with MeTet-, Az- and HC-alkyne. Data is based on treatment of the proteome of *S. aureus* SH1000 at 100 µM probe concentration. All data is based on technical duplicates. *: The samples were irradiated for 10 min at 280-315 nm after 30 min of treatment with the probe.

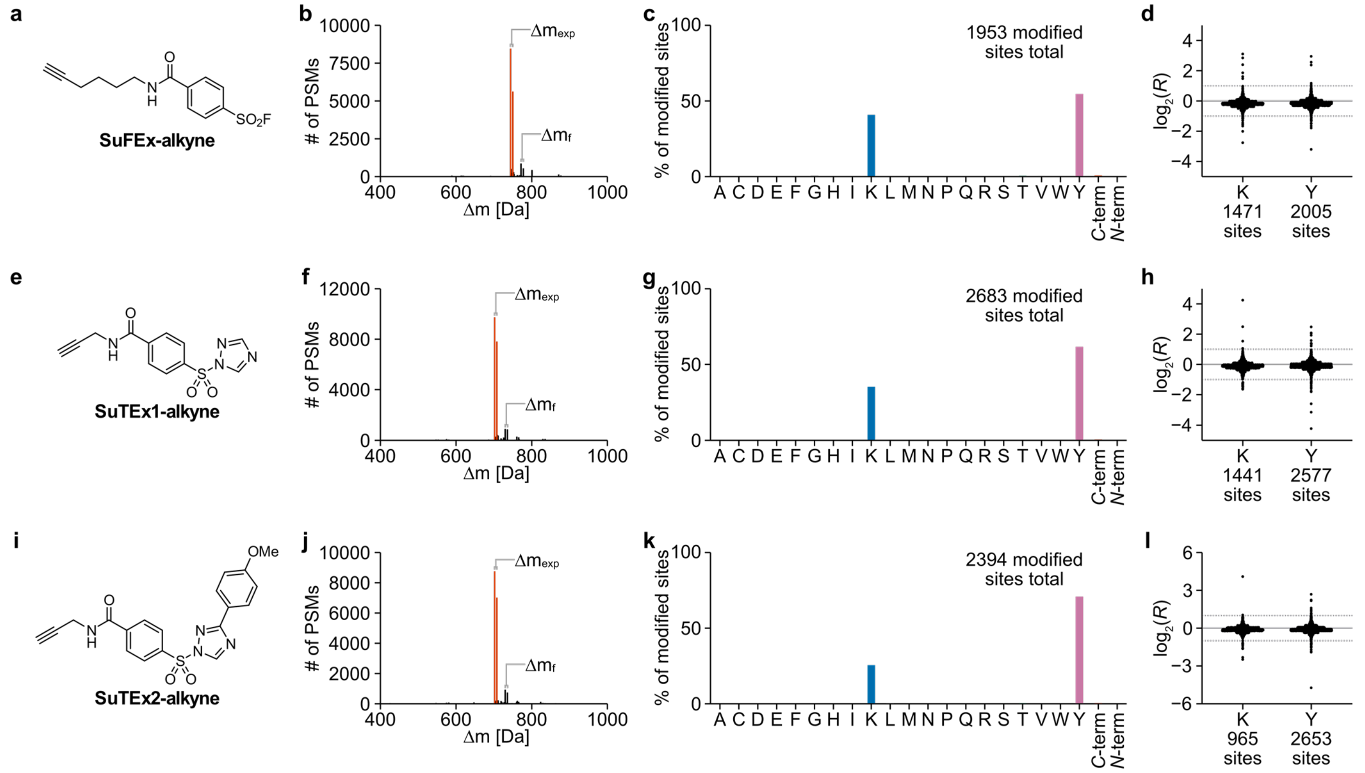

Supplementary Figure 49 | Masses of modification, amino acid selectivity and quantification for SuFEx-, SuTEx1- and SuTEx2-alkyne. **a**,**e**,**i**, Structures of the probes that were used for treatment of the proteome of *S. aureus* SH1000 at 100 μM probe concentration. **b**,**f**,**j**, Masses of modification determined through analysis with an Open Search in MSFragger[11, 12]-based FragPipe. The peaks highlighted in red are the masses that have been selected for further analysis within the same row. The expected masses are labelled as Δm_{exp}. Further modification of the modified peptides by formylation (Δm_f) is also indicated. **c**,**g**,**k**, One peak pair (indicated in red in the same row) is selected for a Mass Offset Search[11] that localizes this modification to the modified amino acid(s). In this way, selectivity is assessed across all proteinogenic amino acids. The bar graph represents the fraction of all modified sites that is modified at the indicated amino acid. *C*-term = *C*-terminal modification. *N*-term = *N*-terminal modification. **d**,**h**,**l**, Specific amino acid(s) are selected for quantification at the selected masses (indicated in red in the same row) using a Closed Search[11] and the IonQuant[13] feature. The heavy and light samples were mixed at a ratio of 1:1. The grey, solid line indicates the expected values of $\log_2(R) = 0$. The grey, dashed lines indicate the preferred window of quantification ($-1 < \log_2(R) < 1$). All data is based on technical duplicates.

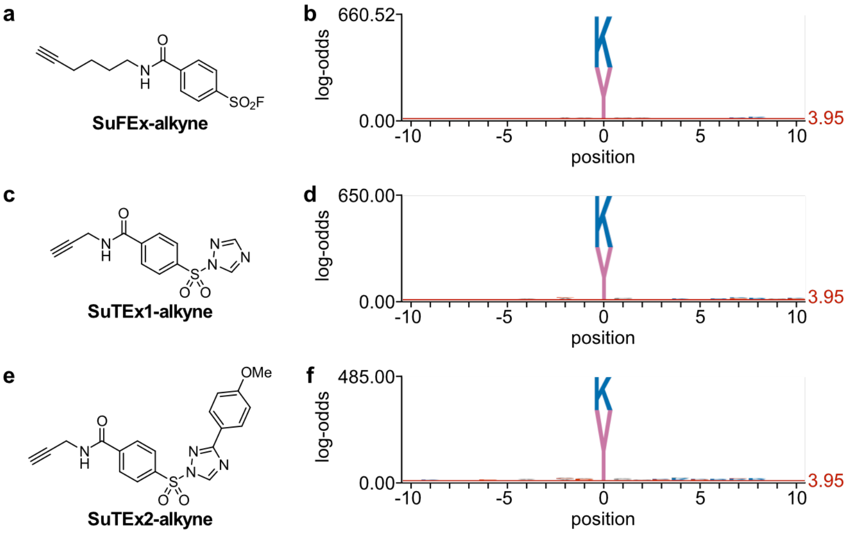

Supplementary Figure 50 | Sequence logos around sites modified by SuFEx-, SuTEx1- and SuTEx2-alkyne. **a**,**c**,**e**, Structures of the probes that were used for treatment of the proteome of *S. aureus* SH1000 at 100 μM probe concentration. **b**,**d**,**f**, Sequence logos for all modified sequences determined using pLogo[17] based on the modified sites identified in the Mass Offset Searches. Position 0 indicates the modified site. The y-axis shows the log-odds of the binomial probability (log-odds) for the shown amino acids. The red line indicates the cut-off of $p < 0.05$. X indicates a residue that is outside of the sequence of the protein (before the *N*-terminus for positions −10 to −1 or after the *C*-terminus for positions 1 to 10) All data is based on technical duplicates.

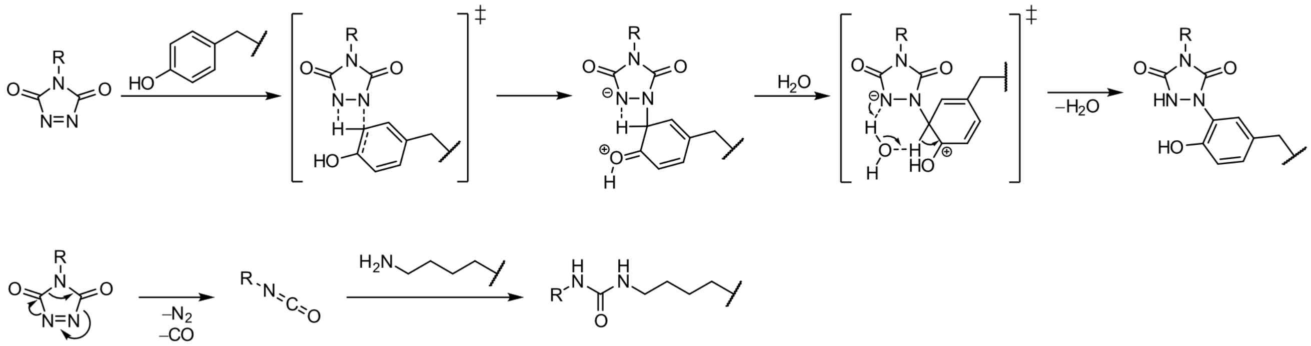

Supplementary Figure 51 | Mechanism of tyrosine-labelling with 4-substituted 3*H*-1,2,4-triazole-3,5(4*H*)-diones (PTAD-alkyne) through electrophilic aromatic substitution as described by Kaiser *et al.*[32] (top). Furthermore, formation of isocyanates from 4-substituted 3*H*-1,2,4-triazole-3,5(4*H*)-diones (**PTAD-alkyne**) and subsequent labelling of lysine and the protein *N*-terminus as described by Ban *et al.* is shown (bottom).[33]

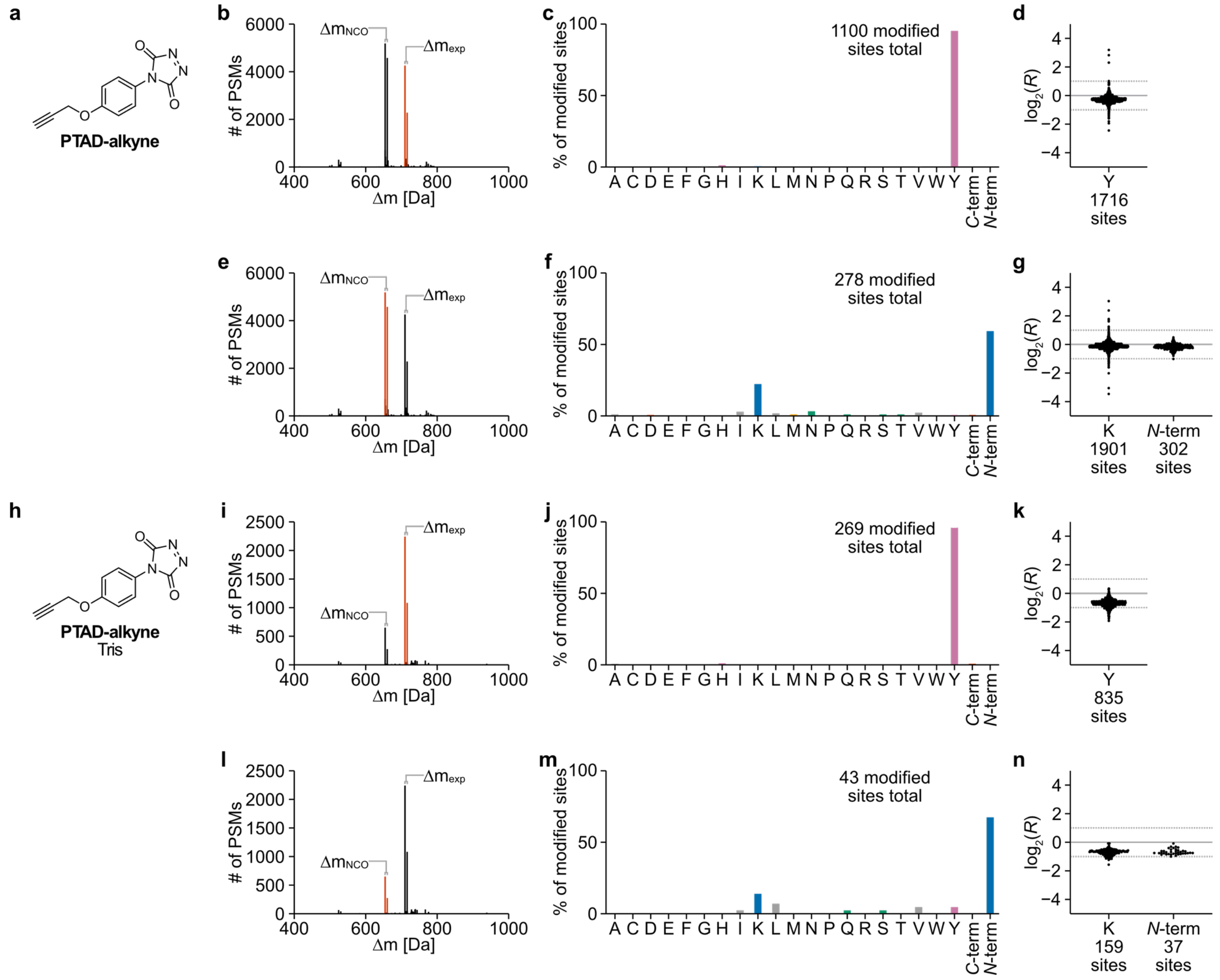

Supplementary Figure 52 | Masses of modification, amino acid selectivity and quantification for PTAD-alkyne. a,**h**, Structure of the probe that was used for treatment of the proteome of *S. aureus* SH1000 at 100 µM probe concentration in the absence (**a**) or presence (**h**) of Tris. **b**,**e**,**i**,**l**, Masses of modification determined through analysis with an Open Search in MSFragger[11, 12]-based FragPipe. The peaks highlighted in red are the masses that have been selected for further analysis within the same row. The expected masses are labelled as Δm_{exp}. The mass that corresponds to reactivity through formation of the isocyanate is labelled as Δm_{NCO}. **c**,**f**,**j**,**m**, One peak pair (indicated in red in the same row) is selected for a Mass Offset Search[11] that localizes this modification to the modified amino acid(s). In this way, selectivity is assessed across all proteinogenic amino acids. The bar graph represents the fraction of all modified sites that is modified at the indicated amino acid. *C*-term = *C*-terminal modification. *N*-term = *N*-terminal modification. **d**,**g**,**k**,**n**, Specific amino acid(s) are selected for quantification at the selected masses (indicated in red in the same row) using a Closed Search[11] and the IonQuant[13] feature. The heavy and light samples were mixed at a ratio of 1:1. The grey, solid line indicates the expected values of $\log_2(R) = 0$. The grey, dashed lines indicate the preferred window of quantification ($-1 < \log_2(R) < 1$). All data is based on technical duplicates.

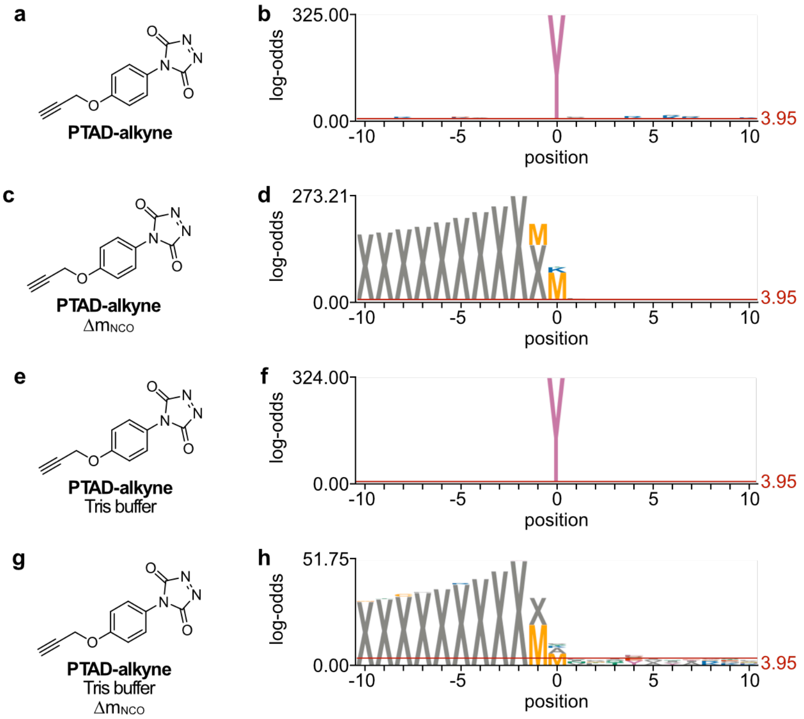

Supplementary Figure 53 | Sequence logos around sites modified by PTAD-alkyne. a,**c**,**e**,**g**, Structure of the probe that was used for treatment of the proteome of *S. aureus* SH1000 at 100 μM probe concentration in the absence (**a**,**c**) or presence (**e**,**g**) of Tris. Reaction with the isocyanate (**c**,**g**) resulting from fragmentation of the probe was detected as an additional modification. **b**,**d**,**f**,**h**, Sequence logo for all modified sequences determined using pLogo[17] based on the modified sites identified in the Mass Offset Searches. Position 0 indicates the modified site. The y-axis shows the log-odds of the binomial probability (log-odds) for the shown amino acids. The red line indicates the cut-off of $p < 0.05$. X indicates a residue that is outside of the sequence of the protein (before the *N*-terminus for positions −10 to −1 or after the *C*-terminus for positions 1 to 10) All data is based on technical duplicates.

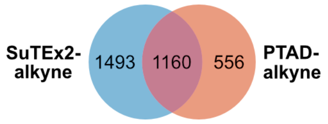

Supplementary Figure 54 | Venn-diagram of the quantified tyrosines with SuTEx2- and PTAD-alkyne. Data is based on treatment of the proteome of *S. aureus* SH1000 at 100 μM probe concentration. All data is based on technical duplicates.

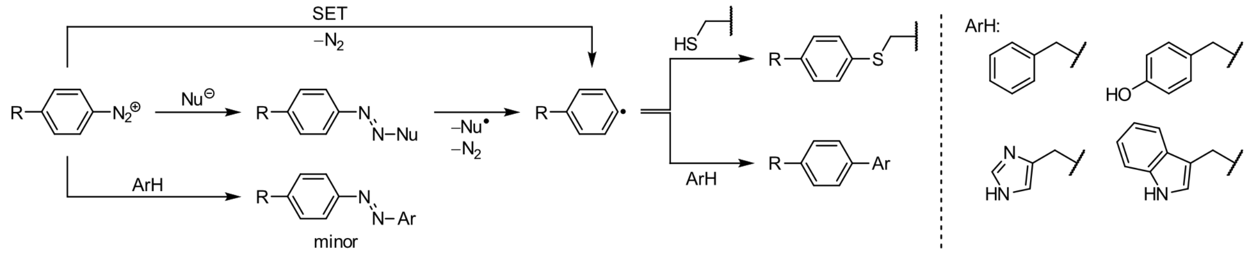

Supplementary Figure 55 | Plausible radical arylation of cysteine and aromatic amino acids after *in situ* generation of aryl radicals from diazonium ions (DA1-, DA2- and DA3-alkyne) as described by Naveen *et al.* for cysteine[5] and by Fehler *et al.* for tyrosine and tryptophan.[6] In the complex setting of cellular lysates, aryl radicals could be formed through either an addition-fragmentation mechanism or a single electron transfer (SET) and elimination of N_2.[34] Among others, NADH, ascorbate and glutathione have been described as reductants of diazonium ions under physiological conditions.[3, 4]

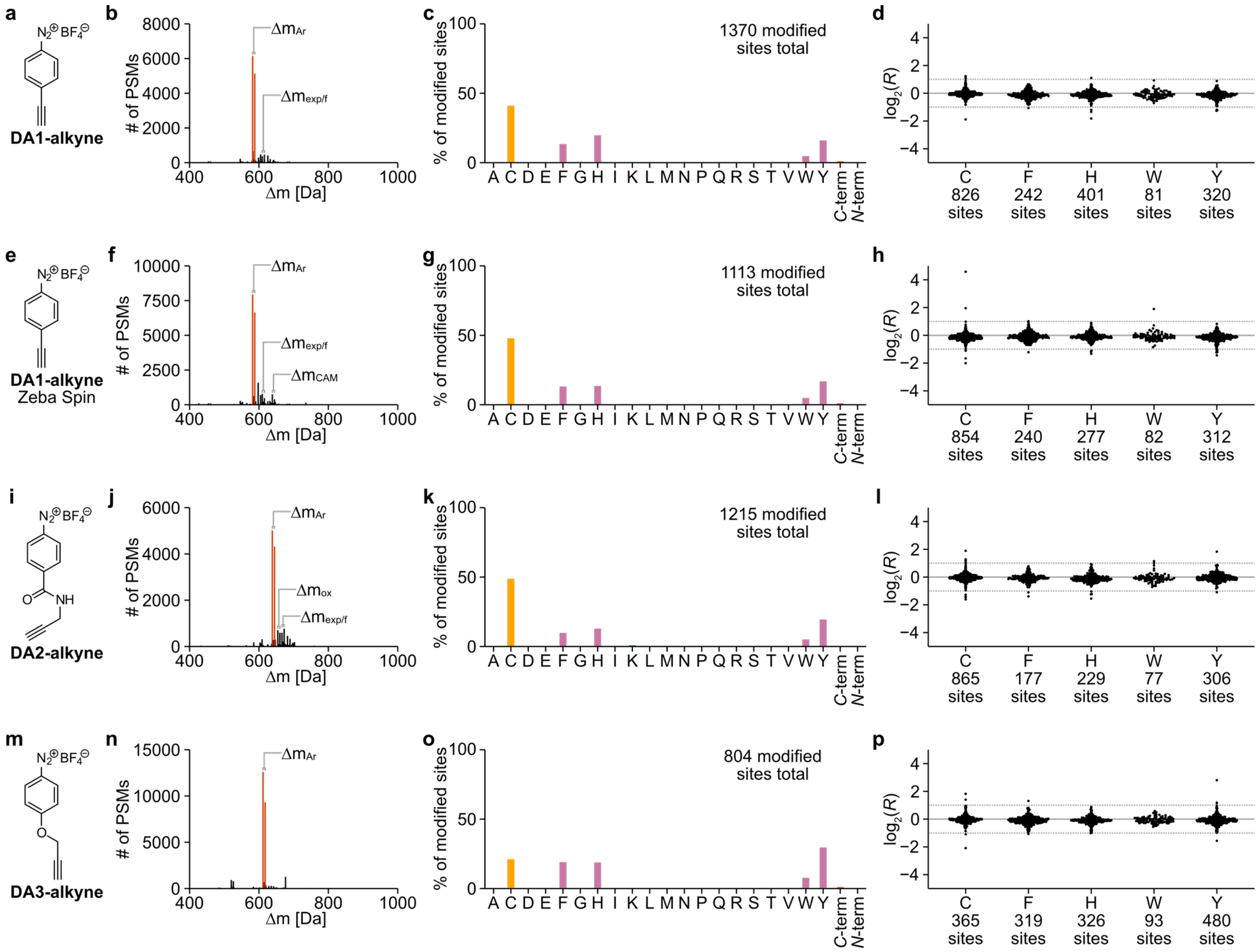

Supplementary Figure 56 | Masses of modification, amino acid selectivity and quantification for diazonium salt-containing probes. a,**e**,**i**,**m**, Structures of the probes that were used for treatment of the proteome of *S. aureus* SH1000 at 100 µM probe concentration. For **DA1-alkyne**, Zeba Spin purification (**e**) was conducted before CuAAC to isoDTB tags as a control experiment. **b**,**f**,**j**,**n**, Masses of modification determined through analysis with an Open Search in MSFragger[11, 12]-based FragPipe. The peaks highlighted in red are the masses that have been selected for further analysis within the same row. The expected masses for azo coupling are labelled as $\Delta m_{exp/f}$ and could also correspond to formylation of the arylation product. The peaks corresponding to arylation are labelled as Δm_{Ar}. Further modification of the arylated peptides by oxidation (Δm_{ox}) or carbamidomethylation on a second cysteine (Δm_{CAM}) is also indicated if the respective peaks were detected. **c**,**g**,**k**,**o**, One peak pair (indicated in red in the same row) is selected for a Mass Offset Search[11] that localizes this modification to the modified amino acid(s). In this way,

selectivity is assessed across all proteinogenic amino acids. The bar graph represents the fraction of all modified sites that is modified at the indicated amino acid. *C*-term = *C*-terminal modification. *N*-term = *N*-terminal modification. **d**,**h**,**l**,**p**, Specific amino acid(s) are selected for quantification at the selected masses (indicated in red in the same row) using a Closed Search[11] and the IonQuant[13] feature. The heavy and light samples were mixed at a ratio of 1:1. The grey, solid line indicates the expected values of log(*R*) = 0. The grey, dashed lines indicate the preferred window of quantification ($-1 < \log_2(R) < 1$). All data is based on technical duplicates.

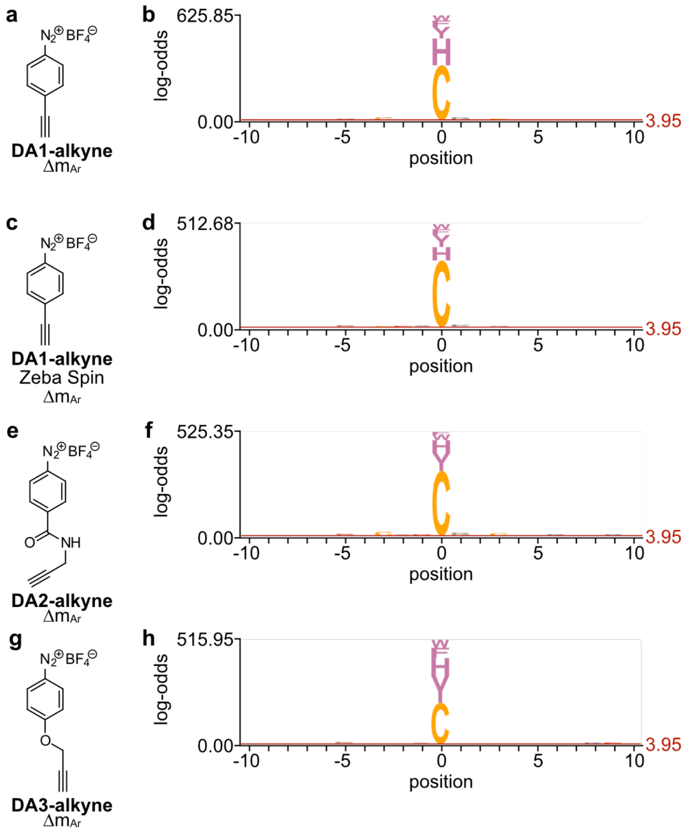

Supplementary Figure 57 | Sequence logos around sites modified by DA1-, DA2- and DA3-alkyne. **a**,**c**,**e**,**g**, Structures of the probes that were used for treatment of the proteome of *S. aureus* SH1000 at 100 μM probe concentration. For **DA1-alkyne**, Zeba Spin purification (**e**) was conducted before CuAAC to isoDTB tags as a control experiment. **b**,**d**,**f**,**h**, Sequence logo for all modified sequences determined using pLogo[17] based on the modified sites identified in the Mass Offset Searches. Position 0 indicates the modified site. The y-axis shows the log-odds of the binomial probability (log-odds) for the shown amino acids. The red line indicates the cut-off of $p < 0.05$. X indicates a residue that is outside of the sequence of the protein (before the *N*-terminus for positions −10 to −1 or after the *C*-terminus for positions 1 to 10) All data is based on technical duplicates.

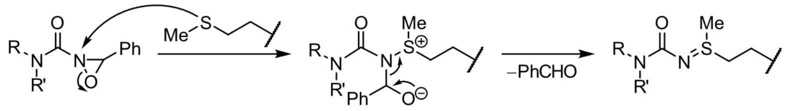

Supplementary Figure 58 | Mechanism of labelling of methionine with oxaziridines (OxMet1- and OxMet2-alkyne) as described by Lin *et al.*[35]

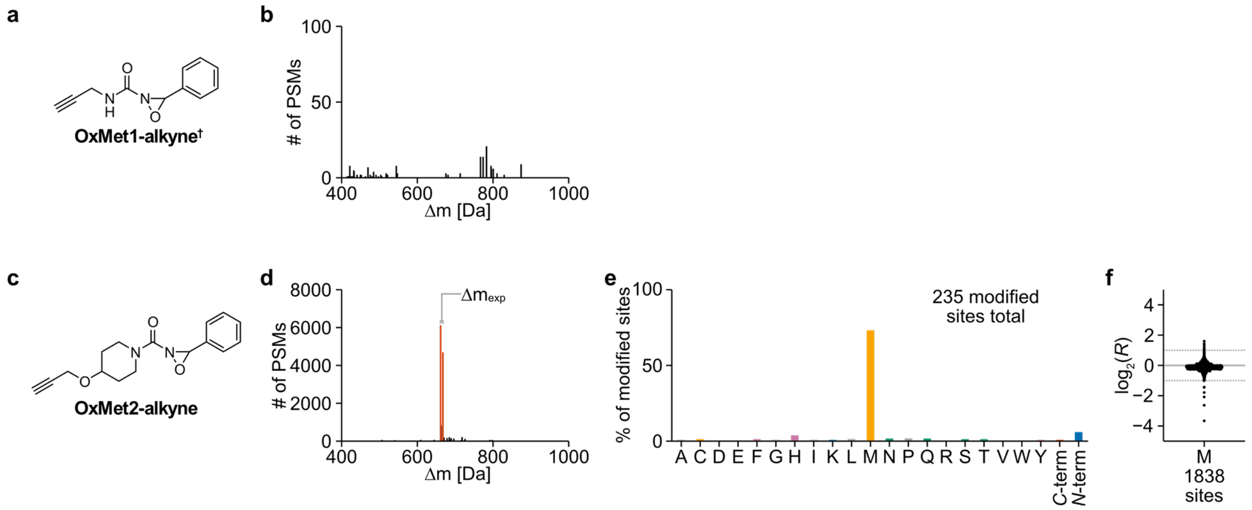

Supplementary Figure 59 | Masses of modification, amino acid selectivity and quantification for oxaziridine-based probes. a,**c**, Structures of the probes that were used for treatment of the proteome of *S. aureus* SH1000 at 100 μM probe concentration. **b**,**d**, Masses of modification determined through analysis with an Open Search in MSFragger[11, 12]-based FragPipe. The peaks highlighted in red are the masses that have been selected for further analysis within the same row. The expected masses are labelled as Δm_{exp}. **e**, One peak pair (indicated in red in the same row) is selected for a Mass Offset Search[11] that localizes this modification to the modified amino acid(s). In this way, selectivity is assessed across all proteinogenic amino acids. The bar graph represents the fraction of all modified sites that is modified at the indicated amino acid. *C*-term = *C*-terminal modification. *N*-term = *N*-terminal modification. **f**, Specific amino acid(s) are selected for quantification at the selected masses (indicated in red in the same row) using a Closed Search[11] and the IonQuant[13] feature. The heavy and light samples were mixed at a ratio of 1:1. The grey, solid line indicates the expected values of $\log_2(R) = 0$. The grey, dashed lines indicate the preferred window of quantification ($-1 < \log_2(R) < 1$). All data is based on technical duplicates. †: No clear mass of modification was detected and therefore no analysis of the amino acid selectivity was possible.

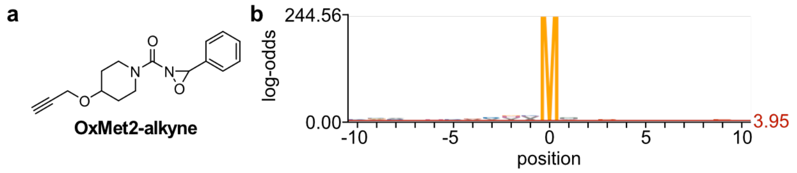

Supplementary Figure 60 | Sequence logos around sites modified by OxMet2-alkyne. a, Structure of the probe that was used for treatment of the proteome of *S. aureus* SH1000 at 100 μM probe concentration. **b**, Sequence logo for all modified sequences determined using pLogo[17] based on the modified sites identified in the Mass Offset Searches. Position 0 indicates the modified site. The y-axis shows the log-odds of the binomial probability (log-odds) for the shown amino acids. The red line indicates the cut-off of $p < 0.05$. X indicates a residue that is outside of the sequence of the protein (before the *N*-terminus for positions −10 to −1 or after the *C*-terminus for positions 1 to 10) All data is based on technical duplicates.

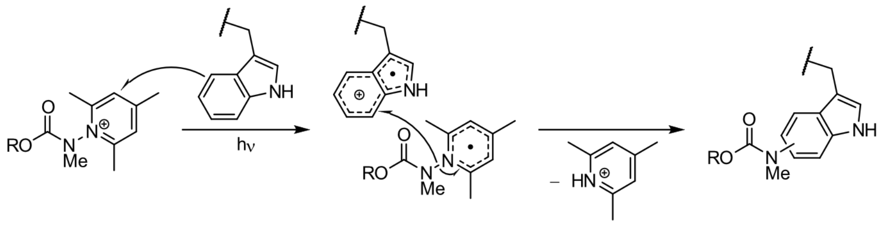

Supplementary Figure 61 | Mechanism of tryptophan-labelling with *N*-carbamoylpyridinium salts (CP-alkyne) through photoinduced electron transfer (PET) as described by Tower *et al.*[36]

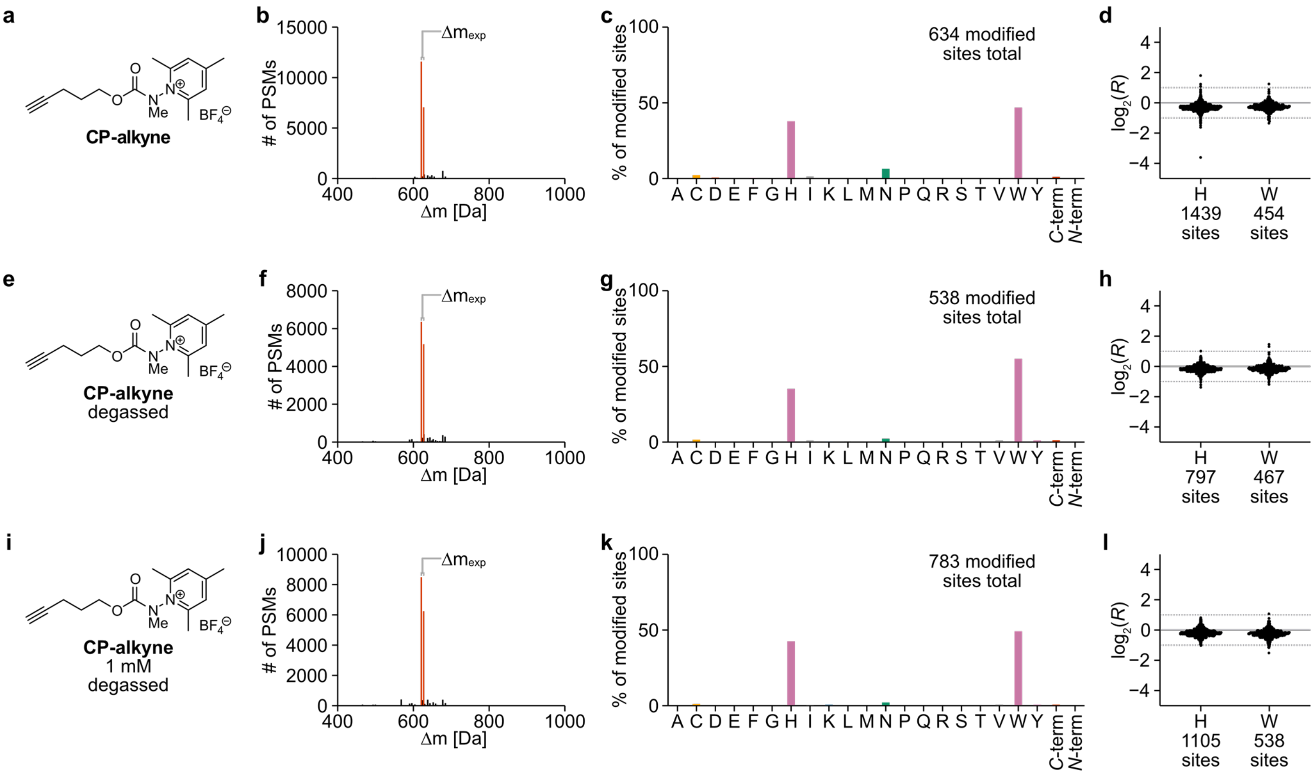

Supplementary Figure 62 | Masses of modification, amino acid selectivity and quantification for CP-alkyne. **a**,**e**,**i**, Structure of the probe that was used for treatment of the proteome of *S. aureus* SH1000 at 100 μM (**a**,**e**) or 1 mM (**i**) probe concentration either open to air (**a**) or in a degassed solution under argon (**e**,**i**). The samples were irradiated for 10 min at 280-315 nm after 30 min of treatment with the probe. During the 30 min pre-treatment the samples were degassed by passing argon through the solution if indicated. **b**,**f**,**j**, Masses of modification determined through analysis with an Open Search in MSFragger[11, 12]-based FragPipe. The peaks highlighted in red are the masses that have been selected for further analysis within the same row. The expected masses are labelled as Δm_{exp}. **c**,**g**,**k**, One peak pair (indicated in red in the same row) is selected for a Mass Offset Search[11] that localizes this modification to the modified amino acid(s). In this way, selectivity is assessed across all proteinogenic amino acids. The bar graph represents the fraction of all modified sites that is modified at the indicated amino acid. *C*-term = *C*-terminal modification. *N*-term = *N*-terminal modification. **d**,**h**,**l**, Specific amino acid(s) are selected for quantification at the selected masses (indicated in red in the same row) using a Closed Search[11] and the IonQuant[13] feature. The heavy and light samples were mixed at a ratio of 1:1. The grey, solid line indicates the expected values of $\log_2(R) = 0$. The grey, dashed lines indicate the preferred window of quantification ($-1 < \log_2(R) < 1$). All data is based on technical duplicates.

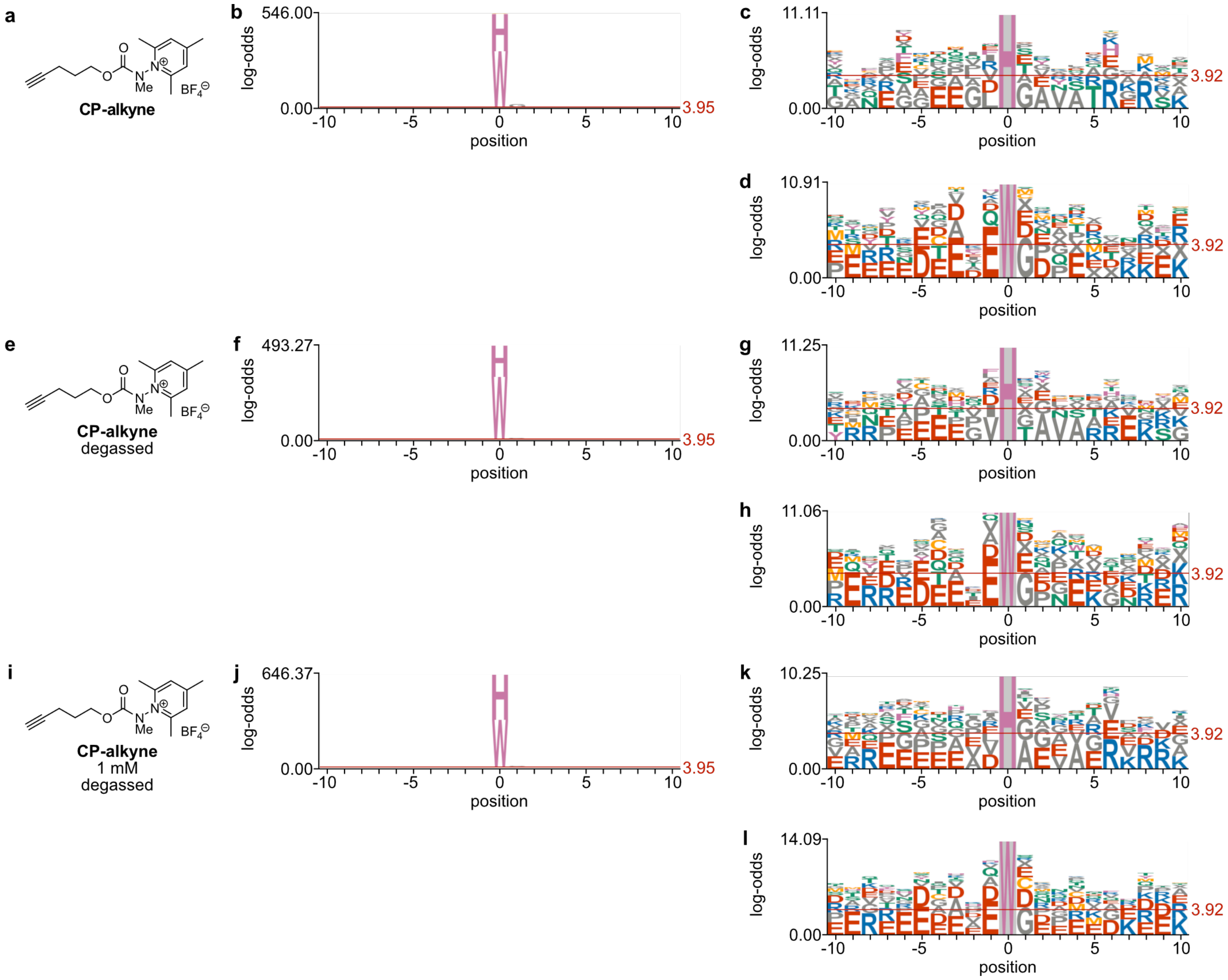

Supplementary Figure 63 | Sequence logos around sites modified by CP-alkyne. a,**e**,**i**, Structure of the probe for treatment of the proteome of *S. aureus* SH1000 at 100 μM (**a**,**e**) or 1 mM (**i**) probe concentration either open to air (**a**) or in a degassed solution under argon (**e**,**i**). The samples were irradiated for 10 min at 280-315 nm after 30 min of treatment with the probe. During the 30 min pre-treatment the samples were degassed by passing argon through the solution if indicated. **b**-**d**,**f**-**h**,**j**-**l**, Sequence logos determined using pLogo[17] based on the modified sites identified in the Mass Offset Searches. Either the sequence logos for all modified sequences (**b**,**f**,**j**) or the sequence logos for a subset of sites that are modified at histidine (**c**,**g**,**k**) or tryptophan (**d**,**h**,**l**) are shown. Position 0 indicates the modified site. The y-axis shows the log-odds of the binomial probability (log-odds) for the shown amino acids. The red line indicates the cut-off of $p < 0.05$. X indicates a residue that is outside of the sequence of the protein (before the *N*-terminus for positions −10 to −1 or after the *C*-terminus for positions 1 to 10) All data is based on technical duplicates.

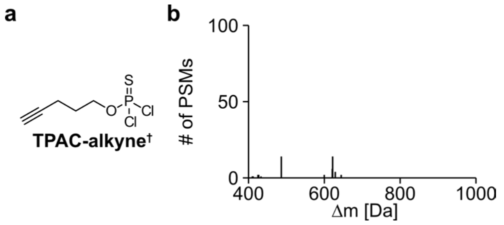

Supplementary Figure 64 | Masses of modification for TPAC-alkyne. a, Structure of the probe that was used for treatment of the proteome of *S. aureus* SH1000 at 100 µM probe concentration. **b**, Masses of modification determined through analysis with an Open Search in MSFragger[11, 12]-based FragPipe. All data is based on technical duplicates. †: No clear mass of modification was detected and therefore no analysis of the amino acid selectivity was possible.

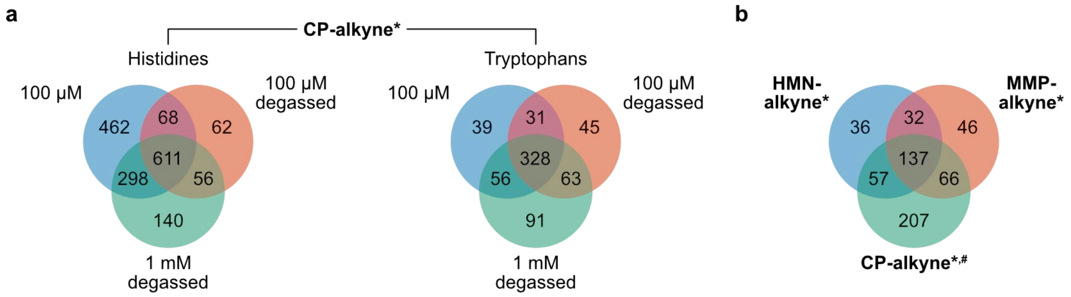

Supplementary Figure 65 | Venn-diagrams of the quantified histidines with CP-alkyne and tryptophans with CP-, HMN- and MMP-alkyne. a, Comparison of the histidines and tryptophans quantified with **CP-alkyne** under different conditions. **b**, Comparison of the three probes used to monitor tryptophan. Data is based on treatment of the proteome of *S. aureus* SH1000 at 100 µM probe concentration unless otherwise indicated. *: Labelling was performed using UV-activation at 280-315 nm (**CP-** and **MMP-alkyne**) or 365 nm (**HMN-alkyne**) for 10 min. #: Labelling was performed in degassed lysate under argon. All data is based on technical duplicates.

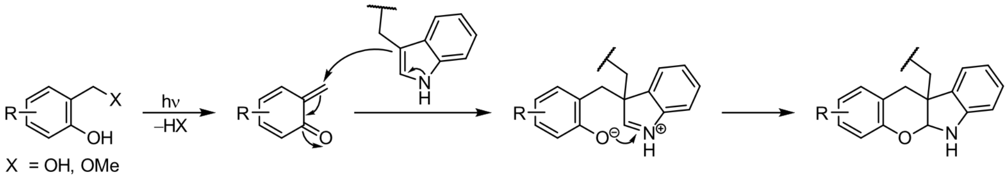

Supplementary Figure 66 | Mechanism of tryptophan-labelling through reaction of the indole with *in situ* generated *ortho*-quinone methides (from HMN-, HMP- and MMP-alkyne) as described by Lin *et al.*[37] Alternatively, the reaction could also proceed through a concerted [4+2]-cycloaddition as reported by Uyanik *et al.*[38]

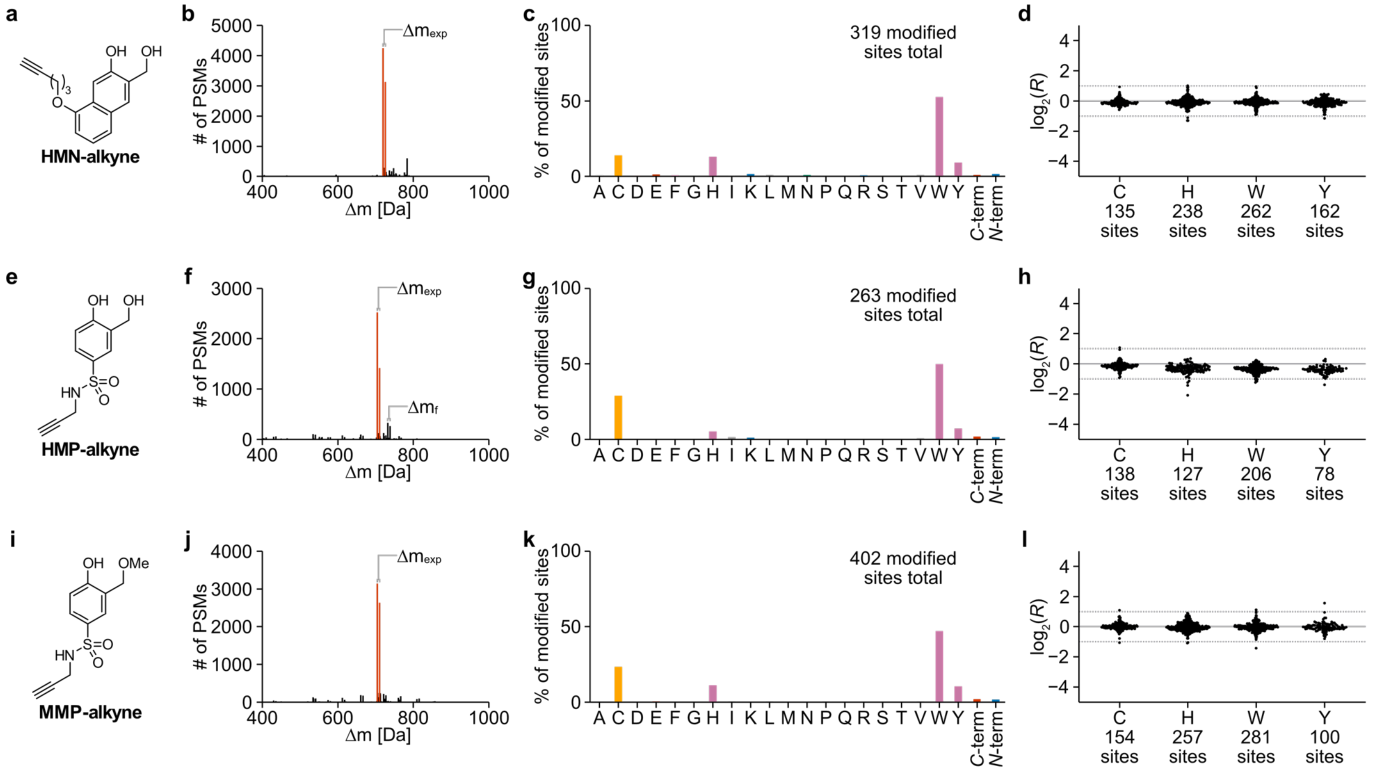

Supplementary Figure 67 | Masses of modification, amino acid selectivity and quantification for probes that react as *ortho*-quinone methides after photoactivation. **a**,**e**,**i**, Structures of the probes that were used for treatment of the proteome of *S. aureus* SH1000 at 100 μM probe concentration. The samples were irradiated for 10 min at 365 nm (**a**) or at 280-315 nm (**e**,**i**) after 30 min of treatment with the probe. **b**,**f**,**j**, Masses of modification determined through analysis with an Open Search in MSFragger[11, 12]-based FragPipe. The peaks highlighted in red are the masses that have been selected for further analysis within the same row. The expected masses are labelled as Δm_{exp}. Further modification of the modified peptides by formylation (Δm_f) is also indicated if the respective peaks were detected. **c**,**g**,**k**, One peak pair (indicated in red in the same row) is selected for a Mass Offset Search[11] that localizes this modification to the modified amino acid(s). In this way, selectivity is assessed across all proteinogenic amino acids. The bar graph represents the fraction of all modified sites that is modified at the indicated amino acid. *C*-term = *C*-terminal modification. *N*-term = *N*-terminal modification. **d**,**h**,**l**, Specific amino acid(s) are selected for quantification at the selected masses (indicated in red in the same row) using a Closed Search[11] and the IonQuant[13] feature. The heavy and light samples were mixed at a ratio of 1:1. The grey, solid line indicates the expected values of $\log_2(R) = 0$. The grey, dashed lines indicate the preferred window of quantification ($-1 < \log_2(R) < 1$). All data is based on technical duplicates.

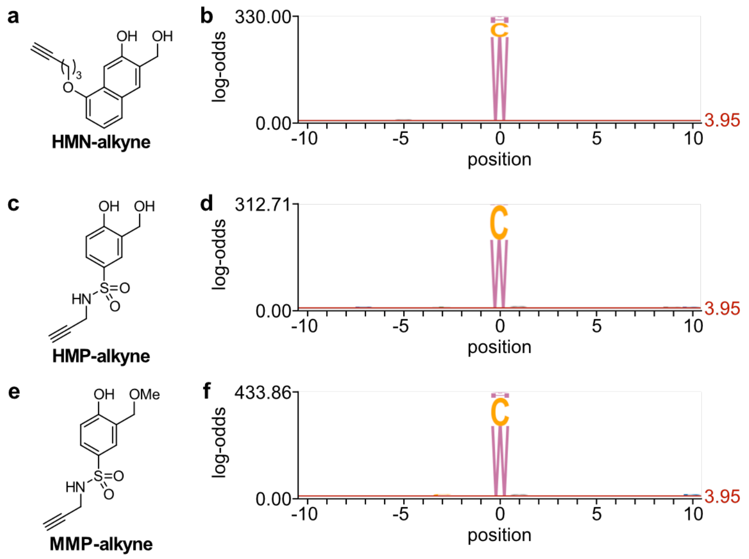

Supplementary Figure 68 | Sequence logos around sites modified by HMN-, HMP-, and MMP-alkyne. **a**,**c**,**e**, Structures of the probes that were used for treatment of the proteome of *S. aureus* SH1000 at 100 μM probe concentration. The samples were irradiated for 10 min at 365 nm (**a**) or at 280-315 nm (**c**,**e**) after 30 min of treatment with the probe. **b**,**d**,**f**, Sequence logo for all modified sequences determined using pLogo[17] based on the modified sites identified in the Mass Offset Searches. Position 0 indicates the modified site. The y-axis shows the log-odds of the binomial probability (log-odds) for the shown amino acids. The red line indicates the cut-off of $p < 0.05$. X indicates a residue that is outside of the sequence of the protein (before the *N*-terminus for positions −10 to −1 or after the *C*-terminus for positions 1 to 10) All data is based on technical duplicates.

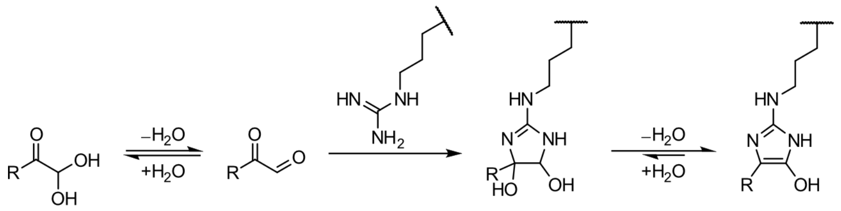

Supplementary Figure 69 | Reaction of a glyoxal (hydrate) (PhGO-alkyne) with arginine as described by Thompson *et al.*[39]

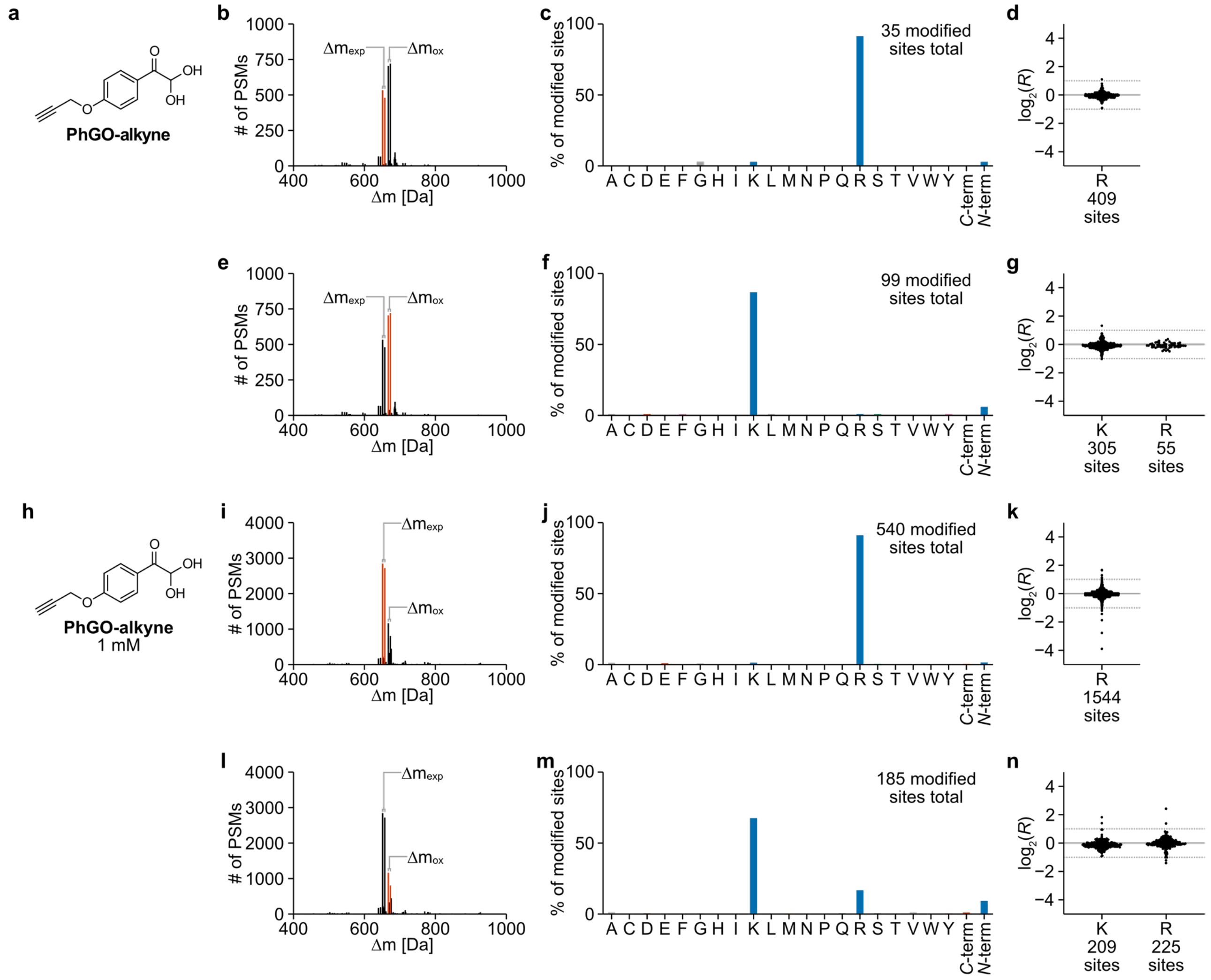

Supplementary Figure 70 | Masses of modification, amino acid selectivity and quantification for PhGO-alkyne. a,**h**, Structure of the probe that was used for treatment of the proteome of *S. aureus* SH1000 at 100 µM (**a**) or 1 mM (**h**) probe concentration. **b**,**e**,**i**,**l**, Masses of modification determined through analysis with an Open Search in MSFragger[11, 12]-based FragPipe. The peaks highlighted in red are the masses that have been selected for further analysis within the same row. The expected masses are labelled as Δm_{exp}. Further modification of the modified peptides by oxidation (Δm_{ox}) is also indicated. **c**,**f**,**j**,**m**, One peak pair (indicated in red in the same row) is selected for a Mass Offset Search[11] that localizes this modification to the modified amino acid(s). In this way, selectivity is assessed across all proteinogenic amino acids. The bar graph represents the fraction of all modified sites that is modified at the indicated amino acid. *C*-term = *C*-terminal modification. *N*-term = *N*-terminal modification. **d**,**g**,**k**,**n**, Specific amino acid(s) are selected for quantification at the selected masses (indicated in red in the same row) using a Closed Search[11] and the IonQuant[13] feature. The heavy and light samples were mixed at a ratio of 1:1. The grey, solid line indicates the expected values of $\log_2(R) = 0$. The grey, dashed lines indicate the preferred window of quantification ($-1 < \log_2(R) < 1$). All data is based on technical duplicates.

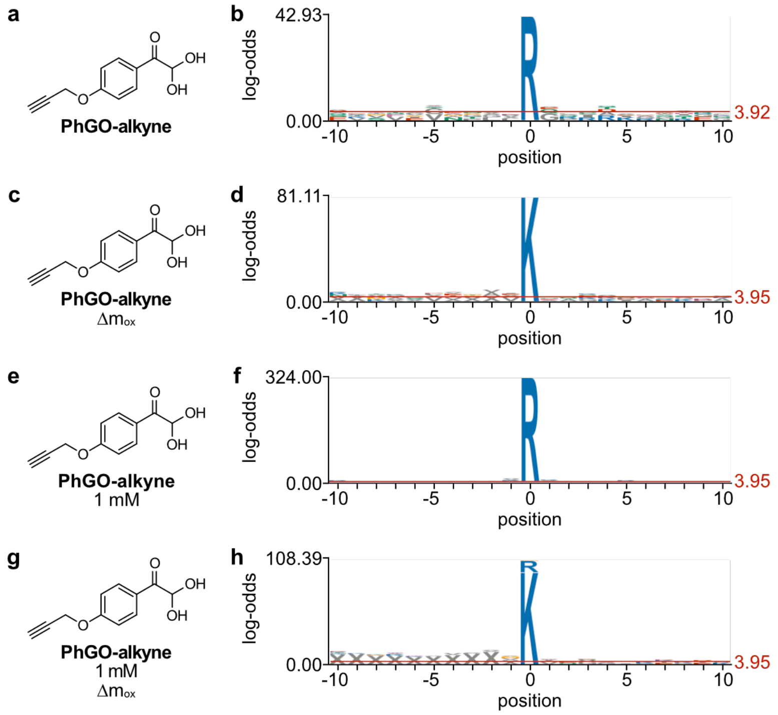

Supplementary Figure 71 | Sequence logos around sites modified by PhGO-alkyne. a,**c**,**e**,**g**, Structure of the probe that was used for treatment of the proteome of *S. aureus* SH1000 at 100 μM (**a**,**c**) or 1 mM (**e**,**g**) probe concentration. Oxidation (**c**,**g**) was detected as an additional modification. **b**,**d**,**f**,**h**, Sequence logo for all modified sequences determined using pLogo[17] based on the modified sites identified in the Mass Offset Searches. Position 0 indicates the modified site. The y-axis shows the log-odds of the binomial probability (log-odds) for the shown amino acids. The red line indicates the cut-off of $p < 0.05$. X indicates a residue that is outside of the sequence of the protein (before the *N*-terminus for positions −10 to −1 or after the *C*-terminus for positions 1 to 10) All data is based on technical duplicates.

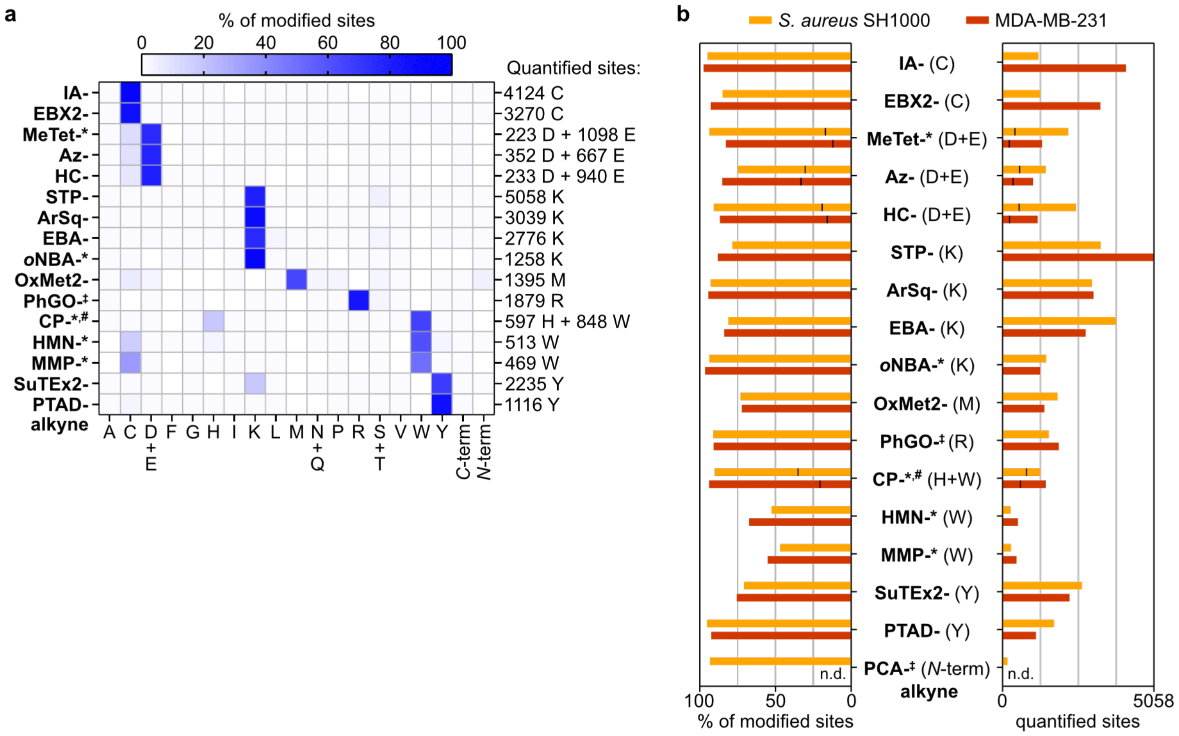

Supplementary Figure 72 | A set of 16 electrophilic probes that enables studying nine different amino acids in the lysate of the human cancer cell line MDA-MB-231. a, For a selection of the probes that allow studying diverse residues in the proteome, their selectivity is plotted in a heatmap. The colour is scaled by the fraction of all modified sites that is modified at the indicated amino acid. Samples were prepared using 100 μM of the indicated probe in the lysate of the human cancer cell line MDA-MB-231. **b**, Comparison of probe performance in the lysates of *S. aureus* SH1000 and the human cancer cell line MDA-MB-231. Selectivities and quantified sites for a probe are given for the amino acid indicated in parenthesis. In cases where two amino acids are aggregated, the distribution among those amino acids is indicated by a black line with aspartate or histidine making up the fraction starting from the zero line. *C*-term = *C*-terminal modification. *N*-term = *N*-terminal modification. n.d.: not detected. *: Labelling was performed using UV-activation at 280-315 nm (**MeTet-**, **CP-** and **MMP-alkyne**) or 365 nm (***o*NBA-** and **HMN-alkyne**) for 10 min. ‡: Data for the indicated probe at 1 mM is shown. #: Labelling was performed in degassed lysate under argon. All data is based on technical duplicates.

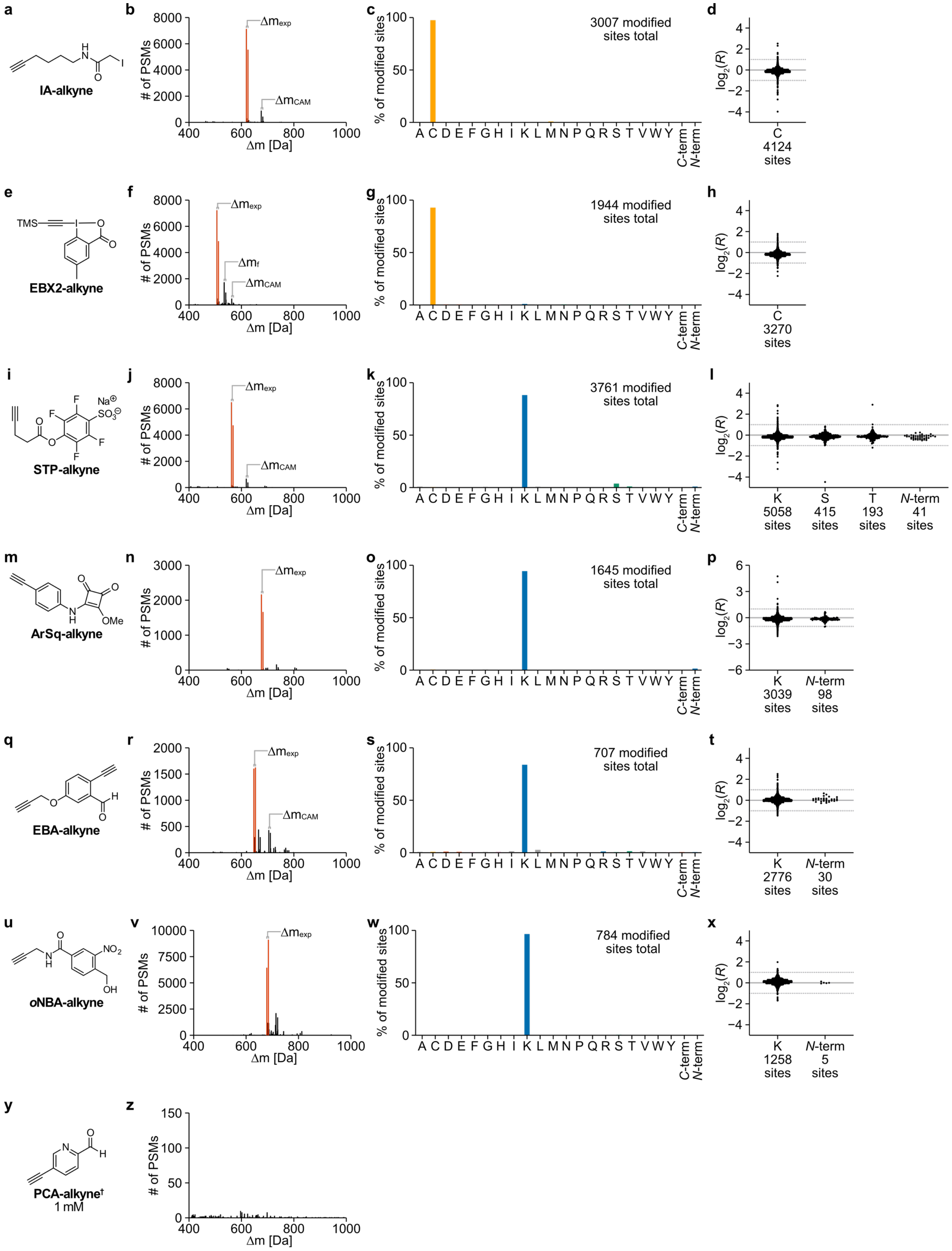

Supplementary Figure 73 | Masses of modification, amino acid selectivity and quantification for cysteine-, lysine- and protein *N*-terminus-directed probes in lysate of the human cancer cell line MDA-MB-231. **a**,**e**,**i**,**m**,**q**,**u**,**y**, Structures of the probes that were used for treatment of the proteome of MDA-MB-231 at 100 μM

(**a**,**e**,**i**,**m**,**q,u**) or 1 mM (**y**) probe concentration. For ***o*****NBA-alkyne**, the samples were irradiated for 10 min at 365 nm after 30 min of treatment with the probe. **b**,**f**,**j**,**n**,**r**,**v**,**z**, Masses of modification determined through analysis with an Open Search in MSFragger[11, 12]-based FragPipe. The peaks highlighted in red are the masses that have been selected for further analysis within the same row. The expected masses are labelled as Δm_{exp}. Further modification of the modified peptides by oxidation (Δm_{ox}) or carbamidomethylation on a second cysteine (Δm_{CAM}) is also indicated if the respective peaks were detected. **c**,**g**,**k**,**o**,**s**,**w**, One peak pair (indicated in red in the same row) is selected for a Mass Offset Search[11] that localizes this modification to the modified amino acid(s). In this way, selectivity is assessed across all proteinogenic amino acids. The bar graph represents the fraction of all modified sites that is modified at the indicated amino acid. *C*-term = *C*-terminal modification. *N*-term = *N*-terminal modification. **d**,**h**,**l**,**p**,**t**,**x**, Specific amino acid(s) are selected for quantification at the selected masses (indicated in red in the same row) using a Closed Search[11] and the IonQuant[13] feature. The heavy and light samples were mixed at a ratio of 1:1. The grey, solid line indicates the expected values of $\log_2(R) = 0$. The grey, dashed lines indicate the preferred window of quantification ($-1 < \log_2(R) < 1$). All data is based on technical duplicates. †: No clear mass of modification was detected and therefore no analysis of the amino acid selectivity was possible.

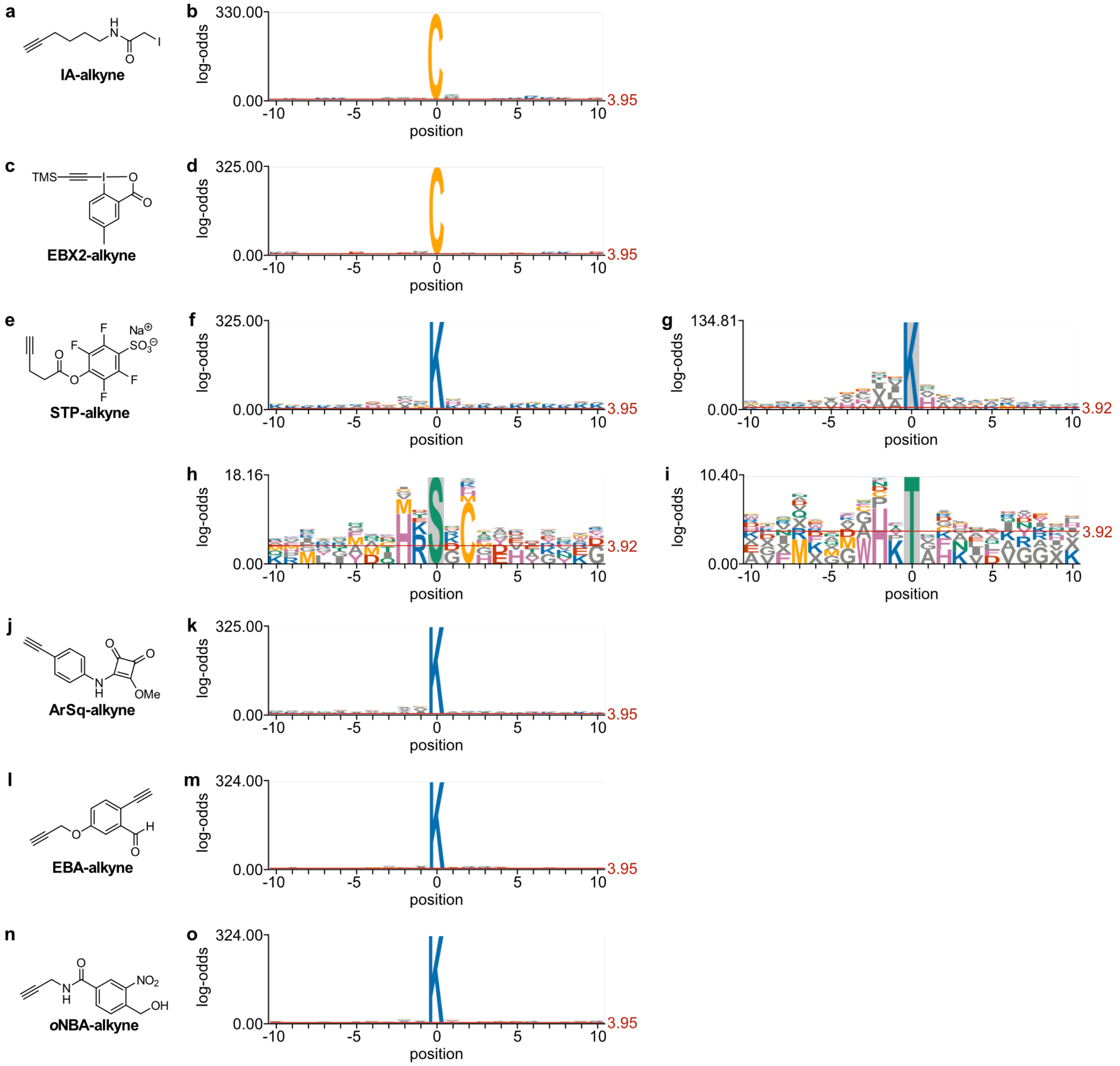

Supplementary Figure 74 | Sequence logos around sites modified by IA-, EBX2-, STP-, ArSq-, EBA-, and *o*NBA-alkyne in lysate of the human cancer cell line MDA-MB-231. a,**c**,**e**,**j**,**l**,**n**, Structures of the probes that were used for treatment of the proteome of MDA-MB-231 at 100 μM probe concentration. **b**,**d**,**f-i**,**k**,**m**,**o**, Sequence logos determined using pLogo[17] based on the modified sites identified in the Mass Offset Searches. Either the sequence logos for all modified sequences (**b**,**d**,**f**,**k**,**m**,**o**) or the sequence logos for a subset of sites that are modified at lysine (**g**), serine (**h**) or threonine (**i**) are shown. Position 0 indicates the modified site. The y-axis shows the log-odds of the binomial probability (log-odds) for the shown amino acids. The red line indicates the cut-off of $p < 0.05$. X indicates a residue that is outside of the sequence of the protein (before the *N*-terminus for positions −10 to −1 or after the *C*-terminus for positions 1 to 10) All data is based on technical duplicates.

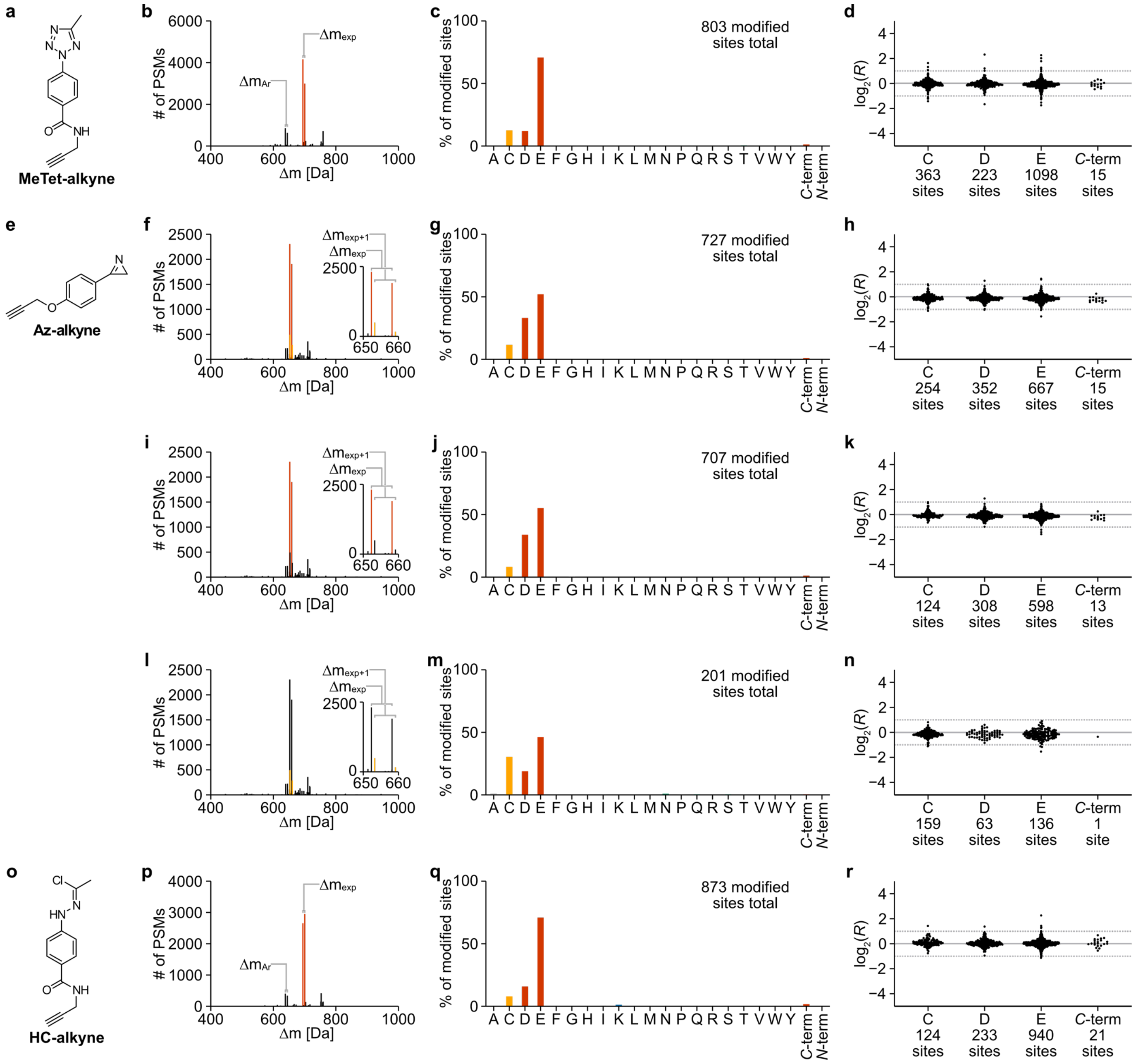

Supplementary Figure 75 | Masses of modification, amino acid selectivity and quantification for aspartate- and glutamate-directed probes in lysate of the human cancer cell line MDA-MB-231. a,**e**,**o**, Structures of the probes that were used for treatment of the proteome of MDA-MB-231 at 100 μM probe concentration. For **MeTet-alkyne**, the samples were irradiated for 10 min at 280-315 nm after 30 min of treatment with the probe. **b**,**f**,**i**,**l**,**p**, Masses of modification determined through analysis with an Open Search in MSFragger[11, 12]-based FragPipe. The peaks highlighted in red or yellow are the masses that have been selected for further analysis within the same row. The expected masses are labelled as Δm_{exp}. The expected mass +1 Da is labelled as Δm_{exp+1} if the respective peaks were detected. The additionally identified modification by arylation is additionally highlighted as Δm_{Ar} if the respective peaks were detected. **c**,**g**,**j**,**m**,**q**, One peak pair (indicated in red or yellow in the same row, **b**,**i**,**l**,**p**) or both peak pairs (indicated in red and yellow in the same row, **f**) are selected for a Mass Offset Search[11] that localizes this modification to the modified amino acid(s). In this way, selectivity is assessed across all proteinogenic amino acids. The bar graph represents the fraction of all modified sites that is modified at the indicated amino acid. *C*-term = *C*-terminal modification. *N*-term = *N*-terminal modification. **d**,**h**,**k**,**n**,**r**, Specific amino acid(s) are selected for quantification at the selected masses (indicated in red or yellow in the same row, **b**,**i**,**l**,**p**, or indicated in red and yellow in the same row, **f**) using a Closed Search[11] and the IonQuant[13] feature. The heavy and light samples were mixed at a ratio of 1:1. The grey, solid line indicates the expected values of $\log_2(R) = 0$. The grey, dashed lines indicate the preferred window of quantification ($-1 < \log_2(R) < 1$). All data is based on technical duplicates.

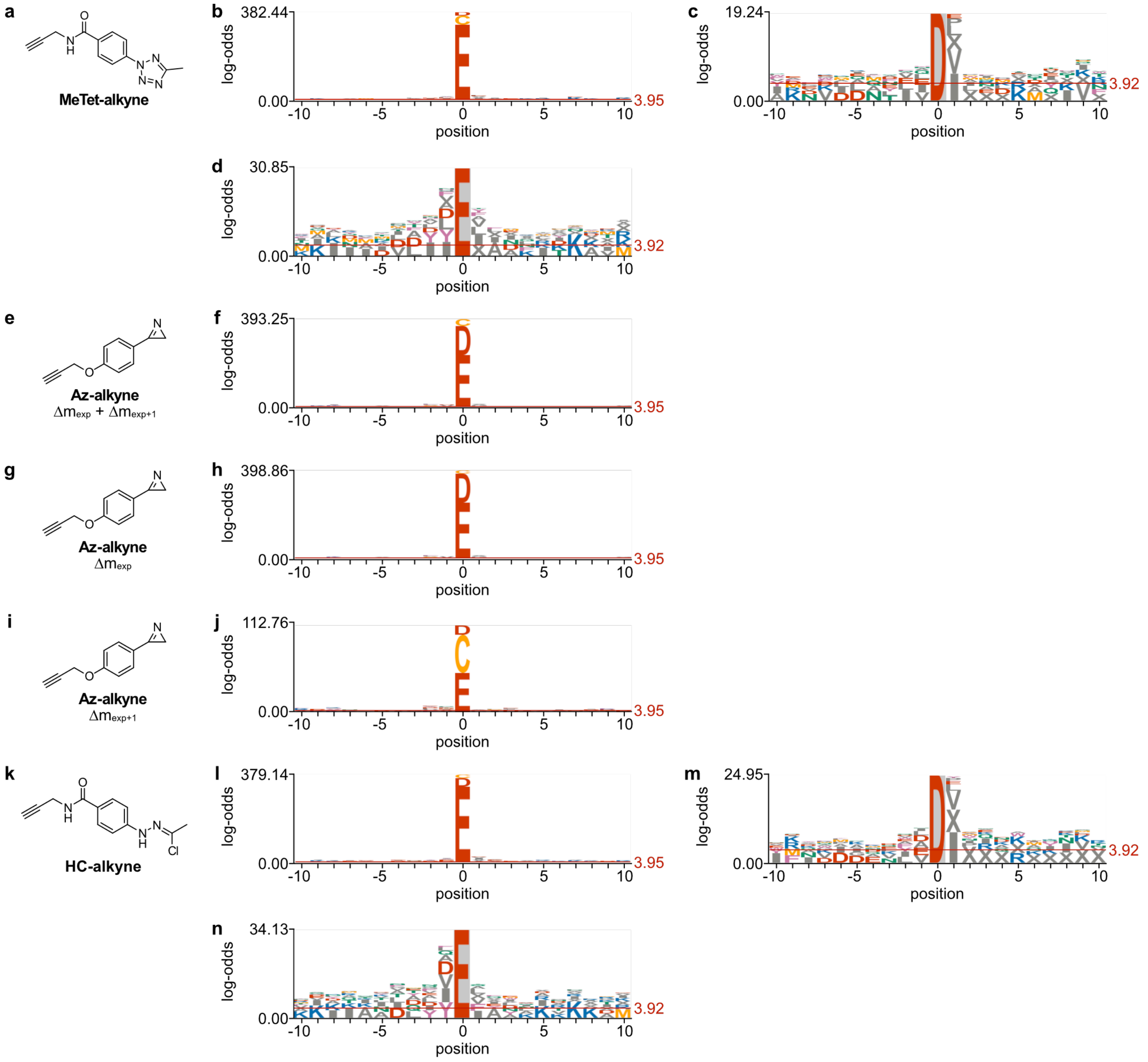

Supplementary Figure 76 | Sequence logos around sites modified by MeTet-, Az-, and HC-alkyne in lysate of the human cancer cell line MDA-MB-231. a,**e**,**g**,**i**,**k**, Structures of the probes that were used for treatment of the proteome of MDA-MB-231 at 100 μM probe concentration. For **MeTet-alkyne**, the samples were irradiated for 10 min at 280-315 nm after 30 min of treatment with the probe. For **Az-alkyne**, it was not possible to fully differentiate the modifications Δm_{exp} and Δm_{exp+1} computationally and so they were analysed together (**e**) and in separate (**g**,**i**) runs. **b-d**,**f**,**h**,**j**,**l-n**, Sequence logos determined using pLogo[17] based on the modified sites identified in the Mass Offset Searches. Either the sequence logos for all modified sequences (**b**,**f**,**h**,**j**,**l**) or the sequence logos for a subset of sites that are modified at aspartate (**c**,**m**) or glutamate (**d**,**n**) are shown. Position 0 indicates the modified site. The y-axis shows the log-odds of the binomial probability (log-odds) for the shown amino acids. The red line indicates the cut-off of $p < 0.05$. X indicates a residue that is outside of the sequence of the protein (before the *N*-terminus for positions −10 to −1 or after the *C*-terminus for positions 1 to 10) All data is based on technical duplicates.

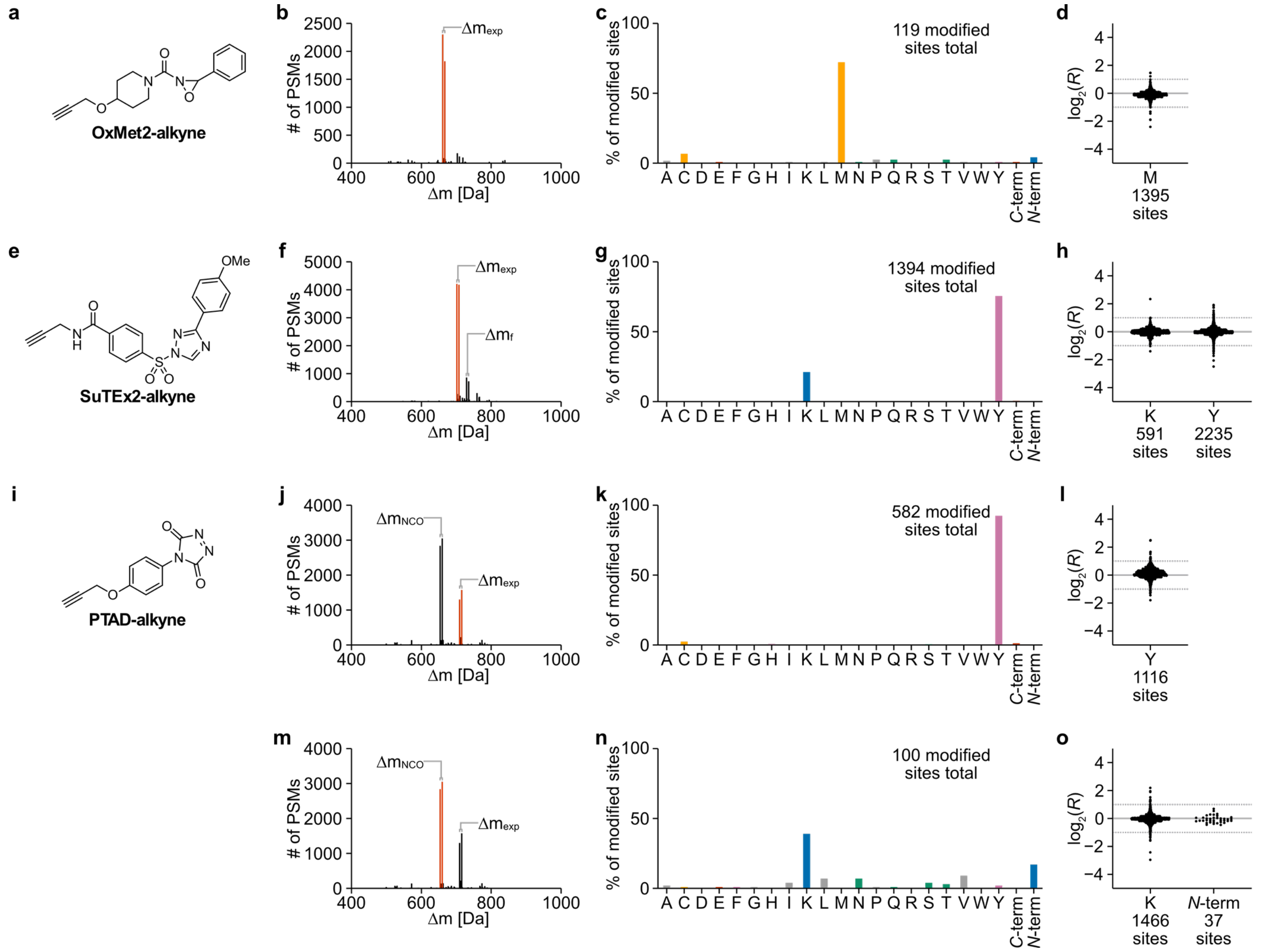

Supplementary Figure 77 | Masses of modification, amino acid selectivity and quantification for methionine- and tyrosine-directed probes in lysate of the human cancer cell line MDA-MB-231. a,**e**,**i**, Structures of the probes that were used for treatment of the proteome of MDA-MB-231 at 100 μM probe concentration. **b**,**f**,**j**,**m**, Masses of modification determined through analysis with an Open Search in MSFragger[11, 12]-based FragPipe. The peaks highlighted in red are the masses that have been selected for further analysis within the same row. The expected masses are labelled as Δm_{exp}. Further modification of the modified peptides by formylation (Δm_{f}) is also indicated if the respective peaks were detected. The mass that corresponds to reactivity through formation of the isocyanate is labelled as Δm_{NCO}. **c**,**g**,**k**,**n**, One peak pair (indicated in red in the same row) is selected for a Mass Offset Search[11] that localizes this modification to the modified amino acid(s). In this way, selectivity is assessed across all proteinogenic amino acids. The bar graph represents the fraction of all modified sites that is modified at the indicated amino acid. *C*-term = *C*-terminal modification. *N*-term = *N*-terminal modification. **d**,**h**,**l**,**o**, Specific amino acid(s) are selected for quantification at the selected masses (indicated in red in the same row) using a Closed Search[11] and the IonQuant[13] feature. The heavy and light samples were mixed at a ratio of 1:1. The grey, solid line indicates the expected values of $\log_2(R) = 0$. The grey, dashed lines indicate the preferred window of quantification ($-1 < \log_2(R) < 1$). All data is based on technical duplicates.

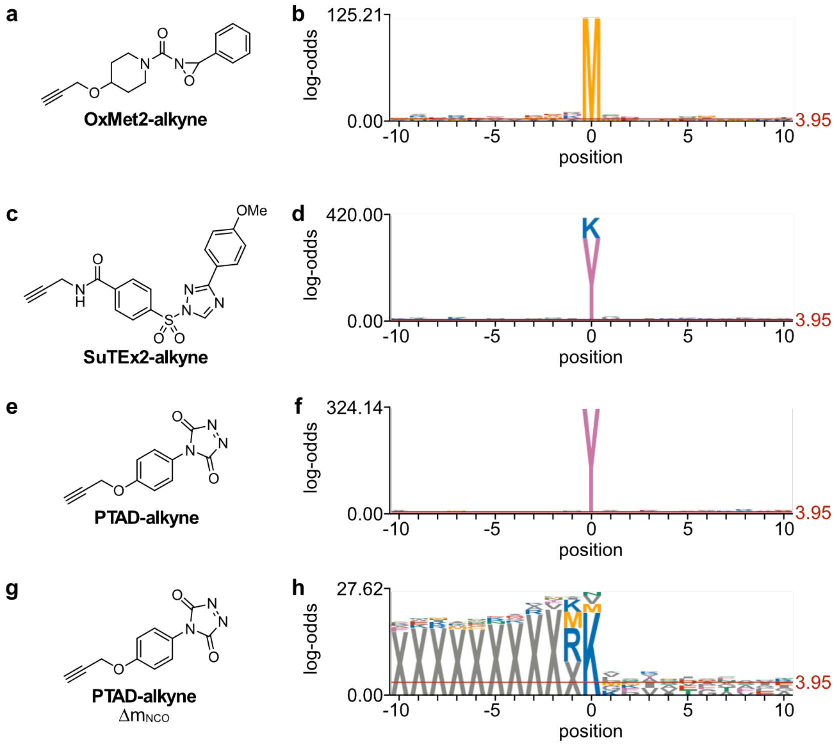

Supplementary Figure 78 | Sequence logos around sites modified by OxMet2-, SuTEx2- and PTAD-alkyne in lysate of the human cancer cell line MDA-MB-231. **a**,**c**,**e**,**g**, Structures of the probes that were used for treatment of the proteome of MDA-MB-231 at 100 μM probe concentration. For **PTAD-alkyne**, reaction with the isocyanate (**g**) resulting from fragmentation of the probe was detected as an additional modification. **b**,**d**,**f**,**h**, Sequence logo for all modified sequences determined using pLogo[17] based on the modified sites identified in the Mass Offset Searches. Position 0 indicates the modified site. The y-axis shows the log-odds of the binomial probability (log-odds) for the shown amino acids. The red line indicates the cut-off of $p < 0.05$. X indicates a residue that is outside of the sequence of the protein (before the *N*-terminus for positions −10 to −1 or after the *C*-terminus for positions 1 to 10) All data is based on technical duplicates.

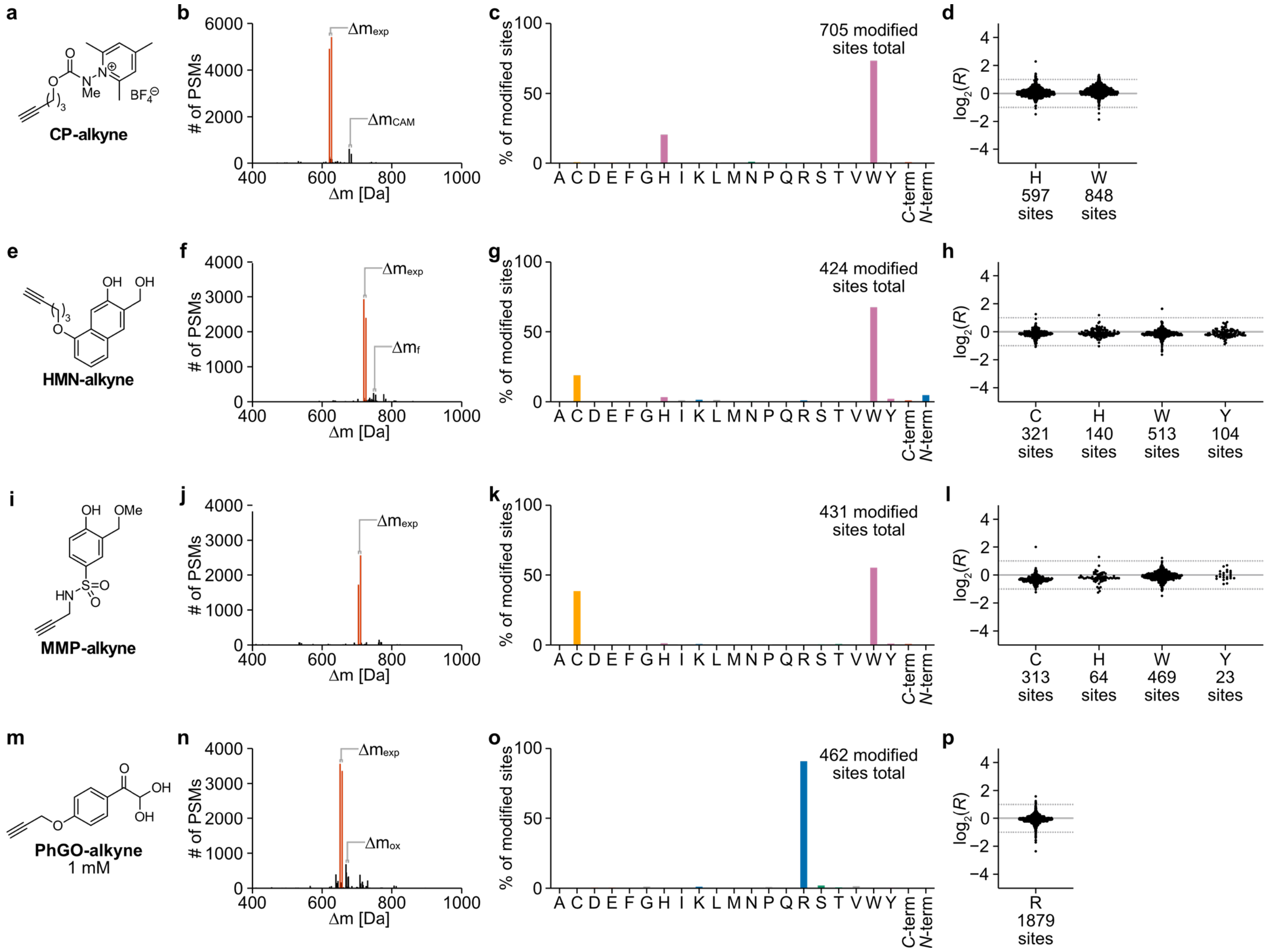

Supplementary Figure 79 | Masses of modification, amino acid selectivity and quantification for tryptophan- and arginine-directed probes in lysate of the human cancer cell line MDA-MB-231. a,**e**,**i**,**m**, Structures of the probes that were used for treatment of the proteome of MDA-MB-231 at 100 µM (**a**,**e**,**i**) or 1 mM (**m**) probe concentration. For **CP-** and **MMP-alkyne**, the samples were irradiated for 10 min at 280-315 nm after 30 min of treatment with the probe. For **HMN-alkyne**, the samples were irradiated for 10 min at 365 nm after 30 min of treatment with the probe. In the case of **CP-alkyne**, during the 30 min pre-treatment the samples were degassed by passing argon through the solution. **b**,**f**,**j**,**n**, Masses of modification determined through analysis with an Open Search in MSFragger[11, 12]-based FragPipe. The peaks highlighted in red are the masses that have been selected for further analysis within the same row. The expected masses are labelled as Δm_{exp}. Further modification of the modified peptides by formylation (Δm_f) or carbamidomethylation on a second cysteine (Δm_{CAM}) is also indicated if the respective peaks were detected. **c**,**g**,**k**,**o**, One peak pair (indicated in red in the same row) is selected for a Mass Offset Search[11] that localizes this modification to the modified amino acid(s). In this way, selectivity is assessed across all proteinogenic amino acids. The bar graph represents the fraction of all modified sites that is modified at the indicated amino acid. *C*-term = *C*-terminal modification. *N*-term = *N*-terminal modification. **d**,**h**,**l**,**p**, Specific amino acid(s) are selected for quantification at the selected masses (indicated in red in the same row) using a Closed Search[11] and the IonQuant[13] feature. The heavy and light samples were mixed at a ratio of 1:1. The grey, solid line indicates the expected values of $\log_2(R) = 0$. The grey, dashed lines indicate the preferred window of quantification ($-1 < \log_2(R) < 1$). All data is based on technical duplicates.

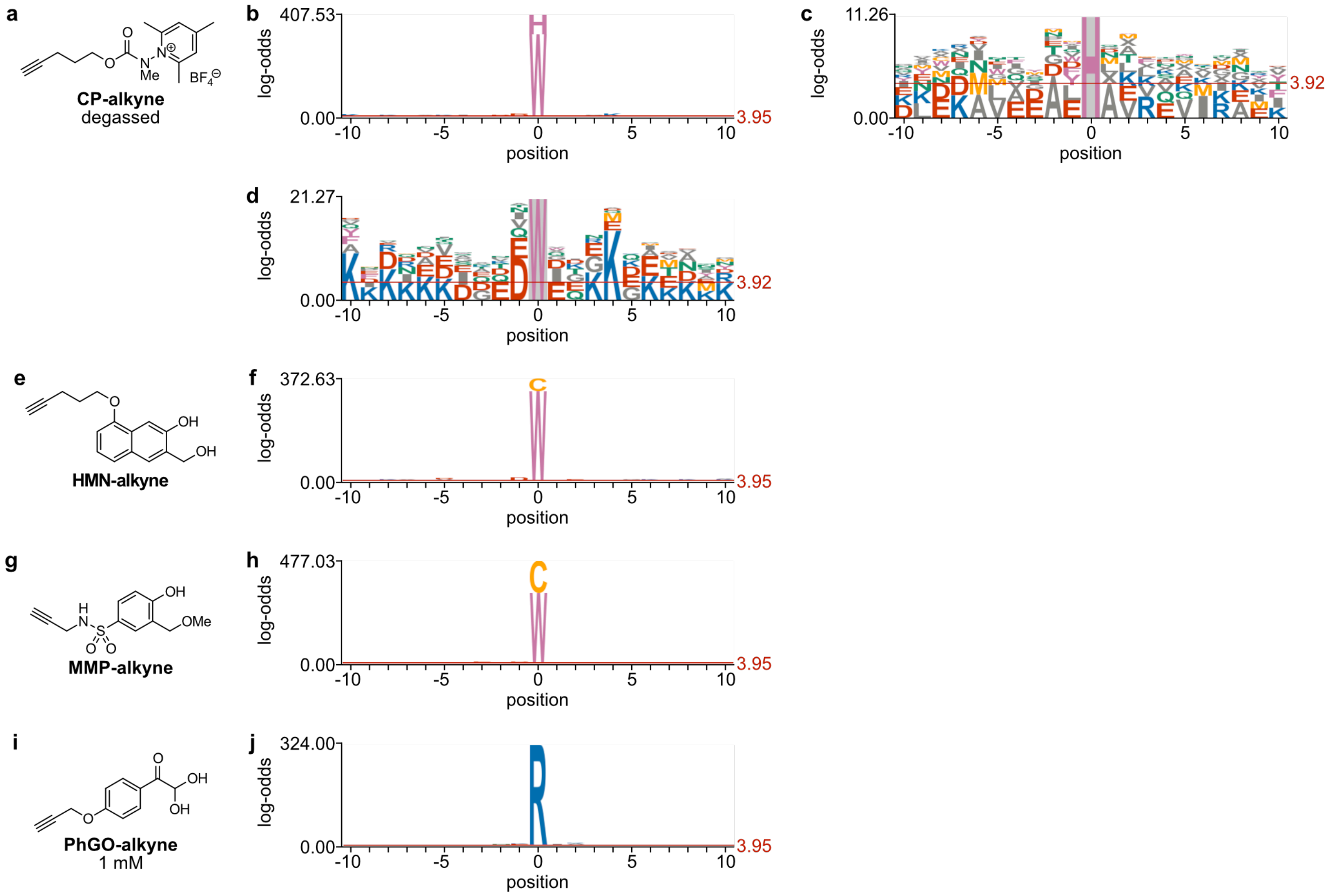

Supplementary Figure 80 | Sequence logos around sites modified by HMN-, MMP-, CP-, and PhGO-alkyne in lysate of the human cancer cell line MDA-MB-231. a,**e**,**g**,**i**, Structures of the probes that were used for treatment of the proteome of MDA-MB-231 at 100 µM (**a**,**e**,**g**) or 1 mM (**i**) probe concentration. For **CP-** and **MMP-alkyne**, the samples were irradiated for 10 min at 280-315 nm after 30 min of treatment with the probe. For **HMN-alkyne**, the samples were irradiated for 10 min at 365 nm after 30 min of treatment with the probe. In the case of **CP-alkyne**, during the 30 min pre-treatment the samples were degassed by passing argon through the solution. **b**-**d**,**f**,**h**,**j**, Sequence logos determined using pLogo[17] based on the modified sites identified in the Mass Offset Searches. Either the sequence logos for all modified sequences (**b**,**f**,**h**,**j**) or the sequence logos for a subset of sites that are modified at histidine (**c**) or tryptophan (**d**) are shown. Position 0 indicates the modified site. The y-axis shows the log-odds of the binomial probability (log-odds) for the shown amino acids. The red line indicates the cut-off of $p < 0.05$. X indicates a residue that is outside of the sequence of the protein (before the *N*-terminus for positions −10 to −1 or after the *C*-terminus for positions 1 to 10) All data is based on technical duplicates.

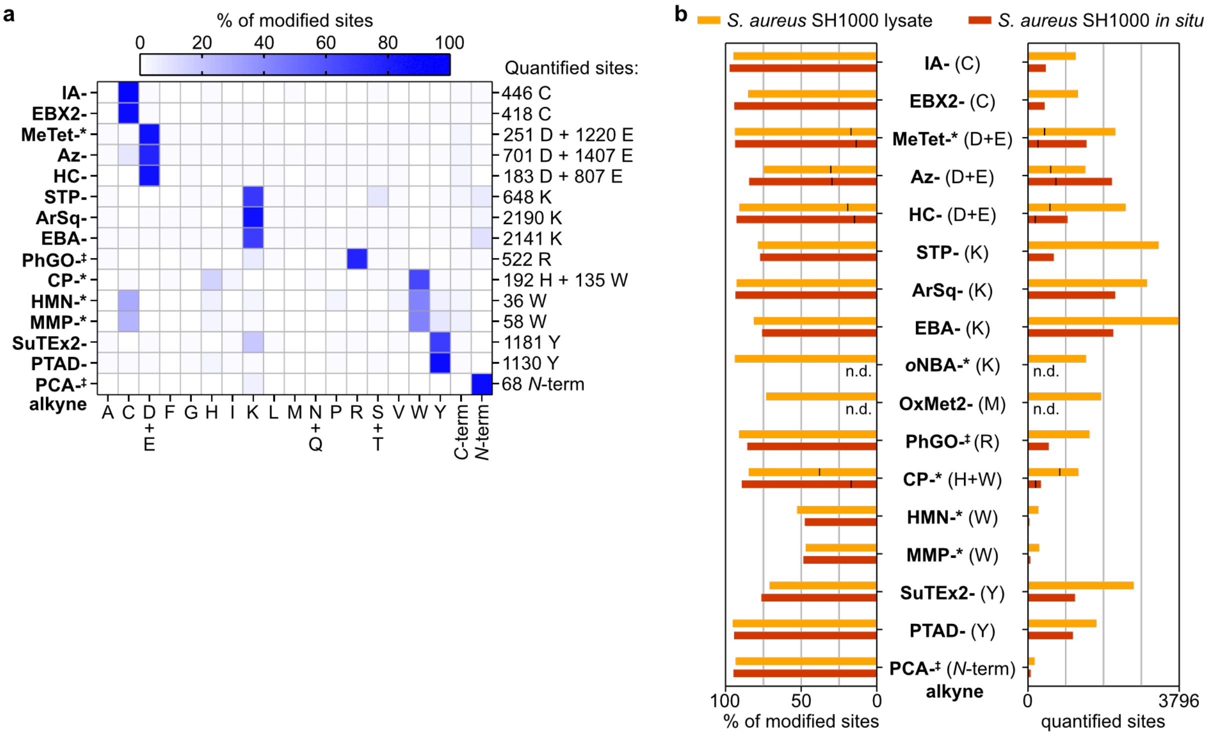

Supplementary Figure 81 | A set of 15 electrophilic probes that enables studying eight different amino acids and the protein *N*-terminus *in situ*. a, The selectivities of probes that allow studying diverse residues in the proteome are plotted in a heatmap. The colour is scaled by the fraction of all modified sites that is modified at the indicated amino acid. Samples were prepared using 100 μM of the indicated probe in live cells of *S. aureus* SH1000. **b**, Comparison of probe performance in live cells and lysates of *S. aureus* SH1000. Selectivities and quantified sites for a probe are given for the amino acid indicated in parenthesis. In cases where two amino acids are aggregated, the distribution among those amino acids is indicated by a black line with aspartate or histidine making up the fraction starting from the zero line. *C*-term = *C*-terminal modification. *N*-term = *N*-terminal modification. n.d.: not detected. *: Labelling was performed using UV-activation at 280-315 nm (**MeTet-**, **CP-** and **MMP-alkyne**) or 365 nm (***o*NBA-** and **HMN-alkyne**) for 10 min. ‡: Data for the indicated probe at 1 mM is shown. All data is based on technical duplicates.

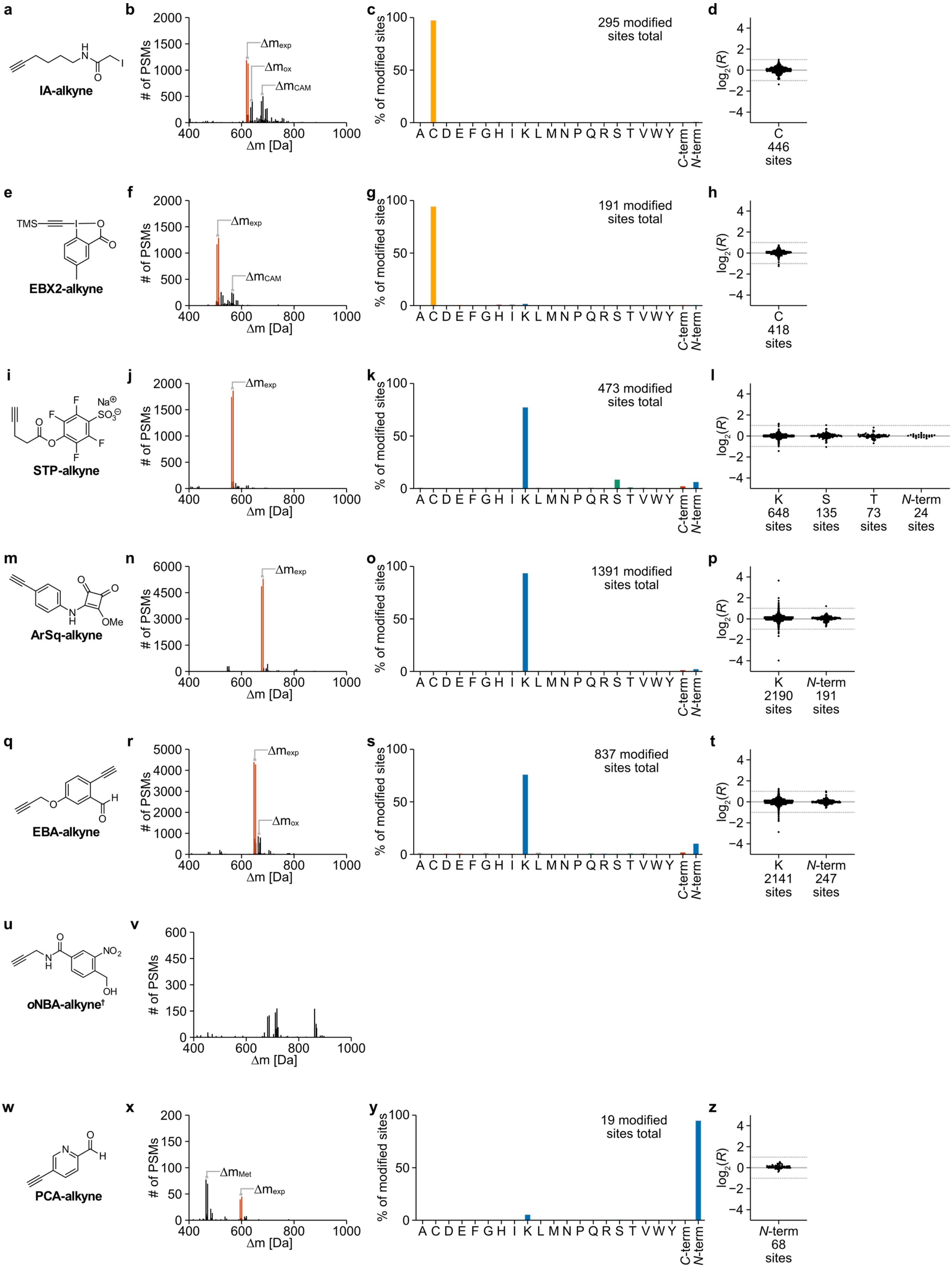

Supplementary Figure 82 | Masses of modification, amino acid selectivity and quantification for cysteine-, lysine- and protein *N*-terminus-directed probes *in situ*. a,**e**,**i**,**m**,**q**,**u**,**w**, Structures of the probes that were used for *in situ* treatment of *S. aureus* SH1000 at 100 μM (**a**,**e**,**i**,**m**,**q**,**u**) or 1 mM (**w**) probe concentration. For ***o*NBA-alkyne**,

the samples were irradiated for 10 min at 365 nm after 30 min of treatment with the probe. **b**,**f**,**j**,**n**,**r**,**v**,**x**, Masses of modification determined through analysis with an Open Search in MSFragger[11, 12]-based FragPipe. The peaks highlighted in red are the masses that have been selected for further analysis within the same row. The expected masses are labelled as Δm_{exp}. Further modification of the modified peptides by oxidation (Δm_{ox}) or carbamidomethylation on a second cysteine (Δm_{CAM}) is also indicated if the respective peaks were detected. **c**,**g**,**k**,**o**,**s**,**y**, One peak pair (indicated in red in the same row) is selected for a Mass Offset Search[11] that localizes this modification to the modified amino acid(s). In this way, selectivity is assessed across all proteinogenic amino acids. The bar graph represents the fraction of all modified sites that is modified at the indicated amino acid. *C*-term = *C*-terminal modification. *N*-term = *N*-terminal modification. **d**,**h**,**l**,**p**,**t**,**z**, Specific amino acid(s) are selected for quantification at the selected masses (indicated in red in the same row) using a Closed Search[11] and the IonQuant[13] feature. The heavy and light samples were mixed at a ratio of 1:1. The grey, solid line indicates the expected values of $\log_2(R) = 0$. The grey, dashed lines indicate the preferred window of quantification ($-1 < \log_2(R) < 1$). All data is based on technical duplicates. †: No clear mass of modification was detected and therefore no analysis of the amino acid selectivity was possible.

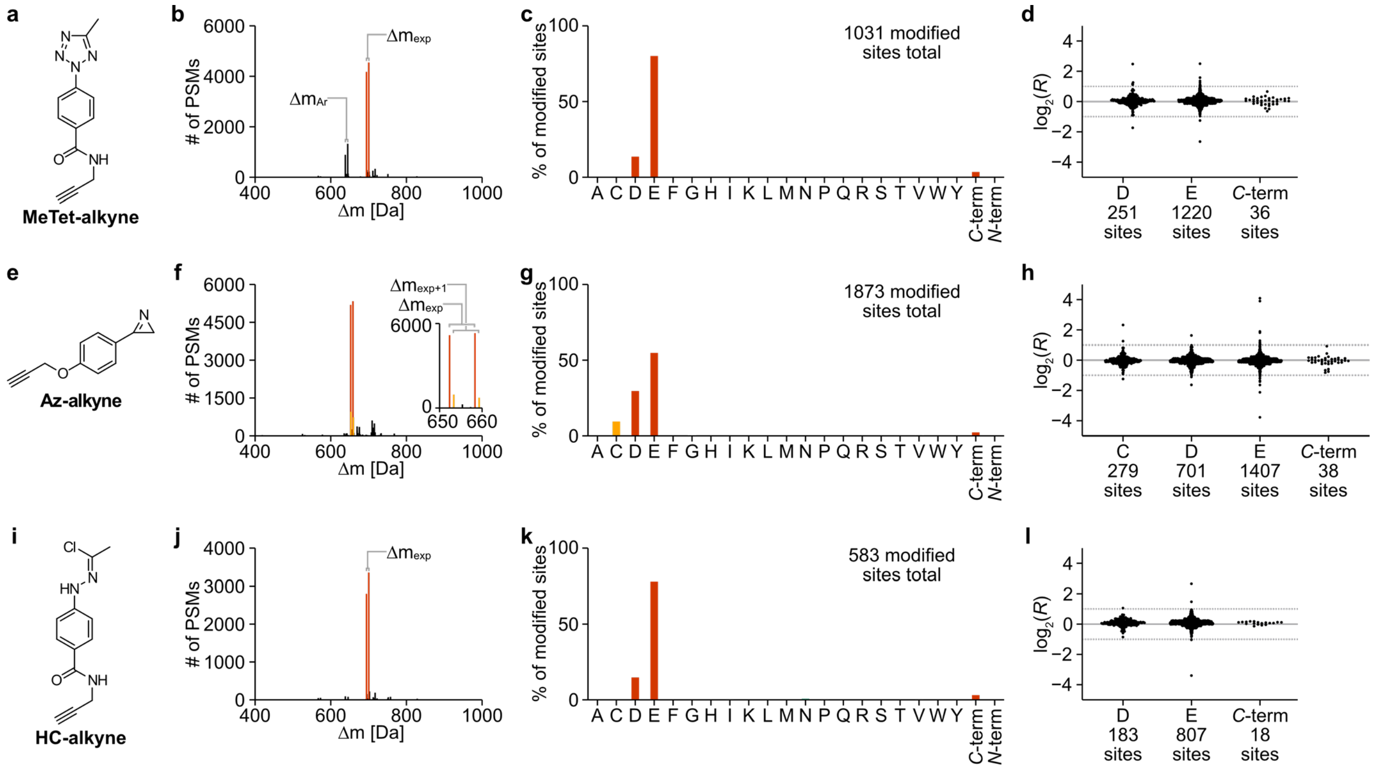

Supplementary Figure 83 | Masses of modification, amino acid selectivity and quantification for aspartate- and glutamate-directed probes *in situ*. **a**,**e**,**i**, Structures of the probes that were used *in situ* treatment of *S. aureus* SH1000 at 100 µM probe concentration. For **MeTet-alkyne**, the samples were irradiated for 10 min at 280-315 nm after 30 min of treatment with the probe. **b**,**f**,**i**, Masses of modification determined through analysis with an Open Search in MSFragger[11, 12]-based FragPipe. The peaks highlighted in red or yellow are the masses that have been selected for further analysis within the same row. The expected masses are labelled as Δm_{exp}. The expected mass +1 Da is labelled as Δm_{exp+1} if the respective peaks were detected. The additionally identified modification by arylation is additionally highlighted as Δm_{Ar} if the respective peaks were detected. **c**,**g**,**k**, One peak pair (indicated in red in the same row, **b**,**j**) or both peak pairs (indicated in red and yellow in the same row, **f**) are selected for a Mass Offset Search[11] that localizes this modification to the modified amino acid(s). In this way, selectivity is assessed across all proteinogenic amino acids. The bar graph represents the fraction of all modified sites that is modified at the indicated amino acid. *C*-term = *C*-terminal modification. *N*-term = *N*-terminal modification. **d**,**h**,**l**, Specific amino acid(s) are selected for quantification at the selected masses (indicated in red or yellow in the same row, **b**,**j**, or indicated in red and yellow in the same row, **f**) using a Closed Search[11] and the IonQuant[13] feature. The heavy and light samples were mixed at a ratio of 1:1. The grey, solid line indicates the expected values of $\log_2(R) = 0$. The grey, dashed lines indicate the preferred window of quantification ($-1 < \log_2(R) < 1$). All data is based on technical duplicates.

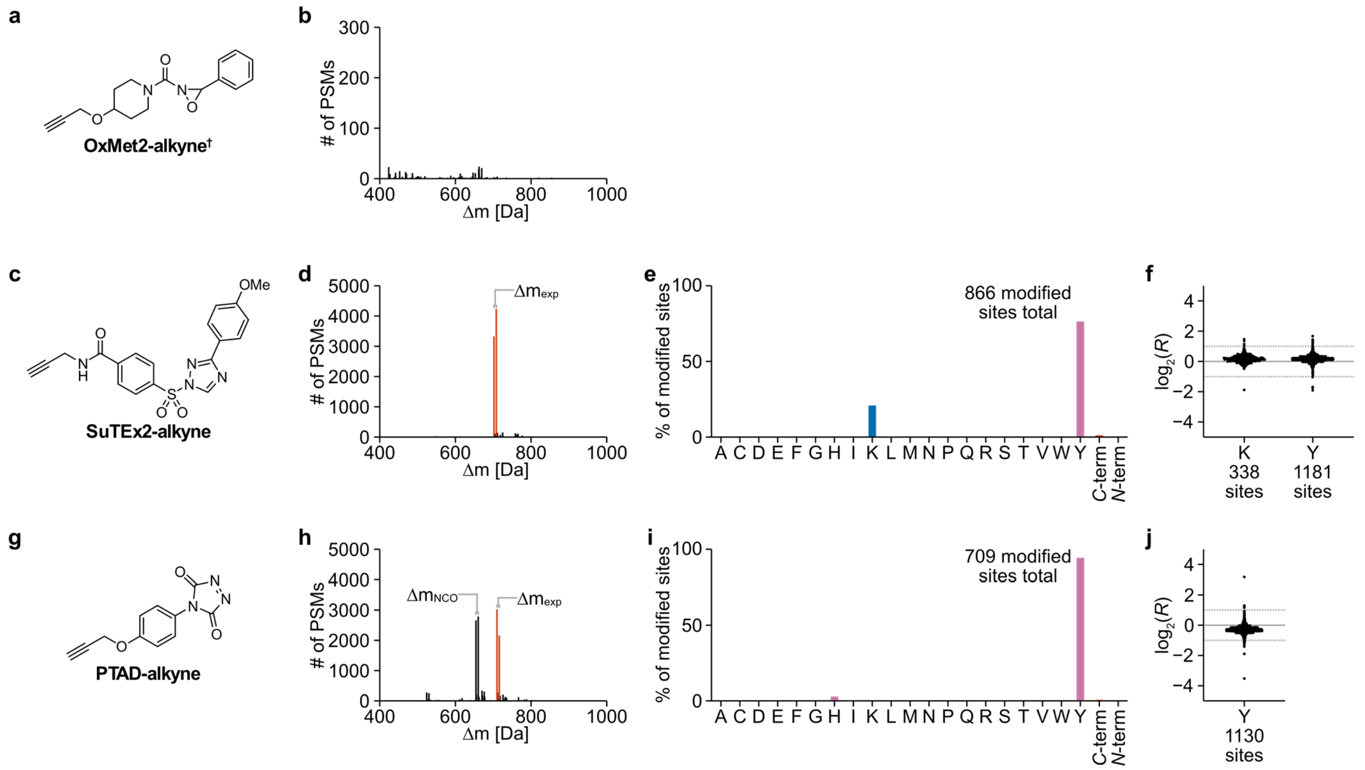

Supplementary Figure 84 | Masses of modification, amino acid selectivity and quantification for methionine- and tyrosine-directed probes *in situ*. a,**c**,**g**, Structures of the probes that were used for *in situ* treatment of *S. aureus* SH1000 at 100 μM probe concentration. **b**,**d**,**h**, Masses of modification determined through analysis with an Open Search in MSFragger[11, 12]-based FragPipe. The peaks highlighted in red are the masses that have been selected for further analysis within the same row. The expected masses are labelled as Δm_{exp}. The mass that corresponds to reactivity through formation of the isocyanate is labelled as Δm_{NCO}. **e**,**i**, One peak pair (indicated in red in the same row) is selected for a Mass Offset Search[11] that localizes this modification to the modified amino acid(s). In this way, selectivity is assessed across all proteinogenic amino acids. The bar graph represents the fraction of all modified sites that is modified at the indicated amino acid. *C*-term = *C*-terminal modification. *N*-term = *N*-terminal modification. **f**,**j**, Specific amino acid(s) are selected for quantification at the selected masses (indicated in red in the same row) using a Closed Search[11] and the IonQuant[13] feature. The heavy and light samples were mixed at a ratio of 1:1. The grey, solid line indicates the expected values of $\log_2(R) = 0$. The grey, dashed lines indicate the preferred window of quantification ($-1 < \log_2(R) < 1$). All data is based on technical duplicates.

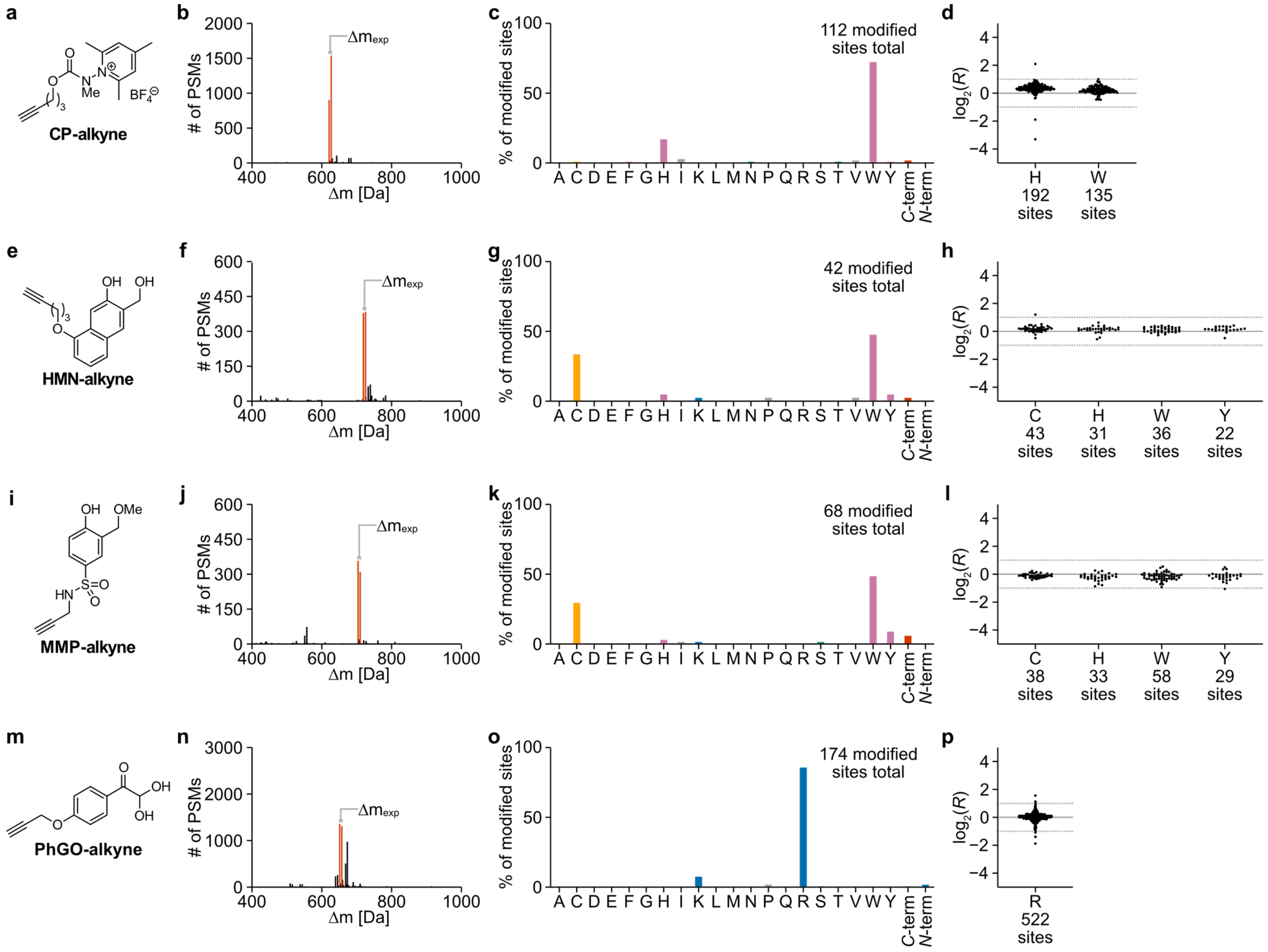

Supplementary Figure 85 | Masses of modification, amino acid selectivity and quantification for tryptophan- and arginine-directed probes *in situ*. **a**,**e**,**i**,**m**, Structures of the probes that were used for *in situ* treatment of *S. aureus* SH1000 at 100 μM (**a**,**e**,**i**) or 1 mM (**m**) probe concentration. For **CP-** and **MMP-alkyne**, the samples were irradiated for 10 min at 280-315 nm after 30 min of treatment with the probe. For **HMN-alkyne**, the samples were irradiated for 10 min at 365 nm after 30 min of treatment with the probe. **b**,**f**,**j**,**n**, Masses of modification determined through analysis with an Open Search in MSFragger[11, 12]-based FragPipe. The peaks highlighted in red are the masses that have been selected for further analysis within the same row. The expected masses are labelled as Δm_{exp}. **c**,**g**,**k**,**o**, One peak pair (indicated in red in the same row) is selected for a Mass Offset Search[11] that localizes this modification to the modified amino acid(s). In this way, selectivity is assessed across all proteinogenic amino acids. The bar graph represents the fraction of all modified sites that is modified at the indicated amino acid. *C*-term = *C*-terminal modification. *N*-term = *N*-terminal modification. **d**,**h**,**l**,**p**, Specific amino acid(s) are selected for quantification at the selected masses (indicated in red in the same row) using a Closed Search[11] and the IonQuant[13] feature. The heavy and light samples were mixed at a ratio of 1:1. The grey, solid line indicates the expected values of $\log_2(R) = 0$. The grey, dashed lines indicate the preferred window of quantification ($-1 < \log_2(R) < 1$). All data is based on technical duplicates.

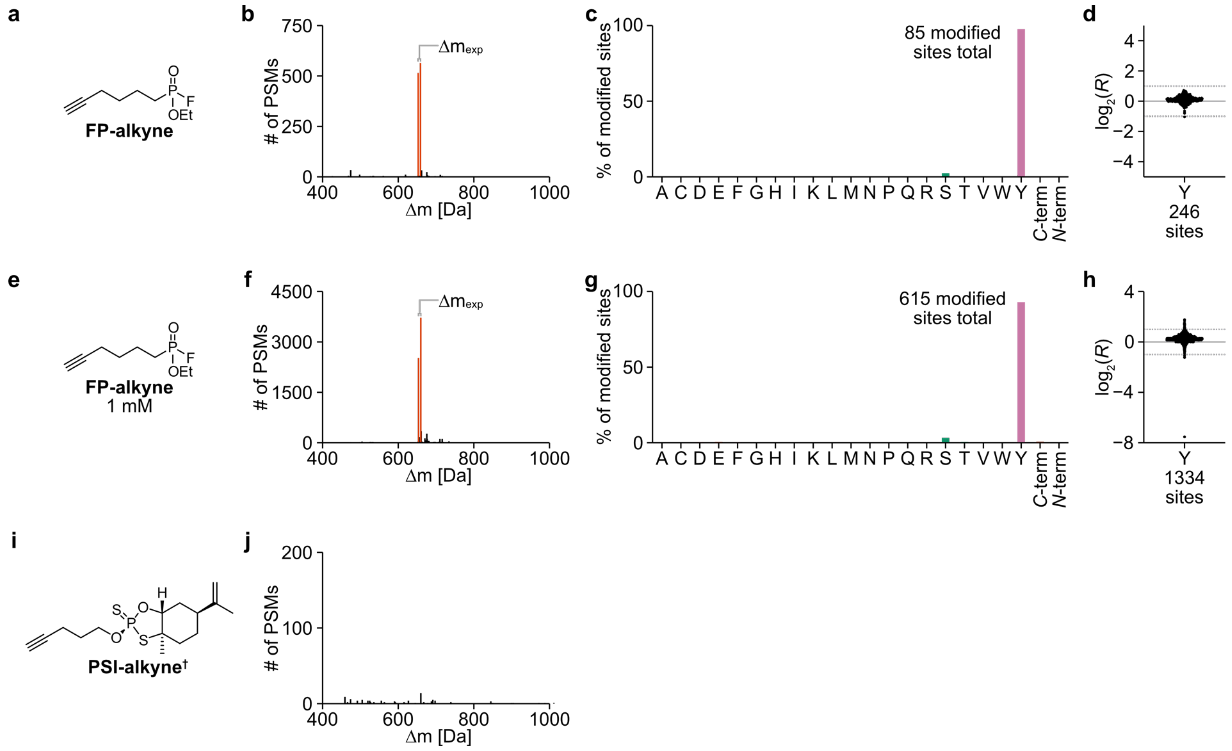

Supplementary Figure 86 | Masses of modification for FP- and PSI-alkyne. a,**e**,**i**, Structure of the probes that were used for treatment of the proteome of *S. aureus* SH1000 at 100 μM (**a**,**i**) or 1 mM (**e**) probe concentration. **b**,**f**,**j**, Masses of modification determined through analysis with an Open Search in MSFragger[11, 12]-based FragPipe. All data is based on technical duplicates. **c**,**g**, One peak pair (indicated in red in the same row) is selected for a Mass Offset Search[11] that localizes this modification to the modified amino acid(s). In this way, selectivity is assessed across all proteinogenic amino acids. The bar graph represents the fraction of all modified sites that is modified at the indicated amino acid. *C*-term = *C*-terminal modification. *N*-term = *N*-terminal modification. **d**,**h**, Specific amino acid(s) are selected for quantification at the selected masses (indicated in red in the same row) using a Closed Search[11] and the IonQuant[13] feature. The heavy and light samples were mixed at a ratio of 1:1. The grey, solid line indicates the expected values of $\log_2(R) = 0$. The grey, dashed lines indicate the preferred window of quantification ($-1 < \log_2(R) < 1$). †: No clear mass of modification was detected and therefore no analysis of the amino acid selectivity was possible.

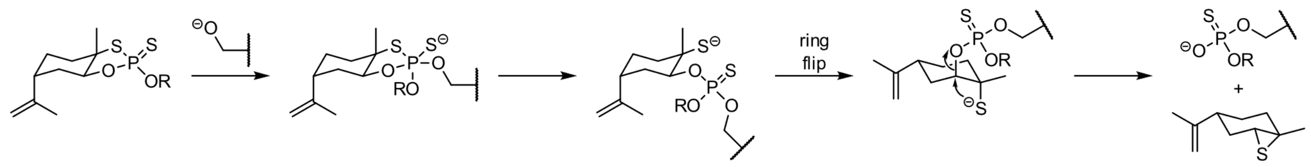

Supplementary Figure 87 | Mechanism of serine-labelling with P(V) reagents (PSI-alkyne) as described by Vantourout *et al.*[40]

Supplementary Tables

Supplementary Table 1 | Overview of the chemical mechanisms of the used electrophiles. A summary of the chemical mechanisms of the used electrophiles. This data can be found as additional data file "Zanon_et_al_SI_Table_1".

Supplementary Table 2 | Overview of electrophile reactivity. A summary of the key information on masses of modification, amino acid selectivity and quantification for all probes. This data can be found as additional data file "Zanon_et_al_SI_Table_2".

Supplementary Table 3 | Mass of modification data for all probes. Masses of modification were determined using an Open Search in MSFragger[11, 12]-based FragPipe. This data can be found as additional data file "Zanon_et_al_SI_Table_3".

Supplementary Table 4 | Amino acid selectivity data for all probes. Amino acid selectivity was determined using a Mass Offset Search in MSFragger[11, 12]-based FragPipe. This data can be found as additional data file "Zanon_et_al_SI_Table_4".

Supplementary Table 5 | Quantification using all probes. Quantification was mainly performed using MSFragger[11, 12] Closed Search and IonQuant[13] labelling based quantification. Individual data sets are also included that were quantified using MSFragger[11, 12] Mass Offset Search and IonQuant[13] labelling based quantification or using MaxQuant[14] or pFind 3.[15] This data can be found as additional data file "Zanon_et_al_SI_Table_5".

Supplementary Table 6 | Data analysis parameters. A summary of the parameters used for data evaluation of all probes.

Probe	Masses of modification	Quantified amino acid(s)*	Comments	Solvent**
**IA-alkyne**	618.3602 Da<br>624.3677 Da	C		DMSO
**IA-alkyne**	137.0841 Da	none	No enrichment	DMSO
**CA-alkyne**	618.3602 Da<br>624.3677 Da	C		DMSO
**CA-nitrile**	619.3554 Da<br>625.3629 Da	none	Masses of modification not detected	DMSO
**BMK-alkyne**	589.3336 Da<br>595.3411 Da	C		DMSO
**PFPSA-alkyne**	760.2738 Da<br>766.2813 Da	C		DMSO
**BrBT-alkyne**	695.2962 Da<br>701.3037 Da	C		DMSO
**MSBT-alkyne**	695.2962 Da<br>701.3037 Da	C		DMSO
**MST-alkyne**	679.3303 Da<br>685.3378 Da	C		DSMO
**MSOD-alkyne**	679.3190 Da<br>685.3265 Da	C		DMSO
**EBX1-alkyne**	587.3180 Da<br>593.3255 Da	C	Masses of modification for alkynylation	DMSO
**EBX1-alkyne**	715.2303 Da<br>721.2378 Da	C	Masses of modification for modification as vinyl iodide	DMSO
**EBX1-alkyne**	835.2514 Da<br>841.2589 Da	C	Masses of modification for modification as vinylbenziodoxolone	DMSO
**EBX2-alkyne**	505.2761 Da<br>511.2836 Da	C		DMSO
**Ep-alkyne**	577.3336 Da<br>583.3411 Da	C		DMSO
**Ts-alkyne**	533.3074 Da<br>539.3149 Da	C; E		DMSO
**MI-alkyne**	733.3871 Da<br>739.3946 Da	C	Masses of modification after hydrolysis of the succinimide	DMSO
**AlkPA-alkyne**	630.3602 Da<br>636.3677 Da	C		DMSO
**ArPA-alkyne**	650.3289 Da<br>656.3364 Da	C		DMSO
**AlkAA-alkyne**	632.3758 Da<br>638.3833 Da	C; H + K + N-term		DMSO
**ArAA-alkyne**	652.3445 Da<br>658.3520 Da	C; H + K + N-term		DMSO
**AlkFAA-alkyne**	650.3664 Da<br>656.3739 Da	C; H + K + N-term	Masses of modification not detected	DMSO
**AlkVS-alkyne**	683.3425 Da<br>689.3500 Da	C; H + K + N-term		DMSO
**ArVS-alkyne**	730.3221 Da<br>736.3296 Da	C; H + K + N-term		DMSO
**AlkVSA-alkyne**	668.3428 Da<br>674.3503 Da	C; H + K + N-term		DMSO
**ArVSA-alkyne**	688.3115 Da<br>694.3190 Da	C; H + K + N-term		DMSO
**STP-alkyne**	561.3023 Da<br>567.3098 Da	K + S + T + N-term		DMSO
**TFP-alkyne**	561.3023 Da<br>567.3098 Da	K + S + T + N-term		DMSO
**NHS-alkyne**	575.3180 Da	K + S + T + N-term		DMSO

	581.3255 Da			
**ATT-alkyne**	575.3180 Da<br>581.3255 Da	K + S + T + N-term		DMSO
**NASA-alkyne**	575.3180 Da<br>581.3255 Da	K + S + T + N-term		DMSO
**AlkSq-alkyne**	628.3081 Da<br>634.3156 Da	K		DMSO
**ArSq-alkyne**	676.3081 Da<br>682.3156 Da	K		DMSO
**EBA-alkyne**	647.3180 Da<br>653.3255 Da	K		DMSO
**oNBA-alkyne**	679.3190 Da<br>685.3265 Da	K	irradiation at 365 nm	DMSO
**TCA-alkyne**	612.3245 Da<br>618.3320 Da	N-term	The additional masses of modification that accommodate for clipping of the *N*-terminal methionine (481.2844 Da, 487.2920 Da) were not included in the Offset or Closed Searches as those are taken into account by enabling “Clip N-term M”.	DMSO
**PCA-alkyne**	594.3027 Da<br>600.3102 Da	N-term	The additional masses of modification that accommodate for clipping of the *N*-terminal methionine (463.2624 Da, 469.2706 Da) were not included in the Offset or Closed Searches as those are taken into account by enabling “Clip N-term M”.	DMSO
**PhTet-alkyne**	756.3820 Da<br>762.3895 Da	C; D + E	irradiation at 280-315 nm	DMSO
**AmTet-alkyne**	723.3565 Da<br>729.3640 Da	C; D + E	irradiation at 280-315 nm	DMSO
**MeTet-alkyne**	694.3663 Da<br>700.3738 Da	(C;) D + E***	irradiation at 280-315 nm	DMSO
**MeTet-alkyne**	638.3289 Da<br>644.3364 Da	C; F + H + W + Y	Masses of modification for arylation	DMSO
**Isx-alkyne**	750.4177 Da<br>756.4252 Da	D + E + K + N-term		DMSO
**Az-alkyne**	652.3445 Da;<br>653.3285 Da<br>658.3520 Da;<br>659.3360 Da	C; D + E	Masses of modification for the expected modification and the formal hydrolysis product leading to loss of NH_3. In most analyses the two pairs of masses of modification were quantified together. In indicated cases the two pairs of masses of modification were analysed separately.	DMSO
**HC-alkyne**	694.3663 Da<br>700.3738 Da	(C;) D + E***		DMF****
**SuFEx-alkyne**	744.3377 Da<br>750.3452 Da	K + Y		DMF****
**SuTEx1-alkyne**	702.2908 Da<br>708.2983 Da	K + Y		DMF****
**SuTEx2-alkyne**	702.2908 Da<br>708.2983 Da	K + Y		DMF****
**PTAD-alkyne**	710.3249 Da<br>716.3323 Da	Y		DMF****
**PTAD-alkyne**	654.3238 Da<br>660.3313 Da	K + N-term	Masses of modification for the reactivity as isocyanate, which is formed through fragmentation of **PTAD-alkyne**	DMF****
**DA1-alkyne**	581.3074 Da<br>587.3149 Da	C; F + H + W + Y	Masses of modification for arylation	DMF****

**DA2-alkyne**	638.3289 Da<br>644.3364 Da	C; F + H + W + Y	Masses of modification for arylation	DMF****
**DA3-alkyne**	611.3180 Da<br>617.3255 Da	C; F + H + W + Y	Masses of modification for arylation	DMF****
**OxMet1-alkyne**	577.3085 Da<br>583.3160 Da	none	Masses of modification not detected	DMF****
**OxMet2-alkyne**	661.3660 Da<br>667.3735 Da	M		DMF*****
**CP-alkyne**	620.3394 Da<br>626.3469 Da	H + W	irradiation at 280-315 nm under argon or open to air as indicated	DMSO
**TPAC-alkyne**	643.2666 Da<br>649.2740 Da	none	Masses of modification not detected	DMF****
**HMN-alkyne**	719.3755 Da<br>725.3830 Da	C; H + W + Y	irradiation at 365 nm	DMSO
**HMP-alkyne**	704.3064 Da<br>710.3139 Da	C; H + W + Y	irradiation at 280-315 nm	DMSO
**MMP-alkyne**	704.3064 Da<br>710.3139 Da	C; H + W + Y	irradiation at 280-315 nm	DMSO
**PhGO-alkyne**	651.3129 Da<br>657.3204 Da	R		DMSO
**PhGO-alkyne**	667.3078 Da<br>673.3153 Da	K + R	Masses of modification for the oxidation product	DMSO
**FP-alkyne**	653.3414 Da<br>659.3489 Da	Y		DMSO
**PSI-alkyne**	657.2822 Da<br>663.2897 Da	none	Masses of modification not detected	DMSO

*: Modification of cysteine was quantified in an individual analysis and modification of all other amino acids was quantified in the same analysis. This is indicated by a ";" after "C" and "+" between all other amino acids. **: Solvent used for making stocks of the probes. Probes were routinely stored as 50 mM or 5 mM stocks at −20 °C until use. ***: Quantification at cysteine was only performed for data produced based on human lysate as only in these cases a significant number of sites was detected at cysteine. ****: Fresh stocks in DMF were prepared and immediately used for the analysis. *****: Stocks were made in DMF and stored at 50 mM concentration at −80 °C until use.

Supplementary Table 7 | Overview of all MS samples included in this study.

Probe	Conc.	Proteome	Samples	File names*	Remarks	Elution**	Filtration ***
**IA-alkyne**	1 mM	*S. aureus* SH1000	181207_P05<br>181207_P06	181228_SMH_181207_P05<br>181228_SMH_181207_P06	1:1 mixture of heavy and light, pre-published data set	0.1% FA	1% FA
**IA-alkyne**	1 mM	*S. aureus* SH1000	181207_P15<br>181207_P16	181228_SMH_181207_P15<br>181228_SMH_181207_P16	4:1 mixture of heavy and light, pre-published data set	0.1% FA	1% FA
**IA-alkyne**	100 µM	*S. aureus* SH1000	200626_P13<br>200626_P14	200706_SMH_200626_P13<br>200706_SMH_200626_P14		0.1% TFA	1% FA
**IA-alkyne**	1 mM	*S. aureus* SH1000	200730_P09<br>200730_P10	200803_SMH_200730_P09<br>200803_SMH_200730_P10		0.1% TFA	1% FA
**IA-alkyne**	100 µM	*S. aureus* SH1000	210511_P25<br>210511_P26	210514_PZ_210511_P25<br>210514_PZ_210511_P26	No enrichment	0.1% TFA	0.1% TFA
**IA-alkyne**	100 µM	*S. aureus* SH1000	210507_P01<br>210507_P02	210507_SMH_210507_P01<br>210507_SMH_210507_P02	*in situ* treatment	0.1% TFA	0.1% TFA
**IA-alkyne**	100 µM	MDA-MB-231	200103_P13<br>200103_P14	200103_SMH_200103_P13<br>200103_SMH_200103_P14		0.1% TFA	1% FA
**CA-alkyne**	100 µM	*S. aureus* SH1000	200913_P11<br>200913_P12	200918_SMH_200913_P11<br>200918_SMH_200913_P12		0.1% TFA	0.1% TFA
**CA-nitrile**	100 µM	*S. aureus* SH1000	200913_P09<br>200913_P10	200918_SMH_200913_P09<br>200918_SMH_200913_P10		0.1% TFA	0.1% TFA
**BMK-alkyne**	100 µM	*S. aureus* SH1000	190504_P19<br>190504_P20	190511_SMH_190504_P19<br>190511_SMH_190504_P20		0.1% FA	1% FA
**PFPSA-alkyne**	100 µM	*S. aureus* SH1000	200626_P07<br>200626_P08	200706_SMH_200626_P07<br>200706_SMH_200626_P08		0.1% TFA	1% FA
**BrBT-alkyne**	100 µM	*S. aureus* SH1000	201105_P11<br>201105_P12	201112_SMH_201105_P11<br>201112_SMH_201105_P12		0.1% TFA	0.1% TFA
**MSBT-alkyne**	100 µM	*S. aureus* SH1000	200626_P23<br>200626_P24	200706_SMH_200626_P23<br>200706_SMH_200626_P24		0.1% TFA	1% FA
**MST-alkyne**	100 µM	*S. aureus* SH1000	200626_P15<br>200626_P16	200706_SMH_200626_P15<br>200706_SMH_200626_P16		0.1% TFA	1% FA
**MSOD-alkyne**	100 µM	*S. aureus* SH1000	200611_P09<br>200611_P10	200609_SMH_200611_P09<br>200609_SMH_200611_P10		0.1% TFA	1% FA
**EBX1-alkyne**	100 µM	*S. aureus* SH1000	190504_P13<br>190504_P14	190511_SMH_190504_P13<br>190511_SMH_190504_P14		0.1% FA	1% FA
**EBX2-alkyne**	100 µM	*S. aureus* SH1000	201105_P23<br>201105_P24	201112_SMH_201105_P23<br>201112_SMH_201105_P24		0.1% TFA	0.1% TFA
**EBX2-alkyne**	100 µM	*S. aureus* SH1000	210507_P03<br>210507_P04	210507_SMH_210507_P03<br>210507_SMH_210507_P04	*in situ* treatment	0.1% TFA	0.1% TFA
**EBX2-alkyne**	100 µM	MDA-MB-231	200611_P19	200701_SMH_200611_P19		0.1% TFA	1% FA

			200611_P20	200701_SMH_200611_P20			
**Ep-alkyne**	100 μM	*S. aureus* SH1000	201022_P01<br>201022_P02	201023_SMH_201022_P01<br>201023_SMH_201022_P02		0.1% TFA	0.1% TFA
**Ep-alkyne**	1 mM	*S. aureus* SH1000	201105_P19<br>201105_P20	201112_SMH_201105_P19<br>201112_SMH_201105_P20		0.1% TFA	0.1% TFA
**Ts-alkyne**	100 μM	*S. aureus* SH1000	190611_P15<br>190611_P16	190613_SMH_190611_P15<br>190613_SMH_190611_P16		0.1% FA	1% FA
**Ts-alkyne**	1 mM	*S. aureus* SH1000	201105_P05<br>201105_P06	201112_SMH_201105_P05<br>201112_SMH_201105_P06		0.1% TFA	0.1% TFA
**MI-alkyne**	100 μM	*S. aureus* SH1000	200718_P17<br>200718_P18	200722_SMH_200718_P17<br>200722_SMH_200718_P18		0.1% TFA	1% FA
**AlkPA-alkyne**	100 μM	*S. aureus* SH1000	201211_P01<br>201211_P02	201212_PZ_201211_P01<br>201212_PZ_201211_P02		0.1% TFA	0.1% TFA
**ArPA-alkyne**	100 μM	*S. aureus* SH1000	201015_P23<br>201015_P24	201016_SMH_201015_P23<br>201016_SMH_201015_P24		0.1% TFA	0.1% TFA
**AlkAA-alkyne**	100 μM	*S. aureus* SH1000	191218_P17<br>191218_P18	191220_SMH_191218_P17<br>191220_SMH_191218_P18		0.1% TFA	1% FA
**ArAA-alkyne**	100 μM	*S. aureus* SH1000	200626_P05<br>200626_P06	200706_SMH_200626_P05<br>200706_SMH_200626_P06		0.1% TFA	1% FA
**AlkFAA-alkyne**	100 μM	*S. aureus* SH1000	200103_P03<br>200103_P04	200103_SMH_200103_P03<br>200103_SMH_200103_P04		0.1% TFA	1% FA
**AlkVS-alkyne**	100 μM	*S. aureus* SH1000	200626_P09<br>200626_P10	200706_SMH_200626_P09<br>200706_SMH_200626_P10		0.1% TFA	1% FA
**ArVS-alkyne**	100 μM	*S. aureus* SH1000	200626_P17<br>200626_P18	200706_SMH_200626_P17<br>200706_SMH_200626_P18		0.1% TFA	1% FA
**AlkVSA-alkyne**	100 μM	*S. aureus* SH1000	201015_P21<br>201015_P22	201016_SMH_201015_P21<br>201016_SMH_201015_P22		0.1% TFA	0.1% TFA
**ArVSA-alkyne**	100 μM	*S. aureus* SH1000	200315_P19<br>200315_P20	200318_SMH_200315_P19<br>200318_SMH_200315_P19		0.1% FA	1% FA
**STP-alkyne**	100 μM	*S. aureus* SH1000	200520_P29<br>200520_P30	200522_SMH_200520_P29<br>200522_SMH_200520_P30		0.1% TFA	1% FA
**STP-alkyne**	1 mM	*S. aureus* SH1000	200913_P27<br>200913_P28	200918_SMH_200913_P27<br>200918_SMH_200913_P28		0.1% TFA	0.1% TFA
**STP-alkyne**	100 μM	*S. aureus* SH1000	210507_P09<br>210507_P10	210507_SMH_210507_P09<br>210507_SMH_210507_P10	*in situ* treatment	0.1% TFA	0.1% TFA
**STP-alkyne**	100 μM	MDA-MB-231	200103_P15<br>200103_P16	200103_SMH_200103_P15<br>200103_SMH_200103_P16		0.1% TFA	1% FA
**TFP-alkyne**	100 μM	*S. aureus* SH1000	201015_P15<br>201015_P16	201016_SMH_201015_P15<br>201016_SMH_201015_P16		0.1% TFA	0.1% TFA
**TFP-alkyne**	1 mM	*S. aureus* SH1000	201015_P17<br>201015_P18	201016_SMH_201015_P17<br>201016_SMH_201015_P18		0.1% TFA	0.1% TFA

**NHS-alkyne**	100 μM	*S. aureus* SH1000	200626_P33<br>200626_P34	200706_SMH_200626_P33<br>200706_SMH_200626_P34		0.1% TFA	1% FA
**ATT-alkyne**	100 μM	*S. aureus* SH1000	200913_P21<br>200913_P22	200918_SMH_200913_P21<br>200918_SMH_200913_P22		0.1% TFA	0.1% TFA
**NASA-alkyne**	100 μM	*S. aureus* SH1000	200520_P31<br>200520_P32	200522_SMH_200520_P31<br>200522_SMH_200520_P32		0.1% TFA	1% FA
**AlkSq-alkyne**	100 μM	*S. aureus* SH1000	201105_P27<br>201105_P28	201112_SMH_201105_P27<br>201112_SMH_201105_P28		0.1% TFA	0.1% TFA
**ArSq-alkyne**	100 μM	*S. aureus* SH1000	201022_TM01<br>201022_TM02	201023_TM_201022_TM1<br>201023_TM_201022_TM2		0.1% TFA	0.1% TFA
**ArSq-alkyne**	100 μM	*S. aureus* SH1000	210507_P13<br>210507_P14	210507_SMH_210507_P13<br>210507_SMH_210507_P14	*in situ* treatment	0.1% TFA	0.1% TFA
**ArSq-alkyne**	100 μM	MDA-MB-231	201105_P35<br>201105_P36	201112_SMH_201105_P35<br>201112_SMH_201105_P36		0.1% TFA	0.1% TFA
**EBA-alkyne**	100 μM	*S. aureus* SH1000	200821_P05<br>200821_P06	200821_SMH_200821_P05<br>200821_SMH_200821_P06		0.1% TFA	1% FA
**EBA-alkyne**	100 μM	*S. aureus* SH1000	210507_P15<br>210507_P16	210507_SMH_210507_P15<br>210507_SMH_210507_P16	*in situ* treatment	0.1% TFA	0.1% TFA
**EBA-alkyne**	100 μM	MDA-MB-231	201211_P07<br>201211_P08	201212_PZ_201211_P07<br>201212_PZ_201211_P08		0.1% TFA	0.1% TFA
**oNBA-alkyne**	100 μM	*S. aureus* SH1000	201105_P01<br>201105_P02	201112_SMH_201105_P01<br>201112_SMH_201105_P02	irradiation at 365 nm for 10 min	0.1% TFA	0.1% TFA
**oNBA-alkyne**	1 mM	*S. aureus* SH1000	201211_P05<br>201211_P06	201212_PZ_201211_P05<br>201212_PZ_201211_P06	irradiation at 365 nm for 10 min	0.1% TFA	0.1% TFA
**oNBA-alkyne**	100 μM	*S. aureus* SH1000	210507_P33<br>210507_P34	210507_SMH_210507_P33<br>210507_SMH_210507_P34	*in situ* treatment; irradiation at 365 nm for 10 min	0.1% TFA	0.1% TFA
**oNBA-alkyne**	100 μM	MDA-MB-231	201211_P03<br>201211_P04	201212_PZ_201211_P03<br>201212_PZ_201211_P04	irradiation at 365 nm for 10 min	0.1% TFA	0.1% TFA
**TCA-alkyne**	100 μM	*S. aureus* SH1000	201105_P29<br>201105_P30	201112_SMH_201105_P29<br>201112_SMH_201105_P30		0.1% TFA	0.1% TFA
**TCA-alkyne**	1 mM	*S. aureus* SH1000	200626_P29<br>200626_P30	200706_SMH_200626_P29<br>200706_SMH_200626_P30		0.1% TFA	1% FA
**PCA-alkyne**	100 μM	*S. aureus* SH1000	200520_P35<br>200520_P36	200522_SMH_200520_P35<br>200522_SMH_200520_P36		0.1% TFA	1% FA
**PCA-alkyne**	1 mM	*S. aureus* SH1000	201105_P09<br>201105_P10	201112_SMH_201105_P09<br>201112_SMH_201105_P10		0.1% TFA	0.1% TFA
**PCA-alkyne**	100 μM	*S. aureus* SH1000	210507_P25<br>210507_P26	210507_SMH_210507_P25<br>210507_SMH_210507_P26	*in situ* treatment	0.1% TFA	0.1% TFA
**PCA-alkyne**	1 mM	MDA-MB-231	200718_P11<br>200718_P12	200722_SMH_200718_P11<br>200722_SMH_200718_P12		0.1% TFA	1% FA

**PhTet-alkyne**	100 µM	*S. aureus* SH1000	190222_P01_1<br>190222_P01_2	190301_KAB_190222_P1-1<br>190301_KAB_190222_P1-2	irradiation at 280-315 nm for 10 min	0.1% FA	1% FA
**AmTet-alkyne**	100 µM	*S. aureus* SH1000	190222_P03_1<br>190222_P03_2	190301_KAB_190222_P3-1<br>190301_KAB_190222_P3-2	irradiation at 280-315 nm for 10 min	0.1% FA	1% FA
**MeTet-alkyne**	100 µM	*S. aureus* SH1000	200305_P17<br>200305_P18	200417_SMH_200305_P17<br>200417_SMH_200305_P18	irradiation at 280-315 nm for 10 min	0.1% FA	1% FA
**MeTet-alkyne**	1 mM	*S. aureus* SH1000	200913_P07<br>200913_P08	200918_SMH_200913_P07<br>200918_SMH_200913_P08	irradiation at 280-315 nm for 10 min	0.1% TFA	0.1% TFA
**MeTet-alkyne**	100 µM	*S. aureus* SH1000	200305_P15<br>200305_P16	200417_SMH_200305_P15<br>200417_SMH_200305_P16	irradiation at 280-315 nm for 5 min	0.1% FA	1% FA
**MeTet-alkyne**	100 µM	*S. aureus* SH1000	200305_P19<br>200305_P20	200417_SMH_200305_P19<br>200417_SMH_200305_P20	irradiation at 280-315 nm for 30 min	0.1% FA	1% FA
**MeTet-alkyne**	100 µM	*S. aureus* SH1000	210507_P27<br>210507_P28	210507_SMH_210507_P27<br>210507_SMH_210507_P28	*in situ* treatment; irradiation at 280-315 nm for 10 min	0.1% TFA	0.1% TFA
**MeTet-alkyne**	100 µM	MDA-MB-231	200103_P19<br>200103_P20	200103_SMH_200103_P19<br>200103_SMH_200103_P20	irradiation at 280-315 nm for 10 min	0.1% TFA	1% FA
**Isx-alkyne**	100 µM	*S. aureus* SH1000	210511_P01<br>210511_P02	210514_PZ_210511_P01<br>210514_PZ_210511_P02		0.1% TFA	0.1% TFA
**Isx-alkyne**	1 mM	*S. aureus* SH1000	210511_P03<br>210511_P04	210514_PZ_210511_P03<br>210514_PZ_210511_P04		0.1% TFA	0.1% TFA
**Az-alkyne**	100 µM	*S. aureus* SH1000	200513_P05<br>200513_P06	200513_SMH_200513_P05<br>200513_SMH_200513_P06		0.1% FA	1% FA
**Az-alkyne**	100 µM	*S. aureus* SH1000	210507_P05<br>210507_P06	210507_SMH_210507_P05<br>210507_SMH_210507_P06	*in situ* treatment	0.1% TFA	0.1% TFA
**Az-alkyne**	100 µM	MDA-MB-231	200718_P13<br>200718_P14	200722_SMH_200718_P13<br>200722_SMH_200718_P14.		0.1% TFA	1% FA
**HC-alkyne**	100 µM	*S. aureus* SH1000	201211_P11<br>201211_P12	201212_PZ_201211_P11<br>201212_PZ_201211_P12		0.1% TFA	0.1% TFA
**HC-alkyne**	1 mM	*S. aureus* SH1000	201211_P13<br>201211_P14	201212_PZ_201211_P13<br>201212_PZ_201211_P14		0.1% TFA	0.1% TFA
**HC-alkyne**	100 µM	*S. aureus* SH1000	210507_P07<br>210507_P08	210507_SMH_210507_P07<br>210507_SMH_210507_P08	*in situ* treatment	0.1% TFA	0.1% TFA
**HC-alkyne**	100 µM	MDA-MB-231	201211_P09<br>201211_P10	201212_PZ_201211_P09<br>201212_PZ_201211_P10		0.1% TFA	0.1% TFA
**SuFEx-alkyne**	100 µM	*S. aureus* SH1000	200520_P41<br>200520_P42	200522_SMH_200520_P41<br>200522_SMH_200520_P42		0.1% TFA	1% FA
**SuTEx1-alkyne**	100 µM	*S. aureus* SH1000	200520_P45<br>200520_P46	200522_SMH_200520_P45<br>200522_SMH_200520_P46		0.1% TFA	1% FA
**SuTEx2-alkyne**	100 µM	*S. aureus* SH1000	200520_P47<br>200520_P48	200522_SMH_200520_P47<br>200522_SMH_200520_P48		0.1% TFA	1% FA
**SuTEx2-alkyne**	100 µM	*S. aureus* SH1000	210507_P21	210507_SMH_210507_P21	*in situ* treatment	0.1% TFA	0.1% TFA

			210507_P22	210507_SMH_210507_P22			
**SuTEx2-alkyne**	100 μM	MDA-MB-231	200611_P17<br>200611_P18	200701_SMH_200611_P17<br>200701_SMH_200611_P18		0.1% TFA	1% FA
**PTAD-alkyne**	100 μM	*S. aureus* SH1000	200305_P07<br>200305_P08	200308_SMH_200305_P7<br>200308_SMH_200305_P8		0.1% FA	1% FA
**PTAD-alkyne**	100 μM	*S. aureus* SH1000	200626_P35<br>200626_P36	200706_SMH_200626_P35<br>200706_SMH_200626_P36	200 mM Tris-HCl pH = 7.4 added to sample	0.1% TFA	1% FA
**PTAD-alkyne**	100 μM	*S. aureus* SH1000	210507_P23<br>210507_P24	210507_SMH_210507_P23<br>210507_SMH_210507_P24	*in situ* treatment	0.1% TFA	0.1% TFA
**PTAD-alkyne**	100 μM	MDA-MB-231	201216_P03<br>201216_P04	201218_PZ_201216_P03<br>201218_PZ_201216_P04		0.1% TFA	0.1% TFA
**DA1-alkyne**	100 μM	*S. aureus* SH1000	200529_P15<br>200529_P16	200530_SMH_200529_P15<br>200530_SMH_200529_P16		0.1% TFA	1% FA
**DA1-alkyne**	100 μM	*S. aureus* SH1000	200718_P19<br>200718_P20	200722_SMH_200718_P19<br>200722_SMH_200718_P20	Zeba Spin purification before CuAAC to isoDTB tags	0.1% TFA	1% FA
**DA2-alkyne**	100 μM	*S. aureus* SH1000	200529_P17<br>200529_P18	200530_SMH_200529_P17<br>200530_SMH_200529_P18		0.1% TFA	1% FA
**DA3-alkyne**	100 μM	*S. aureus* SH1000	201015_P19<br>201015_P20	201016_SMH_201015_P19<br>201016_SMH_201015_P20		0.1% TFA	0.1% TFA
**OxMet1-alkyne**	100 μM	*S. aureus* SH1000	200227_P01<br>200227_P02	200407_SMH_200227_P01<br>200407_SMH_200227_P02		0.1% FA	1% FA
**OxMet2-alkyne**	100 μM	*S. aureus* SH1000	200529_P09<br>200529_P10	200530_SMH_200529_P09<br>200530_SMH_200529_P10		0.1% TFA	1% FA
**OxMet2-alkyne**	100 μM	*S. aureus* SH1000	210507_P17<br>210507_P18	210507_SMH_210507_P17<br>210507_SMH_210507_P18	*in situ* treatment	0.1% TFA	0.1% TFA
**OxMet2-alkyne**	100 μM	MDA-MB-231	200103_P17<br>200103_P18	200103_SMH_200103_P17<br>200103_SMH_200103_P18		0.1% TFA	1% FA
**CP-alkyne**	100 μM	*S. aureus* SH1000	200529_P03<br>200529_P04	200530_SMH_200529_P03<br>200530_SMH_200529_P04	irradiation at 280-315 nm for 10 min open to air	0.1% TFA	1% FA
**CP-alkyne**	100 μM	*S. aureus* SH1000	200529_P01<br>200529_P02	200530_SMH_200529_P01<br>200530_SMH_200529_P02	irradiation at 280-315 nm for 10 min under argon	0.1% TFA	1% FA
**CP-alkyne**	1 mM	*S. aureus* SH1000	200913_P01<br>200913_P02	200918_SMH_200913_P01<br>200918_SMH_200913_P02	irradiation at 280-315 nm for 10 min under argon	0.1% TFA	0.1% TFA
**CP-alkyne**	100 μM	*S. aureus* SH1000	210507_P29<br>210507_P30	210507_SMH_210507_P29<br>210507_SMH_210507_P30	*in situ* treatment; irradiation at 280-315 nm for 10 min open to air	0.1% TFA	0.1% TFA
**CP-alkyne**	100 μM	MDA-MB-231	201015_P05<br>201015_P06	201016_SMH_201015_P05<br>201016_SMH_201015_P06	irradiation at 280-315 nm for 10 min under argon	0.1% TFA	0.1% TFA
**TPAC-alkyne**	100 μM	*S. aureus* SH1000	200529_P13<br>200529_P14	200530_SMH_200529_P13<br>200530_SMH_200529_P14		0.1% TFA	1% FA

**HMN-alkyne**	100 µM	*S. aureus* SH1000	200529_P07<br>200529_P08	200530_SMH_200529_P07<br>200530_SMH_200529_P08	irradiation at 365 nm for 10 min	0.1% TFA	1% FA
**HMN-alkyne**	100 µM	*S. aureus* SH1000	210507_P35<br>210507_P36	210507_SMH_210507_P35<br>210507_SMH_210507_P36	*in situ* treatment; irradiation at 365 nm for 10 min	0.1% TFA	0.1% TFA
**HMN-alkyne**	100 µM	MDA-MB-231	200718_P07<br>200718_P08	200722_SMH_200718_P07<br>200722_SMH_200718_P08	irradiation at 365 nm for 10 min	0.1% TFA	1% FA
**HMP-alkyne**	100 µM	*S. aureus* SH1000	200806_P23<br>200806_P24	200809_SMH_200806_P23<br>200809_SMH_200806_P24	irradiation at 280-315 nm for 10 min	0.1% TFA	1% FA
**MMP-alkyne**	100 µM	*S. aureus* SH1000	200821_P03<br>200821_P04	200821_SMH_200821_P03<br>200821_SMH_200821_P04	irradiation at 280-315 nm for 10 min	0.1% TFA	1% FA
**MMP-alkyne**	100 µM	*S. aureus* SH1000	210507_P31<br>210507_P32	210507_SMH_210507_P31<br>210507_SMH_210507_P32	*in situ* treatment; irradiation at 280-315 nm for 10 min	0.1% TFA	0.1% TFA
**MMP-alkyne**	100 µM	MDA-MB-231	201015_P01<br>201015_P02	201016_SMH_201015_P01<br>201016_SMH_201015_P02	irradiation at 280-315 nm for 10 min	0.1% TFA	0.1% TFA
**PhGO-alkyne**	100 µM	*S. aureus* SH1000	200821_P11<br>200821_P12	200821_SMH_200821_P11<br>200821_SMH_200821_P12		0.1% TFA	1% FA
**PhGO-alkyne**	1 mM	*S. aureus* SH1000	200821_P13<br>200821_P14	200821_SMH_200821_P13<br>200821_SMH_200821_P14		0.1% TFA	1% FA
**PhGO-alkyne**	100 µM	*S. aureus* SH1000	210507_P19<br>210507_P20	210507_SMH_210507_P19<br>210507_SMH_210507_P20	*in situ* treatment	0.1% TFA	0.1% TFA
**PhGO-alkyne**	1 mM	MDA-MB-231	200821_P17<br>200821_P18	200821_SMH_200821_P17<br>200821_SMH_200821_P18		0.1% TFA	1% FA
**FP-alkyne**	100 µM	*S. aureus* SH1000	210610_P01<br>210610_P02	210610_PZ_210610_P01<br>210610_PZ_210610_P02		0.1% TFA	0.1% TFA
**FP-alkyne**	1 mM	*S. aureus* SH1000	210610_P03<br>210610_P04	210610_PZ_210610_P03<br>210610_PZ_210610_P04		0.1% TFA	0.1% TFA
**PSI-alkyne**	100 µM	*S. aureus* SH1000	201015_P27<br>201015_P28	201016_SMH_201015_P27<br>201016_SMH_201015_P28		0.1% TFA	0.1% TFA

*: The file name gives information on the date, when the mass spectrometric measurement was set up, the initials of the scientist to set up the experiment and the sample name in that order. **: Elution was performed with 50% acetonitrile in water with the indicated concentration of the indicated acid. **: Samples were re-dissolved and filtered for mass spectrometric analysis in water with the indicated concentration of the indicated acid.

Supplementary Table 8 | SMILES for all probes used.

Probe	SMILES
**IA-alkyne**	C#CCCCCNC(CI)=O
**CA-alkyne**	C#CCCCCNC(CCl)=O
**CA-nitrile**	O=C(CCl)NCCCCC#N
**BMK-alkyne**	O=C(CBr)CCCC#C
**PFPSA-alkyne**	FC1=C(F)C(F)=C(S(NCCC#C)(=O)=O)C(F)=C1F
**BrBT-alkyne**	BrC1=NC2=CC=C(C(NCC#C)=O)C=C2S1
**MSBT-alkyne**	O=C(NCC#C)C1=CC=C(N=C(S(=O)(C)=O)S2)C2=C1
**MST-alkyne**	C#CCOC(C=C1)=CC=C1N2C(S(=O)(C)=O)=NN=N2
**MSOD-alkyne**	C#CCOC(C=C1)=CC=C1C2=NN=C(S(=O)(C)=O)O2
**EBX1-alkyne**	O=C1C2=CC=CC=C2I(C#CCCOCC#C)O1
**EBX2-alkyne**	O=C1C2=CC(C)=CC=C2I(C#C[Si](C)(C)C)O1
**Ep-alkyne**	C#CCCC1CO1
**Ts-alkyne**	C#CCCOS(C1=CC=C(C)C=C1)(=O)=O
**MI-alkyne**	C#CCCCC(NCCN1C(C=CC1=O)=O)=O
**AlkPA-alkyne**	C#CCCCCNC(C#C)=O
**ArPA-alkyne**	C#CC(C=C1)=CC=C1NC(C#C)=O
**AlkAA-alkyne**	C#CCCCCNC(C=C)=O
**ArAA-alkyne**	C#CC(C=C1)=CC=C1NC(C=C)=O
**AlkFAA-alkyne**	C#CCCCCNC(C(F)=C)=O
**AlkVS-alkyne**	C=CS(CCOCCCC#C)(=O)=O
**ArVS-alkyne**	O=C(NCC#C)C(C=C1)=CC=C1S(=O)(C=C)=O
**AlkVSA-alkyne**	C#CCCCCNS(=O)(C=C)=O
**ArVSA-alkyne**	C#CC(C=C1)=CC=C1NS(C=C)(=O)=O
**STP-alkyne**	C#CCCC(OC1=C(F)C(F)=C(S(=O)([O-])=O)C(F)=C1F)=O.[Na+]
**TFP-alkyne**	C#CCCC(OC1=C(F)C(F)=CC(F)=C1F)=O
**NHS-alkyne**	C#CCCCC(ON1C(CCC1=O)=O)=O
**ATT-alkyne**	S=C1SCCN1C(CCCC#C)=O
**NASA-alkyne**	O=C(N(CC#N)S(=O)(C1=CC=CC=C1)=O)CCCC#C
**AlkSq-alkyne**	O=C1C(NCCC#C)=C(OC)C1=O
**ArSq-alkyne**	O=C1C(NC2=CC=C(C#C)C=C2)=C(OC)C1=O
**EBA-alkyne**	C#CC1=CC=C(OCC#C)C=C1C([H])=O
***o*NBA-alkyne**	OCC1=CC=C(C(NCC#C)=O)C=C1[N+]([O-])=O
**TCA-alkyne**	C#CCCN1N=NC(C([H])=O)=C1
**PCA-alkyne**	C#CC1=CN=C(C([H])=O)C=C1
**PhTet-alkyne**	O=C(C(C=C1)=CC=C1N2N=NC(C3=CC=CC=C3)=N2)NCC#C
**AmTet-alkyne**	O=C(C(C=C1)=CC=C1N2N=NC(C(N)=O)=N2)NCC#C
**MeTet-alkyne**	CC1=NN(C2=CC=C(C=C2)C(NCC#C)=O)N=N1
**Isx-alkyne**	O=S(C(F)(F)F)([O])=O.CC[N+](O1)=CC=C1C2=CC(OCC#C)=CC=C2
**Az-alkyne**	C#CCOC1=CC=C(C2=NC2)C=C1
**HC-alkyne**	C/C(Cl)=N/NC1=CC=C(C=C1)C(NCC#C)=O
**SuFEx-alkyne**	O=C(NCCCCC#C)C1=CC=C(S(=O)(F)=O)C=C1
**SuTEx1-alkyne**	O=C(NCC#C)C1=CC=C(S(=O)(N2N=CN=C2)=O)C=C1
**SuTEx2-alkyne**	O=C(NCC#C)C1=CC=C(S(N2N=C(C3=CC=C(OC)C=C3)N=C2)(=O)=O)C=C1
**PTAD-alkyne**	O=C(N=NC1=O)N1C2=CC=C(OCC#C)C=C2
**DA1-alkyne**	C#CC1=CC=C([N+]#N)C=C1.F[B-](F)(F)F.
**DA2-alkyne**	O=C(NCC#C)C1=CC=C([N+]#N)C=C1.F[B-](F)(F)F
**DA3-alkyne**	C#CCOC1=CC=C([N+]#N)C=C1.F[B-](F)(F)F
**OxMet1-alkyne**	O=C(NCC#C)N(O1)C1C2=CC=CC=C2
**OxMet2-alkyne**	O=C(N1CCC(OCC#C)CC1)N(O2)C2C3=CC=CC=C3
**CP-alkyne**	CC1=CC(C)=[N+](N(C(OCCCC#C)=O)C)C(C)=C1.F[B-](F)(F)F
**TPAC-alkyne**	S=P(Cl)(Cl)OCCCC#C
**HMN-alkyne**	C#CCCCOC1=C(C=C2O)C(C=C2CO)=CC=C1
**HMP-alkyne**	OCC1=C(O)C=CC(S(NCC#C)(=O)=O)=C1
**MMP-alkyne**	OC1=C(COC)C=C(S(NCC#C)(=O)=O)C=C1
**PhGO-alkyne**	O=C(C(O)O)C1=CC=C(OCC#C)C=C1
**FP-alkyne**	C#CCCCCP(F)(OCC)=O
**PSI-alkyne**	S=[P@@]1(OCCCC#C)S[C@]2(C)CC[C@H](C(C)=C)C[C@@]2([H])O1

Supplementary Table 9 | Mass Offset and Closed Search results for all probes in the lysate of *S. aureus* SH1000. Values show selectivities for the main modification of the indicated probes for the indicated amino acids in percent rounded to whole numbers. Zeroes were omitted for clarity. Selectivities ≥50% are indicated in red, selectivities <10% are indicated in grey. Quantified sites are only given for amino acids that were labelled with high selectivity. *: Labelling was performed using UV-activation at 280-315 nm (**MeTet-**, **CP-** and **MMP-alkyne**) or 365 nm (***o*NBA-** and **HMN-alkyne**) for 10 min. ‡: Data for the indicated probe at 1 mM is shown. #: Labelling was performed in degassed lysate under argon. All data is based on technical duplicates.

	A	C	D + E	F	G	H	I	K	L	M	N + Q	P	R	S + T	V	W	Y	C-term	*N-term*	Quantified sites
**IA-**		95				1				2										1197 C
**IA-‡**		87	1			3				6									1	1316 C
**CA-**		96	1							1					1				1	92 C
**BMK-**		89	1		3	2								1			1	1		976 C
**PFPSA-**		89				2		3									2			631 C
**BrBT-**		83	1	2		2	2	1								2	5	2	2	362 C
**MSBT-**		92	1		1		1	1	1				1				1			963 C
**MST-**	1	87	1			1	1	1			1	1		1	1		1			950 C
**MSOD-**		79	1			5		3			1			1			9			1061 C
**EBX1-**	1	91	1		1		1	2	1		1			1						1231 C
**EBX2-**	1	85	1		1	7		2		1	1	1		1						1251 C
**Ep-**		85						5							5				5	41 C
**Ep-‡**		82	5		2	6	2			1					1			1	1	284 C
**Ts-**		85	1			5														68 C
**Ts-‡**		63	33			3													1	386 C
**MI-**		95			1				1	1					1					707 C
**ArPA-**	1	93	1		1	1		1	1						1					283 C
**AlkPA-**		95					1			1	1		1						1	752 C
**AlkAA-**		97	1							1							1		1	294 C
**ArAA-**		95		1				1											2	501 C
**AlkVSA-**		93						2											2	811 C
**ArVSA-**		57			1	9		24											5	968 C
**AlkVS-**		78	1			4		9										1	6	446 C
**ArVS-**		57	1			9		24										1	5	945 C
**PhTet-**		13	79				1	1			2							2		649 D + 1350 E
**AmTet-**		10	80			5												2		712 D + 1691 E
**MeTet-**			94					1										3		416 D + 1776 E
**MeTet-‡**			93					1										4		753 D + 2568 E
**Isx-**	2	1	20		1	5	1	19	1		11		2		1		1		37	
**Isx-‡**	1		25		1	6	1	21	1	1	3			1	1			1	36	
**Az-**		18	75		1					1	1							1		569 D + 870 E
**HC-**		3	91					1						1				3		551 D + 1899 E
**HC-‡**		1	92					1										4		744 D + 2584 E
**STP-**	1				1			79						1	1			1	5	3277 K
**STP-‡**	1		1		1			79	1					9	1			1	5	4065 K
**TFP-**	1				1		1	82	1		1			7				2	5	2145 K
**TFP-‡**	1		1		1			82	1					7	1			1	5	4643 K
**NHS-**	1		1		1		1	78	1		1			5	1			2	7	4404 K
**ATT-**	1		1	1	1		1	83	1		1			3	1			1	4	3380 K
**NASA-**	1		1				1	78	1		1			8	1		1	1	5	2488 K
**AlkSq-**	1						1	9	1					2	1			1	3	1339 K
**ArSq-**	1		1					93										1	2	2990 K
**EBA-**	1		2		1		1	81	1		1		1	1	1			2	5	3796 K
**oNBA-**		1						94										1	2	1456 K
**oNBA-‡**								93					1	1				2	2	1954 K
**OxMet2-**	1	1		1	1	4	1	1	1	73	3	2		3			1	1	6	1838 M
**PhGO-**					3			3					91						3	409 R
**PhGO-‡ alkyne**	1		1		1			1					91	1	1			1	1	1544 R

Supplementary Table 9 (continued)

	A	C	D + E	F	G	H	I	K	L	M	N + Q	P	R	S + T	V	W	Y	*C*-term	*N*-term	Quantified sites
**CP-**		2	1			38	1				7					47		1		1439 H + 454 W
**CP-#**		2				35	1		1		2				1	55	1	1		797 H + 467 W
**CP-*,‡,#**		1				43		1			2				1	49	1	1		1105 H + 538 W
**HMN-**	1	14	2	1		13		2	1		1		1		1	53	9	1	2	513 W
**HMP-**		29				5	2	1							1	50	7	2	2	206 W
**MMP-**		23				11										47	10	2	2	281 W
**DA1-**		41	1	13	1	20								1		5	16	1		
**DA2-**		49		10		13		1						1		5	19			
**DA3-**		21		19		19								1		8	30	1		
**SuFEx-**					1			41						1			55	1		2005 Y
**SuTEx1-**			1					35									62	1		2577 Y
**SuTEx2-**								26						1			71	1		2653 Y
**PTAD-**					1	1		1									95			1116 Y
**FP-**														2			98			246 Y
**FP-‡**			1											4			93		1	1334 Y
**TCA-**																			100	43 *N*-term
**TCA-‡**		6							11						6				78	127 *N*-term
**PCA-**																			100	120 *N*-term
**PCA-‡ alkyne**	2	1	1						1			1		1					93	167 *N*-term

Supplementary Table 10 | A set of 16 electrophilic probes that enables studying nine different amino acids in the lysate of the human cancer cell line MDA-MB-231. Values show selectivities for the main modification of the indicated probes for the indicated amino acids in percent rounded to whole numbers. Zeroes were omitted for clarity. Selectivities ≥50% are indicated in red, selectivities <10% are indicated in grey. Quantified sites are only given for amino acids that were labelled with high selectivity. *: Labelling was performed using UV-activation at 280-315 nm (**MeTet-**, **CP-** and **MMP-alkyne**) or 365 nm (***o*NBA-** and **HMN-alkyne**) for 10 min. ‡: Data for the indicated probe at 1 mM is shown. #: Labelling was performed in degassed lysate under argon. All data is based on technical duplicates.

	A	C	D + E	F	G	H	I	K	L	M	N + Q	P	R	S + T	V	W	Y	*C*-term	*N*-term	Quantified sites
**IA-**		97								1										4124 C
**EBX2-**		93	1		1			1			1			1						3270 C
**MeTet-***		12	83											1				1		223 D + 1098 E
**Az-**		12	85															1		352 D + 667 E
**HC-**		8	87					1										2		233 D + 940 E
**STP**	1				1		1	88	1					5	1				1	5058 K
**ArSq-**		1			1			94			1								1	3039 K
**EBA-**	1	1	2	1	1	1	1	84	3				1	2	1				1	2776 K
o*NBA-								97						1						1258 K
**OxMet2-**	2	7	3				1		1	72	3	3		3	1		1	1	4	1395 M
**PhGO-‡**			1		1			1				1	91	3	1					1879 R
**CP-*,#**		1	1			21					1					73		1		597 H + 848 W
**HMN-***		19				3	1	1	1		1		1			67	2	1		513 W
**MMP-***		39				1		1						1		55	1	1		469 W
**SuTEx2-**								21						1			76	1		2235 Y
**PTAD-alkyne**		2			1	1								1			92	1		1116 Y

Supplementary Table 11 | A set of 15 electrophilic probes that enables studying eight different amino acids and the protein *N*-terminus *in situ*. Values show selectivities for the main modification of the indicated probes for the indicated amino acids in percent rounded to whole numbers. Zeroes were omitted for clarity. Selectivities ≥50% are indicated in red, selectivities <10% are indicated in grey. Quantified sites are only given for amino acids that were labelled with high selectivity. *: Labelling was performed using UV-activation at 280-315 nm (**MeTet-**, **CP-** and **MMP-alkyne**) or 365 nm (**HMN-alkyne**) for 10 min. ‡: Data for the indicated probe at 1 mM is shown. All data is based on technical duplicates.

	A	C	D + E	F	G	H	I	K	L	M	N + Q	P	R	S + T	V	W	Y	C-term	*N*-term	Quantified sites
**IA-**		97				1													1	446 C
**EBX2-**		94	1			1	1	2									1	1	1	418 C
**MeTet-***			94															3		251 D + 1220 E
**Az-**		9	84								1				1			2		701 D + 1407 E
**HC-**			93				1				1	1			1			3		183 D + 807 E
**STP**	1				1			77	1		1			10	1			2	6	648 K
**ArSq-**								93			1							1	2	2190 K
**EBA-**	1		1		1	1	1	76	2		1		1	1	1			2	10	2141 K
**PhGO-‡**	1				1			7	1			2	86	1	1			1	2	522 R
**CP-***		1		1		17	3				1			1	2	72	1	2		192 H + 135 W
**HMN-***		33				5		2				2			2	48	5	2		36 W
**MMP-***		29				3	1	1						1		49	9	6		58 W
**SuTEx2-**								21									76	1		1181 Y
**PTAD-**						3	1										94	1		1130 Y
**PCA-‡ alkyne**								5											95	68 *N*-term

Experimental Procedures

General remarks to synthetic procedures

The syntheses of **TFP-**,[41] **MeTet-**, **PhTet-**, **AmTet-**[42] and **OxMet1-alkyne**[35] have been described previously. **EBX1-alkyne[18]** was a kind gift from the group of Jérôme Waser, Institute of Chemical Sciences and Engineering, École Polytechnique Fédérale de Lausanne. **PSI-alkyne[40]** was a kind gift from the group of Phil Baran, Department of Chemistry, Scripps Research, La Jolla. **SuTEx1-** (HHS-475) and **SuTEx2-alkyne** (HHS-482)**[43]** were kind gifts from Ku-Lung Hsu, Department of Chemistry, University of Virginia. Hex-5-yn-1-yl tosylate[44] and 6-iodohex-1-yne[45] were kind gifts from Stephan A. Sieber, Department of Chemistry, Technical University of Munich. **MI-** (Lumiprobe), **NHS-** (Lumiprobe), **PCA-** (BLDpharm), **STP-** (Lumiprobe) and **Ts-alkyne** (Alfa Aesar) were purchased from chemical vendors. Hex-5-yn-1-amine (**1**) was either obtained commercially (Sigma-Aldrich) or synthesized from the respective phthalimide as described below.

All reactions that are sensitive to air and moisture were carried out in flame-dried glassware under an argon atmosphere using standard Schlenk techniques. Commercially available chemicals were used without further purification unless otherwise mentioned. Unless indicated otherwise, reactions were stirred at room temperature.

Due to potential explosion hazards, azides and diazonium salts should be handled and synthesised with caution. They were handled and transferred with plastic or glass spatulas and pipettes; metal was avoided and their synthesis was carried out in the dark and behind a blast shield. Aqueous layers possibly containing residual NaN_3 or IN_3 were not extracted with halogenated solvents and their pH was adjusted to 8-10 with saturated aqueous $NaHCO_3$ before disposal. With these precautions taken, no safety issues were encountered during the performance of this project, yet caution is advised.

Mg turnings were activated with aqueous HCl (0.1 M), washed with H_2O, acetone and Et_2O and stored under argon atmosphere.

Solvent mixtures are given in v/v. For reactants with given concentrations or mass fractions, the given mass corresponds to the mass of the pure reactant.

Technical solvents for column chromatography were used after distillation. Flash column chromatography was performed on silica gel (VWR, 40-63 µm) with the indicated eluent mixture. Thin Layer Chromatography (TLC) was performed on silica-coated aluminium sheets (Merck, TLC Silicagel 60 F254) with detection by staining with a $KMnO_4$ (1.50 g $KMnO_4$, 10.0 g K_2CO_3 and 1.25 mL 10 wt% aqueous NaOH in 200 mL H_2O), a phosphomolybdic acid (PMA; 10.0 g $H_3P(Mo_3O_{10})_4$ in 100 mL EtOH) or a cerium phosphomolybdate (CPM; 5.56 g $H_3P(Mo_3O_{10})_4$, 2.22 g $Ce(SO_4)_2 \cdot 4H_2O$, 7.1 mL concentrated H_2SO_4 in 200 mL H_2O) solution followed by heat treatment or staining with a 2,4-dinitrophenylhydrazine (DNP; 6.00 g DNP in 30 mL concentrated H_2SO_4, 40 mL H_2O and 100 mL EtOH) without heat treatment.

Preparative High Performance Liquid Chromatography (HPLC) was performed on an XBridge Prep C18 column (30 × 150 mm, 5 µm; Waters) using a gradient of MeCN in H_2O for elution and a system of FlexInject (Waters), 2545 Quaternary Gradient Module (Waters), 2998 Photodiode Array Detector (Waters) and Fraction Collector III (Waters). The eluent was removed by lyophilisation.

NMR-spectra were recorded on an AVHD-300, AVHD-400 or AVHD-500 (Bruker). Multiplets are described using following abbreviations: s - singlet, d - duplet, t - triplet, q - quartet, p - pentet, hept. - heptet, m - multiplet. ^{1}H-NMR spectra were calibrated to the residual solvent signal (δ = 1.94 ppm (p) for CD_3CN; δ = 2.50 ppm (p) for DMSO-d_6; δ = 3.31 ppm (p) for CD_3OD; δ = 5.32 ppm (t) for CD_2Cl_2; δ = 7.26 ppm (s) for $CDCl_3$). ^{13}C-NMR spectra were calibrated to the respective ^{13}C-D multiplets (δ = 1.32 ppm (hept.) for CD_3CN; δ = 39.52 ppm (hept.) for DMSO-d_6; δ = 49.00 ppm (hept.) for CD_3OD; δ = 54.00 ppm (p) for CD_2Cl_2; δ = 77.16 ppm (t) for $CDCl_3$). ^{13}C- and ^{19}F-NMR experiments were run with ^{1}H-decoupling. Apparent multiplets resulting from coincidental equality of coupling constants to those of magnetically non-equivalent protons are denoted accordingly. If signals of inseparable isomers are clearly distinguishable, their integrals are normalized for the individual isomers and the ratio of the isomers is given.

HRMS spectra were recorded in ESI or APCI mode with either an LTQ-FT Ultra (Thermo Fisher Scientific) or an LTQ Orbitrap XL (Thermo Fisher Scientific) coupled to an UltiMate 3000 HPLC system (Thermo Fisher Scientific).

Synthetic procedures

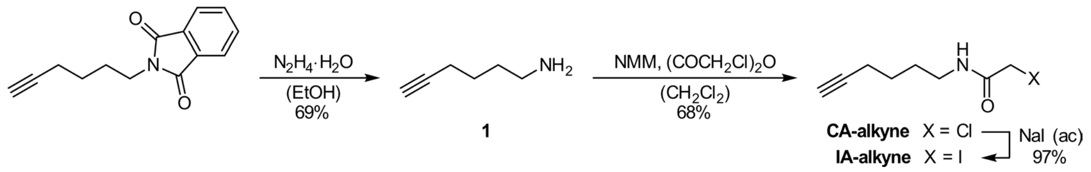

Hex-5-yn-1-amine (1): Synthesis of **1** was carried out similar to literature procedure.[46] A mixture of *N*-(5-Hexynyl)phthalimide (5.00 g, 22.0 mmol, 1.00 equiv.) and $N_2H_4{\cdot}H_2O$ (4.27 mL, 4.41 g, 88.0 mmol, 4.00 equiv.)* in EtOH (90 mL) was heated to reflux for 1 h, during which the reaction mixture solidified. After cooling to room temperature, H_2O (400 mL) was added, the pH was adjusted to 11 with aqueous NaOH (2 M) and the mixture was extracted with CH_2Cl_2 (6 × 200 mL). The combined organic layers were washed with brine, dried over Na_2SO_4, filtered and concentrated *in vacuo*. Vacuum distillation (46 °C, 86 mbar) gave **1** (1.48 g, 15.2 mmol, 69%) as a colourless oil. ^{1}H-NMR (300 MHz, $CDCl_3$, 300 K): δ [ppm] = 1.19 (br. s, 2H), 1.48-1.62 (m, 4H), 1.93 (t, J = 2.7 Hz, 1H), 2.15-2.24 (m, 2H), 2.66-2.74 (m, 2H). The analytical data is in accordance with literature.[47]

*CAUTION: $N_2H_4{\cdot}H_2O$ is highly toxic.

2-Chloro-*N*-(hex-5-yn-1-yl)acetamide (CA-alkyne): Synthesis of **CA-alkyne** was carried out similar to literature procedure.[10, 48] *N*-Methylmorpholine (340 µL, 312 mg, 3.09 mmol, 3.00 equiv.) was added to a solution of **1** (100 mg, 1.03 mmol, 1.00 equiv.) in dry CH_2Cl_2 (5 mL) at 0 °C. Chloroacetic anhydride (352 mg, 2.06 mmol, 2.00 equiv.) was added in portions over 10 min and the reaction mixture was stirred for 21 h, during which it warmed to room temperature. After addition of Et_2O (80 mL), the mixture was washed with aqueous HCl (1 M, 30 mL), aqueous NaOH (1 M, 30 mL) and brine. The organic layer was dried over Na_2SO_4, filtered and concentrated *in vacuo*. Column chromatography (pentane:EtOAc 3:1) gave **CA-alkyne** as a yellow oil (122 mg, 702 µmol, 68%).* TLC (pentane:EtOAc 1:1): R_f = 0.38 [$KMnO_4$]. ^{1}H-NMR (500 MHz, $CDCl_3$, 300 K): δ [ppm] = 1.53-1.61 (m, 2H), 1.65-1.73 (m, 2H), 1.97 (t, J = 2.6Hz, 1H), 2.20-2.26 (m, 2H), 3.34 (apparent q, $J \approx$ 6.8 Hz, 2H), 4.05 (s, 2H), 6.61 (br. s, 1H). ^{13}C-NMR (75.5 MHz, $CDCl_3$, 300 K): δ [ppm] = 18.2 (s, 1C), 25.7 (s, 1C), 28.5 (s, 1C), 39.5 (s, 1C), 42.8 (s, 1C), 69.0 (s, 1C), 83.9 (s, 1C), 166.0 (s, 1C). HRMS-APCI (m/z): calc. ($C_8H_{13}ClNO$ [M+H]$^+$): 174.0680; found: 174.0680. The analytical data is in accordance with literature.[10, 48]

*purification was carried out for use in proteomics experiments, crude product is sufficiently pure for synthesis of **IA-alkyne** as described previously.[10, 48]

2-Iodo-*N*-(hex-5-yn-1-yl)acetamide (IA-alkyne): Synthesis of **IA-alkyne** was carried out similar to literature procedure.[10, 48] A mixture of **CA-alkyne** (75.0 mg, 432 µmol, 1.00 equiv.) and NaI (194 mg, 1.30 mmol, 3.00 equiv.) in dry acetone (2.1 mL) was stirred for 46 h in the dark before concentration *in vacuo*. Purification by column chromatography (pentane:EtOAc 2.5:1) gave **IA-alkyne** (111 mg, 420 µmol, 97%) as a pale yellow solid. TLC (pentane:EtOAc 1:1): R_f = 0.35 [$KMnO_4$]. ^{1}H-NMR (300 MHz, $CDCl_3$, 300 K): δ [ppm] = 1.49-1.73 (m, 4H), 1.96 (t, J = 2.6Hz, 1H), 2.24 (td, J = 6.7, 2.6 Hz, 2H), 3.31-3.37 (apparent q, $J \approx$ 6.7 Hz, 2H), 3.69 (s, 2H), 6.31 (br. s, 1H). ^{13}C-NMR (75.5 MHz, $CDCl_3$, 300 K): δ [ppm] = −0.2 (s, 1C), 18.2 (s, 1C), 25.7 (s, 1C), 28.4 (s, 1C), 40.0 (s, 1C), 69.0 (s, 1C), 84.0 (s, 1C), 167.1 (s, 1C). HRMS-ESI (m/z): calc. ($C_8H_{13}INO$ [M+H]$^+$): 266.0036; found: 266.0037. The analytical data is in accordance with literature.[10, 48]

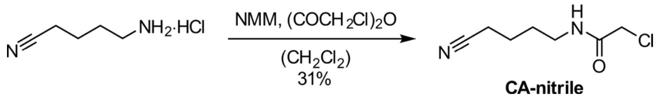

2-Chloro-*N*-(4-cyanobutyl)acetamide (CA-nitrile): *N*-Methylmorpholine (453 µL, 416 mg, 4.12 mmol, 4.00 equiv.) was added to a solution of 5-aminopentanenitrile hydrochloride (139 mg, 1.03 mmol, 1.00 equiv.) in dry CH_2Cl_2 (5 mL) at 0 °C. Chloroacetic anhydride (352 mg, 2.06 mmol, 2.00 equiv.) was added in portions over 10 min and the reaction mixture was stirred for 21 h, during which it warmed to room temperature. After addition of Et_2O (80 mL), the mixture was washed with aqueous HCl (1 M, 30 mL), aqueous NaOH (1 M, 30 mL) and brine. The organic layer was dried over Na_2SO_4, filtered and concentrated *in vacuo*. Purification by column chromatography (pentane:EtOAc 1:1 → 1:2) gave **CA-nitrile** as a yellow oil (55.7 mg, 319 µmol, 31%). TLC (pentane:EtOAc 1:3): R_f = 0.28 [$KMnO_4$]. ^{1}H-NMR (300 MHz, $CDCl_3$, 300 K): δ [ppm] = 1.65-1.78 (m, 4H), 2.36-2.46 (m, 2H), 3.31-3.42 (m, 2H), 4.05 (s, 2H), 6.65 (br. s, 1H). ^{13}C-NMR (75.5 MHz, $CDCl_3$, 300 K): δ [ppm] = 17.0 (s, 1C), 22.8 (s, 1C), 28.7 (s,

1C), 38.8 (s, 1C), 42.7 (s, 1C), 119.4 (s, 1C), 166.2 (s, 1C). HRMS-APCI (m/z): calc. ($C_7H_{12}ClN_2O$ $[M+H]^+$): 175.0633; found: 175.0633.

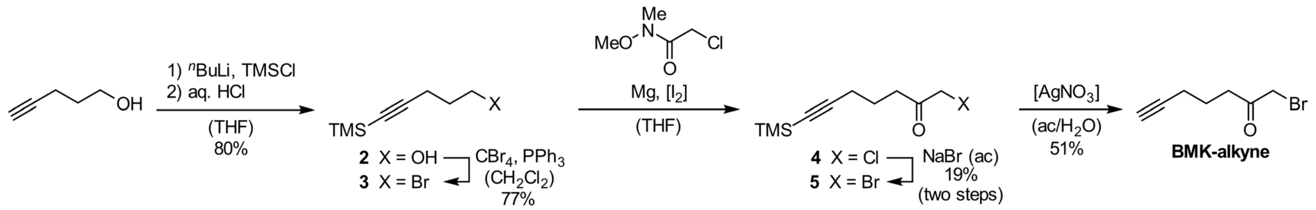

5-(Trimethylsilyl)pent-4-yn-1-ol (2): Synthesis of **2** was carried out similar to literature procedure.[49] To a solution of 4-pentyn-1-ol (505 mg, 6.00 mmol, 1.00 equiv.) in dry THF (15 mL), ^{n}BuLi (2.5 M in hexane, 5.28 mL, 846 mg, 13.2 mmol, 2.20 equiv.) was added dropwise at −78 °C. After stirring for 1 h, TMSCl (2.28 mL, 1.96 g, 18.0 mmol, 3.00 equiv.) was added and the reaction mixture was allowed to warm to room temperature overnight. Aqueous HCl (2 M, 18.0 mL, 1.31 g, 36.0 mmol, 6.00 equiv.) was added at 0 °C and stirring was continued at room temperature for 30 min. Et_2O (12 mL) was added, layers were separated and the aqueous layer was extracted with Et_2O (2 × 12 mL). The combined organic layers were washed with brine (12 mL), saturated aqueous $NaHCO_3$ (12 mL) and brine (12 mL), dried over Na_2SO_4, filtered and concentrated *in vacuo*. Column chromatography (pentane:EtOAc 7:3) gave **2** (751 mg, 4.80 mmol, 80%) as a colourless oil. TLC (pentane:EtOAc 1:1): R_f = 0.54 [$KMnO_4$]. ^{1}H-NMR (400 MHz, $CDCl_3$, 298 K): δ [ppm] = 0.14 (s, 9H), 1.72-1.82 (apparent p, $J \approx 6.5$ Hz, 2H), 2.35 (t, $J = 6.9$ Hz, 2H), 3.76 (t, $J = 6.1$ Hz, 2H). ^{13}C-NMR (101 MHz, $CDCl_3$, 300 K): δ [ppm] = 0.2 (s, 3C), 16.7 (s, 1C), 31.3 (s, 1C), 62.1 (s, 1C), 85.5 (s, 1C), 106.8 (s, 1C). The analytical data is in accordance with literature.[49]

(5-Bromopent-1-yn-1-yl)trimethylsilane (3): Synthesis of **3** was carried out similar to literature procedure.[50] CBr_4 (2.39 g, 7.21 mmol, 1.50 equiv.) was added to a solution of **2** (751 mg, 4.80 mmol, 1.00 equiv.) in dry CH_2Cl_2 (7 mL). At 0 °C, PPh_3 (1.89 g, 7.21 mmol, 1.50 equiv.) was added in small portions and the reaction mixture was stirred for 2 h at room temperature. After concentration *in vacuo*, cold pentane (20 mL) was added, the mixture was filtered and the filtrate was concentrated *in vacuo*. Column chromatography (pentane) gave **3** (813 mg, 3.71 mmol, 77%) as a colourless oil. TLC (pentane): R_f = 0.22 [$KMnO_4$]. ^{1}H-NMR (400 MHz, $CDCl_3$, 298 K): δ [ppm] = 0.15 (s, 9H), 2.00-2.09 (apparent p, $J \approx 6.6$ Hz, 2H), 2.41 (t, $J = 6.8$ Hz, 2H), 3.51 (t, $J = 6.5$ Hz, 2H). ^{13}C-NMR (101 MHz, $CDCl_3$, 300 K): δ [ppm] = 0.2 (s, 3C), 18.8 (s, 1C), 31.6 (s, 1C), 32.4 (s, 1C), 85.9 (s, 1C), 105.2 (s, 1C). The analytical data is in accordance with literature.[49]

1-Chloro-7-(trimethylsilyl)hept-6-yn-2-one (4): A few drops of a solution of **3** (1.70 g, 7.76 mmol, 1.30 equiv.) in dry THF (5 mL) were added to a suspension of activated magnesium turnings (232 mg, 9.55 mmol, 1.60 equiv.) and iodine (15.1 mg, 59.7 µmol, 1 mol%) in dry THF (5 mL) and the mixture was heated to reflux until an exothermic reaction was observed. Subsequently, the rest of the solution was added dropwise and the mixture was stirred for 30 min at 65 °C. After cooling to room temperature, the mixture was added slowly to a solution of 2-chloro-*N*-methoxy-*N*-methylacetamide (821 mg, 5.97 mmol, 1.00 equiv.) in dry THF (20 mL) at 0 °C. Stirring was continued at room temperature for 2 h before quenching by addition of saturated aqueous NH_4Cl (30 mL) at 0 °C. The aqueous layer was extracted with Et_2O (3 × 30 mL), the combined organic layers were dried over Na_2SO_4, filtered and concentrated *in vacuo*. Column chromatography (pentane:EtOAc 9:1) gave a 84:16 mixture of **4** (345 mg, 1.59 mmol, 27%) and **5** (79.2 mg, 303 µmol, 5%) as a colourless oil. TLC (pentane:EtOAc 9:1): R_f = 0.51 [$KMnO_4$]. ^{1}H-NMR (400 MHz, $CDCl_3$, 298 K): δ [ppm] = 0.15 (s, 9H), 1.78-1.88 (apparent p, $J \approx 7.0$ Hz, 2H), 2.29 (t, $J = 6.9$ Hz, 2H), 2.74 (t, $J = 7.1$ Hz, 2H), 4.10 (s, 2H). ^{13}C-NMR (101 MHz, $CDCl_3$, 300 K): δ [ppm] = 0.2 (s, 3C), 19.2 (s, 1C), 22.3 (s, 1C), 38.3 (s, 1C), 48.4 (s, 1C), 86.1 (s, 1C), 105.9 (s, 1C), 202.3 (s, 1C).

1-Bromo-7-(trimethylsilyl)hept-6-yn-2-one (5): NaBr (1.01 g, 9.78 mmol, 6.17 equiv.) was added to a solution of the above mixture of **4** (345 mg, 1.59 mmol, 1.00 equiv.) and **5** (79.2 mg, 303 µmol, 0.19 equiv.) in dry acetone (10 mL) and the mixture was stirred at 70 °C for six days over which additional NaBr (2.62 g, 25.4 mmol, 16.0 equiv.) was added in portions until completion of the reaction was indicated by ^{1}H-NMR. Pentane (10 mL) was added, the suspension was filtered and the filtrate was washed with water (5 mL). The organic layer was dried over Na_2SO_4 and concentrated *in vacuo* to give **5** (290 mg, 1.11 mmol, 19% over two steps) as a light-yellow oil, which was used without further purifications. TLC (pentane:EtOAc 9:1): R_f = 0.51 [$KMnO_4$]. ^{1}H-NMR (400 MHz, $CDCl_3$, 298 K): 0.14 (s, 9H), 1.78-1.88 (apparent p, $J \approx 7.0$ Hz, 2H), 2.29 (t, $J = 6.8$ Hz, 2H), 2.80 (t, $J = 7.1$ Hz, 2H), 3.90 (s, 2H).

^{13}C-NMR (101 MHz, $CDCl_3$, 300 K): δ [ppm] = 0.3 (s, 3C), 19.2 (s, 1C), 22.6 (s, 1C), 34.4 (s, 1C), 38.4 (s, 1C), 86.1 (s, 1C), 106.0 (s, 1C), 201.8 (s, 1C).

1-Bromohept-6-yn-2-one (BMK-alkyne): $AgNO_3$ (10.7 mg, 63.0 µmol, 10 mol%) was added to a solution of **5** (165 mg, 761 µmol, 1.00 equiv.) in acetone (4.7 mL) and water (1.1 mL) and the mixture was stirred for 68 h in the dark. After addition of H_2O (7 mL), the reaction mixture was extracted with Et_2O (6 × 3.5 mL), the combined organic layers were washed with H_2O (3.5 mL), dried over Na_2SO_4, filtered and concentrated *in vacuo*.* Column chromatography (pentane:Et_2O 94:6) gave **BMK-alkyne** (61.0 mg, 323 µmol, 51%) as a light yellow oil. TLC (pentane:Et_2O 9:1): R_f = 0.30 [$KMnO_4$]. ^1H-NMR (300 MHz, $CDCl_3$, 300 K): 1.78-1.91 (apparent p, $J \approx 7.0$ Hz, 2H), 1.98 (t, J = 2.7 Hz, 2H), 2.26 (td, J = 6.9, 2.7 Hz, 2H), 2.81 (t, J = 7.1 Hz, 2H), 3.90 (s, 2H). ^{13}C-NMR (101 MHz, $CDCl_3$, 300 K): δ [ppm] = 17.7 (s, 1C), 22.5 (s, 1C), 34.4 (s, 1C), 38.4 (s, 1C), 69.6 (s, 1C), 83.3 (s, 1C), 201.6 (s, 1C).

*washing the organic layers with brine resulted in transhalogenation.

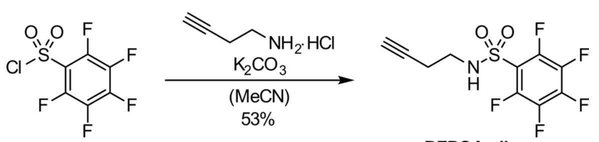

***N*-(But-3-yn-1-yl)-2,3,4,5,6-pentafluorobenzenesulfonamide (PFPSA-alkyne):** Pentafluorobenzenesulfonyl chloride (105 µL, 189 mg, 710 µmol, 1.00 equiv.) was added to a solution of but-3-ynylamine hydrochloride (75.0 mg, 710 µmol, 1.00 equiv.) and K_2CO_3 (108 mg, 782 µmol, 1.10 equiv.) in dry MeCN (5 mL) at 0 °C. The reaction mixture was stirred overnight, concentrated *in vacuo* and the resulting residue was dissolved in CH_2Cl_2 (5 mL). After washing with aqueous HCl (0.1 M), saturated aqueous $NaHCO_3$ and brine, the organic layer was dried over Na_2SO_4, filtered and concentrated *in vacuo*. Column chromatography (pentane:EtOAc 2:1) gave **PFPSA-alkyne** (113 mg, 379 µmol, 53%) as a white solid. TLC (pentane:EtOAc 2:1): R_f = 0.73 [UV, $KMnO_4$]. ^1H-NMR (400 MHz, $CDCl_3$, 298 K): δ [ppm] = 2.05 (t, J = 2.6 Hz, 1H), 2.47 (td, J = 6.3, 2.6 Hz, 2H), 3.31-3.39 (apparent q, $J \approx 6.3$ Hz, 2H), 5.39 (t, J = 5.8 Hz, 1H). ^{13}C-NMR (101 MHz, $CDCl_3$, 298 K): δ [ppm] = 20.0 (s, 1C), 42.3 (s, 1C), 71.6 (s, 1C), 79.7 (s, 1C), 136.8, 139.3, 142.8, 143.3, 145.4, 145.8.* ^{19}F-NMR (376 MHz, $CDCl_3$, 298 K): δ [ppm] = −158.5-−158.3 (m, 2F), −145.8 (tt, J = 21.1, 6.4 Hz, 1F), −136.8-−136.6 (m, 2F). HRMS-APCI (m/z): calc. ($C_{10}H_5F_5NO_2S$ $[M-H]^-$): 297.9967; found: 297.9967.

*aromatic ^{13}C-signals could not be clearly identified as a result of ^{13}C-^{19}F-coupling.

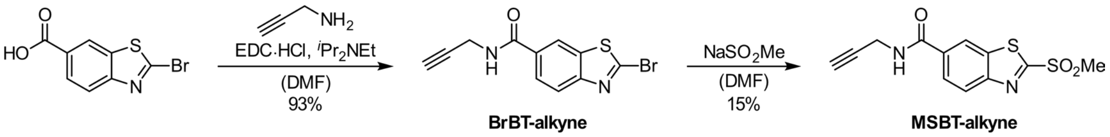

2-Bromo-*N*-(prop-2-yn-1-yl)benzo[*d*]thiazole-6-carboxamide (BrBT-alkyne): Synthesis of **BrBT-alkyne** was carried out similar to literature procedure.[51] EDC·HCl (445 mg, 2.32 mmol, 1.50 equiv.) was added to a solution of 2-bromobenzo[d]thiazole-6-carboxylic acid (400 mg, 1.55 mmol, 1.00 equiv.) and propargylamine (248 µL, 213 mg, 3.87 mmol, 2.50 equiv.) in dry DMF (4 mL). After 5 min, $^iPr_2NEt$ (658 µL, 488 mg, 3.78 mmol, 2.44 equiv.) was added dropwise and stirring was continued for 4 h. The reaction mixture was concentrated *in vacuo*, then diluted with water and acidified with a few drops of aqueous HCl (1 M). The resulting suspension was sonicated for 1 min and stirred for 5 min before filtration. The filter residue was washed with H_2O and then dried *in vacuo*. Column chromatography (CH_2Cl_2:MeOH 19:1) gave **BrBT-alkyne** (427 mg, 1.45 mmol, 93%) as a yellow-orange solid. TLC (CH_2Cl_2:MeOH 19:1): R_f = 0.43 [UV, $KMnO_4$]. ^1H-NMR (300 MHz, CD_3CN, 300 K): δ [ppm] = 2.49 (t, J = 2.5 Hz, 1H), 4.16 (dd, J = 5.6, 2.5 Hz, 2H), 7.46 (br. s, 1H), 7.89 (dd, J = 8.6, 1.8 Hz, 1H), 8.01 (dd, J = 8.6, 0.5 Hz, 1H), 8.41 (dd, J = 1.8, 0.5 Hz, 1H). ^{13}C-NMR (75.5 MHz, CD_3CN, 300 K): δ [ppm] = 29.9 (s, 1C), 71.9 (s, 1C), 81.3 (s, 1C), 122.2 (s, 1C), 123.3 (s, 1C), 126.5 (s, 1C), 132.6 (s, 1C), 138.6 (s, 1C), 142.8 (s, 1C), 155.1 (s, 1C), 166.8 (s, 1C). HRMS-ESI (m/z): calc. ($C_{11}H_6BrN_2OS$ $[M-H]^-$): 292.9390; found: 292.9392.

2-(Methylsulfonyl)-*N*-(prop-2-yn-1-yl)benzo[*d*]thiazole-6-carboxamide (MSBT-alkyne): Synthesis of **MSBT-alkyne** was carried out similar to literature procedure.[51] $NaSO_2Me$ (132 mg, 1.30 mmol, 1.50 equiv.) was

added to a solution of **BrBT-alkyne** (255 mg, 864 µmol, 1.00 equiv.) in dry DMF (3.8 mL). After stirring for 3.5 h at 70 °C the solvent was removed *in vacuo*. Column chromatography (CH_2Cl_2:MeOH 19:1) gave **MSBT-alkyne** (37.5 mg, 127 µmol, 15%) as a white solid. TLC (CH_2Cl_2:MeOH 19:1): R_f = 0.51 [UV, $KMnO_4$]. ^{1}H-NMR (500 MHz, CD_3CN, 300 K): δ [ppm] = 2.50 (t, J = 2.5 Hz, 1H), 3.41 (s, 3H), 4.18 (dd, J = 5.6, 2.6 Hz, 2H), 7.53 (br. s, 1H), 8.05 (dd, J = 8.7, 1.8 Hz, 1H), 8.26 (dd, J = 8.7, 0.5 Hz, 1H), 8.60 (d, J = 1.8, 0.5 Hz, 1H). ^{13}C-NMR (75.5 MHz, CD_3CN, 300 K): δ [ppm] = 29.9 (s, 1C), 43.0 (s, 1C), 72.0 (s, 1C), 81.1 (s, 1C), 123.7 (s, 1C), 125.9 (s, 1C), 127.5 (s, 1C), 134.7 (s, 1C), 137.7 (s, 1C), 155.3 (s, 1C), 166.6 (s, 1C), 170.6 (s, 1C). HRMS-ESI (m/z): calc. ($C_{12}H_{11}N_2O_3S_2$ $[M+H]^+$): 295.0206; found: 295.0204. The analytical data is in accordance with literature.[51]

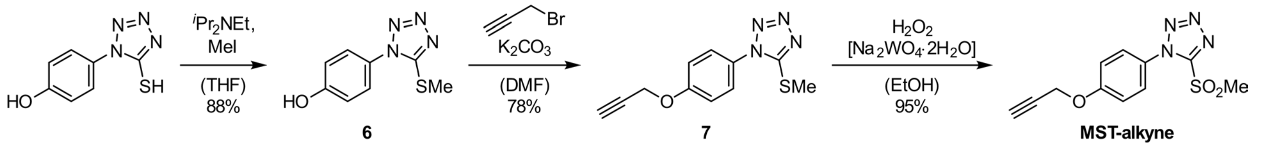

4-(5-(Methylthio)-1*H*-tetrazol-1-yl)phenol (6): Synthesis of **6** was carried out similar to literature procedure.[51] At 0 °C, ^{i}Pr$_2$NEt (753 µL, 559 mg, 4.33 mmol, 1.20 equiv.) was slowly added to a solution of 4-(5-mercapto-1H-tetrazol-1-yl)phenol (700 mg, 3.60 mmol, 1.00 equiv.) in dry THF (10 mL). After 5 min iodomethane (269 µL, 614 mg, 4.33 mmol, 1.20 equiv.) was added, stirring was continued at 0 °C for 10 min before removal of the ice bath. After stirring for 21 h at room temperature, EtOAc (70 mL) was added and the reaction mixture was washed with brine (3 × 100 mL), dried over Na_2SO_4, filtered and concentrated *in vacuo* to give **6** (663 mg, 3.18 mmol, 88%) as a white solid, which was used without further purification. TLC (pentane:EtOAc 1:1): R_f = 0.40 [UV, $KMnO_4$]. ^{1}H-NMR (400 MHz, $CDCl_3$, 298 K): δ [ppm] = 2.82 (s, 3H), 6.20 (br.s, 1H), 6.98-7.12 (m, 2H), 7.36-7.50 (m, 2H). HRMS-ESI (m/z): calc. ($C_8H_9N_4OS$ $[M+H]^+$): 209.0492; found: 209.0490. The analytical data is in accordance with literature.[52]

5-(Methylthio)-1-(4-(prop-2-yn-1-yloxy)phenyl)-1*H*-tetrazole (7): Synthesis of **7** was carried out similar to literature procedure.[51] K_2CO_3 (598 mg, 4.33 mmol, 1.36 equiv.) was added to a solution of **6** (663 mg, 3.18 mmol, 1.00 equiv.) in dry DMF (5 mL). After 30 min, propargyl bromide (80 wt% in PhMe, 482 µL, 515 mg, 4.33 mmol, 1.36 equiv.) was slowly added. After stirring for 21 h, H_2O (70 mL) was added and the resulting suspension was sonicated for 1 min, stirred 5 min and then filtered. The solid was washed with H_2O and *n*-hexane, and drying *in vacuo* gave **7** (613 mg, 2.49 mmol, 78%) as a white solid. TLC (pentane:EtOAc 1:1): R_f = 0.59 [UV, $KMnO_4$]. ^{1}H-NMR (400 MHz, $CDCl_3$, 298 K): δ [ppm] = 2.58 (t, J = 2.4 Hz, 1H), 2.82 (s, 3H), 4.77 (d, J = 2.4 Hz, 2H), 7.11-7.17 (m, 2H), 7.45-7.55 (m, 2H). ^{13}C-NMR (101 MHz, $CDCl_3$, 298 K): δ [ppm] = 15.5 (s, 1C), 56.3 (s, 1C), 76.5 (s, 1C), 77.8 (s, 1C), 116.1 (s, 2C), 125.5 (s, 2C), 127.3 (s, 1C), 155.2 (s, 1C), 158.7 (s, 1C). HRMS-ESI (m/z): calc. ($C_{11}H_{11}N_4OS$ $[M+H]^+$): 247.0648; found: 247.0647. The analytical data is in accordance with literature.[51]

5-(Methylsulfonyl)-1-(4-(prop-2-yn-1-yloxy)phenyl)-1*H*-tetrazole (MST-alkyne): Synthesis of **MST-alkyne** was carried out similar to literature procedure.[51] $Na_2WO_4 \cdot 2H_2O$ (22.8 mg, 69.0 µmol, 10 mol%) and H_2O_2 (30 wt% in H_2O, 1.50 mL, 500 mg, 14.7 mmol, 21.3 equiv.) were added to a suspension of **7** (170 mg, 690 µmol, 1.00 equiv.) in EtOH (2 mL) at 0 °C and the ice bath was removed after 40 min. After stirring for 20 h at room temperature, the reaction mixture was concentrated *in vacuo*. Column chromatography (CH_2Cl_2) gave **MST-alkyne** (182 mg, 654 µmol, 95%) as a white solid. TLC (CH_2Cl_2): R_f = 0.42 [UV, $KMnO_4$]. ^{1}H-NMR (400 MHz, $CDCl_3$, 298 K): δ [ppm] = 2.58 (t, J = 2.4 Hz, 1H), 3.62 (s, 3H), 4.78 (d, J = 2.4 Hz, 2H), 7.12-7.20 (m, 2H), 7.58-7.66 (m, 2H). ^{13}C-NMR (101 MHz, $CDCl_3$, 298 K): δ [ppm] = 43.9 (s, 1C), 56.3 (s, 1C), 76.7 (s, 1C), 77.6 (s, 1C), 116.0 (s, 2C), 126.4 (s, 1C), 126.5 (s, 2C), 154.1 (s, 1C), 159.8 (s, 1C). HRMS-ESI (m/z): calc. ($C_{11}H_{11}N_4O_3S$ $[M+H]^+$): 279.0546; found: 279.0544. The analytical data is in accordance with literature.[51]

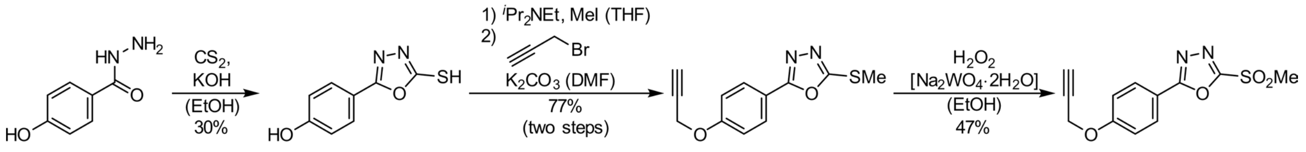

8 9 MSOD-alkyne

4-(5-Mercapto-1,3,4-oxadiazol-2-yl)phenol (8): Synthesis of **8** was carried out similar to literature procedure.[51] CS_2 (1.21 mL, 1.52 g, 20.0 mmol, 4.00 equiv.) was added to a suspension of 4-hydroxybenzohydrazide (761 mg, 5.00 mmol, 1.00 equiv.) and KOH (561 mg, 10.0 mmol, 2.00 equiv.) in EtOH (17.5 mL). After stirring for 19 h at 80 °C, the suspension was cooled to room temperature, filtered, washed with EtOH, dried *in vacuo*, resuspended in H_2O (5 mL) by sonication and acidified with aqueous HCl (1M). The suspension was sonicated for 5 min, filtered, washed with H_2O and dried for 10 min at 130 °C. Additional drying *in vacuo* gave **8** (296 mg, 1.52 mmol, 30%) as a white solid, which was used without further purification. TLC (pentane:EtOAc 1:1): R_f = 0.27 [UV, $KMnO_4$]. ^{1}H-NMR (300 MHz, CD_3CN, 300 K): δ [ppm] = 6.90-6.99 (m, 2H), 7.71-7.80 (m, 2H), 9.47 (br. s, 2H). ^{13}C-NMR (75.5 MHz, CD_3CN, 300 K): δ [ppm] = 115.5 (s, 1C), 117.0 (s, 2C), 129.3 (s, 2C), 161.5 (s, 1C), 162.3 (s, 1C), 179.0 (s, 1C). HRMS-ESI (m/z): calc. ($C_8H_7N_2O_2S$ $[M+H]^+$): 195.0223; found: 195.0222. The analytical data is in accordance with literature.[51]

2-(Methylthio)-5-(4-(prop-2-yn-1-yloxy)phenyl)-1,3,4-oxadiazole (9): Synthesis of **9** was carried out similar to literature procedure.[51] At 0 °C, $^{i}Pr_2NEt$ (270 µL, 200 mg, 1.55 mmol, 2.00 equiv.) was slowly added to a solution of **8** (150 mg, 772 µmol, 1.00 equiv.) in dry THF (3 mL). After 5 min iodomethane (52.9 µL, 121 mg, 849 µmol, 1.10 equiv.) was added and stirring was continued for 30 min before concentration *in vacuo* to give a white semi-solid (161 mg), which was used without further purification. This residue was dissolved in dry DMF (2 mL) and K_2CO_3 (213 mg, 1.54 mmol, 2.00 equiv.) was added. After 30 min, propargyl bromide (80 wt% in PhMe, 258 µL, 276 mg, 2.32 mmol, 3.00 equiv.) was slowly added. The reaction mixture was stirred for 16 h and then concentrated *in* vacuo. The resulting residue was suspended in H_2O (2 mL), acidified with aqueous HCl (1M), sonicated, filtered and washed with H_2O. Drying *in vacuo* gave **9** (146 mg, 593 µmol, 77%) as a grey solid, which was used directly without further purification. TLC (pentane:EtOAc 1:1): R_f = 0.50 [UV, $KMnO_4$]. ^{1}H-NMR (300 MHz, $CDCl_3$, 300 K): δ [ppm] = 2.56 (t, J = 2.4 Hz, 1H), 2.77 (s, 3H), 4.76 (d, J = 2.4 Hz, 2H), 6.99-7.13 (m, 2H), 7.83-8.04 (m, 2H). ^{13}C-NMR (75.5 MHz, $CDCl_3$, 300 K): δ [ppm] = 14.8 (s, 1C), 56.1 (s, 1C), 76.3 (s, 1C), 77.9 (s, 1C), 115.5 (s, 2C), 117.3 (s, 1C), 128.5 (s, 2C), 160.2 (s, 1C), 164.6 (s, 1C), 165.8 (s, 1C). HRMS-ESI (m/z): calc. ($C_{12}H_{11}N_2O_2S$ $[M+H]^+$): 247.0536; found: 247.0534. The analytical data is in accordance with literature.[51]

2-(Methylsulfonyl)-5-(4-(prop-2-yn-1-yloxy)phenyl)-1,3,4-oxadiazole (MSOD-alkyne): Synthesis of **MSOD-alkyne** was carried out similar to literature procedure.[51] At 0 °C, $Na_2WO_4 \cdot 2H_2O$ (13.4 mg, 40.6 µmol, 20 mol%) and H_2O_2 (30 wt% in H_2O, 300 µL, 99.9 mg, 2.94 mmol, 14.5 equiv.) were added to a suspension of **9** (50.0 mg, 203 µmol, 1.00 equiv.) in EtOH (1 mL). After stirring for 4.5 h at room temperature, the reaction mixture was concentrated *in vacuo*. Column chromatography (CH_2Cl_2) gave **MSOD-alkyne** (26.6 mg, 95.6 µmol 47%) as a white solid. TLC (CH_2Cl_2): R_f = 0.27 [UV, $KMnO_4$]. ^{1}H-NMR (300 MHz, $CDCl_3$, 300 K): δ [ppm] = 2.58 (t, J = 2.4 Hz, 1H), 3.51 (s, 3H), 4.79 (d, J = 2.4 Hz, 2H), 7.09-7.18 (m, 2H), 8.06-8.14 (m, 2H). ^{13}C-NMR (101 MHz, $CDCl_3$, 300 K): δ [ppm] = 43.1 (s, 1C), 56.1 (s, 1C), 76.6 (s, 1C), 77.6 (s, 1C), 115.3 (s, 1C), 115.9 (s, 2C), 129.8 (s, 2C), 161.5 (s, 1C), 161.9 (s, 1C), 166.6 (s, 1C). HRMS-ESI (m/z): calc. ($C_{12}H_{11}N_2O_4S$ $[M+H]^+$): 279.0434; found: 279.0433. The analytical data is in accordance with literature.[51]

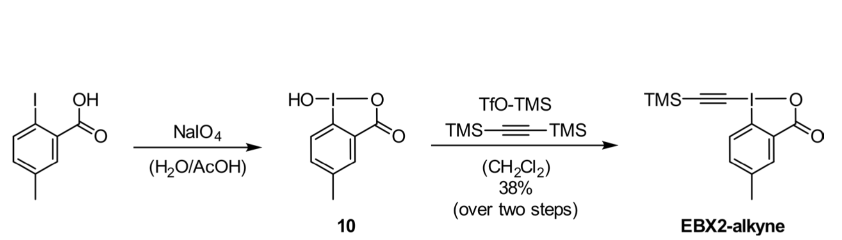

10 EBX2-alkyne

5-Methyl-1-[(trimethylsilyl)ethynyl]-1,2-benziodoxol-3(1*H*)-one (EBX2-alkyne): Synthesis of **EBX2-alkyne** was carried out according to literature procedure.[22] 2-Iodo-5-methylbenzoic acid (9.96 g, 38.0 mmol, 1.00 equiv.) and $NaIO_4$ (8.53 g, 39.9 mmol, 1.05 equiv.) in 30% aqueous AcOH (100 mL) were heated to reflux for 4 h. After addition of 270 mL water, the reaction mixture was allowed to cool to room temperature and was stirred in the dark for 45 min. The suspension was filtered and the residue washed with ice water (3 × 25 mL) and cold acetone (3 × 25 mL). Air-drying the product overnight in the dark gave intermediate **10** (9.62 g) as a colourless solid, which was used without further purification. To a solution of **10** (9.55 g, 34.4 mmol, 1.00 equiv.) in CH_2Cl_2 (100 mL), TfO-TMS

(6.86 mL, 8.44 g, 38.0 mmol, 1.11 equiv.) was added dropwise and the mixture was stirred for 1 h. Bis(trimethylsilyl)acetylene (8.60 mL, 6.80 g, 39.9 mmol, 1.16 equiv.) was added dropwise and stirring was continued for 6 h, before addition of saturated aqueous $NaHCO_3$ (100 mL) and stirring for additional 30 min. The layers were separated and the organic layer was washed with saturated aqueous $NaHCO_3$ (3 × 50 mL), dried over Na_2SO_4, filtered and concentrated *in vacuo*. Recrystallization from MeCN (40 mL) gave **EBX2-alkyne** (5.16 g, 14.4 mmol, 38% over two steps) as a white solid. ^{1}H-NMR (300 MHz, $CDCl_3$, 300 K): δ [ppm] = 0.28 (s, 9H), 2.47 (s, 3H), 7.52-7.60 (apparent ddd, J = 8.5, 2.2, 0.6 Hz, 1H), 7.99 (d, J = 8.5 Hz, 1H), 8.18 (dd, J = 2.2, 0.6 Hz, 1H). ^{13}C-NMR (75.5 MHz, $CDCl_3$, 300 K): δ [ppm] = −0.3 (s, 3C), 20.8 (s, 1C), 64.1 (s, 1C), 111.8 (s, 1C), 116.6 (s, 1C), 126.0 (s, 1C), 131.3 (s, 1C), 133.0 (s, 1C), 135.8 (s, 1C), 142.5 (s, 1C), 166.8 (s, 1C). HRMS-APCI (m/z): calc. ($C_{13}H_{16}IO_2Si$ [M+H]$^+$): 358.9959; found: 358.9958. The analytical data is in accordance with literature.[22]

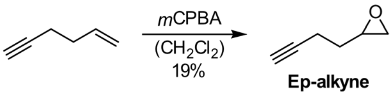

2-(But-3-yn-1-yl)oxirane (Ep-alkyne): A solution of *m*CPBA (77 wt%, 339 mg, 1.97 mmol, 1.05 equiv.) in CH_2Cl_2 (11 mL) was added dropwise to a solution of hex-1-en-5-yne (150 mg, 1.87 mmol, 1.00 equiv.) in CH_2Cl_2 (7.5 mL) over 50 min. After stirring for 10 min at room temperature, the mixture was heated to reflux for 1 h and then stirred again at room temperature for 16 h. Pentane (2.5 mL) was added at 0 °C, the solid was filtered off and rinsed with pentane. The filtrate was washed with saturated aqueous $NaHSO_3$ (3 × 15 mL), aqueous $NaHCO_3$ (5 wt%, 3 × 15 mL) and brine (15 mL), dried over Na_2SO_4, filtered and carefully concentrated *in vacuo* (≥ 550 mbar at 40 °C). Column chromatography (pentane → pentane:Et_2O 20:1) gave **Ep-alkyne** (34.9 mg, 363 µmol, 19%) as a colourless oil. TLC (pentane:Et_2O 5:1): R_f = 0.59 [$KMnO_4$]. ^{1}H-NMR (300 MHz, CD_2Cl_2, 300 K): δ [ppm] = 1.62-1.85 (m, 2H), 2.02 (t, J = 2.7 Hz, 1H), 2.34 (ddd, J = 7.5, 7.0, 2.7 Hz, 2H), 2.50 (dd, J = 5.0, 2.6 Hz, 1H), 2.75 (ddd, J = 5.0, 4.0, 0.3 Hz, 1H), 3.00 (dddd, J = 6.5, 4.9, 4.0, 2.6 Hz, 1H). ^{13}C-NMR (101 MHz, CD_2Cl_2, 298 K): δ [ppm] = 15.7 (s, 1C), 32.1(s, 1C), 47.4 (s, 1C), 51.4 (s, 1C), 69.1 (s, 1C), 83.9 (s, 1C).

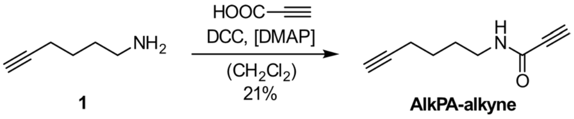

***N*-(Hex-5-yn-1-yl)propiolamide (AlkPA-alkyne):** Propiolic acid (58.2 µL, 65.8 mg, 939 µmol, 1.10 equiv.) was added to a mixture of **1** (82.9 mg, 854 µmol, 1.00 equiv.), DCC (203 mg, 982 µmol, 1.15 equiv.) and DMAP (15.6 mg, 128 µmol, 15 mol%) in dry CH_2Cl_2 (1 mL) at 0 °C. The reaction mixture was stirred overnight during which it was allowed to warm to room temperature. The precipitate was removed by filtration and the filtrate was concentrated *in vacuo*. Column chromatography (Pentane:EtOAc 4:1 → 2:1) gave **AlkPA-alkyne** (27.3 mg, 183 µmol, 21%) as a colourless oil. The observed ratio of cis/trans-isomers in the ^{1}H-NMR experiment is 1:10. TLC (pentane:EtOAc 1:1): R_f = 0.41 [$KMnO_4$]. ^{1}H-NMR (300 MHz, $CDCl_3$, 300 K): δ [ppm] = 1.50-1.75 (m, 4H, both), 1.96 (t, J = 2.7 Hz, 1H, both), 2.23 (td, J = 6.8, 2.7 Hz, 2H, major), 2.20-2.28 (m, 2H, minor), 2.77 (s, 1H, major), 3.11 (s, 1H, minor), 3.29-3.38 (apparent q, $J \approx$ 6.7 Hz, 2H, major), 3.43-3.51 (apparent q, $J \approx$ 6.7 Hz, 2H, minor), 5.98 (br. s, 1H, both). ^{13}C-NMR (75.5 MHz, CD_3CN, 300 K): δ [ppm] = 18.4 (s, 1C, major), 26.2 (s, 1C, minor), 26.5 (s, 1C, major), 28.9 (s, 1C, major), 30.4 (s, 1C, minor), 39.6 (s, 1C, major), 43.3 (s, 1C, minor), 69.9 (s, 1C, major), 73.9 (s, 1C, major), 78.4 (s, 1C, major), 80.1 (s, 1C, minor), 85.1 (s, 1C, major), 152.8 (s, 1C, major).* HRMS-ESI (m/z): calc. ($C_9H_{11}NO$ [M+H]$^+$): 150.0913; found: 150.0911.

*individual ^{13}C-signals could not be detected for the minor isomer due to their low intensity.

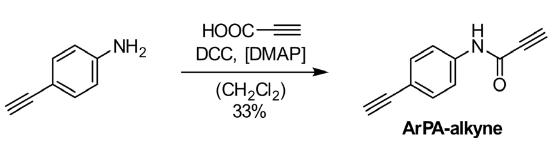

***N*-(4-Ethynylphenyl)propiolamide (ArPA-alkyne):** Propiolic acid (58.2 µL, 65.8 mg, 939 µmol, 1.10 equiv.) was added to a mixture of 4-ethynylaniline (100 mg, 854 µmol, 1.00 equiv.), DCC (203 mg, 982 µmol, 1.15 equiv.) and DMAP (15.6 mg, 128 µmol, 15 mol%.) in dry CH_2Cl_2 (1 mL) at 0 °C. The reaction mixture was stirred overnight during which it was allowed to warm to room temperature. The precipitate was removed by filtration and the filtrate was

concentrated *in vacuo*. Column chromatography (Pentane:EtOAc 7:1 → 6:1) gave **ArPA-alkyne** (48.0 mg, 284 µmol, 33%) as a brown solid. TLC (pentane:EtOAc 1:1): R_f = 0.51 [UV, $KMnO_4$]. ^{1}H-NMR (400 MHz, $CDCl_3$, 298 K): *δ* [ppm] = 2.95 (s, 1H), 3.07 (s, 1H), 7.40-7.57 (m, 4H), 7.76 (br. s, 1H). ^{1}H-NMR (400 MHz, CD_3CN, 298 K): *δ* [ppm] = 3.36 (s, 1H), 3.37 (s, 1H), 7.42-7.49 (m, 2H), 7.52-7.59 (m, 2H), 8.97 (br. s, 1H). ^{13}C-NMR (101 MHz, CD_3CN, 298 K): *δ* [ppm] = 75.9 (s, 1C), 78.1 (s, 1C), 78.7 (s, 1C), 83.8 (s, 1C), 118.8 (s, 1C), 120.6 (s, 2C), 133.7 (s, 2C), 139.4 (s, 1C), 150.8 (s, 1C). HRMS-ESI (m/z): calc. ($C_{11}H_{18}NO$ $[M+H]^+$): 170.0600; found: 170.0599.

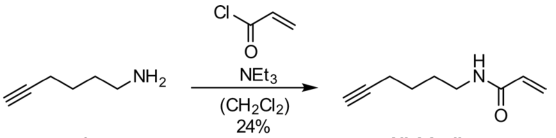

1 AlkAA-alkyne

***N*-(Hex-5-yn-1-yl)acrylamide (AlkAA-alkyne):** Acryloyl chloride (134 µL, 149 mg, 1.64 mmol, 1.20 equiv.)* was added dropwise to a solution of **1** (133 mg, 1.37 mmol, 1.00 equiv.) and NEt_3 (210 µL, 152 mg, 1.51 mmol, 1.10 equiv.) in dry CH_2Cl_2 (2.1 mL) at 0 °C. The reaction mixture was allowed to warm to room temperature while stirring for 18 h and H_2O (1.5 mL) was added. The layers were separated and the organic layer was washed with H_2O (3 × 1.5 mL). The combined aqueous layers were extracted with CH_2Cl_2 (3 × 2 mL) and the combined organic layers were washed with aqueous NaOH (1 wt%, 3 × 1.5 mL) and brine (2 × 1.5 mL) and then dried over Na_2SO_4, filtered and concentrated *in vacuo*. Column chromatography (pentane:EtOAc 1:1) gave **AlkAA-alkyne** (50.0 mg, 331 µmol, 24%) as a white solid. TLC (pentane:EtOAc 1:1): R_f = 0.18 [$KMnO_4$]. ^{1}H-NMR (300 MHz, $CDCl_3$, 300 K): *δ* [ppm] = 1.51-1.74 (m, 4H), 1.96 (t, *J* = 2.6 Hz, 1H), 2.23 (td, *J* = 6.7, 2.6 Hz, 2H), 3.31-3.42 (apparent q, *J* ≈ 6.7 Hz, 2H), 5.63 (dd, *J* = 10.2, 1.6 Hz, 1H), 5.67 (br. s, 1H), 6.08 (dd, *J* = 17.0, 10.2 Hz, 1H), 6.27 (dd, *J* = 17.0, 1.6 Hz, 1H). ^{13}C-NMR (75.5 MHz, $CDCl_3$, 300 K): *δ* [ppm] = 18.2 (s, 1C), 25.8 (s, 1C), 28.8 (s, 1C), 39.2 (s, 1C), 68.9 (s, 1C), 84.1 (s, 1C), 126.5 (s, 1C), 131.0 (s, 1C), 165.7 (s, 1C). HRMS-ESI (m/z): calc. ($C_9H_{14}NO$ $[M+H]^+$): 152.1070; found: 152.1070.

*CAUTION: acryloyl chloride is highly toxic.

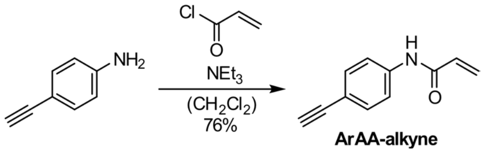

ArAA-alkyne

***N*-(4-Ethynylphenyl)acrylamide (ArAA-alkyne):** Synthesis of **ArAA-alkyne** was carried out similar to literature procedure.[53] Acryloyl chloride (124 µL, 139 mg, 1.54 mmol, 1.20 equiv.)* was added dropwise to a solution of 4-ethynylaniline (150 mg, 1.28 mmol, 1.00 equiv.) in dry CH_2Cl_2 (2 mL) at 0 °C, followed by dropwise addition of dry NEt_3 (214 µL, 156 mg, 1.54 mmol, 1.20 equiv.). After 1 h the reaction mixture was allowed to warm to room temperature and stirring was continued for 1 h. H_2O (7 mL) was added, layers were separated and the organic layer was washed with H_2O, brine, dried over Na_2SO_4, filtered and concentrated *in vacuo*. Column chromatography (pentane:EtOAc 7:1 → 3:1) gave **ArAA-alkyne** (166 mg, 970 µmol, 76%) as a white solid. TLC (pentane:EtOAc 1:1): R_f = 0.47 [UV, $KMnO_4$]. ^{1}H-NMR (400 MHz, CD_3CN, 298 K): *δ* [ppm] = 3.34 (s, 1H), 5.75 (dd, *J* = 6.9, 5.1 Hz, 1H), 6.33 (s, 1H), 6.35 (d, *J* = 1.9 Hz, 1H), 7.41-7.47 (m, 2H), 7.62-7.67 (m, 2H), 8.57 (br. s, 1H). ^{13}C-NMR (101 MHz, CD_3CN, 298 K): *δ* [ppm] = 78.3 (s, 1C), 84.1 (s, 1C), 118.0 (s, 1C), 120.3 (s, 2C), 128.0 (s, 1C), 132.3 (s, 1C), 133.6 (s, 2C), 140.5 (s, 1C), 165.6 (s, 1C). HRMS-ESI (m/z): calc. ($C_{11}H_{10}NO$ $[M+H]^+$): 172.0757; found: 172.0756. The analytical data is in accordance with literature.[53]

*CAUTION: acryloyl chloride is highly toxic.

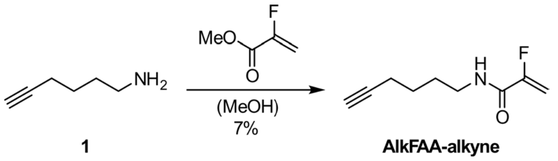

2-Fluoro-*N*-(hex-5-yn-1-yl)acrylamide (AlkFAA-alkyne): To a solution of methyl 2-fluoroacrylate (224 µL, 250 mg, 2.40 mmol, 1.00 equiv.) in MeOH (2 mL), a solution of **1** (350 mg, 3.60 mmol, 1.50 equiv.) in MeOH (4 mL) was added dropwise. After stirring for 22 h, the mixture was concentrated *in vacuo* and column chromatography (pentane:EtOAc 3:1) gave **AlkFAA** (43.0 mg, 254 µmol, 7%) as a yellow oil. TLC (pentane:EtOAc 1:1): R_f = 0.57 [$KMnO_4$]. ^{1}H-NMR (400 MHz, $CDCl_3$, 298 K): δ [ppm] = 1.52-1.63 (m, 2H), 1.63-1.74 (m, 2H), 1.96 (t, J = 2.6 Hz, 1H), 2.23 (td, J = 6.9, 2.6 Hz, 2H), 3.34-3.41 (apparent q, $J \approx$ 6.7 Hz, 2H), 5.10 (dd, J = 15.4, 3.2 Hz, 1H), 5.66 (dd, J = 47.9, 3.2 Hz, 1H), 6.35 (br. s, 1H). ^{13}C-NMR (101 MHz, $CDCl_3$, 300 K): δ [ppm] = 18.2 (s, 1C), 25.7 (s, 1C), 28.5 (s, 1C), 39.0 (s, 1C), 69.0 (s, 1C), 83.9 (s, 1C), 98.9 (d, J = 14.9 Hz, 1C), 156.5 (d, J = 270.1 Hz, 1C), 159.7 (d, J = 30.7 Hz, 1C). ^{19}F-NMR (376 MHz, $CDCl_3$, 300 K): δ [ppm] = −121.2 (s). HRMS-ESI (m/z): calc. ($C_9H_{13}FNO$ $[M+H]^+$): 170.0976; found: 170.0976.

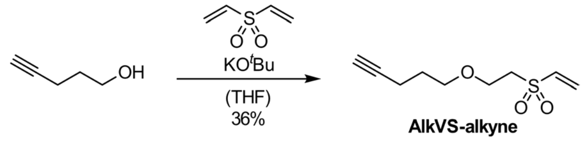

5-(2-(Vinylsulfonyl)ethoxy)pent-1-yne (AlkVS-alkyne): KO^{t}Bu (459 mg, 4.09 mmol, 2.00 equiv.) in dry THF (20 mL) was slowly added to a solution of 4-pentyn-1-ol (181 µL, 172 mg, 2.04 mmol, 1.00 equiv.) and divinyl sulfone (821 µL, 966 mg, 8.18 mmol, 4.00 equiv.)* in dry THF (15 mL). After stirring for 1 h, H_2O (10 mL) was added and THF was removed *in vacuo*. The aqueous residue was extracted with EtOAc (5 × 10 mL) and the combined organic layers were concentrated *in vacuo*. Column chromatography (CH_2Cl_2:MeOH 499:1 + 1 vol% NEt_3) gave **AlkVS-alkyne** (150 mg, 742 µmol, 36%) as a yellow oil. TLC (CH_2Cl_2:MeOH 499:1): R_f = 0.28 [$KMnO_4$]. ^{1}H-NMR (300 MHz, $CDCl_3$, 300 K): δ [ppm] = 1.72-1.84 (m, 2H), 1.96 (t, J = 2.7 Hz, 1H), 2.27 (td, J = 7.0, 2.7 Hz, 2H), 3.25 (t, J = 5.7 Hz, 2H), 3.56 (t, J = 6.1 Hz, 2H), 3.85 (t, J = 5.7 Hz, 2H), 6.09 (d, J = 9.9 Hz, 1H), 6.41 (d, J = 16.6 Hz, 1H), 6.72 (dd, J = 16.6, 9.9 Hz, 1H). ^{13}C-NMR (75.5 MHz, $CDCl_3$, 300 K): δ [ppm] = 15.3 (s, 1C), 28.3 (s, 1C), 55.2 (s, 1C), 64.4 (s, 1C), 69.0 (s, 1C), 69.7 (s, 1C), 83.5 (s, 1C), 129.0 (s, 1C), 137.8 (s, 1C). HRMS-ESI (m/z): calc. ($C_9H_{15}NO_3S$ $[M+H]^+$): 203.0736; found: 203.0737.

*CAUTION: divinyl sulfone is highly toxic.

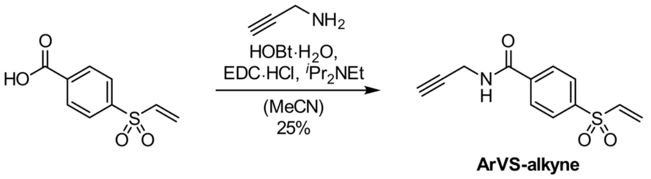

***N*-(Prop-2-yn-1-yl)-4-(vinylsulfonyl)benzamide (ArVS-alkyne):** HOBt·H_2O (41.4 mg, 270 µmol, 1.20 equiv.) and EDC·HCl (51.8 mg, 270 µmol, 1.20 equiv.) were added to 4-(vinylsulfonyl)benzoic acid (47.8 mg, 225 µmol, 1.00 equiv.) in MeCN (2.5 mL). After stirring for two hours, propargylamine (28.8 µL, 24.8 mg, 450 µmol, 2.00 equiv.) and ^{i}Pr$_2$NEt (78.5 µL, 58.2 mg, 450 µmol, 2.00 equiv.) were added and stirring was continued for 2 h. CH_2Cl_2 (20 mL) and H_2O (20 mL) were added, the layers were separated. The aqueous layer was extracted with CH_2Cl_2 (2 × 20 mL) and the combined organic layers were washed with brine, dried over Na_2SO_4, filtered and concentrated *in vacuo*. Column chromatography (pentane:EtOAc 1:1) gave **ArVS-alkyne** (13.8 mg, 55.4 µmol, 25%) as a white solid. TLC (pentane:EtOAc 1:3): R_f = 0.26 [UV, $KMnO_4$]. ^{1}H-NMR (300 MHz, CD_3CN, 300 K): δ [ppm] = 2.49 (t, J = 2.6 Hz, 1H), 4.14 (dd, J = 5.6, 2.6 Hz, 2H), 6.16 (dd, J = 9.9, 0.5 Hz, 1H), 6.44 (dd, J = 16.5, 0.5 Hz, 1H), 6.83 (dd, J = 16.5, 9.9 Hz, 1H), 7.50 (br. s, 1H), 7.92-8.02 (m, 4H). ^{13}C-NMR (126 MHz, CD_3CN, 300 K): δ [ppm] = 29.8 (s, 1C), 72.0 (s, 1C), 81.0 (s, 1C), 128.9 (s, 2C), 129.3 (s, 2C), 130.1 (s, 1C), 138.9 (s, 1C), 139.8 (s, 1C), 143.3 (s, 1C), 166.2 (s, 1C). HRMS-ESI (m/z): calc. ($C_{12}H_{12}NO_3S$ $[M+H]^+$): 250.0532; found: 250.0533.

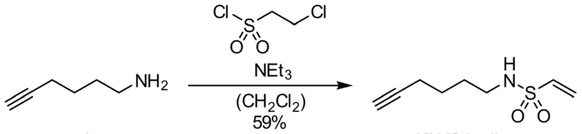

1 **AlkVSA-alkyne**

***N*-(Hex-5-yn-1-yl)ethenesulfonamide (AlkVSA-alkyne):** A solution of 2-chloroethanesulfonyl chloride (113 µL, 176 mg, 1.08 mmol, 1.05 equiv.) in dry CH_2Cl_2 (3 mL) was added dropwise to a solution of **1** (100 mg, 1.03 mmol, 1.00 equiv.) and NEt_3 (359 µL, 260 mg, 2.57 mmol, 2.50 equiv.) in dry CH_2Cl_2 (10 mL) at 0 °C. The reaction mixture was stirred for 1 h at 0 °C and 3 h at room temperature before being concentrated *in vacuo*. Column chromatography (pentane:EtOAc 4:1 → 3:1) gave **AlkVSA-alkyne** (114 mg, 609 µmol, 59%) as a colourless oil. TLC (pentane:EtOAc 2:1): R_f = 0.35 [$KMnO_4$]. ^{1}H-NMR (300 MHz, $CDCl_3$, 300 K): δ [ppm] = 1.52-1.64 (m, 2H), 1.64-1.76 (m, 2H), 1.96 (t, *J* = 2.6 Hz, 1H), 2.23 (td, *J* = 6.7, 2.6 Hz, 2H), 3.00-3.12 (apparent q, *J* ≈ 6.6 Hz, 2H), 4.35 (br. s, 1H), 5.95 (d, *J* = 9.8 Hz, 1H), 6.25 (dd, *J* = 16.6, 0.6 Hz, 1H), 6.52 (dd, *J* = 16.6, 9.8 Hz, 1H). ^{13}C-NMR (101 MHz, $CDCl_3$, 300 K): δ [ppm] = 18.1 (s, 1C), 25.4 (s, 1C), 29.0 (s, 1C), 42.7 (s, 1C), 69.1 (s, 1C), 83.8 (s, 1C), 126.8 (s, 1C), 136.1 (s, 1C). HRMS-ESI (m/z): calc. ($C_8H_{14}NO_2S$ [M+H]$^+$): 188.0740; found: 188.0740.

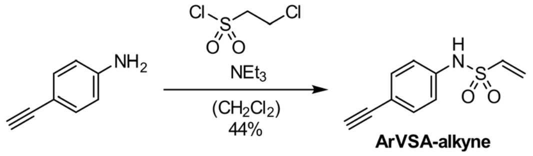

ArVSA-alkyne

***N*-(4-Ethynylphenyl)ethenesulfonamide (ArVSA-alkyne):** A solution of 2-chloroethanesulfonyl chloride (155 µL, 242 mg, 1.48 mmol, 1.05 equiv.) in dry CH_2Cl_2 (3.5 mL) was added dropwise to a solution of 4-ethynylaniline (165 mg, 1.41 mmol, 1.00 equiv.) and NEt_3 (492 µL, 357 mg, 3.53 mmol, 2.50 equiv.) in dry CH_2Cl_2 (14 mL) at 0 °C. The reaction mixture was allowed to warm to room temperature while stirring for 3 h. Additional 2-chloroethanesulfonyl chloride (59.0 µL, 92.1 mg, 565 µmol, 0.40 equiv.) was added at room temperature and the reaction mixture was concentrated *in vacuo* after stirring for 30 min. Column chromatography (pentane:EtOAc 6:1) gave **ArVSA-alkyne** (129 mg, 622 µmol, 44%) as a slightly yellow solid. TLC (pentane:EtOAc 4:1): R_f = 0.17 [UV, $KMnO_4$]. ^{1}H-NMR (500 MHz, $CDCl_3$, 300 K): δ [ppm] = 3.07 (s, 1H), 6.00 (d, *J* = 9.9 Hz, 1H), 6.31 (d, *J* = 16.5 Hz, 1H), 6.56 (dd, *J* = 16.5, 9.9 Hz, 1H), 6.91 (s, 1H), 7.09-7.14 (m, 2H), 7.41-7.46 (m, 2H). ^{13}C-NMR (101 MHz, $CDCl_3$, 300 K): δ [ppm] = 77.7 (s, 1C), 82.9 (s, 1C), 119.0 (s, 1C), 120.1 (s, 2C), 129.1 (s, 1C), 133.5 (s, 2C), 135.1 (s, 1C), 136.9 (s, 1C). HRMS-ESI (m/z): calc. ($C_{10}H_9NO_2S$ [M+H]$^+$): 207.0354; found: 207.0349.

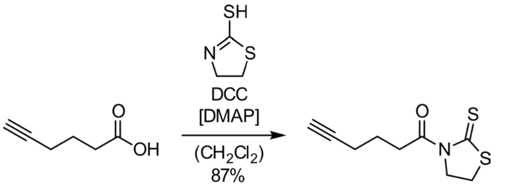

ATT-alkyne

1-(2-Thioxothiazolidin-3-yl)hex-5-yn-1-one (ATT-alkyne): To a solution of 5-hexynoic acid (98.4 µL, 100 mg, 892 µmol, 1.00 equiv.) in CH_2Cl_2 (3 mL), DCC (202 mg, 981 µmol, 1.10 equiv.), 2-thiazoline-2-thiol (117 mg, 981 µmol, 1.10 equiv.) and DMAP (10.9 mg, 89.2 µmol, 10 mol%) were added. The resulting suspension was stirred for 1 h, filtered and CH_2Cl_2 (7 mL) was added to the filtrate. After washing with aqueous NaOH (0.5 M, 10 mL), aqueous HCl (0.1 M, 5 mL) and brine (5 mL), the organic layer was dried over Na_2SO_4, filtered through a plug of silica and concentrated *in vacuo* to give **ATT-alkyne** (166 mg, 778 µmol, 87%) as a pale yellow solid. TLC (pentane:EtOAc 1:1): R_f = 0.58 [UV, $KMnO_4$]. ^{1}H-NMR (400 MHz, $CDCl_3$, 298 K): δ [ppm] = 1.86-1.95 (apparent p, *J* ≈ 7.0 Hz, 2H), 1.97 (t, *J* = 2.6 Hz, 1H), 2.28 (dt, *J* = 7.0, 2.6 Hz, 2H), 3.29 (t, *J* = 7.5 Hz, 2H), 3.37 (t, *J* = 7.2 Hz, 2H), 4.57 (t, *J* = 7.5 Hz, 2H). ^{13}C-NMR (101 MHz, $CDCl_3$, 298 K): δ [ppm] = 17.8 (s, 1C), 23.6 (s, 1C), 28.4 (s, 1C), 37.5 (s, 1C), 56.1 (s, 1C), 69.3 (s, 1C), 83.5 (s, 1C), 174.2 (s, 1C), 201.7 (s, 1C). HRMS-ESI (m/z): calc. ($C_9H_{12}NOS_2$ [M+H]$^+$): 214.0355; found: 214.0360.

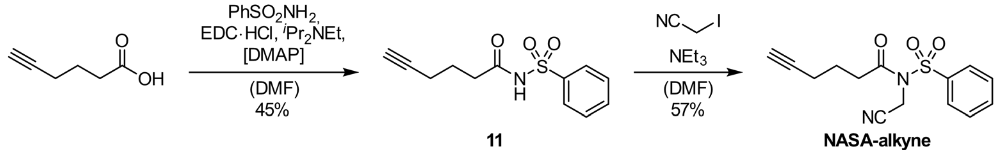

N-(Phenylsulfonyl)hex-5-ynamide (11): 5-Hexynoic acid (579 µL, 589 mg, 5.25 mmol, 1.10 equiv.), EDC·HCl (1.83 g, 9.54 mmol, 2.00 equiv.), DMAP (175 mg, 1.43 mmol, 30 mol%) and $^{i}Pr_2NEt$ (3.71 mL, 2.75 g, 21.3 mmol, 4.46 equiv.) were added to a solution of benzenesulfonamide (750 mg, 4.77 mmol, 1.00 equiv.) in dry DMF (24 mL). After stirring for 40 h, the reaction mixture was concentrated *in vacuo*. Column chromatography (CH_2Cl_2:MeOH 25:1) gave **11** (539 mg, 2.14 mmol, 45%) as a yellow solid. TLC (CH_2Cl_2:EtOAc 1:1): R_f = 0.49 [UV, $KMnO_4$]. ^{1}H-NMR (500 MHz, $CDCl_3$, 298 K): δ [ppm] = 1.74-1.81 (apparent p, $J \approx 7.1$ Hz, 2H), 1.91 (t, J = 2.6 Hz, 1H), 2.17 (dt, J = 6.8, 2.6 Hz, 2H), 2.41 (t, J = 7.3 Hz, 2H), 7.51-7.58 (m, 2H), 7.61-7.67 (m, 1H), 8.03-8.10 (m, 2H), 9.38 (br. s, 1H). ^{13}C-NMR (75.5 MHz, $CDCl_3$, 300 K): δ [ppm] = 17.7 (s, 1C), 22.9 (s, 1C), 34.7 (s, 1C), 69.7 (s, 1C), 83.1 (s, 1C), 128.4 (s, 2C), 129.1 (s, 2C), 134.0 (s, 1C), 139.0 (s, 1C), 170.6 (s, 1C). HRMS-ESI (m/z): calc. ($C_{12}H_{14}NO_3S$ $[M+H]^+$): 252.0689; found: 252.0689.

N-(Cyanomethyl)-N-(phenylsulfonyl)hex-5-ynamide (NASA-alkyne): Iodoacetonitrile (68.5 µL, 158 mg, 948 µmol, 1.61 equiv.) and NEt_3 (264 µL, 192 mg, 1.90 mmol, 3.21 equiv.) were added to a solution of **11** (148 mg, 591 µmol, 1.00 equiv.) in dry DMF (10 mL). After stirring for 16 h, the reaction mixture was concentrated *in vacuo*. Since the conversion was incomplete, the resulting residue was again subjected to the reaction conditions for 18 h, before concentration *in vacuo*. Column chromatography (pentane:EtOAc 3:1) gave **NASA-alkyne** (98.0 mg, 338 µmol, 57%) as an off-white solid. TLC (pentane:EtOAc 1:1): R_f = 0.55 [UV, $KMnO_4$]. ^{1}H-NMR (500 MHz, DMSO-d_6, 298 K): δ [ppm] = 1.81 (m, 2H), 1.88 (t, J = 2.6 Hz, 1H), 2.18-2.24 (td, J = 6.7, 2.6 Hz, 2H), 2.85 (t, J = 7.2 Hz, 2H), 4.78 (s, 2H), 7.64 (t, J = 7.8 Hz, 2H), 7.73 (t, J = 7.5 Hz, 1H), 7.98-8.04 (m, 2H). ^{13}C-NMR (75.5 MHz, $CDCl_3$, 300 K): δ [ppm] = 17.6 (s, 1C), 22.9 (s, 1C), 33.1 (s,1C), 34.8 (s, 1C), 69.7 (s, 1C), 82.9 (s, 1C), 114.8 (s,1C), 127.8 (s, 2C), 130.0 (s, 2C), 135.0 (s, 1C), 138.3 (s, 1C), 171.6 (s, 1C). HRMS-ESI (m/z): calc. ($C_{14}H_{15}N_2O_3S$ $[M+H]^+$): 291.0798; found: 291.0797.

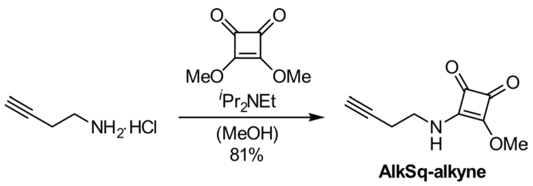

3-(But-3-yn-1-ylamino)-4-methoxycyclobut-3-ene-1,2-dione (AlkSq-alkyne): Dimethyl squarate (85.3 mg, 600 µmol, 1.20 equiv.), 1-amino-3-butyne hydrochloride (52.8 mg, 500 µmol, 1.00 equiv.) and $^{i}Pr_2NEt$ (43.5 µL, 32.3 mg, 250 µmol, 0.50 equiv.) were stirred in MeOH (5 mL) overnight, after which the reaction mixture was concentrated *in vacuo*. Column chromatography (pentane:EtOAc 1:1) gave **AlkSq-alkyne** (72.7 mg, 406 µmol, 81%) as an off-white solid. The observed ratio of cis/trans-isomers in the ^{1}H-NMR experiment is 0.8:1. TLC (pentane:EtOAc 1:4): R_f = 0.48 [UV/$KMnO_4$]. ^{1}H-NMR (400 MHz, DMSO-d_6, 298 K): δ [ppm] = 2.43 (td, J = 6.7, 2.5 Hz, 2H, both), 2.85-2.93 (m, 1H, both), 3.35-3.44 (apparent q, $J \approx 6.4$ Hz, 2H, major), 3.54-3.66 (apparent q, $J \approx 6.3$ Hz, 2H, minor), 4.25-4.33 (m, 3H, both), 8.68 (br. s, 1H, minor), 8.87 (br. s, 1H, major). ^{13}C-NMR (101 MHz, DMSO-d_6, 300 K): δ [ppm] = 19.9 (s, 1C, major), 20.3 (s, 1C, minor), 42.2 (s, 1C, minor), 42.8 (s,1C, major), 60.0 (s, 1C, minor), 60.2 (s, 1C, major), 72.9 (s, 1C, both), 81.2 (s, 1C, both), 172.2 (s, 1C, minor), 172.5 (s, 1C, major), 177.3 (s, 1C, both), 182.3 (s, 1C, minor), 182.5 (s, 1C, minor or major), 189.1 (s, 1C, minor or major), 189.3 (s, 1C. major). HRMS-ESI (m/z): calc. ($C_9H_{10}NO_3$ $[M+H]^+$): 180.0655; found: 180.0654.

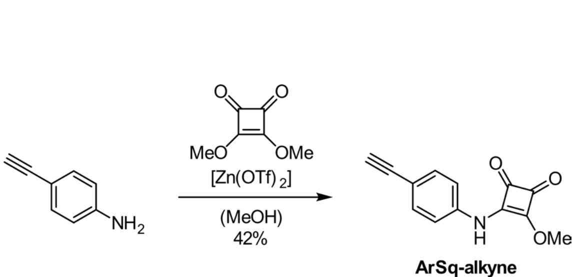

3-Methoxy-4-((4-ethynylphenyl)amino)cyclobut-3-ene-1,2-dione (ArSq-alkyne): Dimethyl squarate (171 mg, 1.20 mmol, 1.19 equiv.), 4-ethynylaniline (118 mg, 1.01 mmol, 1.00 equiv.) and $Zn(OTf)_2$ (74.6 mg, 205 µmol,

20 mol%) were stirred in MeOH (5 mL) for 2 h, after which the reaction mixture was concentrated *in vacuo*. Column chromatography (CH_2Cl_2/EtOAc 9:1) gave **ArSq-alkyne** (95.3 mg, 419 µmol, 42%) as an off-white solid. TLC (pentane:EtOAc 1:4): R_f = 0.61 [UV, $KMnO_4$]. ^{1}H-NMR (400 MHz, DMSO-d_6, 298 K): δ [ppm] = 4.14 (s, 1H), 4.39 (s, 3H), 7.34-7.41 (m, 2H), 7.43-7.48 (m, 2H), 10.88 (br. s, 1H). ^{13}C-NMR (126 MHz, DMSO-d_6,, 300 K): δ [ppm] = 60.7 (s, 1C), 80.6 (s, 1C), 83.3 (s, 1C), 116.8 (s, 1C), 119.3 (s, 2C), 132.7 (s, 2C), 138.5 (s, 1C), 169.0 (s, 1C), 179.2 (s, 1C), 184.2 (s, 1C), 187.7 (s, 1C). HRMS-ESI (m/z): calc. ($C_{14}H_{12}NO_3$ $[M+H]^+$): 228.0655; found: 228.0655.

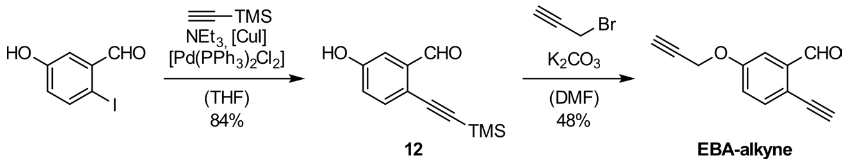

5-Hydroxy-2-((trimethylsilyl)ethynyl)benzaldehyde (12): A mixture of $Pd(PPh_3)_2Cl_2$ (35.4 mg, 50.4 µmol, 5 mol%) and CuI (19.2 mg, 101 µmol, 10 mol%) in dry THF (1.5 mL) was degassed by three freeze-pump-thaw cycles. To this, 5-hydroxy-2-iodobenzaldehyde (250 mg, 1.01 mmol, 1.00 equiv.) and dry NEt_3 (421 µL, 306 mg, 3.02 mmol, 3.00 equiv.) were added. After stirring for 15 min, a solution of trimethylsilylacetylene (158 µL, 109 mg, 1.11 mmol, 1.10 equiv.) in dry THF (160 µL) was added dropwise and stirring was continued for 2.5 h. The reaction mixture was filtered through a plug of silica, which was rinsed with EtOAc. The filtrate was concentrated *in vacuo* and the residual red oil was purified *via* column chromatography (pentane:EtOAc 9:1) to give **12** (184 mg, 844 µmol, 84%) as an orange solid. TLC (pentane:EtOAc 2:1): R_f = 0.53 [UV, $KMnO_4$]. ^{1}H-NMR (400 MHz, $CDCl_3$, 298 K): δ [ppm] = 0.26 (s, 9H), 6.36 (s, 1H), 7.08 (dd, J = 8.4, 2.7 Hz, 1H), 7.44 (d, J = 2.7 Hz, 1H), 7.49 (d, J = 8.4 Hz, 1H), 10.47 (s, 1H). ^{13}C-NMR (101 MHz, $CDCl_3$, 298 K): δ [ppm] = 0.0 (s, 3C), 100.1 (s, 1C), 100.7 (s, 1C), 113.0 (s, 1C), 119.7 (s, 1C), 122.0 (s, 1C), 135.5 (s, 1C), 137.6 (s, 1C), 156.6 (s, 1C), 192.6 (s, 1C). HRMS-ESI (m/z): calc. ($C_{12}H_{13}O_2Si$ $[M-H]^-$): 217.0690; found: 217.0690.

2-Ethynyl-5-(prop-2-yn-1-yloxy)benzaldehyde (EBA-alkyne): To a suspension of **12** (40.0 mg, 183 µmol, 1.00 equiv.) and K_2CO_3 (179 mg, 1.30 mmol, 7.07 equiv.) in dry DMF (600 µL), propargyl bromide (80 wt% in PhMe, 30.6 µL, 32.7 mg, 275 µmol, 1.50 equiv.) was added. After stirring for 24 h, water (6 mL) and EtOAc (15 mL) were added and layers were separated. The aqueous layer was extracted EtOAc (3 × 10 mL). The combined organic layers were washed with brine, dried over Na_2SO_4, filtered and concentrated *in vacuo*. Column chromatography (pentane:EtOAc 9:1) gave **EBA-alkyne** (16.3 mg, 88.5 µmol, 48%) as a slightly yellow solid. TLC (pentane:EtOAc 9:1): R_f = 0.29 [UV, $KMnO_4$]. ^{1}H-NMR (400 MHz, $CDCl_3$, 298 K): δ [ppm] = 2.55 (t, J = 2.4 Hz, 1H), 3.38 (s, 1H), 4.76 (d, J = 2.4 Hz, 2H), 7.18 (dd, J = 8.5, 2.8 Hz, 1H), 7.48 (d, J = 2.8 Hz, 1H), 7.56 (d, J = 8.5 Hz, 1H), 10.50 (s, 1H). ^{13}C-NMR (101 MHz, $CDCl_3$, 298 K): δ [ppm] = 56.2 (s, 1C), 76.6 (s, 1C), 77.6 (s, 1C), 79.1 (s, 1C), 83.2 (s, 1C), 111.5 (s, 1C), 119.0 (s, 1C), 122.1 (s, 1C), 135.5 (s, 1C), 138.1 (s, 1C), 158.1 (s, 1C), 191.2 (s, 1C). HRMS-APCI (m/z): calc. ($C_{12}H_9O_2$ $[M+H]^+$): 185.0597; found: 185.0598. The analytical data is in accordance with literature.[24]

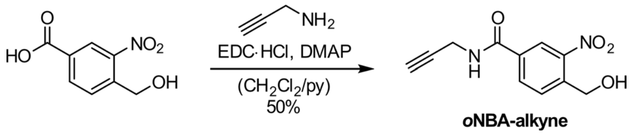

4-(Hydroxymethyl)-3-nitro-*N*-(prop-2-yn-1-yl)benzamide (*o*NBA-alkyne): Synthesis of ***o*NBA-alkyne** was carried out similar to literature procedure.[54] EDC·HCl (117 mg, 609 µmol, 1.20 equiv.), propargylamine (195 µL, 168 mg, 3.04 mmol, 6.00 equiv.) and DMAP (74.4 mg, 609 µmol, 1.20 equiv.) were added to a solution of 4-(hydroxymethyl)-3-nitrobenzoic acid (100 mg, 507 µmol, 1.00 equiv.) in dry CH_2Cl_2 (1.75 mL) and dry pyridine (350 µL). After stirring in the dark for 24 h, the reaction mixture was diluted with EtOAc (10 mL), washed with saturated aqueous $NaHCO_3$ (10 mL), aqueous HCl (1M, 3 × 15 mL), H_2O (10 mL), brine (10 mL), dried over Na_2SO_4, filtered and concentrated *in vacuo* to give ***o*NBA-alkyne** (59.0 mg, 252 µmol, 50%) as a brown solid. TLC (pentane:EtOAc 1:1): R_f = 0.48 [UV, $KMnO_4$]. ^{1}H-NMR (300 MHz, CD_3CN, 300 K): δ [ppm] = 2.49 (t, J = 2.6 Hz, 1H), 3.59 (br. s, 1H), 4.15 (dd, J = 5.6, 2.6 Hz, 2H), 4.96 (s, 2H), 7.54 (br. s, 1H), 7.94 (dt, J = 8.1, 1.0 Hz, 1H), 8.11 (dd, J = 8.1, 1.8 Hz, 1H), 8.45 (d, J = 8.1 Hz, 1H). ^{13}C-NMR (101 MHz, CD_3CN, 300 K): δ [ppm] = 29.8 (s, 1C), 61.6 (s, 1C), 72.1 (s, 1C), 81.0 (s, 1C),

124.4 (s, 1C), 129.7 (s, 1C), 132.9 (s, 1C), 134.6 (s, 1C), 142.4 (s, 1C), 148.0 (s, 1C), 165.5 (s, 1C). HRMS-ESI (m/z): calc. ($C_{11}H_9N_2O_4$ $[M-H]^-$): 233.0568; found: 233.0568. The analytical data is in accordance with literature.[55]

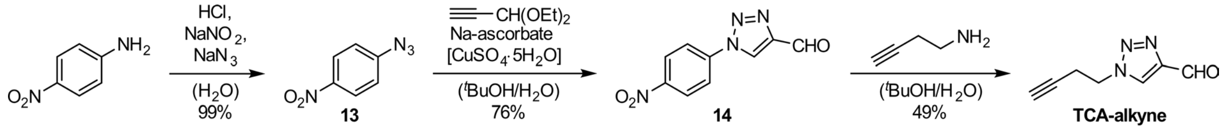

1-Azido-4-nitrobenzene (13): Synthesis of **13** was carried out similar to literature procedure.[56] Concentrated aqueous HCl (950 µL, 416 mg, 11.4 mmol, 2.10 equiv.) was added to 4-nitroaniline (750 mg, 5.43 mmol, 1.00 equiv.) suspended in H_2O (6 mL) at 0 °C. A solution of $NaNO_2$ (375 mg, 5.43 mmol, 1.00 equiv.) in H_2O (1 mL) was added dropwise, followed after 10 min by dropwise addition of a solution of NaN_3 (353 mg, 5.43 mmol, 1.00 equiv.) in H_2O (2 mL).* Within 30 min the reaction mixture became tough to stir due to precipitation and H_2O (3 mL) was added. Stirring was continued for 15 min at room temperature before addition of EtOAc (20 mL). The layers were separated, the aqueous layer was extracted with EtOAc (3 × 15 mL) and the combined organic layers were dried over Na_2SO_4 and filtered. Concentration *in vacuo* gave **13** (880 mg, 5.36 mmol, 99%) as a light brown crystalline solid. TLC (pentane:EtOAc 1:1): R_f = 0.74 [UV, $KMnO_4$]. ^{1}H-NMR (300 MHz, $CDCl_3$, 300 K): δ [ppm] = 7.10-7.17 (m, 2H), 8.21-8.28 (m, 2H). The analytical data is in accordance with literature.[56]

*CAUTION: Potential explosion hazard! Take precautions as described under general remarks.

1-(4-Nitrophenyl)-1*H*-1,2,3-triazole-4-carbaldehyde (14): Synthesis of **14** was carried out similar to literature procedure.[57] A suspension of **13** (500 mg, 3.05 mmol, 1.00 equiv.) in 50 vol% aqueous ^{t}BuOH (15 mL) was degassed by argon-sparging.* $CuSO_4 \cdot 5H_2O$ (152 mg, 609 µmol, 20 mol%), sodium ascorbate (241 mg, 1.22 mmol, 0.40 equiv.) and 3,3-diethoxyprop-1-yne (437 µL, 391 mg, 3.05 mmol, 1.00 equiv.) were added and the reaction mixture stirred at 70 °C for 17 h. CH_2Cl_2 (20 mL) and brine (25 mL) were added, the layers were separated and the aqueous layer was extracted with CH_2Cl_2 (3 × 25 mL). The combined organic layers were washed with brine, dried over Na_2SO_4, filtered and concentrated *in vacuo*. Column chromatography (CH_2Cl_2 → CH_2Cl_2:EtOAc 20:1) gave **14** (508 mg, 2.33 mmol, 76%) as light yellow solid. TLC (pentane:EtOAc 1:1): R_f = 0.55 [UV, $KMnO_4$]. ^{1}H-NMR (400 MHz, $CDCl_3$, 298 K): δ [ppm] = 8.00-8.07 (m, 2H), 8.44-8.51 (m, 2H), 8.64 (s, 1H), 10.25 (s, 1H). The analytical data is in accordance with literature.[26]

*CAUTION: Potential explosion hazard! Take precautions as described under general remarks.

1-(But-3-yn-1-yl)-1*H*-1,2,3-triazole-4-carbaldehyde (TCA-alkyne): A suspension of **14** (109 mg, 500 µmol, 1.00 equiv.) and 1-amino-3-butyne (45.0 µL, 38.0 mg, 550 µmol, 1.10 equiv.) in 50 vol% aqueous ^{t}BuOH (2.5 mL) was stirred at 70 °C for 24 h. After cooling to room temperature, Et_2O (25 mL) and aqueous HCl (6 M, 50 mL)* were added, the layers separated and the aqueous layer was extracted with EtOAc (3 × 20 mL). The combined organic layers were washed with brine, dried over Na_2SO_4, filtered and concentrated *in vacuo*. Column chromatography (pentane:EtOAc 4:1) gave **TCA-alkyne** (36.6 mg, 245 µmol, 49%) as a yellow solid. TLC (pentane:EtOAc 1:1): R_f = 0.44 [UV, $KMnO_4$]. ^{1}H-NMR (400 MHz, $CDCl_3$, 298 K): δ [ppm] = 2.11 (t, J = 2.6 Hz, 1H), 2.85 (td, J = 6.5, 2.6 Hz, 2H), 4.59 (t, J = 6.5 Hz, 2H), 8.26 (s, 1H), 10.16 (s, 1H). ^{13}C-NMR (101 MHz, $CDCl_3$, 298 K): δ [ppm] = 20.6 (s, 1C), 49.3 (s, 1C), 72.5 (s, 1C), 78.9 (s, 1C), 125.8 (s, 1C), 147.9 (s, 1C), 185.2 (s, 1C). HRMS-ESI (m/z): calc. ($C_7H_8N_3O$ $[M+H]^+$): 150.0662; found: 150.0663.

*aqueous HCl (2 M) was insufficient for imine hydrolysis.

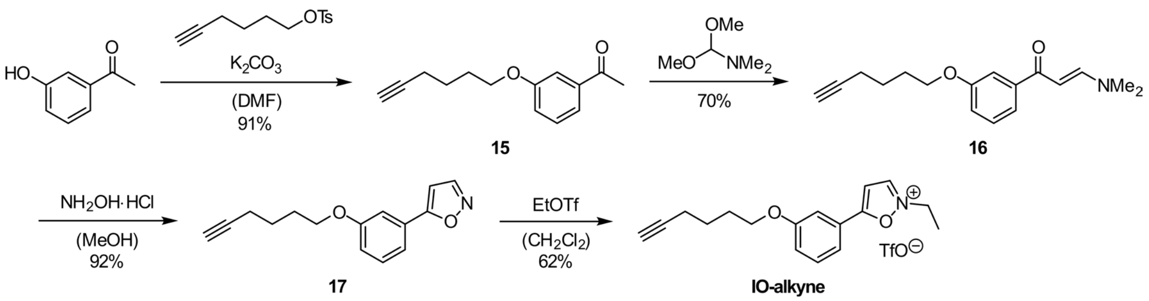

1-(3-(Hex-5-yn-1-yloxy)phenyl)ethan-1-one (15): Synthesis of **15** was carried out similar to literature procedure.[58] A mixture of 3-hydroxyacetophenone (1.36 g, 10.0 mmol, 1.00 equiv.), hex-5-yn-1-yl tosylate (2.52 g, 10.0 mmol, 1.00 equiv.) and K_2CO_3 (2.76 g, 20.0 mmol, 2.00 equiv.) in dry DMF (20 mL) was stirred at 90 °C for 17 h. After cooling to room temperature, EtOAc (50 mL) was added and the mixture was washed with brine, dried over Na_2SO_4, filtered and concentrated *in vacuo*. Column chromatography (pentane:Et_2O 5:1 → 3:1) gave **15** (1.96 g, 9.06 mmol, 91%) as a colourless oil. TLC (pentane:Et_2O 1:1): R_f = 0.53 [UV, $KMnO_4$]. ^{1}H-NMR (500 MHz, $CDCl_3$, 300 K): δ [ppm] = 1.70-1.77 (m, 2H), 1.90-1.96 (m, 2H), 1.97 (t, J = 2.6 Hz, 1H), 2.29 (td, J = 7.1, 2.6 Hz, 2H), 2.59 (s, 3H), 4.04 (t, J = 6.3 Hz, 2H), 7.10 (ddd, J = 6.8, 2.6, 0.9 Hz, 1H), 7.34-7.38 (apparent t, $J \approx$ 7.9 Hz, 1H), 7.47 (dd, J = 2.6, 1.6 Hz, 1H), 7.51-7.55 (apparent dt, J = 7.6 Hz, $J \approx$ 1.2 Hz, 1H). The analytical data is in accordance with literature.[58]

(*E*)-3-(Dimethylamino)-1-(3-(hex-5-yn-1-yloxy)phenyl)prop-2-en-1-one (16): Synthesis of **16** was carried out similar to literature procedure.[58] A solution of **15** (1.50 g, 6.94 mmol, 1.00 equiv.) in *N*,*N*-dimethylformamide dimethyl acetal (4.61 mL, 4.13 g, 34.7 mmol, 5.00 equiv.) was stirred at 120 °C for 43 h. After cooling to room temperature, EtOAc (30 mL) was added and the mixture was washed with brine, dried over Na_2SO_4, filtered and concentrated *in vacuo*. Column chromatography (hexane:EtOAc 1:5) gave **16** (1.32 g, 4.88 mmol, 70%) as a brown crystalline solid. TLC (pentane:EtOAc 1:5): R_f = 0.14 [UV, $KMnO_4$]. ^{1}H-NMR (300 MHz, $CDCl_3$, 300 K): δ [ppm] = 1.66-1.79 (m, 2H), 1.86-1.98 (m, 2H), 1.96 (t, J = 2.6 Hz, 1H), 2.28 (td, J = 7.0, 2.6 Hz, 2H), 2.95 (br. s, 3H), 3.13 (br. s, 3H), 4.04 (t, J = 6.3 Hz, 2H), 5.69 (d, J = 12.3 Hz, 1H), 6.98 (ddd, J = 8.1, 2.6, 1.1 Hz, 1H), 7.29 (apparent t, $J \approx$ 8.1 Hz, 1H), 7.41-7.48 (m, 2H), 7.79-7.87 (d, J = 12.3 Hz, 1H). The analytical data is in accordance with literature.[58]

5-(3-(Hex-5-yn-1-yloxy)phenyl)isoxazole (17): Synthesis of **17** was carried out similar to literature procedure.[58] A solution of **16** (750 mg, 2.76 mmol, 1.00 equiv.) and $NH_2OH \cdot HCl$ (211 mg, 3.04 mmol, 1.10 equiv.) in dry MeOH (6 mL) was stirred at 50 °C for 12.5 h. After cooling to room temperature, EtOAc (20 mL) and water (10 mL) were added and the layers were separated. The aqueous layer was extracted with EtOAc (3 × 15 mL) and the combined organic layers were washed with brine, dried over Na_2SO_4, filtered and concentrated *in vacuo*. Column chromatography (pentane:Et_2O 2:1) gave **17** (611 mg, 2.53 mmol, 92%) as a yellow oil. TLC (pentane:EtOAc 1:5): R_f = 0.71 [UV, $KMnO_4$]. ^{1}H-NMR (300 MHz, $CDCl_3$, 300 K): 1.68-1.82 (m, 2H), 1.89-2.02 (m, 2H), 1.98 (t, J = 2.6 Hz, 1H), 2.30 (td, J = 7.0, 2.6 Hz, 2H), 4.05 (t, J = 6.2 Hz, 2H), 6.50 (d, J = 1.9 Hz, 1H), 6.93-7.01 (m, 1H), 7.31-7.34 (m, 1H), 7.34-7.39 (m, 2H), 8.28 (d, J = 1.9 Hz, 1H). The analytical data is in accordance with literature.[58]

2-Ethyl-5-(3-(hex-5-yn-1-yloxy)phenyl)isoxazol-2-ium trifluoromethanesulfonate (Isx-alkyne): To **17** (200 mg, 829 µmol, 1.00 equiv.) in dry CH_2Cl_2 (2 mL) was added EtOTf (136 µL, 162 mg, 912 µmol, 1.10 equiv.) and the mixture was stirred for 23 h. After concentration *in vacuo*, the residue was redissolved in CH_2Cl_2 (500 µL) and added to Et_2O (45 mL). The Et_2O layer was decanted and the product layer was washed with Et_2O (3 × 40 mL). Concentration *in vacuo* gave **Isx-alkyne** (217 mg, 517 µmol, 62%) as a brown oil. ^{1}H-NMR (400 MHz, $CDCl_3$, 298 K): δ [ppm] = 1.68-1.77 (apparent p, $J \approx$ 7.3 Hz, 5H), 1.88-1.97 (m, 2H), 1.97 (t, J = 2.6 Hz, 1H), 2.28 (td, J = 7.0, 2.6 Hz, 2H), 4.06 (t, J = 6.2 Hz, 2H), 4.88 (q, J = 6.2 Hz, 2H), 7.12-7.20 (m, 1H), 7.34-7.38 (m, 1H), 7.42-7.50 (m, 3H), 9.69-9.75 (m, 1H). ^{13}C-NMR (101 MHz, $CDCl_3$, 298 K): δ [ppm] = 13.5 (s, 1C), 18.2 (s, 1C), 25.0 (s, 1C), 28.2 (s, 1C), 51.2 (s, 1C), 68.1 (s, 1C), 68.9 (s, 1C), 84.1 (s, 1C), 104.2 (s, 1C), 112.6 (s, 1C), 119.8 (s, 1C), 120.7 (q, 1C) 121.1 (s, 1C), 123.9 (s, 1C), 131.1 (s, 1C), 150.1 (s, 1C), 160.0 (s, 1C), 172.8 (s, 1C). ^{19}F-NMR (376 MHz, $CDCl_3$, 298 K): δ [ppm] = −78.5 (s). HRMS-ESI (m/z): calc. ($C_{17}H_{20}NO_2$ [M−OTf]$^+$): 270.1489; found: 270.1491.

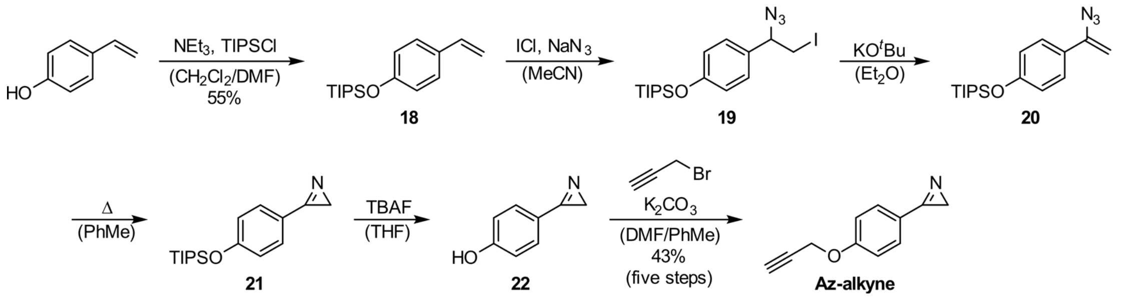

Tri*iso*propyl(4-vinylphenoxy)silane (18): To a solution of 4-vinylphenol (1.20 g, 10.0 mmol, 1.00 equiv.), in dry CH_2Cl_2 (36 mL) and dry DMF (4 mL), were added NEt_3 (3.48 mL, 2.53 g, 25.0 mmol, 2.50 equiv.) and TIPSCl (5.35 mL, 4.82 g, 25.0 mmol, 2.50 equiv.). After stirring for 16 h, the reaction mixture was washed with H_2O (2 × 75 mL), the organic layer was dried over Na_2SO_4, filtered and concentrated *in vacuo*. Column chromatography (pentane) gave **18** (1.52 g, 5.50 mmol, 55%) as a colourless oil. TLC (pentane): R_f = 0.29 [UV, $KMnO_4$]. ^{1}H-NMR (300 MHz, CD_3CN, 300 K): δ [ppm] = 1.06-1.13 (m, 18H), 1.20-1.34 (m, 3H), 5.12 (dd, J = 10.9, 1.1 Hz, 1H), 5.64 (dd, J = 17.6, 1.1 Hz, 1H), 6.68 (dd, J = 17.6, 10.9 Hz, 1H), 6.83-6.90 (m, 2H), 7.30-7.37 (m, 2H). ^{13}C-NMR (75.5 MHz, CD_3CN, 300 K): δ [ppm] = 13.5 (s, 3C), 18.3 (s, 6C), 112.4 (s, 1C), 120.9 (s, 2C), 128.4 (s, 2C), 131.9 (s, 1C), 137.2 (s, 1C), 156.9 (s, 1C). The analytical data is in accordance with literature.[59]

(4-(1-Azido-2-iodoethyl)phenoxy)tri*iso*propylsilane (19): Synthesis of **19** was carried out similar to literature procedure.[60] A solution of iodine monochloride (471 µL, 1.46 g, 9.00 mmol, 3.00 equiv.) in dry MeCN (4.5 mL) was added to a suspension of NaN_3 (975 mg, 15.0 mmol, 5.00 equiv.) in dry MeCN (7.5 mL) at −20 °C.* After 30 min, a solution of **18** (829 mg, 3.00 mmol, 1.00 equiv.) in dry CH_2Cl_2 (4.5 mL) was slowly added and the reaction mixture was stirred for 1.5 h before slow addition of saturated aqueous $Na_2S_2O_3$. The layers were separated, the aqueous layers extracted with EtOAc (4 × 20 mL) and the combined organic layers were dried over Na_2SO_4, giving **19** (1.39 g) as a yellow oil, which was used without further purification. TLC (pentane): R_f = 0.28 [UV, $KMnO_4$]. ^{1}H-NMR (300 MHz, $CDCl_3$, 300 K): δ [ppm] = 1.03-1.15 (m, 18H), 1.18-1.33 (m, 3H), 3.34-3.39 (m, 2H), 4.65 (t, J = 7.1 Hz, 1H), 6.86-6.93 (m, 2H), 7.13-7.20 (m, 2H). ^{13}C-NMR (75.5 MHz, $CDCl_3$, 300 K): δ [ppm] = 8.6 (s, 1C), 12.8 (s, 3C), 18.0 (s, 6C), 67.0 (s, 1C), 120.6 (s, 2C), 128.0 (s, 2C), 130.4 (s, 1C), 156.9 (s, 1C). HRMS-ESI (m/z): calc. ($C_{17}H_{28}IOSi$ $[M-N_3]^+$): 403.0949; found: 403.0951.

*CAUTION: Significant explosion hazard! Take precautions as described under general remarks.

(4-(1-Azidovinyl)phenoxy)tri*iso*propylsilane (20): Synthesis of **20** was carried out similar to literature procedure.[60] KO^{t}Bu (505 mg, 4.50 mmol, 1.59 equiv.) was added to a solution of crude **19** (1.26 g, ≤ 2.83 mmol, 1.00 equiv.) in dry Et_2O (11 mL) at 0 °C in the dark.* After stirring for 2 h at 0 °C and 6 h at room temperature, additional KO^{t}Bu (168 mg, 1.50 mmol, 0.53 equiv.) was added and stirring was continued for 16 h. Saturated aqueous NH_4Cl (10 mL) was added, layers were separated and the aqueous layer extracted with EtOAc (3 × 15 mL). The combined organic layers were washed with brine, dried over Na_2SO_4. The solvents were removed *in vacuo* to give 965 mg of yellow oil consisting of a mixture of **20** and starting material **19** in a molar ratio of 5.9:1, which was used without any further purification. TLC (pentane): R_f = 0.33 [UV, $KMnO_4$]. ^{1}H-NMR (500 MHz, $CDCl_3$, 300 K): δ [ppm] = 1.05-1.14 (m, 18H), 1.19-1.31 (m, 3H), 4.85 (d, J = 2.4 Hz, 1H), 5.31 (d, J = 2.4 Hz, 1H), 6.82-6.87 (m, 2H), 7.40-7.45 (m, 2H). ^{13}C-NMR (75.5 MHz, $CDCl_3$, 300 K): δ [ppm] = 12.8 (s, 3C), 18.0 (s, 6C), 96.3 (s, 1C), 119.9 (s, 2C), 127.0 (s, 2C), 127.3 (s, 1C), 144.9 (s, 1C), 157.2 (s, 1C). HRMS-ESI (m/z): calc. ($C_{17}H_{28}NOSi$ $[M-N_2+H]^+$): 290.1940; found: 290.1942.

*CAUTION: Potential explosion hazard! Take precautions as described under general remarks.

3-(4-((Tri*iso*propylsilyl)oxy)phenyl)-2*H*-azirine (21): Synthesis of **21** was carried out similar to literature procedure.[60] Crude **20** (960 mg, ≤ 2.82 mmol) from above was stirred in dry toluene (15 mL) at 110 °C for 1.5 h. After cooling to room temperature, the solvent was removed *in vacuo* to give crude **21** (913 mg) as an orange oil, which was used without any further purification. TLC (pentane): R_f = 0.40 [UV, $KMnO_4$]. ^{1}H-NMR (300 MHz, $CDCl_3$, 300 K): δ [ppm] = 1.03-1.15 (m, 18H), 1.18-1.33 (m, 3H), 1.73 (s, 2H), 6.86-6.93 (m, 2H), 7.13-7.20 (m, 2H). HRMS-ESI (m/z): calc. ($C_{17}H_{28}NOSi$ $[M+H]^+$): 290.1935; found: 290.1936. The analytical data is in accordance with literature.[60]

4-(2*H*-Azirin-3-yl)phenol (22): Synthesis of **22** was carried out similar to literature procedure.[60] A solution of TBAF (1.0 M in THF, 3.45 mL, 903 mg, 3.45 mmol, 2.00 equiv.) was added to crude **21** (500 mg, ≤ 1.73 mmol, 1.00 equiv.)

in dry THF (4 mL). After 1 h, saturated aqueous NH_4Cl was added and THF was removed *in vacuo*. The aqueous residue was extracted with EtOAc (3 × 10 mL), the combined organic layers were washed with brine, dried over Na_2SO_4 and filtered. Concentration *in vacuo* gave crude **22** (637 mg), which was used without any further purification. TLC (pentane:EtOAc 1:1): R_f = 0.43 [UV, $KMnO_4$].

3-(4-(Prop-2-yn-1-yloxy)phenyl)-2*H*-azirine (Az-alkyne): Synthesis of **Az-alkyne** was carried out similar to literature procedure.[60] K_2CO_3 (311 mg, 2.25 mmol, 1.30 equiv.) and propargyl bromide (80 wt% in PhMe, 251 µL, 267 mg, 2.25 mmol, 1.30 equiv.) were added to crude **22** (629 mg, ≤ 1.71 mmol, 1.00 equiv.) from above in dry DMF (10 mL). H_2O (40 mL) and EtOAc (30 mL) were added after 16 h and layers were separated. The aqueous layer was extracted with EtOAc (3 × 30 mL) and the combined organic layers were washed with brine, dried over Na_2SO_4, filtered and concentrated *in vacuo*. Column chromatography (pentane:Et_2O 4:1) gave **Az-alkyne** (124 mg, 725 µmol, 43% over five steps) as a white solid.* TLC (pentane:EtOAc 1:1): R_f = 0.63 [UV, $KMnO_4$]. ^{1}H-NMR (500 MHz, $CDCl_3$, 300 K): *δ* [ppm] = 1.75 (s, 2H), 2.57 (t, *J* = 2.4 Hz, 1H), 4.79 (d, *J* = 2.4 Hz, 2H), 7.12-7.17 (m, 2H), 7.85-7.90 (m, 2H). ^{13}C-NMR (101 MHz, $CDCl_3$, 298 K): *δ* [ppm] = 19.6 (s, 1C), 56.1 (s, 1C), 76.4 (s, 1C), 77.8 (s, 1C), 115.6 (s, 2C), 119.1 (s, 1C), 131.6 (s, 2C), 161.2 (s, 1C), 164.6 (s, 1C). HRMS-ESI (m/z): calc. ($C_{11}H_{10}NO$ $[M+H]^+$): 172.0757; found: 172.0754. The analytical data is in accordance with literature.[60]

*Bulk compound turned brown after standing at room temperature with no decomposition detected by ^{1}H-NMR.

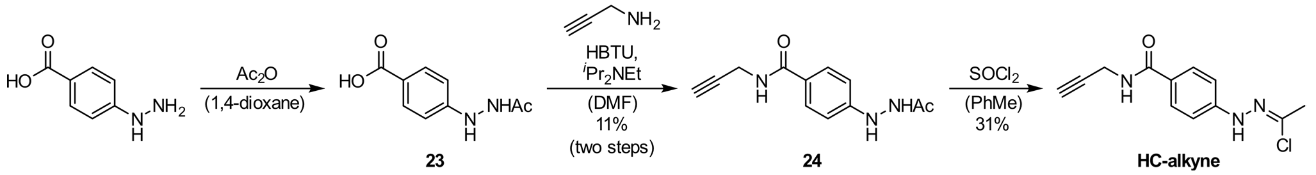

4-(2-Acetylhydrazineyl)benzoic acid (23): To a solution of 4-hydrazineylbenzoic acid (1.98 g, 13.0 mmol, 1.00 equiv.) in dry 1,4-dioxane (15 mL), a solution of acetic anhydride (2.46 mL, 2.65 g, 26.0 mmol, 2.00 equiv.) in dry 1,4-dioxane (10 mL) was added dropwise over 1.5 h. After stirring overnight, EtOAc (30 mL) was added and the mixture was extracted with aqueous NaOH (1 M, 4 × 25 mL). The combined aqueous layers were acidified with concentrated aqueous HCl and then extracted with EtOAc (4 × 20 mL). The combined organic layers were washed with brine, dried over Na_2SO_4, filtered and concentrated *in vacuo* to give crude **23** (2.03 g) as a light brown solid which was used without any further purification. ^{1}H-NMR (300 MHz, DMSO-d_6, 300 K): *δ* [ppm] = 1.91 (s, 3H), 6.65-6.72 (m, 2H), 7.69-7.76 (m, 2H), 8.31 (d, *J* = 1.8 Hz, 1H), 9.72 (d, *J* = 1.8 Hz, 1H), 12.24 (br. s, 1H). ^{13}C-NMR (75.5 MHz, DMSO-d_6, 300 K): *δ* [ppm] = 20.6 (s, 1C), 110.8 (s, 2C), 119.8 (s, 1C), 130.9 (s, 2C), 153.1 (s, 1C), 167.3 (s, 1C), 169.1 (s, 1C). HRMS-ESI (m/z): calc. ($C_9H_9N_2O_3$ $[M-H]^-$): 193.0619; found: 193.0615.

4-(2-Acetylhydrazineyl)-*N*-(prop-2-yn-1-yl)benzamide (24): To a solution of crude **23** (570 mg, 2.94 mmol, 1.00 equiv.) in dry DMF (3.75 mL) were added propargylamine (207 µL, 178 mg, 3.23 mmol, 1.10 equiv.) and HBTU (1.17 g, 3.08 mmol, 1.05 equiv.) at 0 °C. The mixture was stirred for 10 min before addition of ^{i}Pr$_2$NEt (684 µL, 507 mg, 3.92 mmol, 1.34 equiv.) and stirring was continued overnight. EtOAc (200 mL) was added, the mixture was washed with aqueous HCl (1 M, 2 × 100 mL), quarter-saturated aqueous Na_2CO_3 (2 × 100 mL) and brine. After drying over Na_2SO_4 and filtering, the crude product was dry-loaded onto kieselgur and purified *via* column chromatography (EtOAc:acetone 1:2) to give **24** (87.1 mg, 377 µmol, 11% over two steps) as a light brown solid. TLC (CH_2Cl_2:MeOH 9:1): R_f = 0.16 [UV, $KMnO_4$]. ^{1}H-NMR (500 MHz, DMSO-d_6, 300 K): *δ* [ppm] = 1.91 (s, 3H), 3.06 (t, *J* = 2.5 Hz, 1H), 4.00 (dd, *J* = 5.4, 2.5 Hz, 2H), 6.65-6.70 (m, 2H), 7.65-7.69 (m, 2H), 8.13 (br. s, 1H), 8.54 (t, *J* = 5.4 Hz, 1H), 9.67 (d, *J* = 2.0 Hz, 1H). ^{13}C-NMR (101 MHz, DMSO-d_6, 298 K): *δ* [ppm] = 20.6 (s, 1C), 28.3 (s, 1C), 72.5 (s, 1C), 81.8 (s, 1C), 110.8 (s, 2C), 123.3 (s, 1C), 128.5 (s, 2C), 151.9 (s, 1C), 165.7 (s, 1C), 169.1 (s, 1C). HRMS-ESI (m/z): calc. ($C_{12}H_{14}N_3O_2$ $[M+H]^+$): 232.1081; found: 232.1083.

(*Z*)-*N*-(4-(Prop-2-yn-1-ylcarbamoyl)phenyl)acetohydrazonoyl chloride (HC-alkyne): To a solution of **24** (50.0 mg, 216 µmol, 1.00 equiv.) in dry toluene (1 mL), was dropwise added a solution of $SOCl_2$ (31.4 µL, 51.4 mg, 432 µmol, 2.00 equiv.) in dry toluene (200 µL). After stirring at 70 °C for 3.5 h and cooling to room temperature, ice-cold water (1.5 mL) and CH_2Cl_2 (3 mL) were added. The layers were separated and the aqueous layer was extracted with CH_2Cl_2 (2 × 10 mL). The combined organic layers were washed with aqueous $NaHCO_3$ (5 wt%, 2 × 10 mL) and brine, dried over Na_2SO_4, filtered and concentrated *in vacuo*. Column chromatography

(pentane:EtOAc, 3:1) gave **HC-alkyne** (16.9 mg, 67.7 µmol, 31%) as a yellow solid. TLC (pentane:EtOAc 1:1): R_f = 0.47 [UV, $KMnO_4$]. ^{1}H-NMR (500 MHz, $CDCl_3$, 300 K): δ [ppm] = 2.27 (t, J = 2.5 Hz, 1H), 2.41 (s, 3H), 4.24 (dd, J = 5.0, 2.5 Hz, 2H), 6.18 (br. s, 1H), 7.03-7.07 (m, 2H), 7.69-7.74 (m, 2H), 7.78 (br. s, 1H). ^{13}C-NMR (75.5 MHz, $CDCl_3$, 300 K): δ [ppm] = 25.6 (s, 1C), 29.9 (s, 1C), 71.9 (s, 1C), 79.9 (s, 1C), 112.6 (s, 2C), 125.4 (s, 1C), 125.6 (s, 1C), 128.9 (s, 2C), 146.7 (s, 1C), 166.9 (s, 1C). HRMS-ESI (m/z): calc. ($C_{12}H_{13}ClN_3O$ $[M+H]^+$): 250.0742; found: 250.0743.

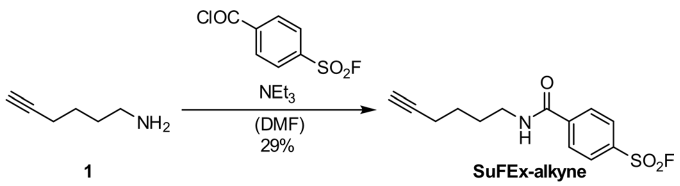

4-(Hex-5-yn-1-ylcarbamoyl)benzenesulfonyl fluoride (SuFEx-alkyne): A solution of 4-(fluorosulfonyl)benzoyl chloride (200 mg, 898 µmol, 1.00 equiv.) in dry DMF (1 mL) was added dropwise to a solution of **1** (87.3 mg, 898 µmol, 1.00 equiv.) and dry NEt_3 (250 µL, 182 mg, 1.80 mmol, 2.00 equiv.) in dry DMF (1 mL) at 0 °C. After 5.5 h, EtOAc (5 mL) and brine (10 mL) were added, layers were separated and the organic layer was washed with aqueous HCl (1 M, 10 mL) and aqueous NaOH (1 M, 10 mL). The organic layer was dried over Na_2SO_4, filtered and concentrated *in vacuo*. Column chromatography (pentane:EtOAc 4:1 → 3:1) gave **SuFEx-alkyne** (74.7 mg, 264 µmol, 29%) as a white solid. TLC (pentane:EtOAc 1:2): R_f = 0.65 [UV, $KMnO_4$]. ^{1}H-NMR (300 MHz, $CDCl_3$, 300 K): δ [ppm] = 1.55-1.69 (m, 2H), 1.71-1.84 (m, 2H), 1.98 (t, J = 2.7 Hz, 1H), 2.26 (td, J = 6.8, 2.7 Hz, 2H), 3.46-3.56 (m, 2H), 6.42 (br. s, 1H), 7.95-8.02 (m, 2H), 8.03-8.10 (m, 2H). ^{13}C-NMR (75.5 MHz, $CDCl_3$, 300 K): δ [ppm] = 18.2 (s, 1C), 25.8 (s, 1C), 28.6 (s, 1C), 40.1 (s, 1C), 69.1 (s, 1C), 84.0 (s, 1C), 128.3 (s, 2C), 128.9 (s, 2C), 135.5 (d, J = 25.4 Hz, 1C), 141.4 (s, 1C), 165.4 (s, 1C). ^{19}F-NMR (377 MHz, $CDCl_3$, 298 K): δ [ppm] = 66.0 (s, 1F). HRMS-ESI (m/z): calc. ($C_{13}H_{15}FNO_3S$ $[M+H]^+$): 284.0751; found: 284.0753.

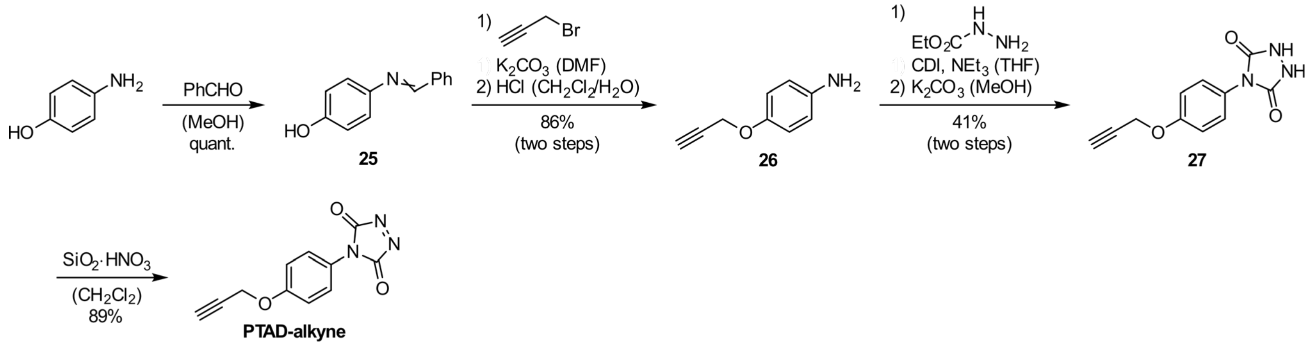

4-(Benzylideneamino)phenol (25): Synthesis of **25** was carried out similar to literature procedure.[61] Benzaldehyde (1.59 g, 15.0 mmol, 1.00 equiv.) was added to a solution of 4-aminophenol (1.64 g, 15.0 mmol, 1.00 equiv.) in MeOH (50 mL). After stirring for 1.5 h, the solvent was removed, giving **25** (2.96 g, 15.0 mmol, quant.) as a pale-yellow solid which was used without any further purification. ^{1}H-NMR (500 MHz, DMSO-d_6, 288 K): δ [ppm] = 6.77-6.84 (m, 2H), 7.16-7.24 (m, 2H), 7.45-7.53 (m, 3H), 7.86-7.93 (m, 2H), 8.61 (s, 1H), 9.56 (s, 1H). The analytical data is in accordance with literature.[62]

4-(Prop-2-yn-1-yloxy)aniline (26): To a suspension of **25** (2.96 g, 15.0 mmol, 1.00 equiv.) and K_2CO_3 (6.22 g, 45.0 mmol, 3.00 equiv.) in dry DMF (50 mL), propargyl bromide (80 wt% in PhMe, 2.01 mL, 2.14 g, 18.0 mmol, 1.20 equiv.) was added slowly. After stirring for 17 h, H_2O (50 mL) was added and the reaction mixture was extracted with EtOAc (3 × 50 mL). The combined organic layers were washed with brine (30 mL), dried over Na_2SO_4 and filtered. The solvent was removed *in vacuo*, the resulting residue was dissolved in CH_2Cl_2 (50 mL) and aqueous HCl (1 M, 150 mL) was added while stirring vigorously. After 2.5 h, the layers were separated, the aqueous layer was neutralised with saturated aqueous $NaHCO_3$ and extracted with CH_2Cl_2 (4 × 50 mL). The combined organic layers were dried over Na_2SO_4, filtered and concentrated *in vacuo*, giving **26** (1.90 g, 12.9 mmol, 86%) as a green-brown oil, which was used without any further purification. TLC (pentane:EtOAc 1:1): R_f = 0.71 [UV, $KMnO_4$]. ^{1}H-NMR (500 MHz, $CDCl_3$, 300 K): δ [ppm] = 2.49 (t, J = 2.4 Hz, 1H), 3.45 (br. s, 2H), 4.60 (d, J = 2.4 Hz, 2H),

6.62-6.67 (m, 2H), 6.80-6.85 (m, 2H). HRMS-ESI (m/z): calc. ($C_9H_{10}NO$ $[M+H]^+$): 148.0757; found: 148.0757. The analytical data is in accordance with literature.[63]

4-(4-(Prop-2-yn-1-yloxy)phenyl)-1,2,4-triazolidine-3,5-dione (27): Synthesis of **27** was carried out similar to literature procedure.[33] 1,1'-Carbonyldiimidazole (1.10 g, 6.79 mmol, 1.00 equiv.) was added to a solution of ethyl carbazate (707 mg, 6.79 mmol, 1.00 equiv.) in dry THF (30 mL), followed by **26** (1.00 g, 6.79 mmol, 1.00 equiv.) and dry NEt_3 (1.89 mL, 1.38 g, 13.6 mmol, 2.00 equiv.) after 1.5 h. After stirring for 21.5 h at 45 °C, EtOAc (15 mL) and aqueous HCl (3.3 M, 15 mL) were added and layers separated. The organic layer was washed with aqueous HCl (3.3 M, 20 mL) and H_2O (20 mL) and the combined aqueous layers were extracted with EtOAc (3 × 20 mL). The combined organic layers were dried over Na_2SO_4, filtered and concentrated *in vacuo*. MeOH (25 mL) and K_2CO_3 (2.65 g, 19.2 mmol, 2.82 equiv.) were added to the resulting residue and the mixture was heated to reflux for 4 h. After adjusting the pH to 2 with concentrated aqueous HCl, the mixture was concentrated by a stream of N_2 followed by evaporation *in vacuo*. Column chromatography (CH_2Cl_2:MeOH 9:1) gave **27** (645 mg, 2.79 mmol, 41%) as a white solid. TLC (CH_2Cl_2:MeOH 9:1): R_f = 0.21 [UV, $KMnO_4$]. ^{1}H-NMR (500 MHz, CD_3CN, 300 K): δ [ppm] = 2.83 (t, J = 2.4 Hz, 1H), 4.77 (d, J = 2.4 Hz, 2H), 7.05-7.10 (m, 2H), 7.33-7.39 (m, 2H), 7.81 (br. s, 2H). HRMS-ESI (m/z): calc. ($C_{11}H_{10}N_3O_3$ $[M+H]^+$): 232.0717; found: 232.0717. The analytical data is in accordance with literature.[64]

4-(4-(Prop-2-yn-1-yloxy)phenyl)-3*H*-1,2,4-triazole-3,5(4*H*)-dione (PTAD-alkyne): Concentrated aqueous HNO_3 (4 mL) was dropped onto silica (2 g) and the resulting slurry was dried by N_2-stream after stirring for 10 min. The resulting $SiO_2{\cdot}HNO_3$ (270 mg) was added to **27** (100 mg, 433 µmol) in CH_2Cl_2 (4.3 mL) and the suspension was stirred for 15 min in the dark. After filtering the reaction mixture, the filtrate was concentrated *in vacuo*, giving **PTAD-alkyne** (87.9 mg, 384 µmol, 89%) as a dark red solid. ^{1}H-NMR (500 MHz, CD_3CN, 300 K): δ [ppm] = 2.85 (t, J = 2.4 Hz, 1H), 4.81 (d, J = 2.4 Hz, 2H), 7.14-7.19 (m, 2H), 7.33-7.38 (m, 2H). ^{13}C-NMR (101 MHz, CD_3CN, 300 K): δ [ppm] = 57.0 (s, 1C), 77.3 (s,1C), 79.1 (s, 1C), 117.0 (s, 2C), 124.0 (s, 1C), 127.6 (s, 2C), 159.1 (s, 1C), 159.7 (s, 2C). The analytical data is in accordance with literature.[65]

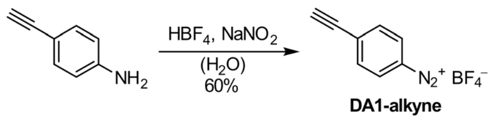

4-Ethynylbenzenediazonium tetrafluoroborate (DA1-alkyne): Synthesis of **DA1-alkyne** was carried out similar to literature procedure.[66] 4-Ethynylaniline (300 mg, 2.56 mmol, 1.00 equiv.) was suspended in H_2O (1.1 mL) and aqueous HBF_4 (48 wt%, 642 µL, 432 mg, 4.92 mmol, 1.92 equiv.). To this, a solution of $NaNO_2$ (177 mg, 2.56 mmol, 1.00 equiv.) in H_2O (400 µL) was added dropwise at 0 °C.* After stirring for 45 min, the reaction mixture was filtered and the filter residue was washed with H_2O, then dissolved in minimal amounts acetone and re-precipitated in cold Et_2O to give **DA1-alkyne** (334 mg, 1.55 mmol, 60%) as a red solid. ^{1}H-NMR (300 MHz, CD_3CN, 300 K): δ [ppm] = 4.22 (s, 1H), 7.92-7.99 (apparent dt, $J \approx$ 9.1, 2.0 Hz, 2H), 8.42-8.51 (apparent dt, $J \approx$ 9.1, 2.1 Hz, 2H). ^{13}C-NMR (75.5 MHz, CD_3CN, 300 K): δ [ppm] = 81.5 (s, 1C), 90.9, (s, 1C), 114.7 (s, 1C), 133.6 (s, 2C), 135.8 (s, 2C), 136.7 (s, 1C). ^{19}F-NMR (376 MHz, DMSO-d$_6$, 298 K): δ [ppm] = −148.3 (s, $^{11}BF_4$), −148.3 (s, $^{10}BF_4$). HRMS-ESI (m/z): calc. ($C_8H_5N_2$ $[M{-}BF_4]^+$): 129.0447; found: 129.0447. The analytical data is in accordance with literature.[67]

*CAUTION: Potential explosion hazard! Take precautions as described under general remarks.

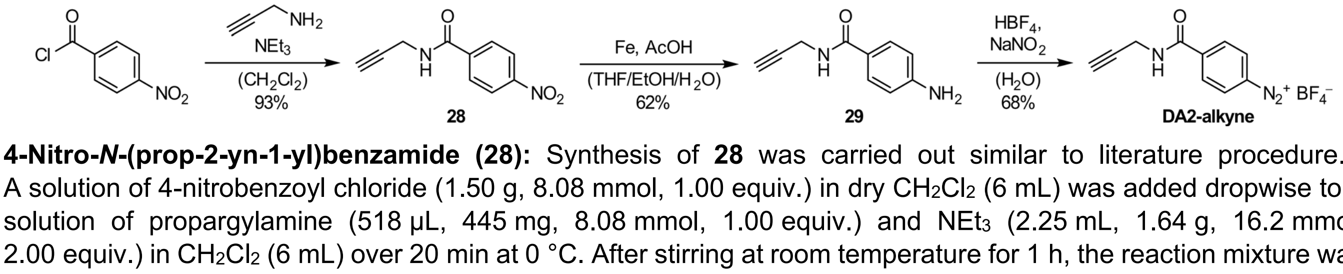

4-Nitro-*N*-(prop-2-yn-1-yl)benzamide (28): Synthesis of **28** was carried out similar to literature procedure.[68] A solution of 4-nitrobenzoyl chloride (1.50 g, 8.08 mmol, 1.00 equiv.) in dry CH_2Cl_2 (6 mL) was added dropwise to a solution of propargylamine (518 µL, 445 mg, 8.08 mmol, 1.00 equiv.) and NEt_3 (2.25 mL, 1.64 g, 16.2 mmol, 2.00 equiv.) in CH_2Cl_2 (6 mL) over 20 min at 0 °C. After stirring at room temperature for 1 h, the reaction mixture was washed with H_2O (10 mL), aqueous NaOH (1 M, 20 mL), aqueous HCl (1 M, 20 mL) and brine (15 mL). Concentration of the organic layer *in vacuo* gave **28** (1.54 g, 7.54 mmol, 93%) as a light-brown solid which was used without any

further purification. TLC (pentane:EtOAc 1:1): R_f = 0.47 [UV, $KMnO_4$]. ^{1}H-NMR (400 MHz, $CDCl_3$, 298 K): δ [ppm] = 2.33 (t, J = 2.5 Hz, 1H), 4.29 (dd, J = 5.2, 2.5 Hz, 2H), 6.37 (br. s, 1H), 7.93-7.99 (m, 2H), 8.28-8.34 (m, 2H). The analytical data is in accordance with literature.[69]

4-Amino-*N*-(prop-2-yn-1-yl)benzamide (29): Synthesis of **29** was carried out similar to literature procedure.[68] Iron powder (2.05 g, 36.7 mmol, 5.00 equiv.) and glacial AcOH (2.10 mL, 2.21 g, 36.7 mmol, 5.00 equiv.) were added to a solution of **28** (1.50 g, 7.35 mmol, 1.00 equiv.) in EtOH (15 mL), THF (15 mL) and H_2O (2 mL). After stirring at 80 °C for 22.5 h, the organic solvent was removed *in vacuo* and the aqueous residue extracted with EtOAc (3 × 70 mL). The combined organic layers were washed with water and brine, dried over Na_2SO_4, filtered and concentrated *in vacuo*, giving **29** (796 mg, 4.57 mmol, 62%) as a light brown solid which was used without any further purification. TLC (pentane:EtOAc 1:1): R_f = 0.18 [UV, $KMnO_4$]. ^{1}H-NMR (500 MHz, $CDCl_3$, 287 K): δ [ppm] = 2.27 (t, J = 2.5 Hz, 1H), 4.00 (br. s, 2H), 4.23 (dd, J = 5.2, 2.5 Hz, 2H), 6.14 (br. s, 1H), 6.64-6.68 (m, 2H), 7.59-7.65 (m, 2H). The analytical data is in accordance with literature.[68]

4-(Prop-2-yn-1-ylcarbamoyl)benzenediazonium tetrafluoroborate (DA2-alkyne): 29 (200 mg, 1.15 mmol, 1.00 equiv.) was suspended in H_2O (700 µL) and aqueous HBF_4 (48 wt%, 300 µL, 101 mg, 2.30 mmol, 2.00 equiv.). To this, a solution of $NaNO_2$ (79.2 mg, 1.15 mmol, 1.00 equiv.) in H_2O (400 µL) was added dropwise at 0 °C.* After stirring for 2 h, the reaction mixture was filtered and the filter residue was dissolved in minimal amounts acetone and reprecipitated in cold Et_2O to give **DA2-alkyne** (214 mg, 784 µmol, 68%) as a red solid. ^{1}H-NMR (300 MHz, CD_3CN, 300 K): δ [ppm] = 2.53 (t, J = 2.6 Hz, 1H), 4.16 (dd, J = 5.6, 2.6 Hz, 2H), 7.29 (br. s, 1H), 8.17-8.24 (m, 2H), 8.54-8.61 (m, 2H). ^{13}C-NMR (75.5 MHz, CD_3CN, 300 K): δ [ppm] = 30.2 (s, 1C), 72.6, (s, 1C), 80.3 (s, 1C), 117.9 (s, 1C), 131.3 (s, 2C), 133.9 (s, 2C), 146.4 (s, 1C), 164.7 (s, 1C). ^{19}F-NMR (376 MHz, DMSO-d_6, 300 K): δ [ppm] = −148.3 (s, $^{11}BF_4$), −148.2 (s, $^{10}BF_4$). HRMS-ESI (m/z): calc. ($C_{10}H_8N_3O$ $[M-BF_4]^+$): 186.0662; found: 186.0662.

*CAUTION: Potential explosion hazard! Take precautions as described under general remarks.

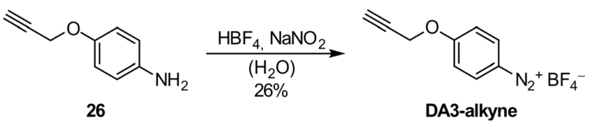

26 DA3-alkyne

4-(Prop-2-yn-1-yloxy)benzenediazonium tetrafluoroborate (DA3-alkyne): To a mixture of **26** (125 mg, 849 µmol, 1.00 equiv.) in H_2O (365 µL), aqueous HBF_4 (48 wt%, 444 µL, 298 mg, 4.00 equiv.), MeCN (400 µL) and CH_2Cl_2 (300 µL), a solution of $NaNO_2$ (58.6 mg, 849 µmol, 1.00 equiv.) in 140 µL H_2O was added dropwise at 0 °C.* After stirring for 2.5 h, the reaction mixture was filtered and the filter residue was washed with H_2O, then dissolved in minimal amounts acetone and re-precipitated in cold Et_2O to give **DA3-alkyne** (54.2 mg, 220 µmol, 26%) as a red solid. ^{1}H-NMR (300 MHz, CD_3CN, 300 K): δ [ppm] = 3.02 (t, J = 2.4 Hz, 1H), 5.03 (d, J = 2.4 Hz, 2H), 7.37-7.44 (m, 2H), 8.38-8.46 (m, 2H). ^{13}C-NMR (75.5 MHz, CD_3CN, 300 K): δ [ppm] = 58.9 (s, 1C), 77.1 (s, 1C), 79.2 (s, 1C), 104.1 (s, 1C), 119.5 (s, 2C), 136.7 (s, 2C), 169.0 (s, 1C). ^{19}F-NMR (376 MHz, CD_3CN, 298 K): δ [ppm] = −151.6 (s, $^{11}BF_4$), −151.7 (s, $^{10}BF_4$). HRMS-ESI (m/z): calc. ($C_9H_7N_2O$ $[M-BF_4]^+$): 159.0553; found: 159.0553.

*CAUTION: Potential explosion hazard! Take precautions as described under general remarks.

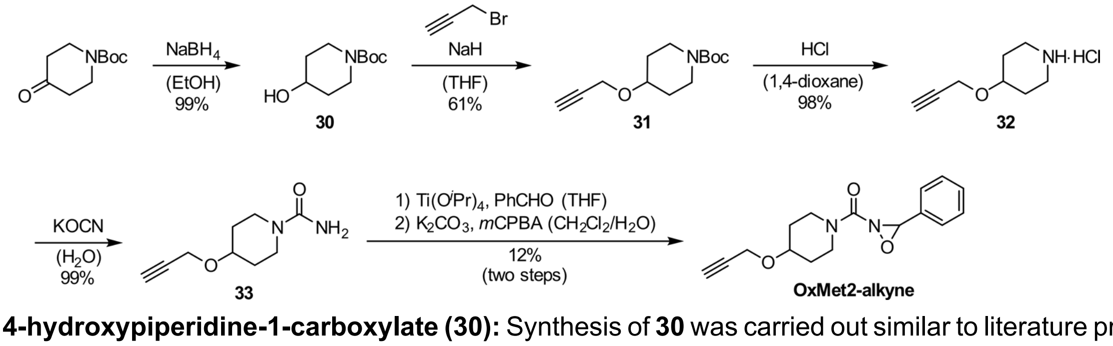

30 31 32 33 OxMet2-alkyne

***tert*-Butyl 4-hydroxypiperidine-1-carboxylate (30):** Synthesis of **30** was carried out similar to literature procedure.[70] $NaBH_4$ (600 mg, 15.9 mmol, 1.59 equiv.) was added in portions to *tert*-butyl 4-oxopiperidine-1-carboxylate (1.99 g, 9.99 mmol, 1.00 equiv.) in EtOH (10 mL) at 0 °C. The reaction mixture was allowed to warm to room temperature while stirring for 4 h. Saturated aqueous NH_4Cl (20 mL) was added at 0 °C and EtOH was removed *in vacuo*. The

aqueous residue was extracted with EtOAc (3 × 25 mL), the combined organic layers were dried over Na_2SO_4, filtered and concentrated *in vacuo*. Column chromatography (CH_2Cl_2/MeOH 99:1 + 0.1 vol% NEt_3 → 95:5 + 0.1 vol% NEt_3) gave **30** (1.98 g, 9.84 mmol, 99%) as a yellow oil. TLC (pentane:EtOAc 7:3): R_f = 0.42 [$KMnO_4$]. ^{1}H-NMR (500 MHz, $CDCl_3$, 300 K): δ [ppm] = 1.41-1.50 (m, 2H), 1.45 (s, 9H), 1.81-1.88 (m, 2H), 3.02 (ddd, J = 13.3, 9.8, 3.3 Hz, 2H), 3.79-3.88 (m, 3H). ^{13}C-NMR (75.5 MHz, $CDCl_3$, 300 K): δ [ppm] = 28.6 (s, 3C), 34.4 (s, 2C), 41.4 (s, 2C), 68.0 (s, 1C), 79.7 (s, 1C), 155.0 (s, 1C). HRMS-ESI (m/z): calc. ($C_{10}H_{20}NO_3$ $[M+H]^+$): 202.1438; found: 202.1437. The analytical data is in accordance with literature.[70]

***tert*-Butyl 4-(prop-2-yn-1-yloxy)piperidine-1-carboxylate (31):** Synthesis of **31** was carried out similar to literature procedure.[65] NaH (60 wt% in mineral oil, 477 mg, 19.9 mmol, 2.00 equiv.) was added to **30** (2.00 g, 9.94 mmol, 1.00 equiv.) in THF (33 mL). After 30 min, propargyl bromide (80 wt% in toluene, 3.19 g, 26.8 mmol, 2.70 equiv.) was added and stirring was continued for 3 h. H_2O (20 mL) was added at 0 °C, layers were separated and the aqueous layer was extracted with EtOAc (3 × 30 mL). The combined organic layers were washed with brine, dried over Na_2SO_4, filtered and concentrated *in vacuo*. Column chromatography (pentane:EtOAc 7:3) gave **31** (1.46 g, 6.10 mmol, 61%). TLC (pentane:EtOAc 7:3): R_f = 0.43 [$KMnO_4$]. ^{1}H-NMR (500 MHz, $CDCl_3$, 300 K): δ [ppm] = 1.45 (s, 9H), 1.52 (dtd, J = 12.9, 8.7, 4.6 Hz, 2H), 1.81-1.89 (m, 2H), 2.41 (t, J = 2.4 Hz, 1H), 3.09 (ddd, J = 13.5, 9.1, 3.5 Hz, 2H), 3.70 (tt, J = 8.2, 3.8 Hz, 1H), 3.74-3.81 (m, 2H), 4.19 (d, 2H, J = 2.4 Hz). ^{13}C-NMR (75.5 MHz, $CDCl_3$, 300 K): δ [ppm] = 28.6 (s, 3C), 30.9 (s, 2C), 41.3 (s, 2C), 55.4 (s, 1C), 73.9 (s, 1C), 74.2 (s, 1C), 79.7 (s, 1C), 80.2 (s, 1C), 155.0 (s, 1C). The analytical data is in accordance with literature.[71]

4-(Prop-2-yn-1-yloxy)piperidine hydrochloride (32): Synthesis of **32** was carried out similar to literature procedure.[72] HCl (4.0 M in 1,4-dioxane, 6.00 mL, 875 mg, 24.0 mmol, 5.74 equiv.) was added dropwise to a solution of **31** (1.00 g, 4.18 mmol, 1.00 equiv.) in methanol (12 mL) at 0 °C. The reaction mixture was stirred for 15 h, during which it was allowed to warm to room temperature, and then concentrated with a stream of compressed air. The residue was washed with Et_2O (5 × 5 mL) to give **32** (720 mg, 4.10 mmol, 98%) as a white solid. ^{1}H-NMR (300 MHz, CD_3OD, 300 K): δ [ppm] = 1.84-1.97 (m, 2H), 1.97-2.10 (m, 2H), 2.88 (t, J = 2.4 Hz, 1H), 3.08-3.19 (m, 2H), 3.24-3.37 (m, 2H)*, 3.91 (tt, J = 6.4, 3.3 Hz, 1H), 4.25 (d, J = 2.4 Hz, 2H) .^{13}C-NMR (75.5 MHz, CD_3OD, 300 K): δ [ppm] = 28.5 (s,2C), 41.9 (s, 2C), 56.6 (s, 1C), 70.7 (s, 1C), 75.9 (s, 1C), 80.7 (s, 1C). HRMS-ESI (m/z): calc. ($C_8H_{14}NO$ $[M-Cl]^+$): 140.1070; found: 140.1070.

*overlap with solvent signal.

4-(Prop-2-yn-1-yloxy)piperidine-1-carboxamide (33): KOCN (1.33 g, 16.4 mmol, 4.00 equiv.) was added to a solution of **32** (720 mg, 4.10 mmol, 1.00 equiv.) in H_2O (4.1 mL). After stirring for 17 h at 60 °C, the solvent was removed *in vacuo* and a mixture of $CHCl_3$ and ^{i}PrOH (85:15, 20 mL) was added to the resulting residue. The suspension was filtered, and the solid was washed with a mixture of $CHCl_3$ and ^{i}PrOH (85:15, 4 × 20 mL). The combined filtrates were dried over Na_2SO_4, filtered and concentrated *in vacuo*, giving **33** (740 mg, 4.06 mmol, 99%) as a brown solid. TLC (EtOAc): R_f = 0.11 [$KMnO_4$]. ^{1}H-NMR (300 MHz, CD_3OD, 300 K): δ [ppm] = 1.52 (dtd, 2H, J = 12.8, 8.4, 3.9 Hz, 2H), 1.79-1.94 (m, 2H), 2.81 (t, J = 2.4 Hz, 1H), 3.16 (ddd, J = 13.6, 8.4, 3.5 Hz, 2H), 3.62-3.73 (m, 2H), 3.78 (tt, J = 8.0, 3.9 Hz, 1H), 4.22 (d, J = 2.4 Hz, 2H). ^{13}C-NMR (75.5 MHz, CD_3OD, 287 K): δ [ppm] = 25.3 (s, 2C), 31.8 (s, 2C), 42.4 (s, 1C), 56.0 (s, 1C), 64.7 (s, 1C), 74.7 (s, 1C), 161.0 (s, 1C). HRMS-ESI (m/z): calc. ($C_9H_{15}N_2O_2$ $[M+H]^+$): 183.1128; found: 183.1128.

(3-Phenyl-1,2-oxaziridin-2-yl)(4-(prop-2-yn-1-yloxy)piperidin-1-yl)methanone (OxMet2-alkyne): Ti(O^{i}Pr)$_4$ (500 µL, 475 mg, 1.67 mmol, 1.51 equiv.) was added to a solution of crude **33** (201 mg, 1.10 mmol, 1.00 equiv.) and benzaldehyde (133 µL, 141 mg, 1.32 mmol, 1.20 equiv.) in THF (4 mL). After stirring for 4 h, the mixture was concentrated *in vacuo* to give the corresponding benzylidene urea* as a crude residue. Saturated aqueous K_2CO_3 (12 mL) and *m*CPBA (2.28 g, 13.2 mmol, 12.0 equiv.) were stirred in CH_2Cl_2 (36 mL) for 10 min. To this mixture, a solution of the crude benzylidene urea in CH_2Cl_2 (12 mL) was slowly added and stirring was continued for 1 h before addition of H_2O (20 mL). Layers were separated and the aqueous layer was extracted with CH_2Cl_2 (3 × 20 mL). The combined organic layers were washed with brine, dried over Na_2SO_4, filtered and concentrated *in vacuo*. Column chromatography (pentane:EtOAc 7:3) gave **OxMet2-alkyne** (37.0 mg, 129 µmol, 12% over two steps) as a clear oil. TLC (pentane:EtOAc 7:3): R_f = 0.28 [PMA]. ^{1}H-NMR (500 MHz, $CDCl_3$, 287 K): δ [ppm] = 1.61-1.80 (m, 2H), 1.82-2.00 (m, 2H), 2.42 (t, J = 2.4 Hz, 1H), 3.37 (ddd, J = 13.4, 8.3, 3.7 Hz, 0.5H), 3.37 (ddd, J = 13.6, 7.7, 3.9 Hz, 1H), 3.64-3.79 (m, 1H), 3.75-3.94 (m, 2H), 4.00-4.14 (m, 0.5H), 4.18-4.23 (apparent t, J ≈ 2.6 Hz, 2H), 5.22 (s, 0.5H), 5.23 (s, 0.5H), 7.37-7.52 (m, 5H). ^{13}C-NMR (75.5 MHz, $CDCl_3$, 300 K): δ [ppm] = 30.3 (s, 0.5C), 30.5 (s, 0.5C), 31.0 (s, 0.5C), 31.2 (s, 0.5C), 40.9 (s, 0.5C), 41.3 (s, 0.5C), 41.9 (s, 0.5C), 42.2 (s, 0.5C), 55.5 (s, 0.5C), 55.6 (s, 0.5C),

72.5 (s, 0.5C), 72.9 (s, 0.5C), 74.5 (s, 1C), 77.4 (s, 0.5C), 78.1 (s, 0.5C), 78.1 (s, 0.5C), 80.0 (s, 0.5C), 128.1 (s, 2C), 128.8 (s, 2C), 130.9 (s, 1C), 133.1 (s, 0.5C), 133.1 (s, 0.5C), 160.7 (s, 1C). HRMS-ESI (m/z): calc. ($C_{16}H_{19}N_2O_3$ $[M+H]^+$): 287.1390; found: 287.1391.

*TLC (pentane:EtOAc 3:2): R_f = 0.30 [$KMnO_4$].

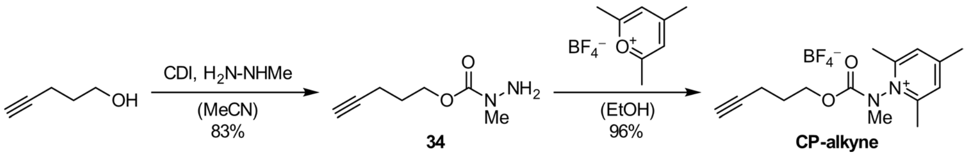

Pent-4-yn-1-yl 1-methylhydrazine-1-carboxylate (34): Pent-4-yn-1-ol (443 µL, 400 mg, 4.76 mmol, 1.00 equiv.) was added dropwise to a suspension of 1,1'-Carbonyldiimidazole (1.16 g, 7.13 mmol, 1.50 equiv.) in dry MeCN (10 mL) over 10 min. After 4 h complete conversion was indicated by TLC*, and methylhydrazine (501 µL, 438 mg, 9.51 mmol, 2.00 equiv.) was added. After 4 h complete reaction was indicated by TLC and the reaction mixture was concentrated by N_2-stream, the residue was dissolved in CH_2Cl_2 (10 mL) and washed with half-saturated aqueous $NaHCO_3$ (4 × 15 mL). Concentration *in vacuo* gave **34** (616 mg, 3.95 mmol, 83%) as a yellow oil, which was used without any further purifications. TLC (pentane:EtOAc 1:3): R_f = 0.18 [$KMnO_4$]. ^{1}H-NMR (500 MHz, $CDCl_3$, 300 K): *δ* [ppm] = 1.82-1.93 (m, 2H), 1.94-1.98 (m, 1H), 2.25-2.33 (m, 2H), 3.05-3.13 (m, 3H), 4.12 (br. s, 2H), 4.17-4.25 (m, 2H). ^{13}C-NMR (101 MHz, $CDCl_3$, 298 K): *δ* [ppm] = 15.3 (s, 1C), 28.0 (s, 1C), 38.4 (s,1C), 64.6 (s, 1C), 69.1 (s,1C), 83.2 (s, 1C), 157.3 (s, 1C). HRMS-ESI (m/z): calc. ($C_7H_{13}N_2O_2$ $[M+H]^+$): 157.0972; found: 157.0971.

*R_f = 0.31 (pentane:EtOAc 1:3)

2,4,6-Trimethyl-1-(methyl((pent-4-yn-1-yloxy)carbonyl)amino)pyridin-1-ium tetrafluoroborate (CP-alkyne): A mixture of **34** (200 mg, 1.28 mmol, 1.10 equiv.) and 2,4,6-trimethyl pyrylium tetrafluoroborate (244 mg, 1.16 mmol, 1.00 equiv.) in dry EtOH (4.5 mL) was stirred for 18 h before concentration *in vacuo*. The solution of the residue in CH_2Cl_2 and EtOH (2:1, 1 mL) was slowly added to Et_2O (11 mL) and the resulting biphasic mixture was stored at −20 °C for 24 h. The supernatant was discarded and the residue washed with Et_2O (12 mL). Removal of residual Et_2O *in vacuo* gave **CP-alkyne** (387 mg, 1.11 mmol, 96%) as a thick orange oil. The observed ratio of rotamers in the ^{1}H-NMR experiment is 1.2:1. ^{1}H-NMR (300 MHz, DMSO-d_6, 300 K): *δ* [ppm] = 1.56-1.68 (apparent p, $J \approx 6.7$ Hz, 2H, minor), 1.82-1.93 (apparent p, $J \approx 6.7$ Hz, 2H, major), 1.97 (td, J = 7.0, 2.7 Hz, 2H, minor), 2.34 (td, J = 7.0, 2.7 Hz, 2H, major), 2.59 (s, 3H, major and minor), 2.64 (s, 6H, major), 2.66 (s, 6H, minor), 2.79 (t, J = 2.7 Hz, 1H, minor), 2.87 (t, J = 2.7 Hz, 1H, major), 3.47 (s, 3H, minor), 3.53 (s, 3H, major), 4.18 (t, J = 6.3 Hz, 2H, minor), 4.33 (t, J = 6.3 Hz, 2H, major), 7.95 (s, 2H, major), 7.96 (s, 2H, minor). ^{13}C-NMR (101 MHz, DMSO-d_6, 298 K): *δ* [ppm] = 14.6 (s, 1C, minor), 14.9 (s, 1C, major), 18.6 (s, 1C, major), 18.7 (s, 1C, minor), 22.0 (s, 1C, major), 22.0 (s, 1C, minor), 27.1 (s, 1C, minor), 27.5 (s, 1C, major), 36.7 (s, 1C, minor), 27.6 (s, 1C, major), 66.9 (s, 1C, minor), 67.3 (s, 1C, major), 72.3 (s, 1C, major), 72.4 (s, 1C, minor), 83.3 (s, 1C, minor), 83.8 (s, 1C, major), 128.8 (s, 2C, minor), 128.9 (s, 2C, major), 151.7 (s, 1C, minor), 153.1 (s,1C, major), 157.1 (s, 2C, minor), 157.3 (s, 2C, major), 161.7 (s, 1C, major), 161.8 (s, 1C, minor). ^{19}F-NMR (376 MHz, DMSO-d_6, 298 K): *δ* [ppm] = −148.3 (s, $^{11}BF_4$), −148.3 (s, $^{10}BF_4$). (s). HRMS-ESI (m/z): calc. ($C_{15}H_{21}N_2O_2$ $[M-BF_4]^+$): 261.1598; found: 261.1600.

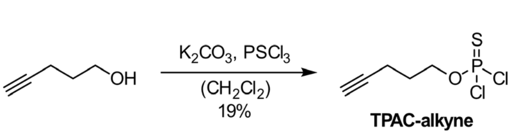

***O*-(Pent-4-yn-1-yl) phosphorodichloridothioate (TPAC-alkyne):** K_2CO_3 (1.35 g, 9.80 mmol, 1.00 equiv.) and pent-4-yn-1-ol (912 µL, 824 mg, 9.80 mmol, 1.00 equiv.) were added to a solution of $PSCl_3$ (994 µL, 1.66 g, 9.80 mmol, 1.00 equiv.) in dry CH_2Cl_2 (10 mL). The reaction mixture was stirred for 17 h, filtered and purified by column chromatography (pentane:EtOAc 100:1 → 10:1) to give **TPAC-alkyne** (406 mg, 1.87 mmol, 19%) as a yellow oil. TLC (pentane:EtOAc 1:3): R_f = 0.59 [$KMnO_4$]. ^{1}H-NMR (300 MHz, $CDCl_3$, 300 K): *δ* [ppm] = 1.96-2.08 (m, 3H), 2.39 (td, J = 6.8, 2.7 Hz, 2H), 4.47 (dt, J = 6.0, 11.0 Hz, 2H). ^{13}C-NMR (75.5 MHz, $CDCl_3$, 300 K): *δ* [ppm] = 14.9 (s, 1C), 28.5 (d, J = 10.0 Hz, 1C), 70.0 (s,1C), 70.6 (d, J = 10.4 Hz, 1C), 82.1 (s,1C). ^{31}P-NMR (122 MHz, $CDCl_3$, 300 K): *δ* [ppm] = 58.1 (s, 1P). HRMS-APCI (m/z): calc. ($C_5H_8Cl_2OPS$ $[M+H]^+$): 216.9405; found: 216.9406.

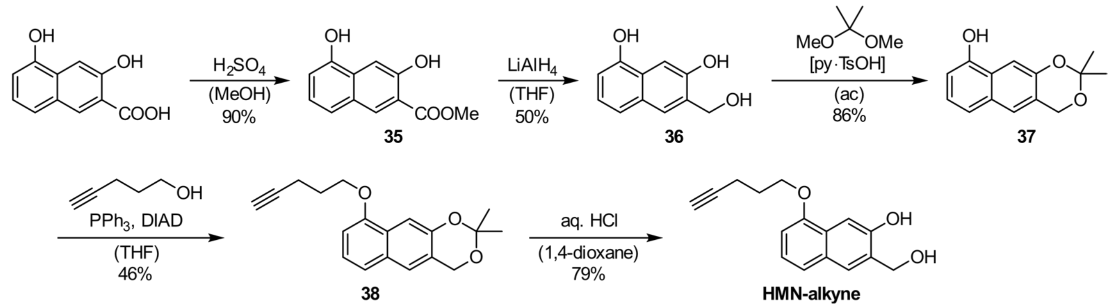

Methyl 3,5-dihydroxy-2-naphthoate (35): 3,5-Dihydroxy-2-naphthoic acid (10.0 g, 49.0 mmol, 1.00 equiv.) was dissolved in MeOH (150 mL), concentrated H_2SO_4 (3.33 mL, 6.00 g, 61.2 mmol, 1.25 equiv.) was added and the mixture was heated to reflux overnight. After cooling to room temperature, the organic solvent was removed *in vacuo*, EtOAc (500 mL) was added to the resulting residue and the layers were separated. The organic layer was washed with aqueous Na_2CO_3 solution (1 M, 5 × 100 mL), water (3 × 100 mL) and brine (2 × 100 mL), dried over $MgSO_4$, filtered and concentrated *in vacuo*, giving **35** (9.59 g, 44.0 mmol, 90%) as an ochre-coloured solid which was used without further purification. TLC (pentane:EtOAc 1:1): R_f = 0.81 [UV, CPM]. ^{1}H-NMR (500 MHz, DMSO-d$_6$, 300 K): δ [ppm] = 3.93 (s, 3H), 6.88 (d, J = 7.4 Hz, 1H), 7.12-7.19 (apparent t, $J \approx$ 7.8 Hz, 1H), 7.40 (d, J = 8.2 Hz, 1H), 7.49 (s, 1H), 8.37 (s, 1H), 10.13 (s, 1H), 10.18 (s, 1H). ^{13}C-NMR (126 MHz, DMSO-d$_6$, 300 K): δ [ppm] = 52.6 (s, 1C), 105.9 (s, 1C), 110.2 (s, 1C), 116.5 (s, 1C), 119.6 (s, 1C), 124.3 (s, 1C), 128.6 (s, 1C), 131.8 (s, 1C), 151.6 (s, 1C), 154.0 (s, 1C), 168.6 (s, 1C). HRMS-ESI (m/z): calc. ($C_{12}H_9O_4$ $[M-H]^-$): 217.0506; found:217.0507.

6-(Hydroxymethyl)naphthalene-1,7-diol (36): To a solution of **35** (9.30 g, 42.6 mmol, 1.00 equiv.) in dry THF (200 mL), a solution of $LiAlH_4$ in THF (1 M, 51.1 mL, 1.94 g, 51.1 mmol, 1.20 equiv.) was added dropwise at 0 °C. The resulting yellow suspension was allowed to warm to room temperature and was stirred for 2 h before being poured into cold Et_2O (200 mL) and addition of aqueous HCl (5%, 60 mL). The layers were separated and the organic layer was washed with water (2 × 100 mL) and brine (100 mL), dried over Na_2SO_4, filtered and concentrated *in vacuo*. Column chromatography (pentane:EtOAc 2.5:1 → 1:2.5) gave **36** (4.07 g, 21.4 mmol, 50%) as a yellow solid which was used without further purification. TLC (pentane:EtOAc 1:1): R_f = 0.49 [UV, CPM]. ^{1}H-NMR (400 MHz, DMSO-d$_6$, 300 K): δ [ppm] = 4.62 (d, J = 5.5 Hz, 2H), 5.12 (d, J = 5.6 Hz, 1H), 6.71 (d, J = 7.4 Hz, 1H), 6.99-7.06 (apparent t, $J \approx$ 7.8 Hz, 1H), 7.20 (d, J = 8.1 Hz, 1H), 7.36 (s, 1H), 8.70 (s, 1H), 9.65 (s, 1H), 9.72 (s, 1H). ^{13}C-NMR (126 MHz, DMSO-d$_6$, 300 K): δ [ppm] = 58.7 (s, 1C), 103.0 (s, 1C), 107.4 (s, 1C), 118.2 (s, 1C), 122.8 (s, 1C), 124.7 (s, 1C), 125.1 (s, 1C), 129.1 (s, 1C), 131.7 (s, 1C), 151.6 (s, 1C), 152.2 (s, 1C). HRMS-ESI (m/z): calc. ($C_{11}H_9O_3$ $[M-H]^-$): 189.0557; found: 189.0557.

2,2-Dimethyl-4*H*-naphtho[2,3-*d*][1,3]dioxin-9-ol (37): To a solution of **36** (7.08 g, 37.2 mmol, 1.00 equiv.) in acetone (150 mL) were added 2,2-dimethoxypropane (16.1 mL, 13.7 g, 131 mmol, 3.53 equiv.) and a catalytic amount of pyridinium *p*-toluenesulfonate (~100 mg). The mixture was stirred at room temperature for 24 h, after which the solvent was removed *in vacuo*. After addition of H_2O (60 mL) to the resulting residue, the crude mixture was extracted with EtOAc (2 × 40 mL). The combined organic layers were washed with water (2 × 30 mL) and brine (30 mL), dried over Na_2SO_4, filtered and concentrated *in vacuo*. Column chromatography (pentane:EtOAc 5:1 → 1:1) gave **37** (7.37 g, 32.0 mmol, 86%) as a yellow solid. TLC (pentane:EtOAc 5:1): R_f = 0.60 [UV, CPM]. ^{1}H-NMR (500 MHz, DMSO-d$_6$, 300 K): δ [ppm] = 1.52 (s, 6H), 5.02 (s, 2H), 6.76 (dd, J = 7.4, 0.8 Hz, 1H), 7.11 (dd, J = 8.2, 7.4 Hz, 1H), 7.21 (d, J = 8.3 Hz, 1H), 7.37 (s, 1H), 7.53 (s, 1H), 9.88 (s, 1H). ^{13}C-NMR (101 MHz, DMSO-d$_6$, 300 K): δ [ppm] = 24.8 (s, 2C), 60.4 (s, 1C), 99.5 (s, 1C), 105.9 (s, 1C), 107.6 (s, 1C), 118.0 (s, 1C), 121.5 (s, 1C), 123.5 (s, 1C), 124.1 (s, 1C), 124.9 (s, 1C), 129.4 (s, 1C), 148.6 (s, 1C), 152.0 (s, 1C). HRMS-ESI (m/z): calc. ($C_{14}H_{15}O_3$ $[M+H]^+$): 231.1016; found: 231.1016.

2,2-Dimethyl-9-(pent-4-yn-1-yloxy)-4*H*-naphtho[2,3-*d*][1,3]dioxine (38): **37** (1.68 g, 7.30 mmol, 1.00 equiv.), 4-pentyn-1-ol (1.09 mL, 982 mg, 11.7 mmol, 1.60 equiv.) and triphenylphosphine (3.07 g, 11.7 mmol, 1.60 equiv.) were sonicated in THF (2.4 mL) until a highly viscous but clear solution was obtained. Di*iso*propylazodicarboxylate (2.29 mL, 2.36 g, 11.7 mmol, 1.60 equiv.) was added dropwise over the course of 2 min while sonicating. The reaction mixture was sonicated for additional 15 min before it was diluted with CH_2Cl_2 (100 mL) and washed with water (2 × 70 mL) and brine (1 × 70 mL). Layers were separated and the organic layer was dried over Na_2SO_4, filtered and concentrated *in vacuo*. Column chromatography (pentane:EtOAc 50:1 → 10:1) gave **38** (1.00 g, 3.38 mmol, 46%) as a pale yellow oil. TLC (pentane:EtOAc 10:1): R_f = 0.76 [UV, CPM]. ^{1}H-NMR (500 MHz, DMSO-d$_6$, 300 K): δ [ppm] = 1.52 (s, 6H), 1.99-2.05 (apparent p, $J \approx$ 6.6 Hz, 2H), 2.44 (td, J = 7.1, 2.7 Hz, 2H), 2.82

(t, J = 2.7 Hz, 1H), 4.17 (t, J = 6.0 Hz, 2H), 5.03 (s, 2H), 6.86 (d, J = 7.5 Hz, 1H), 7.20-7.25 (apparent t, $J \approx$ 7.9 Hz, 1H), 7.35 (d, J = 8.3 Hz, 1H), 7.41 (s, 1H), 7.60 (s, 1H). ^{13}C-NMR (101 MHz, DMSO-d$_6$, 300 K): δ [ppm] = 14.7 (s, 1C), 24.7 (s, 2C), 27.8 (s, 1C), 60.3 (s, 1C), 66.0 (s, 1C), 71.6 (s, 1C), 83.7 (s, 1C), 99.6 (s, 1C), 104.7 (s, 1C), 105.6 (s, 1C), 119.5 (s, 1C), 121.7 (s, 1C), 123.6 (s, 1C), 123.9 (s, 1C), 125.3 (s, 1C), 128.9 (s, 1C), 149.1 (s, 1C), 152.9 (s, 1C). HRMS-ESI (m/z): calc. ($C_{19}H_{21}O_3$ [M+H]$^+$):297.1485; found: 297.1486.

3-(Hydroxymethyl)-8-(pent-4-yn-1-yloxy)naphthalen-2-ol (HMN-alkyne): To a solution of **38** (1.00 g, 3.38 mmol, 1.00 equiv.) in 1,4-dioxane (30 mL), was added aqueous HCl (3 M, 6 mL, 656 mg, 18.0 mmol, 5.33 equiv.) and the reaction mixture was stirred overnight. The organic solvent was removed *in vacuo* and the aqueous residue was extracted with EtOAc (2 × 40 mL). The combined organic phases were washed with water (2 × 40 mL) and brine (40 mL), dried over Na_2SO_4, filtered and concentrated *in vacuo*. Column chromatography (pentane:EtOAc 5:1 → 1:1) gave **HMN-alkyne** (682 mg, 2.66 mmol, 79%) as a beige solid. TLC (pentane:EtOAc 1:1): R_f = 0.50 [UV, CPM]. ^{1}H-NMR (500 MHz, DMSO-d$_6$, 300 K): δ [ppm] = 1.99-2.06 (apparent p, $J \approx$ 6.6 Hz, 2H), 2.46 (td, J = 7.2, 2.7 Hz, 2H), 2.84 (t, J = 2.7 Hz, 1H), 4.17 (t, J = 6.0 Hz, 2H), 4.62 (dd, J = 5.6, 0.9 Hz, 2H), 5.13 (t, J = 5.6 Hz, 1H), 6.81 (d, J = 7.5 Hz, 1H), 7.14 (t, J = 7.9 Hz, 1H), 7.33 (d, J = 8.2 Hz, 1H), 7.43 (s, 1H), 7.76 (s, 1H), 9.75 (s, 1H). ^{13}C-NMR (101 MHz, DMSO-d$_6$, 300 K): δ [ppm] = 14.8 (s, 1C), 28.0 (s, 1C), 58.5 (s, 1C), 66.0 (s, 1C), 71.7 (s, 1C), 83.7 (s, 1C), 102.5 (s, 1C), 104.5 (s, 1C), 119.8 (s, 1C), 122.6 (s, 1C), 125.1 (s, 1C), 125.1 (s, 1C), 128.6 (s, 1C), 132.0 (s, 1C), 152.6 (s, 1C), 152.7 (s, 1C). HRMS-ESI (m/z): calc. ($C_{16}H_{15}O_3$ [M−H]$^-$): 255.1027; found: 255.1027.

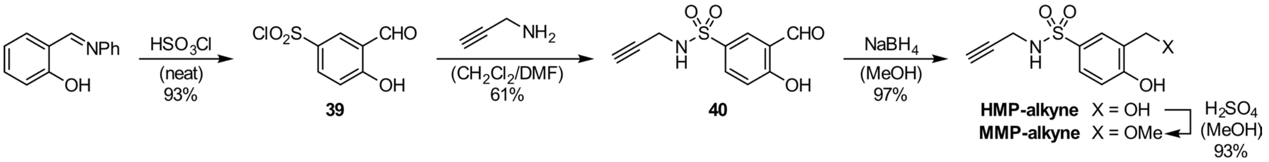

3-Formyl-4-hydroxybenzenesulfonyl chloride (39): Synthesis of **39** was carried out according to literature procedure.[73] Salicylideneaniline (10.0 g, 50.7 mmol, 1.00 equiv.) was slowly added to HSO_3Cl (33.3 mL, 58.3 g, 500 mmol, 9.86 equiv.) at 0 °C. The mixture was stirred for 19 h, during which it was allowed to warm to room temperature, and then added to ice (300 g) in portions. The resulting mixture was extracted with CH_2Cl_2 (3 × 100 mL), the combined organic layers were washed with aqueous H_2SO_4 (9 M, 3 × 100 mL) and brine (100 mL), dried over Na_2SO_4, filtered and concentrated *in vacuo*, giving **39** (10.3 g, 46.5 mmol, 92%) as a yellow solid. TLC (pentane:EtOAc: 2:1): R_f = 0.25 [UV, DNP]. ^{1}H-NMR (300 MHz, $CDCl_3$, 300 K): δ [ppm] = 7.19-7.24 (apparent dt, J = 9.0, $J \approx$ 0.4 Hz, 1H), 8.16 (ddd, J = 9.0, 2.5, 0.4 Hz, 1H), 8.32 (dd, J = 2.5, 0.3 Hz, 1H), 10.00 (s, 1H), 11.70 (s, 1H). ^{13}C-NMR:(101 MHz, $CDCl_3$, 300K): δ [ppm] = 119.9 (s, 1C), 120.0 (s, 1C), 133.8 (s, 1C), 134.9 (s, 1C), 135.9 (s, 1C), 166.6 (s, 1C), 195.4 (s, 1C). The analytical data is in accordance with literature.[73]

3-Formyl-4-hydroxy-*N*-(prop-2-yn-1-yl)benzenesulfonamide (40): To a solution of **39** (1.00 g, 4.53 mmol, 1.00 equiv.) in dry CH_2Cl_2 (15 mL), propargylamine (753 µL, 648 mg, 11.8 mmol, 2.59 equiv.) was added dropwise at 0 °C. Due to precipitation, the reaction was diluted with dry DMF (2 mL). The mixture was stirred for 19 h, during which it was allowed to warm to room temperature, before addition of CH_2Cl_2 (50 mL) and aqueous HCl (6 M, 30 mL). The layers were separated and the aqueous layer was extracted with CH_2Cl_2 (2 × 20 mL). The combined organic layers were washed with H_2O (30 mL) and brine (30 mL), dried over $MgSO_4$, filtered and concentrated *in vacuo*. Column chromatography (pentane:EtOAc 2:1) gave **40** (658 mg, 2.75 mmol, 61%) as a white solid. TLC (pentane:EtOAc 2:1): R_f = 0.33 [UV, DNP]. ^{1}H-NMR (400 MHz, CD_3CN, 300 K): δ [ppm] = 2.34 (t, J = 2.5 Hz, 1H), 3.78 (dd, J = 6.2, 2.5 Hz, 2H), 5.97 (br. t, J = 6.2 Hz, 1H), 7.13 (d, J = 8.8 Hz, 1H), 7.97 (dd, J = 8.8, 2.4 Hz, 1H), 8.22 (d, J = 2.4 Hz, 1H), 10.00 (s, 1H), 11.29 (s, 1H). ^{13}C-NMR:(101 MHz, CD_3CN, 300 K): δ [ppm] = 33.2 (s, 1C), 73.9 (s, 1C), 79.5 (s, 1C), 119.4 (s, 1C), 121.1 (s, 1C), 132.8 (s, 1C), 134.8 (s, 1C), 136.1 (s, 1C), 165.2 (s, 1C), 197.9 (s, 1C). HRMS-ESI (m/z): calc. ($C_{10}H_8NO_4S$ [M−H]$^-$): 238.0180; found: 238.0179.

4-Hydroxy-3-(hydroxymethyl)-*N*-(prop-2-yn-1-yl)benzenesulfonamide (HMP-alkyne): $NaBH_4$ (38.0 mg, 1.00 mmol, 2.00 equiv.) was added to a solution of **40** (120 mg, 502 µmol, 1.00 equiv.) in dry MeOH (12 mL) in portions at 0 °C. After stirring for 6 h at room temperature, the pH was adjusted to 4-5 with glacial acetic acid (~100 mL), before concentration *in vacuo*. EtOAc (50 mL) and H_2O (30 mL) were added to the resulting residue, the layers were separated, the aqueous phase was extracted with EtOAc (2 × 20 mL). The combined organic layers were washed with brine (20 mL) dried over $MgSO_4$, filtered and concentrated *in vacuo*. Column chromatography (pentane:EtOAc 1:2) gave **HMP-alkyne** (117 mg, 485 mmol, 97%) as a colourless oil. TLC (pentane:EtOAc 1:2):

R_f = 0.38 [UV, $KMnO_4$]. ^{1}H-NMR (400 MHz, CD_3CN, 300 K): δ [ppm] = 2.36 (t, J = 2.5 Hz 1H), 3.57 (br. s, 1H), 3.69 (dd, J = 6.2, 2.5 Hz, 2H), 4.69 (s, 2H), 5.78 (t, J = 6.2 Hz, 1H), 6.92 (d, J = 8.5 Hz, 1H), 7.60 (dd, J = 8.5 Hz, J = 2.4 Hz, 1H), 7.72 (d, J = 2.4 Hz, 1H), 8.20 (br. s, 1H). ^{13}C-NMR:(101 MHz, CD_3CN, 300 K): δ [ppm] = 33.2 (s, 1C), 61.1 (s, 1C), 73.5 (s, 1C), 79.8 (s, 1C), 116.3 (s, 1C), 128.0 (s, 1C), 128.9 (s, 1C), 129.0 (s, 1C), 131.7 (s, 1C), 159.7 (s, 1C). HRMS-ESI (m/z): calc. ($C_{10}H_{10}NO_4S$: $[M{-}H]^-$): 240.0336; found 240.0336.

4-Hydroxy-3-(methoxymethyl)-*N*-(prop-2-yn-1-yl)benzenesulfonamide (MMP-alkyne): Concentrated H_2SO_4 (260 µL, 478 mg, 4.88 mmol, 23.5 equiv.) was added dropwise to a solution of **HMP-alkyne** (50.0 mg, 207 µmol, 1.00 equiv.) in dry MeOH (1.6 mL). After 30 min, the reaction mixture was heated to reflux for 2 h and then cooled to room temperature. The pH was adjusted to 3-4 with NaOH (10 M in H_2O) and the mixture was concentrated *in vacuo*. The resulting residue was dissolved in EtOAc (50 mL) and H_2O (25 mL), the layers were separated, the organic layer was washed with brine (25 mL), dried over $MgSO_4$, filtered and concentrated *in vacuo*. Column chromatography (pentane:EtOAc 1:1) gave **MMP-alkyne** (49.3 mg, 193 µmol, 93%) as a white solid. TLC (pentane EtOAc: 1:2): R_f = 0.51 [UV, $KMnO_4$]. ^{1}H-NMR (400 MHz, CD_3CN, 300 K): δ [ppm] = 2.35 (t, J = 2.5 Hz 1H), 3.41 (s, 3H), 3.70 (dd, J = 6.2, 2.5 Hz, 2H), 4.53 (s, 2H), 5.79 (t, J = 6.2 Hz, 1H), 6.95 (d, J = 8.5 Hz, 1H), 7.63 (dd, J = 8.5, 2.3 Hz, 1H), 7.69 (d, J = 2.3 Hz, 1H), 8.00 (br. s, 1H). ^{13}C-NMR:(101 MHz, CD_3CN, 300 K): δ [ppm] = 33.2 (s, 1C), 58.8 (s, 1C), 70.7 (s, 1C), 73.5 (s, 1C), 79.8 (s, 1C), 116.5 (s, 1C), 126.2 (s, 1C), 129.0 (s, 1C), 129.4 (s, 1C), 131.8 (s, 1C), 159.8 (s, 1C). HRMS-ESI (m/z): calc. ($C_{11}H_{12}NO_4S$ $[M{-}H]^-$): 254.0493; found 254.0492.

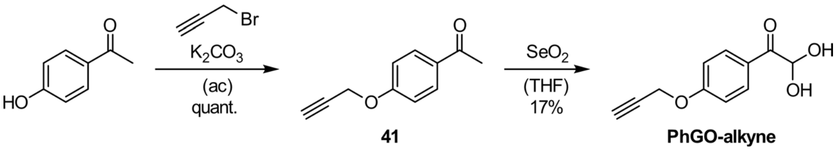

1-(4-(Prop-2-yn-1-yloxy)phenyl)ethan-1-one (41): Synthesis of **41** was carried out similar to literature procedure.[74] Propargyl bromide (80 wt% in PhMe, 491 µL, 524 mg, 4.41 mmol, 1.20 equiv.) was added dropwise to a suspension of 4'-hydroxyacetophenone (500 mg, 3.67 mmol, 1.00 equiv.) and K_2CO_3 (1.02 g, 7.34 mmol, 2.00 equiv.) in dry acetone (5 mL). The reaction mixture was stirred at 50 °C for 8 h, cooled to room temperature, filtered and concentrated *in vacuo* to give **41** (639 mg, 3.67 mmol, quant.) as a white solid. TLC (pentane:EtOAc 3:1): R_f = 0.36 [UV, $KMnO_4$]. ^{1}H-NMR (500 MHz, $CDCl_3$, 300 K): δ [ppm] = 2.55 (t, J = 2.4 Hz, 1H), 2.56 (s, 3H), 4.76 (d, 2.4 Hz, 2H), 6.99-7.06 (m, 2H), 7.92-7.99 (m, 2H). ^{13}C-NMR (101 MHz, $CDCl_3$, 298 K): δ [ppm] = 26.5 (s, 1C), 56.0 (s, 1C), 76.3 (s, 1C), 77.9 (s, 1C), 114.7 (s, 2C), 130.7 (s, 2C), 131.2 (s, 1C), 161.4 (s, 1C), 196.9 (s, 1C). The analytical data is in accordance with literature.[75]

2,2-Dihydroxy-1-(4-(prop-2-yn-1-yloxy)phenyl)ethan-1-one (PhGO-alkyne): A suspension of **41** (200 mg, 1.15 mmol, 1.00 equiv.) and SeO_2 (510 mg, 4.59 mmol, 4.00 equiv.) in THF (2 mL) was heated to reflux for 25 h. After cooling to room temperature, the mixture was filtered over kieselgur, which was rinsed with EtOAc, and the filtrate was concentrated *in vacuo*. Purification by HPLC (MeCN:H_2O 2:3 → 3:2) gave **PhGO-alkyne** (41.2 mg, 200 µmol, 17%) as a white solid.* TLC (pentane:EtOAc 1:1): R_f = 0.31 [UV, $KMnO_4$]. ^{1}H-NMR (500 MHz, CD_3CN, 300 K): 2.86 (t, J = 2.4 Hz, 1H), 4.76-4.82 (m, 2H), 4.84 (d, J = 2.4 Hz, 2H), 5.84 (t, J = 8.1 Hz, 1H), 7.06-7.12 (m, 2H), 8.06-8.12 (m, 2H). ^{1}H-NMR (300 MHz, CD_3CN/D_2O 1:1, 300 K): 2.92 (t, J = 2.4 Hz, 1H), 4.81 (d, J = 2.4 Hz, 2H), 5.85 (s, 1H), 7.03-7.13 (m, 2H), 8.01-8.09 (m, 2H). ^{13}C-NMR (101 MHz, $CD_3CN:D_2O$ 1:1, 298 K): δ [ppm] = 56.8 (s, 1C), 77.8 (s, 1C), 78.9 (s, 1C), 87.8 (s, 1C), 115.8 (s, 2C), 127.7 (s, 1C), 132.9 (s, 2C), 162.8 (s, 1C), 195.8 (s, 1C). HRMS-ESI (m/z): calc. ($C_{11}H_9O_3$ $[M{-}H_2O{+}H]^+$): 189.0546; found: 189.0547

*adding H_2O or D_2O avoids the elimination of H_2O from the glyoxal hydrate to form the glyoxal, which deteriorates in organic solution.

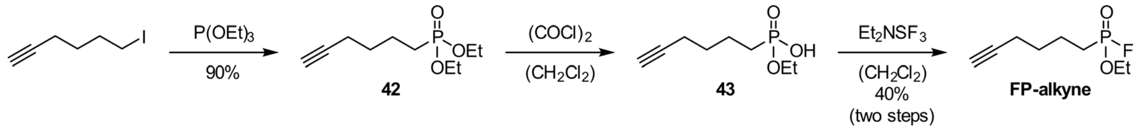

Diethyl hex-5-yn-1-ylphosphonate (42): 6-Iodohex-1-yne (463 mg, 2.23 mmol, 1.00 equiv.) and $P(OEt)_3$ (1.85 mL, 1.79 g, 10.8 mmol, 4.85 equiv.) were heated to 155 °C for 3 h. After cooling to room temperature, the reaction mixture was concentrated *in vacuo*. As purification through column chromatography (CH_2Cl_2/EtOAc 1:1) was unsuccessful,

remaining phosphite was removed *in vacuo* at 85 °C. TLC (CH_2Cl_2:EtOAc 1:1): R_f = 0.20 [$KMnO_4$]. ^{1}H-NMR (300 MHz, $CDCl_3$, 300 K): 1.32 (t, *J* = 7.1 Hz, 6H), 1.55-1.83 (m, 6H), 1.94 (t, *J* = 2.6 Hz, 1H), 2.21 (td, *J* = 6.8, 2.6 Hz, 2H), 4.02-4.17 (m, 4H). ^{31}P-NMR (122 MHz, $CDCl_3$, 300 K): 31.8 (s). The analytical data is in accordance with literature.[76]

Ethyl hydrogen hex-5-yn-1-ylphosphonate (43): To a solution of **42** (239 mg, 1.09 mmol, 1.00 equiv.) in dry CH_2Cl_2 (4 mL), was added $(COCl)_2$ (563 µL, 833 mg, 6.56 mmol, 6.00 equiv.). Stirring was continued for 10 h and the reaction mixture was concentrated *in vacuo.* H_2O (2 mL) was added to the resulting residue and, after stirring for 5 min, this was extracted with CH_2Cl_2 (3 × 5 mL). The combined organic layers were dried over Na_2SO_4 and filtered. Concentration *in vacuo* gave crude **43** (194 mg) as a brown oil, which was used without any further purification. HRMS-ESI (m/z): calc. ($C_8H_{16}O_3P$ $[M+H]^+$): 191.0832; found: 191.0828

Ethyl hex-5-yn-1-ylfluorophosphonate (FP-alkyne): To a solution of crude **43** (75.0 mg, 394 µmol, 1.00 equiv.) in dry CH_2Cl_2 (1 mL), was added Et_2NSF_3 (208 µL, 254 mg, 1.58 mmol, 4.00 equiv.) was added dropwise over 10 min at 0 °C. Stirring was continued for 3 h at room temperature before concentration *in vacuo*. H_2O (1 mL) and CH_2Cl_2 (3 mL) were added to the resulting residue, layers were separated and the aqueous layer was extracted with CH_2Cl_2 (2 × 5 mL). The combined organic layers were dried over Na_2SO_4, filtered and concentrated *in vacuo*. Column chromatography (pentane:EtOAc 3:1) gave **FP-alkyne** (32.8 mg, 171 µmol, 40% over two steps) as a slightly yellow oil. TLC (pentane:EtOAc 1:1): R_f = 0.39 [$KMnO_4$]. ^{1}H-NMR (400 MHz, $CDCl_3$, 298 K): 1.38 (t, *J* = 7.1 Hz, 3H), 1.60-1.69 (apparent quint., *J* ≈ 6.9 Hz, 2H), 1.73-1.86 (m, 2H), 1.86-1.91 (m, 1H), 1.91-1.96 (m, 1H), 1.96 (t, *J* = 2.6 Hz, 1H), 2.21 (td, *J* = 6.9, 2.6 Hz, 2H), 4.19-4.34 (m, 2H). ^{13}C-NMR (101 MHz, $CDCl_3$, 298 K): 16.5 (d, *J* = 5.7 Hz, 1C), 18.0 (s, 1C), 21.2 (d, *J* = 5.3 Hz, 1C), 24.0 (dd, *J* = 144.0, 22.9 Hz, 1C), 28.9 (d, *J* = 17.2 Hz, 1C), 63.3 (d, *J* = 7.3 Hz, 1C), 69.2 (s, 1C), 83.4 (s, 1C). ^{19}F-NMR (376 MHz, $CDCl_3$, 298 K): −64.3 (d, *J* = 1070 Hz). ^{31}P-NMR (162 MHz, $CDCl_3$, 298 K): 31.1 (d, *J* = 1070 Hz). HRMS-ESI (m/z): calc. ($C_8H_{15}FO_2P$ $[M+H]^+$): 193.0788; found: 191.0784.

Synthesis of the isoDTB tags

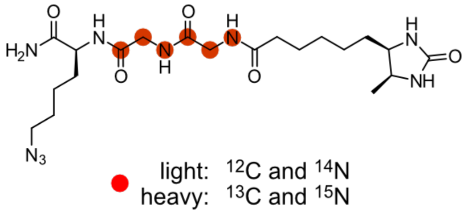

Synthesis of the light and heavy isoDTB tags was carried out identical to literature procedure.[10]

Control experiment for the role of CuAAC conditions in labelling with DA1-alkyne

The two technical replicates were prepared separately as distinct samples starting from the same lysate. Two samples of 1.00 mL freshly prepared *S. aureus* SH1000 lysate were incubated with 20 µL of **DA1-alkyne** (5 mM in DMF) (1 h, r.t.). For each sample, one 5 mL Zeba Spin Desalting Column (7k MWCO, Thermo Scientific, 89891) was used to remove excess probe according to the manufacturer's protocol. For this purpose, the storage solution was removed by centrifugation (2 min, 1,000 ×g) and the resin was washed four times with 2.5 mL PBS using centrifugation (2 min, 1,000 ×g). The samples were eluted by centrifugation (2 min, 1,000 ×g) and 1.00 mL were transferred into an microcentrifuge tube. CuAAC and all subsequent steps were carried out as described above for other isoDTB-ABPP experiments.

isoDTB-ABPP Experiments with CP-alkyne under inert gas

The two technical replicates were prepared separately as distinct samples starting from the same lysate. Two samples of 2.00 mL freshly prepared lysate of the indicated cells were incubated with 40 µL of **CP-alkyne** (5 or 50 mM in DMSO for a final concentration of 100 µM or 1 mM. respectively) (10 min, r.t.) and then sparged with argon (20 min, r.t.). 1.20 mL of each sample was transferred to an argon-filled screw cap glass vial and irradiated (10 min, 280-315 nm). 1.00 mL of each sample was transferred to an microcentrifuge tube. CuAAC and all subsequent steps were carried out as described above.

Chemoproteomic experiment without enrichment

The two technical replicates were prepared separately as distinct samples starting from the same lysate. 200 µL freshly prepared *S. aureus* SH1000 lysate were incubated with 4 µL of **IA-alkyne** (5 mM stock in DMSO, 100 µM final concentration) (1 h, r.t.). The samples were then added to 800 µL cold acetone in order to precipitate all proteins. The precipitates were stored at −20 °C overnight.

The protein precipitates were centrifuged (14,800 rpm, 10 min) and the supernatant was removed. The precipitates were resuspended in 1 mL cold MeOH by sonification and centrifuged (10 min, 21,000 ×g, 4 °C). The supernatant was removed and the washing step with MeOH was repeated once. The pellets were dissolved in 300 µL urea (8 M in 0.1 M aqueous triethylammonium bicarbonate (TEAB)) by sonification and sequentially incubated with 15 µL dithiothreitol (DTT; 31 mg/mL in H_2O) (45 min, 200 rpm, 37 °C), 15 µL iodoacetamide (74 mg/mL in H_2O) (30 min, 200 rpm, r.t.) and 15 µL DTT (31 mg/mL in H_2O) (30 min, 200 rpm, r.t.). The samples were diluted with 900 µL TEAB (0.1 M in H_2O) and incubated with 4 µL trypsin (0.5 mg/mL; Promega, V5113) (overnight, 200 rpm, 37 °C).

The samples were acidified by addition of 20 µL TFA (pH ≤ 3) and desalted over C18 cartridges (Sep-Pak 50 mg, Waters, WAT054955). The cartridges were washed with 2 × 1 mL MeCN and 3 × 1 mL TFA (0.1% in H_2O) prior to loading the samples. After washing with 3 × 1 mL TFA (0.1% in H_2O), peptides were eluted with 3 × 250 µL TFA (0.1% in 80% MeCN in H_2O). The solvent was removed in a rotating vacuum concentrator (~5 h, 30 °C) and the resulting residue was dissolved in 30 µL TFA (0.1% in H_2O) by sonification for 5 min. Samples were filtered through filters (Merck, UVC30GVNB) washed with the same solution by centrifugation (3 min, 17,000 ×g). The samples were then transferred to MS sample vials and stored at −20 °C until measurement.

isoDTB-ABPP Data Analysis

Adding labelling-based quantification support on the MS1-level to IonQuant

IonQuant[13] was designed as a MS1-based label-free quantification tool. In this work, we extended it by adding the module supporting MS1-based labelling-based quantification. After tracing the MS1 peaks for identified ions (peptide sequence + charge + modifications), IonQuant performs a re-tracing procedure to rescue the peaks which are not identified but can be paired with the identified ones. For each identified ion, IonQuant calculates the theoretical m/z

with the complementary label (e.g., for a light labelled ion, IonQuant calculates the m/z by replacing the light label with the heavy label). Then, it traces the MS1 peaks with the calculated m/z and the retention time from the identified ion. After re-tracing, IonQuant pairs light and heavy ions, calculates log-ratios and Pearson correlation coefficient measuring the similarity of two traced curves. If there are more than one pair of traced curves from the same pair of ions, IonQuant picks the one with the smallest retention time difference. Finally, IonQuant generates ion-level and peptide-level reports for each experiment.

General setup of analysis software

Raw data of the LC-MS/MS analyses was converted into the mzML format using the MSconvert tool (version: 3.0.19172-57d620127) of the ProteoWizard software (version: 3.0.19172 64bit)[77] using standard settings with vendor's peak picking enabled. For all data analysis using MSFragger[11, 12]-based FragPipe, the FragPipe interface (version: 14.0) was used with MSFragger (version: 3.1.1),[11, 12] Philosopher (version: 3.3.10)[78], IonQuant (version 1.4.6)[13] and Python (version: 3.7.3) enabled.

For all datasets produced starting from *S. aureus* SH1000 lysate, a FASTA database was downloaded from www.uniport.org using a search for "93061" as "Taxonomy [OC]" at UniProtKB on 27.02.2018. This corresponds to the FASTA database for the strain NCTC8325. Comparative sequencing of these two strains has been reported[79] and the respective changes have been manually made to the FASTA file. These include several point mutations, the deletion of the partial proteins Q2FWJ0 and Q2FWJ1 as well as the addition of the rsbU gene from *S. aureus* Newman (A0A0H3KE27). For all datasets produced starting from lysate of the human cancer cell line MDA-MB-231, a FASTA database was downloaded from www.uniport.org using a search for "UP000005640" in Proteomes on 29.01.2019 with the option "Download one protein sequence per gene (FASTA)" enabled. In both cases, the reverse sequences were manually added to the FASTA databases.

Analysis of the mass of modifications with FragPipe

To survey the landscape of all mass shifts observed on peptides in the data sets, an Open Search was performed with MSFragger. For this purpose, the following settings were used: Precursor mass tolerance −150 to 1000 Da, (initial) fragment mass tolerance 20 ppm, Calibration and Optimization "Mass calibration, parameter optimization" enabled, Isotope Error "0", enzyme name "trypsin", cut after "KR", but not before "P", cleavage "enzymatic", missed cleavages "2", Clip N-term N enabled, peptide length 7 to 50, peptide mass range 500 to 5000 Da, no variable modifications, no fixed modifications, all other options were left at the standard settings. Crystal-C was enabled. PeptideProphet was run with the following settings: "--nonparam --expectscore --decoyprobs --masswidth 1000.0 --clevel -2". PTMProphet was disabled. ProteinProphet was run with the following settings: "--maxppmdiff 2000000". Generate report was enabled with the following settings: "--sequential --razor --mapmods --prot 0.01". Run MS1 quant was disabled. Run TMT-Integrator was disabled. PTM-Shepherd was enabled with the following settings: Smoothing factor "2", Precursor tolerance "0.01 Da", Prominence ratio "0.3", Peak picking width "0.002 Da", Localization background "4", Annotation tolerance "0.01 Da", Custom mass shifts: a custom mass shift list was used including only UniMod modifications with less than 400 Da molecular weight (This list can be found as "reducedMasslist.txt" in the PRIDE upload for this project), Ion Types for modification with "b" and "y" enabled and mass fragment charge "2". Generate Spectral Library was disabled. The FragPipe workflow with these parameters has been deposited in the PRIDE upload as "open_final.workflow". For duplicates, both runs were analysed as the same experiment.

For downstream data analysis, the "global.modsummary.tsv" file was loaded and the values for the number of PSMs ("default-ptmshepherd-dataset (PSMs)") were plotted against the "Theoretical Mass Shift" in the mass range between 400 and 1000 Da.

Analysis of amino acid selectivity with FragPipe

To analyse the amino acid selectivity, a Mass Offset Search was performed in MSFragger[11, 12]. For this purpose, the following settings were used in MSFragger: Precursor mass tolerance −20 to 20 ppm, fragment mass tolerance 20 ppm, Calibration and Optimization "None", Isotope Error "0/1/2", enzyme name "trypsin", cut after "KR", but not before "P", cleavage "enzymatic", missed cleavages "2", Clip N-term N enabled, peptide length 7 to 50, peptide mass range 500 to 5000 Da, variable modification of 57.02146 Da on C with max. 3 occurrences, no fixed modifications, mass offsets set according to Supplementary Table 6 (If more than one set of offsets are indicated, these were analysed simultaneously in the same FragPipe analysis, except for **Az-alkyne**, for which both analysis in the same run and in separate runs was performed), all other options were left at the standard settings. Crystal-C was disabled. PeptideProphet was run with the following settings: "--nonparam --expectscore --decoyprobs --masswidth 1000.0 --clevel -2". PTMProphet was disabled. ProteinProphet was run with the following settings: "--maxppmdiff 2000000". Generate report was enabled with the following settings: "--sequential --razor --mapmods --prot 0.01". Run MS1 quant

was enabled with the following settings: IonQuant enabled, M/Z Window “10 ppm”, RT Window “0.4 min”, Labelling based quant with the masses indicated in Supplementary Table 6 on any amino acid as indicated by “*” (If more than one set of offsets are indicated, these were analysed simultaneously in the same FragPipe analysis, except for **Az-alkyne**, for which both analysis in the same run and in separate runs was performed), Re-quantify enabled, Top N ions “3” Min freq. “0.5”, Min exps “1”, Min isotopes “2”, Normalize disabled. Run TMT-Integrator was disabled. PTM-Shepherd was enabled with the following settings: Smoothing factor “2”, Precursor tolerance 20 ppm, Prominence ratio “0.3”, Peak picking width “20 ppm”, Localization background “4”, Annotation tolerance “0.01 Da”, Custom mass shifts: “Failed_Carbamidomethylation:-57.021464”, a custom mass shift list was used including only UniMod modifications with less than 400 Da molecular weight (This list can be found as “reducedMasslist.txt” in the PRIDE upload for this project), Ion Types for modification with b and y enabled and mass fragment charge “2”. Generate Spectral Library was disabled. The FragPipe workflow with these parameters has been deposited in the PRIDE upload as “offset_final.workflow”. For duplicates, both runs were analysed as the same experiment.

For downstream data analysis, the two “*.tsv” files for the two experiments were individually processed. They were filtered to retain only entries that are present in the “psm.tsv” file, which contains the PSMs filtered by 1% PSM- and protein-level FDR. The column “best locs” indicates the possible residues modified by the mass offset. MSFragger puts the mass offset on each residue one-by-one and calculates hyperscores. The residues with the highest hyperscore are indicated by lower-case letters. Only entries were retained that were localized to a unique residue as seen by containing one lower-case letter (If there is no lower-case letter, the score for the unmodified peptide was higher than that for the best modified peptide and therefore no localization was performed). Next, the entries were filtered for a delta score > 1, where delta score is the difference of the highest hyperscore and the second highest hyperscore during the localization. For each entry, the UniProt Code was isolated from the column “Protein” and the full protein sequence was linked into the table. Based on this information, all peptide sequences that do not occur exactly once in the identified protein were excluded and the residue number of the modified residue was determined. Next, the amino acid at the modified residue was determined. If the protein *N*-terminus (modification at amino acid 1 or amino acid 2 if amino acid 1 is not present in the peptide (clipping of *N*-terminal methionine)) or the protein *C*-terminus (last amino acid of the protein) were modified, this was only counted and labelled as modification of the terminus and not of the respective amino acid at that position. For each entry, an identifier was generated in the format “UniProtCode”_X_”residue number”, where X is the one letter code of the modified amino acid or “N-terminal” or “C-terminal” for terminal modifications. Duplicates of entries with the same identifier were retained only once. The data of both experiments was then combined and only residues were counted in the final analysis that were present in both replicates. The fraction of all sites that was modified at each amino acid and the termini was reported. In some cases, amino acids with the same reactive group (D+E, N+Q and S+T) were clustered together. Letter plots were generated manually by scaling the letters with the percentage of modified sites modified at the respective amino acid. Here, all amino acids that were labelled to less than 5% were clustered as “others”.

If the peptides identified by a Mass Offset Search were quantified, the FragPipe analysis was run with the same parameters, but for duplicates, both runs were analysed as different experiments. For downstream data analysis, the “ion_label_quant.tsv” files of the two experiments were analysed separately. The full protein sequence was linked into the table. Based on this information, all peptide sequences that do not occur exactly once in the same protein were excluded. The “Identifier” was generated in the format “UniProtCode”_”residue number of the first amino acid in the peptide”_”residue number of the last amino acid in the peptide”. For each “Identifier”, the averaged “Log2 ratio HL”, which is the $\log_2$ transformed ratio of heavy and light ions, was determined as average of the “Log2 ratio HL” of all corresponding ions weighted with the “Total intensity” of the ion, which was calculated as the sum of “Light Intensity” and “Heavy Intensity” for each ion. The value was disregarded if the standard deviation of the “Log2 ratio HL” values for all ions of the same “Identifier” was > 1.41. Furthermore, for each “Identifier” the “Total Intensity”, “Total Light Intensity” and “Total Heavy Intensity” were calculated as the sum of all “Total Intensity”, “Light Intensity” and “Heavy Intensity” values of the individual ions, respectively. For all identifiers, the data for both replicates was now combined into one table. The “Total Intensity”, “Total Light Intensity” and “Total Heavy Intensity” was calculated as the sum of all “Total Intensity”, “Total Light Intensity” and “Total Heavy Intensity” values for all replicates, respectively. The “Log2 ratio HL” values for the replicates were named “Log2 ratio HL replicate 1” and “Log2 ratio HL replicate 2”. The average of these two values was calculated and named “Log2 ratio HL”. The value was disregarded, if the standard deviation between the replicates was > 1.41 or if the identifier was only quantified in one of the replicates. The “Log2 ratio HL” data for all “Identifiers” was plotted as a violin plot with all individual values shown. The expected value for the ratio ($\log_2(R) = 0$ for a 1:1 mixture of heavy and light samples as well as the preferred quantification window ($-1 < \log_2(R) < 1$ for a 1:1 mixture of heavy and light samples) were indicated by solid and dashed lines, respectively.

Quantification with FragPipe

To quantify specific amino acids a Closed Search was performed in MSFragger. For this purpose, the following settings were used in MSFragger: Precursor mass tolerance −50 to 50 ppm, fragment mass tolerance 20 ppm, Calibration and Optimization "None", Isotope Error "0/1/2", enzyme name "trypsin", cut after "KR", but not before "P", cleavage "enzymatic", missed cleavages "2", Clip N-term N enabled, peptide length 7 to 50, peptide mass range 500 to 5000 Da, no fixed modifications, no mass offsets, all other options were left at the standard settings. Variable modifications were set to the masses indicated in Supplementary Table 6 on the indicated amino acid(s) indicated in that table with max. 1 occurrence (Here, all pairs of modifications were investigated separately). If modification of C was investigated, this was performed in a separate run and a variable modification of 57.02146 Da on C with max. 3 occurrences was additionally set alongside no fixed modifications. Modifications on all other amino acids were analysed simultaneously in the same run with no additional variable modifications and a fixed modification of 57.02146 Da set on C. Crystal-C was disabled. PeptideProphet was run with the following settings: "--decoyprobs --ppm --accmass --nonparam --expectscore". PTMProphet was disabled. ProteinProphet was run with the following settings: "--maxppmdiff 2000000". Generate report was enabled with the following settings: "--sequential --razor --mapmods --prot 0.01". Run MS1 quant was enabled with the following settings: IonQuant enabled, M/Z Window "10 ppm", RT Window "0.4 min", Labelling based quant with the masses indicated in Supplementary Table 6 on the amino acid(s) indicated in that table (If *N*-terminal or *C*-terminal modification was investigated, labelling was allowed on any amino acid as indicated by "*".), Re-quantify enabled, Top N ions "3" Min freq. "0.5", Min exps "1", Min isotopes "2", Normalize disabled. Run TMT-Integrator was disabled. PTM-Shepherd was disabled. Generate Spectral Library was disabled. The FragPipe workflows with these parameters for quantification of cysteine residues and all other amino acids have been deposited in the PRIDE upload as "closed_C_final.workflow" and "closed_final.workflow", respectively. For duplicates, both runs were analysed as different experiments.

For downstream data analysis, the "ion_label_quant.tsv" files of the two experiments were analysed separately. For each entry, the "Modified peptide" was generated as either the "Light Modified Peptide" or the "Heavy Modified Peptide" based on the entry with the higher "PeptideProphet Probability". The masses of probe modification in the "Modified Peptide" were replaced by an "*" and the mass of carbamidomethylation ([57.0215]) in this entry was deleted, if present. The full protein sequence was linked into the table. Based on this information, all peptide sequences that do not occur exactly once in the same protein were excluded and the residue number of the modified residue was determined. The "Identifier" was generated in the format "UniProtCode"_X_"residue number", where X is the one letter code of the modified amino acid or "N-term" or "C-term" for terminal modifications. For each "Identifier", the averaged "Log2 ratio HL", which is the $\log_2$ transformed ratio of heavy and light ions, was determined as average of the "Log2 ratio HL" of all corresponding ions weighted with the "Total intensity" of the ion, which was calculated as the sum of "Light Intensity" and "Heavy Intensity" for each ion. The value was disregarded if the standard deviation of the "Log2 ratio HL" values for all ions of the same "Identifier" was > 1.41. Furthermore, for each "Identifier" the "Total Intensity", "Total Light Intensity" and "Total Heavy Intensity" were calculated as the sum of all "Total Intensity", "Light Intensity" and "Heavy Intensity" values of the individual ions, respectively. If several different "Modified peptides" were detected for the same "Identifier", the "Modified Peptide" and "Peptide Sequence" with the shortest sequence were kept. For all identifiers, the data for both replicates was now combined into one table. If different "Modified peptides" were detected for the same "Identifier" in the different replicates, the "Modified Peptide" and "Peptide Sequence" with the shortest sequence were kept. The "Total Intensity", "Total Light Intensity" and "Total Heavy Intensity" was calculated as the sum of all "Total Intensity", "Total Light Intensity" and "Total Heavy Intensity" values for all replicates, respectively. The "Log2 ratio HL" values for the replicates were named "Log2 ratio HL replicate 1" and "Log2 ratio HL replicate 2". The average of these two values was calculated and named "Log2 ratio HL". The value was disregarded, if the standard deviation between the replicates was > 1.41 or if the identifier was only quantified in one of the replicates. The "Log2 ratio HL" data for all "Identifiers" was plotted as a violin plot with all individual values shown. If several amino acids were quantified, the data was visualized for each amino acid individually. The expected value for the ratio ($\log_2(R) = 0$ for a 1:1 mixture of heavy and light samples, $\log_2(R) = 2$ for a 4:1 mixture of heavy and light samples) as well as the preferred quantification window ($-1 < \log_2(R) < 1$ for a 1:1 mixture of heavy and light samples, $1 < \log_2(R) < 3$ for a 4:1 mixture of heavy and light samples) were indicated by solid and dashed lines, respectively.

Quantification with MaxQuant

Quantification with MaxQuant[14] was performed analogously to a published procedure utilizing a workaround to also allow quantification of peptides that contain one or more carbamidomethylated cysteines in addition to the isoDTB tag-labelled cysteine.[10] Briefly, in this workaround "U", which normally stands for selenocysteine, is used as a placeholder amino acid for the modified cysteine. To accomplish this, all selenocysteine-containing proteins were deleted from the FASTA databases; which were usually very few. Afterwards, each cysteine in the FASTA database

was individually replaced with a "U" generating n different sequences with a single "U" for a protein with n cysteines. For each individual replacement, an entry in the FASTA database was created, which was named in the format "UniProt code"_"C""number of the cysteine". The unmodified sequence was deleted from the FASTA database, except if the protein did not contain any cysteine, in which case the unmodified entry was renamed to "UniProt Code"_"C0" and kept in the database. In this way, for each cysteine in the database, a unique sequence was created, in which it was marked as the modified cysteine (by being replaced by the placeholder "U") and all other cysteines were marked as unmodified (were remaining "C" in the database). In this way, there was always only one modified cysteine in each peptide to be detected and quantified.

MS raw data were analysed using MaxQuant software (version 1.6.17.0).[14] Standard settings were used with the following changes and additions: The modified FASTA databases with individual substitutions of cysteines with the placeholder "U" were used. Labels were set on the placeholder amino acid "U" for the light isoDTB tag as light label ($C_{28}H_{46}N_{10}O_6S_1Se_{-1}$) and the heavy isoDTB tag as heavy label ($C_{24}{}^{13}C_4H_{46}N_8{}^{15}N_2O^6S_1Se_{-1}$). A multiplicity of 2 was set and a maximum number of labelled amino acids of 1. The digestion enzyme was set to Trypsin/P with a maximum number of missed cleavages of 2. No variable modifications were included. The "Re-quantify" option was enabled. Carbamidomethyl (C_2H_3NO) was used as fixed modification on cysteine. Contaminants were included. Peptides were searched with a minimum peptide length of 7 and a maximum peptide mass of 4,600 Da. "Second peptides" and "Dependent peptides" were disabled and the option "Match between run" was enabled with a Match time window of 0.7 min and an alignment window of 20 min. An FDR of 0.01 was used for Protein FDR, PSM FDR and XPSM FDR.

The "peptides.txt" file of the MaxQuant analysis was used for further analysis. All peptide sequences without a modified cysteine (placeholder "U") and all reverse sequences were deleted. Only the columns "Sequence", "Leading Razor Protein", "Start Position" and the columns for "Ratio H/L" for both replicates were kept. The "Leading Razor Protein" was renamed to the UniProt Code without the indicator for the number of the cysteine. All individual ratios were filtered out if they were "NaN" and all other values were transformed into the $\log_2$-scale. For each peptide, the data was filtered out, if it was not present in both replicates or if the standard deviation between the replicates exceeded a value of 1.41. For each peptide, an identifier was generated in the form "UniProt Code"_"C""residue number of the modified cysteine". The data for the same replicate for all peptides with the same identifier, and therefore the same modified cysteine, were combined. Here, the median of the data was used. The data was filtered out if the standard deviation exceeded a value of 1.41. Each modified cysteine was kept in the dataset once with the shortest peptide sequence as the reported sequence. For each modified cysteine, the values of the two replicates were combined, but the individual values were also reported. The values were combined as the median and the data was filtered out if there was only data in one of the replicates or if the standard deviation exceeded a value of 1.41. These are the final values that are reported.

Quantification with pFind 3

pFind 3 was used in the version pFind 3.1.5[15] and data analysis was performed analogous to a published procedure.[16] The downloaded and adjusted FASTA database (described under "General setup of analysis software") without added reverse sequences was used and the decoys were generated using pConfig.exe. Using this tool, 12C was added as an additional element with the settings "12.000000,98.89%;13.003355,1.11%;". A modification named "IA_C_light_isoDTB" was added with the composition "C(24)H(46)N(8)O(6)12C(4)14N(2)" with the settings Position "Anywhere", Sites "C" and Common enabled. "isoDTB=M:IA_C_light_isoDTB{12C,13C}M:IA_C_light_isoDTB{14N,15N}" was added as a new line to the "quant.ini" and the value for "@NUMBER_LABEL" in this file adjusted accordingly.

Data for the individual replicates was analysed independently starting from the raw file and the default settings for "MS Data" were used including MS instrument "HCD-FTMS". "Trypsin KR_C" was set as protease with the setting "Full-Specific" and up to 3 missed cleavage sites. A precursor tolerance of 10 ppm and a fragment tolerance of 20 ppm were set. Open Search was disabled. "IA_C_light_isoDTB" and "Carbamidomethyl[C]" were set as variable modifications and all other parameters were left at standard settings. For quantification, "Labelling-SILAC" was set with a multiplicity of 2. "isoDTB" was set as heavy label and all other options were left at standard settings.

The post-processing algorithm was downloaded ("https://github.com/morpheusliu/Post-processing-program-for-pFind3-results") and Python (version: 3.7.3) was installed. The files "construct_library.py" and "pfind_post_processing_site_level.py" were combined in the same folder. A "database.txt" file was generated in this folder and the data from the used FASTA was copied into this file. "construct_library.py" was executed. A subfolder "data" was generated and the "pQuant.spectra.list" file was copied into this folder. This file was renamed to "IA_C_light_isoDTB_experimentname" without a file extension. A subfolder "data_protein" was generated and the "'pFind.protein'" file was copied into this folder. This file was renamed to "IA_C_light_isoDTB_experimentname_P" without a file extension. "pfind_post_processing_site_level.py" was executed with the parameters "hl", "0.5" and "a".

The “site_experimentname” file was further analysed. For each entry an “Identifier” was generated in the format “UniProt Code”_”C””residue number of the modified cysteine” based on the columns “AC” and “Site”. The “Modified sequence” was determined as the shortest peptide in the column “Modified sequences”. For all “Identifiers”, the data for both replicates for “Ratio hl” was now combined into one table. The shortest “Modified Sequence” was kept. The data was transformed to the log2 scale and the two columns were named “log2 ratio hl replicate 1” and “log2 ratio hl replicate 2”. For each modified cysteine, the values of the two replicates were combined, but the individual values were also reported. The values were combined as the median and the data was filtered out if there was only data in one of the replicates or if the standard deviation exceeded a value of 1.41. These are the final values that are reported.

Analysis of peptides with multiple probe modifications using FragPipe

To analyse the occurrence of peptides with more than one modification with the probe, a closed search was performed in MSFragger. For this purpose, the same parameters were used as described under “Quantification with FragPipe” with the exception that two variable modifications with **IA-alkyne** clicked to the light (618.3602 Da) or heavy (624.3677 Da) isoDTB tag, one variable modification with the unclicked probe (137.0841 Da), one variable modification by carbamidomethylation (57.02146 Da), a maximum of two total variable modifications and no fixed modifications were allowed. For duplicates, both runs were analysed as the same experiment.

For downstream data analysis, the “psm.tsv” file was used. This was filtered to only contain PSMs with at least one modification with **IA-alkyne** clicked to either the light or the heavy isoDTB tag. PSMs that contained both a modification with **IA-alkyne** clicked to the light isoDTB tag and a modification with **IA-alkyne** clicked to the heavy isoDTB tag were filtered out. For each PSM, the number of modifications with **IA-alkyne** clicked to the isoDTB tag and the number of modifications with **IA-alkyne** not clicked to the isoDTB tag were counted. Based on this data, the number of PSMs containing only one modification with **IA-alkyne** clicked to the isoDTB tag, the number of PSMs containing two such modifications and the number of PSMs containing both one modification with **IA-alkyne** clicked to the isoDTB tag and one modification with unclicked **IA-alkyne** were counted and reported.

Data analysis with pChem

pChem[1] was used in the version pChem1.1. pChem was run by executing “pChem.exe” using the following configuration in the “pChem.cfg” file:

```
# If isotope coding is adopted to facilitate the discovery of unknown modifications (True or False)
isotope_labeling=True

# Path to the output file
output_path=C:\pChem_output

# Path to the protein sequence database
fasta_path=C:\MQ_fastas\SA59.fasta

# Format of MS data, RAW or MZML
msmstype=RAW

# The number and path of MS data
msmsnum=2
msmspath1=C:\pChem_data\181228_SMH_181207_P5.raw
msmspath2=C:\pChem_data\181228_SMH_181207_P6.raw


# Type of MS dissociation method
activation_type=HCD-FTMS

# Usage of open search (True/ False), against Unimod, the common modification can be set if not
open_flag=False
common_modification_number=2
common_modification_list=Carbamidomethyl[C];Oxidation[M];


# Mass tolerance of the mass shift between light isotope and heavy isotope
mass_of_diff_diff=6.00749


# Isotopic mass difference within empirically defined tolerance(Da)
mass_diff_diff_range=0.005
```

# Mass range of unknown modification (Da)
min_mass_modification=200
max_mass_modification=1000

# Isotopic pairs of mass shifts with PSMs less than X% of that of overall PDMs were neglected
filter_frequency=5

# If consider the N-side or C-side for amino acid localization (True or False)
side_position=True

# P-value threshold enabling confident amino acid localization
p_value_threshold=0.001

# If report the statistical information (True or False)
report_statistics=True

The selectivity was directly reported based on the “pChem.summary” file.

Generation of sequence logos with pLogo

The data of the Mass Offset Searches in FragPipe was used as a basis for the generation of pLogos.[17] For each modified site, the peptide sequence starting from ten amino acids before the modified site to ten amino acids after the modified site was generated based on the identifier. If the modified amino acid was closer to the *N*- or *C*-terminus, the placeholder “X” was repeatedly added in front or after the sequence, respectively, to fill up the sequence to ten letters before and after the modified amino acid. Analogously, ten “X” were added in front and after every entry in the corresponding FASTA database. This database was used as background. All sequences around modified sites were pasted into the pLogo webserver interface (https://plogo.uconn.edu/) and the corresponding background was loaded. After generation of the pLogo, the colors were adjusted. pLogos were exported in the png format either without any fixed amino acid or with the indicated amino acid fixed at position 0. If the amino acid, for which a sequence logos should be generated, was not enriched, only the sequences with this amino acid at position 0 were loaded and a pLogo generated with this amino acid fixed at position 0. For *N*-terminal and *C*-terminal modification, only the sequences modified at the *N*- or *C*-terminus, respectively, were loaded and the pLogo generated. For *C*-terminal labelling “X” was fixed at position 1. For *N*-terminal labelling without clipping of the *N*-terminal methionine, “X” was fixed at position −1. For *N*-terminal labelling after clipping of the *N*-terminal methionine, “M” was fixed at position −1 and “X” was fixed at position −2.

Analysis of protein essentiality

Data on the essentiality of proteins in *S. aureus* SH1000 was derived from the parent strain NCTC8325. The data for the database of essential genes (DEG)[80] was used. It was downloaded from aureowiki (aureowiki.med.uni-greifswald.de). The information was linked to the information in the FASTA database through the “ordered locus name” that is reported in aureowiki and in UniProt.

Data availability

All raw data needed to reproduce our findings is the mass spectrometric data for all proteomic analyses and has been deposited to the ProteomeXchange Consortium (http://proteomecentral.proteomexchange.org) *via* the PRIDE partner repository[81] with the dataset identifier PXD024454 and PXD065811, where it is freely available. All evaluated data is freely available in the Source Data files for Figures 2-6 and in the Supplementary Tables 2-5.

The file names for the raw files give information on the date, when the mass spectrometric measurement was set up, the initials of the scientist to set up the experiment and the sample name in that order. The information, which data file corresponds to which experiment, can be found in Supplementary Table 7. Alongside the raw data files, all used FASTA files were also deposited (“HS0-2_plus_rev.fasta” and “SA59_plus_rev.fasta” for all FragPipe analyses, “SA59_CU.fasta” for MaxQuant analysis, “SA59.fasta” for pFind 3 analysis as well as “HS0-2_10X.fasta” and “SA59_10X.fasta” as background for pLogo generation). Furthermore, the FragPipe workflows (“open_final.workflow” for all Open Searches, “offset_final.workflow” for all Mass Offset Searches as well as “closed_C_final.workflow” for Closed Searches on cysteines and “closed_final.workflow” for closed searches on all other amino acids) and the reduced mass list for PTM-Shepherd (“reducedMasslist.txt”) were uploaded. For each experiment, a zip file was uploaded that contains the following information: For Open Searches, the FragPipe parameters (“open_fragger.params”) and the summary of all detected masses of modification (“open_global.modsummary.tsv”). For Mass Offset Searches, the FragPipe parameters (“offset_fragger.params”), the data tables for the two replicates (“offset_*experimentname*.tsv”) and the list of all PSMs after FDR filtering (offset_psm.tsv”). For Closed Searches,

the FragPipe parameters (“closed_*quantified amino acid*_fragger.params”), the FragPipe parameters (“closed_*quantified amino acid*_fragpipe_*date*_*time*.config) and the information on quantified ions in the two replicates (“closed_*quantified amino acid*_ion_label_quant_*experimentname*.tsv”). If two separate quantifications were performed for cysteine and the other amino acid(s), both sets of data files were uploaded. If more than one pair of masses of modification were analysed, the type of mass of modification that was analysed is indicated in the file name. For the datasets that were also analysed with MaxQuant (“MaxQuant_peptides.txt” and “MaxQuant_mqpar.xml”) and pFind3 (“pFIND3_site_hl_IA_C_light_*experimentname*”), the respective files were also uploaded.

NMR Spectra

2-Chloro-*N*-(hex-5-yn-1-yl)acetamide (**CA-alkyne**) (^{1}H, 500 MHz, $CDCl_3$):

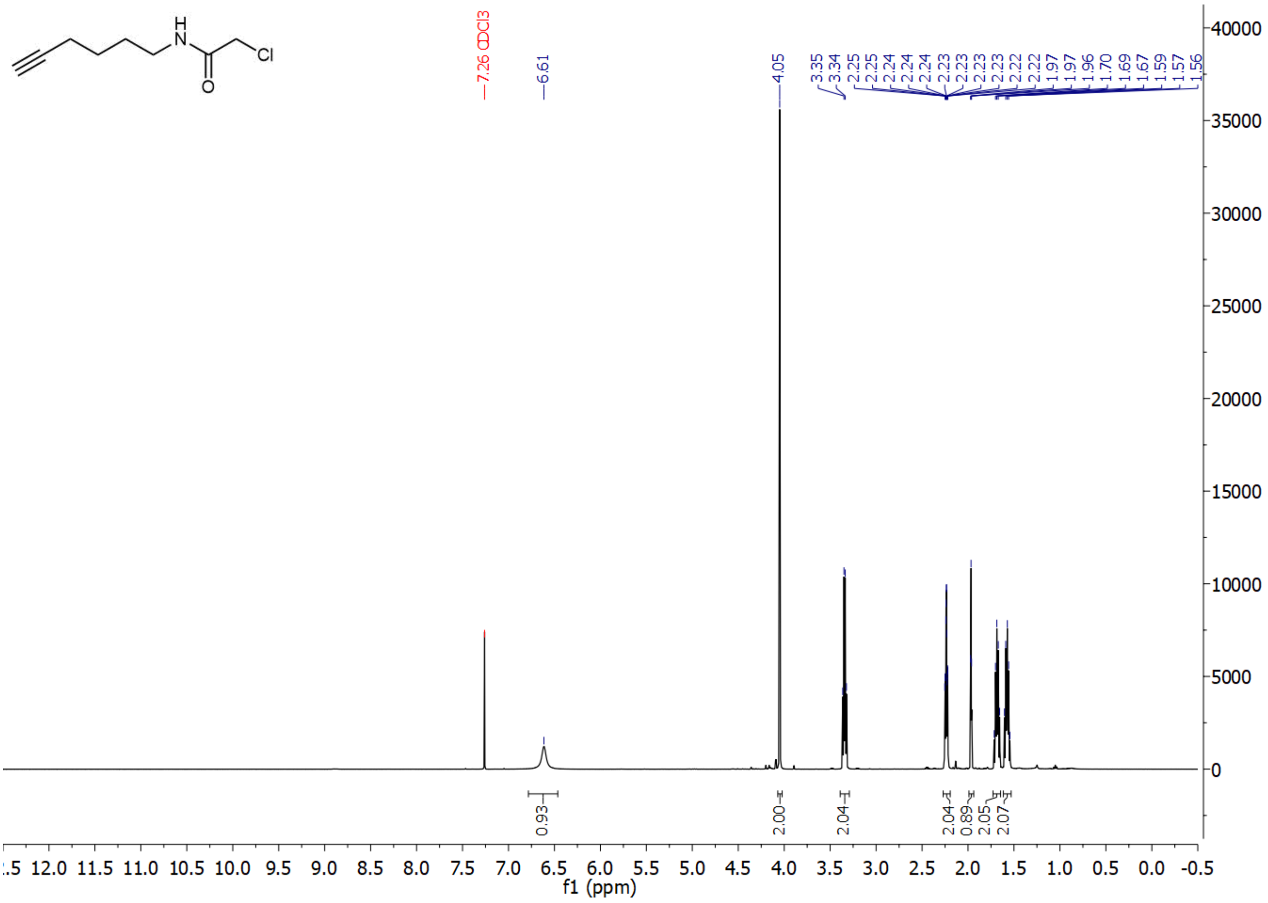

2-Chloro-*N*-(hex-5-yn-1-yl)acetamide (**CA-alkyne**) (^{13}C, 75.5 MHz, $CDCl_3$):

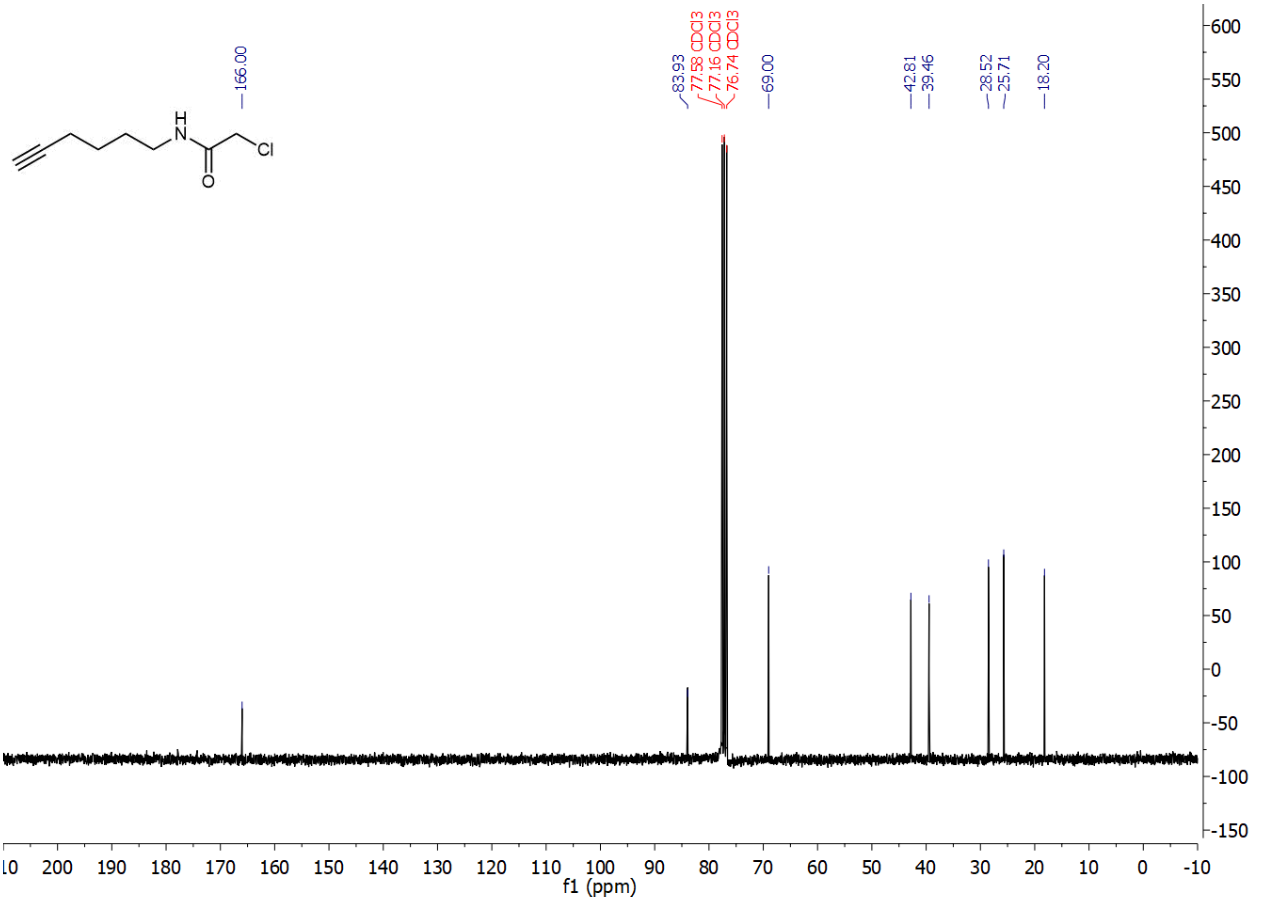

2-Iodo-*N*-(hex-5-yn-1-yl)acetamide (**IA-alkyne**) (^{1}H, 300 MHz, $CDCl_3$):

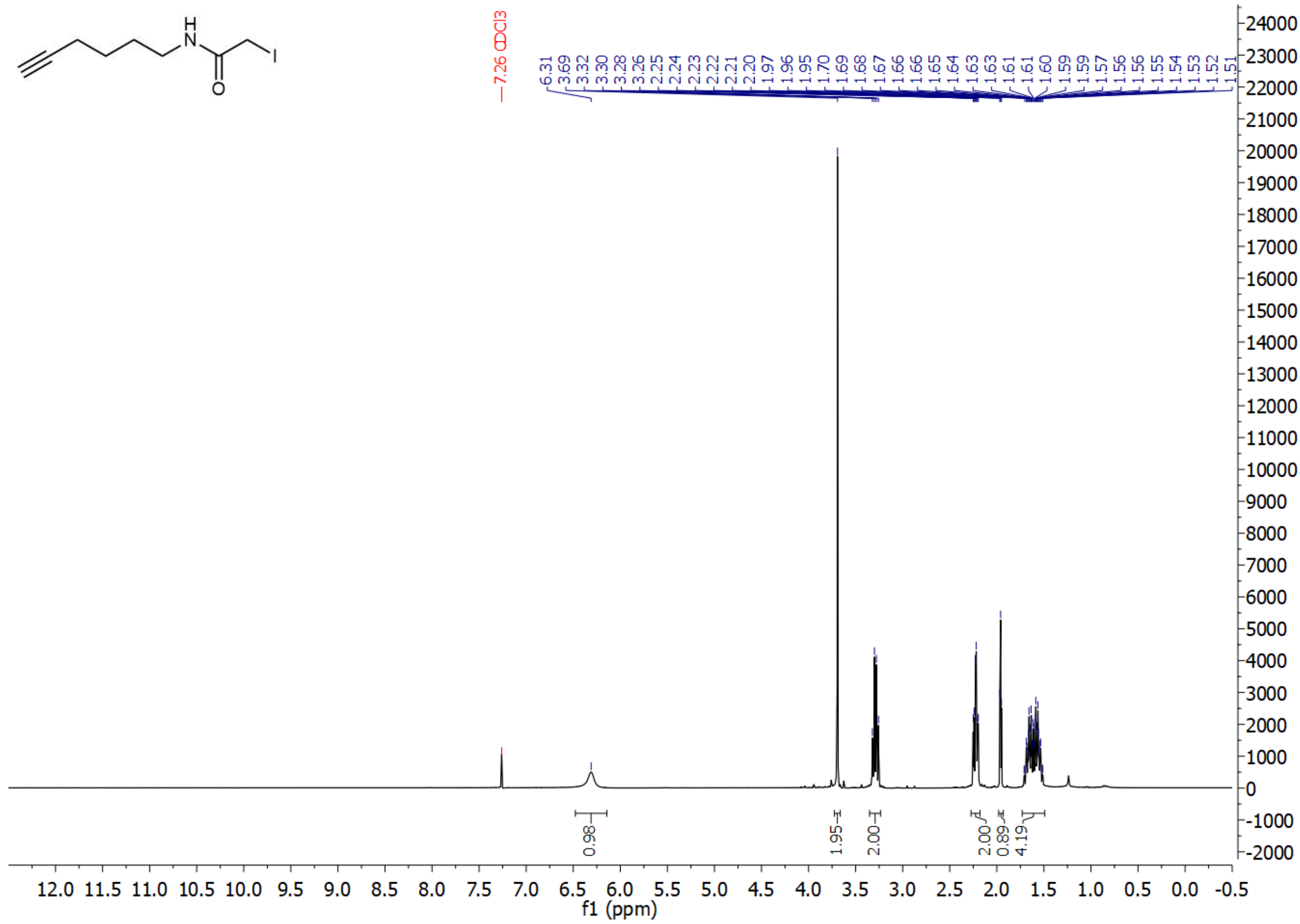

2-Iodo-*N*-(hex-5-yn-1-yl)acetamide (**IA-alkyne**) (^{13}C, 75.5 MHz, $CDCl_3$):

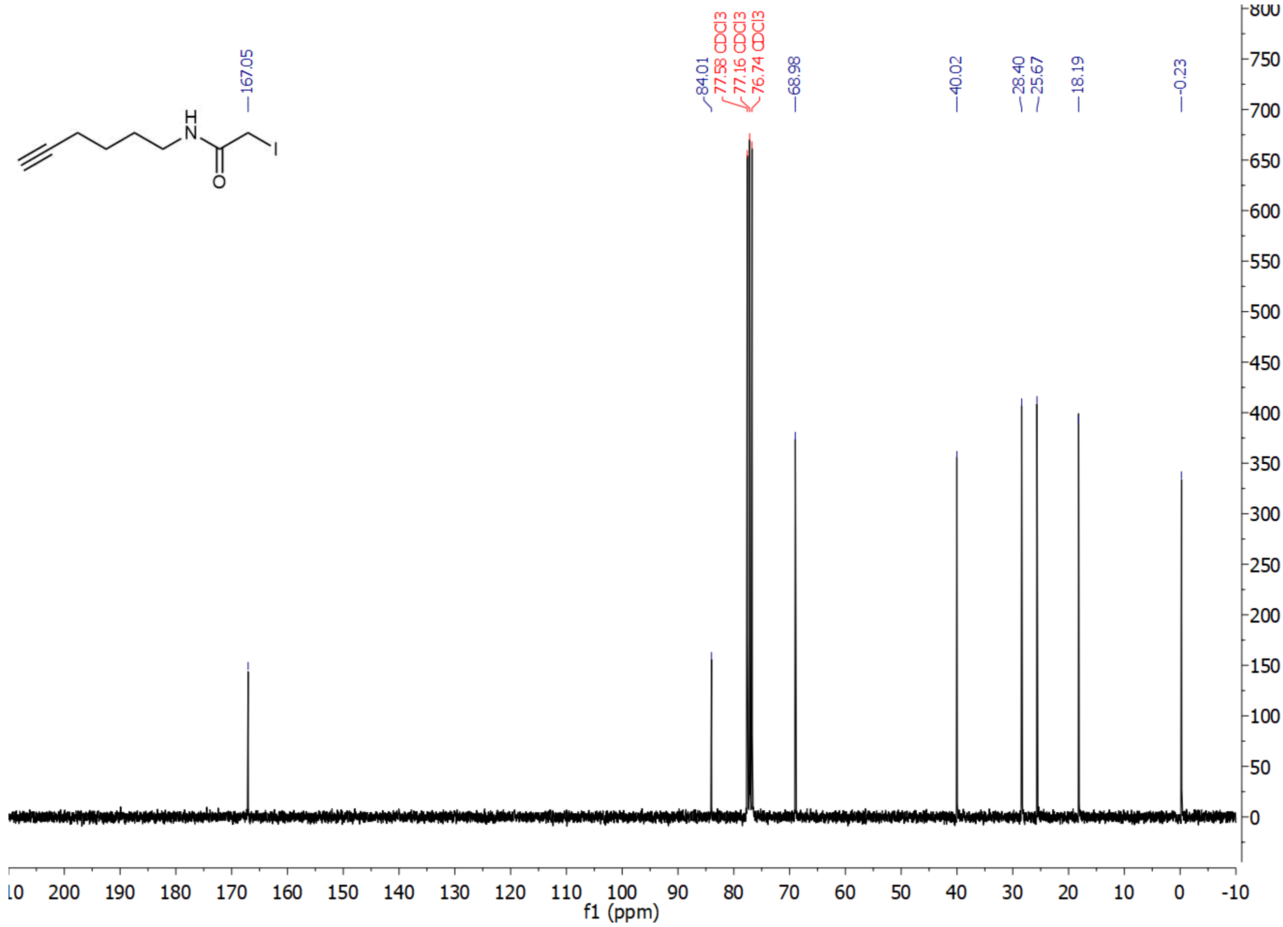

2-Chloro-*N*-(4-cyanobutyl)acetamide (**CA-nitrile**) (^{1}H, 300 MHz, $CDCl_3$):

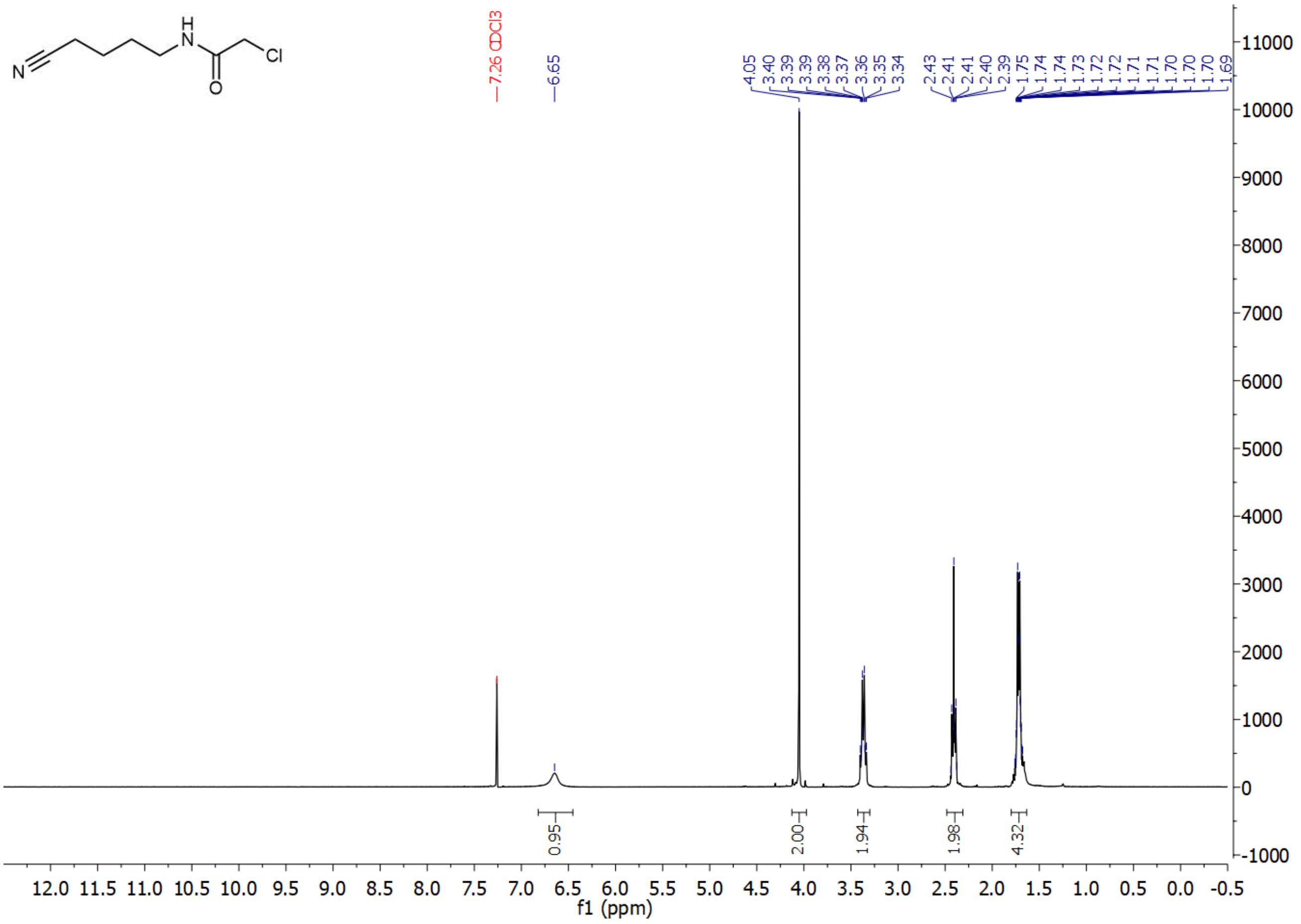

2-Chloro-*N*-(4-cyanobutyl)acetamide (**CA-nitrile**) (^{13}C, 75.5 MHz, $CDCl_3$):

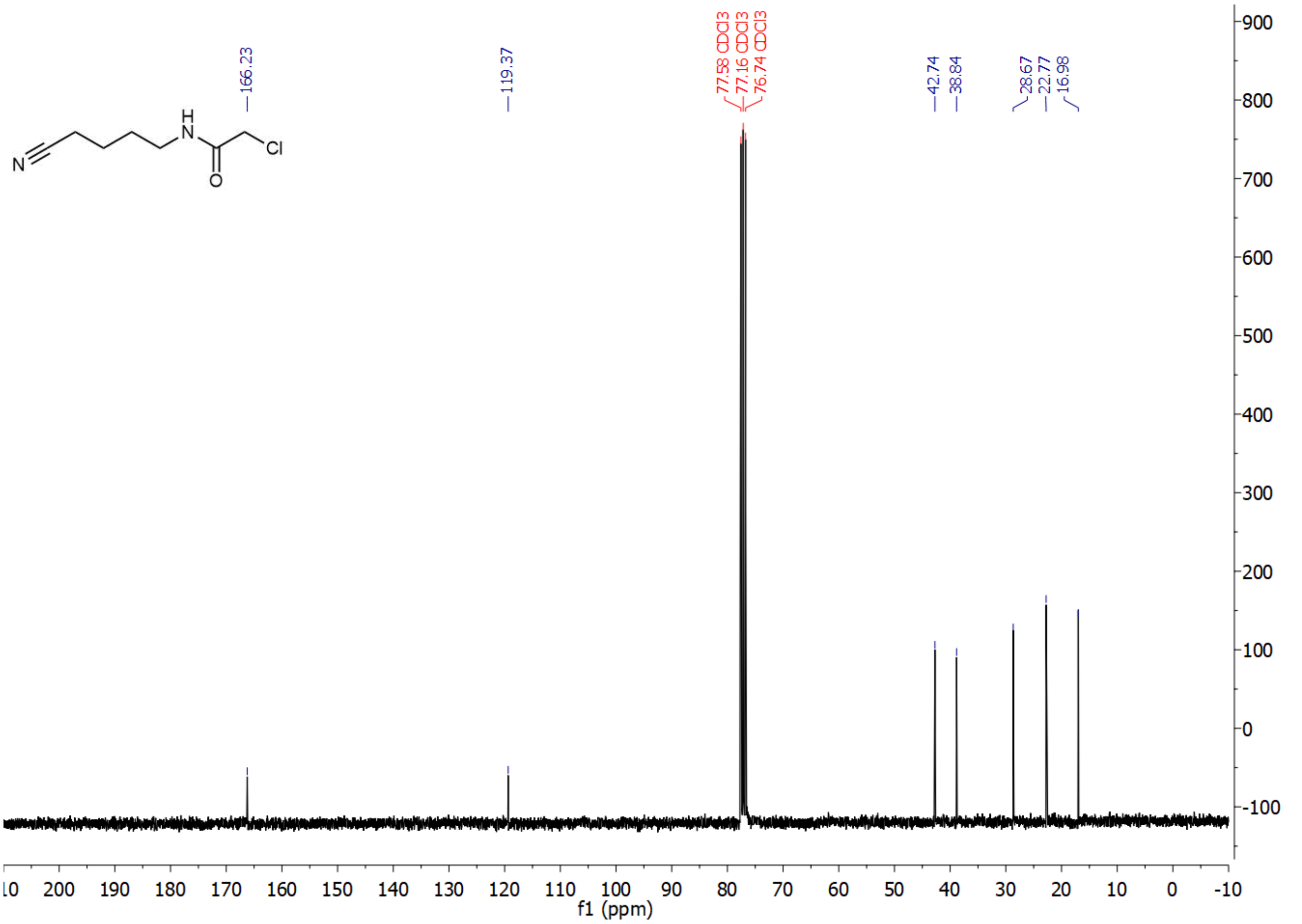

1-Chloro-7-(trimethylsilyl)hept-6-yn-2-one (**4**) (^{1}H, 400 MHz, $CDCl_3$):

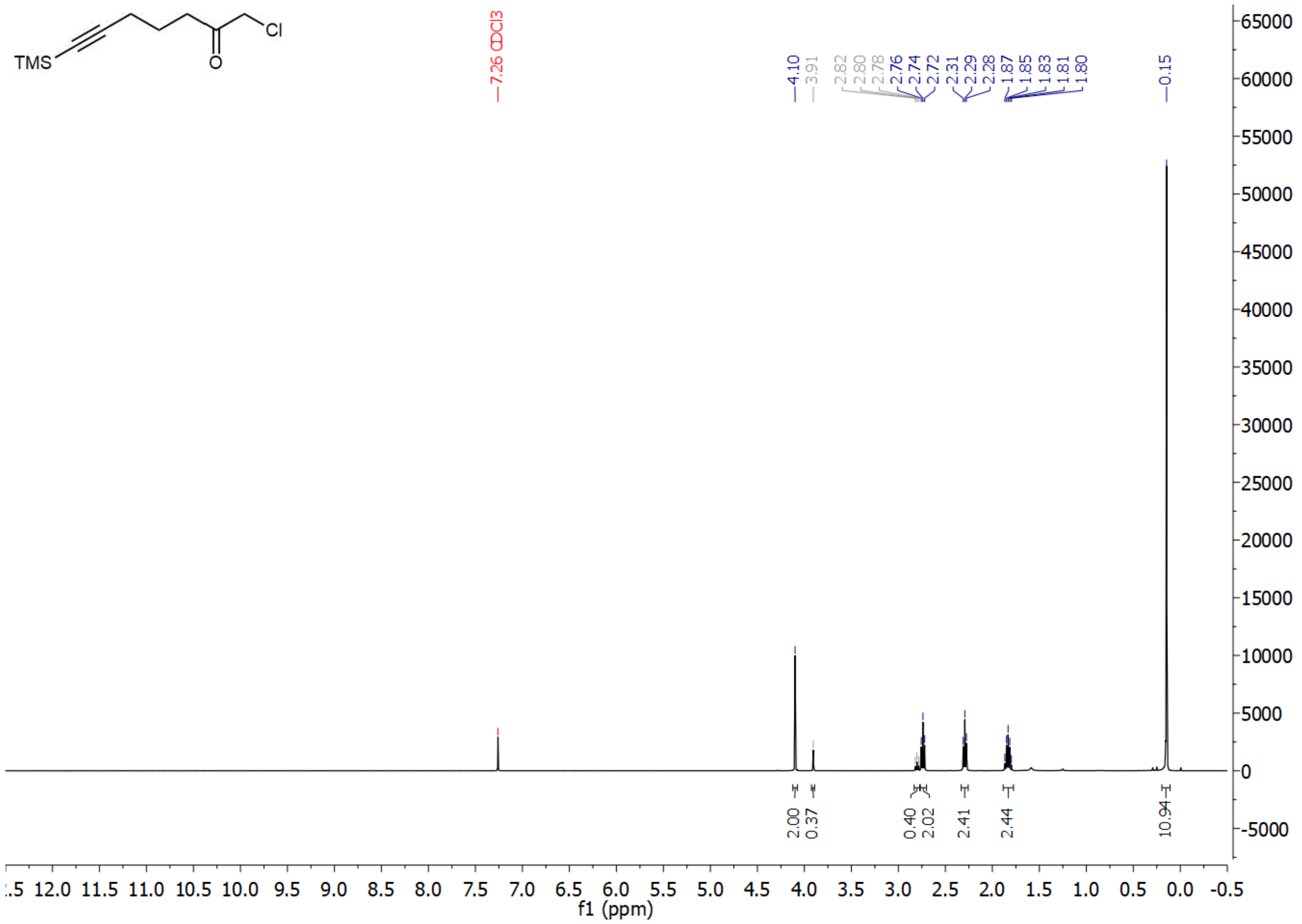

1-Chloro-7-(trimethylsilyl)hept-6-yn-2-one (**4**) (^{13}C-NMR, 101 MHz, $CDCl_3$):

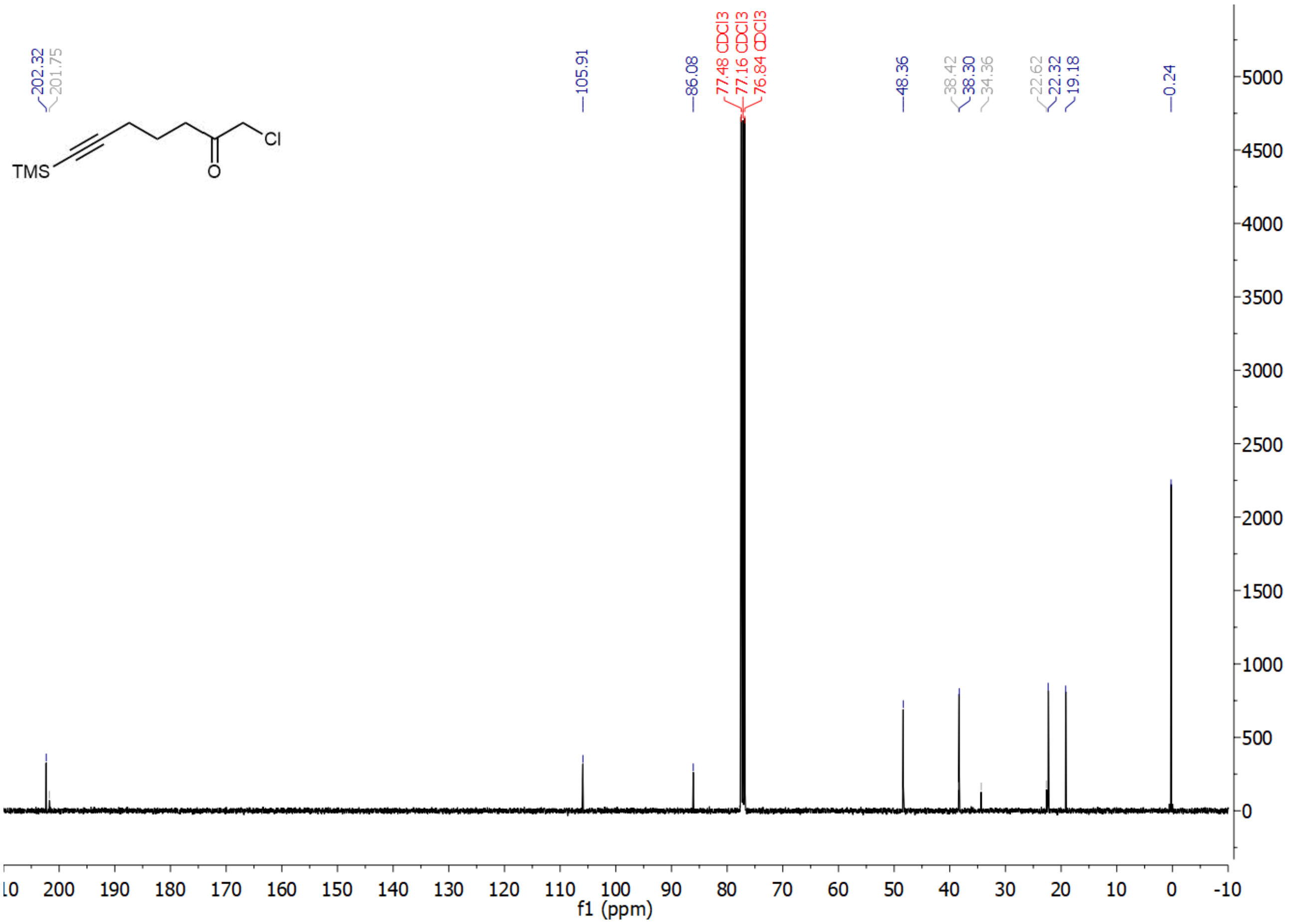

1-Bromo-7-(trimethylsilyl)hept-6-yn-2-one (**5**) (^{1}H, 400 MHz, $CDCl_3$):

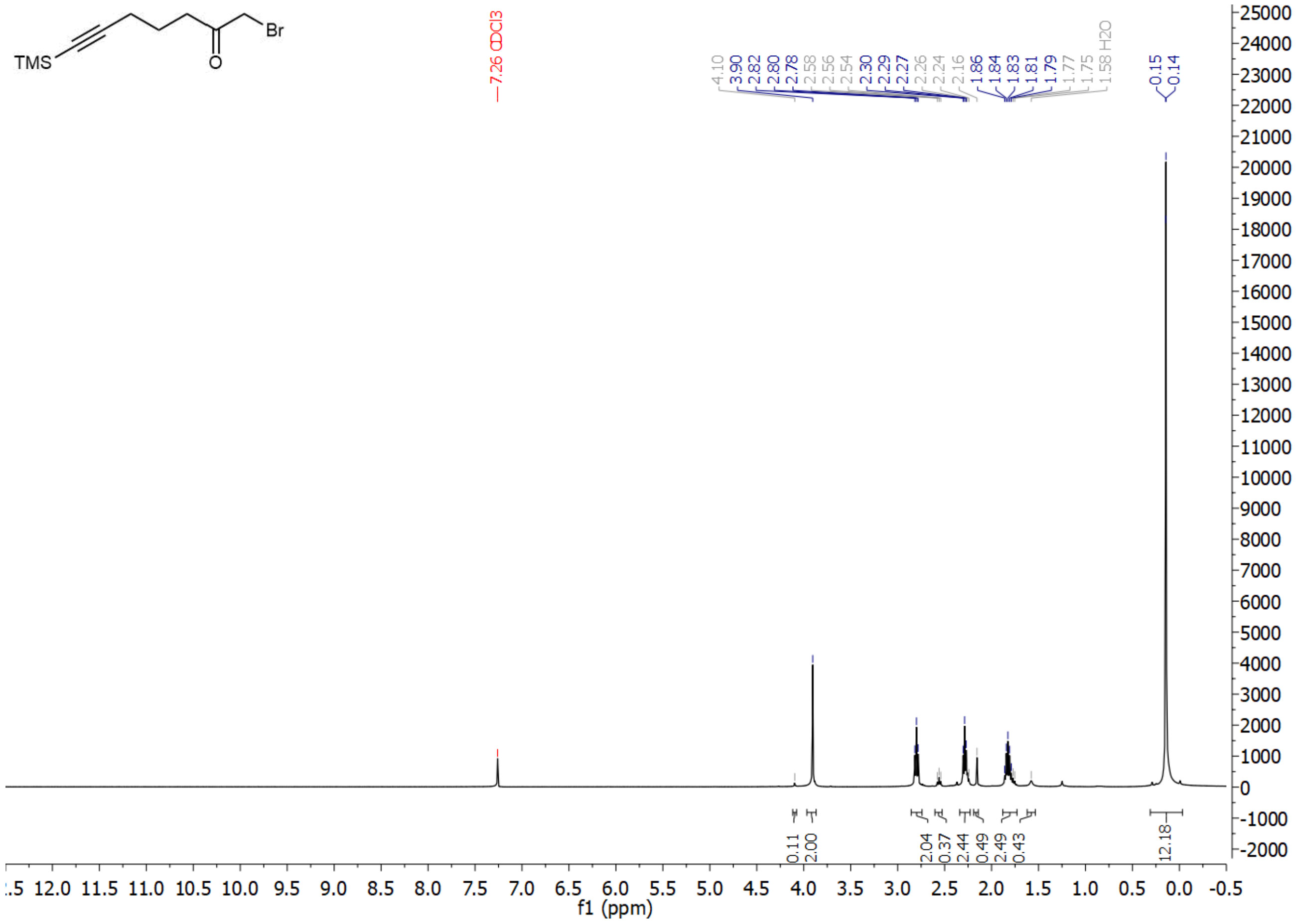

1-Bromo-7-(trimethylsilyl)hept-6-yn-2-one (**5**) (^{13}C-NMR, 101 MHz, $CDCl_3$):

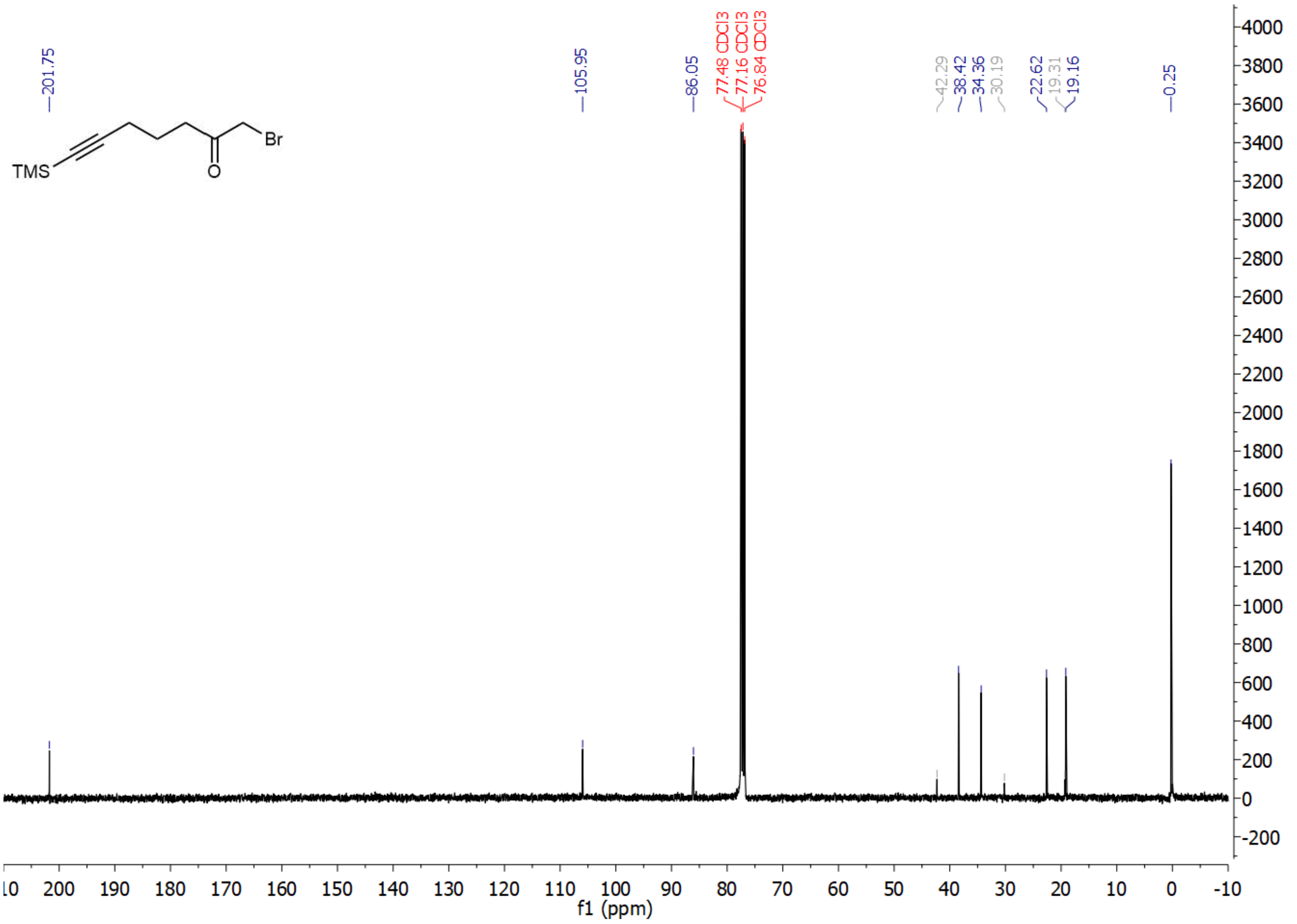

1-Bromohept-6-yn-2-one (**BMK-alkyne**) (^{1}H, 300 MHz, $CDCl_3$):

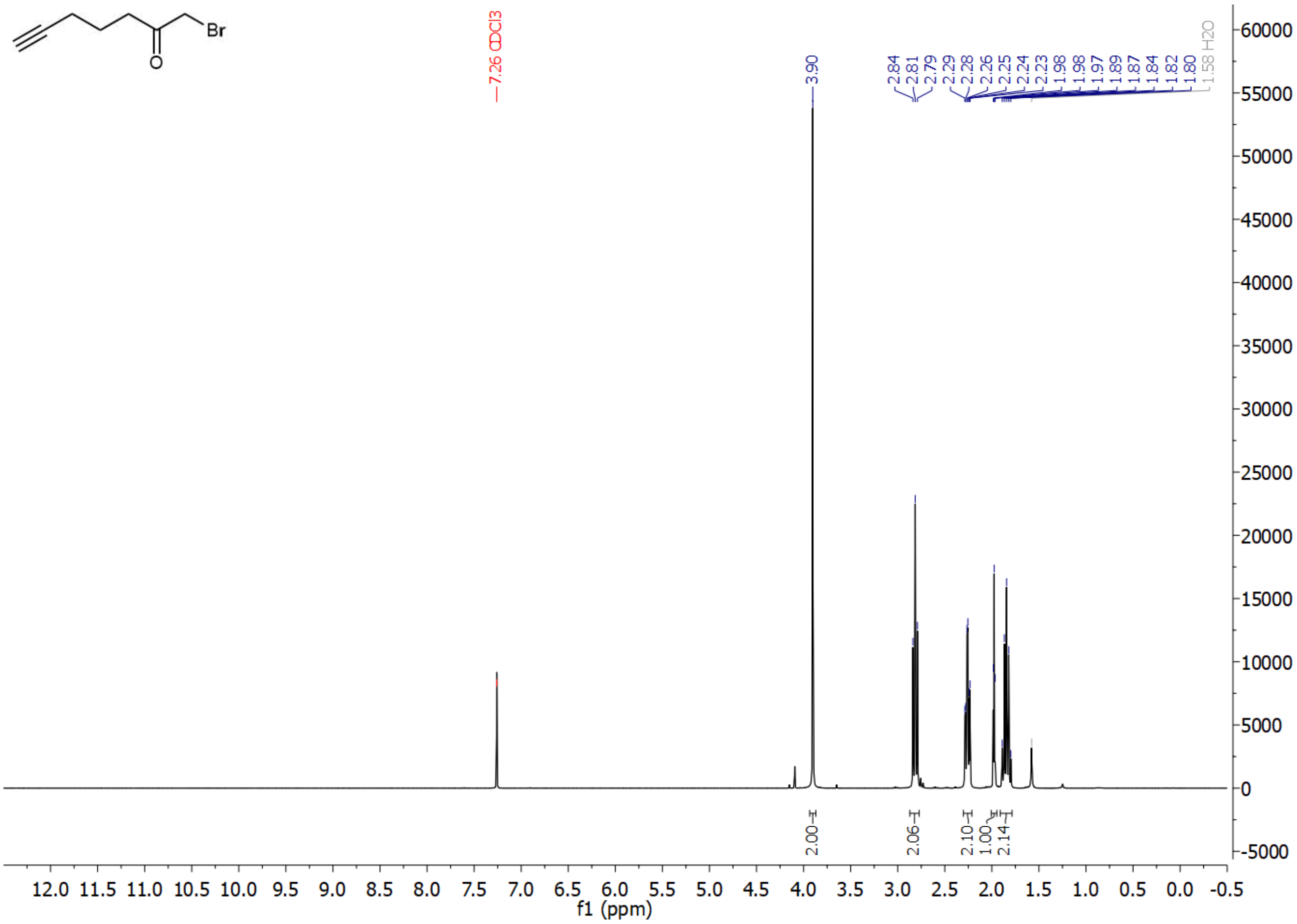

1-Bromohept-6-yn-2-one (**BMK-alkyne**) (^{13}C,101 MHz, $CDCl_3$):

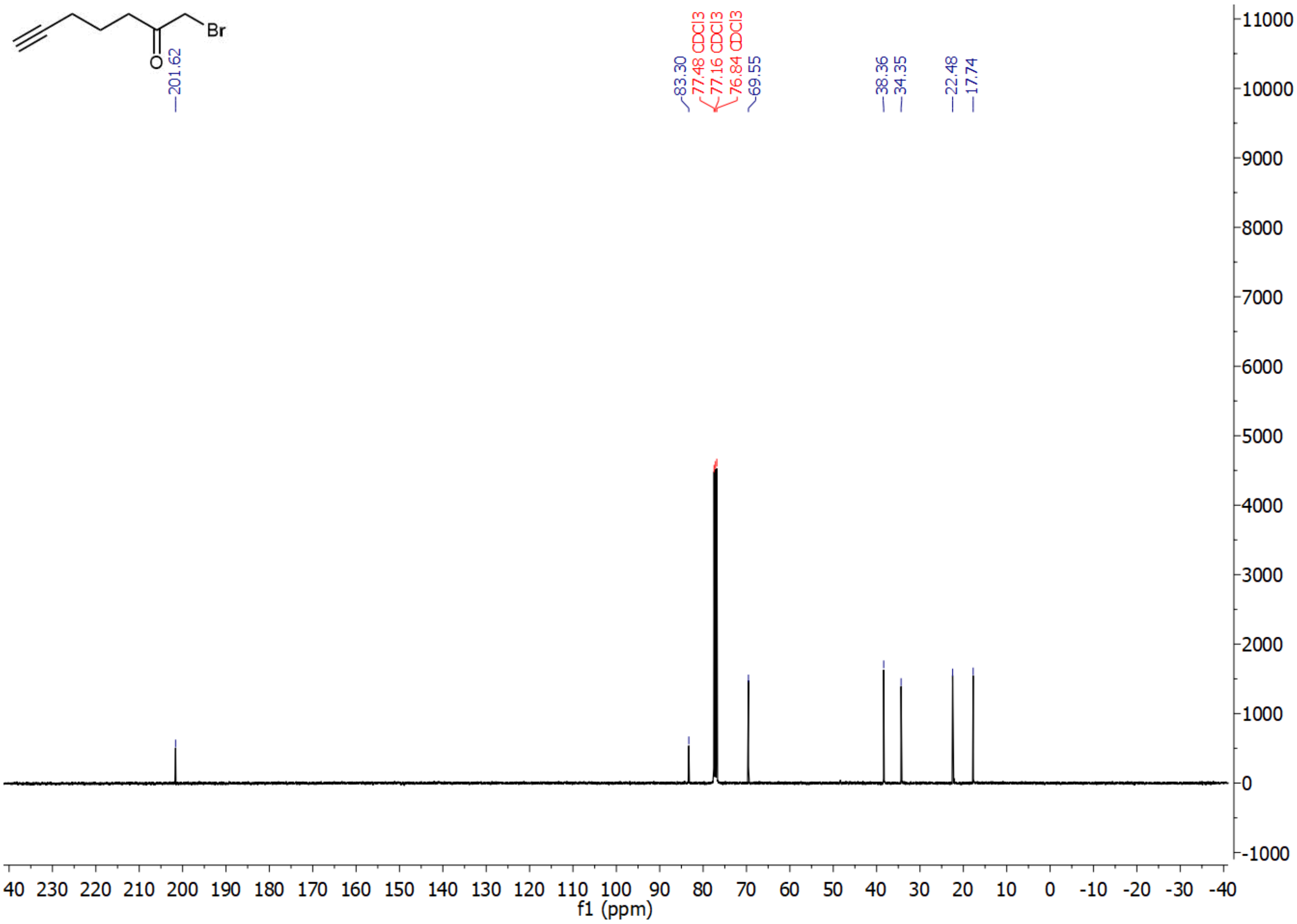

N-(But-3-yn-1-yl)-2,3,4,5,6-pentafluorobenzenesulfonamide (**PFPSA-alkyne**) (^1H, 400 MHz, $CDCl_3$):

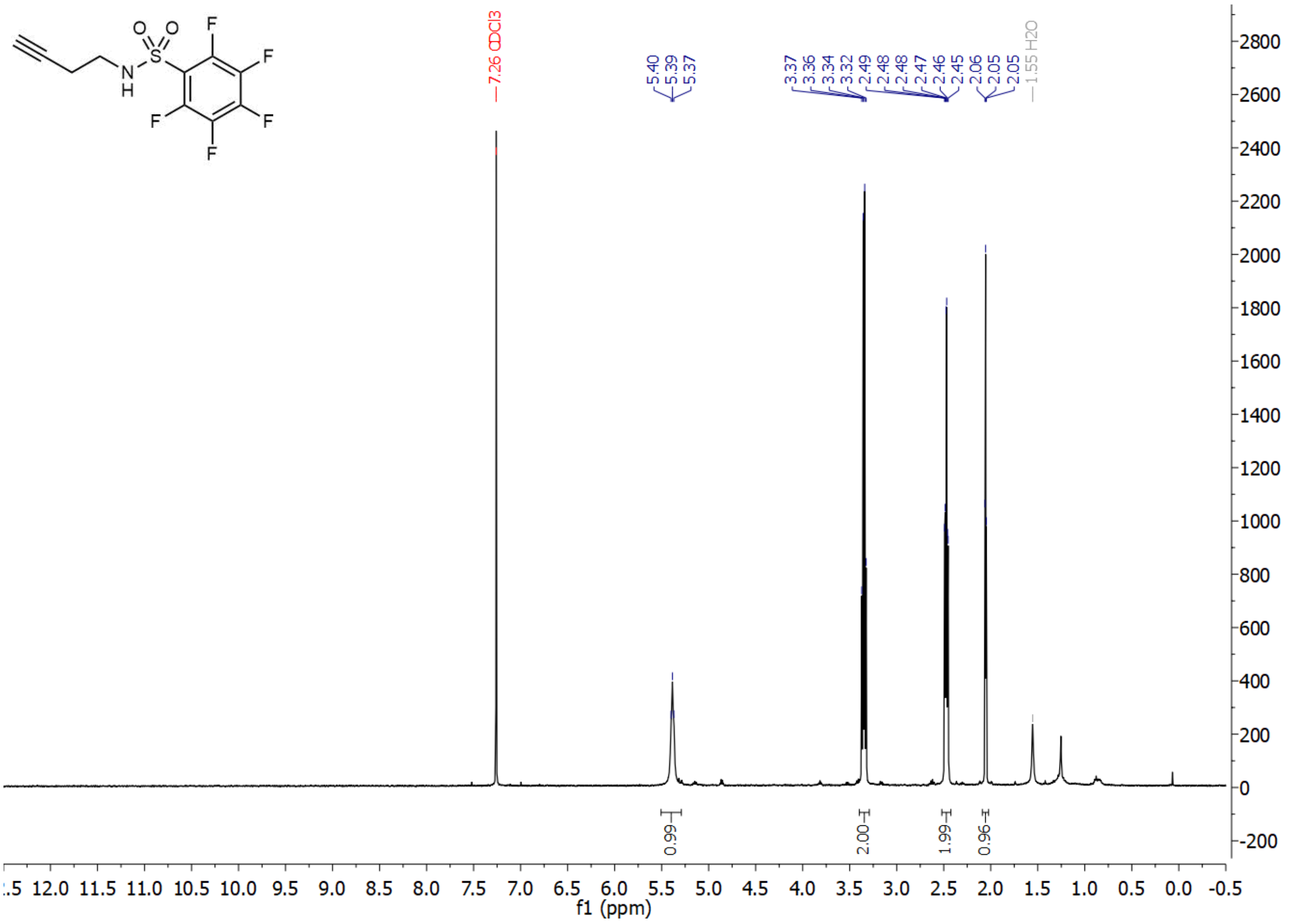

N-(But-3-yn-1-yl)-2,3,4,5,6-pentafluorobenzenesulfonamide (**PFPSA-alkyne**) (^{13}C, 101 MHz, $CDCl_3$):

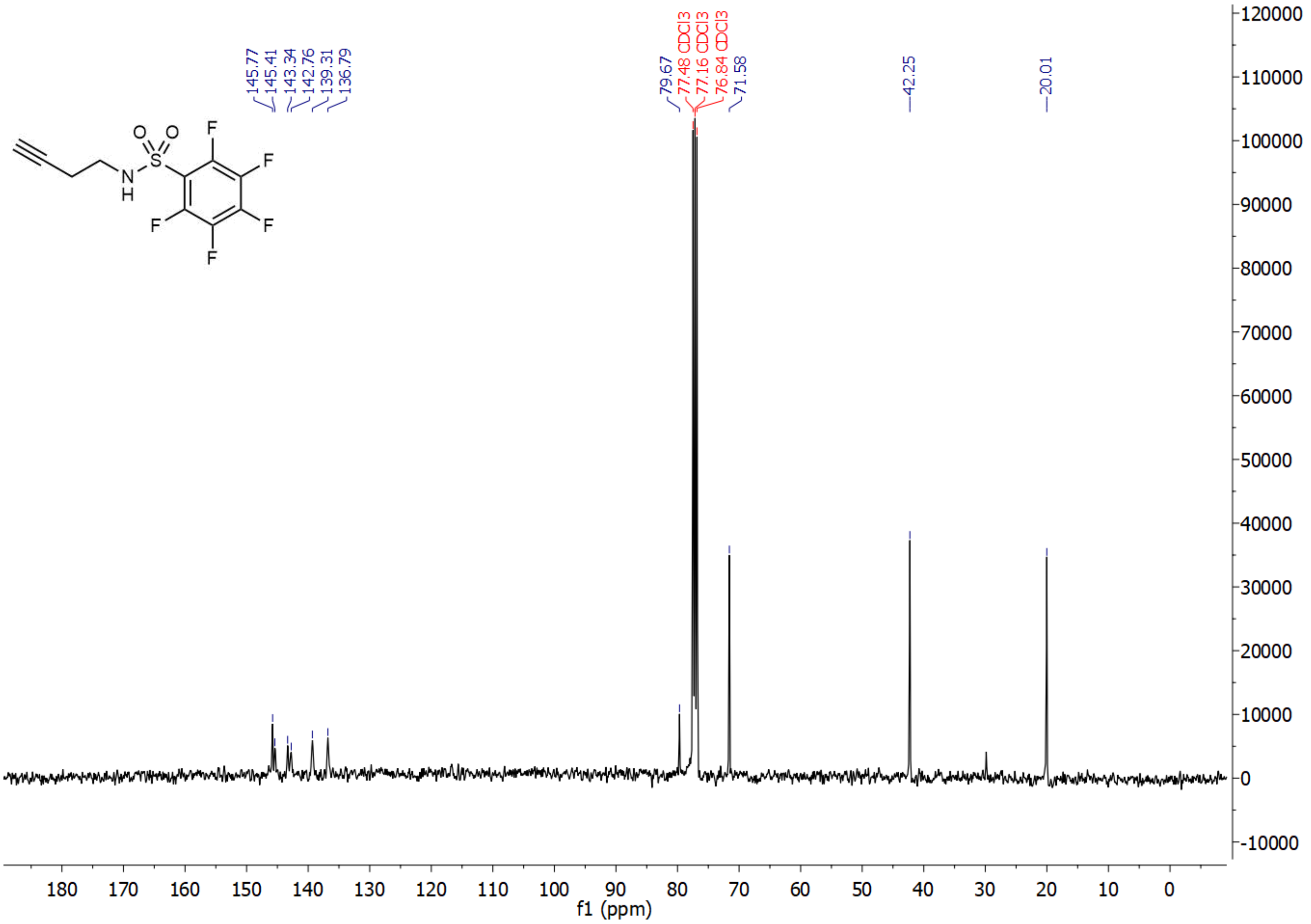

N-(But-3-yn-1-yl)-2,3,4,5,6-pentafluorobenzenesulfonamide (**PFPSA-alkyne**) (^{19}F-NMR (376 MHz, $CDCl_3$):

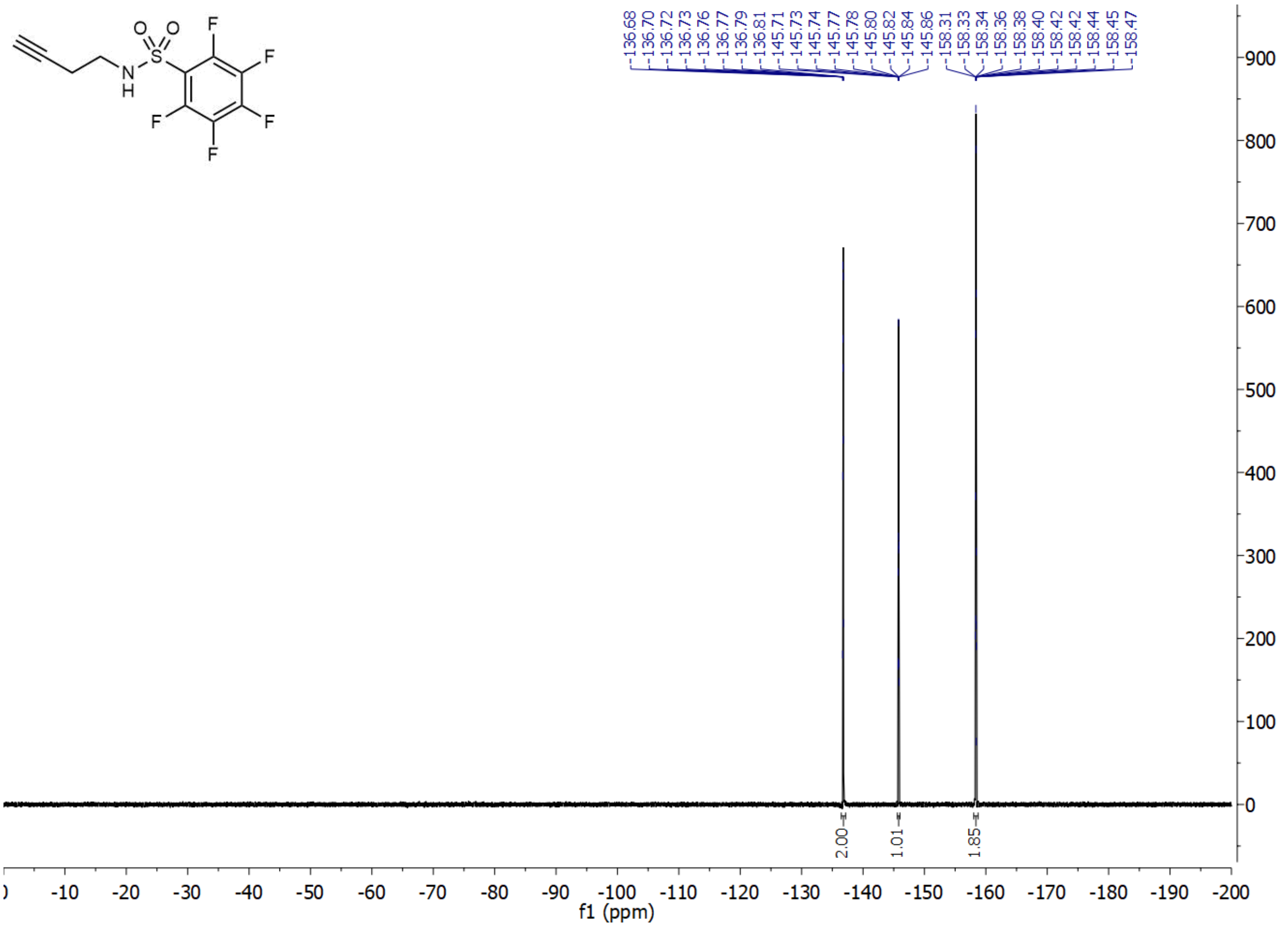

2-Bromo-*N*-(prop-2-yn-1-yl)benzo[*d*]thiazole-6-carboxamide (**BrBT-alkyne**) (^{1}H, 300 MHz, CD_3CN):

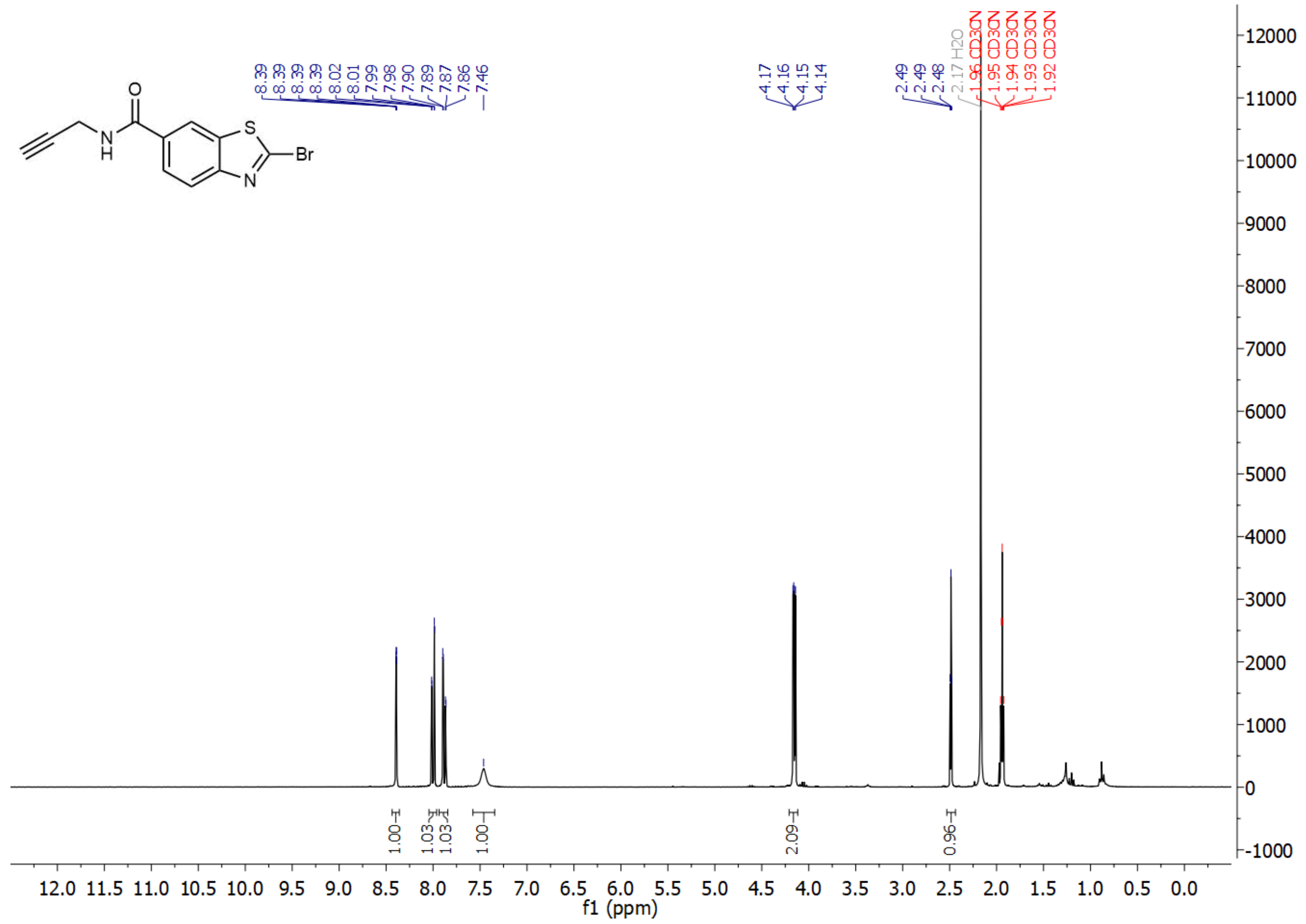

2-Bromo-*N*-(prop-2-yn-1-yl)benzo[*d*]thiazole-6-carboxamide (**BrBT-alkyne**) (^{13}C, 75.5 MHz, CD_3CN):

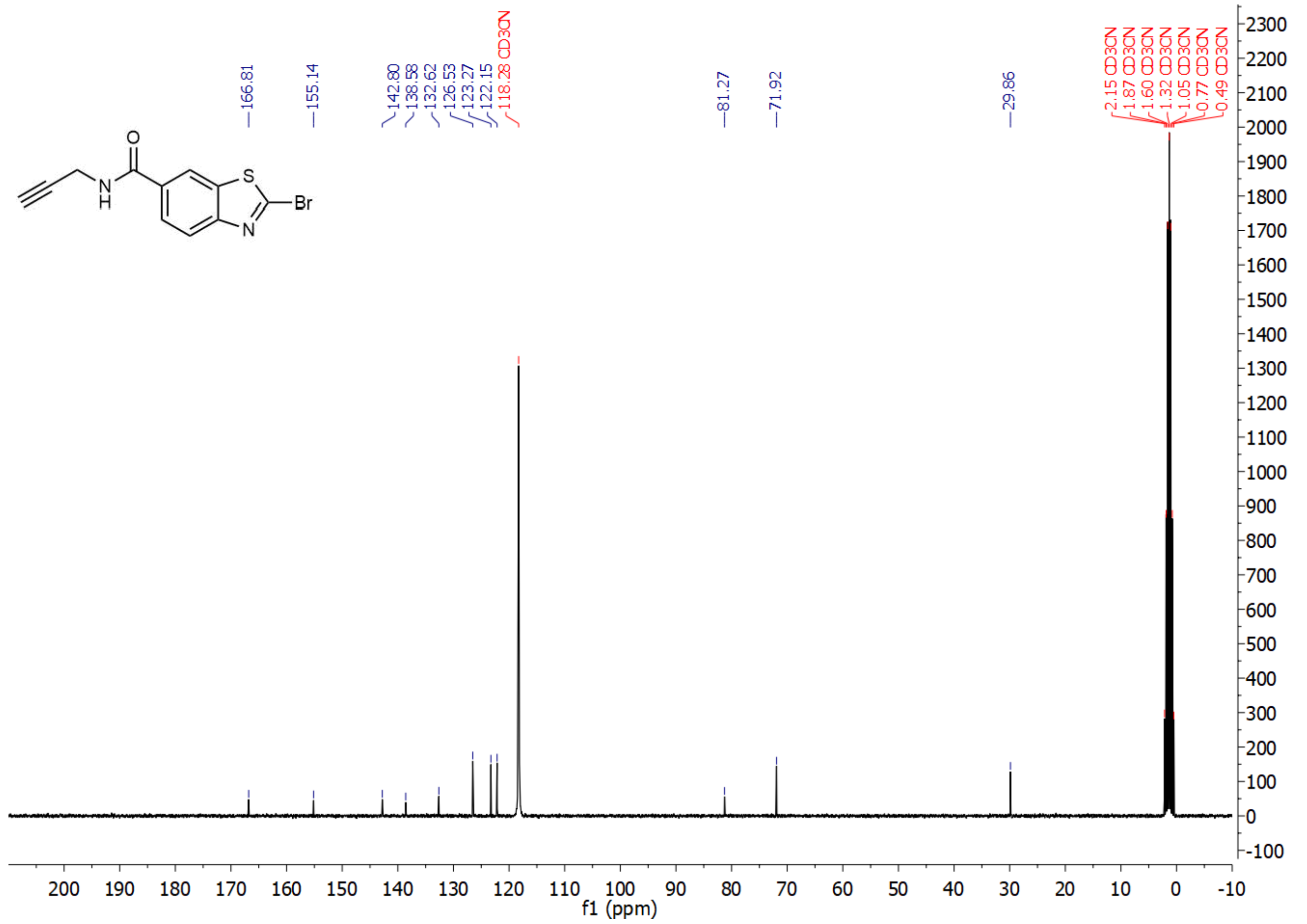

2-(Methylsulfonyl)-*N*-(prop-2-yn-1-yl)benzo[*d*]thiazole-6-carboxamide (**MSBT-alkyne**) (^{1}H, 500 MHz, CD_3CN):

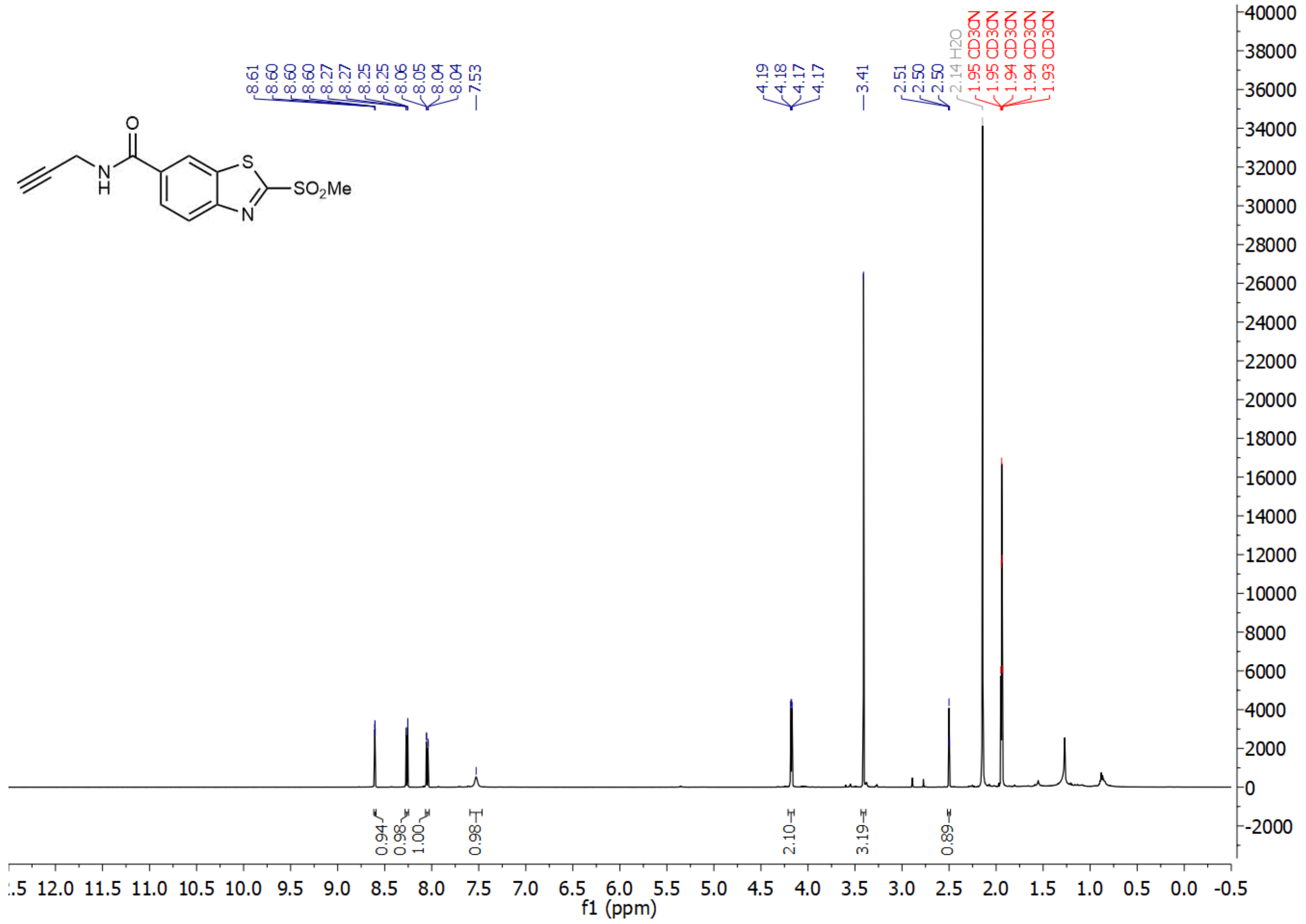

2-(Methylsulfonyl)-*N*-(prop-2-yn-1-yl)benzo[*d*]thiazole-6-carboxamide (**MSBT-alkyne**) (^{13}C, 75.5 MHz, CD_3CN):

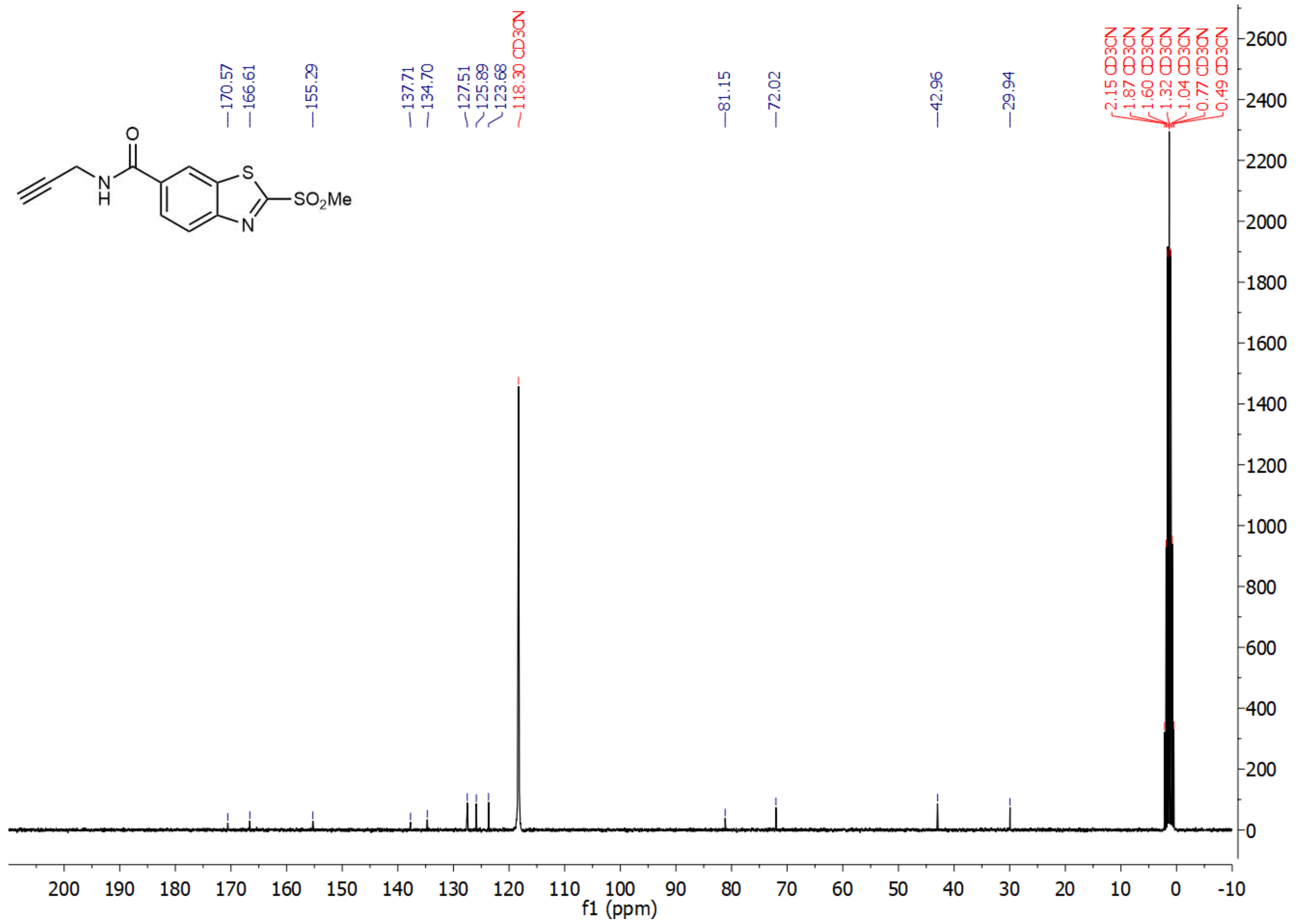

5-(Methylsulfonyl)-1-(4-(prop-2-yn-1-yloxy)phenyl)-1*H*-tetrazole (**MST-alkyne**) (^{1}H, 400 MHz, $CDCl_3$):

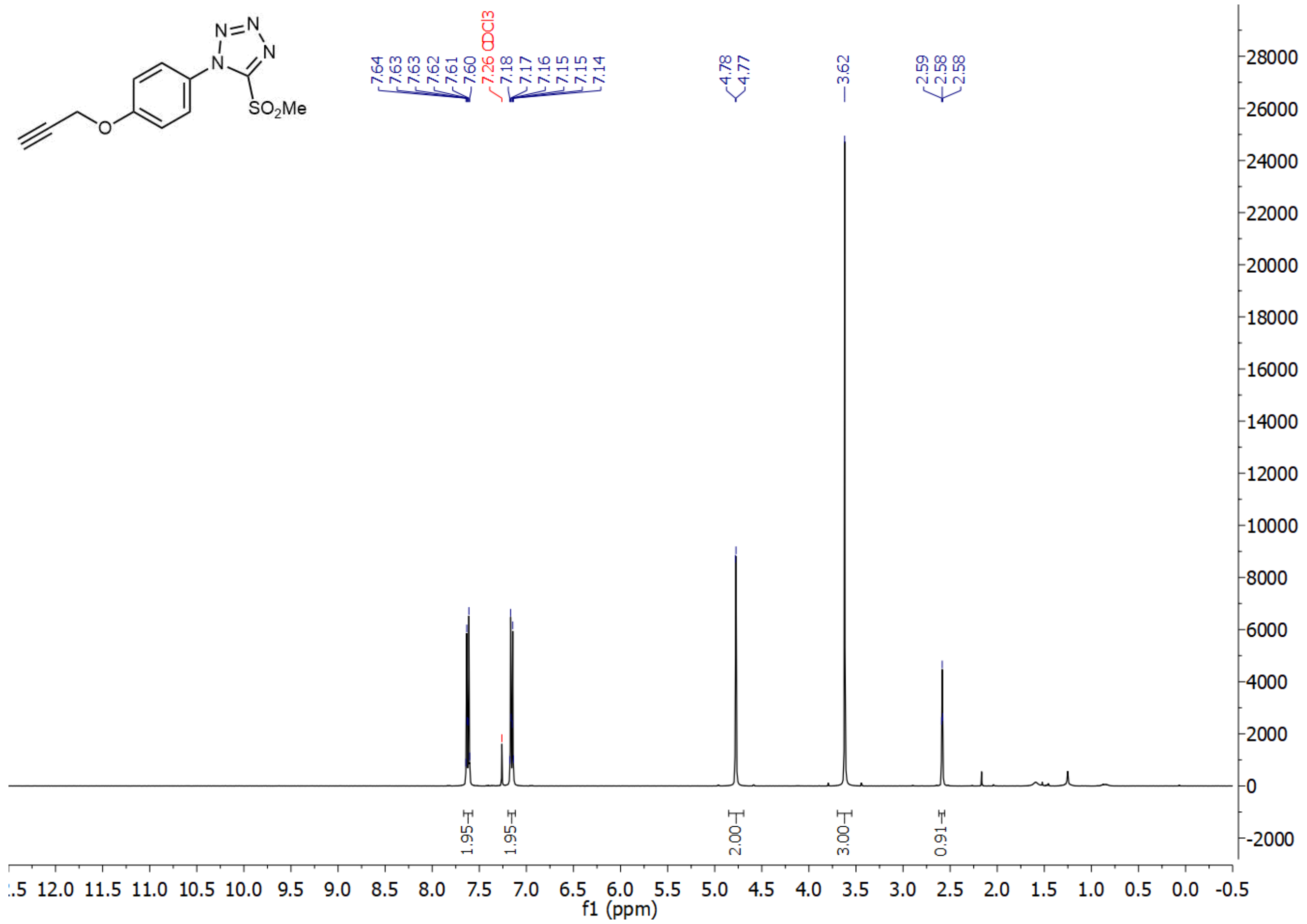

5-(Methylsulfonyl)-1-(4-(prop-2-yn-1-yloxy)phenyl)-1*H*-tetrazole (**MST-alkyne**) (^{13}C, 101 MHz, $CDCl_3$):

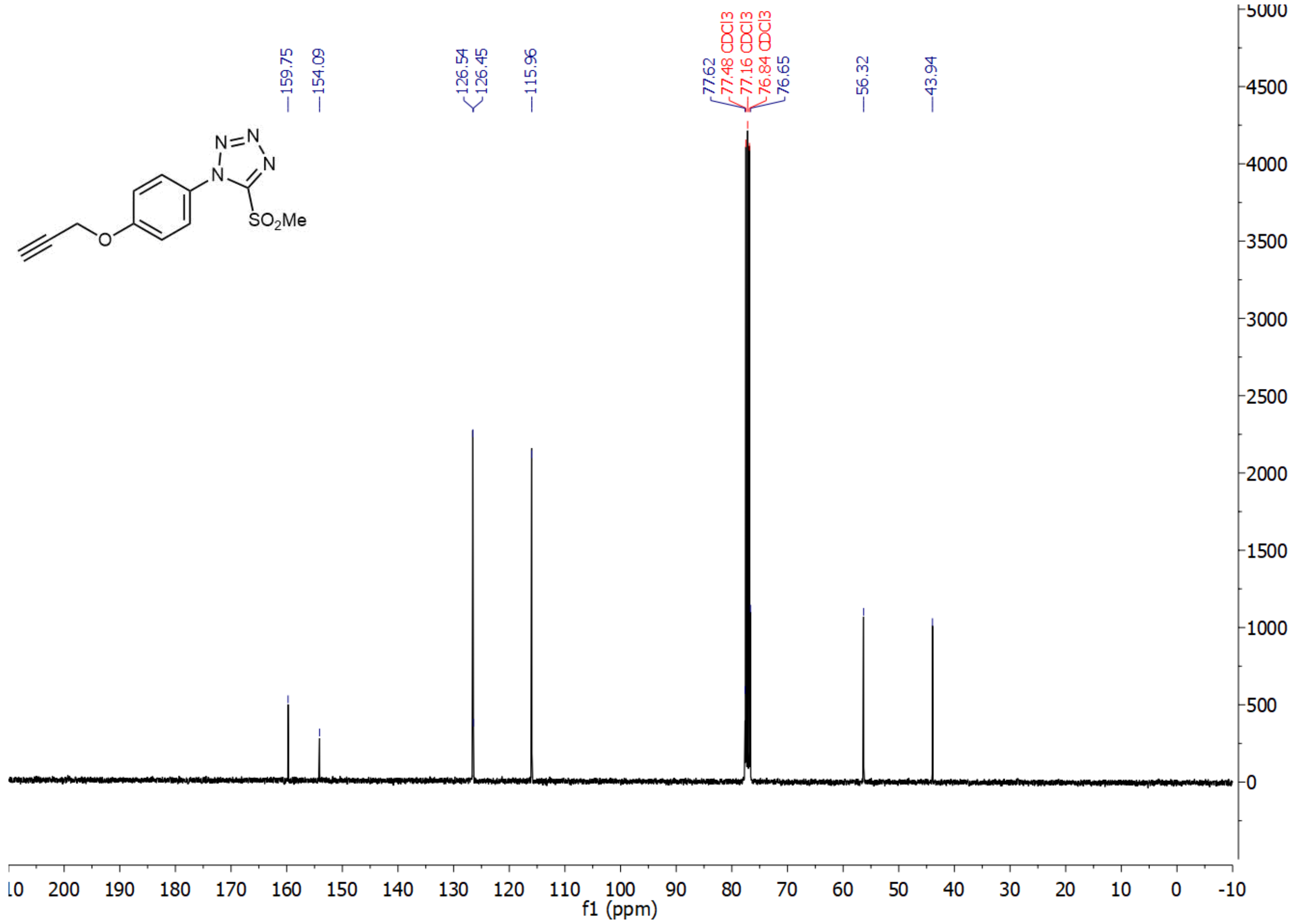

2-(Methylsulfonyl)-5-(4-(prop-2-yn-1-yloxy)phenyl)-1,3,4-oxadiazole (**MSOD-alkyne**) (^{1}H, 300 MHz, $CDCl_3$):

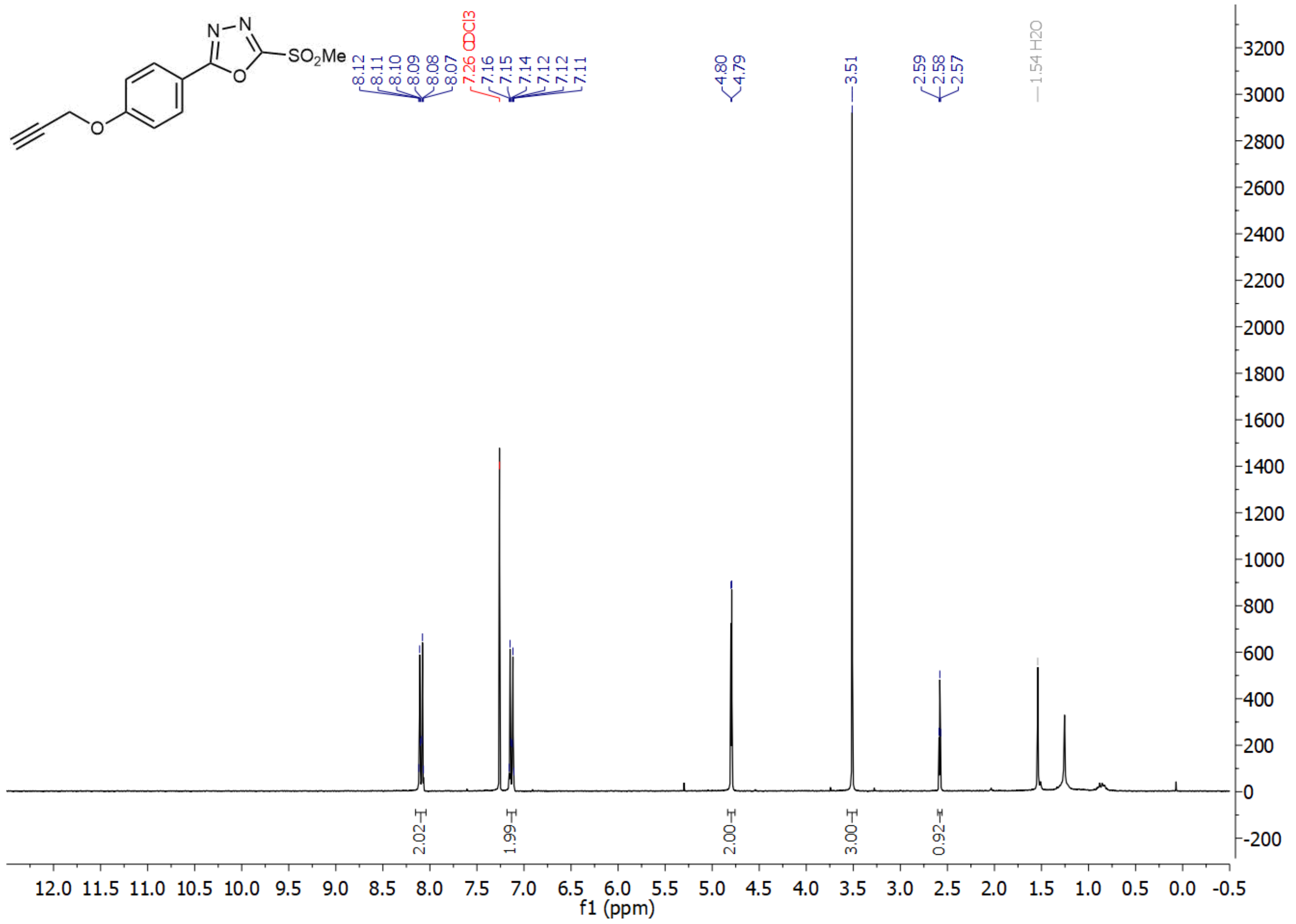

2-(Methylsulfonyl)-5-(4-(prop-2-yn-1-yloxy)phenyl)-1,3,4-oxadiazole (**MSOD-alkyne**) (^{13}C, 101 MHz, $CDCl_3$):

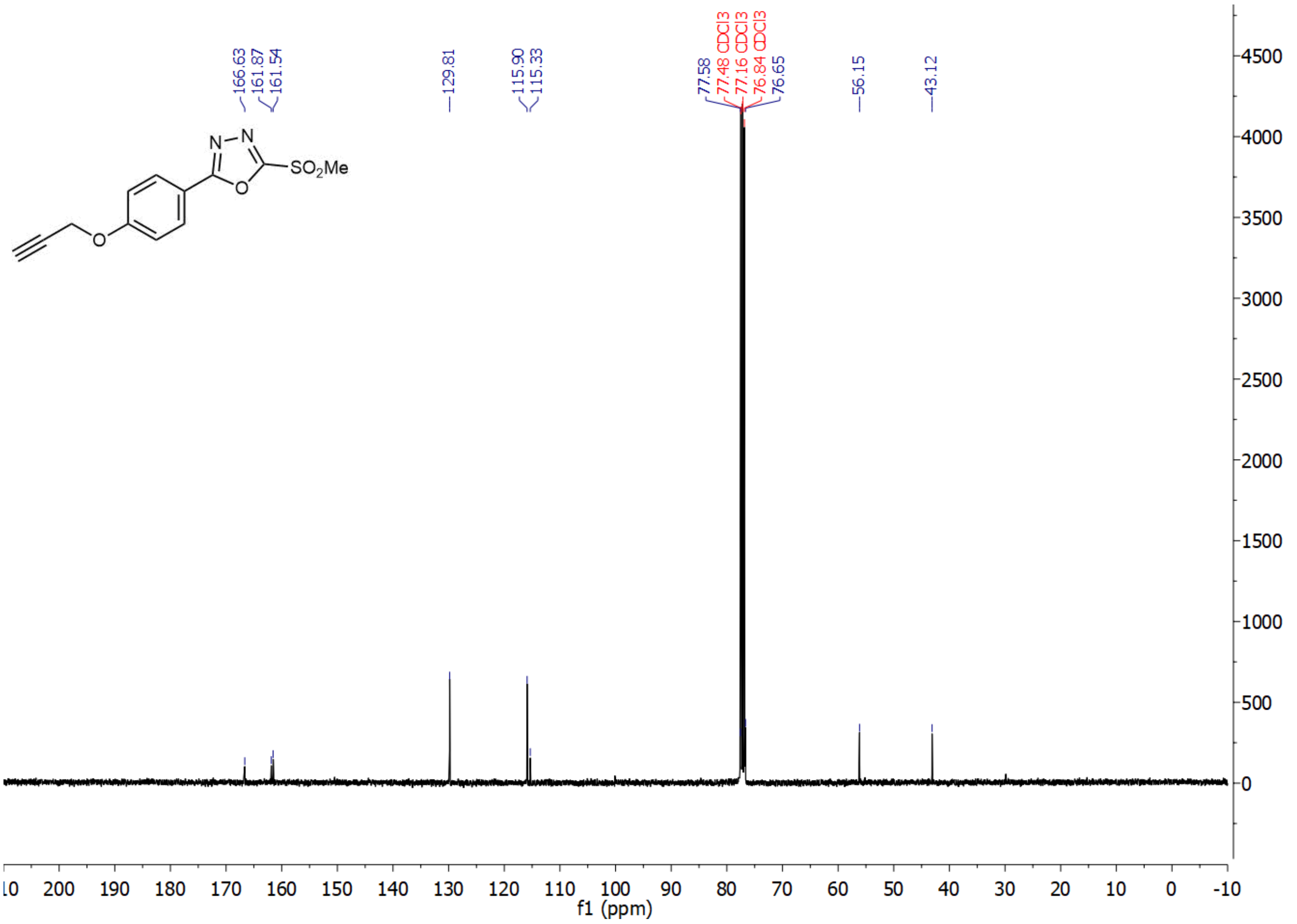

5-Methyl-1-[(trimethylsilyl)ethynyl]-1,2-benziodoxol-3(1*H*)-one (**EBX2-alkyne**) (^{1}H, 300 MHz, $CDCl_3$):

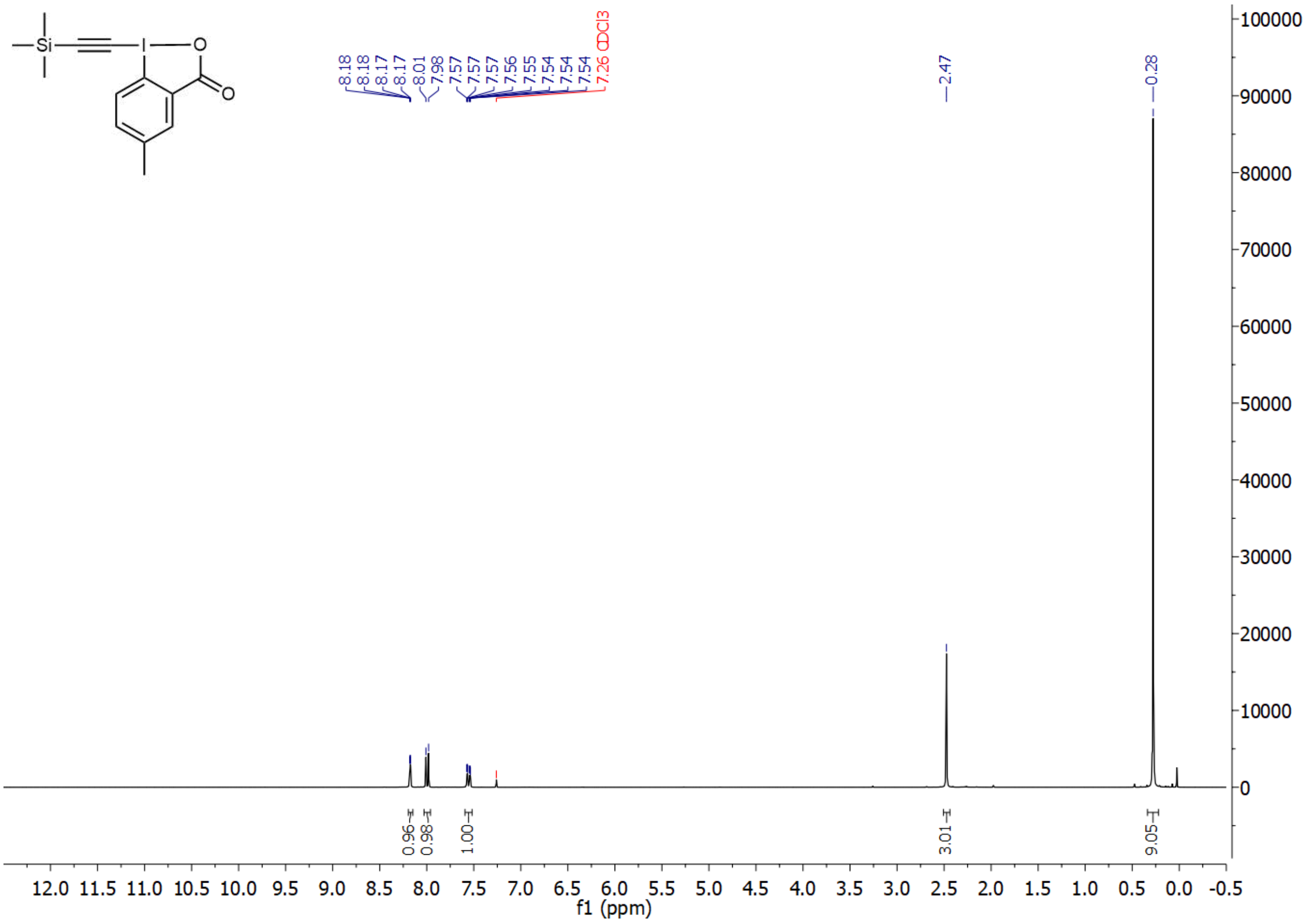

5-Methyl-1-[(trimethylsilyl)ethynyl]-1,2-benziodoxol-3(1*H*)-one (**EBX2-alkyne**) (^{13}C, 75.5 MHz, $CDCl_3$):

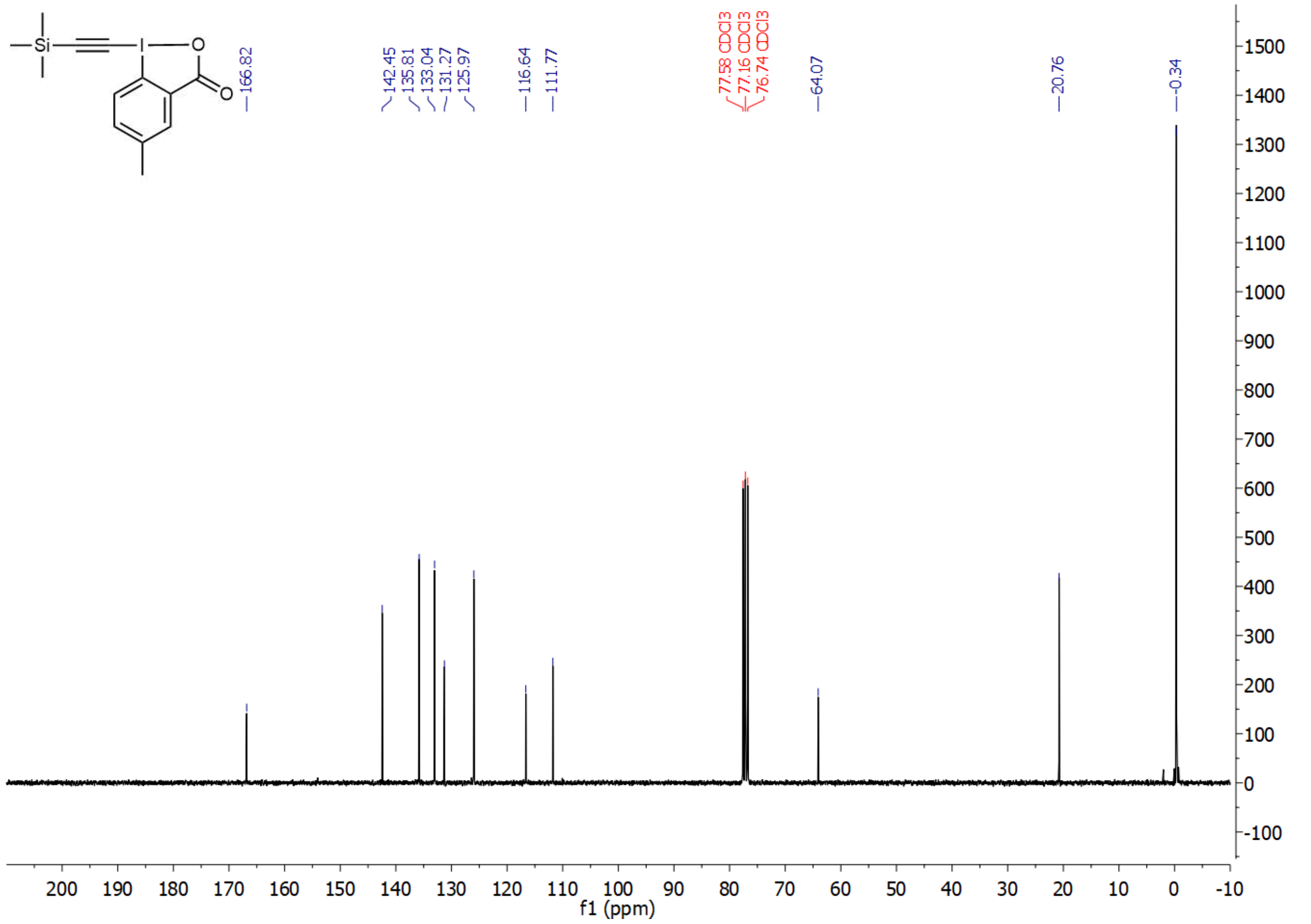

2-(But-3-yn-1-yl)oxirane (**Ep-alkyne**) (^{1}H, 300 MHz, CD_2Cl_2):

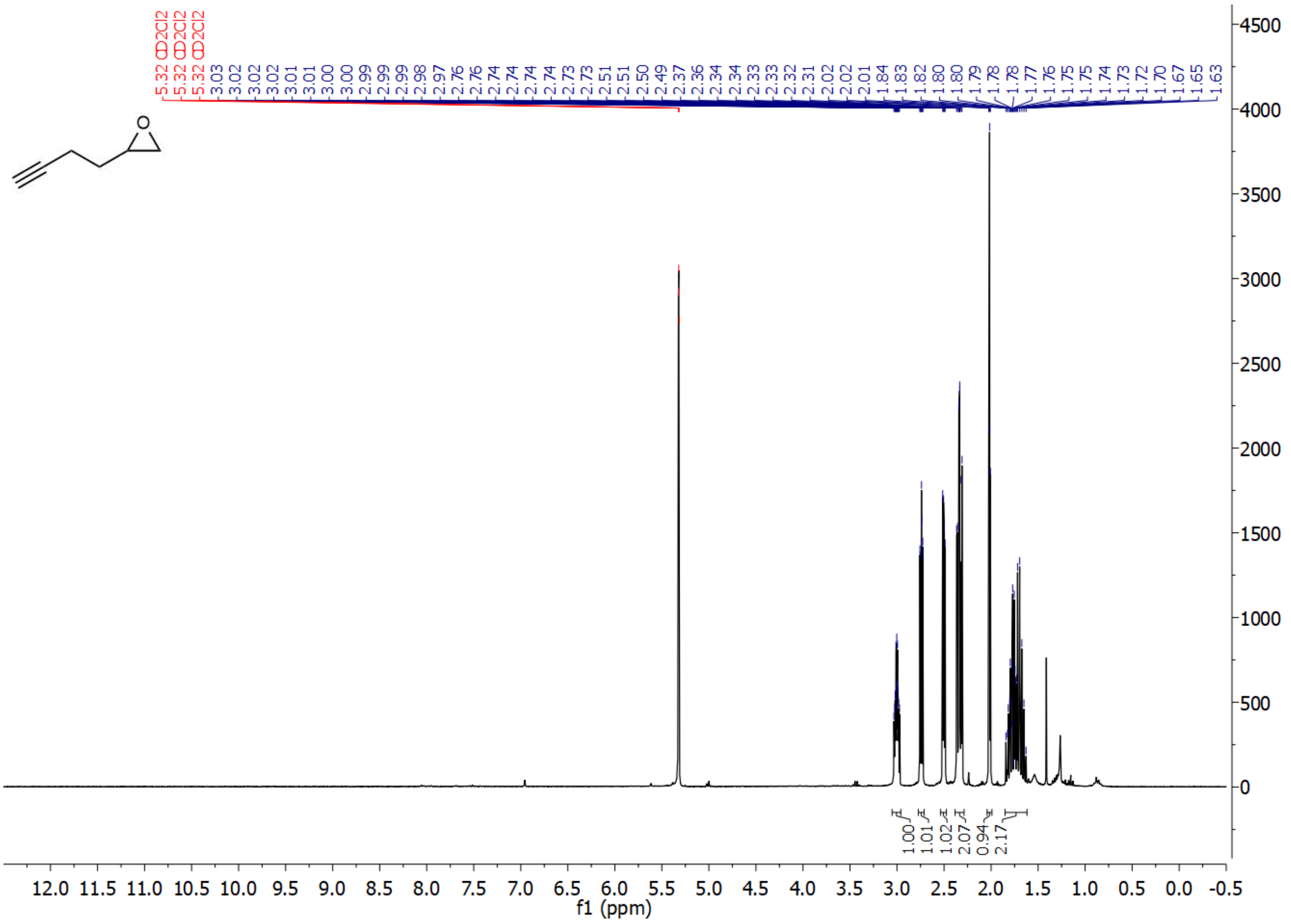

2-(But-3-yn-1-yl)oxirane (**Ep-alkyne**) (^{13}C, 101 MHz, CD_2Cl_2):

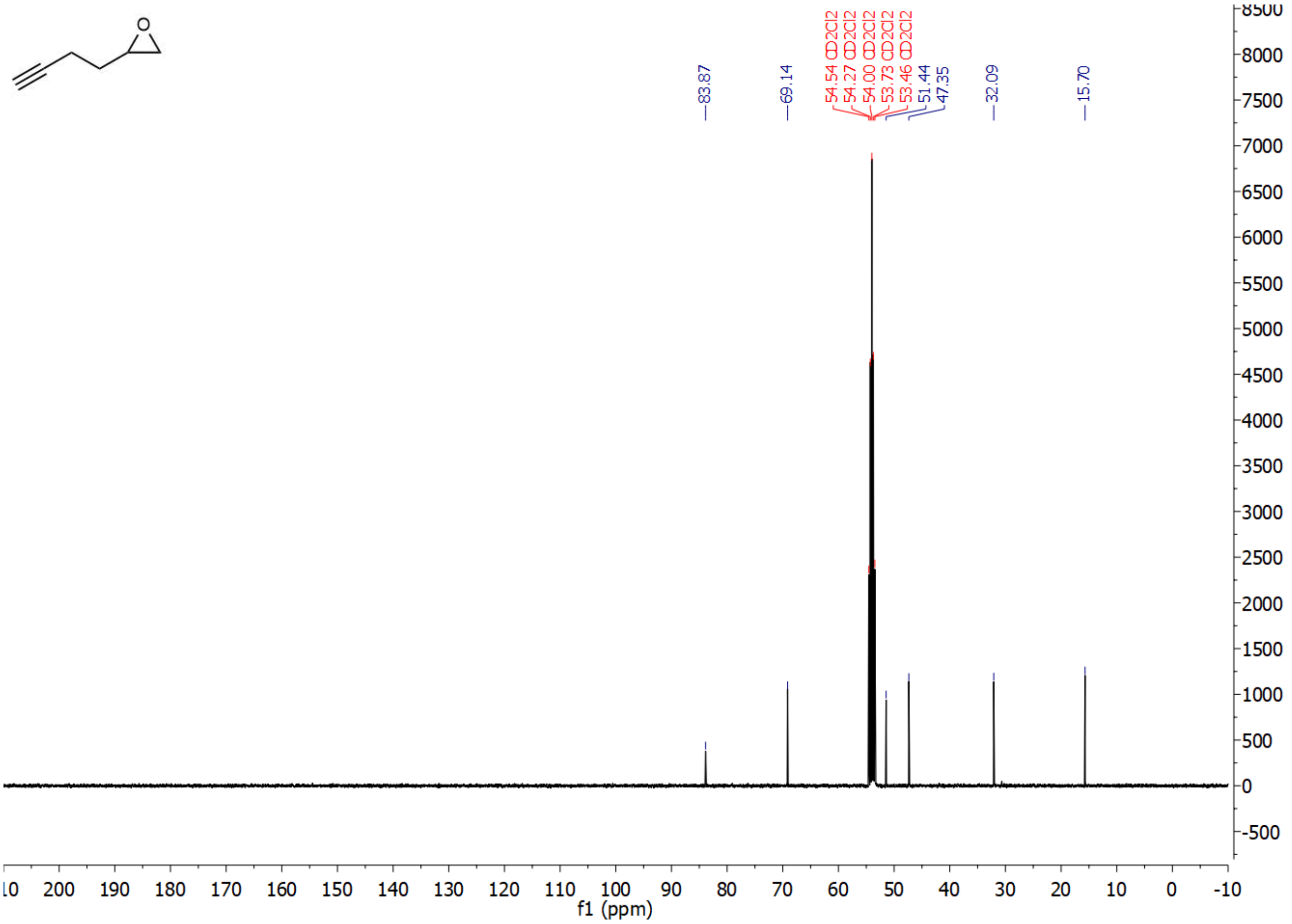

N-(Hex-5-yn-1-yl)propiolamide (**AlkPA-alkyne**) (^{1}H, 300 MHz, $CDCl_3$):

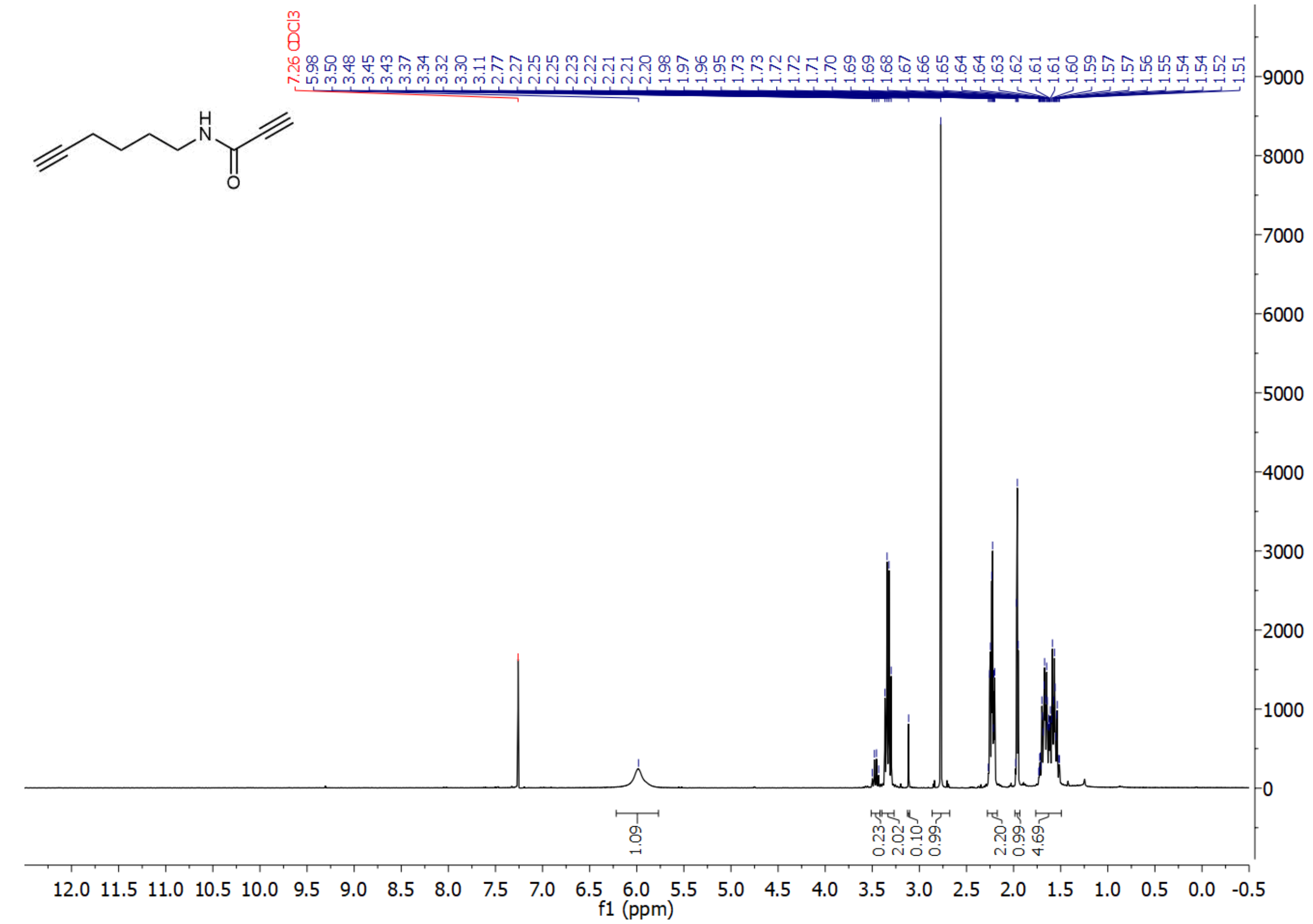

N-(Hex-5-yn-1-yl)propiolamide (**AlkPA-alkyne**) (^{13}C, 75.5 MHz, CD_3CN):

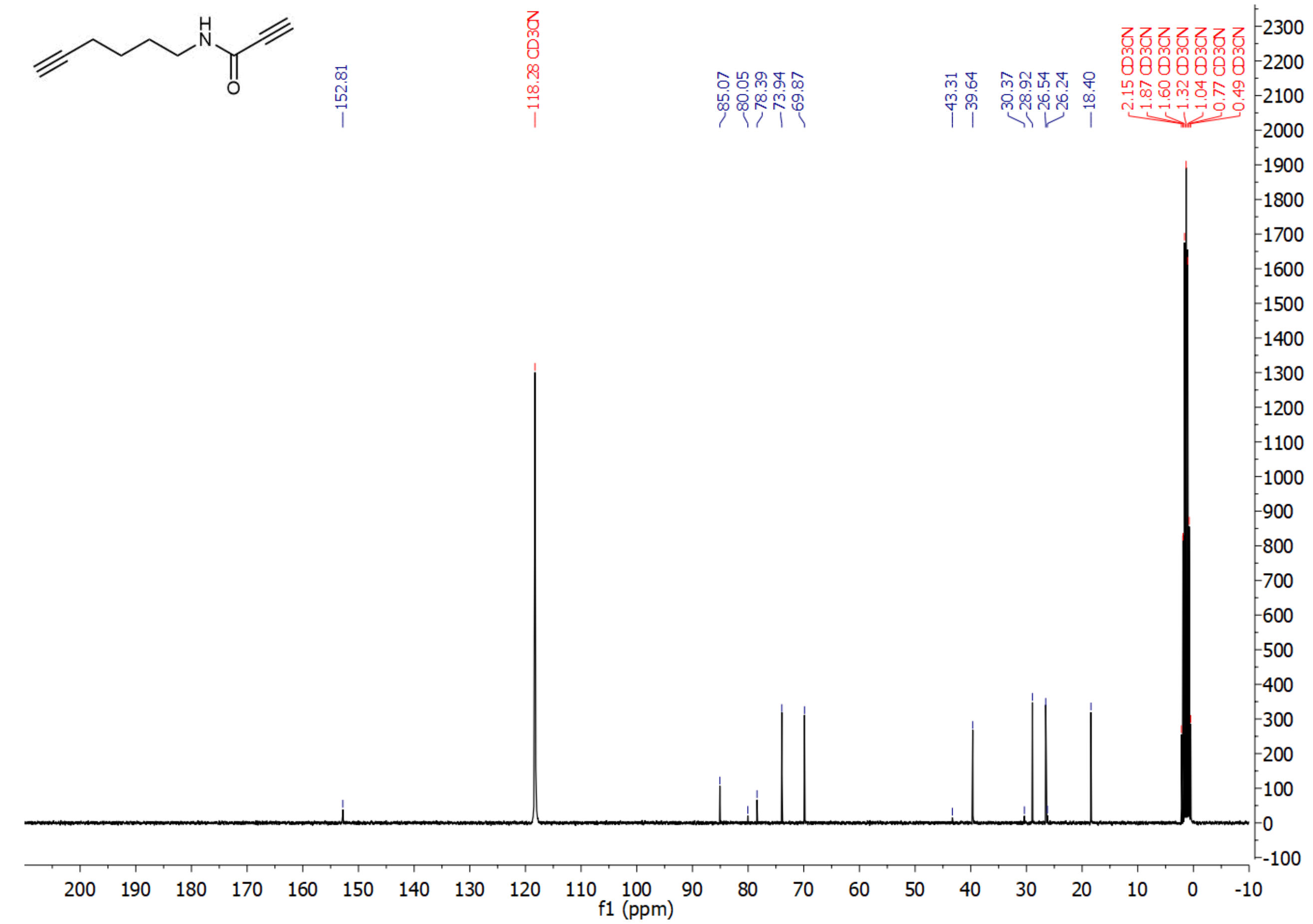

N-(4-Ethynylphenyl)propiolamide (**ArPA-alkyne**) (^{1}H, 400 MHz, CD_3CN):

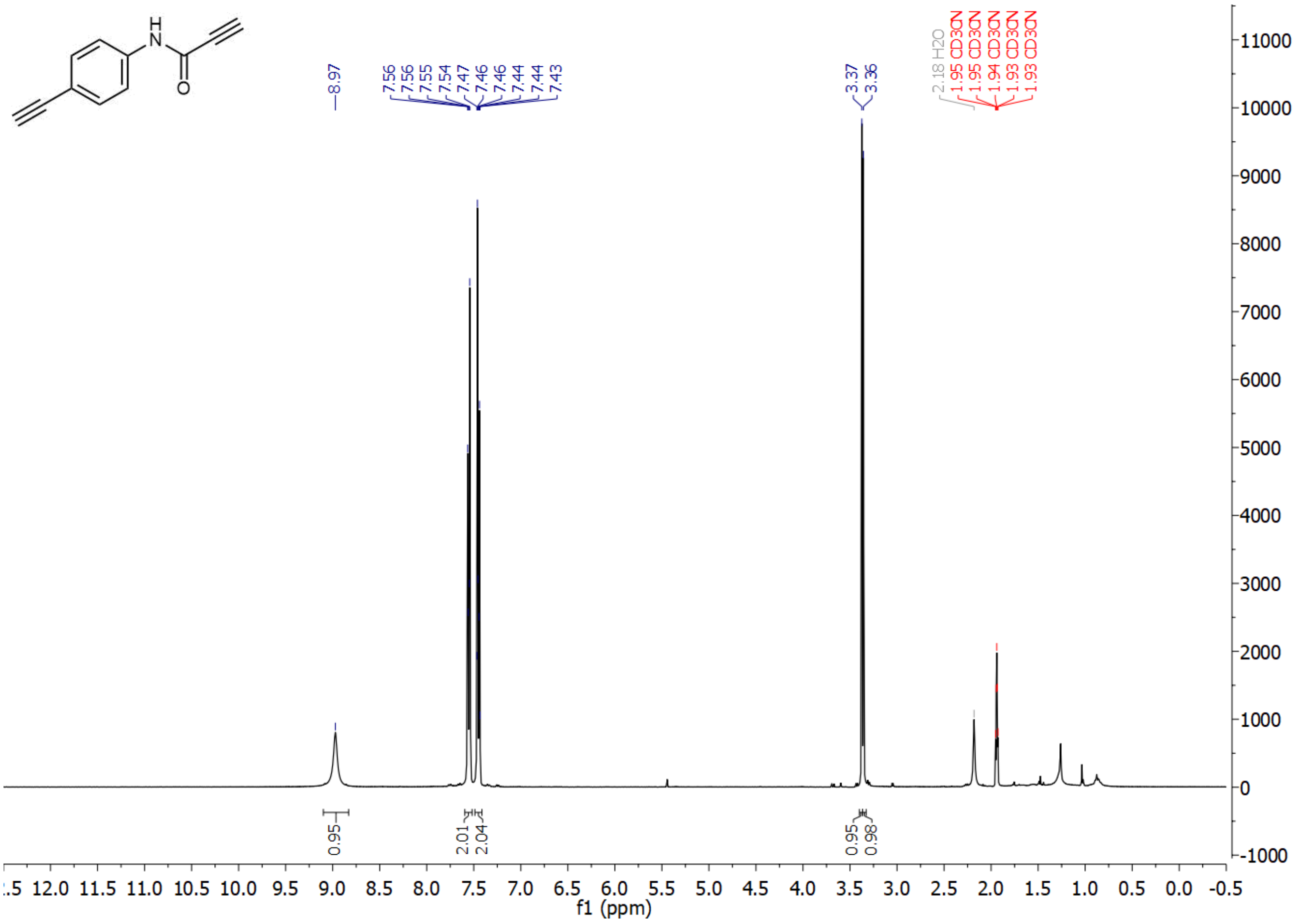

N-(4-Ethynylphenyl)propiolamide (**ArPA-alkyne**) (^{13}C, 101 MHz, CD_3CN):

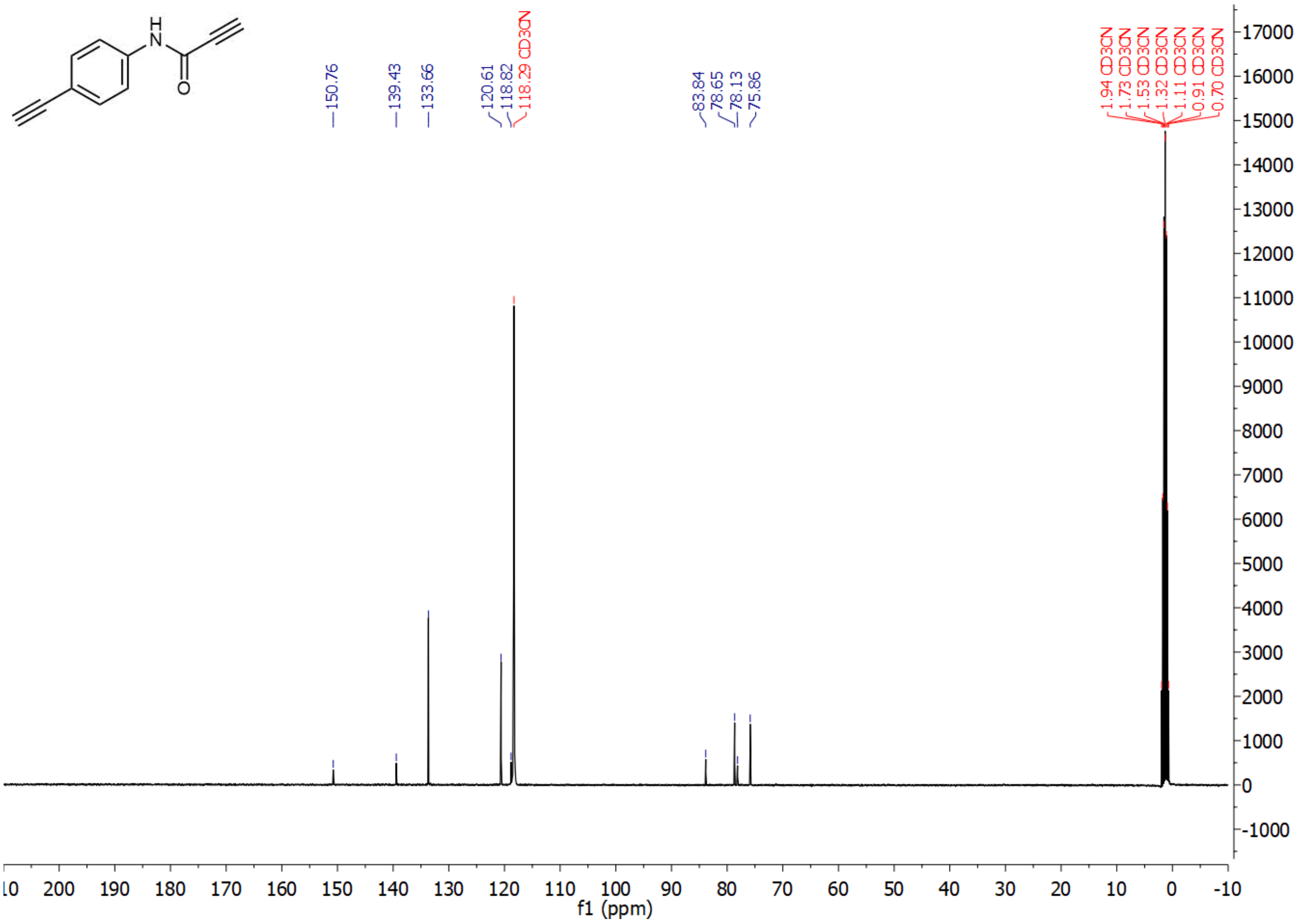

N-(Hex-5-yn-1-yl)acrylamide (**AlkAA-alkyne**) (^{1}H, 300 MHz, $CDCl_3$):

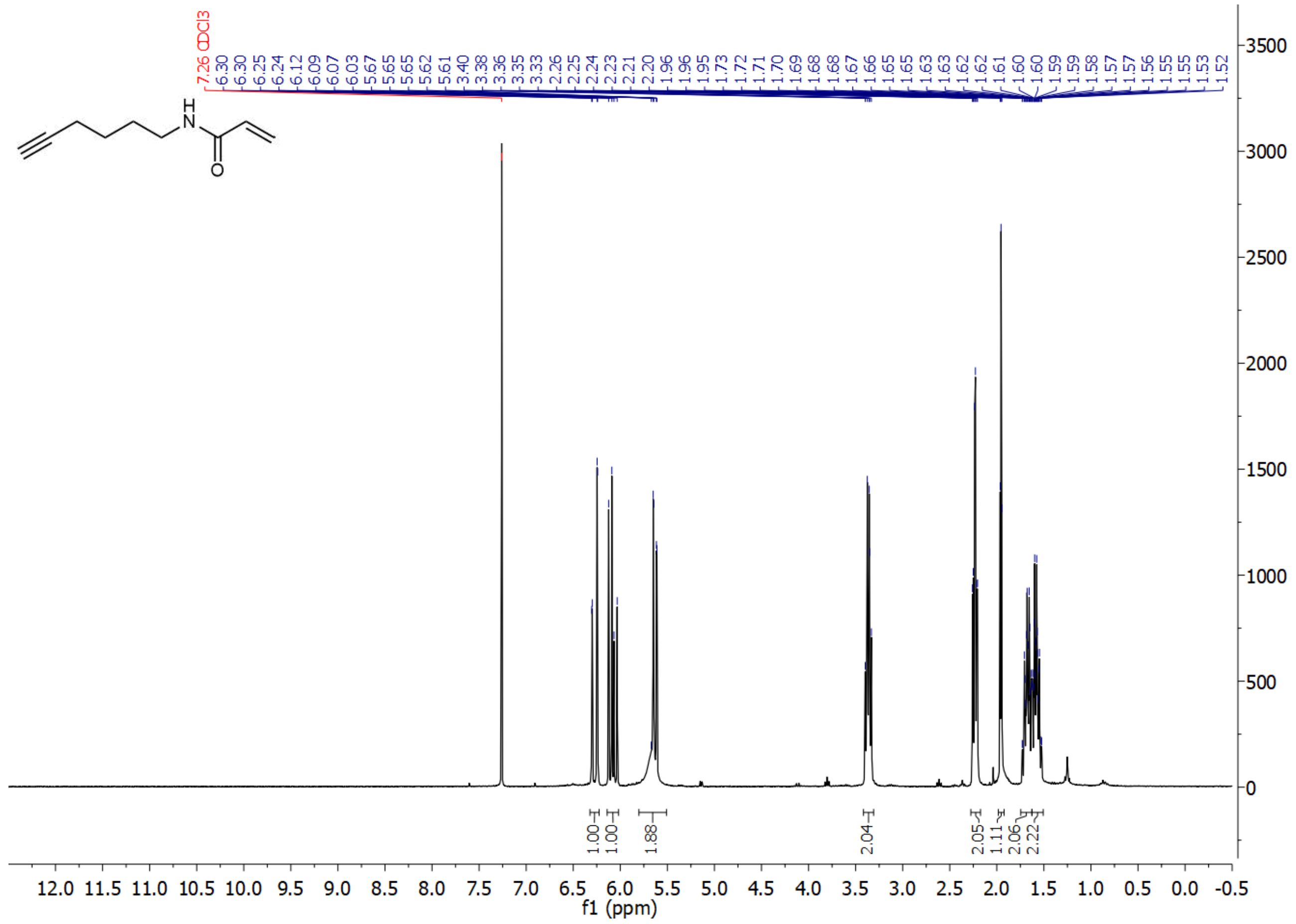

N-(Hex-5-yn-1-yl)acrylamide (**AlkAA-alkyne**) (^{13}C, 75.5 MHz, $CDCl_3$):

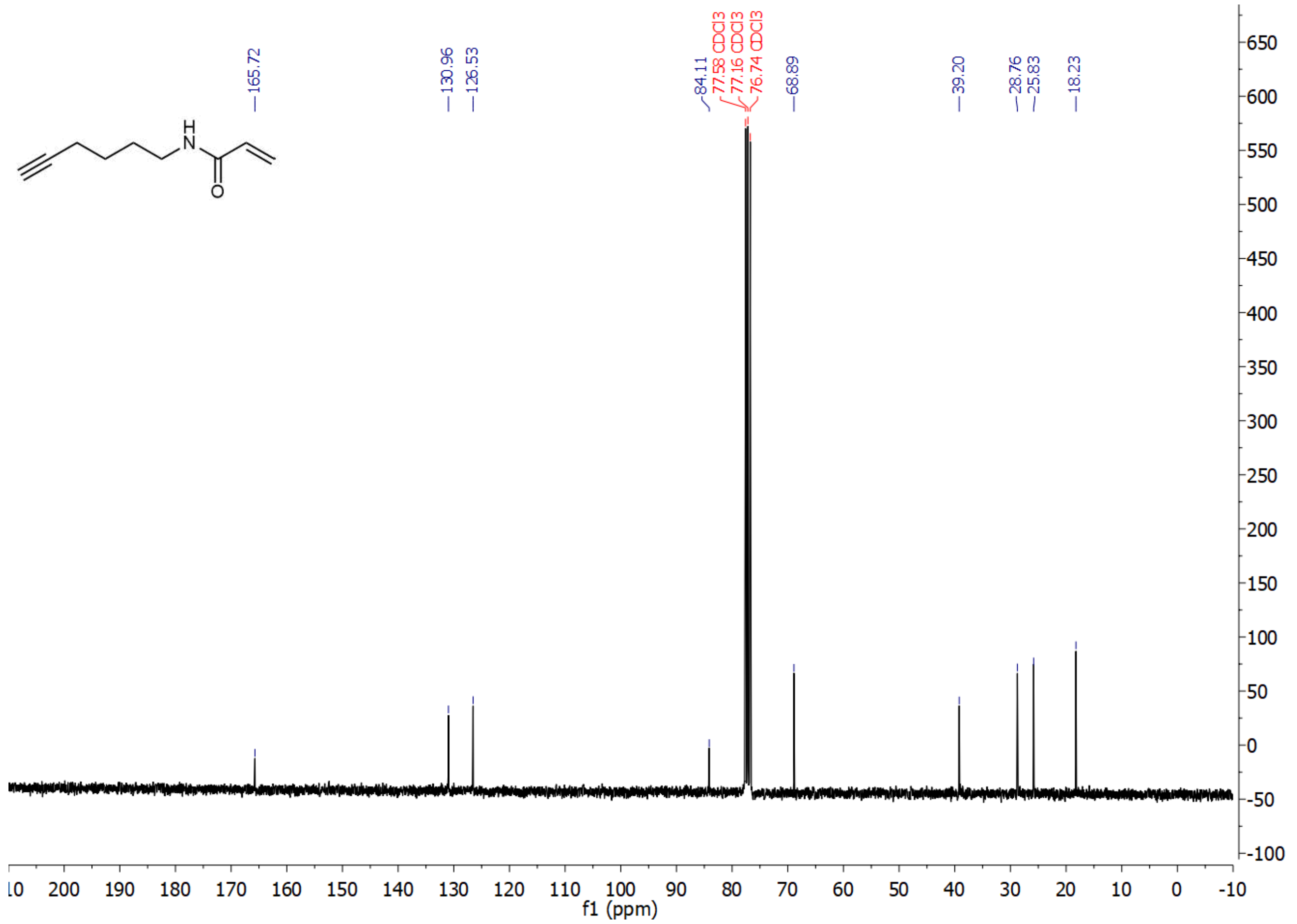

N-(4-Ethynylphenyl)acrylamide (**ArAA-alkyne**) (^{1}H, 400 MHz, CD_3CN):

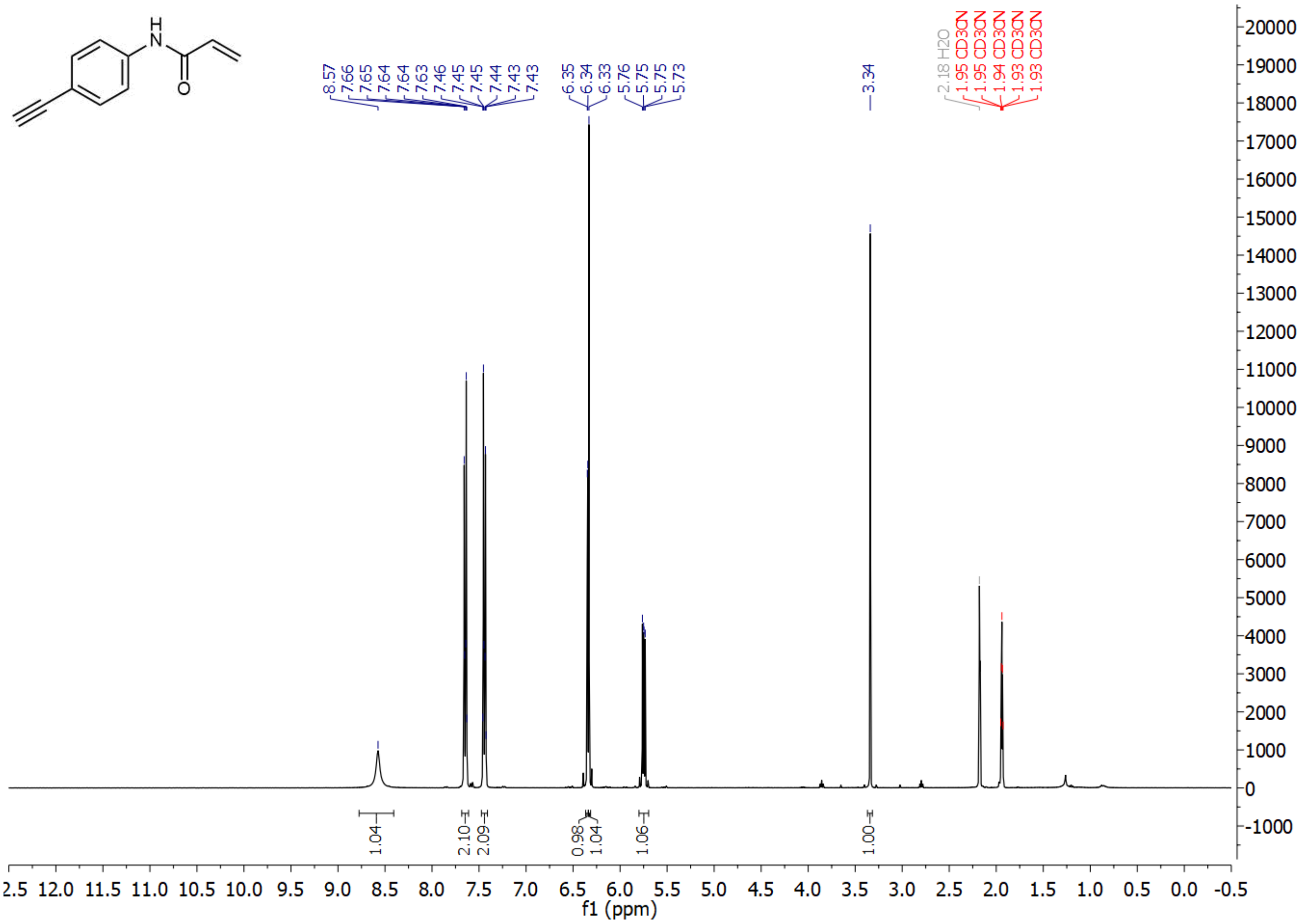

N-(4-Ethynylphenyl)acrylamide (**ArAA-alkyne**) (^{13}C, 101 MHz, CD_3CN):

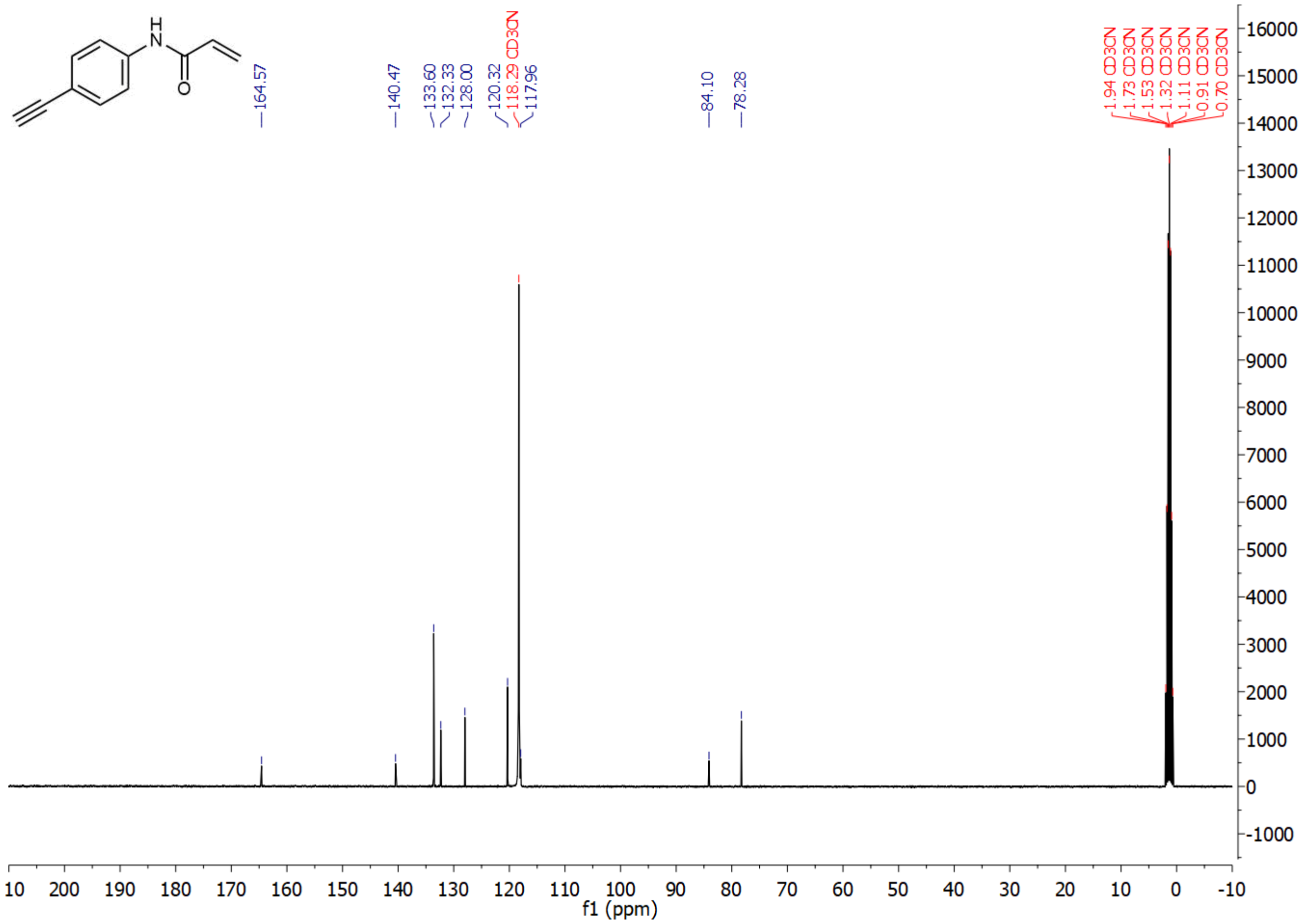

2-Fluoro-*N*-(hex-5-yn-1-yl)acrylamide **(AlkFAA-alkyne)** (^{1}H, 400 MHz, $CDCl_3$):

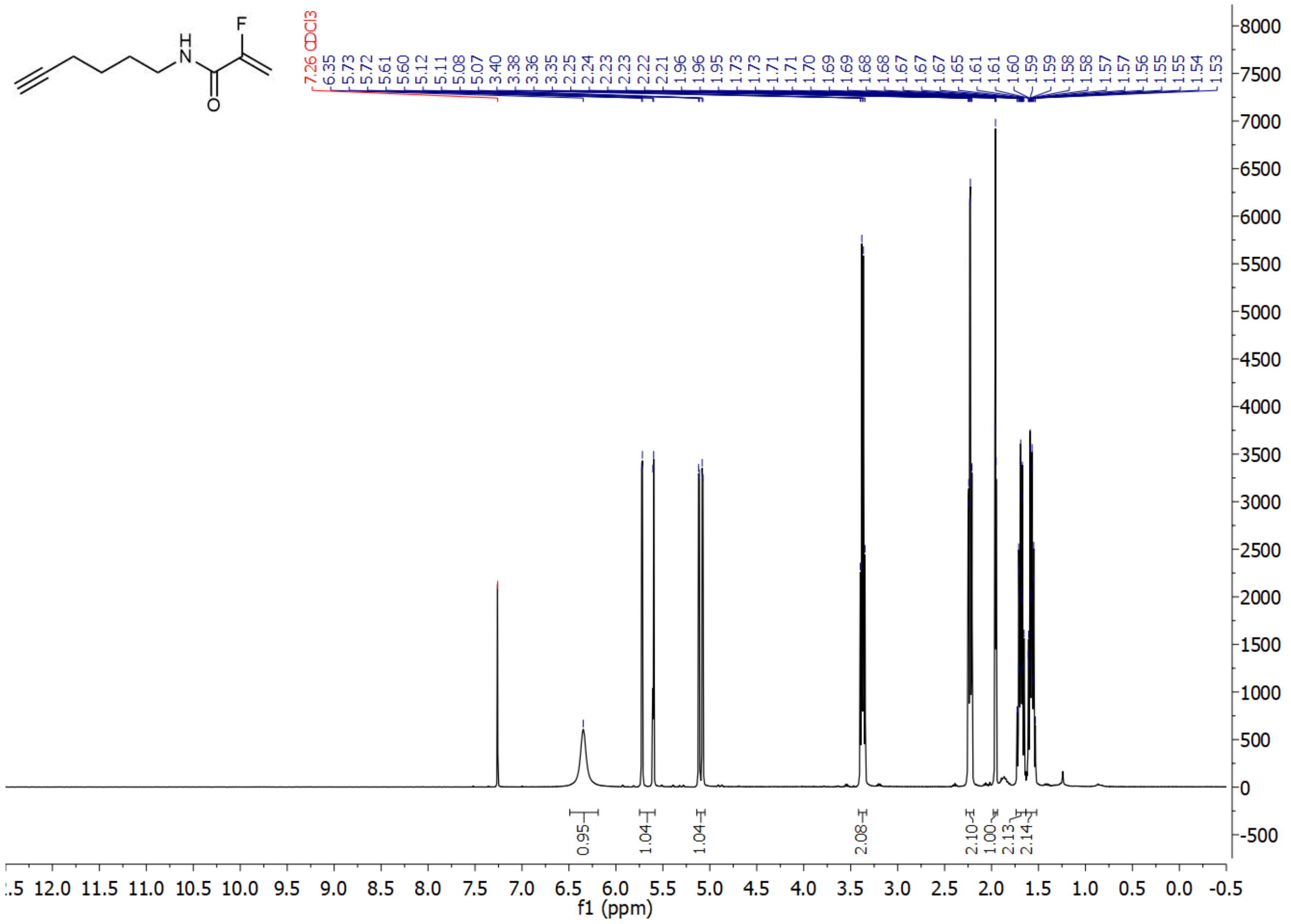

2-Fluoro-*N*-(hex-5-yn-1-yl)acrylamide **(AlkFAA-alkyne)** (^{13}C, 101 MHz, $CDCl_3$):

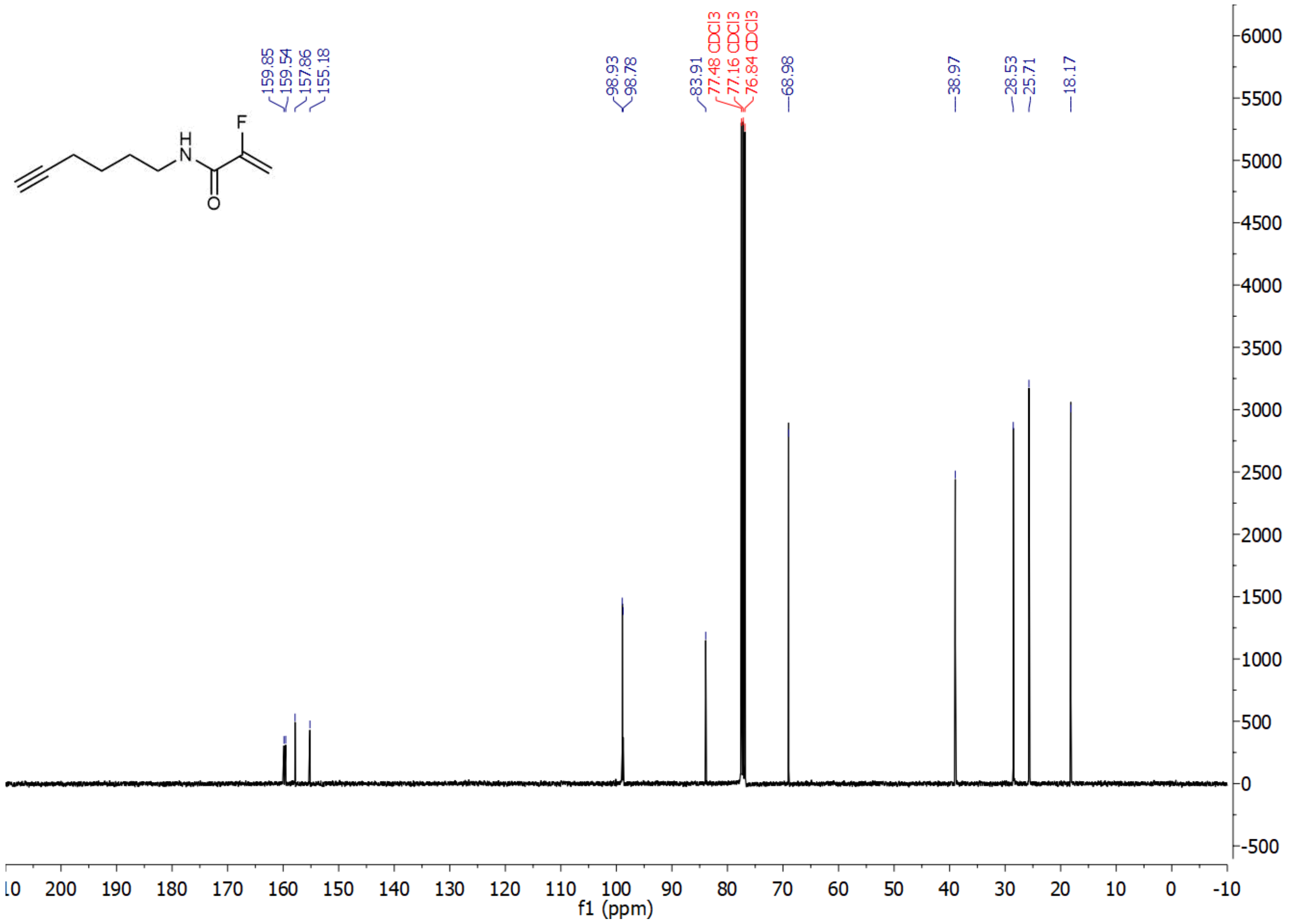

2-Fluoro-*N*-(hex-5-yn-1-yl)acrylamide **(AlkFAA-alkyne)** (^{19}F, 376 MHz, $CDCl_3$):

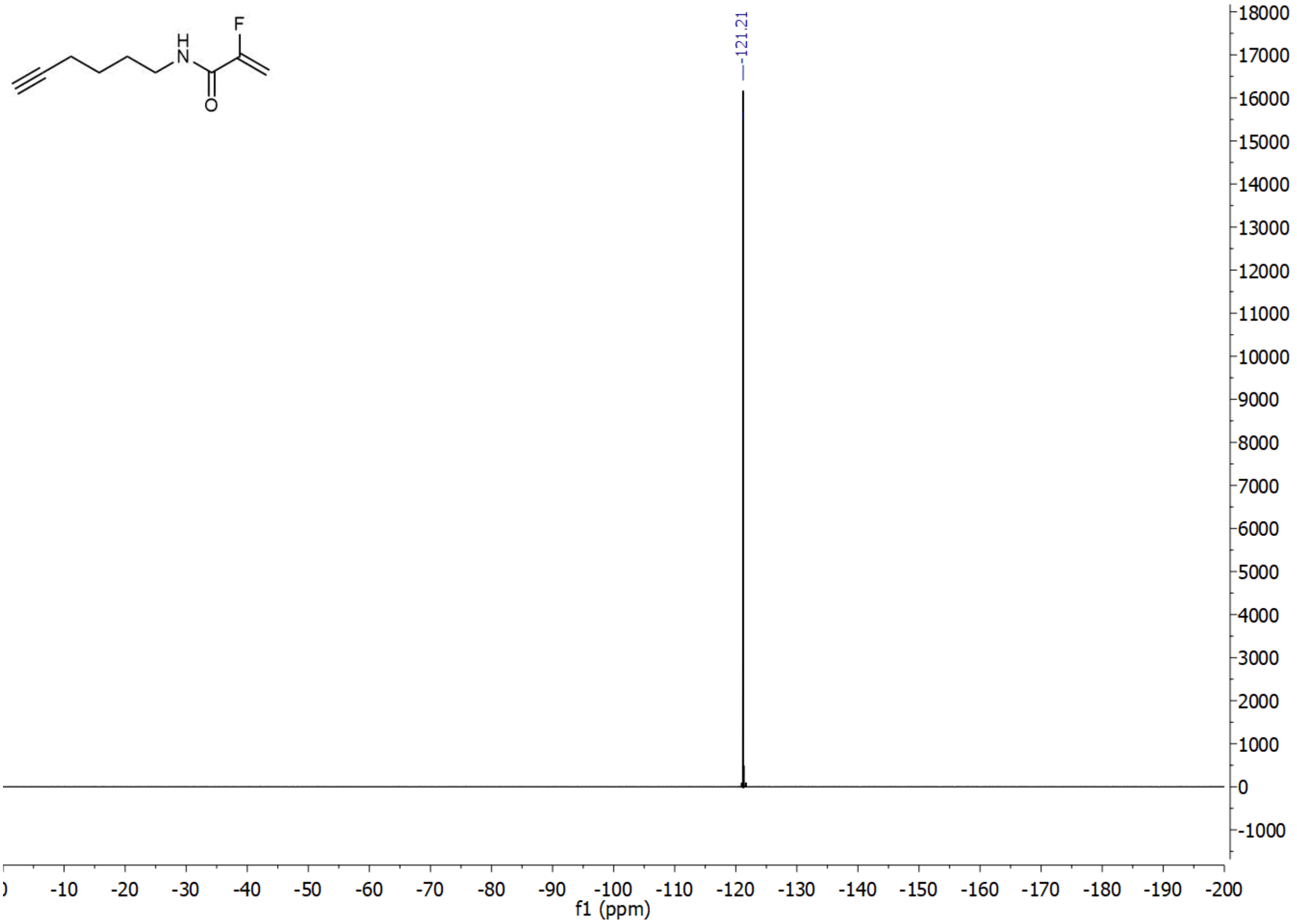

5-(2-(Vinylsulfonyl)ethoxy)pent-1-yne (**AlkVS-alkyne**) (^{1}H, 300 MHz, $CDCl_3$):

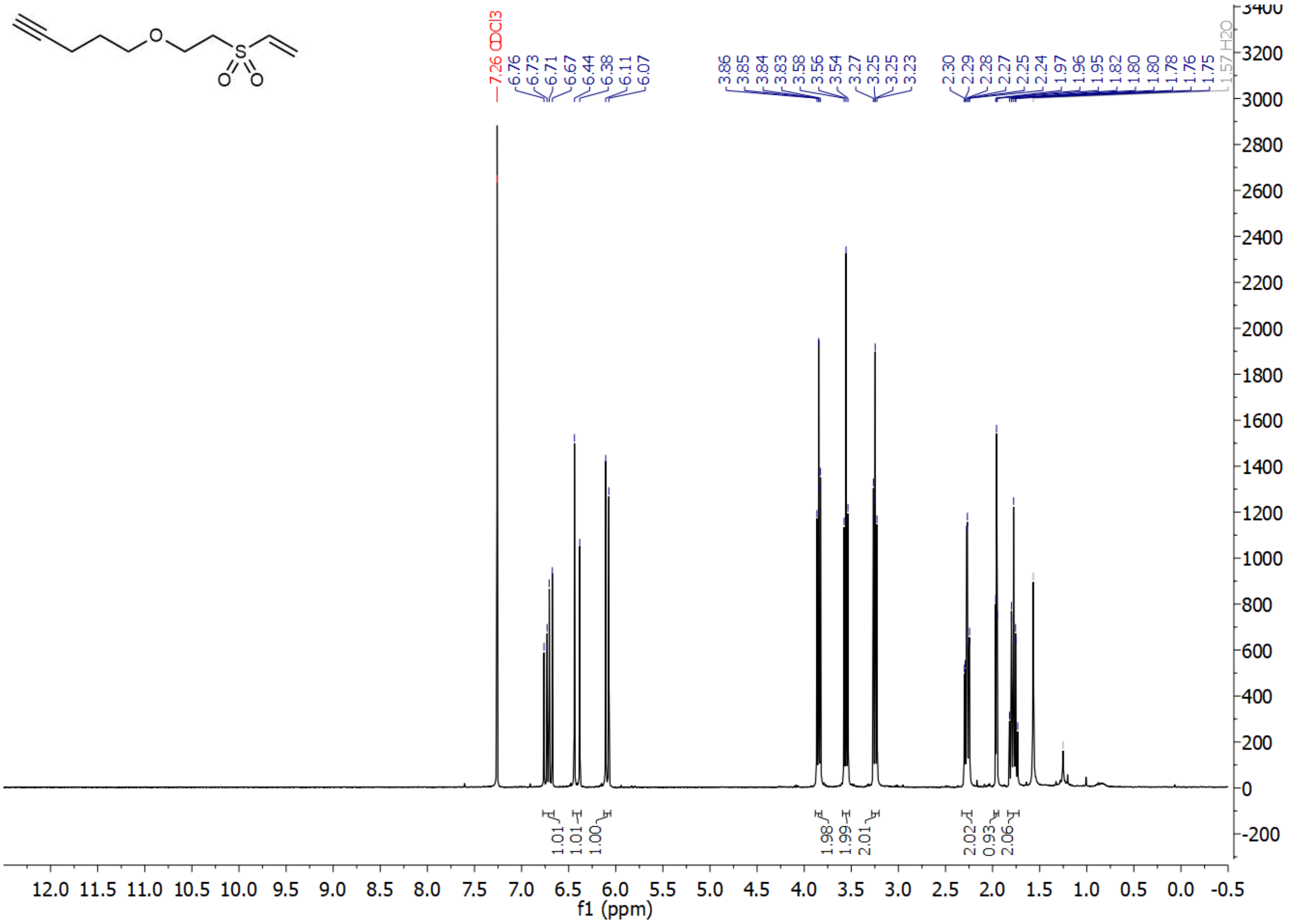

5-(2-(Vinylsulfonyl)ethoxy)pent-1-yne (**AlkVS-alkyne**) (^{13}C, 75.5 MHz, $CDCl_3$):

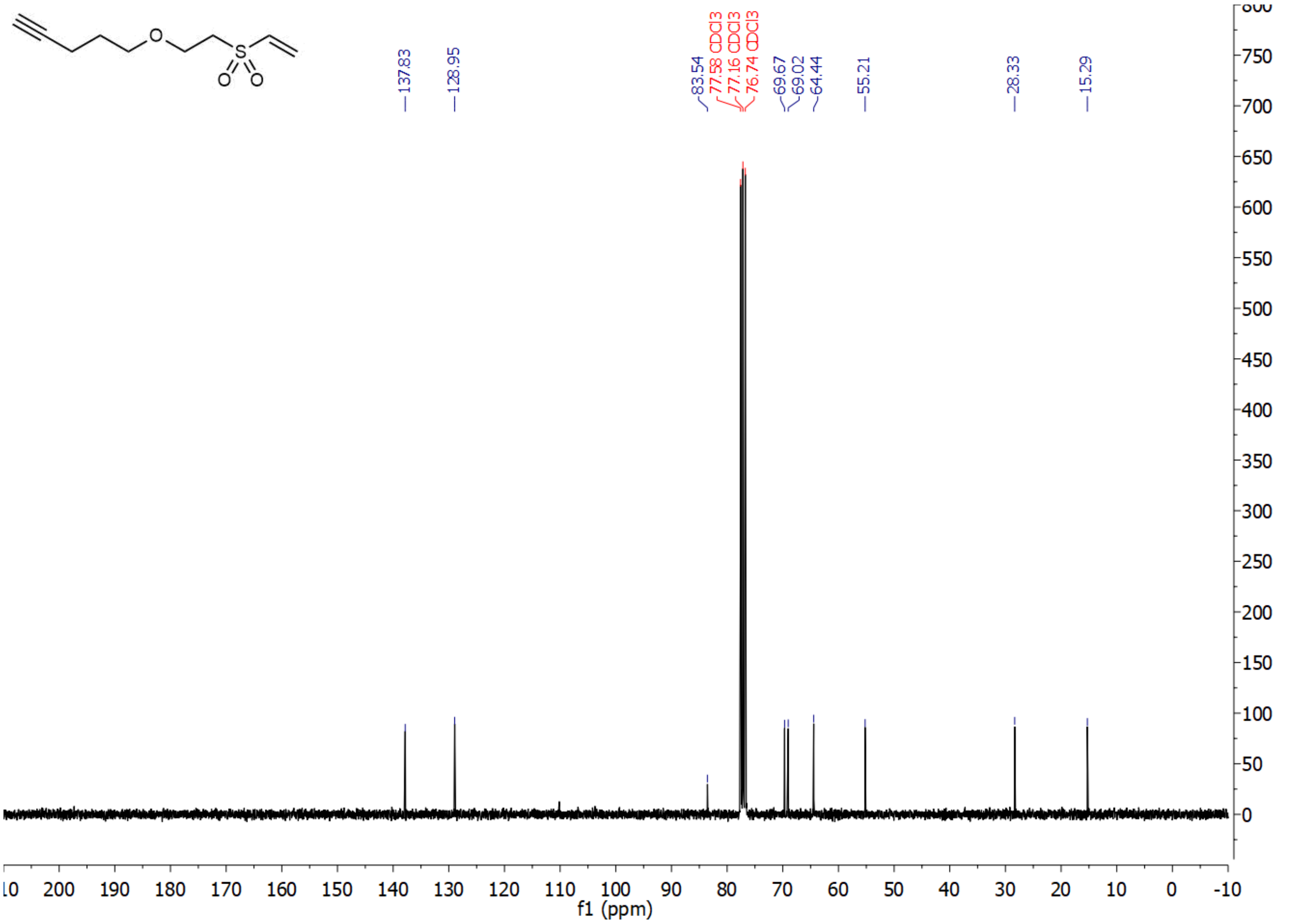

N-(Prop-2-yn-1-yl)-4-(vinylsulfonyl)benzamide (**ArVS-alkyne**) (^{1}H, 300 MHz, CD_3CN):

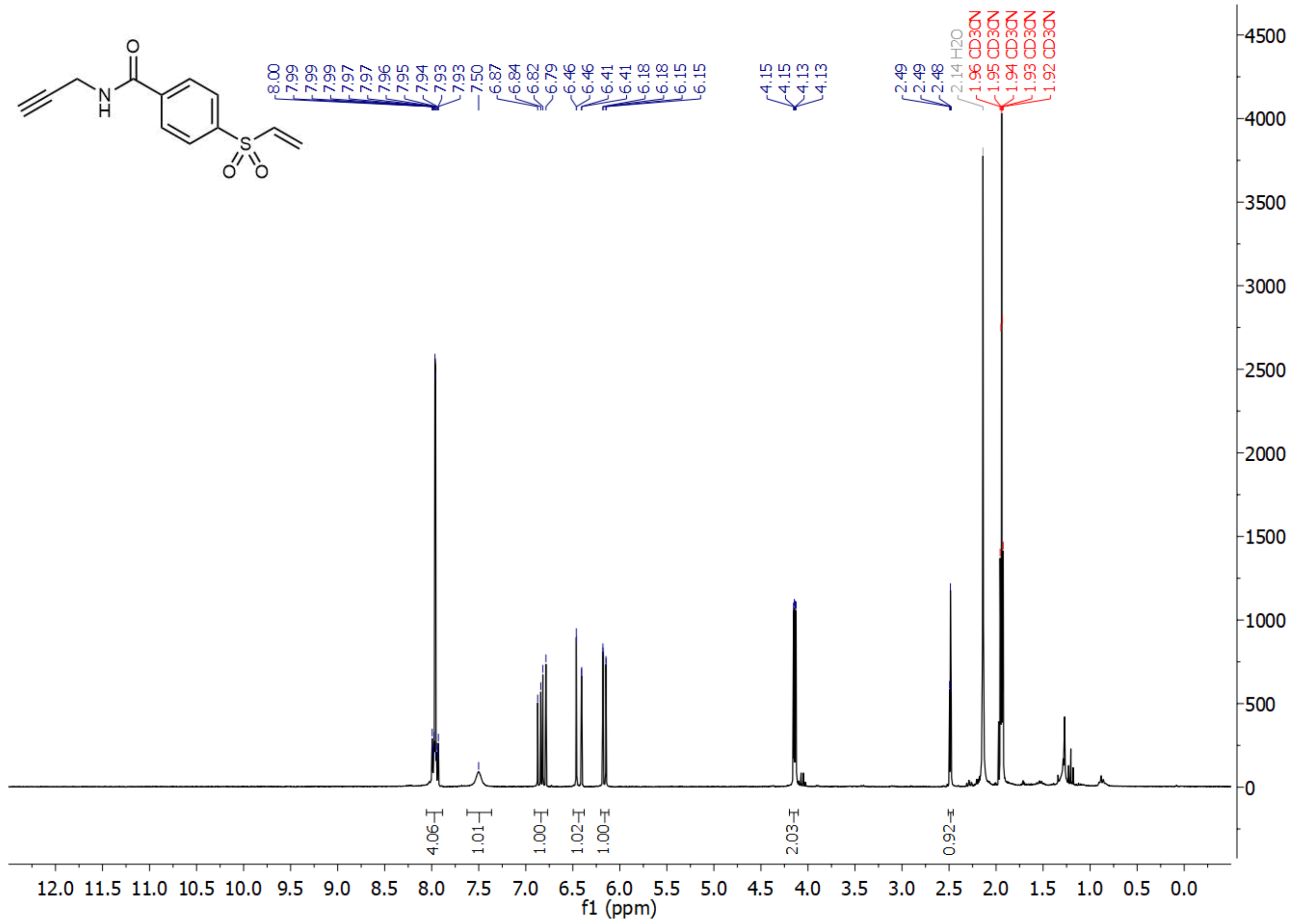

N-(Prop-2-yn-1-yl)-4-(vinylsulfonyl)benzamide (**ArVS-alkyne**) (^{13}C, 126 MHz, CD_3CN):

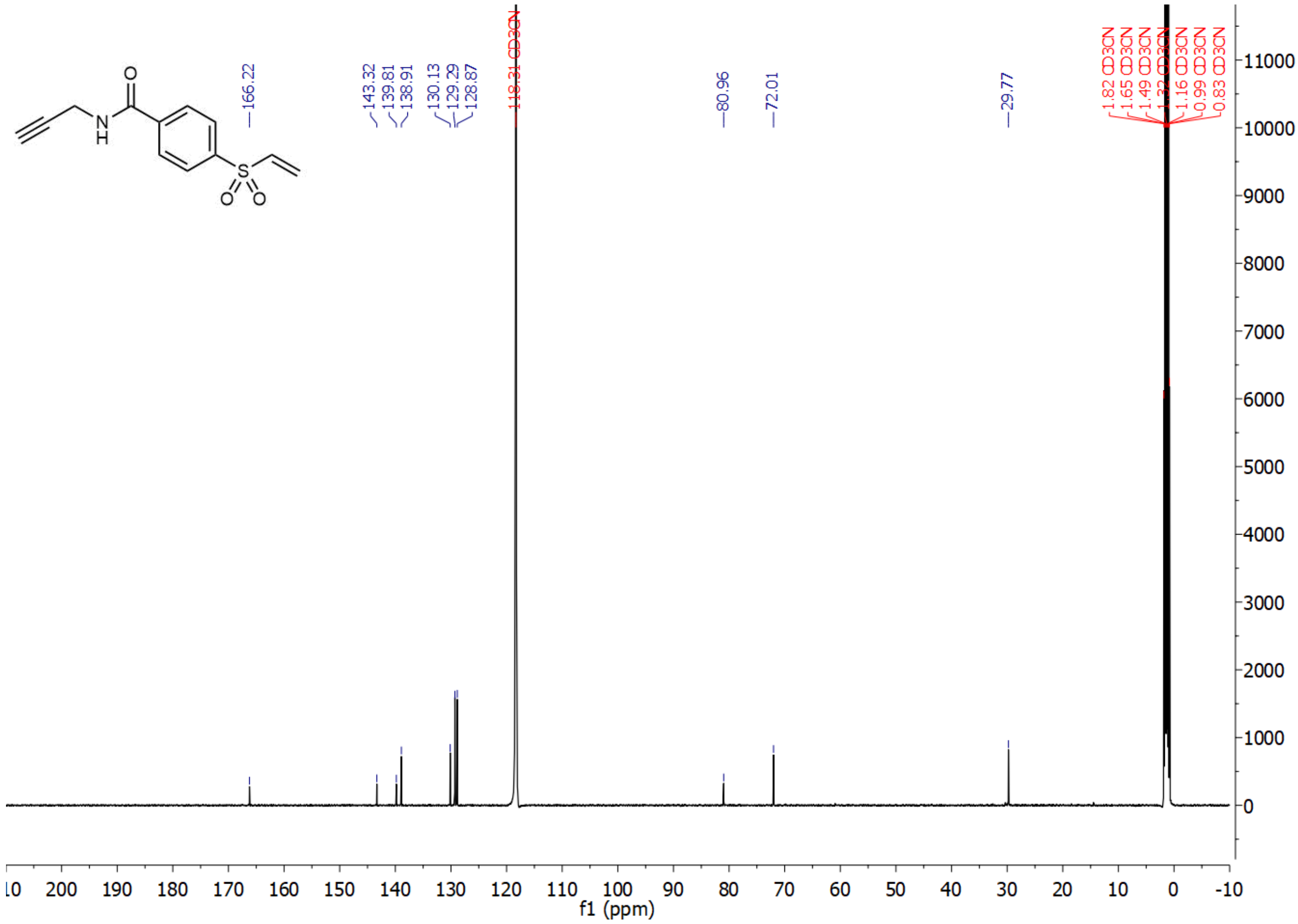

N-(Hex-5-yn-1-yl)ethenesulfonamide (**AlkVSA-alkyne**) (^1H, 300 MHz, $CDCl_3$):

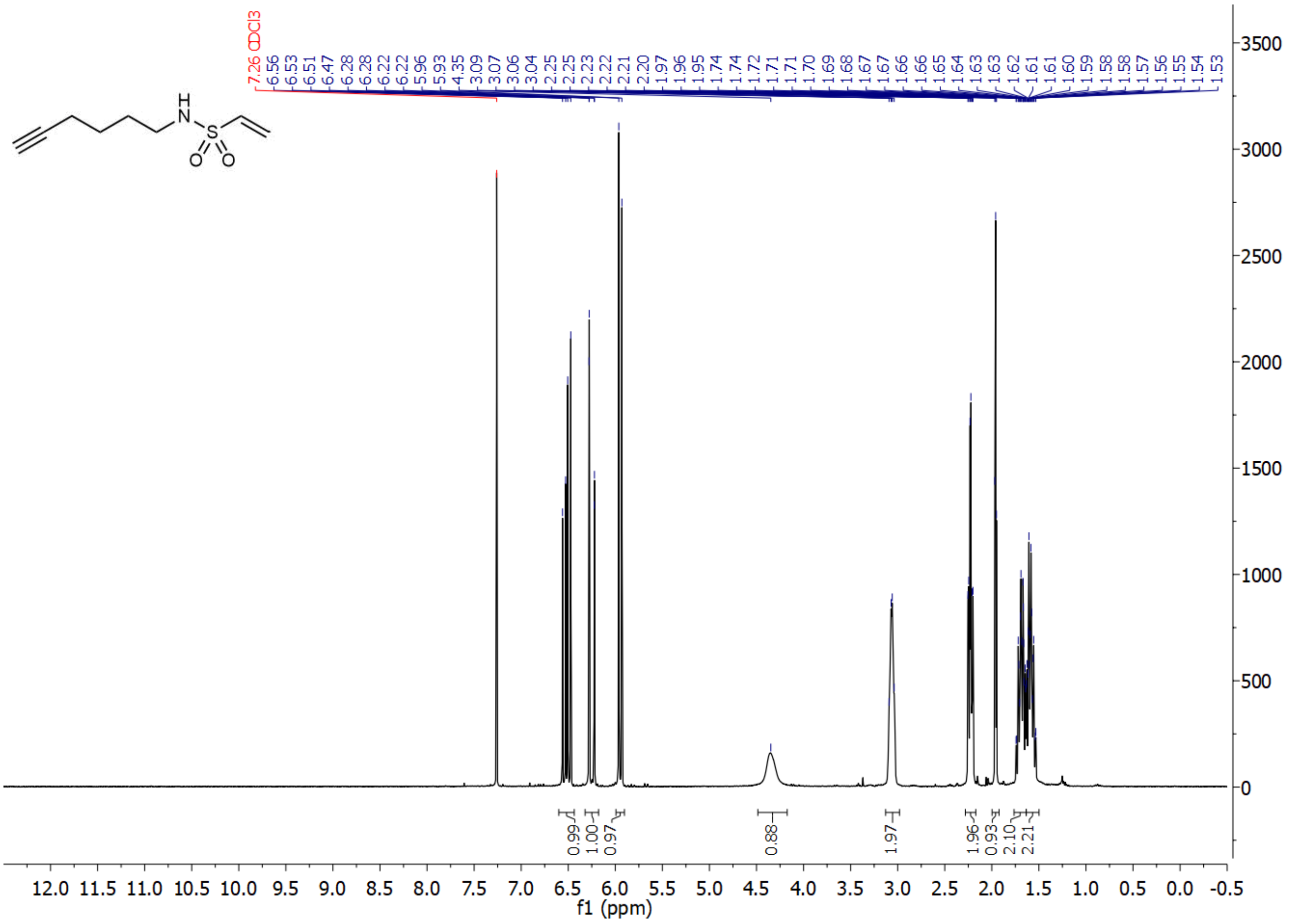

N-(Hex-5-yn-1-yl)ethenesulfonamide (**AlkVSA-alkyne**) (^{13}C, 101 MHz, $CDCl_3$):

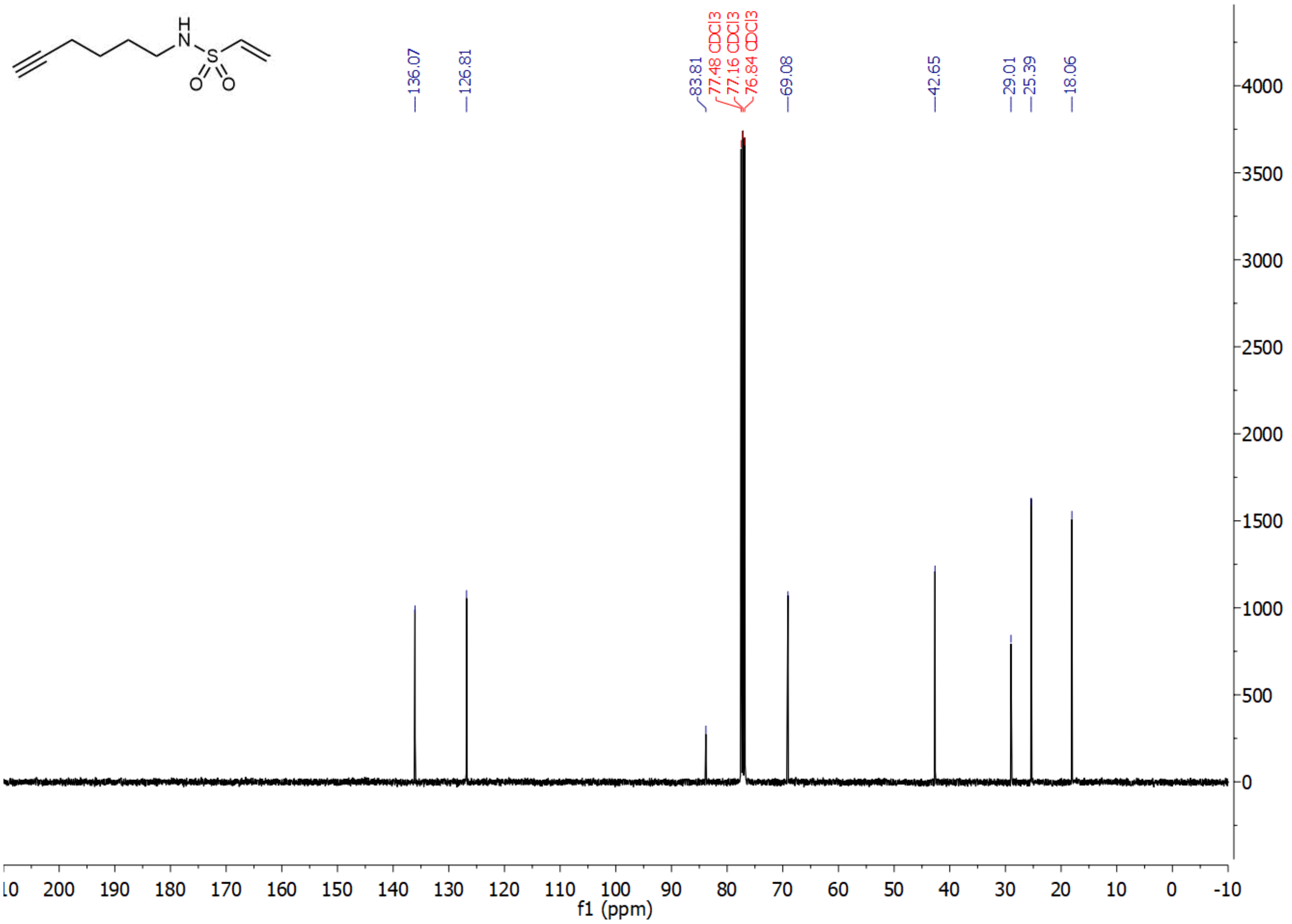

N-(4-Ethynylphenyl)ethenesulfonamide (**ArVSA-alkyne**) (^{1}H, 500 MHz, $CDCl_3$):

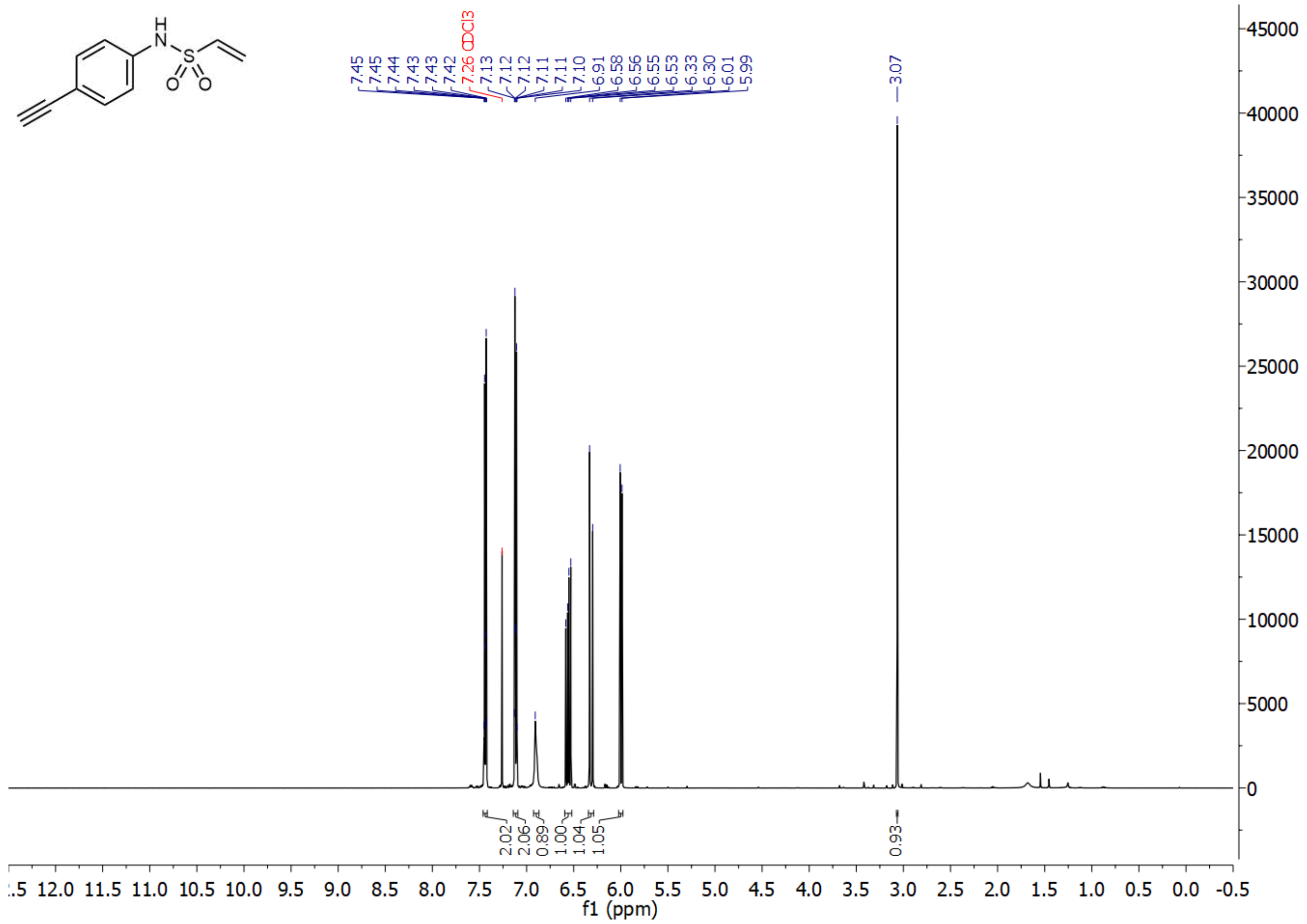

N-(4-Ethynylphenyl)ethenesulfonamide (**ArVSA-alkyne**) (^{13}C, 101 MHz, $CDCl_3$):

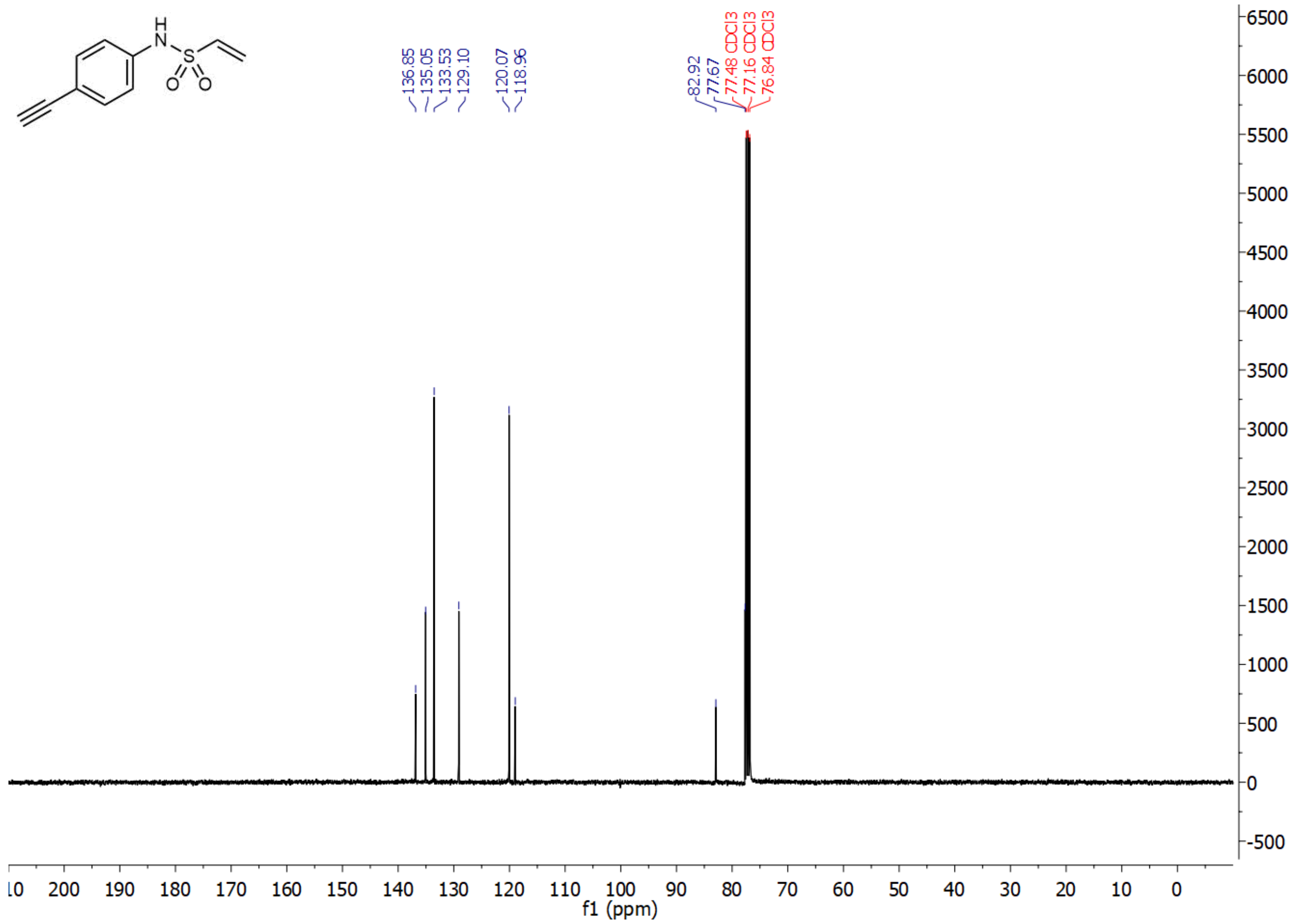

1-(2-Thioxothiazolidin-3-yl)hex-5-yn-1-one (**ATT-alkyne**) (^{1}H, 400 MHz, $CDCl_3$):

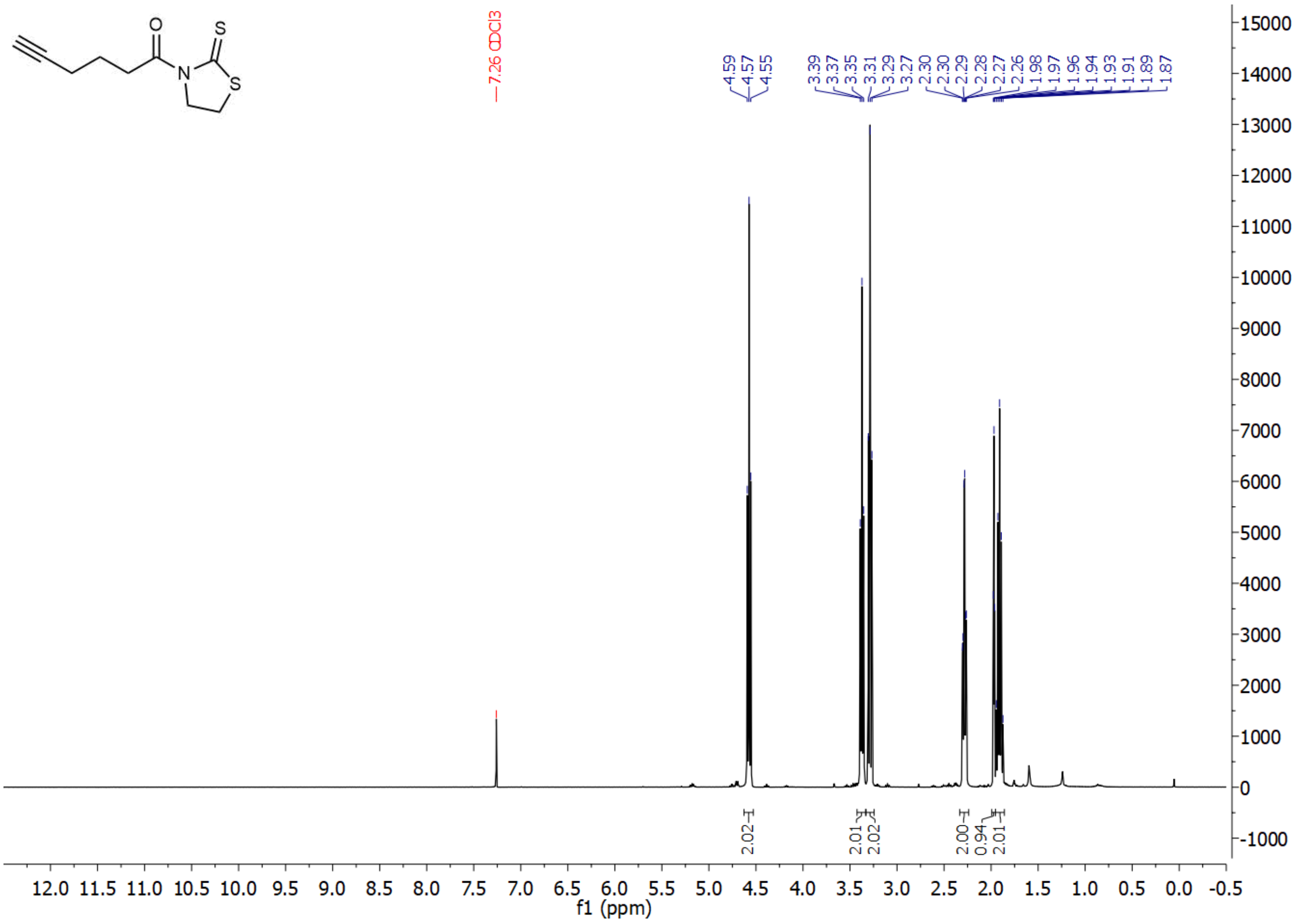

1-(2-Thioxothiazolidin-3-yl)hex-5-yn-1-one (**ATT-alkyne**) (^{13}C, 101 MHz, $CDCl_3$):

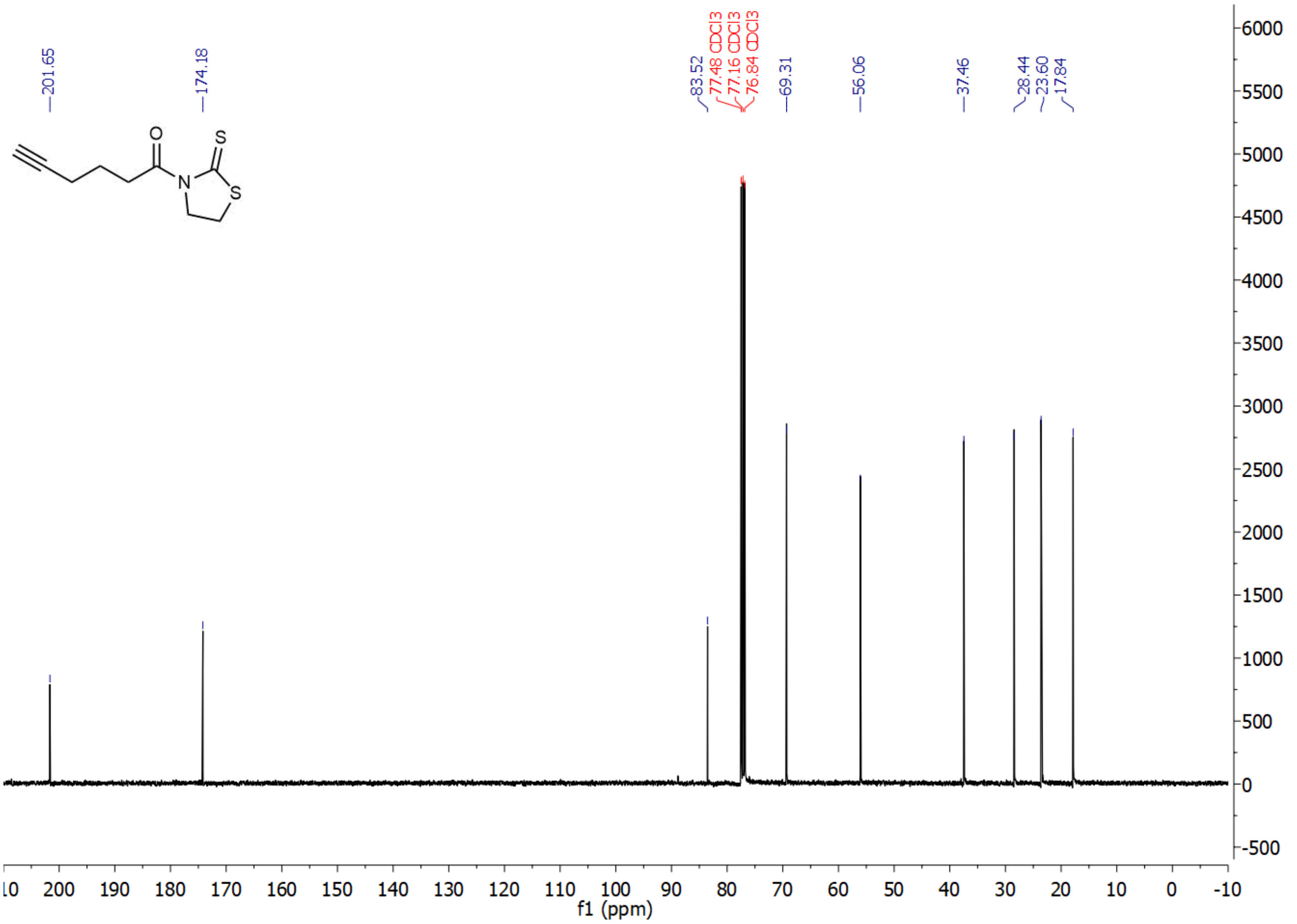

N-(Phenylsulfonyl)hex-5-ynamide (**11**) (^{1}H, 500 MHz, $CDCl_3$):

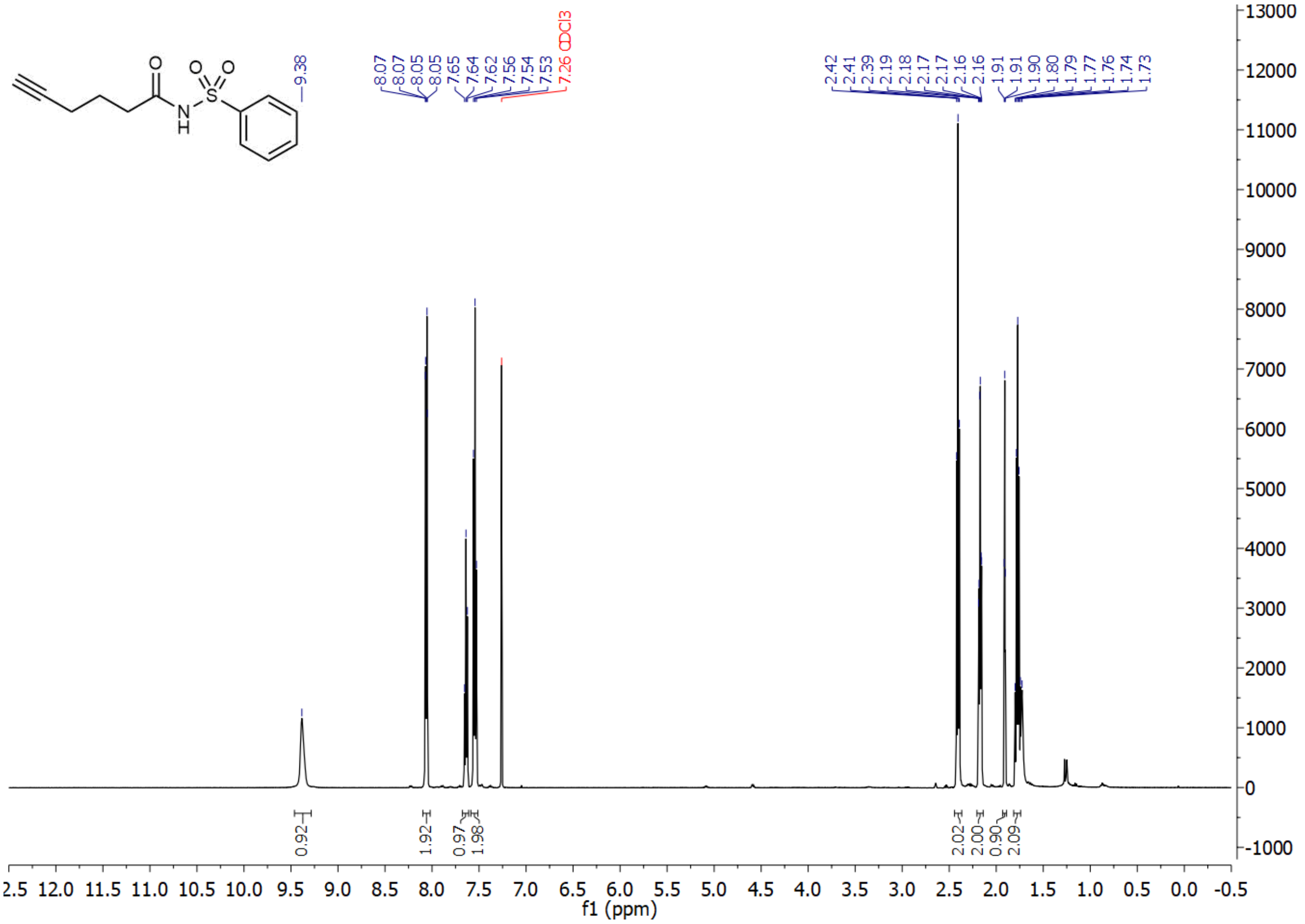

N-(Phenylsulfonyl)hex-5-ynamide (**11**) (^{13}C, 75.5 MHz, $CDCl_3$):

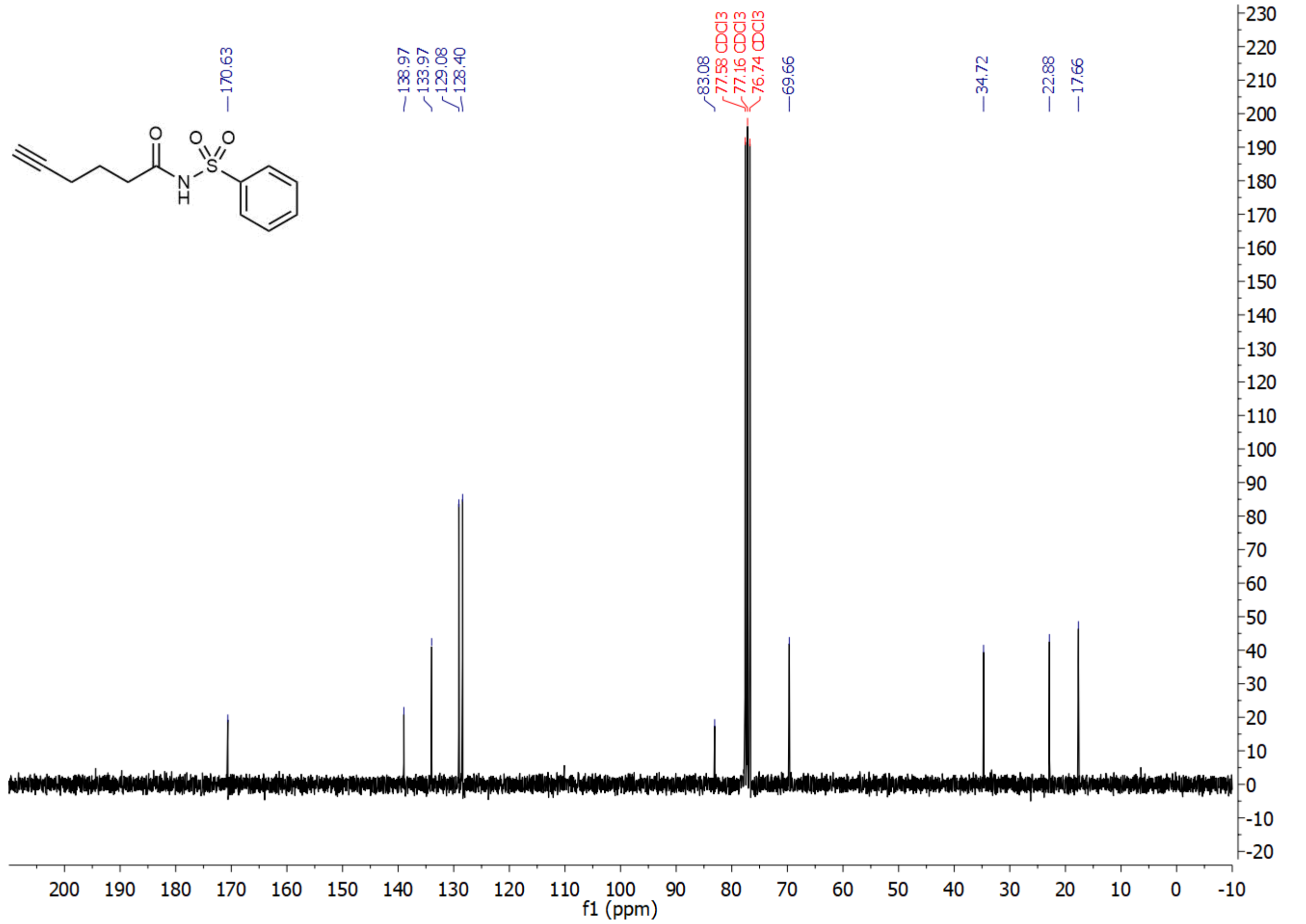

N-(Cyanomethyl)-*N*-(phenylsulfonyl)hex-5-ynamide (**NASA-alkyne**) (^{1}H, 500 MHz, $CDCl_3$):

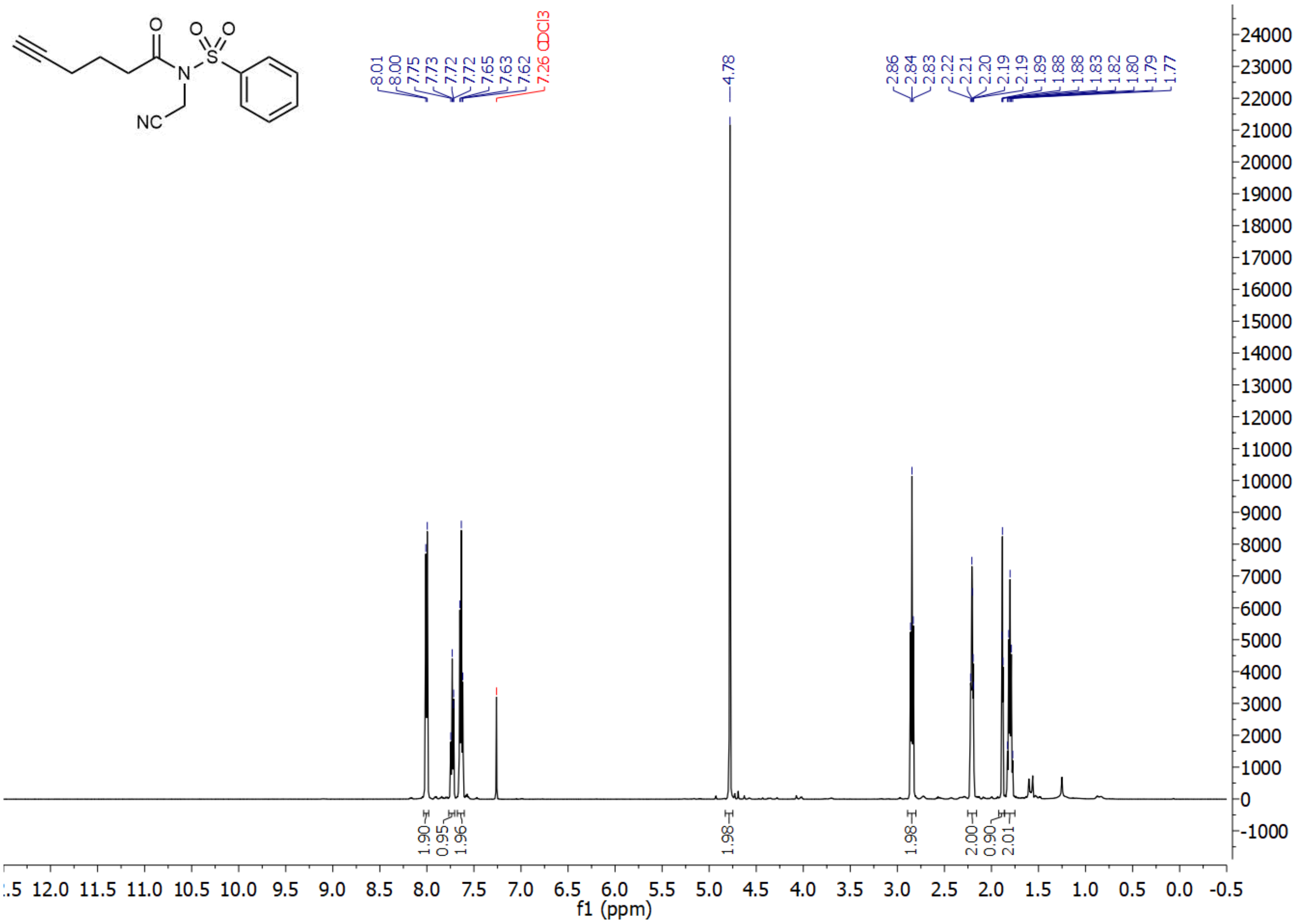

N-(Cyanomethyl)-*N*-(phenylsulfonyl)hex-5-ynamide (**NASA-alkyne**) (^{13}C, 75.5 MHz, $CDCl_3$):

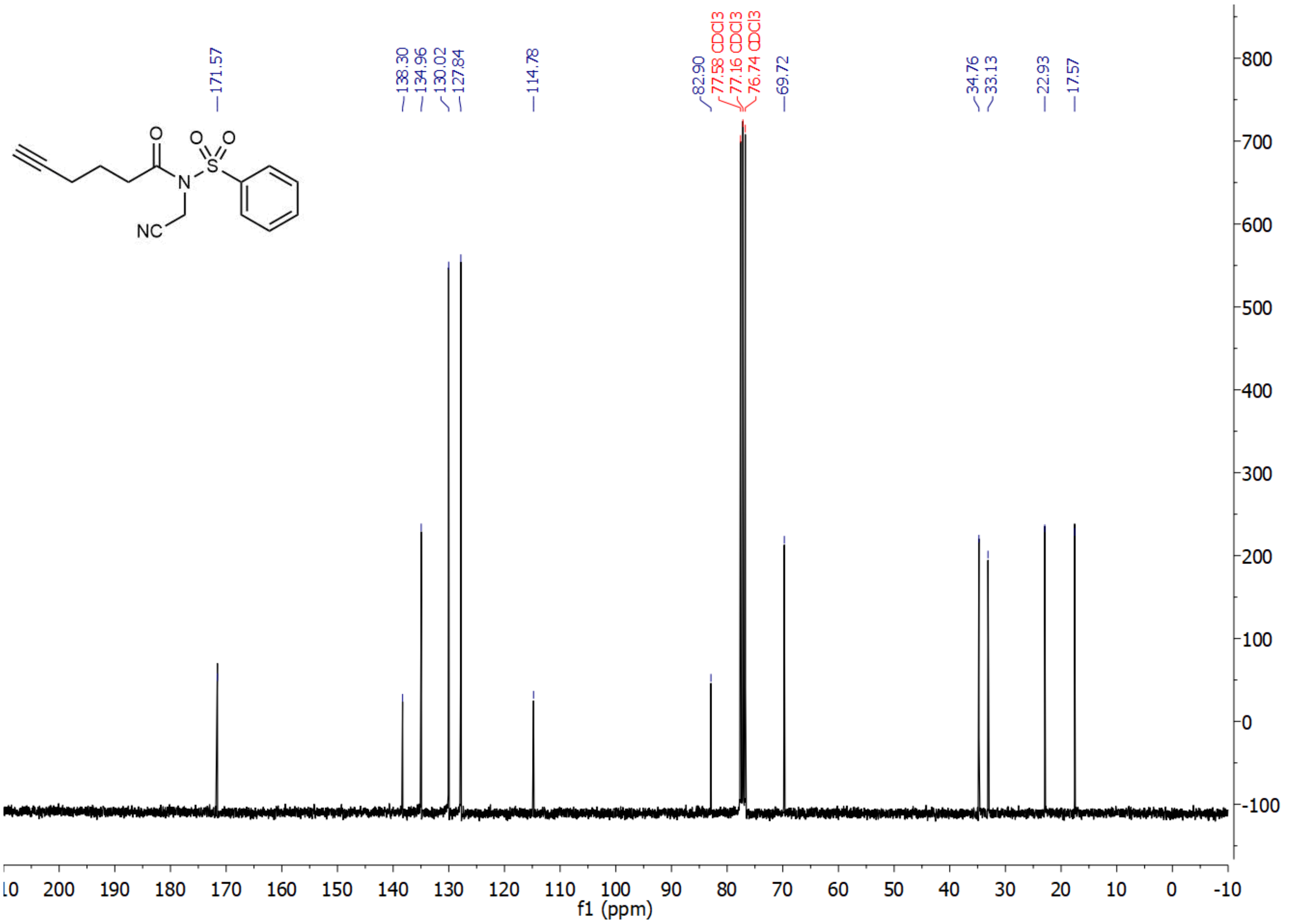

3-(But-3-yn-1-ylamino)-4-methoxycyclobut-3-ene-1,2-dione (**AlkSq-alkyne**) (^{1}H, 400 MHz, DMSO-d6):

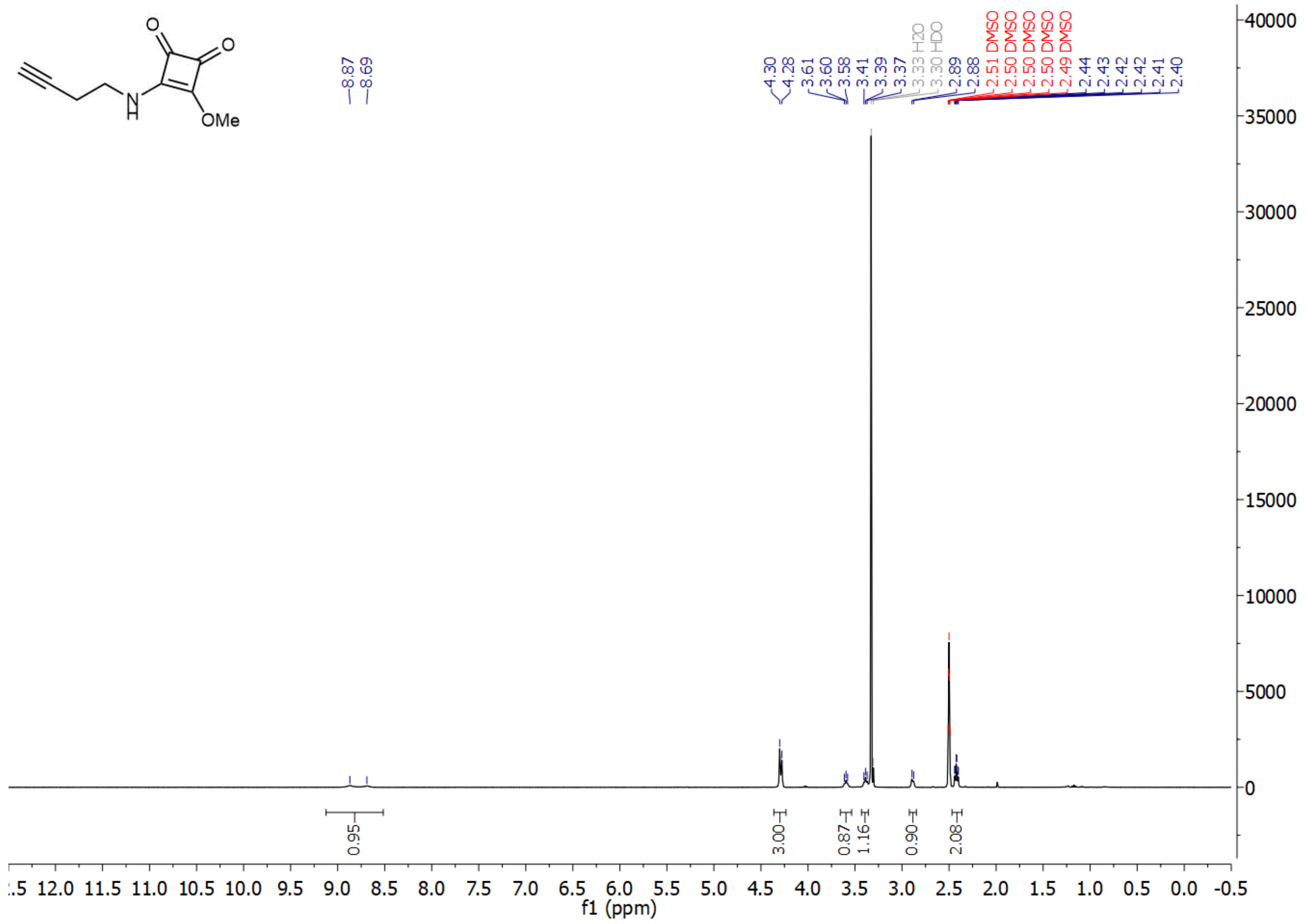

3-(But-3-yn-1-ylamino)-4-methoxycyclobut-3-ene-1,2-dione (**AlkSq-alkyne**) (^{13}C, 101 MHz, DMSO-d6):

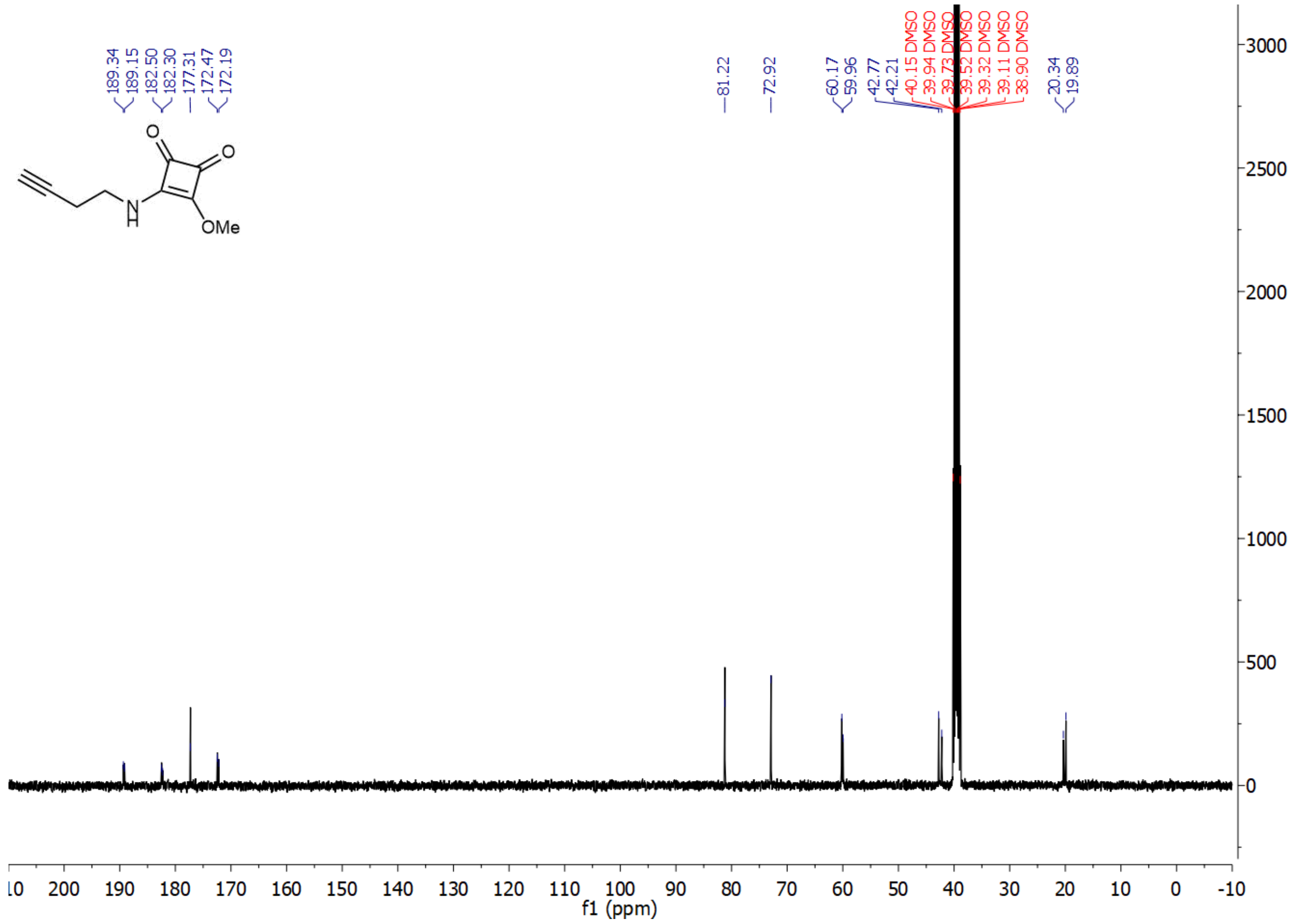

3-Methoxy-4-((4-ethynylphenyl)amino)cyclobut-3-ene-1,2-dione (**ArSq-alkyne**) (^{1}H, 400 MHz, DMSO-d$_6$):

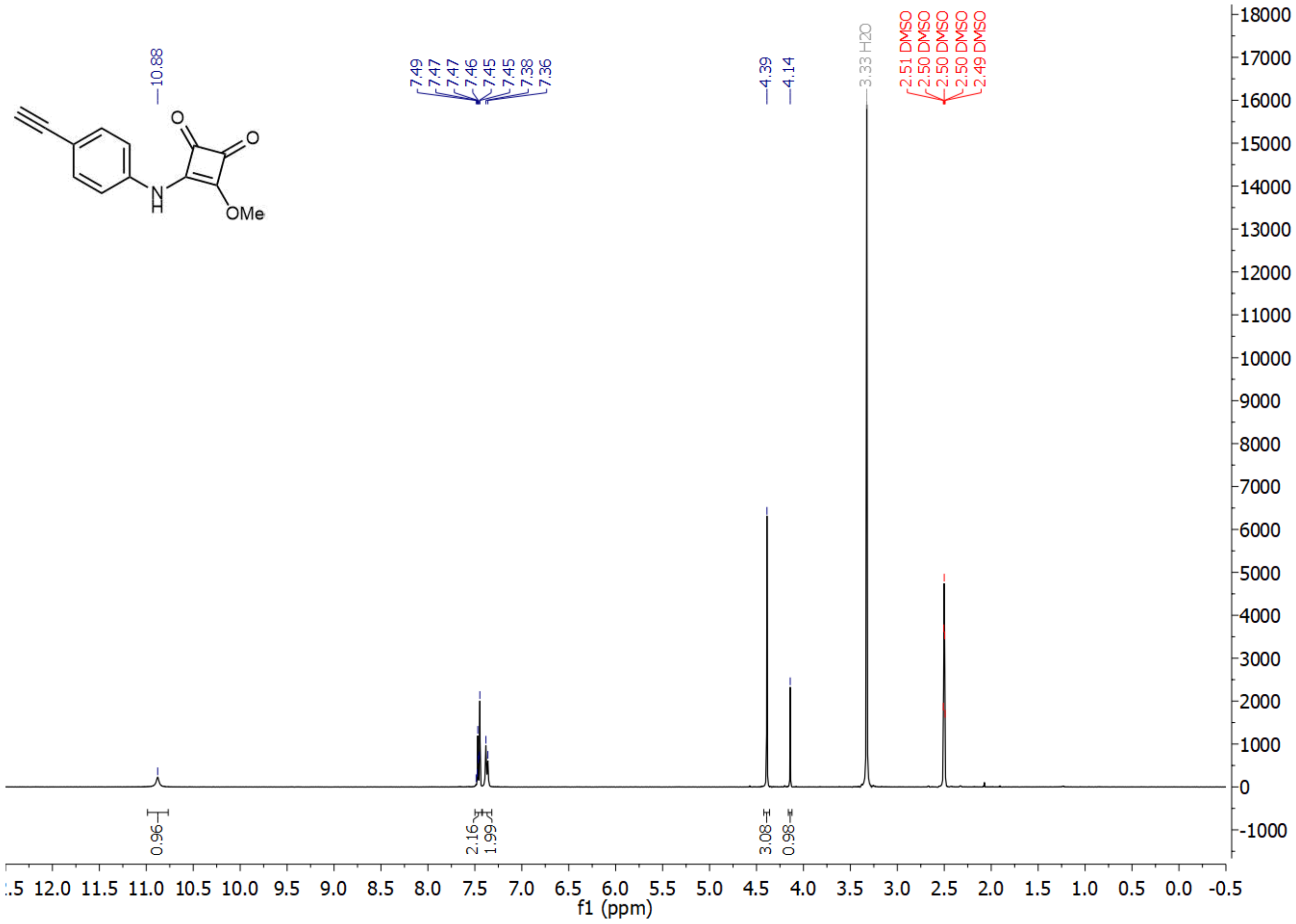

3-Methoxy-4-((4-ethynylphenyl)amino)cyclobut-3-ene-1,2-dione (**ArSq-alkyne**) (^{13}C, 126 MHz, DMSO-d$_6$):

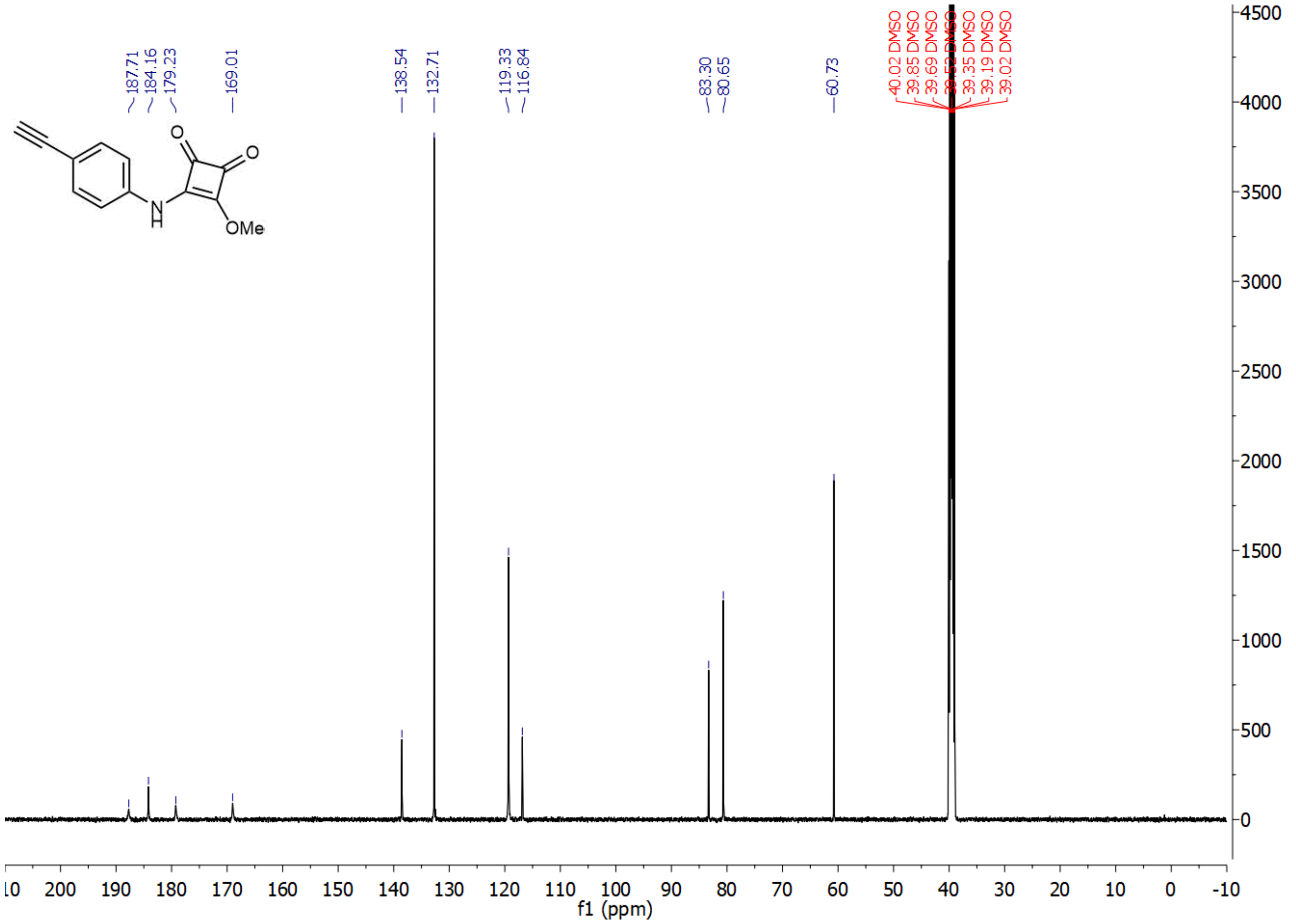

5-Hydroxy-2-((trimethylsilyl)ethynyl)benzaldehyde (**12**) (^{1}H, 400 MHz, $CDCl_3$):

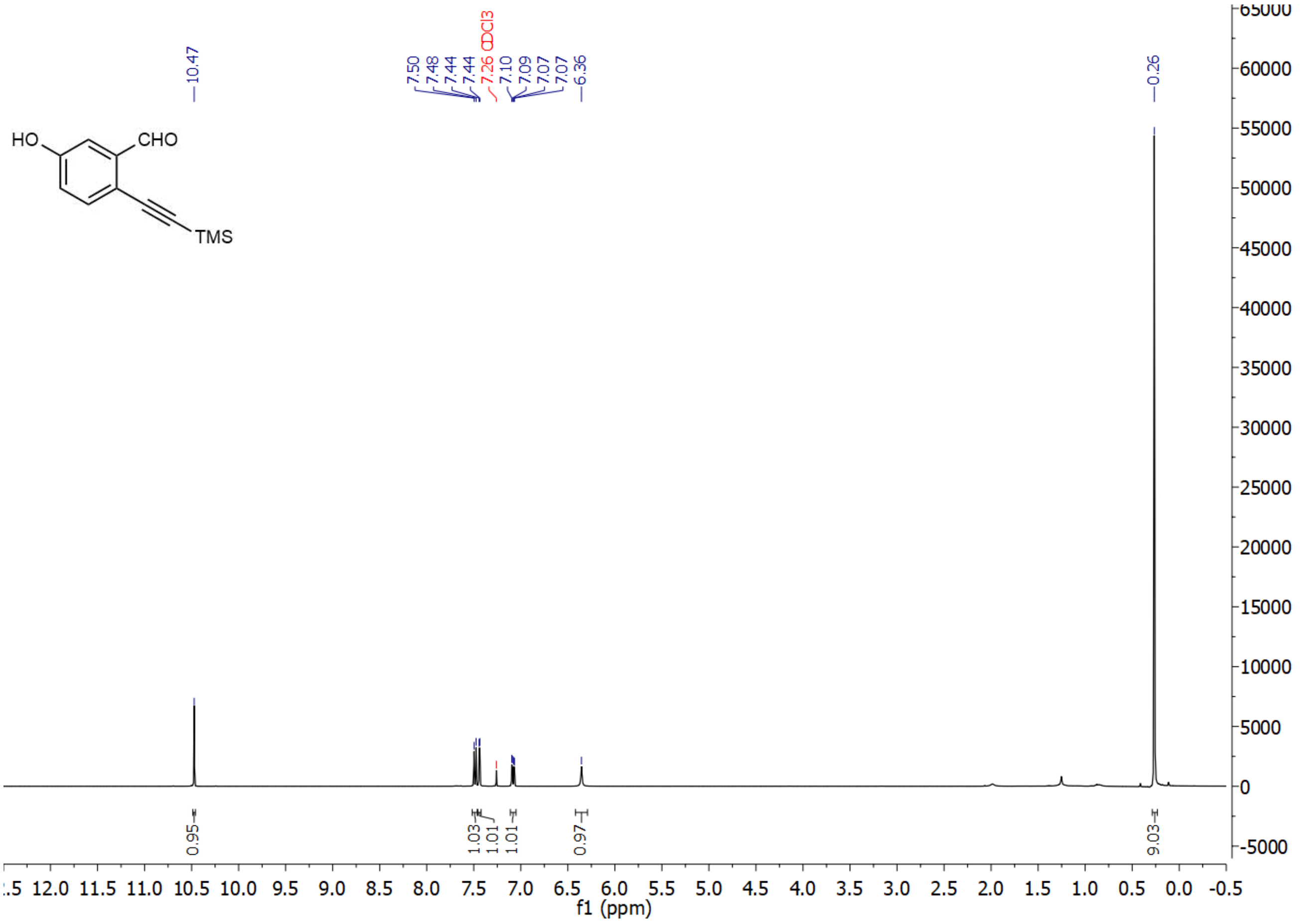

5-Hydroxy-2-((trimethylsilyl)ethynyl)benzaldehyde (**12**) (^{13}C, 101 MHz, $CDCl_3$):

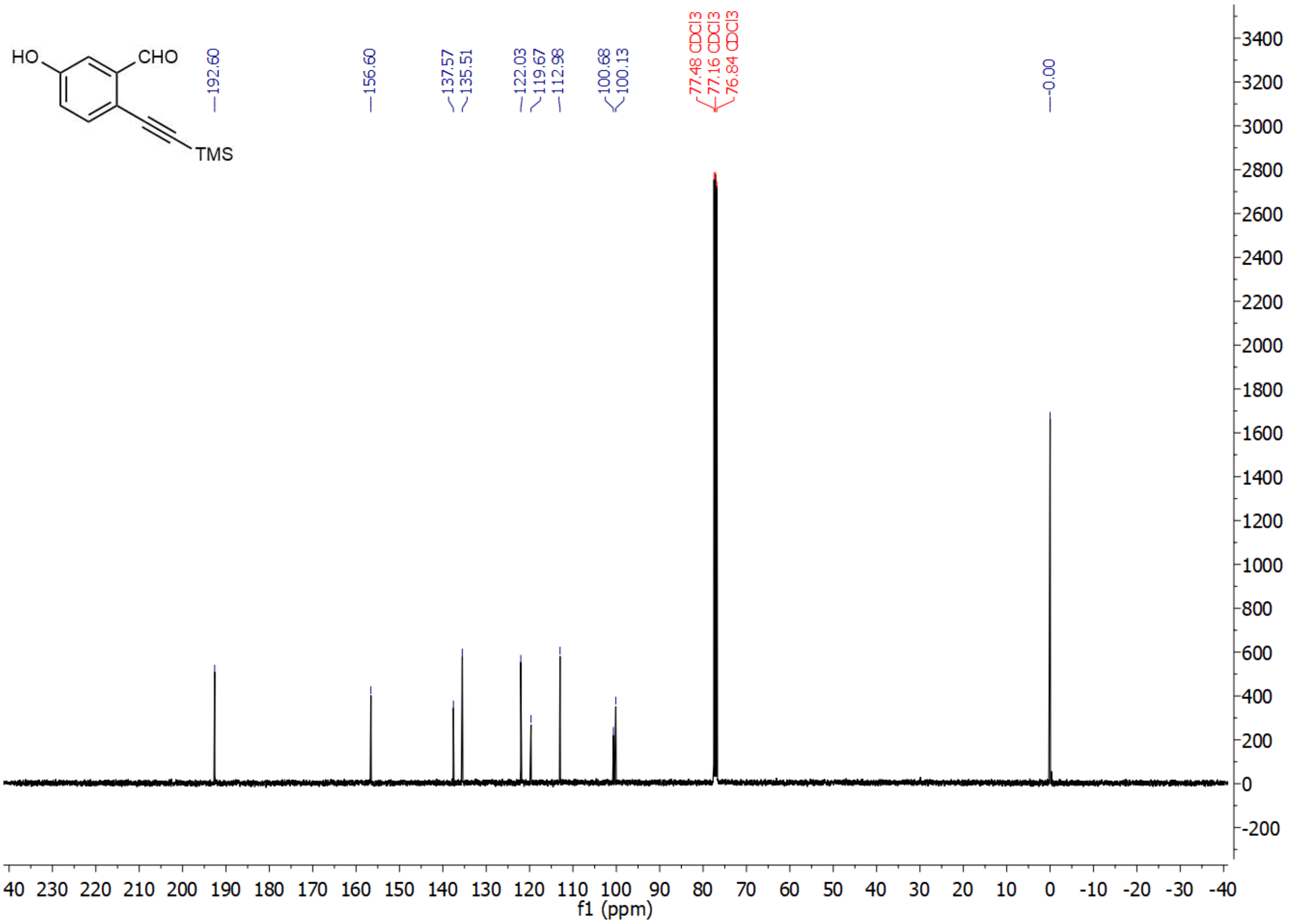

2-Ethynyl-5-(prop-2-yn-1-yloxy)benzaldehyde (**EBA-alkyne**) (^{1}H, 400 MHz, $CDCl_3$):

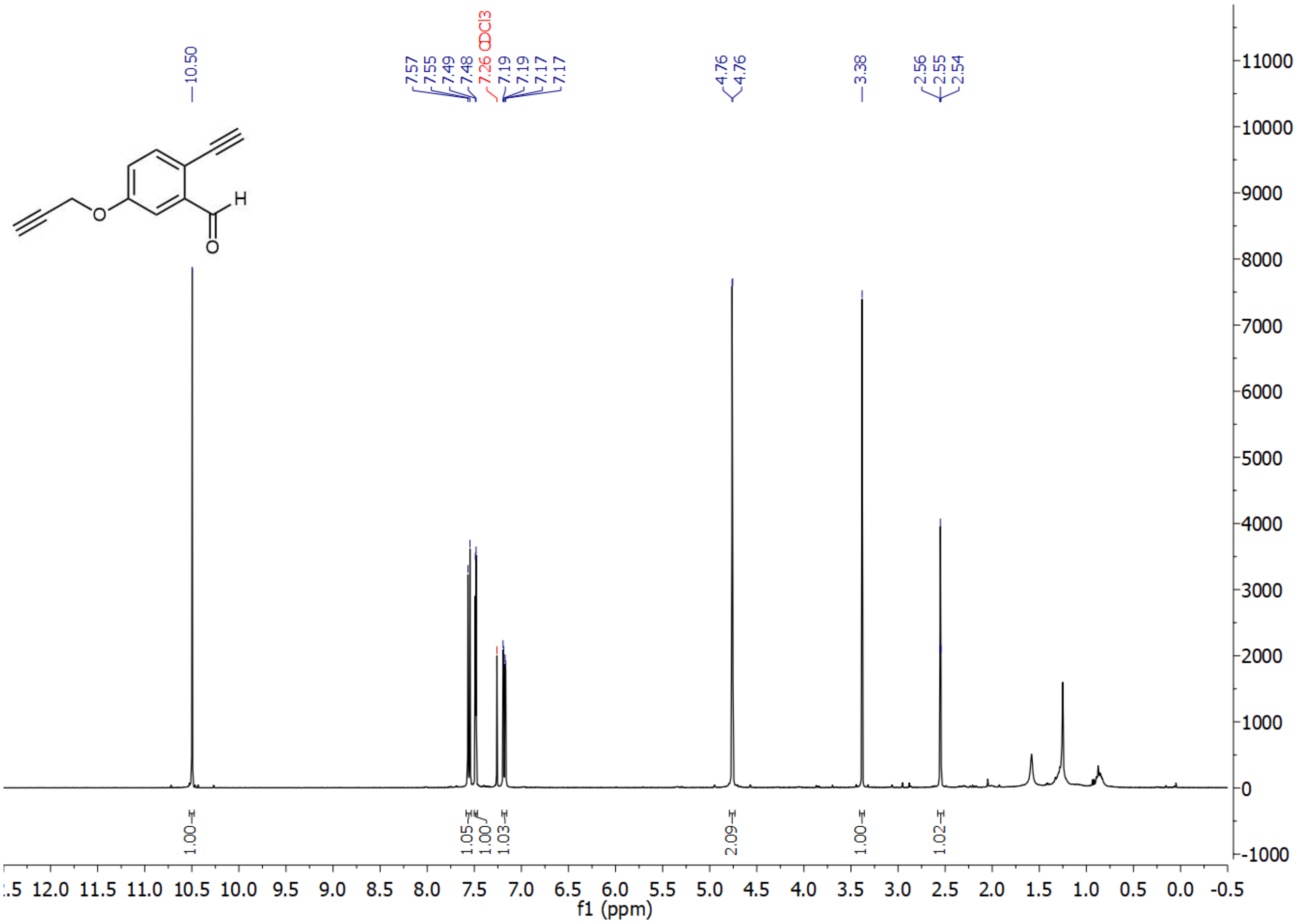

2-Ethynyl-5-(prop-2-yn-1-yloxy)benzaldehyde (**EBA-alkyne**) (^{13}C, 101 MHz, $CDCl_3$):

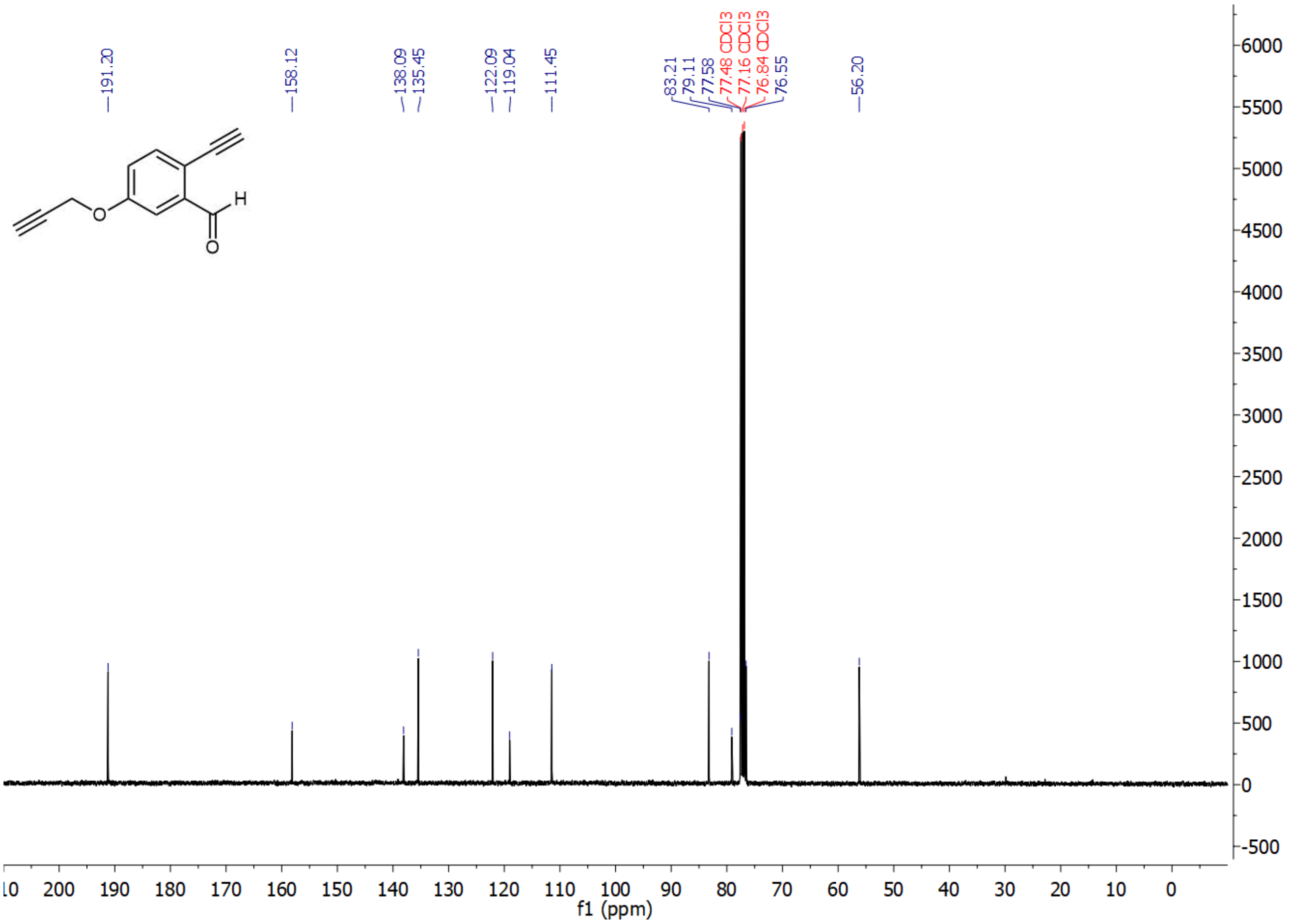

4-(Hydroxymethyl)-3-nitro-*N*-(prop-2-yn-1-yl)benzamide (***o*NBA-alkyne**) (^{1}H, 300 MHz, CD_3CN):

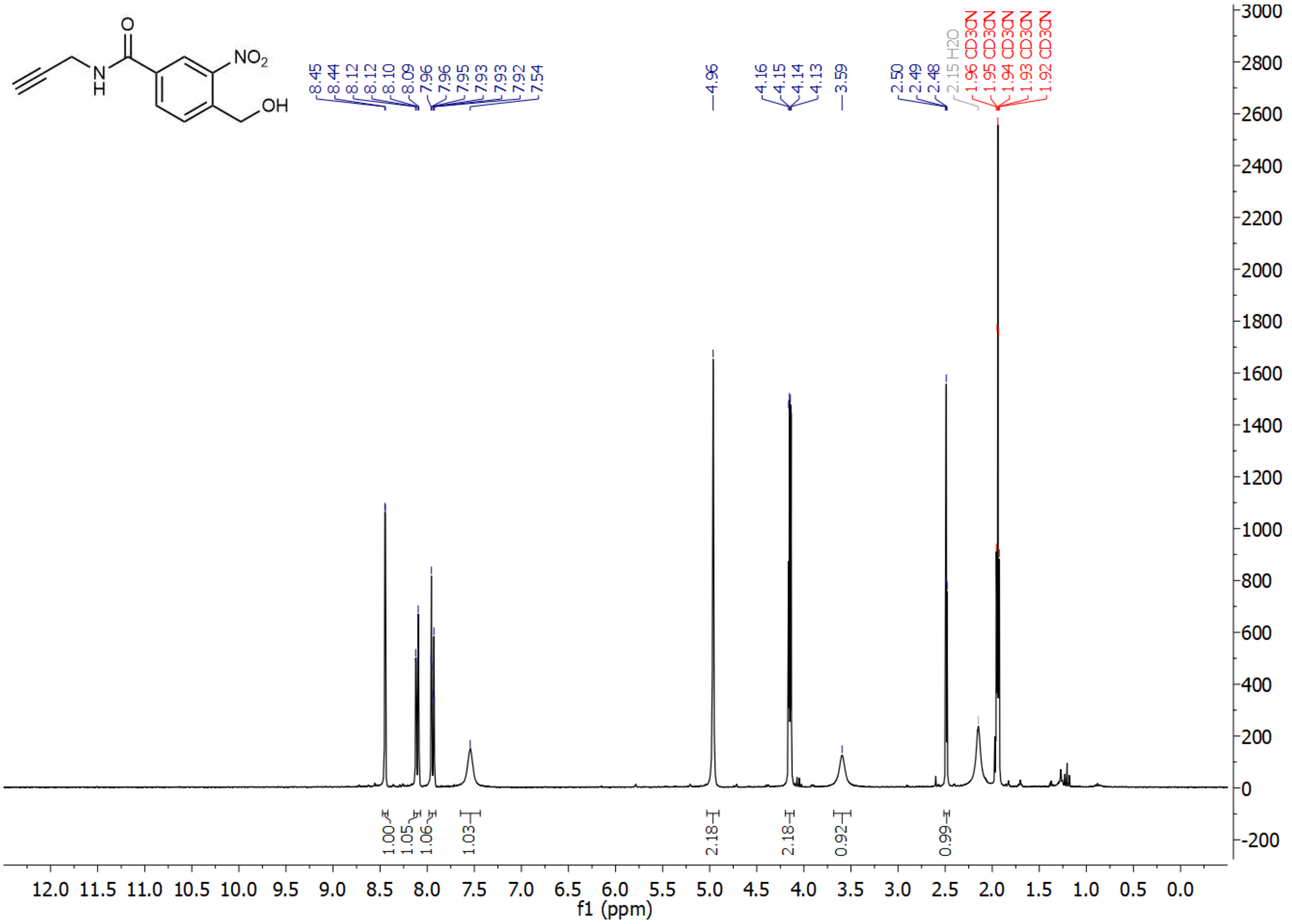

4-(Hydroxymethyl)-3-nitro-*N*-(prop-2-yn-1-yl)benzamide (***o*NBA-alkyne**) (^{13}C, 101 MHz, CD_3CN):

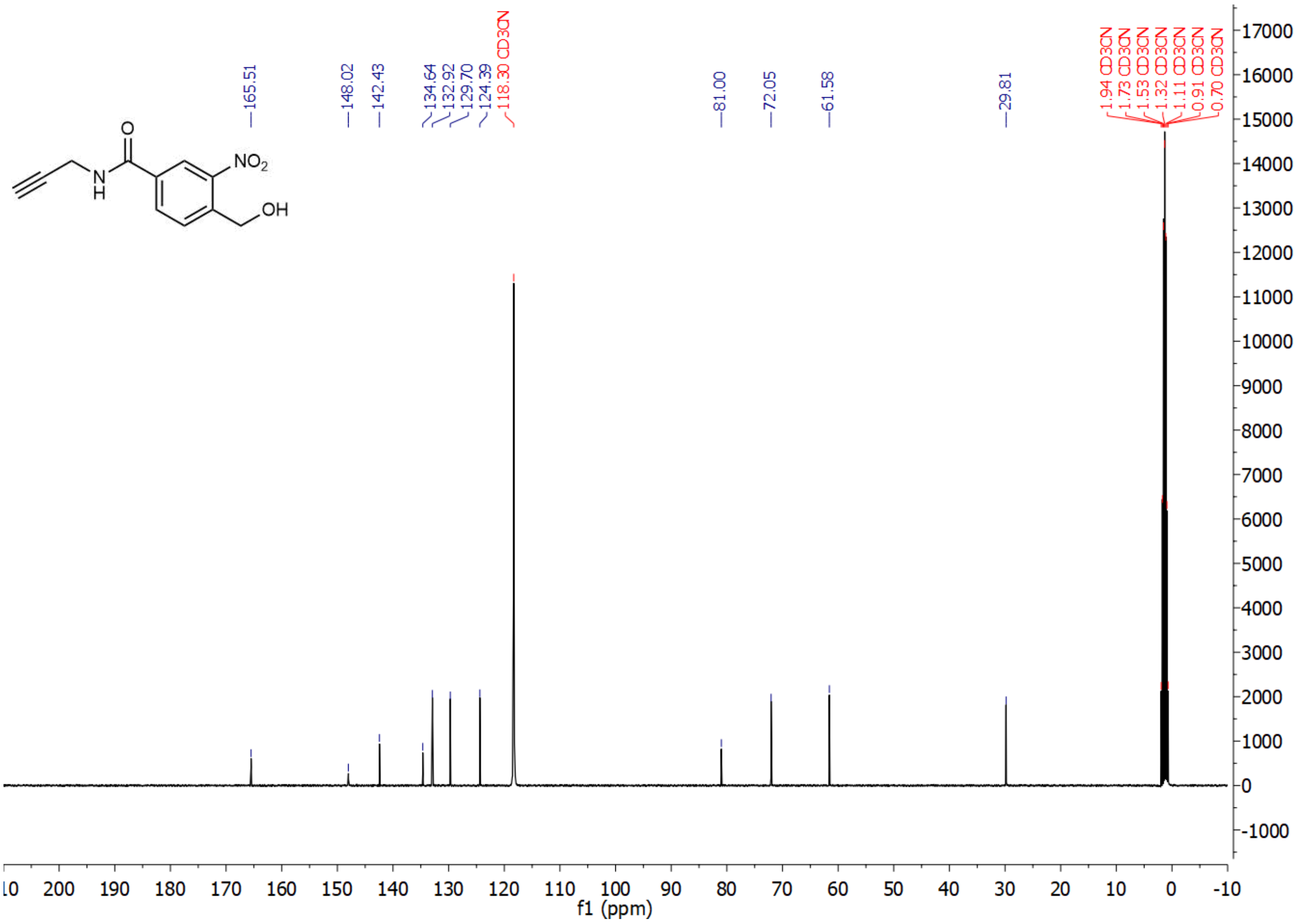

1-(But-3-yn-1-yl)-1*H*-1,2,3-triazole-4-carbaldehyde (**TCA-alkyne**) (^{1}H, 400 MHz, $CDCl_3$):

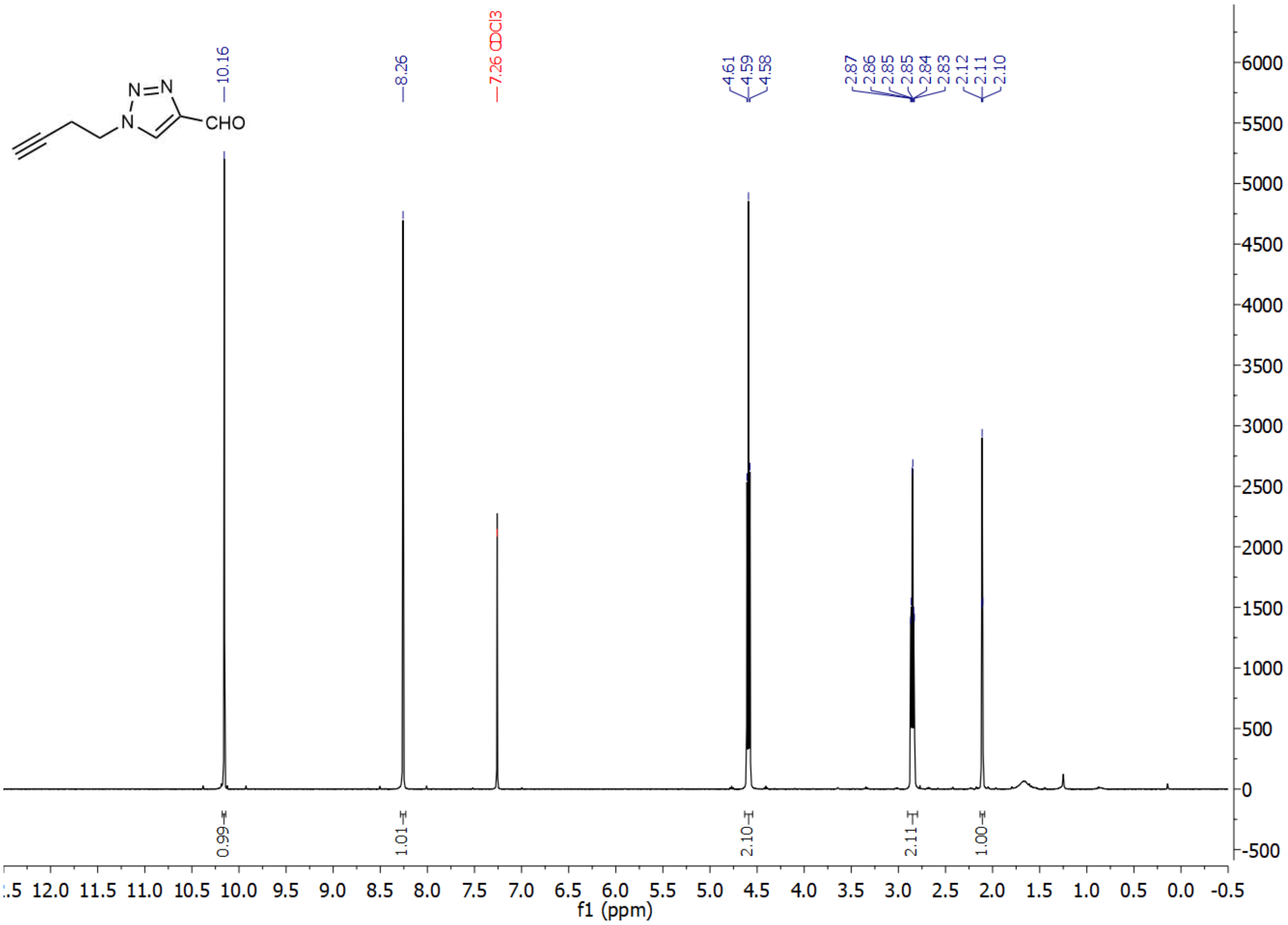

1-(But-3-yn-1-yl)-1*H*-1,2,3-triazole-4-carbaldehyde (**TCA-alkyne**) (^{13}C, 101 MHz, $CDCl_3$):

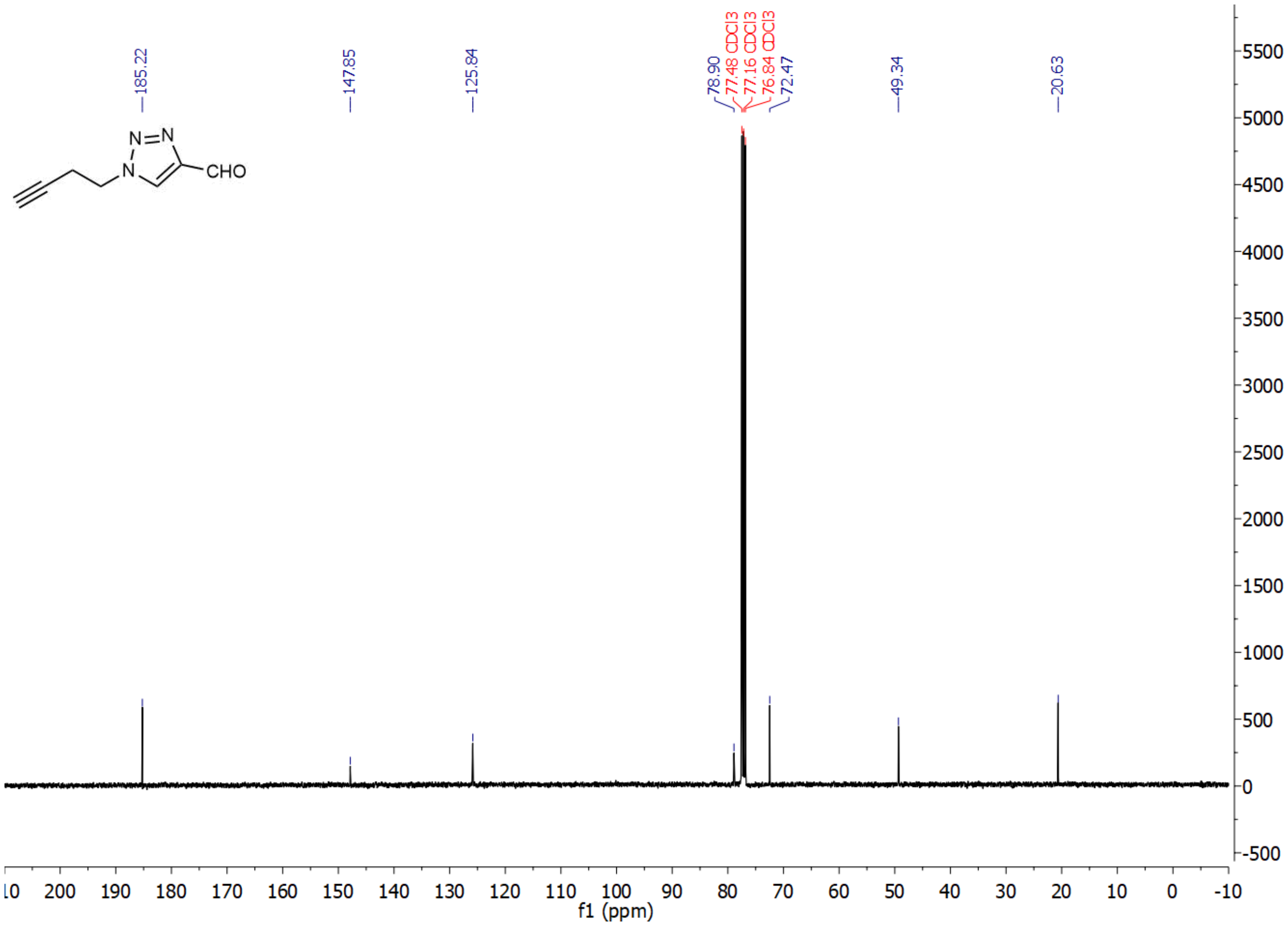

2-Ethyl-5-(3-(hex-5-yn-1-yloxy)phenyl)isoxazol-2-ium trifluoromethanesulfonate (**Isx-alkyne**) (^{1}H, 400 MHz, $CDCl_3$):

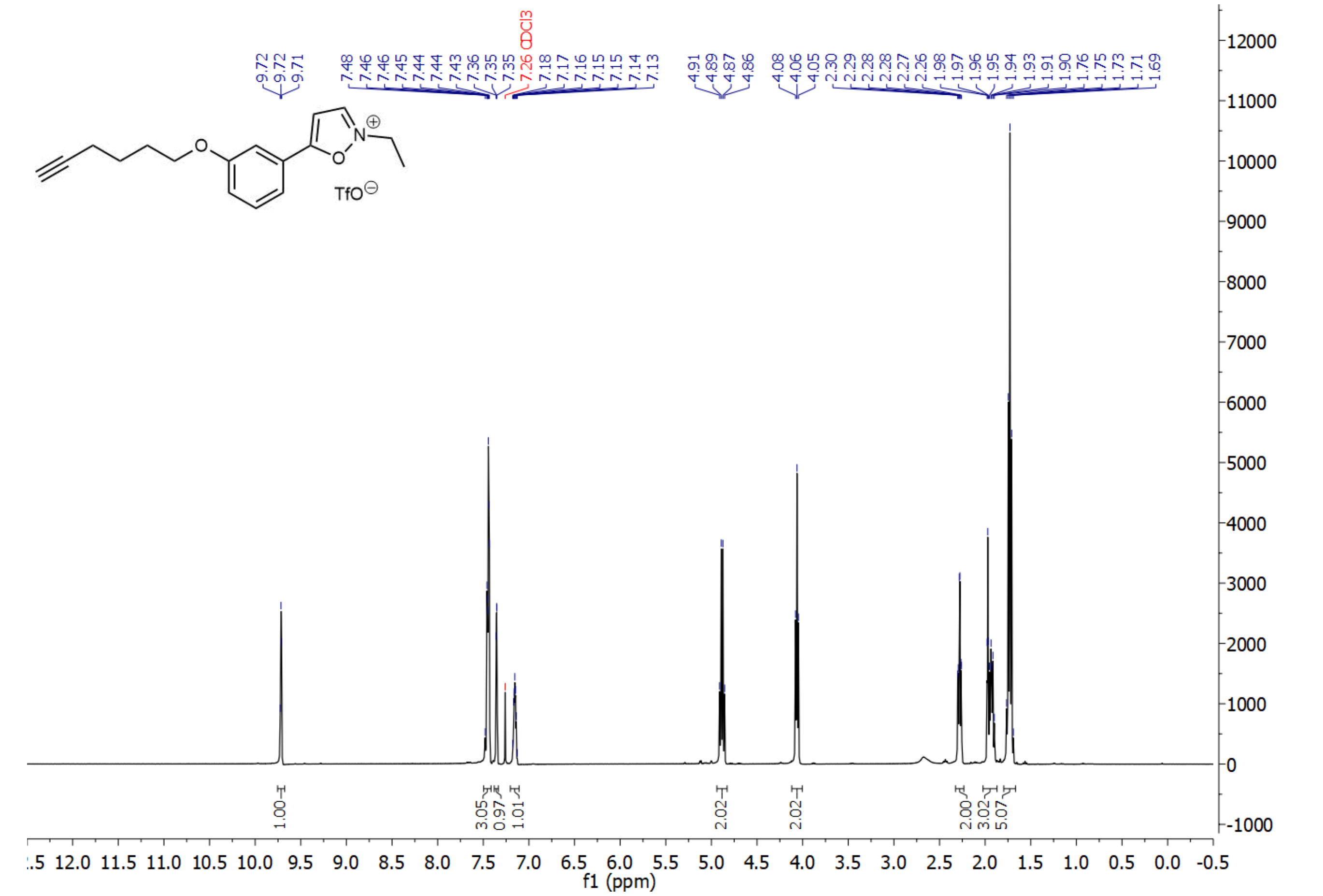

2-Ethyl-5-(3-(hex-5-yn-1-yloxy)phenyl)isoxazol-2-ium trifluoromethanesulfonate (**Isx-alkyne**) (^{13}C, 101 MHz, $CDCl_3$):

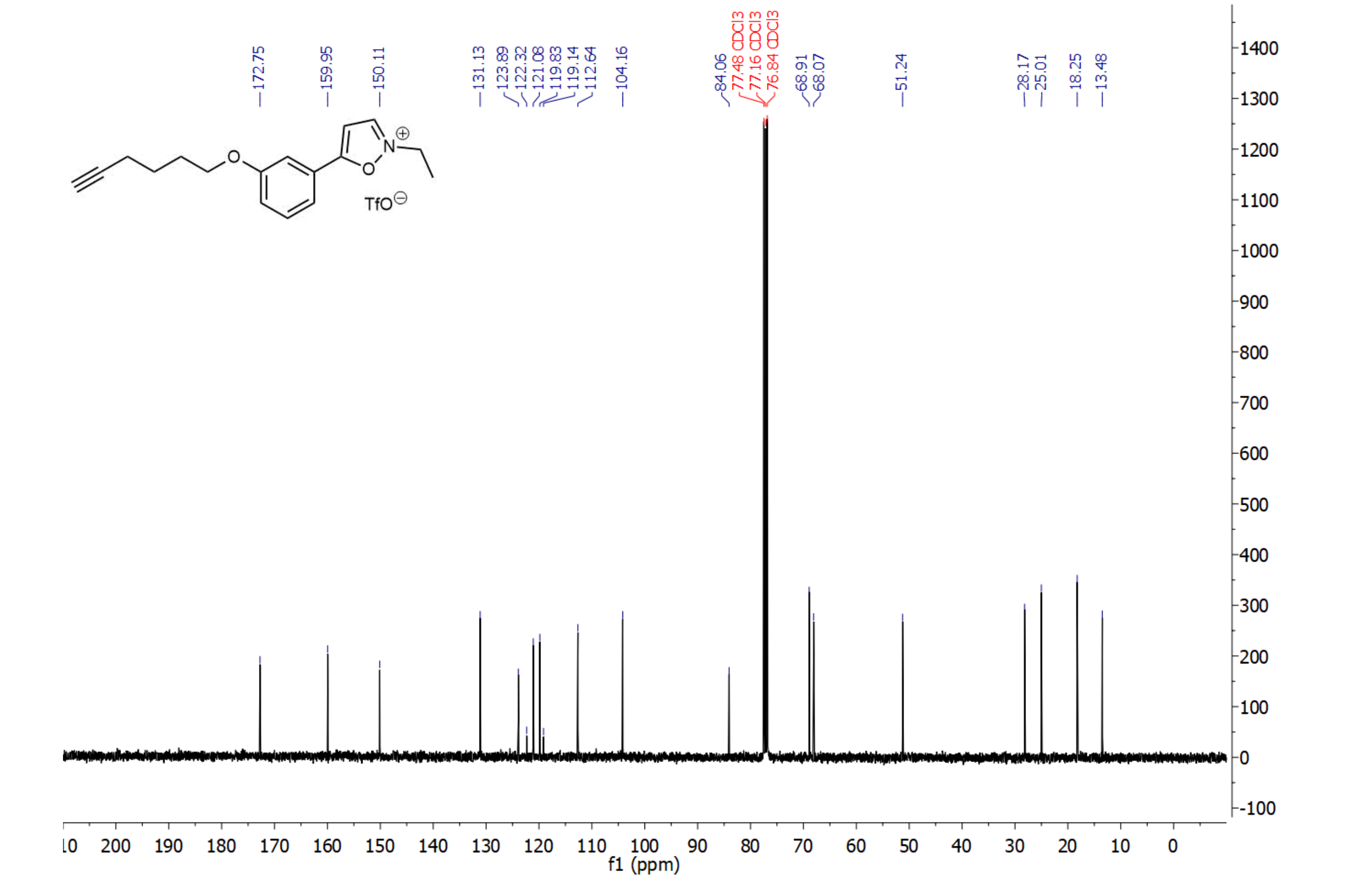

2-Ethyl-5-(3-(hex-5-yn-1-yloxy)phenyl)isoxazol-2-ium trifluoromethanesulfonate (**Isx-alkyne**) (^{19}F, 376 MHz, $CDCl_3$):

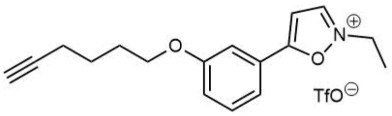

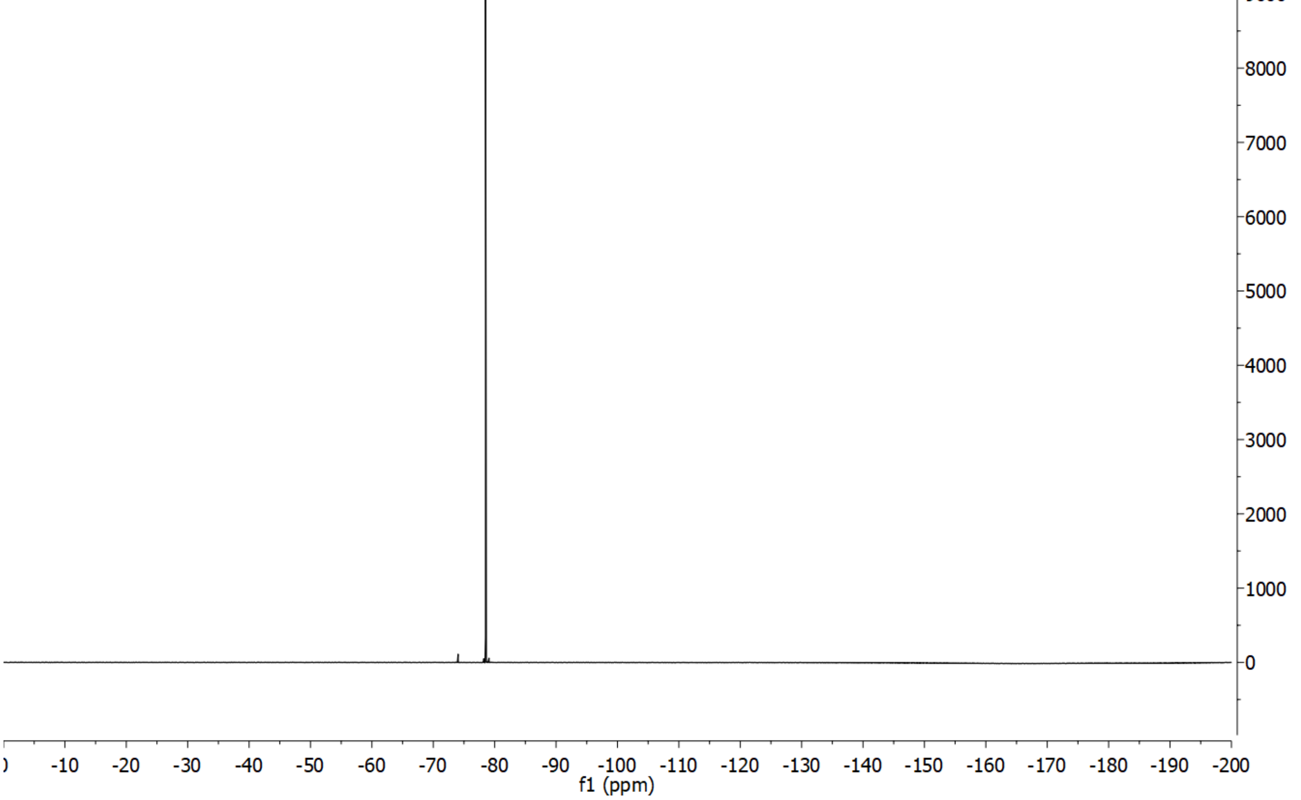

(4-(1-Azido-2-iodoethyl)phenoxy)tri*iso*propylsilane (**19**) (^{1}H, 300 MHz, CDCl$_3$):

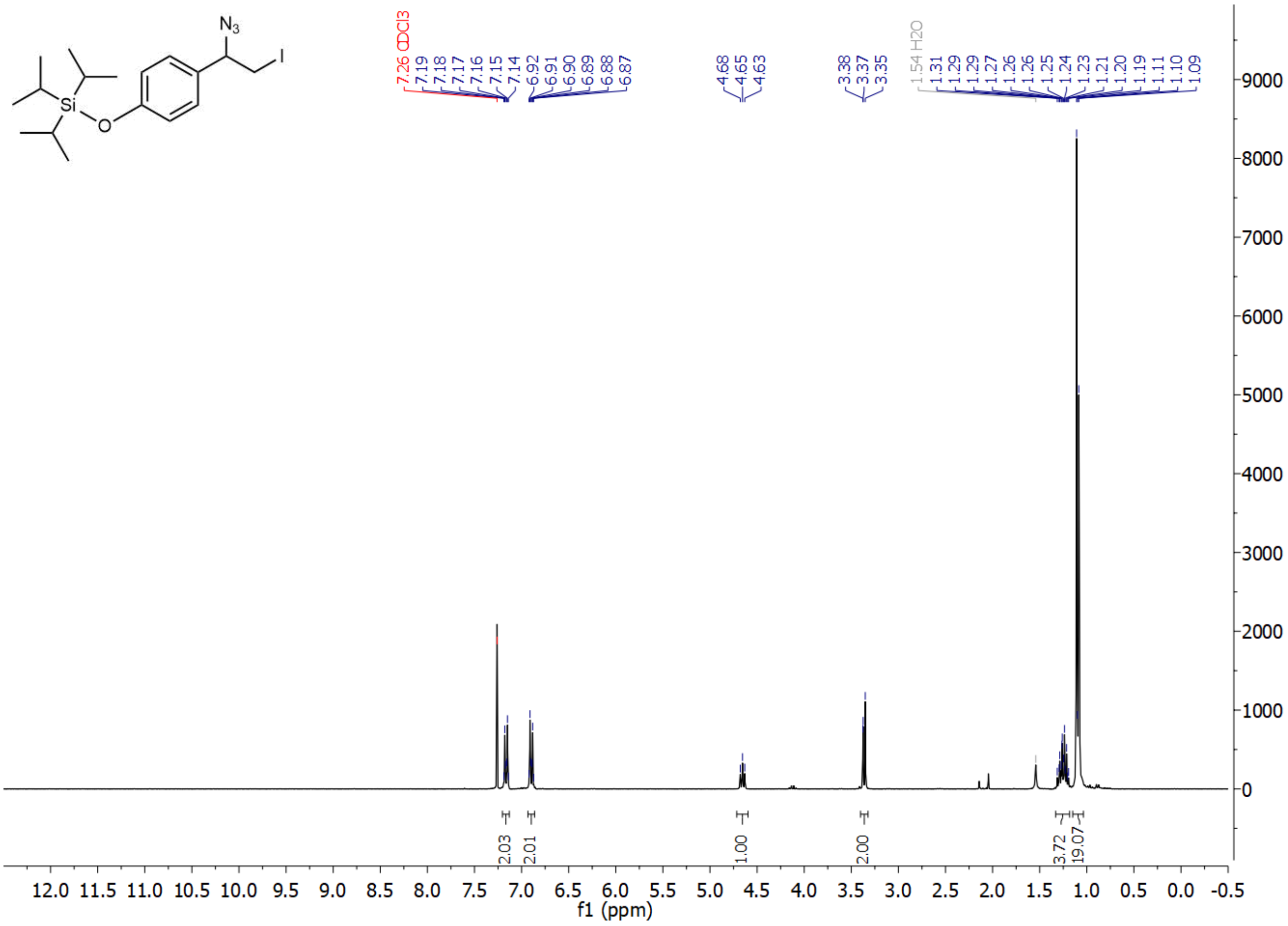

(4-(1-Azido-2-iodoethyl)phenoxy)tri*iso*propylsilane (**19**) (^{13}C, 75.5 MHz, CDCl$_3$):

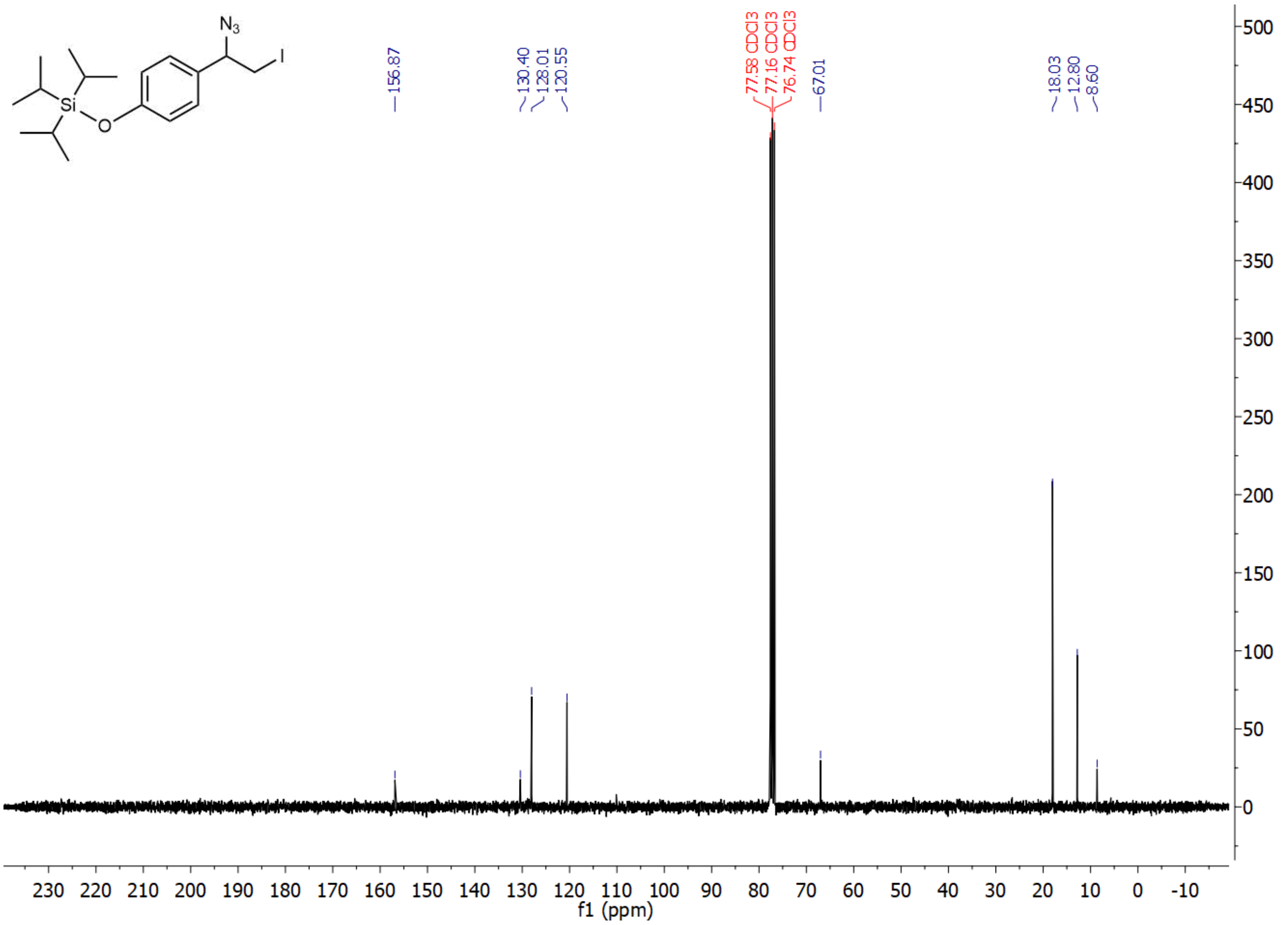

(4-(1-Azidovinyl)phenoxy)tri*iso*propylsilane (**20**) (^{1}H, 500 MHz, $CDCl_3$):

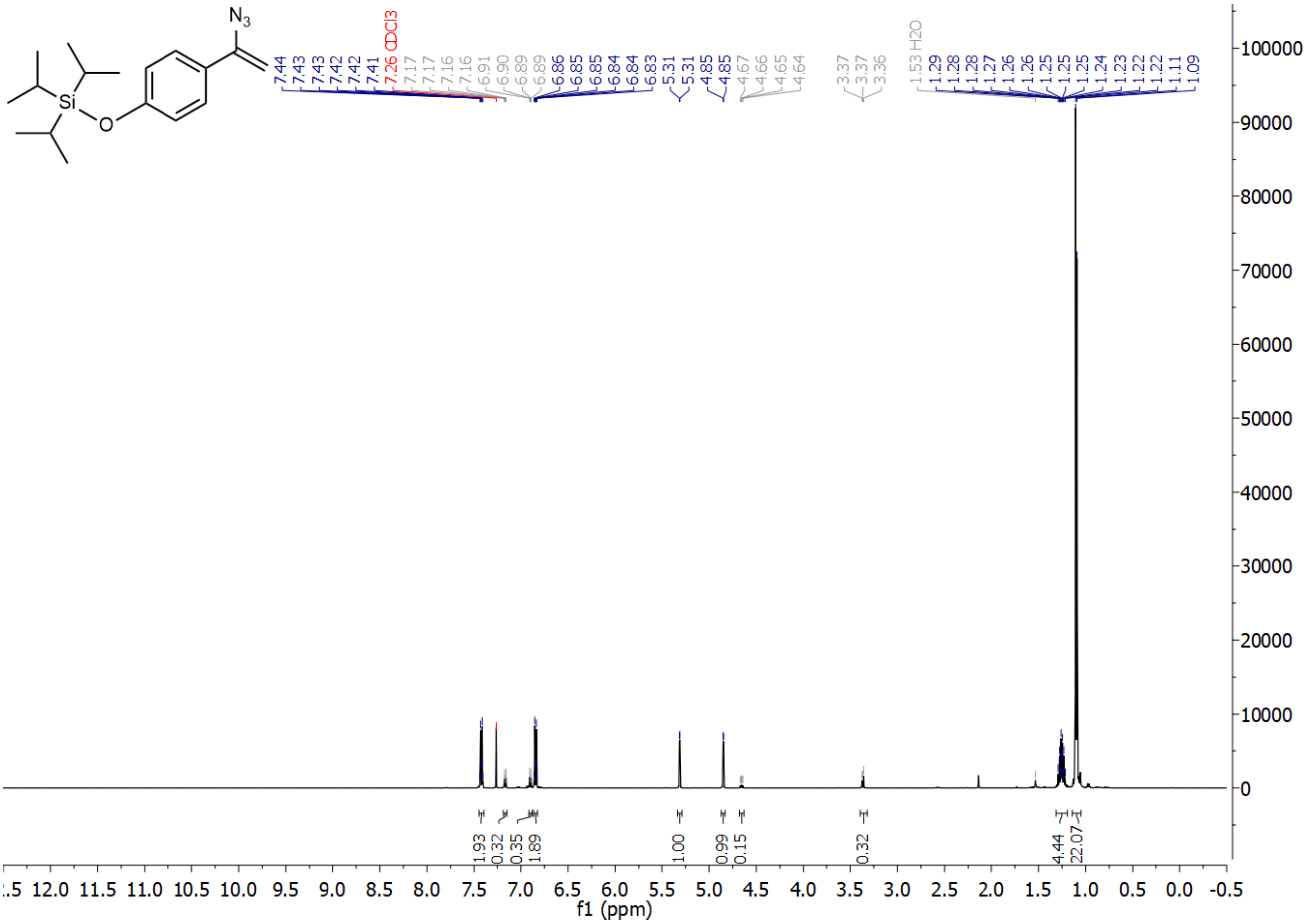

(4-(1-Azidovinyl)phenoxy)tri*iso*propylsilane (**20**) (^{13}C, 75.5 MHz, $CDCl_3$):

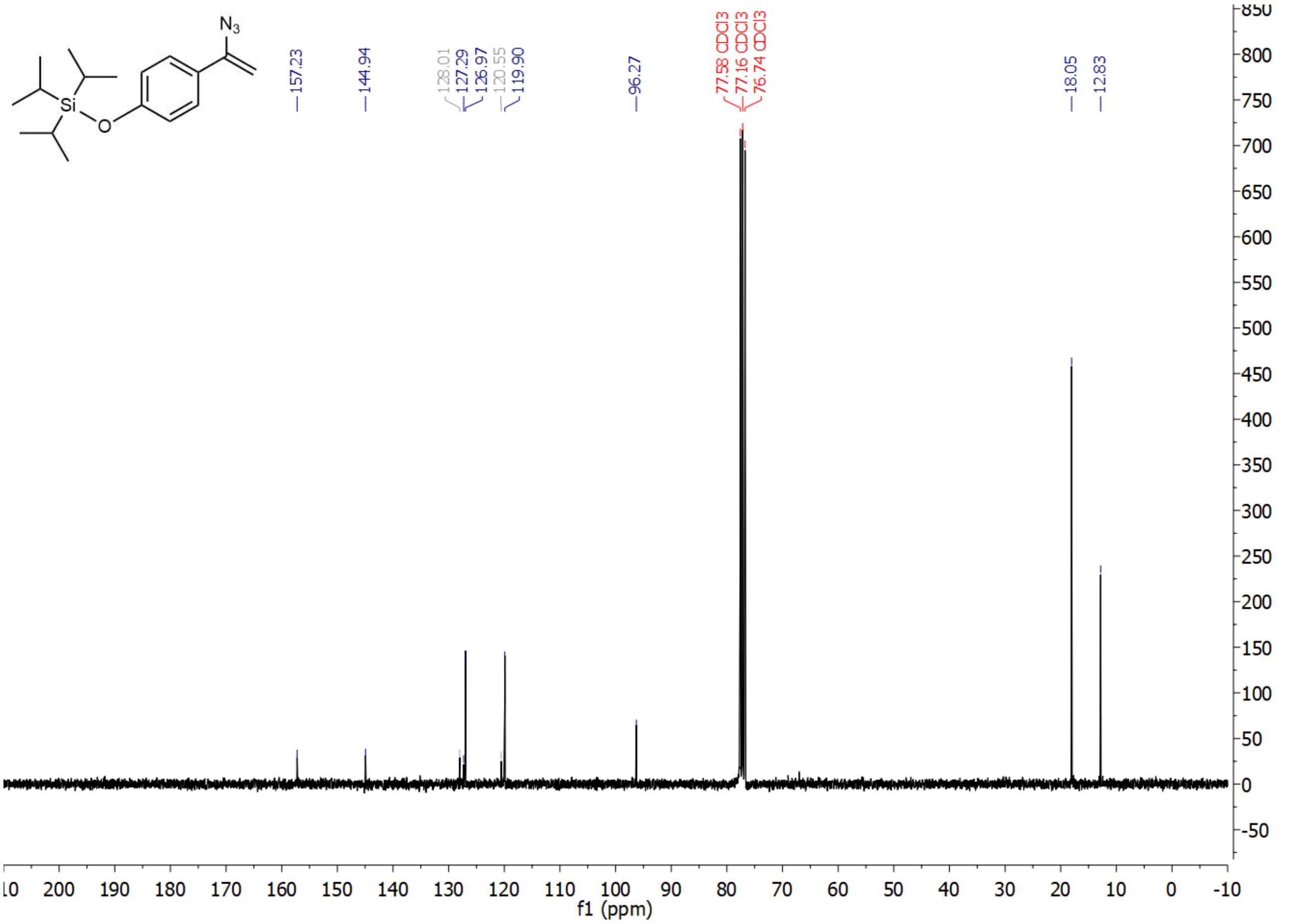

3-(4-(Prop-2-yn-1-yloxy)phenyl)-2*H*-azirine (**Az-alkyne**) (^{1}H, 500 MHz, $CDCl_3$):

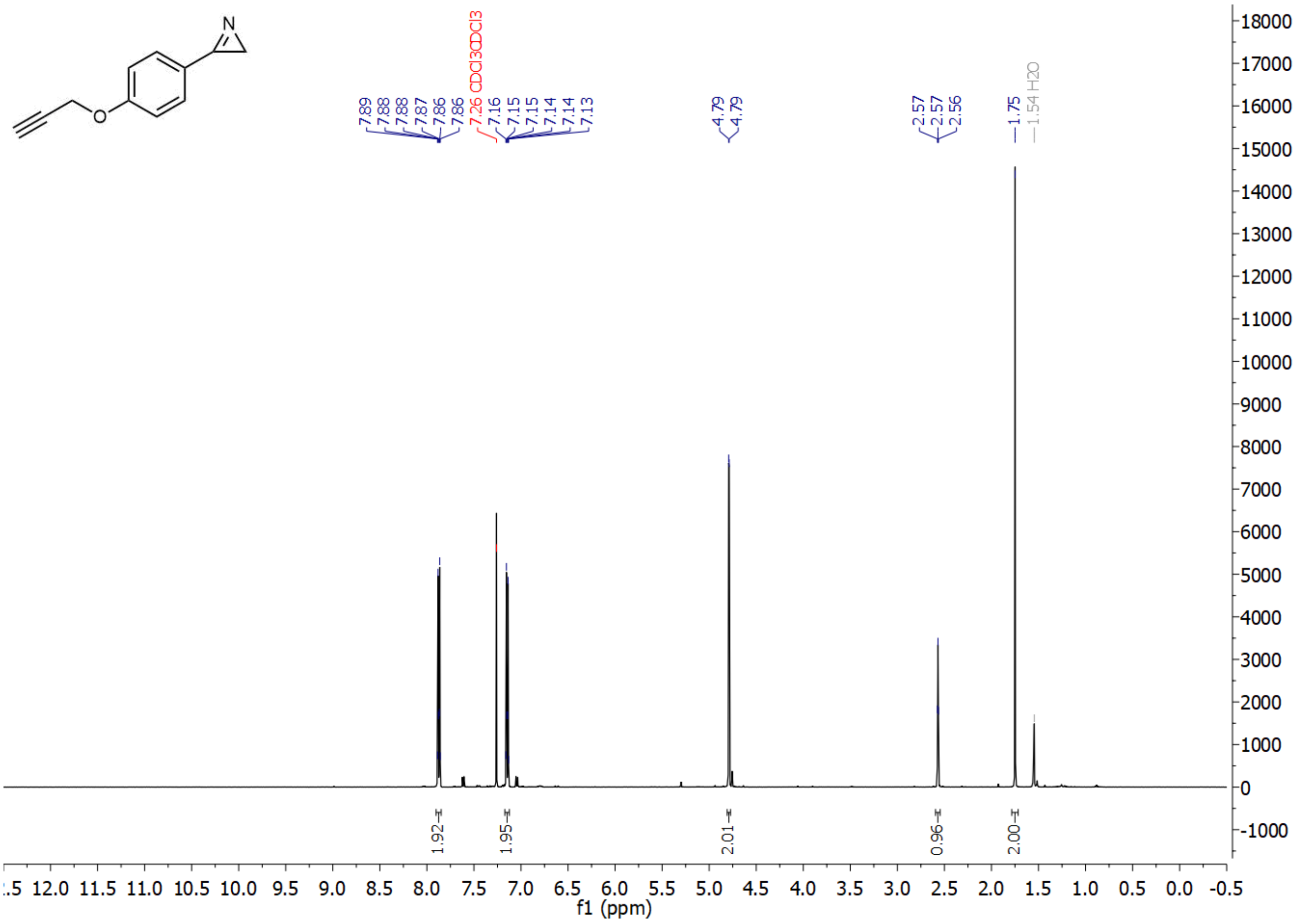

3-(4-(Prop-2-yn-1-yloxy)phenyl)-2*H*-azirine (**Az-alkyne**) (^{13}C, 101 MHz, $CDCl_3$):

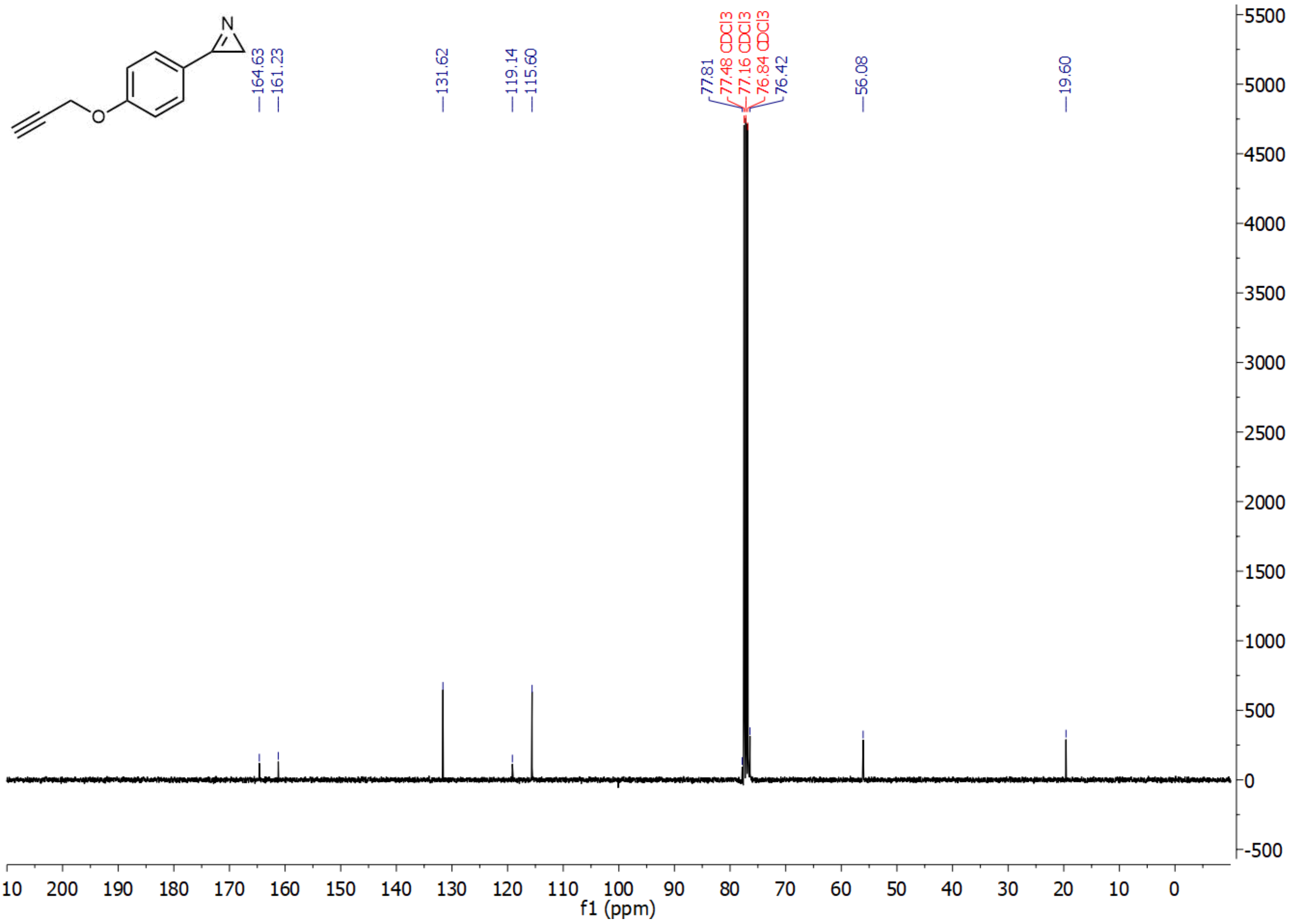

4-(2-Acetylhydrazineyl)benzoic acid (**23**) (^1H, 300 MHz, DMSO-d_6):

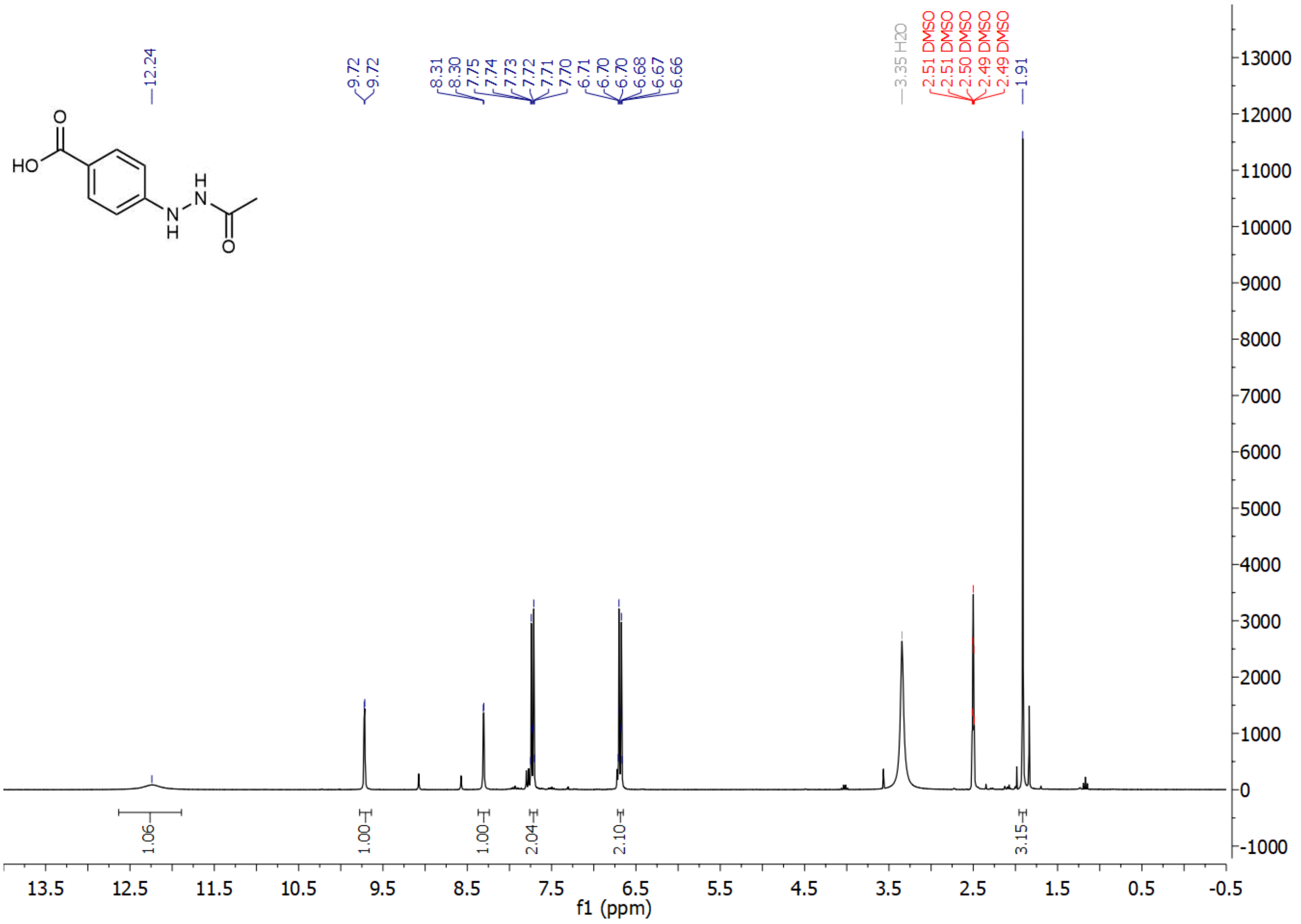

4-(2-Acetylhydrazineyl)benzoic acid (**23**) (^{13}C, 75.5 MHz, DMSO-d_6):

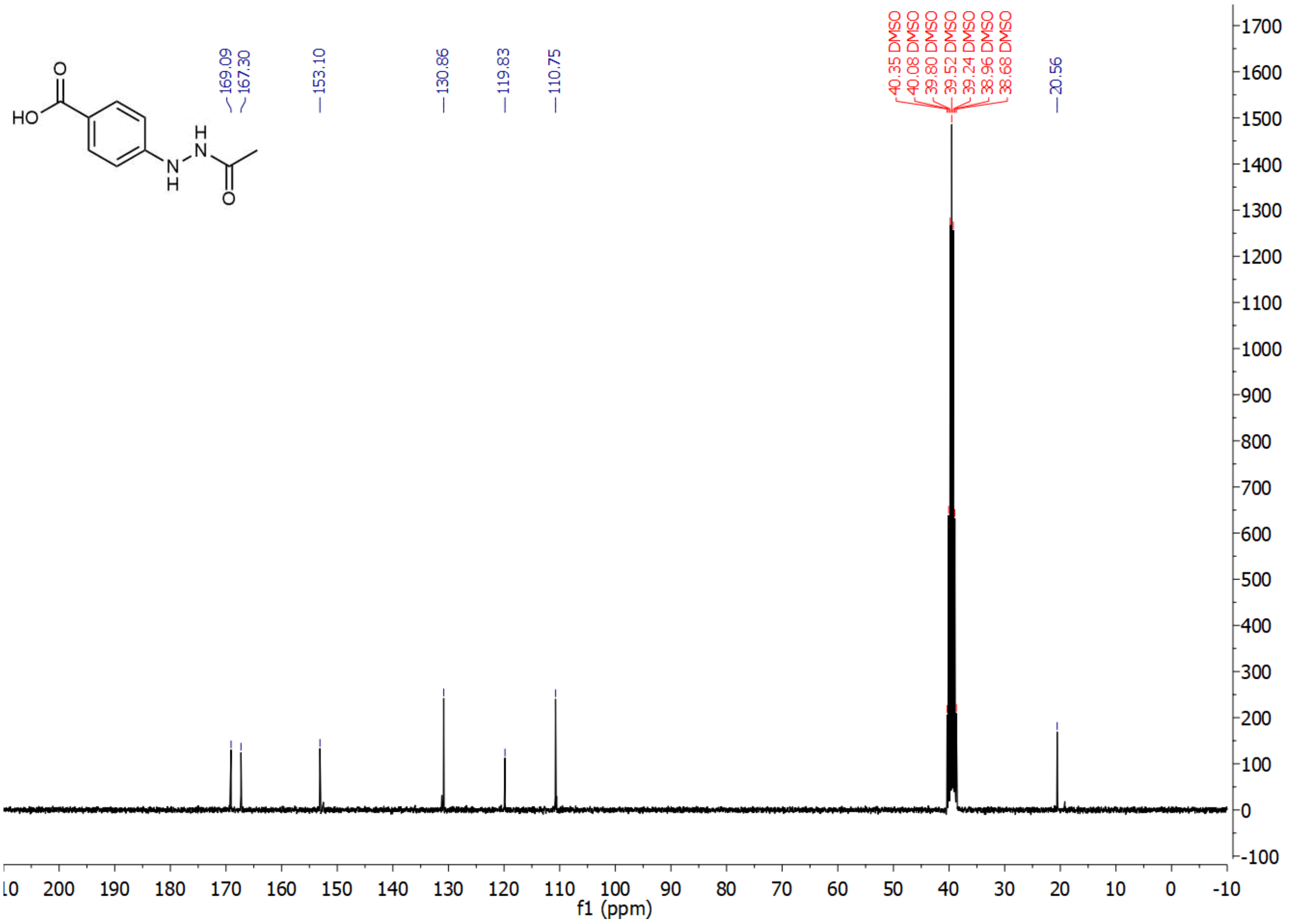

4-(2-Acetylhydrazineyl)-*N*-(prop-2-yn-1-yl)benzamide (**24**) (^{1}H, 500 MHz, DMSO-d_6):

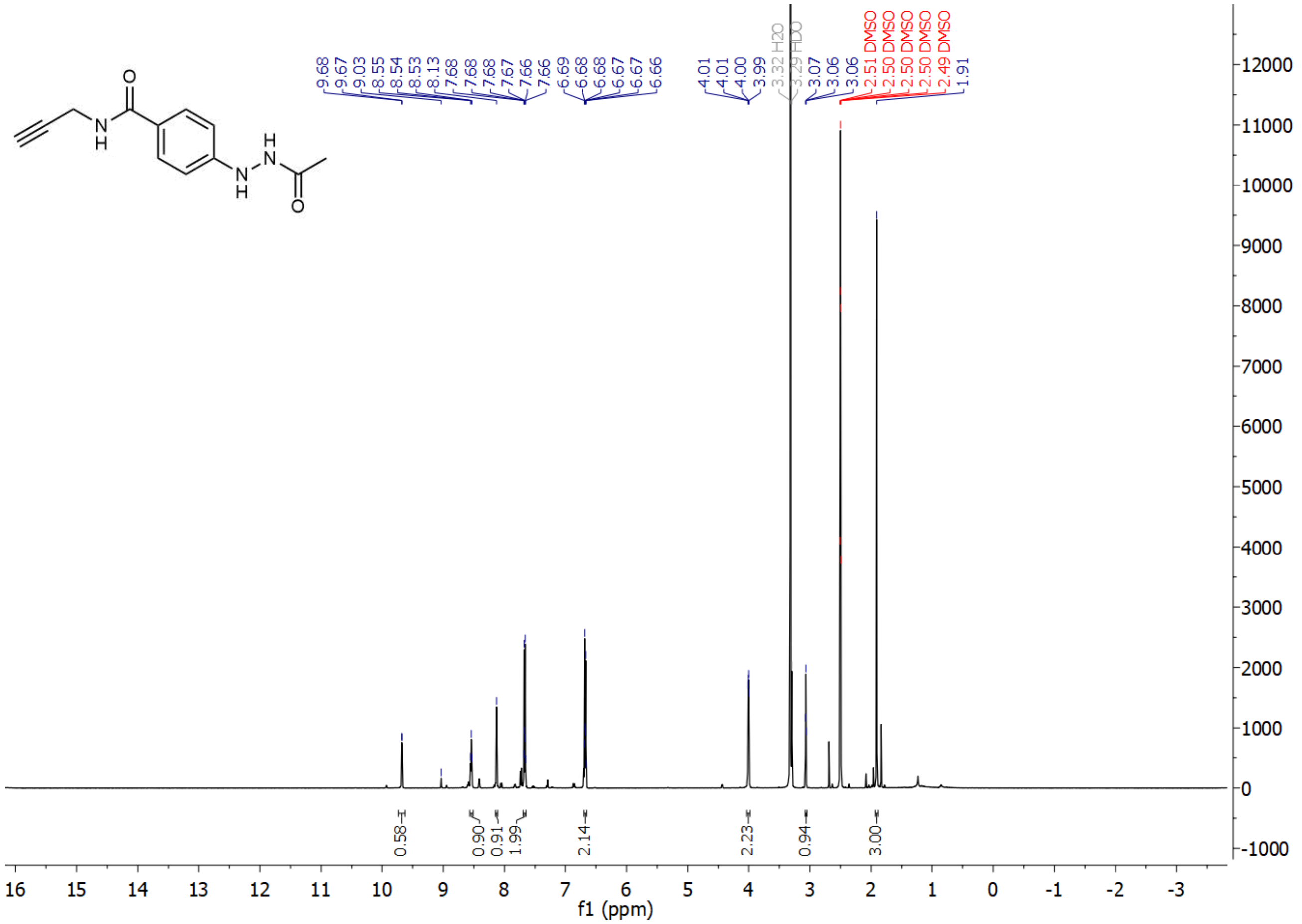

4-(2-Acetylhydrazineyl)-*N*-(prop-2-yn-1-yl)benzamide (**24**) (^{13}C, 101 MHz, DMSO-d_6):

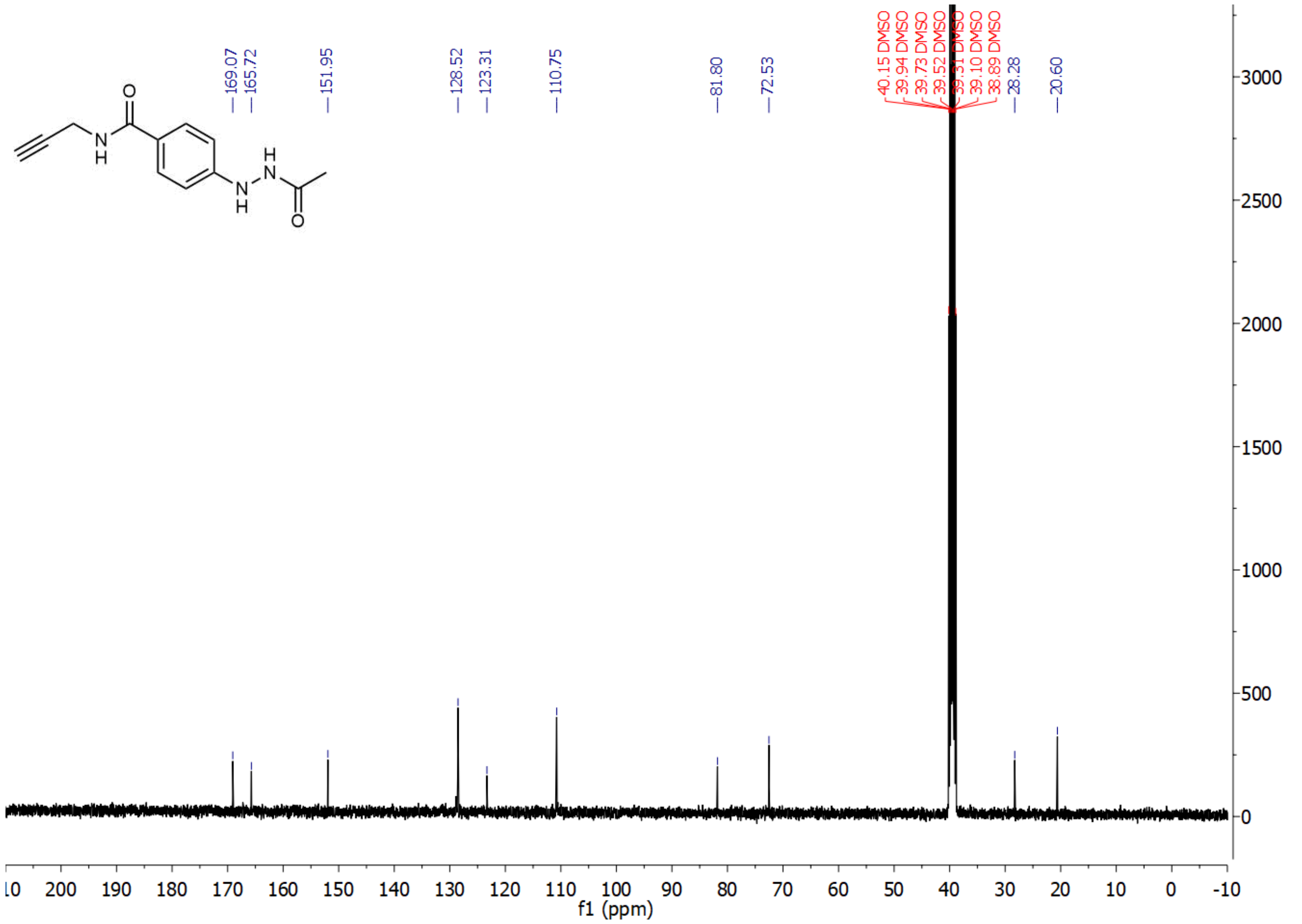

(*Z*)-*N*-(4-(Prop-2-yn-1-ylcarbamoyl)phenyl)acetohydrazonoyl chloride (**HC-alkyne**) (^{1}H, 500 MHz, $CDCl_3$):

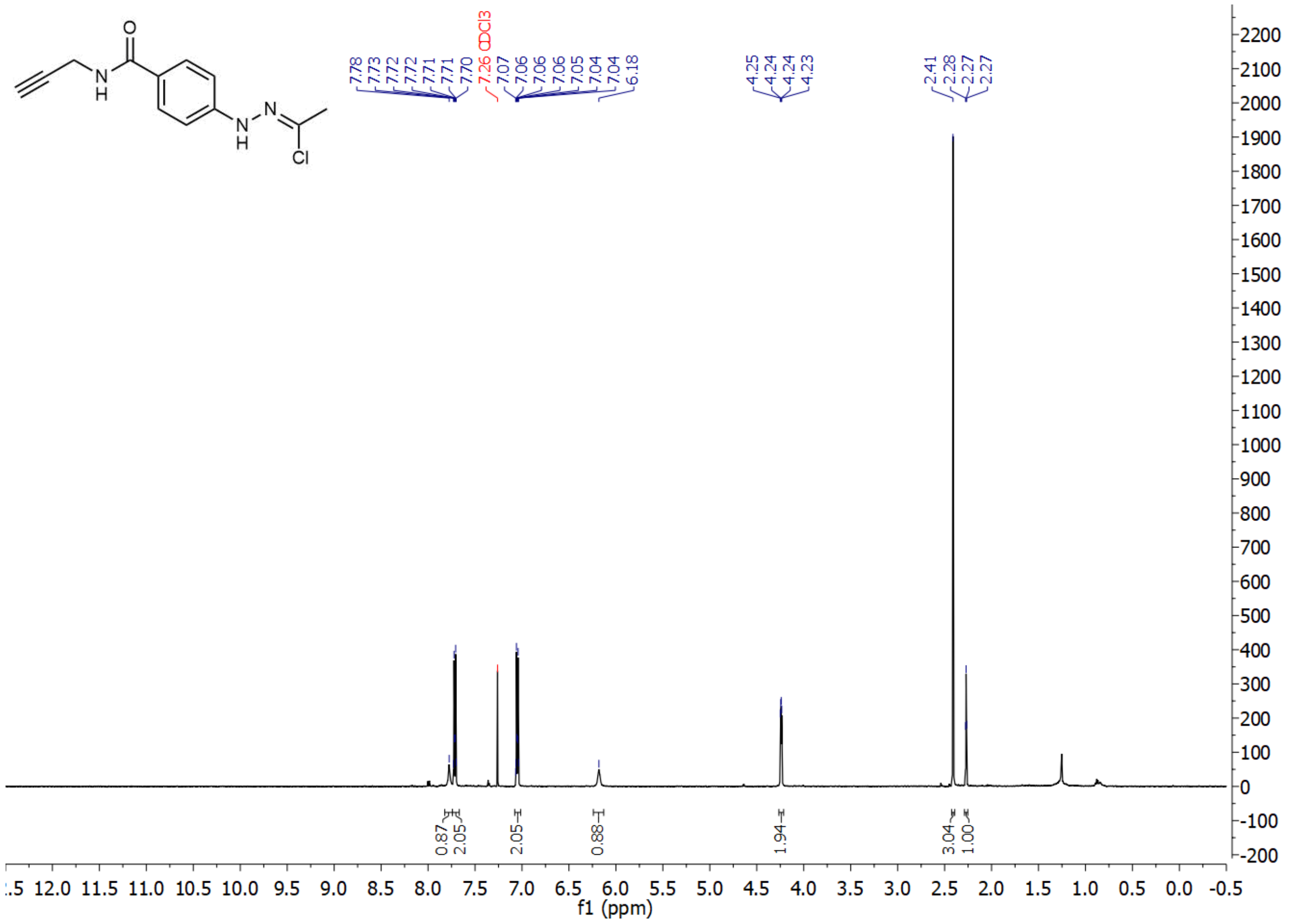

(*Z*)-*N*-(4-(Prop-2-yn-1-ylcarbamoyl)phenyl)acetohydrazonoyl chloride (**HC-alkyne**) (^{13}C, 75.5 MHz, $CDCl_3$):

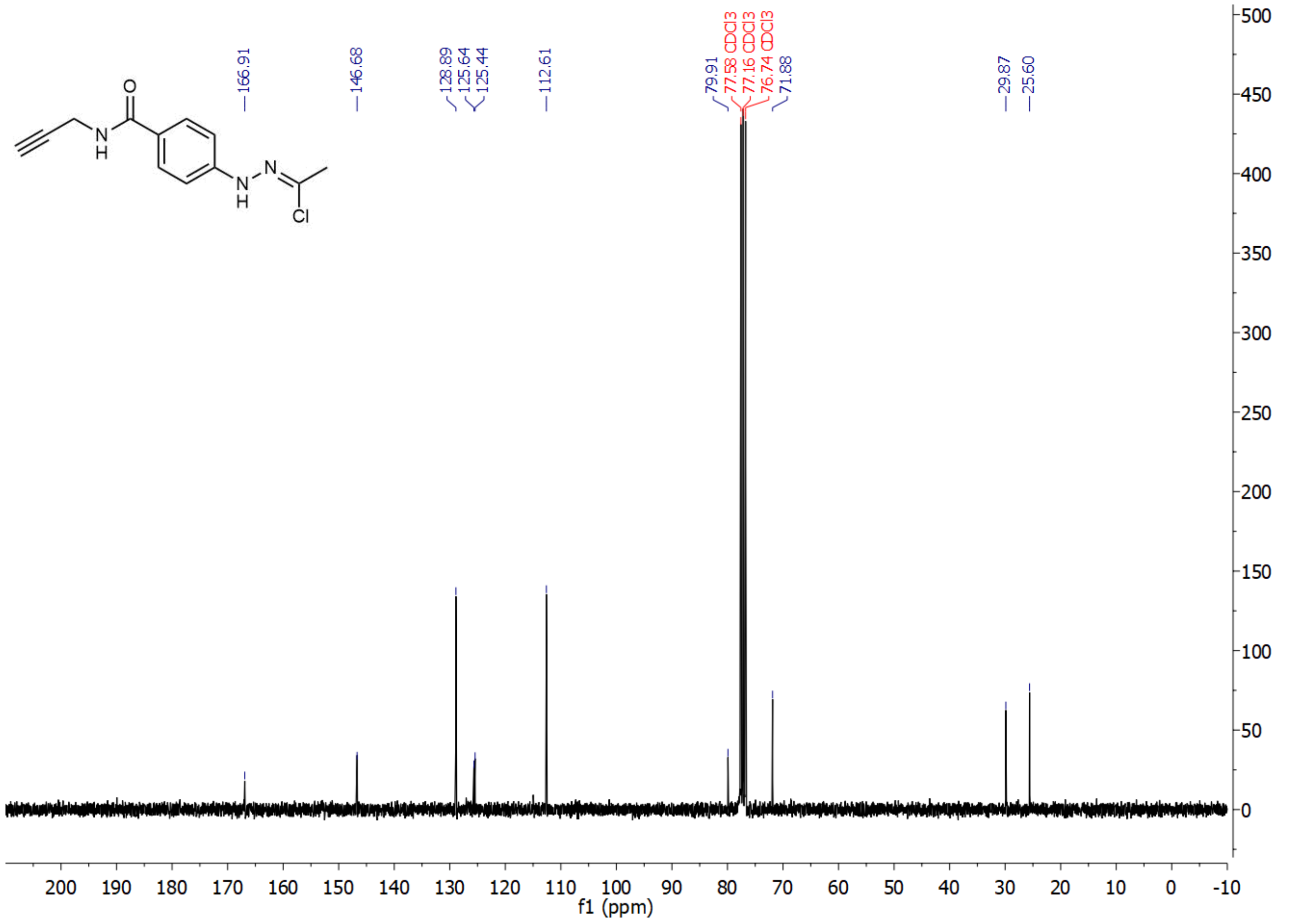

4-(Hex-5-yn-1-ylcarbamoyl)benzenesulfonyl fluoride (**SuFEx-alkyne**) (^1H, 300 MHz, $CDCl_3$):

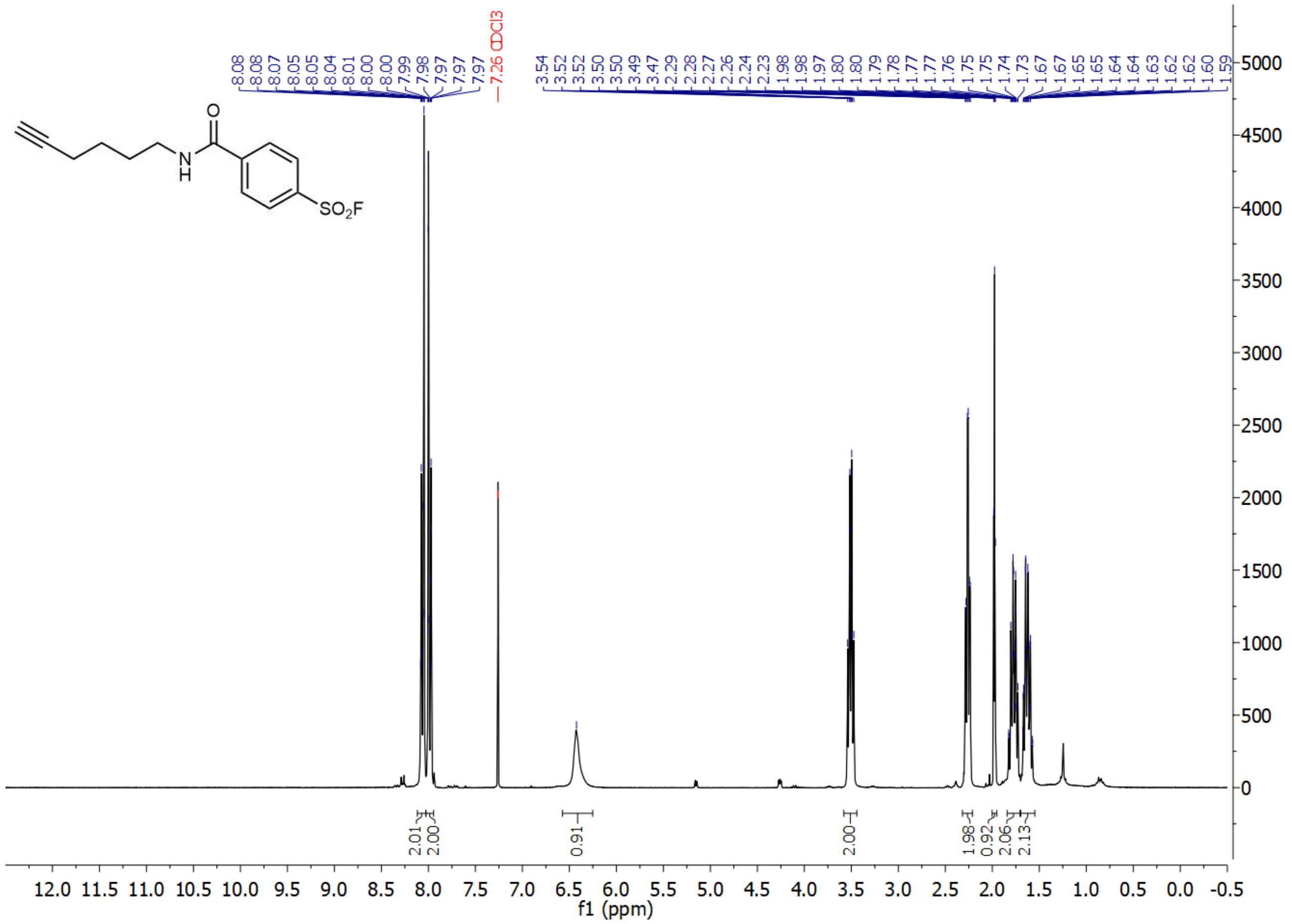

4-(Hex-5-yn-1-ylcarbamoyl)benzenesulfonyl fluoride (**SuFEx-alkyne**) (^{13}C, 75.5 MHz, $CDCl_3$):

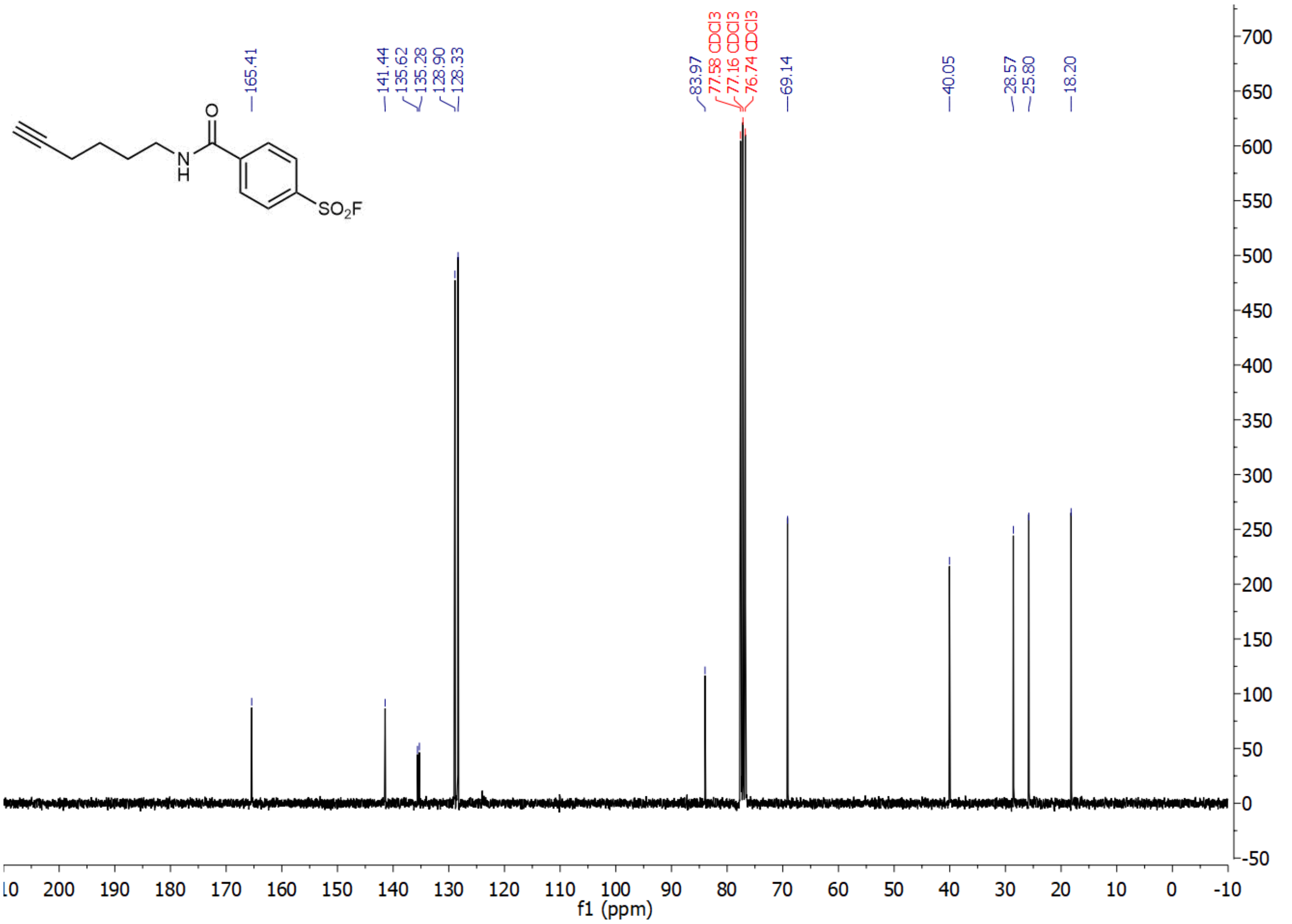

4-(Hex-5-yn-1-ylcarbamoyl)benzenesulfonyl fluoride (**SuFEx-alkyne**) (^{19}F, 377 MHz, $CDCl_3$):

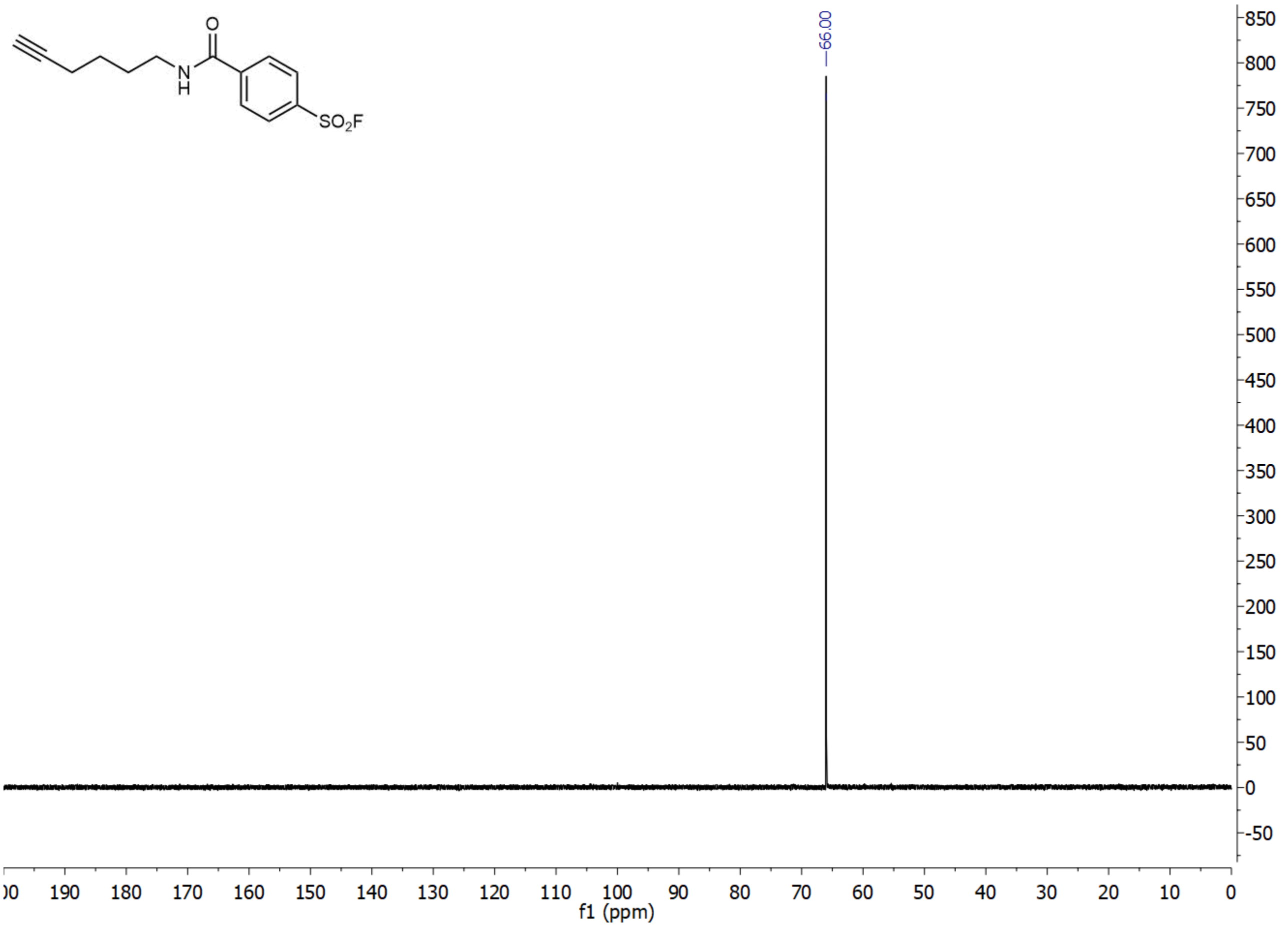

4-(4-(Prop-2-yn-1-yloxy)phenyl)-3*H*-1,2,4-triazole-3,5(4*H*)-dione (**PTAD-alkyne**) (^{1}H, 500 MHz, CD_3CN):

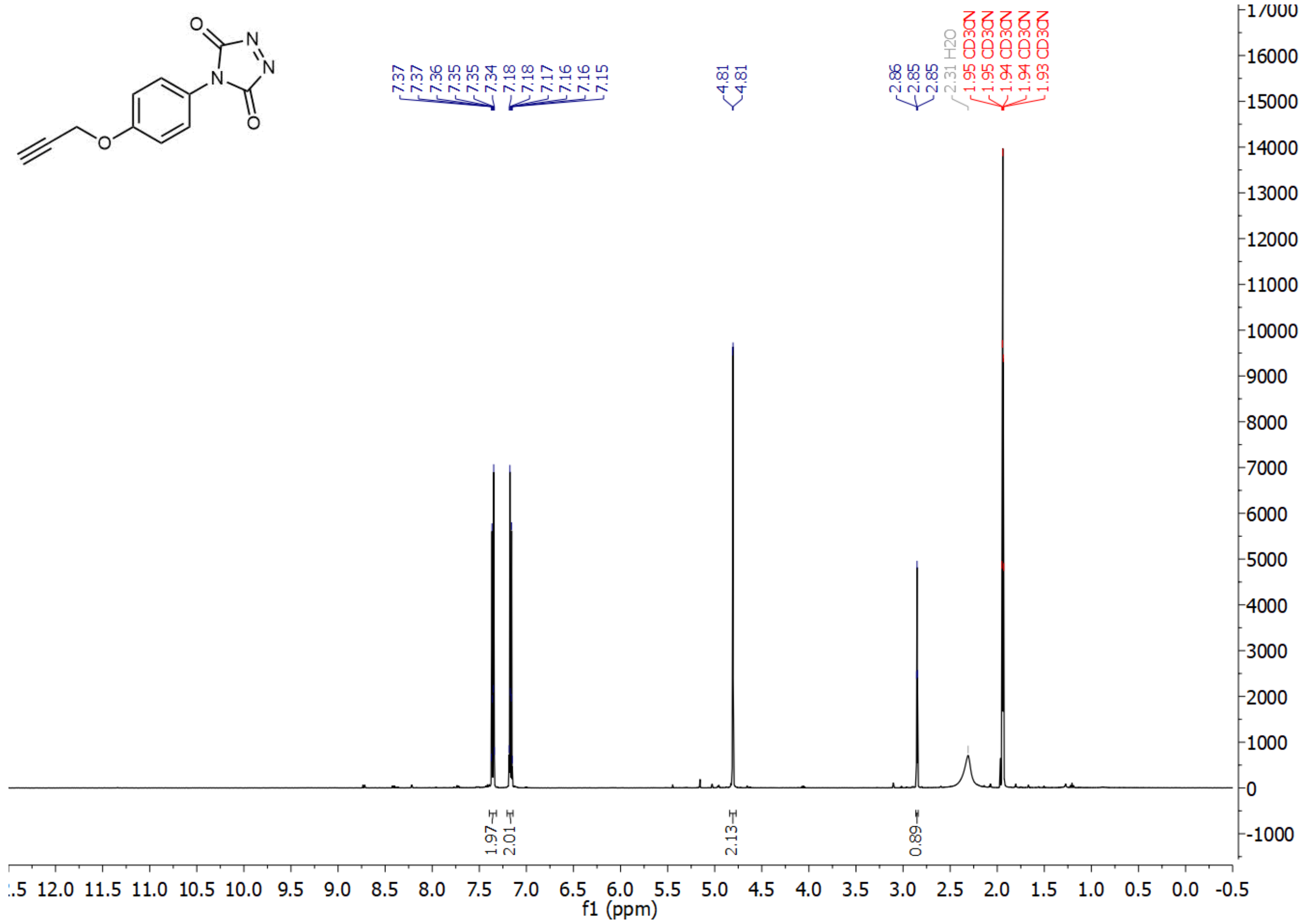

4-(4-(Prop-2-yn-1-yloxy)phenyl)-3*H*-1,2,4-triazole-3,5(4*H*)-dione (**PTAD-alkyne**) (^{13}C, 101 MHz, CD_3CN):

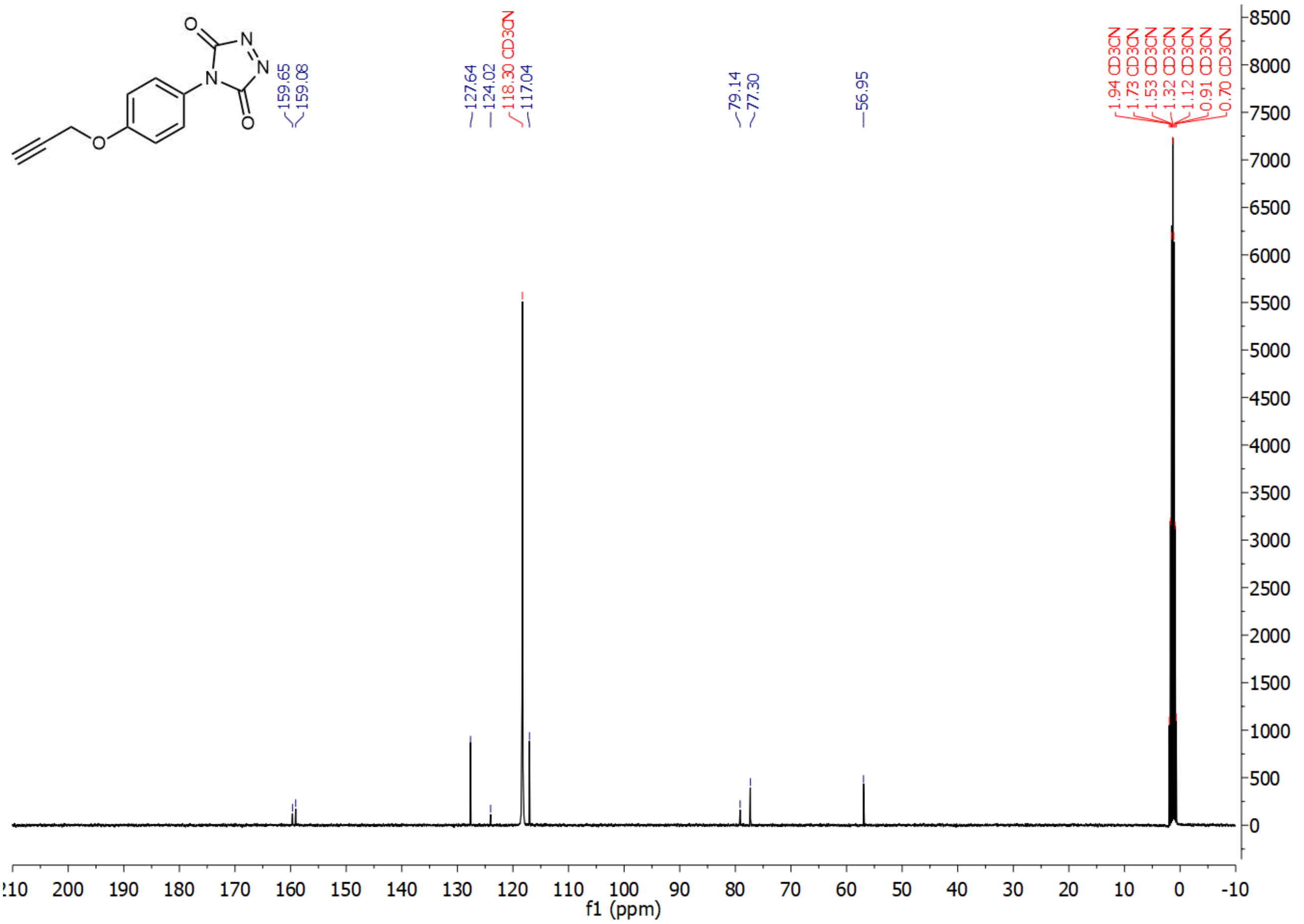

4-Ethynylbenzenediazonium tetrafluoroborate (**DA1-alkyne**) (^1H, 300 MHz, CD_3CN):

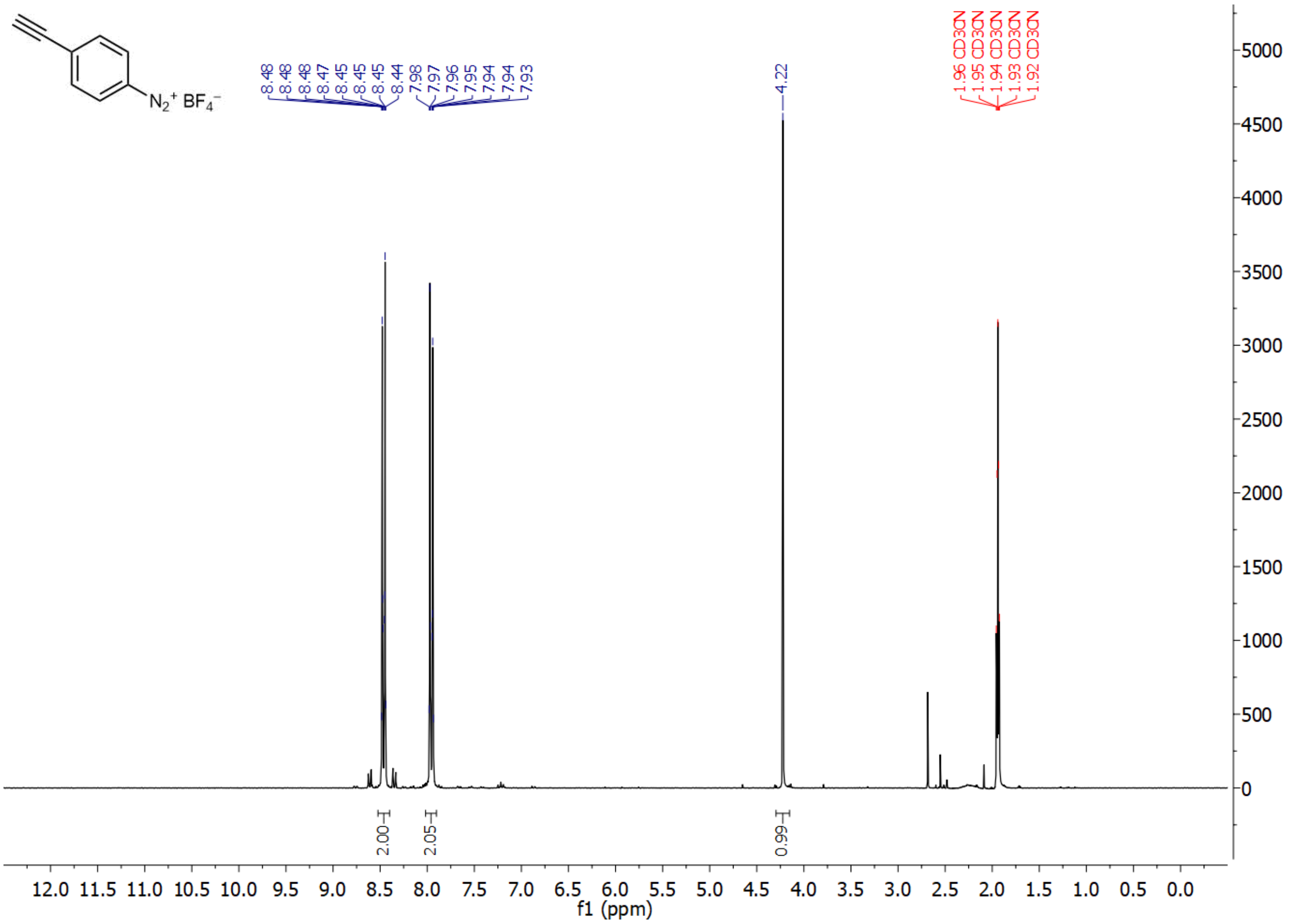

4-Ethynylbenzenediazonium tetrafluoroborate (**DA1-alkyne**) (^{13}C, 75.5 MHz, CD_3CN):

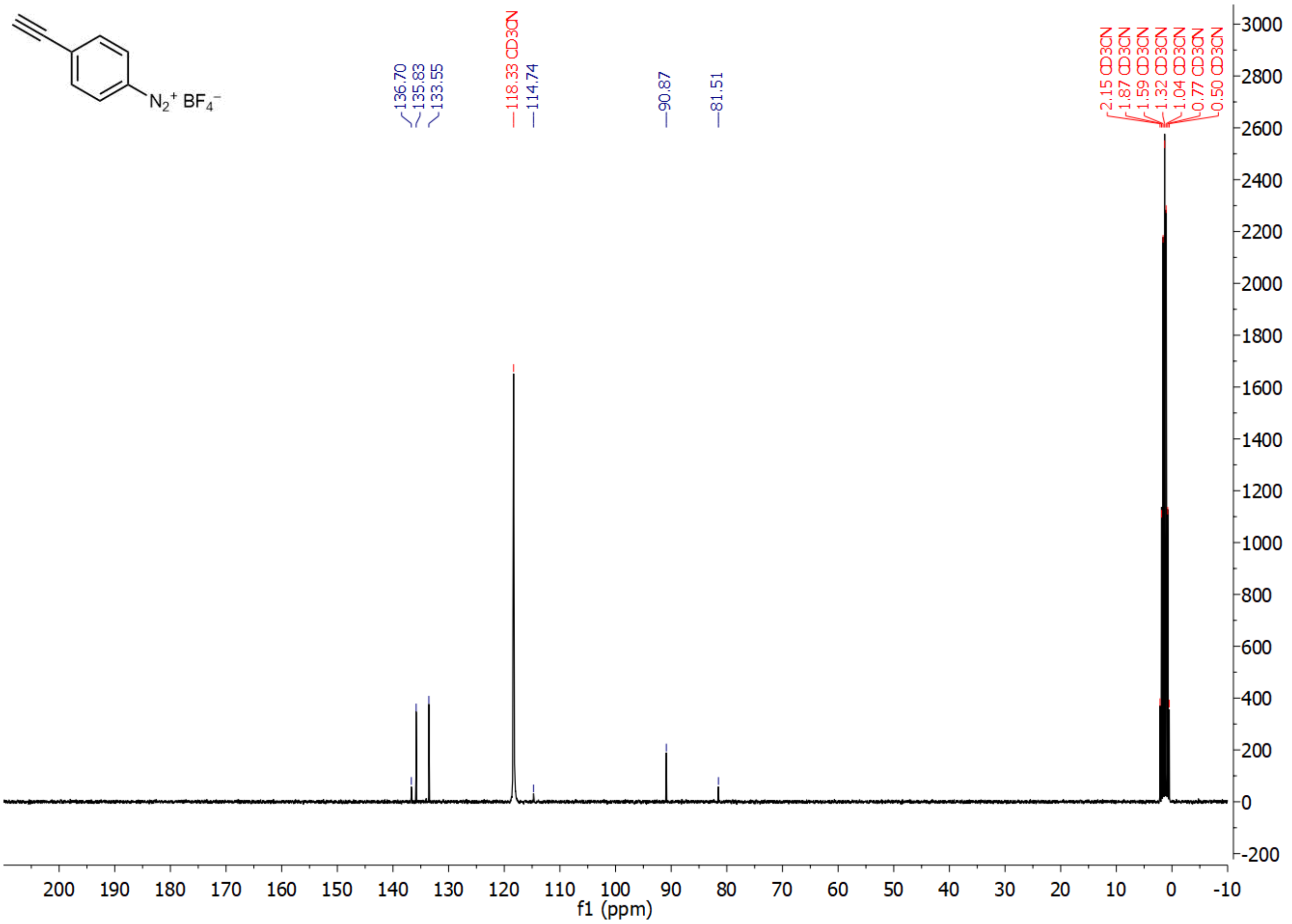

4-Ethynylbenzenediazonium tetrafluoroborate (**DA1-alkyne**) (^{19}F, 376 MHz, CD_3CN):

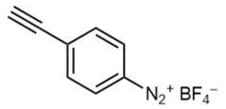

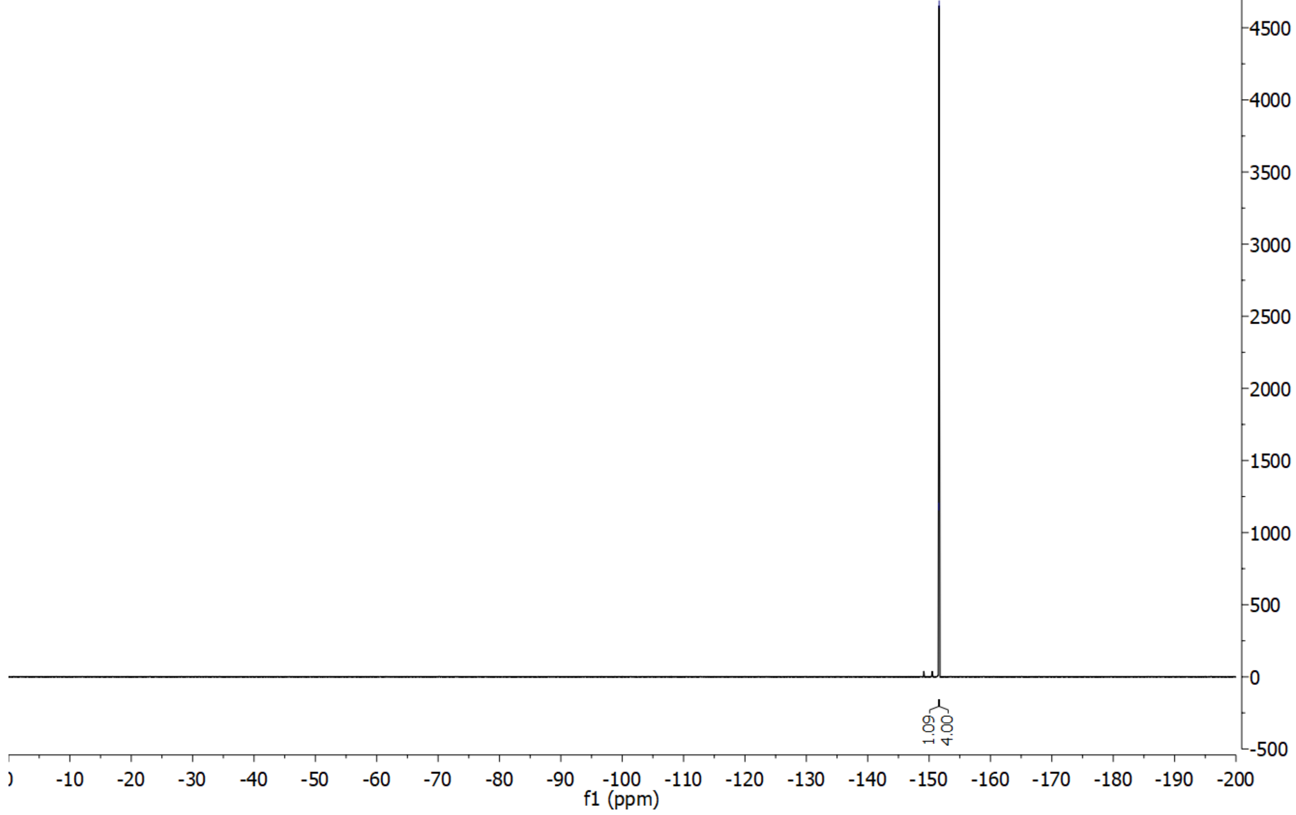

4-(Prop-2-yn-1-ylcarbamoyl)benzenediazonium tetrafluoroborate (**DA2-alkyne**) (^{1}H, 300 MHz, CD_3CN):

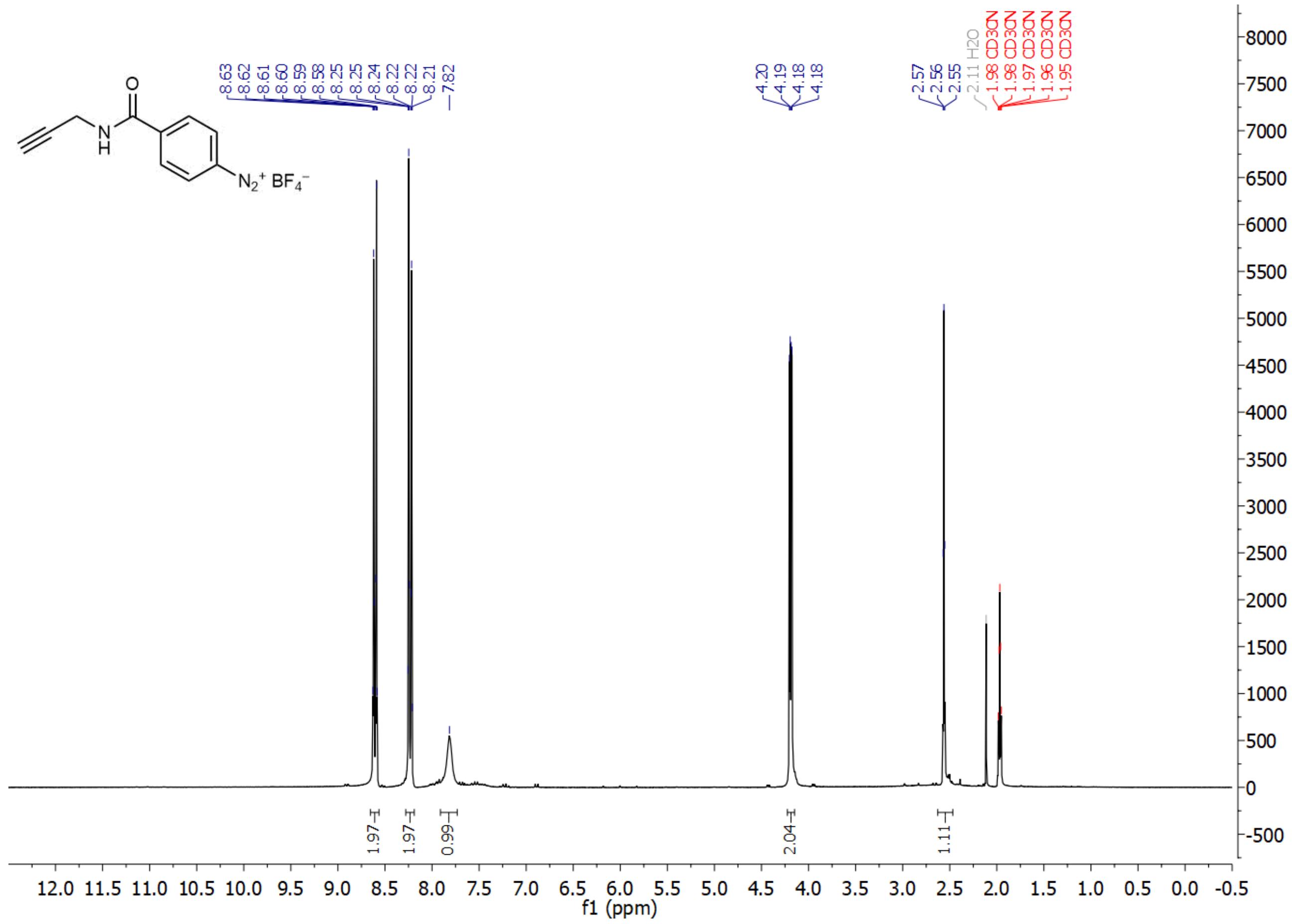

4-(Prop-2-yn-1-ylcarbamoyl)benzenediazonium tetrafluoroborate (**DA2-alkyne**) (^{13}C, 75.5 MHz, CD_3CN):

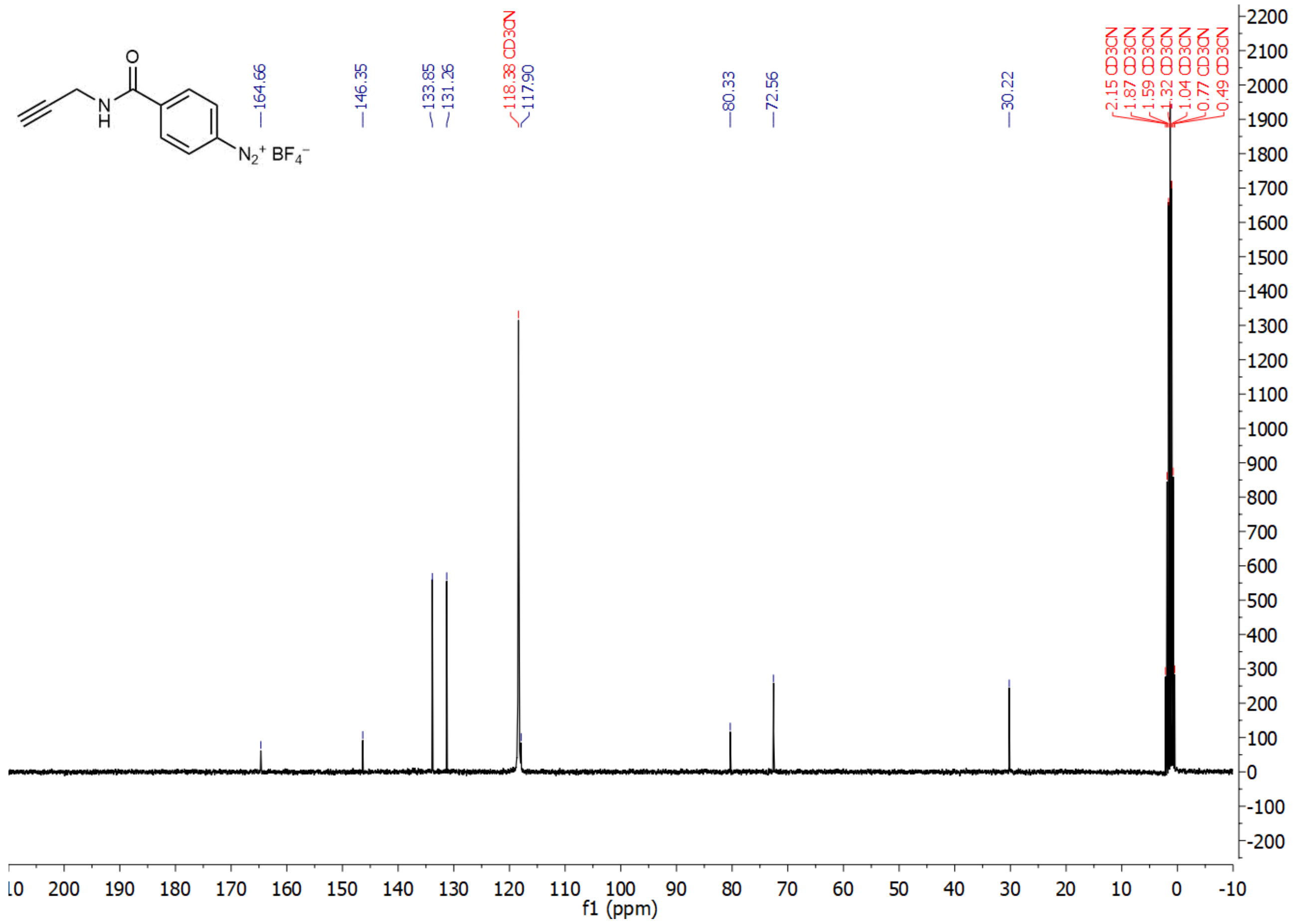

4-(Prop-2-yn-1-ylcarbamoyl)benzenediazonium tetrafluoroborate (**DA2-alkyne**) (^{19}F, 376 MHz, DMSO-d_6):

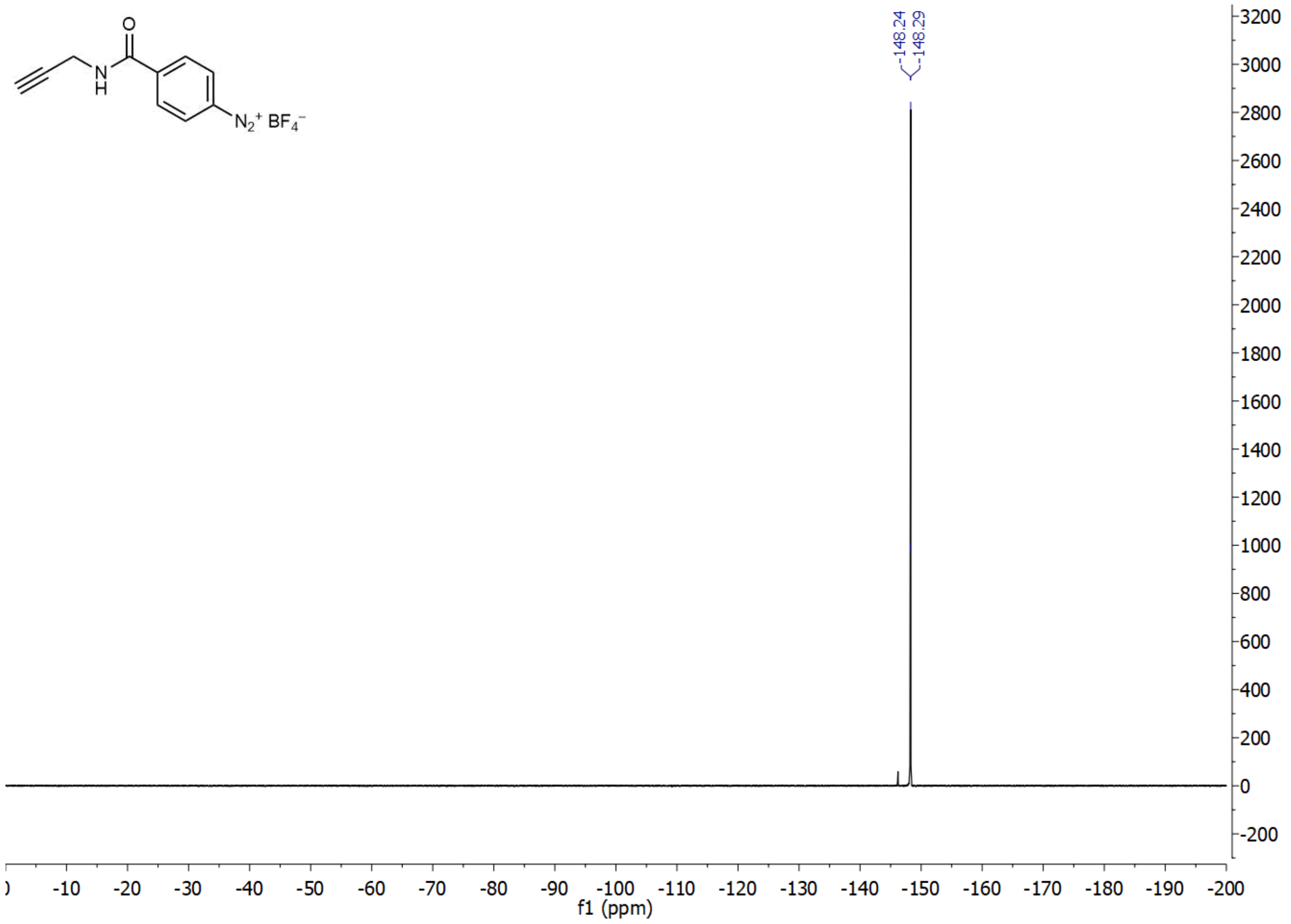

4-(Prop-2-yn-1-yloxy)benzenediazonium tetrafluoroborate (**DA3-alkyne**) (^{1}H, 300 MHz, CD_3CN):

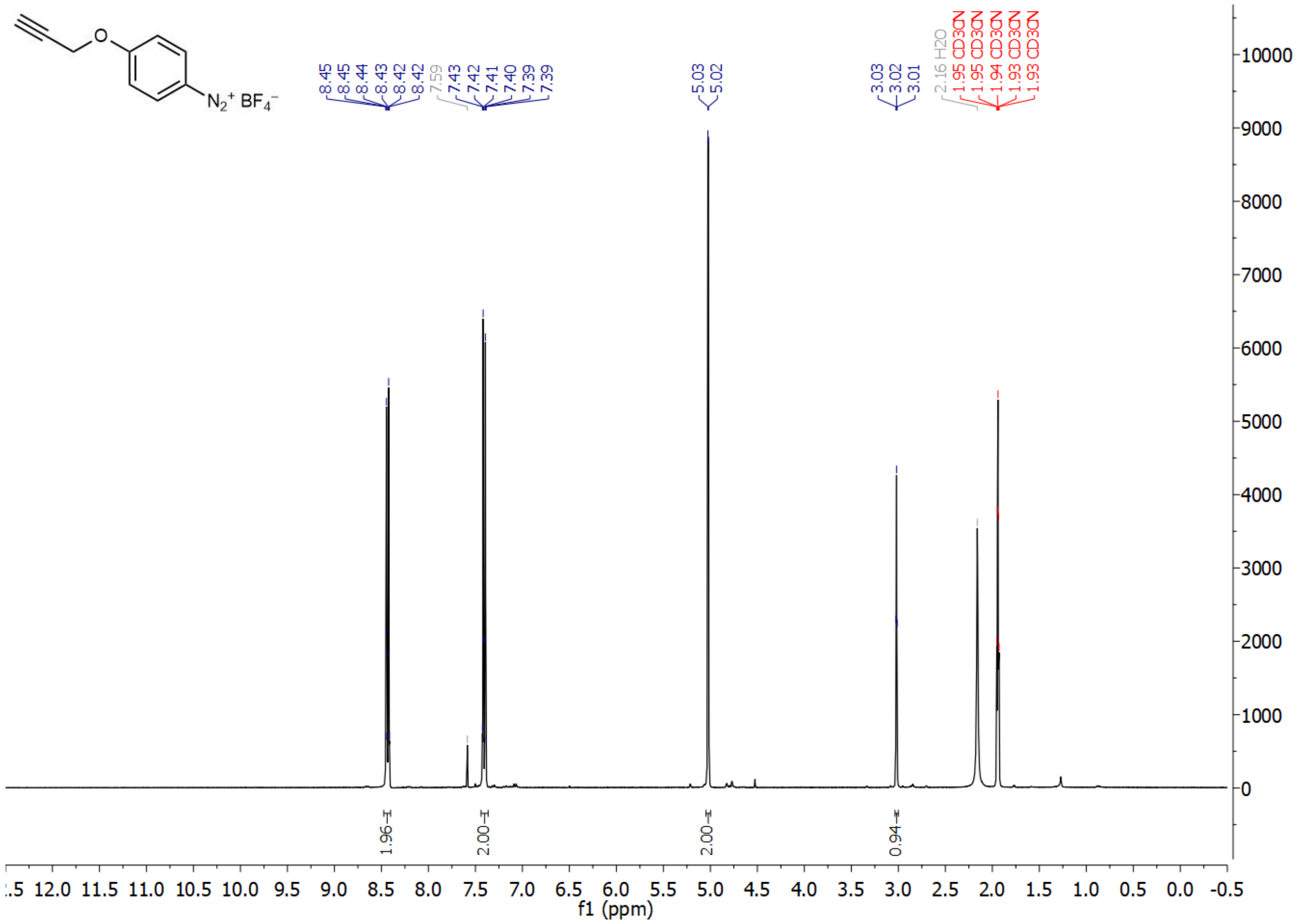

4-(Prop-2-yn-1-yloxy)benzenediazonium tetrafluoroborate (**DA3-alkyne**) (^{13}C, 75.5 MHz, CD_3CN):

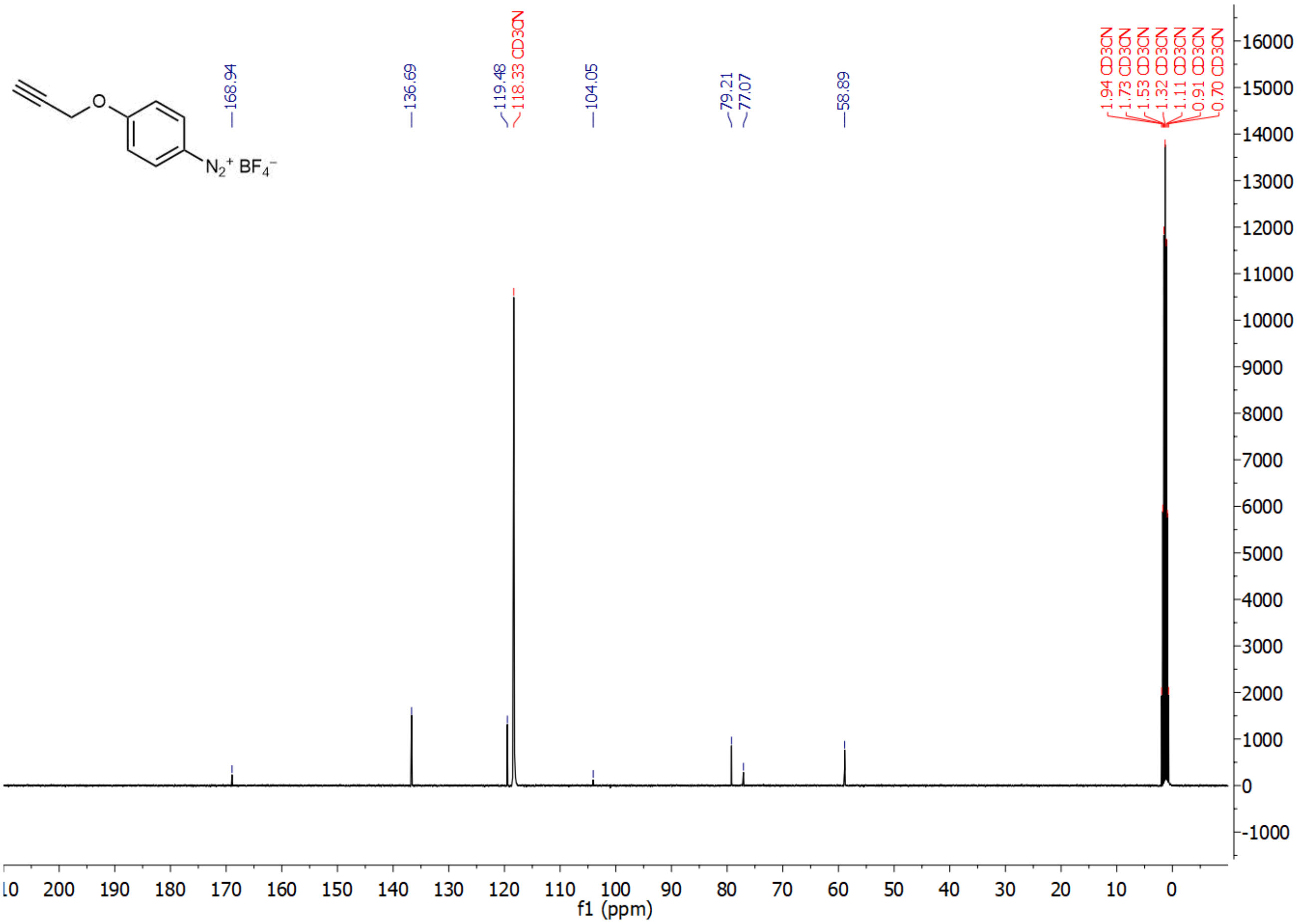

4-(Prop-2-yn-1-yloxy)benzenediazonium tetrafluoroborate (**DA3-alkyne**) (^{19}F, 376 MHz, CD_3CN):

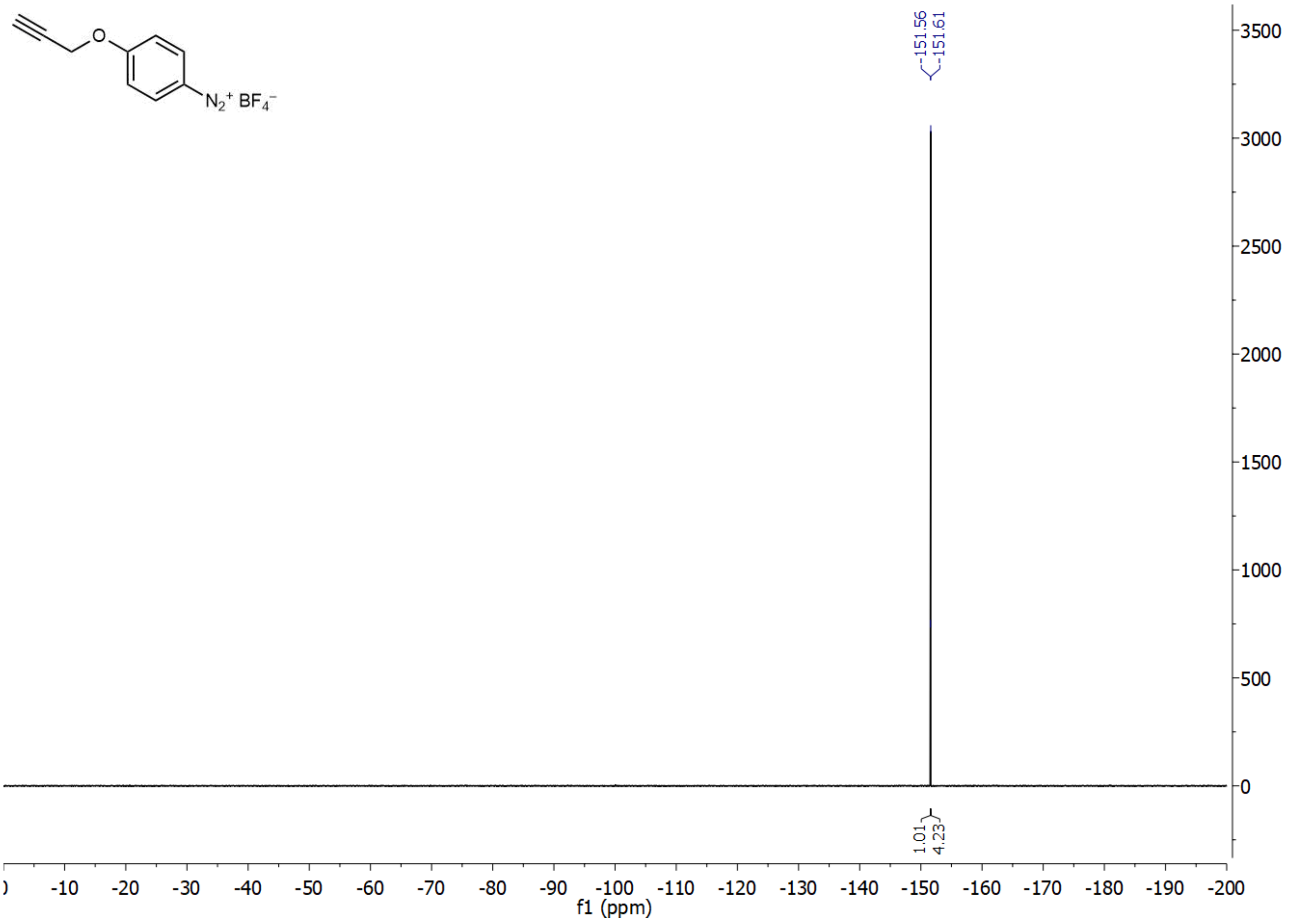

4-(Prop-2-yn-1-yloxy)piperidine hydrochloride (**32**) (^{1}H, 300 MHz, CD_3OD):

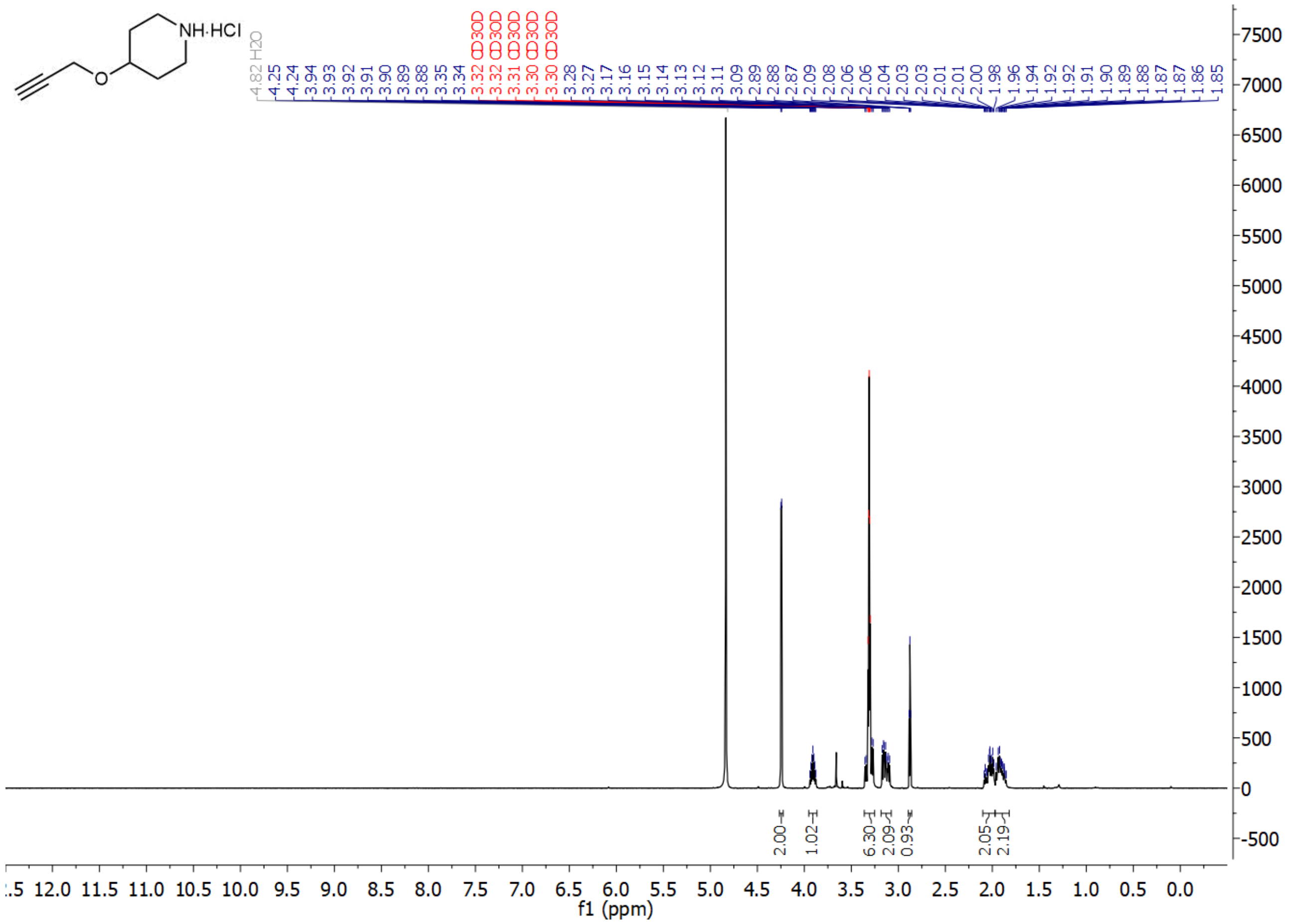

4-(Prop-2-yn-1-yloxy)piperidine hydrochloride (**32**) (^{13}C, 75.5 MHz, CD_3OD):

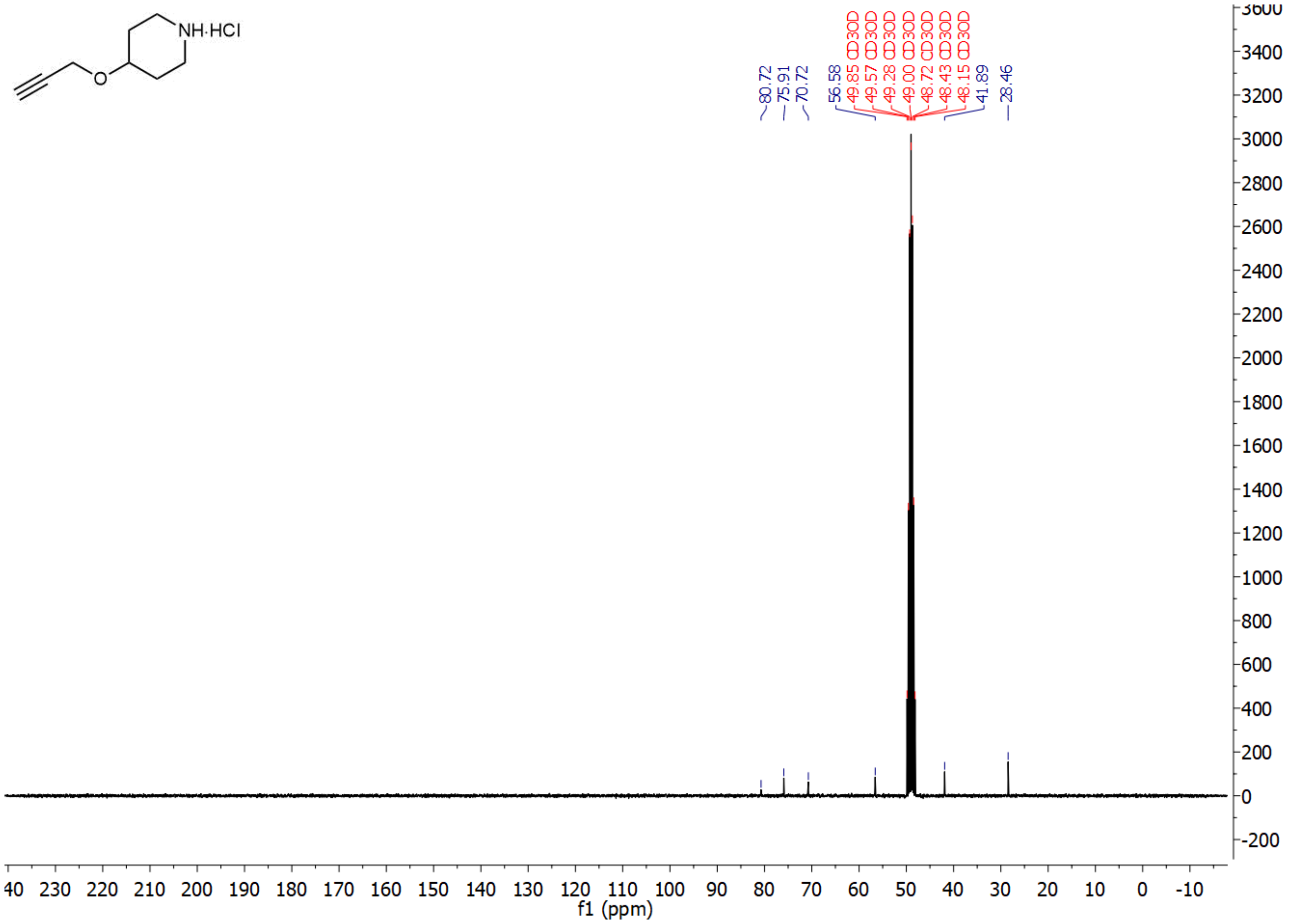

4-(Prop-2-yn-1-yloxy)piperidine-1-carboxamide (**33**) (^{1}H, 300 MHz, CD_3OD):

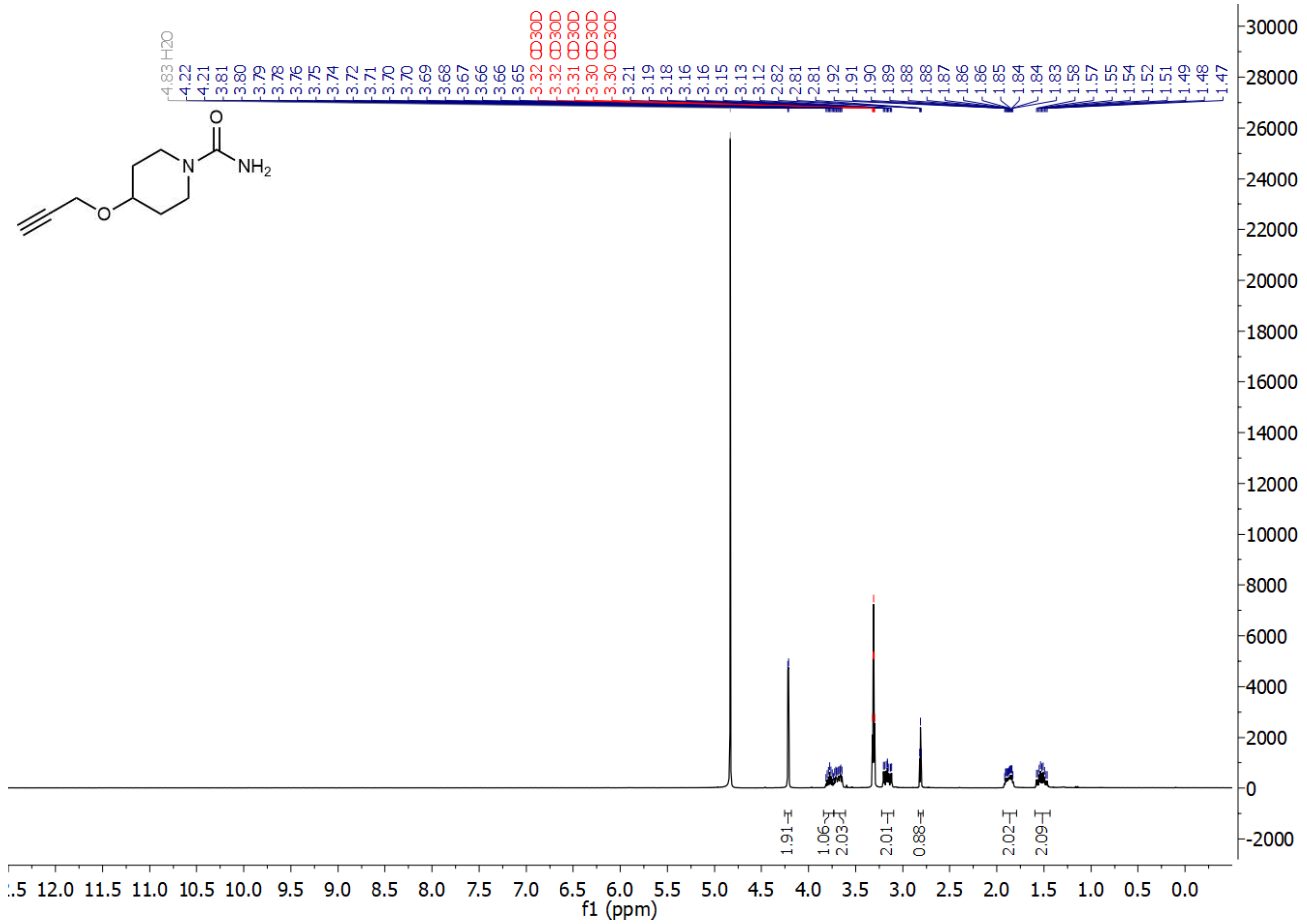

4-(Prop-2-yn-1-yloxy)piperidine-1-carboxamide (**33**) (^{13}C, 75.5 MHz, CD_3OD):

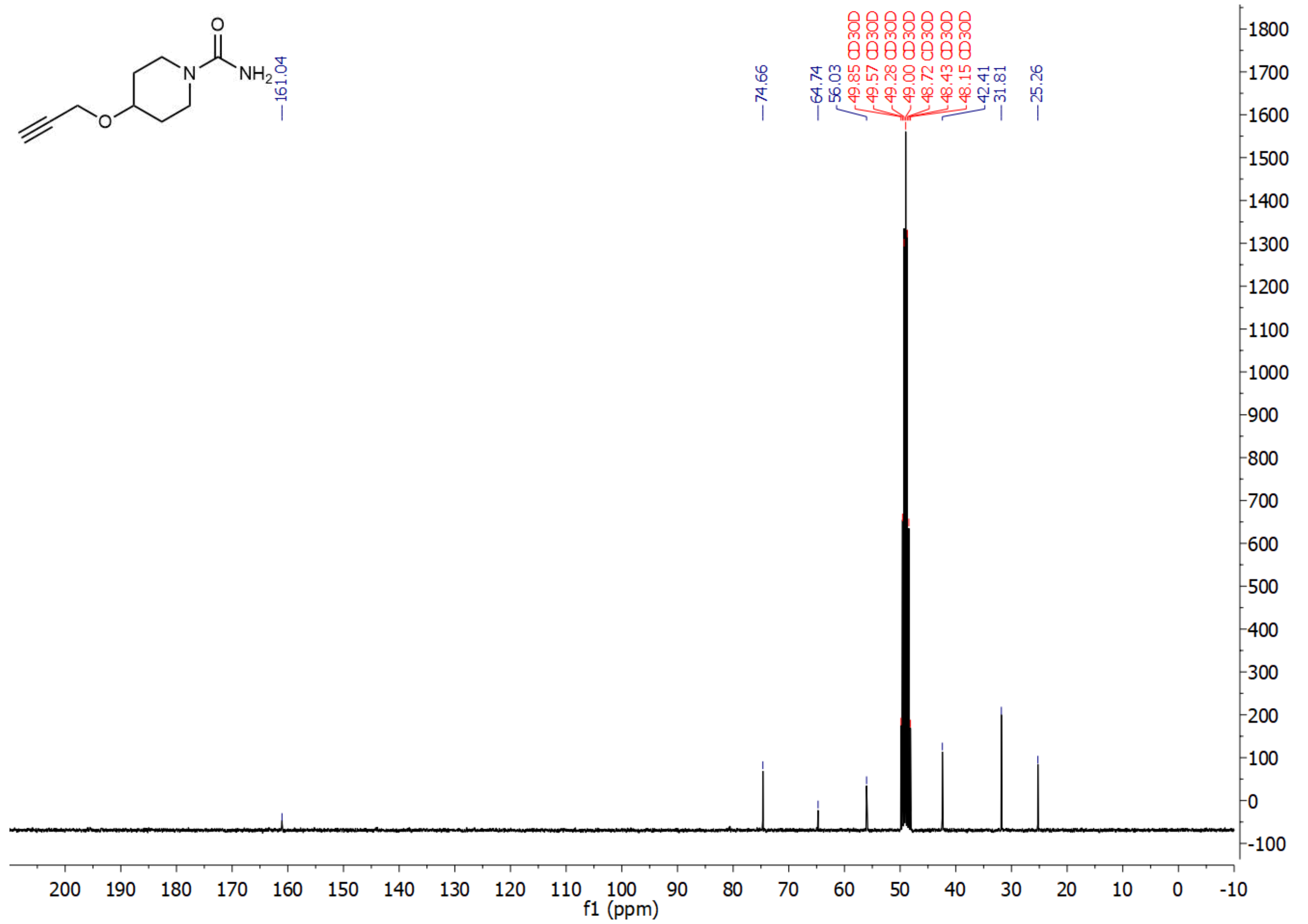

(3-Phenyl-1,2-oxaziridin-2-yl)(4-(prop-2-yn-1-yloxy)piperidin-1-yl)methanone (**OxMet2-alkyne**) (^{1}H, 500 MHz, $CDCl_3$):

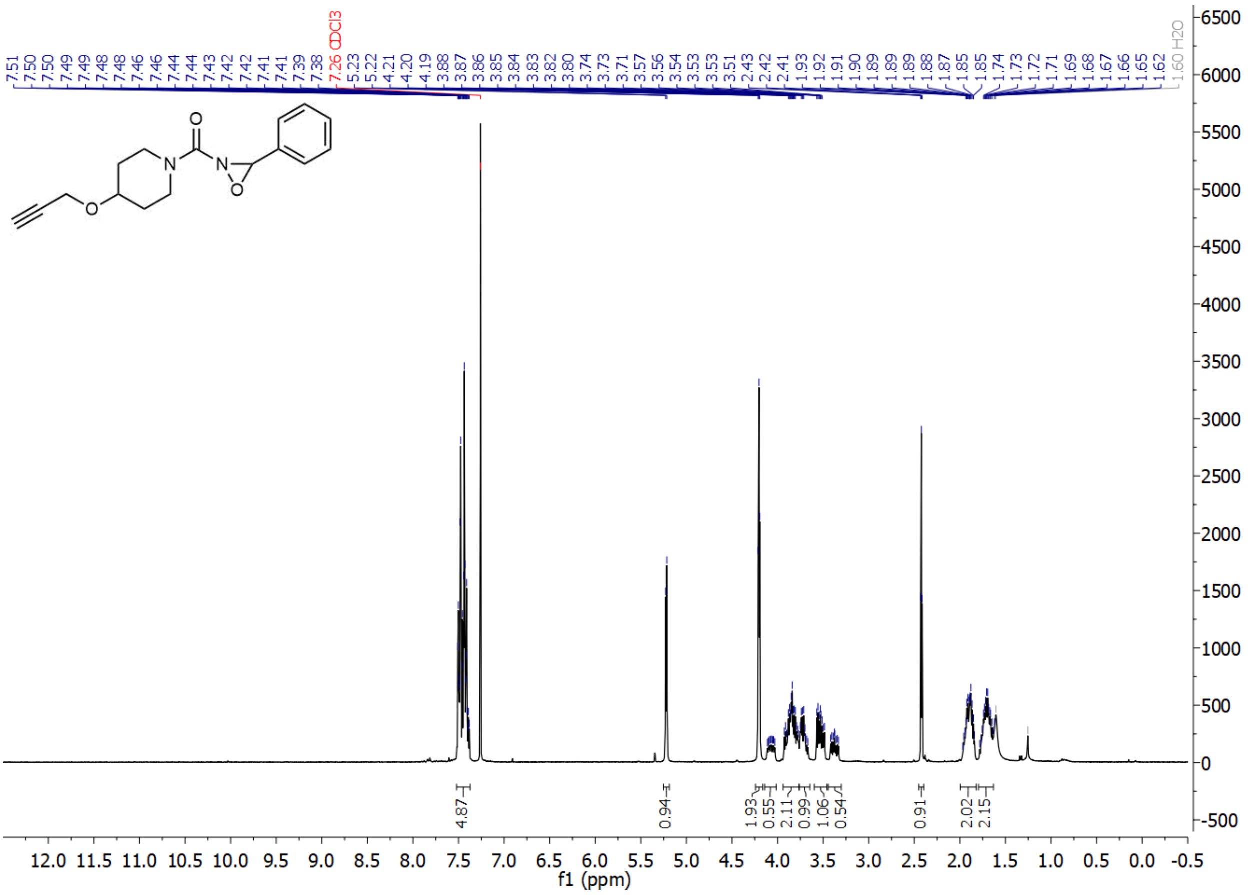

(3-Phenyl-1,2-oxaziridin-2-yl)(4-(prop-2-yn-1-yloxy)piperidin-1-yl)methanone (**OxMet2-alkyne**) (^{13}C, 75.5 MHz, $CDCl_3$):

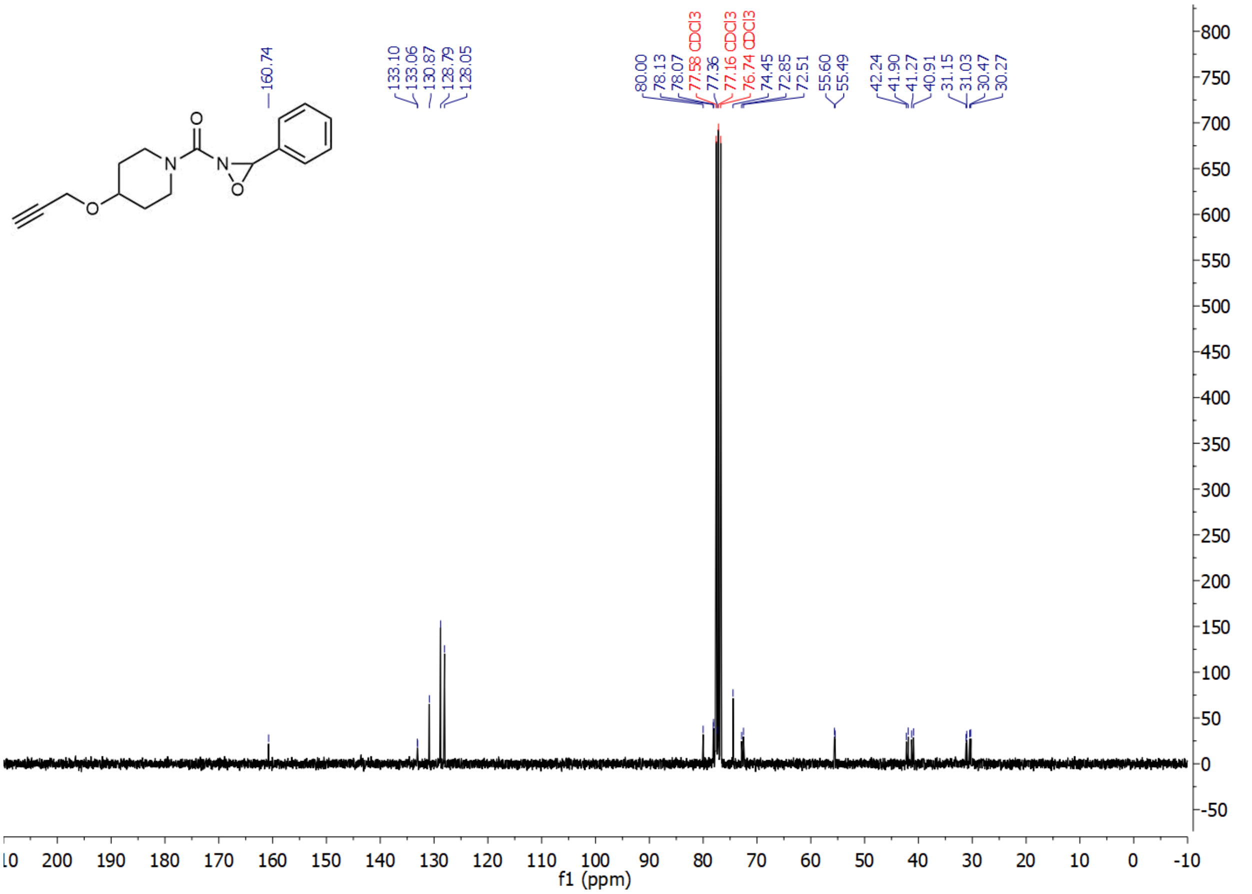

Pent-4-yn-1-yl 1-methylhydrazine-1-carboxylate (**34**) (^{1}H, 500 MHz, $CDCl_3$):

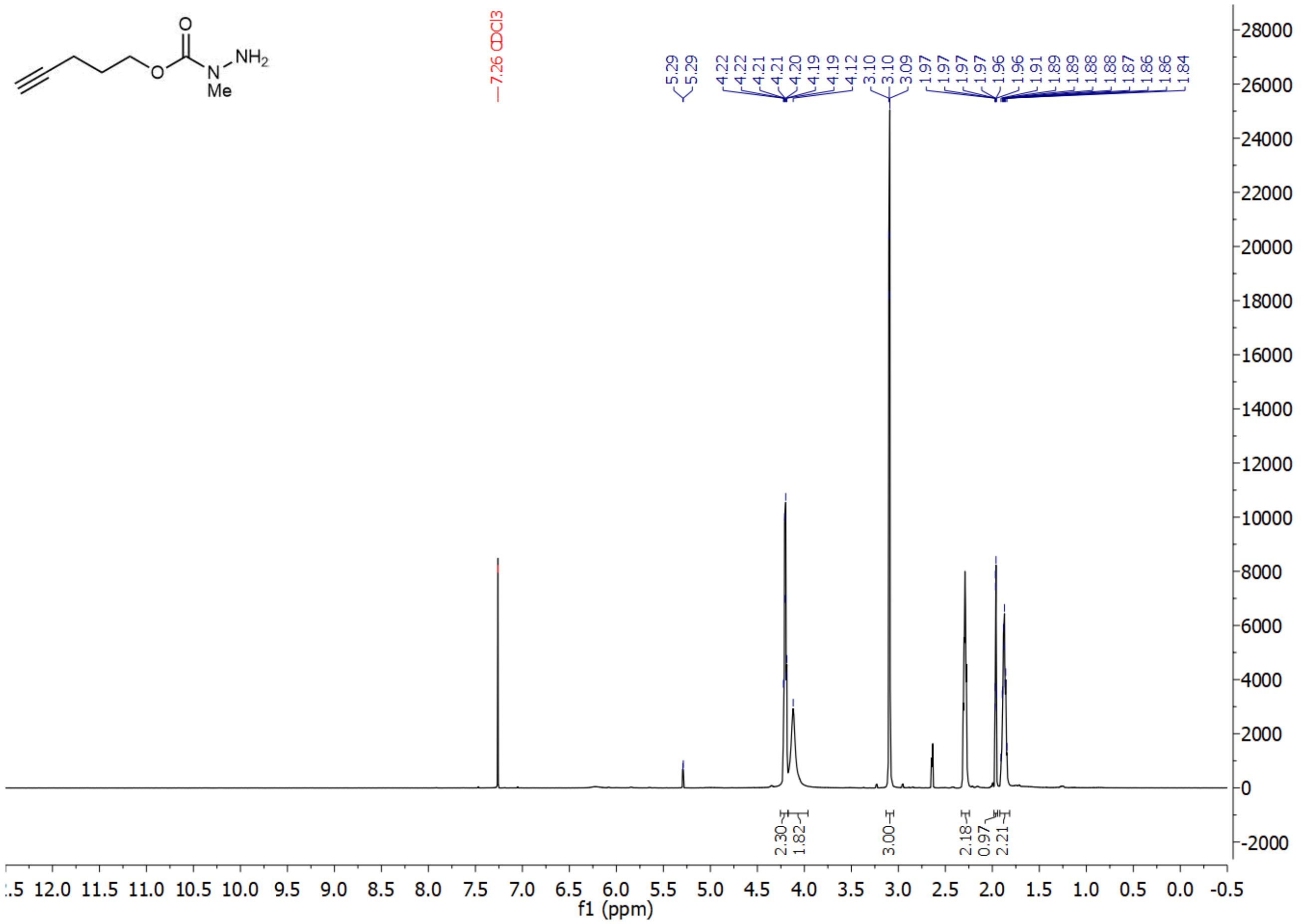

Pent-4-yn-1-yl 1-methylhydrazine-1-carboxylate (**34**) (^{13}C, 101 MHz, $CDCl_3$):

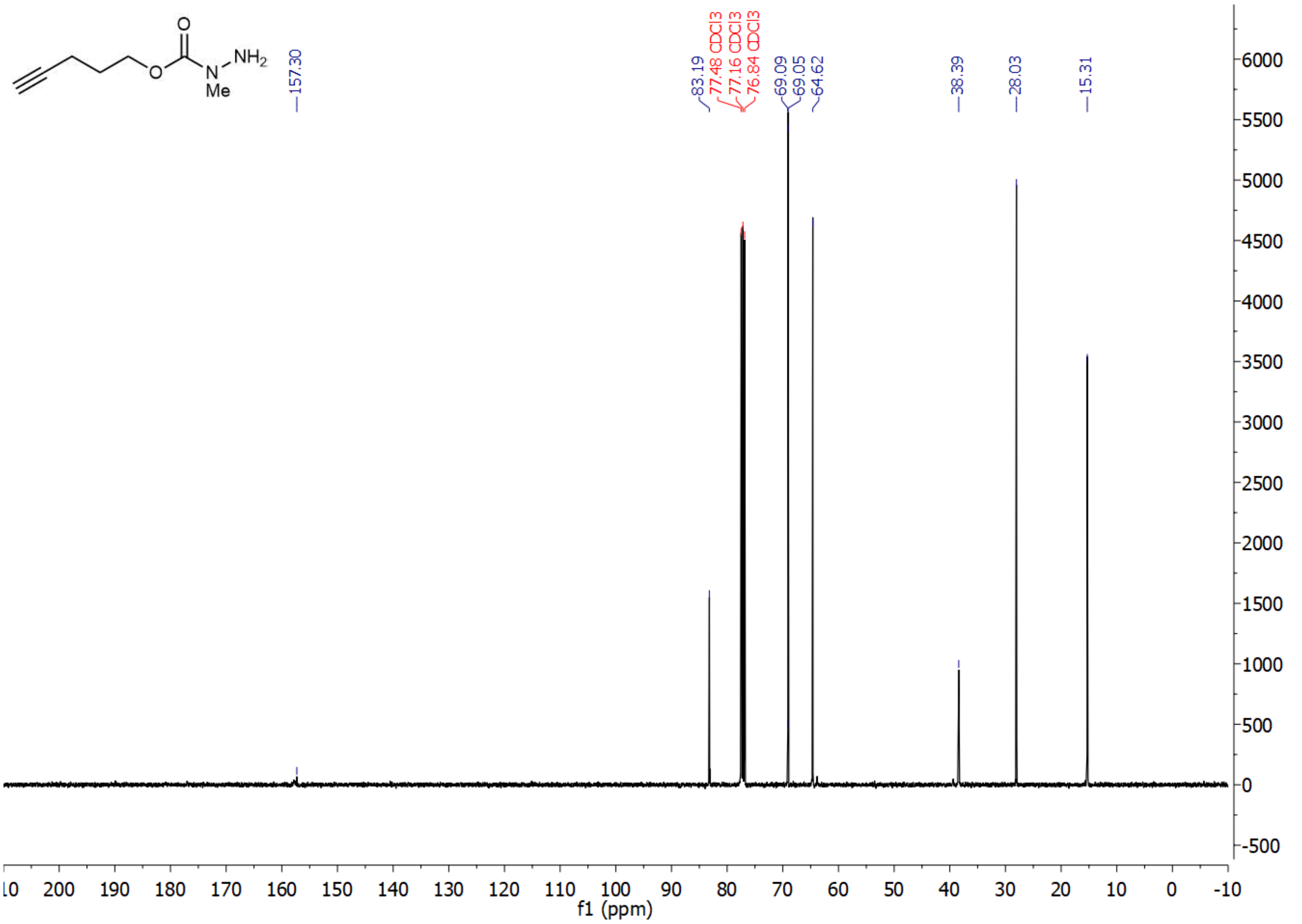

2,4,6-Trimethyl-1-(methyl((pent-4-yn-1-yloxy)carbonyl)amino)pyridin-1-ium tetrafluoroborate (**CP-alkyne**) (^{1}H, 300 MHz, DMSO-d$_6$):

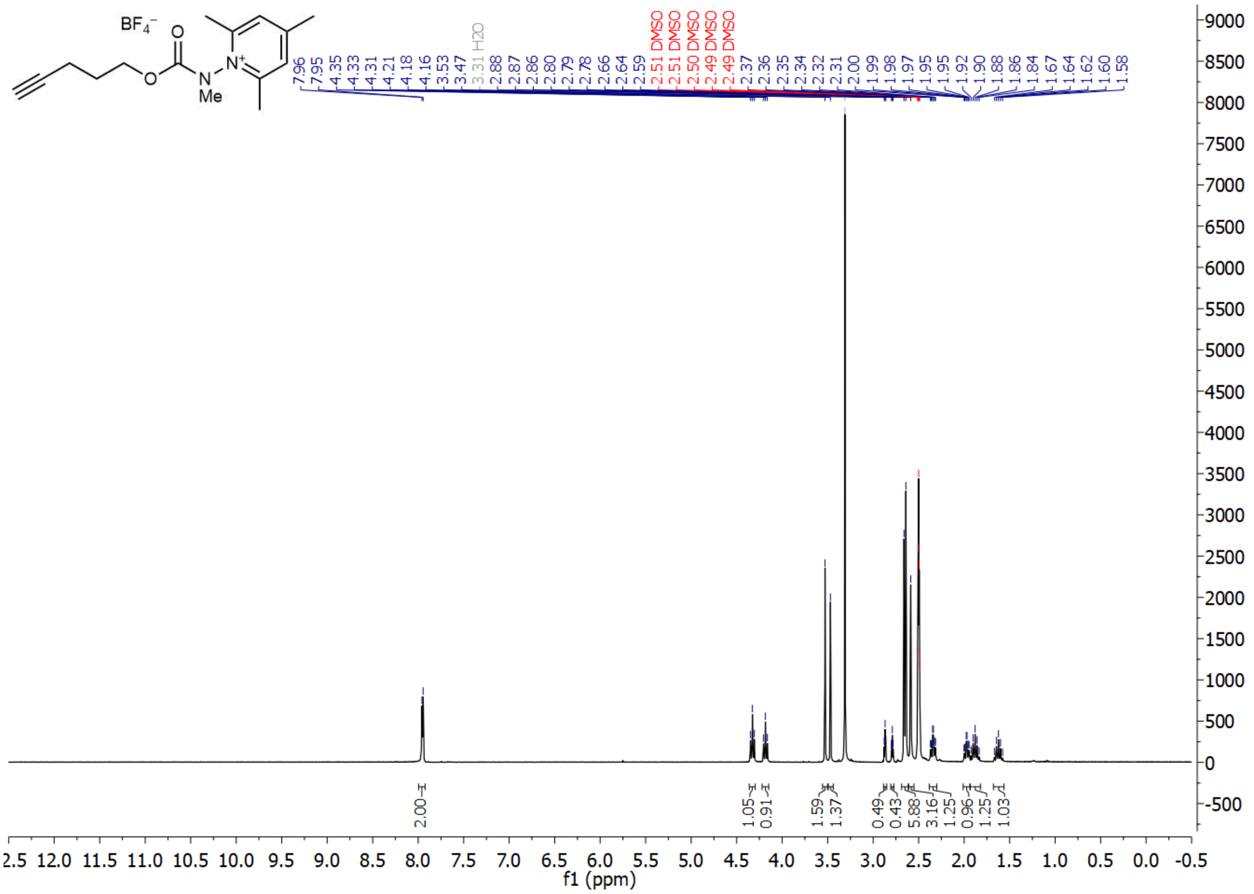

2,4,6-Trimethyl-1-(methyl((pent-4-yn-1-yloxy)carbonyl)amino)pyridin-1-ium tetrafluoroborate (**CP-alkyne**) (^{13}C, 101 MHz, DMSO-d$_6$):

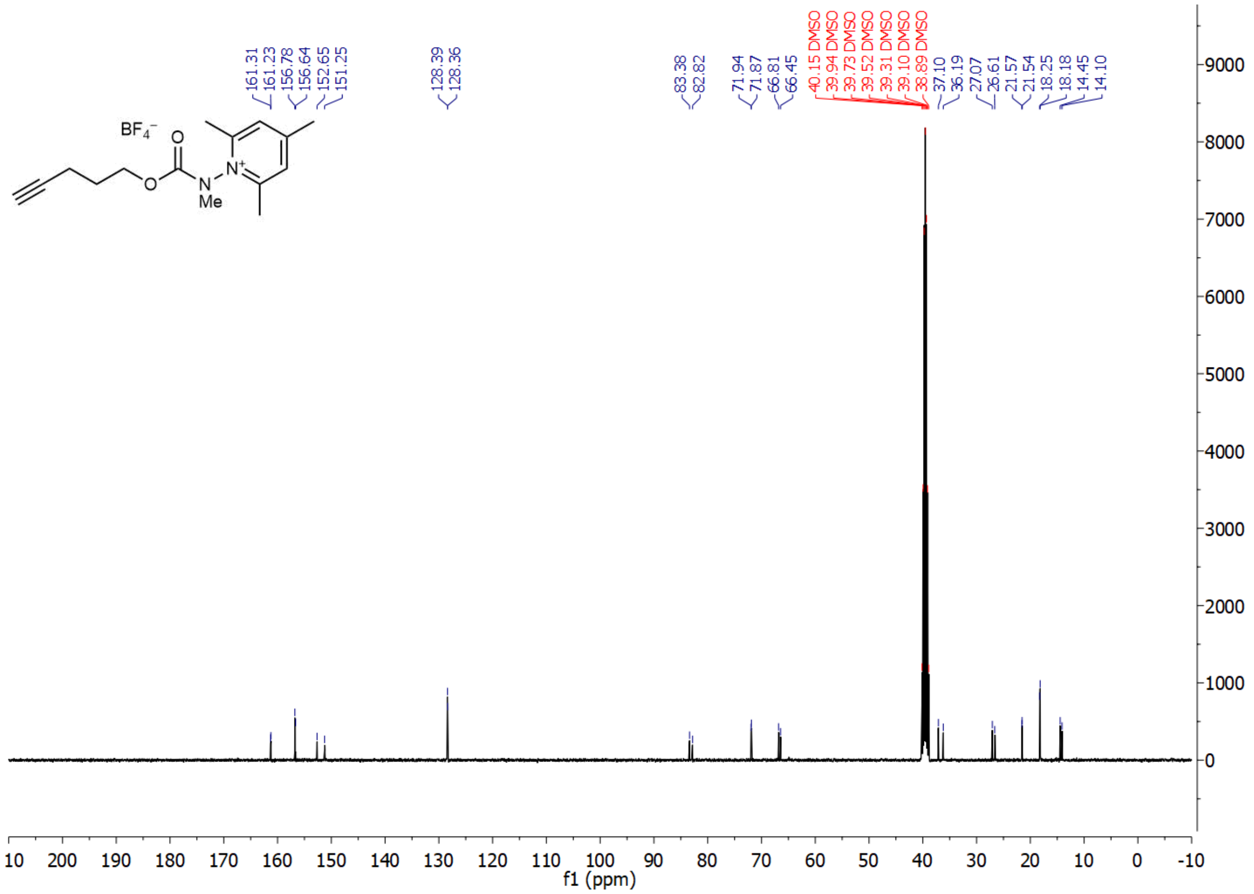

2,4,6-Trimethyl-1-(methyl((pent-4-yn-1-yloxy)carbonyl)amino)pyridin-1-ium tetrafluoroborate (**CP-alkyne**) (^{19}F, 376 MHz, DMSO-d$_6$):

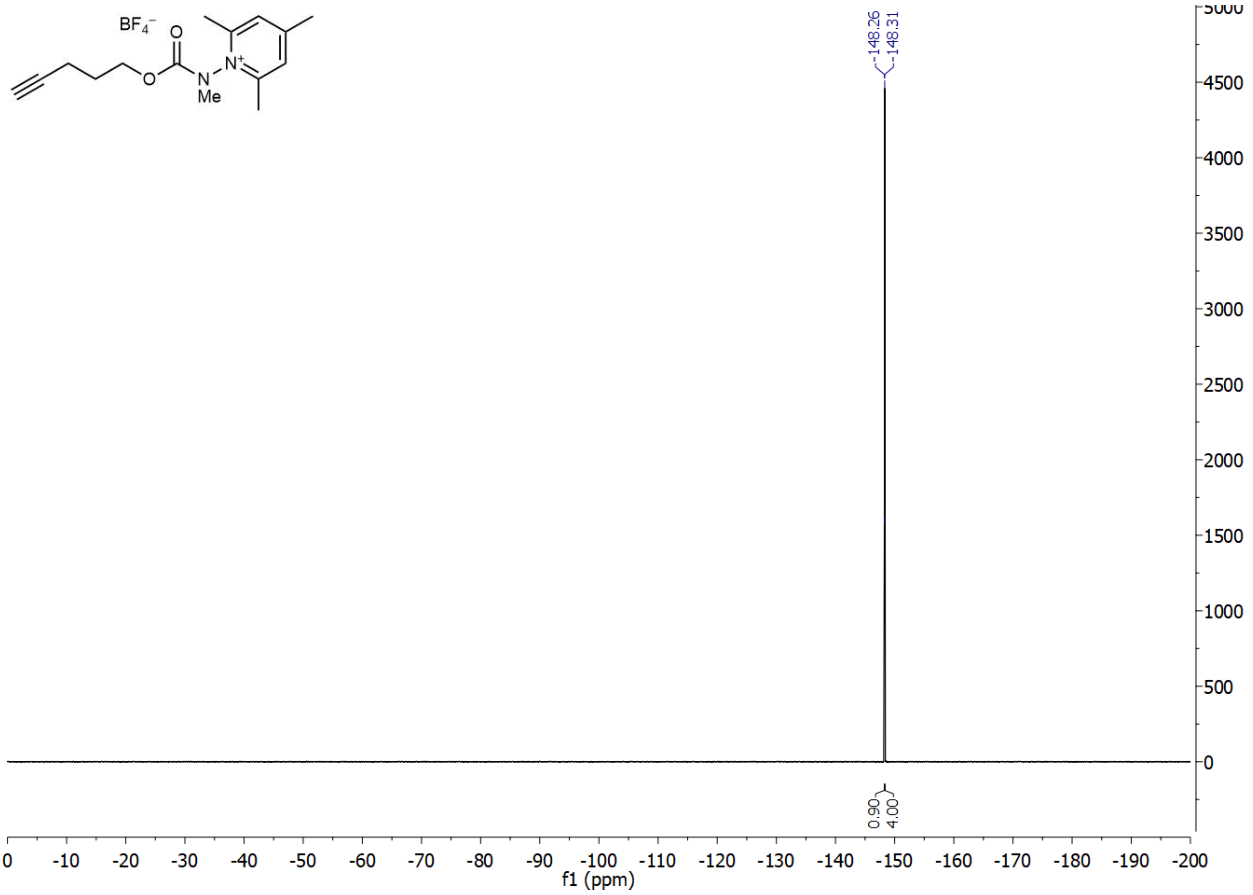

O-(Pent-4-yn-1-yl) phosphorodichloridothioate (**TPAC-alkyne**) (^{1}H, 300 MHz, $CDCl_3$):

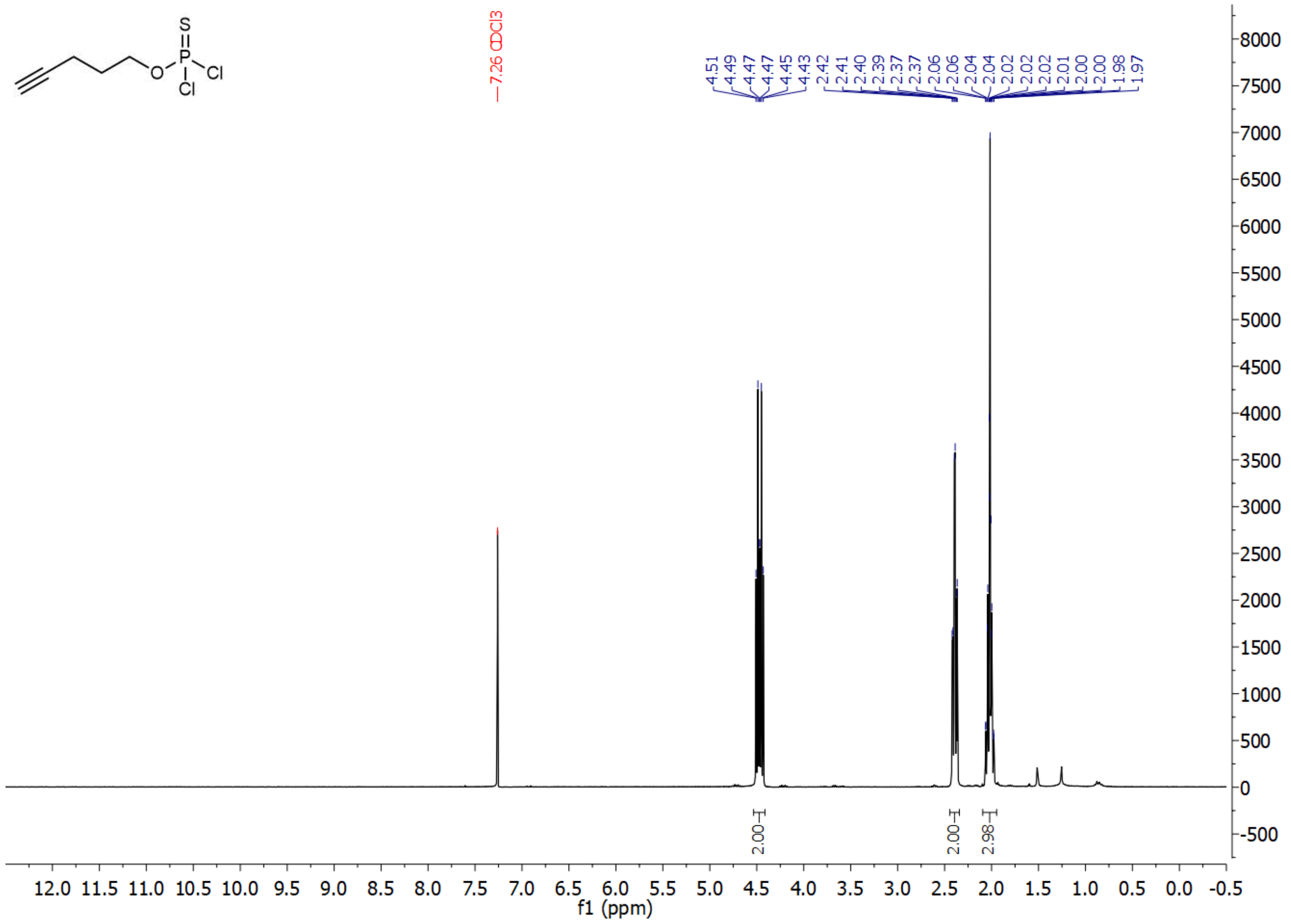

O-(Pent-4-yn-1-yl) phosphorodichloridothioate (**TPAC-alkyne**) (^{13}C, 75.5 MHz, $CDCl_3$):

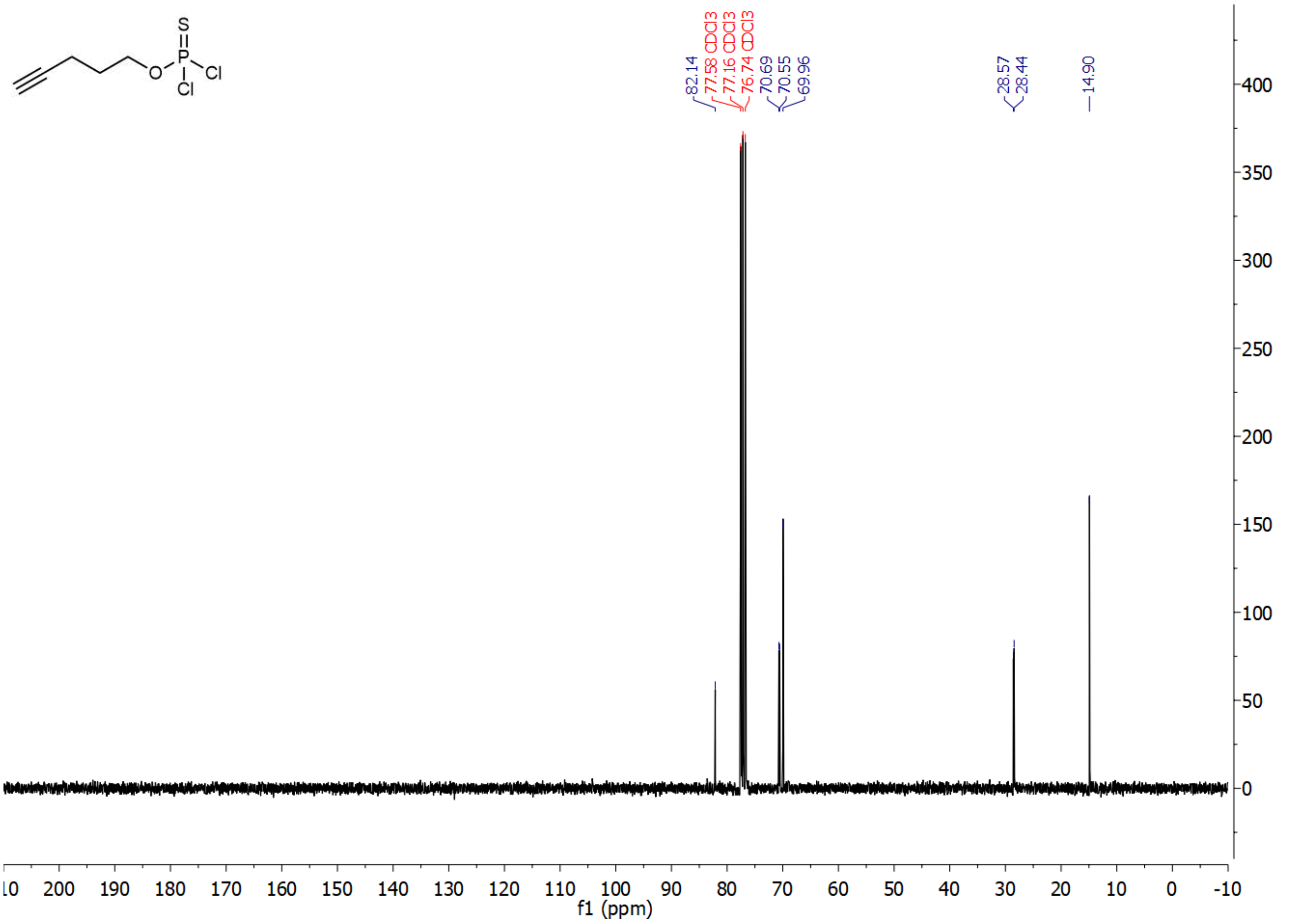

O-(Pent-4-yn-1-yl) phosphorodichloridothioate (**TPAC-alkyne**) (^{31}P, 122 MHz, $CDCl_3$):

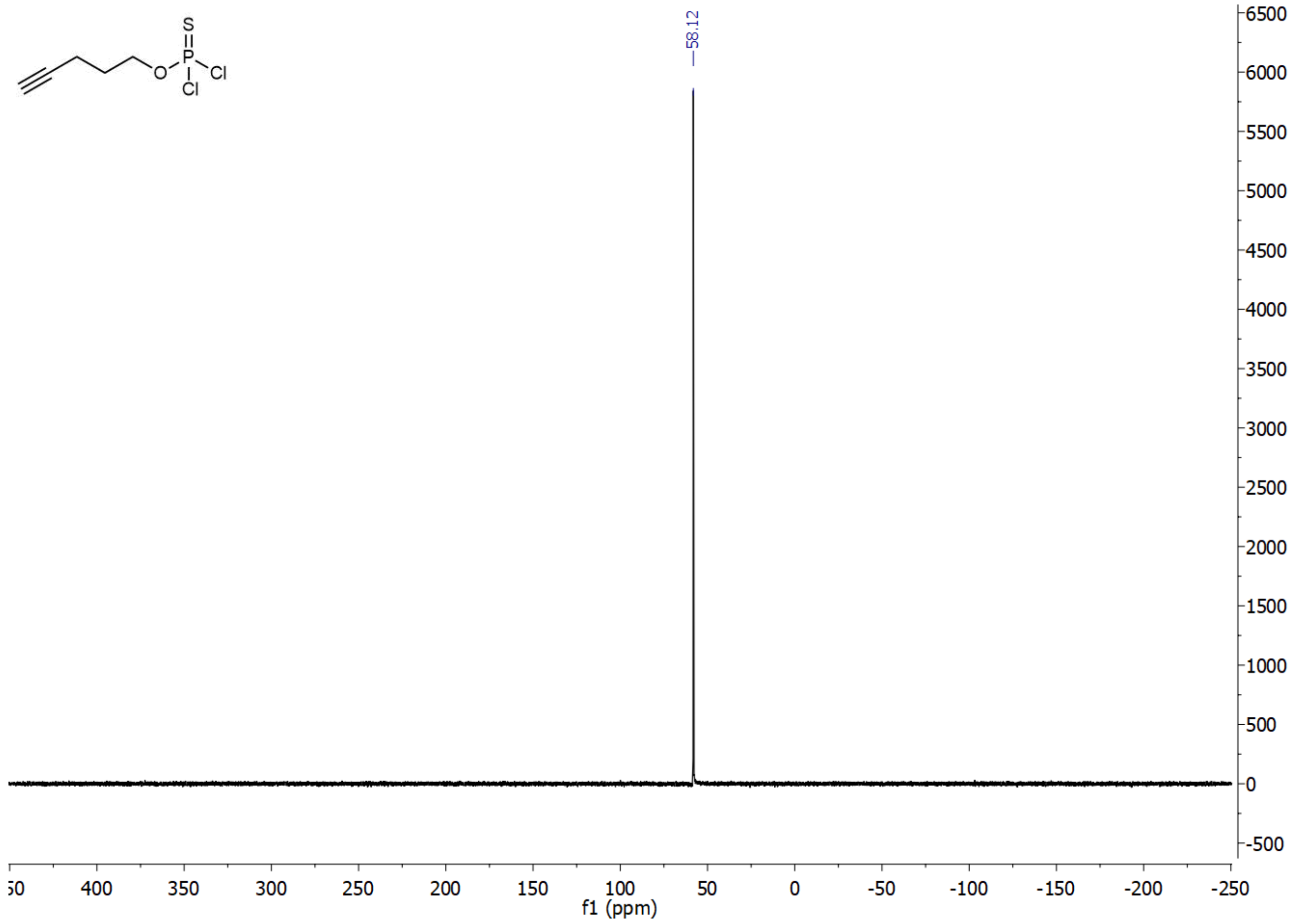

Methyl 3,5-dihydroxy-2-naphthoate (**35**) (^{1}H, 500 MHz, DMSO-d_6):

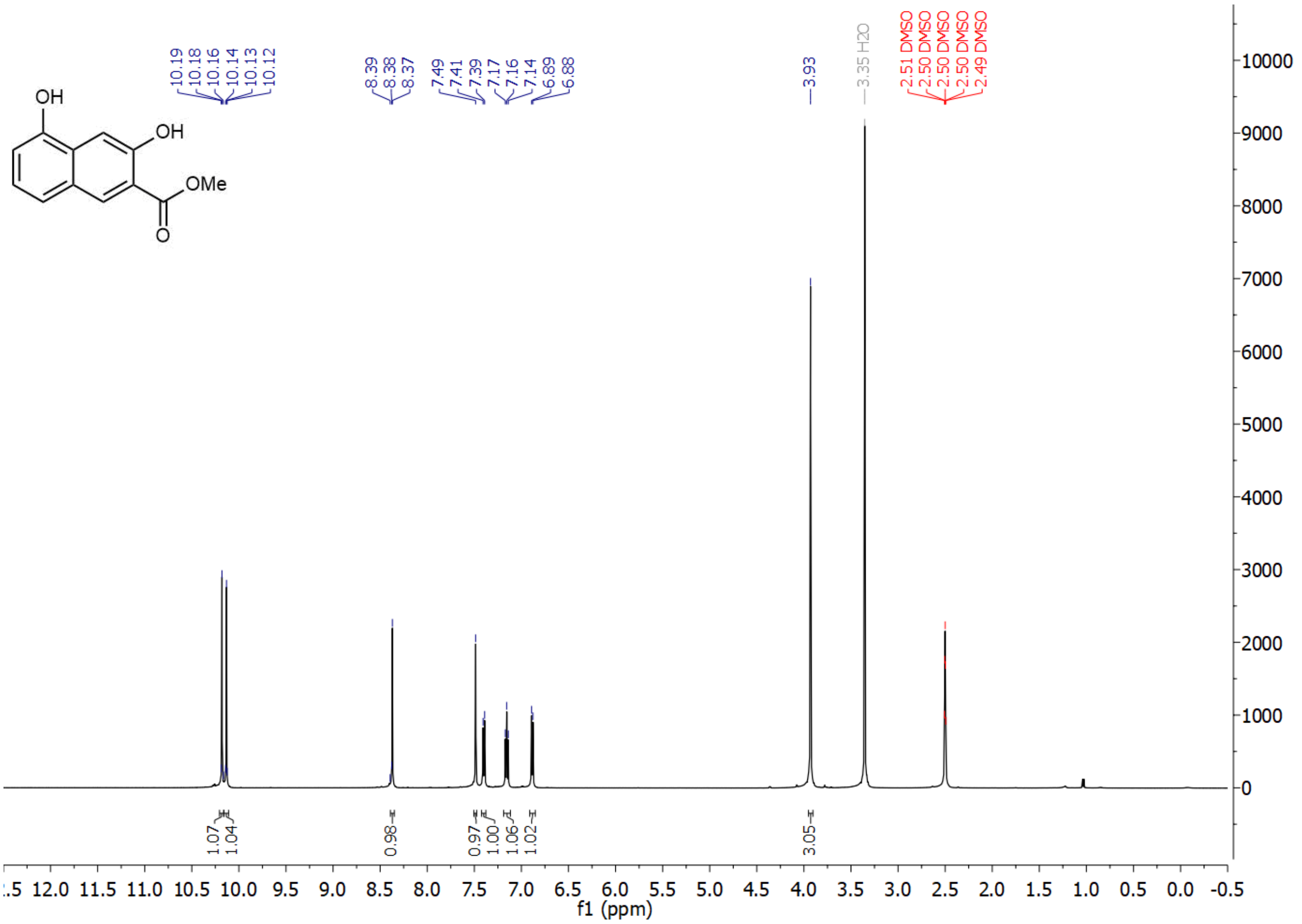

Methyl 3,5-dihydroxy-2-naphthoate (**35**) (^{13}C, 126 MHz, DMSO-d_6):

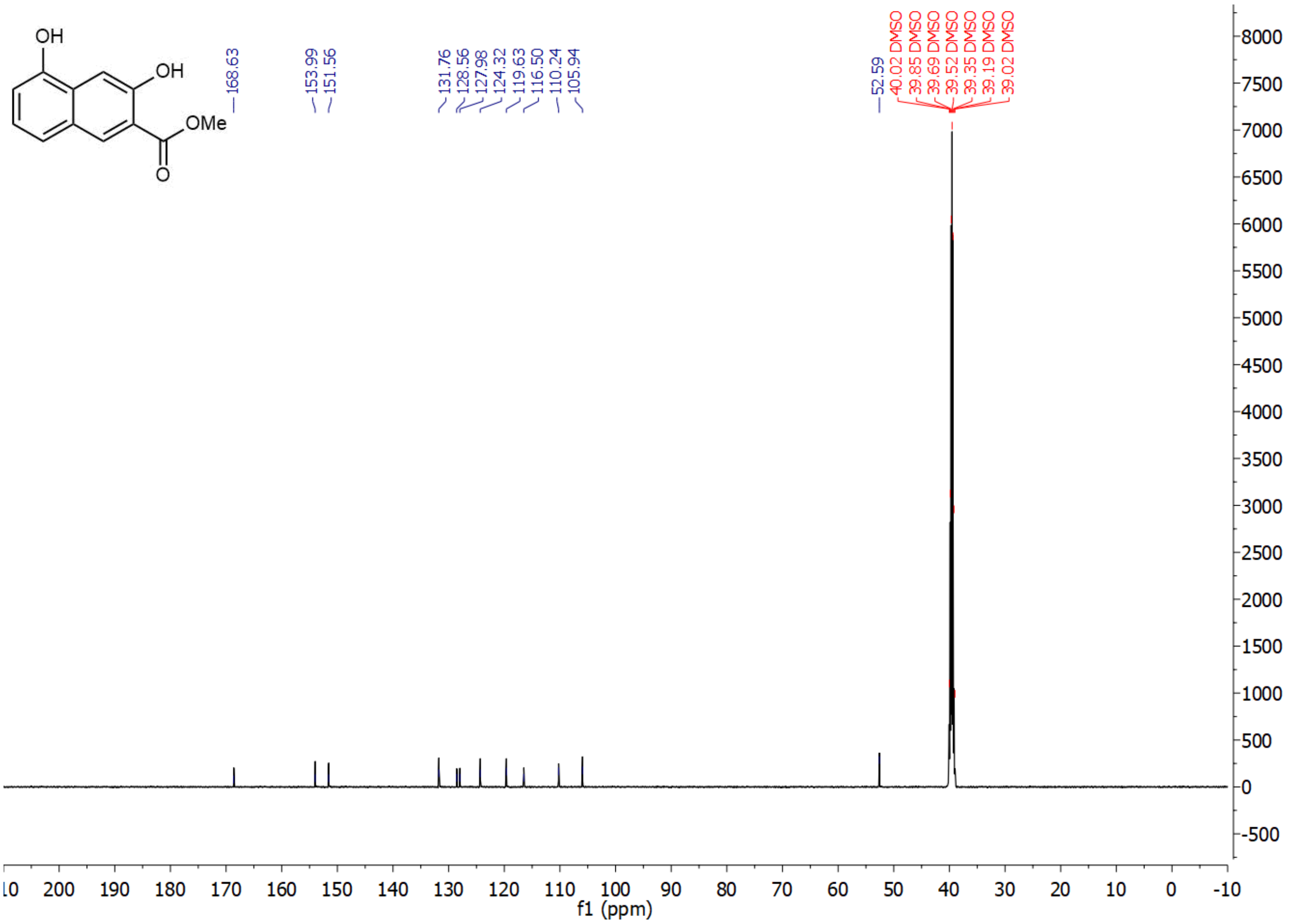

6-(Hydroxymethyl)naphthalene-1,7-diol (**36**) (^{1}H, 400 MHz, DMSO-d_6):

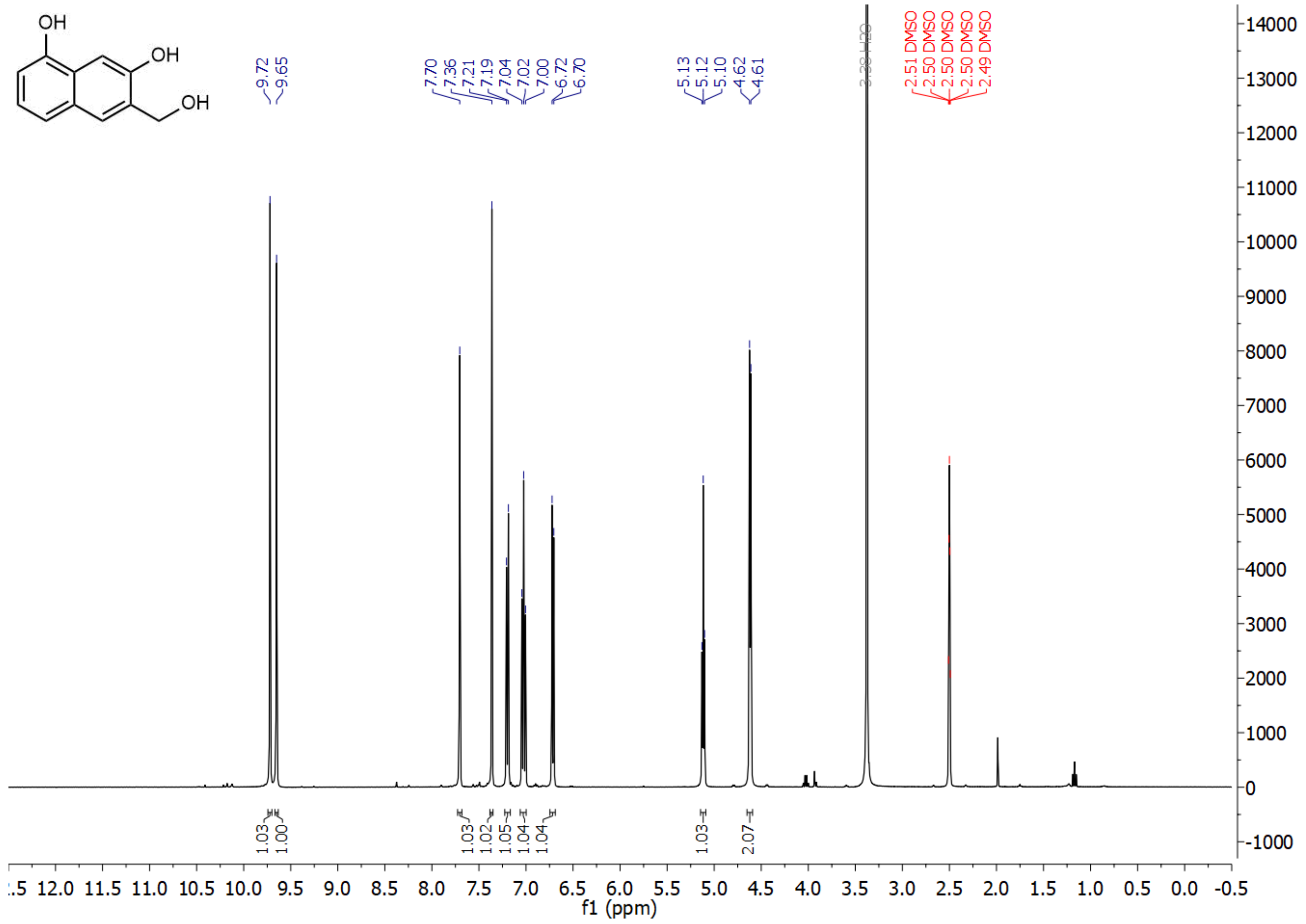

6-(Hydroxymethyl)naphthalene-1,7-diol (**36**) (^{13}C, 126 MHz, DMSO-d_6):

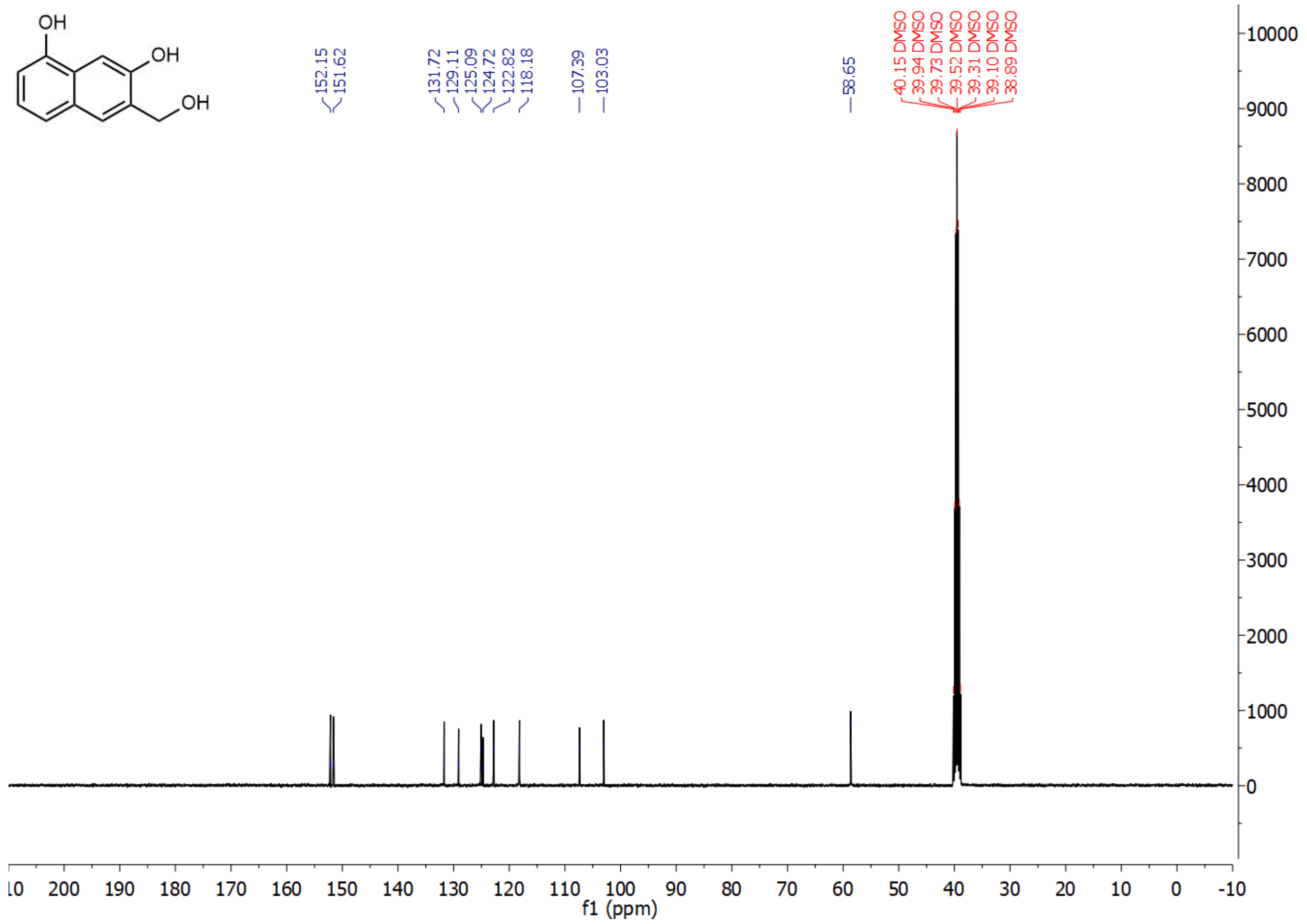

2,2-Dimethyl-4*H*-naphtho[2,3-*d*][1,3]dioxin-9-ol (**37**) (^{1}H, 500 MHz, DMSO-d$_6$):

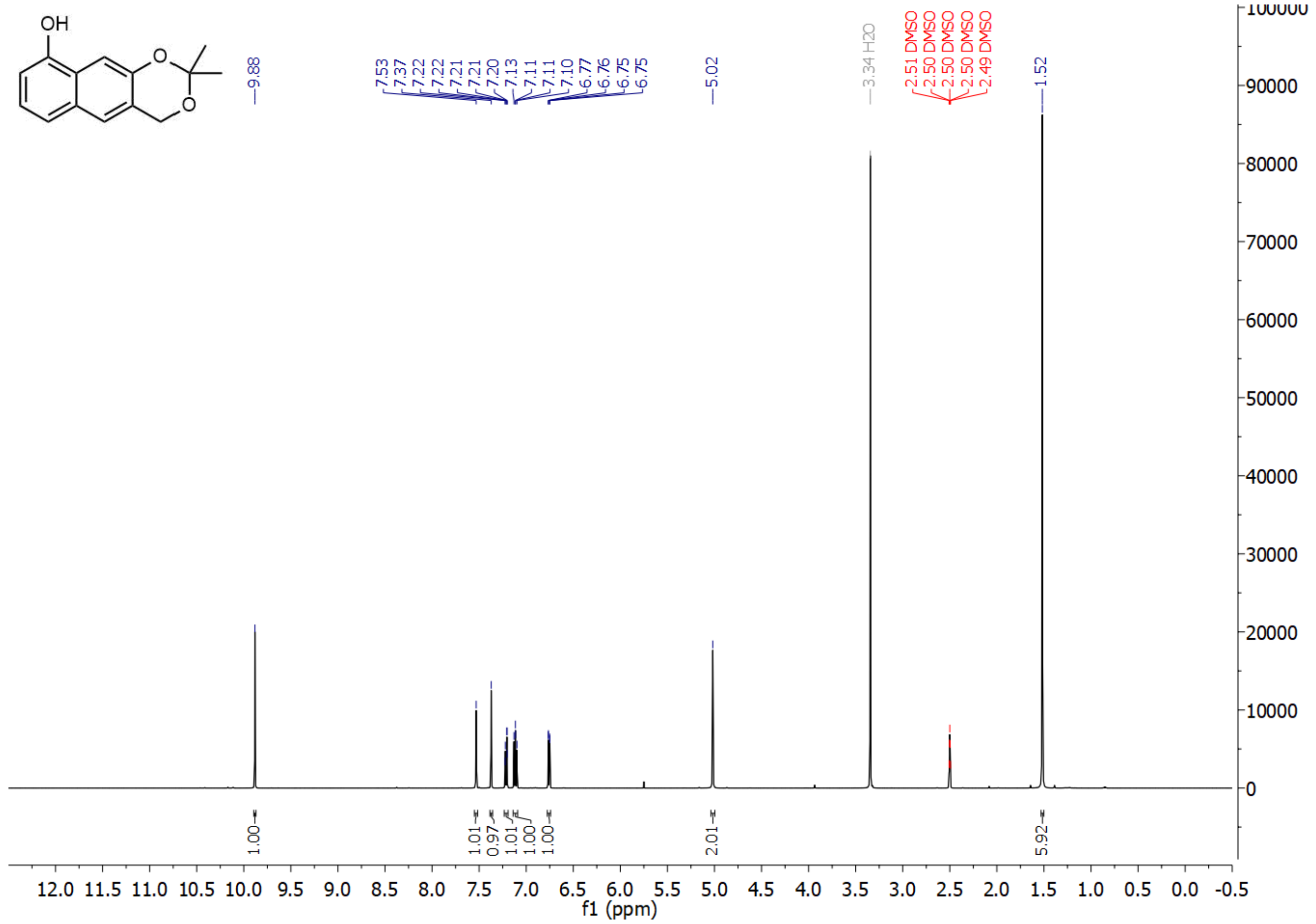

2,2-Dimethyl-4*H*-naphtho[2,3-*d*][1,3]dioxin-9-ol (**37**) (^{13}C, 101 MHz, DMSO-d$_6$):

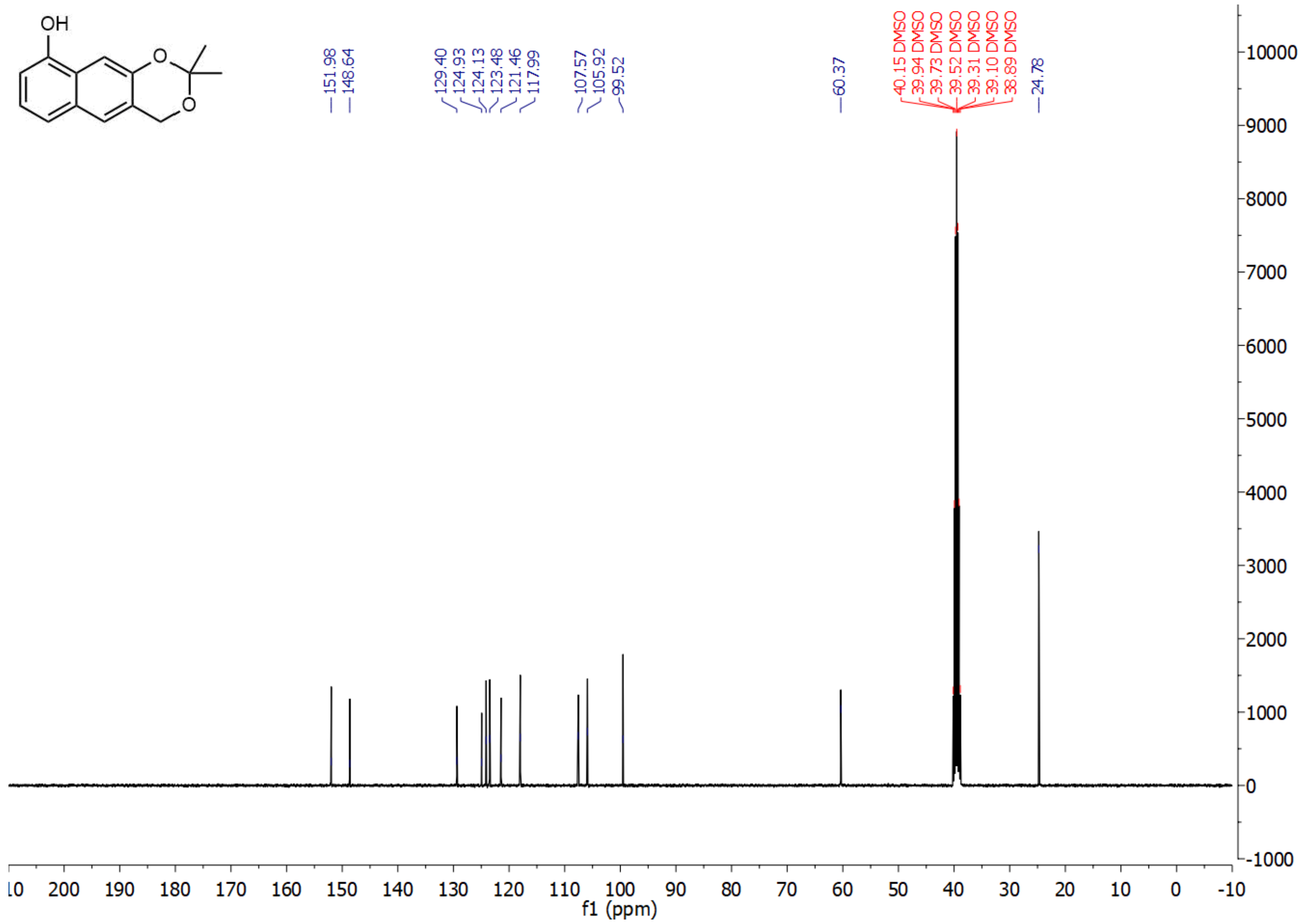

2,2-Dimethyl-9-(pent-4-yn-1-yloxy)-4*H*-naphtho[2,3-*d*][1,3]dioxine (**38**) (^{1}H, 500 MHz, DMSO-d6):

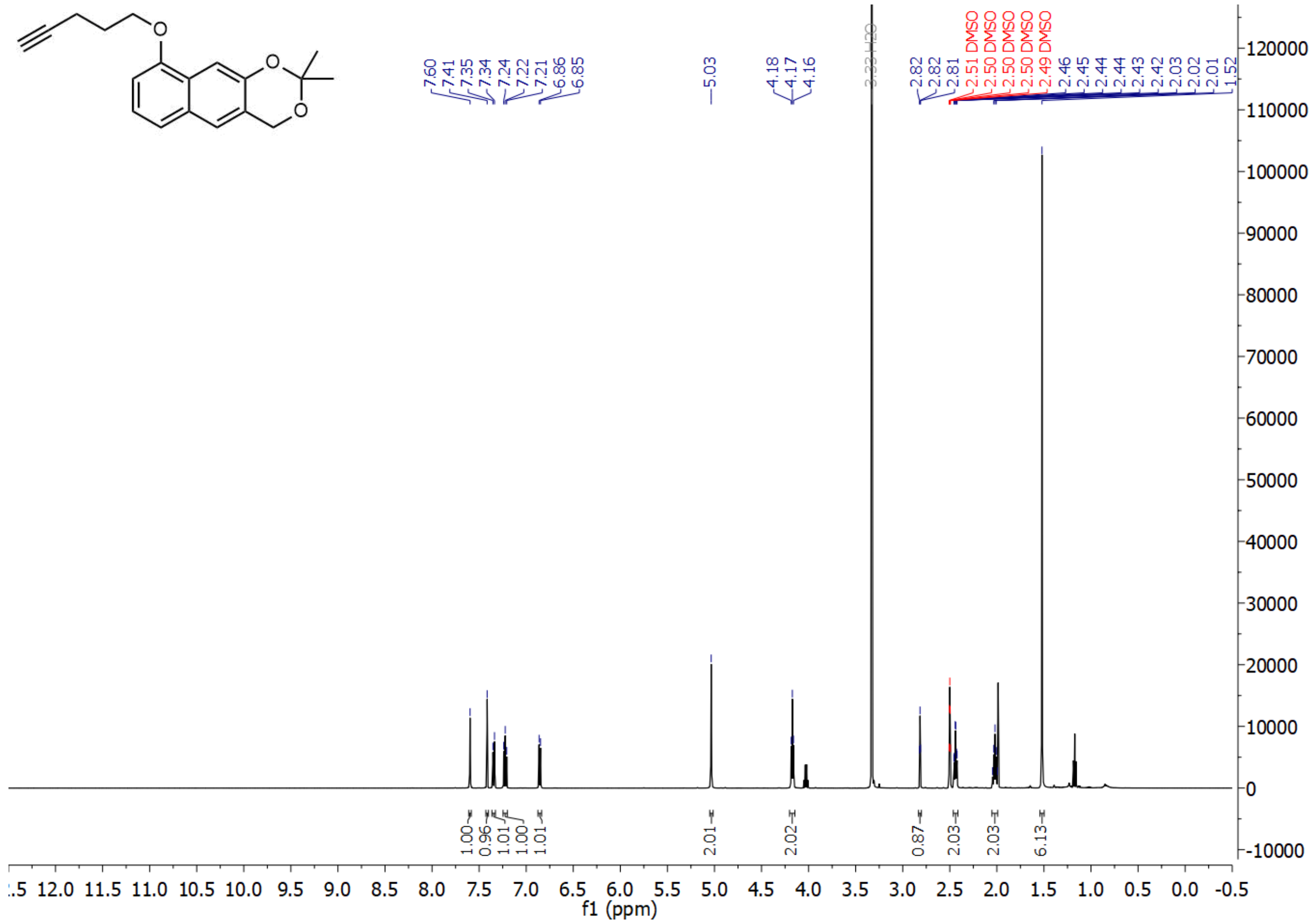

2,2-Dimethyl-9-(pent-4-yn-1-yloxy)-4*H*-naphtho[2,3-*d*][1,3]dioxine (**38**) (^{13}C, 101 MHz, DMSO-d6):

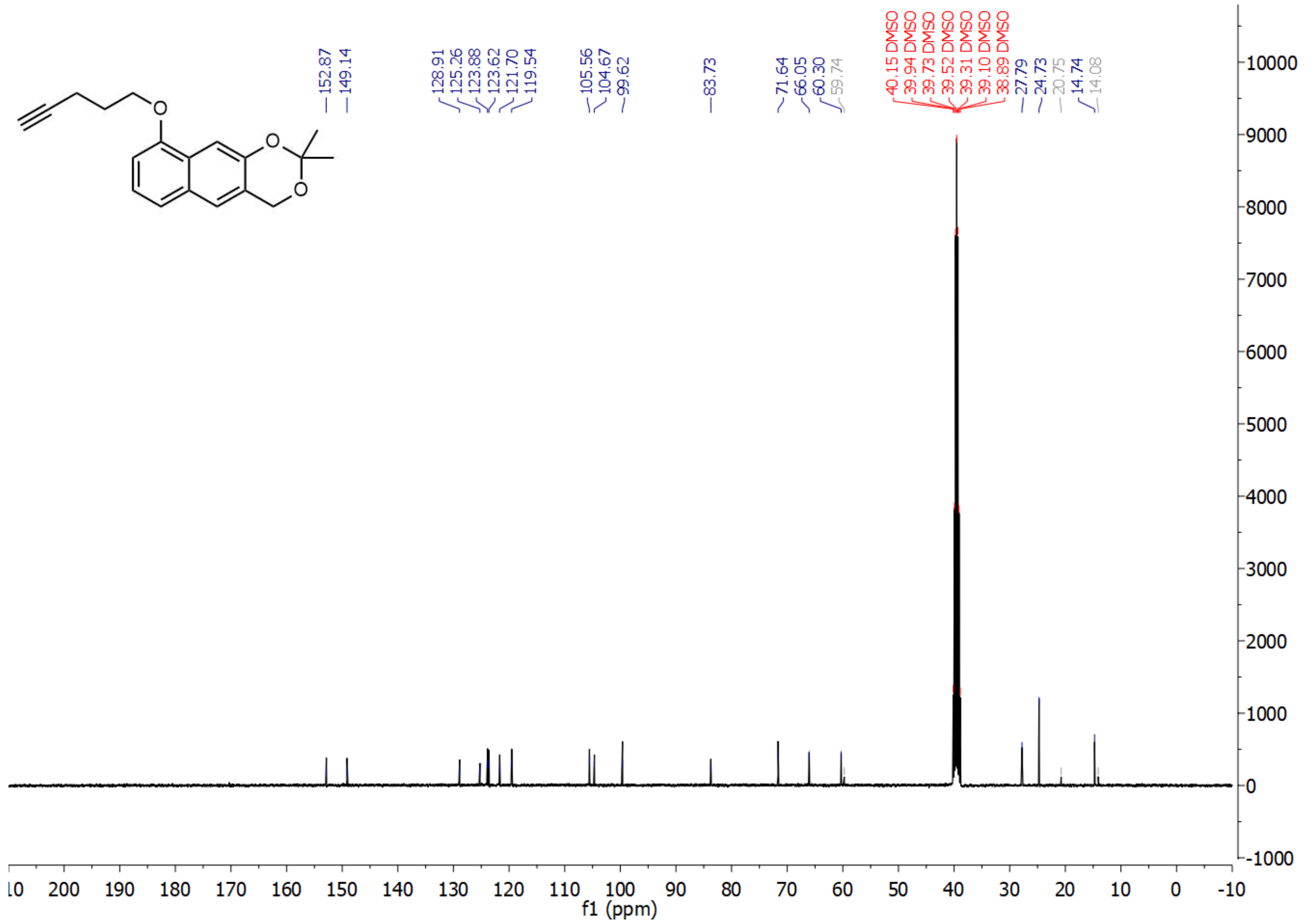

3-(Hydroxymethyl)-8-(pent-4-yn-1-yloxy)naphthalen-2-ol (**HMN-alkyne**) (^{1}H, 500 MHz, DMSO-d_6):

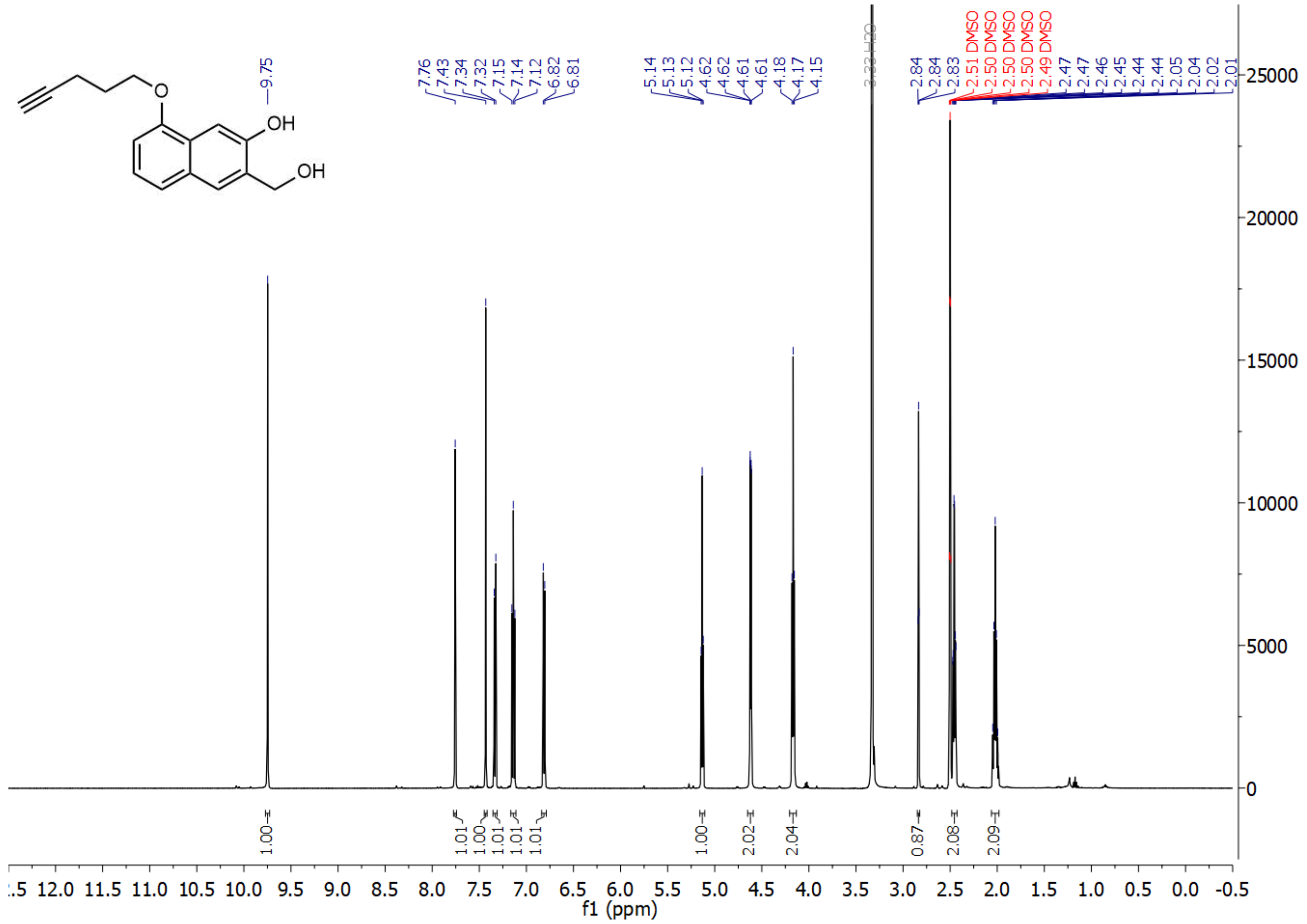

3-(Hydroxymethyl)-8-(pent-4-yn-1-yloxy)naphthalen-2-ol (**HMN-alkyne**) (^{13}C, 101 MHz, DMSO-d_6):

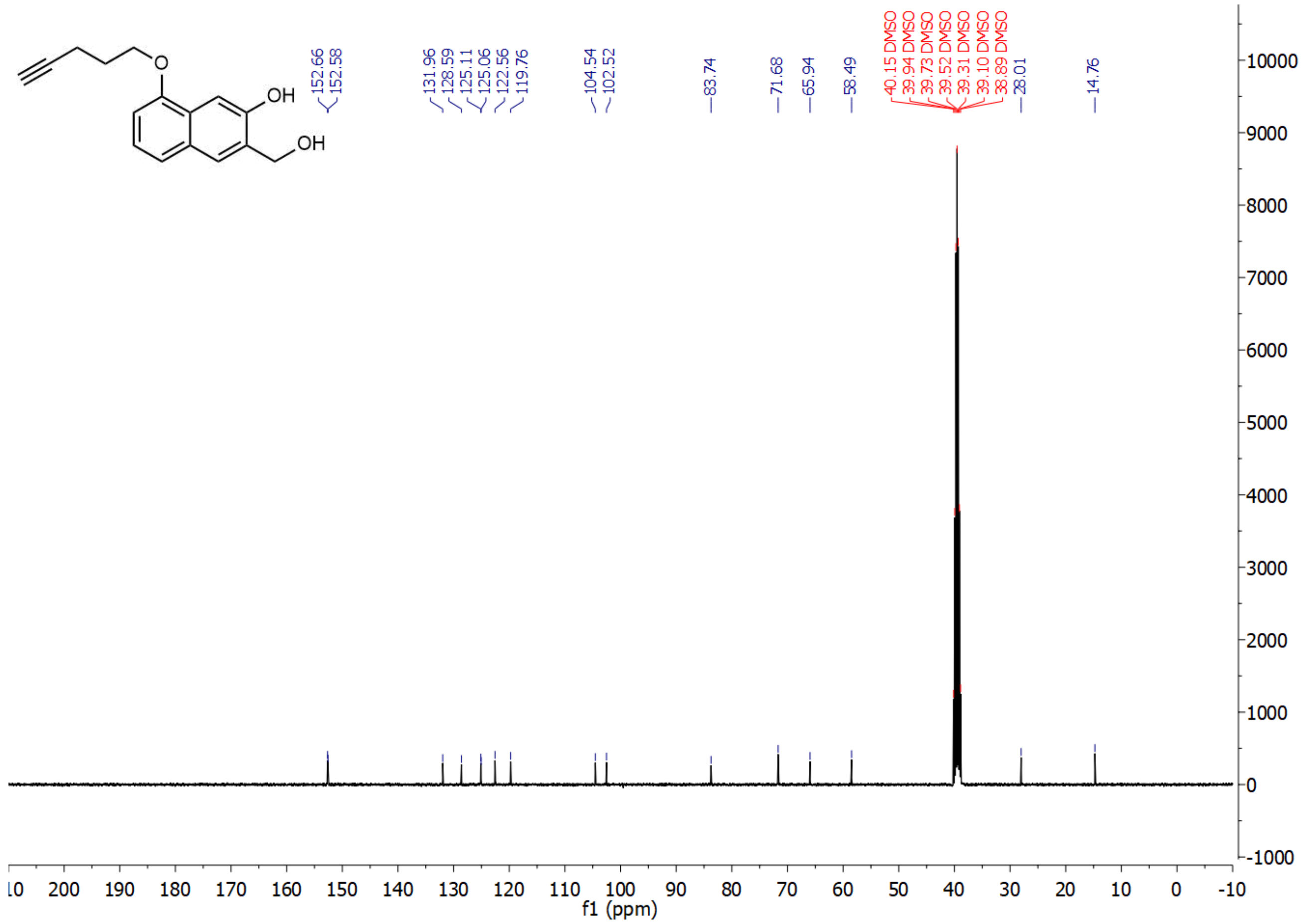

3-Formyl-4-hydroxy-*N*-(prop-2-yn-1-yl)benzenesulfonamide (**40**) (^{1}H, 400 MHz, CD_3CN):

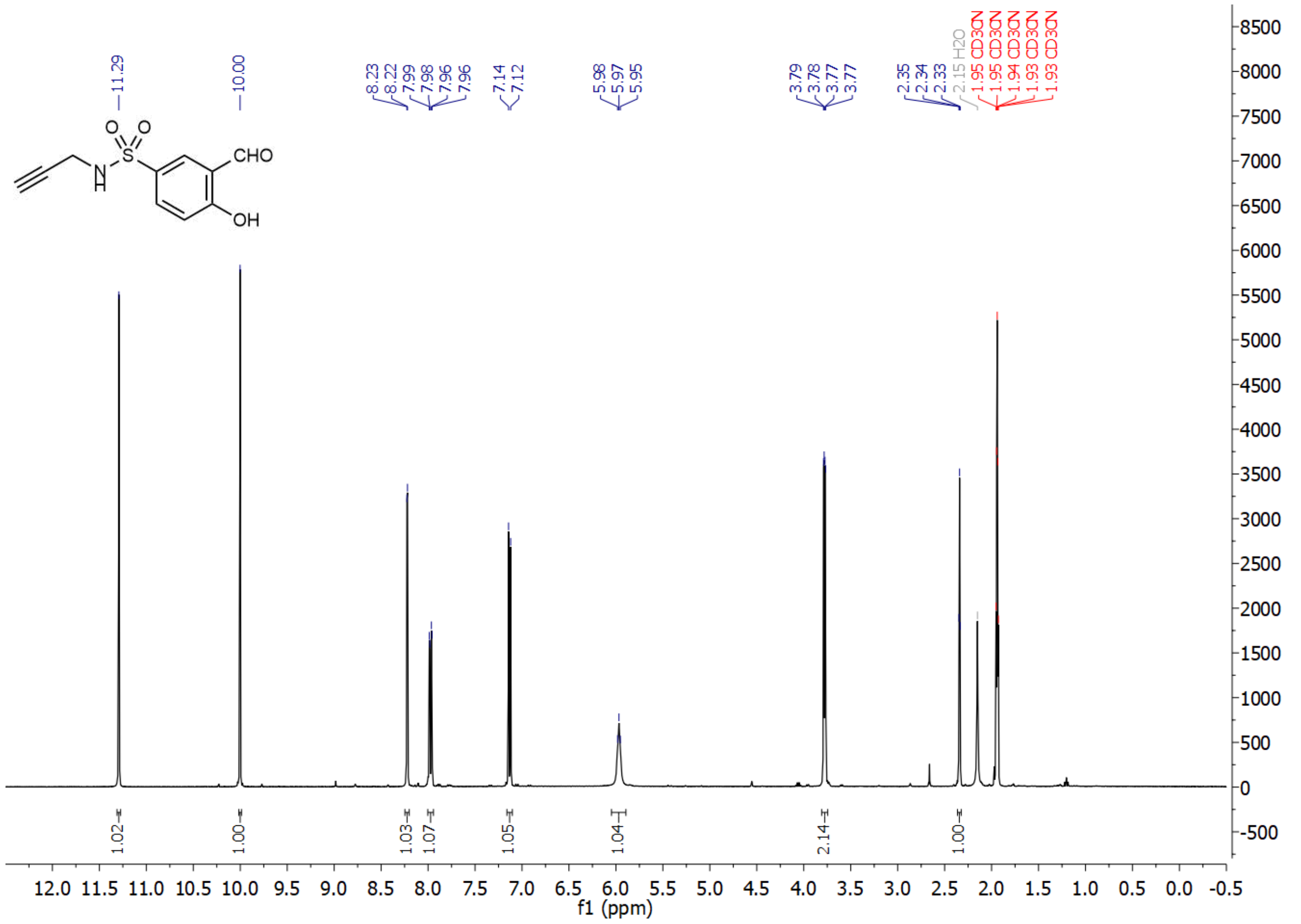

3-Formyl-4-hydroxy-*N*-(prop-2-yn-1-yl)benzenesulfonamide (**40**) (^{13}C, 101 MHz, CD_3CN):

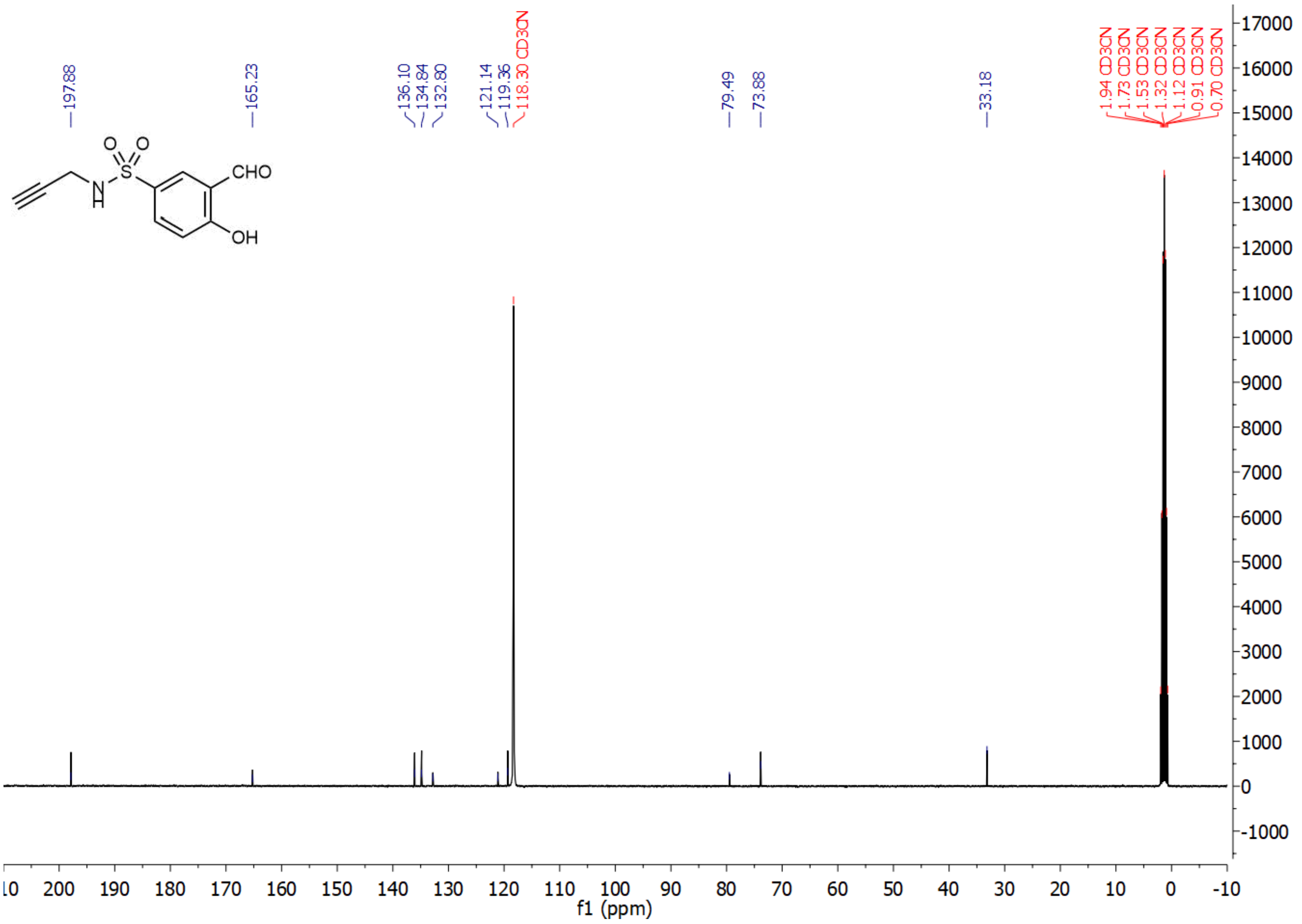

4-Hydroxy-3-(hydroxymethyl)-*N*-(prop-2-yn-1-yl)benzenesulfonamide (**HMP-alkyne**) (^{1}H, 400 MHz, CD_3CN):

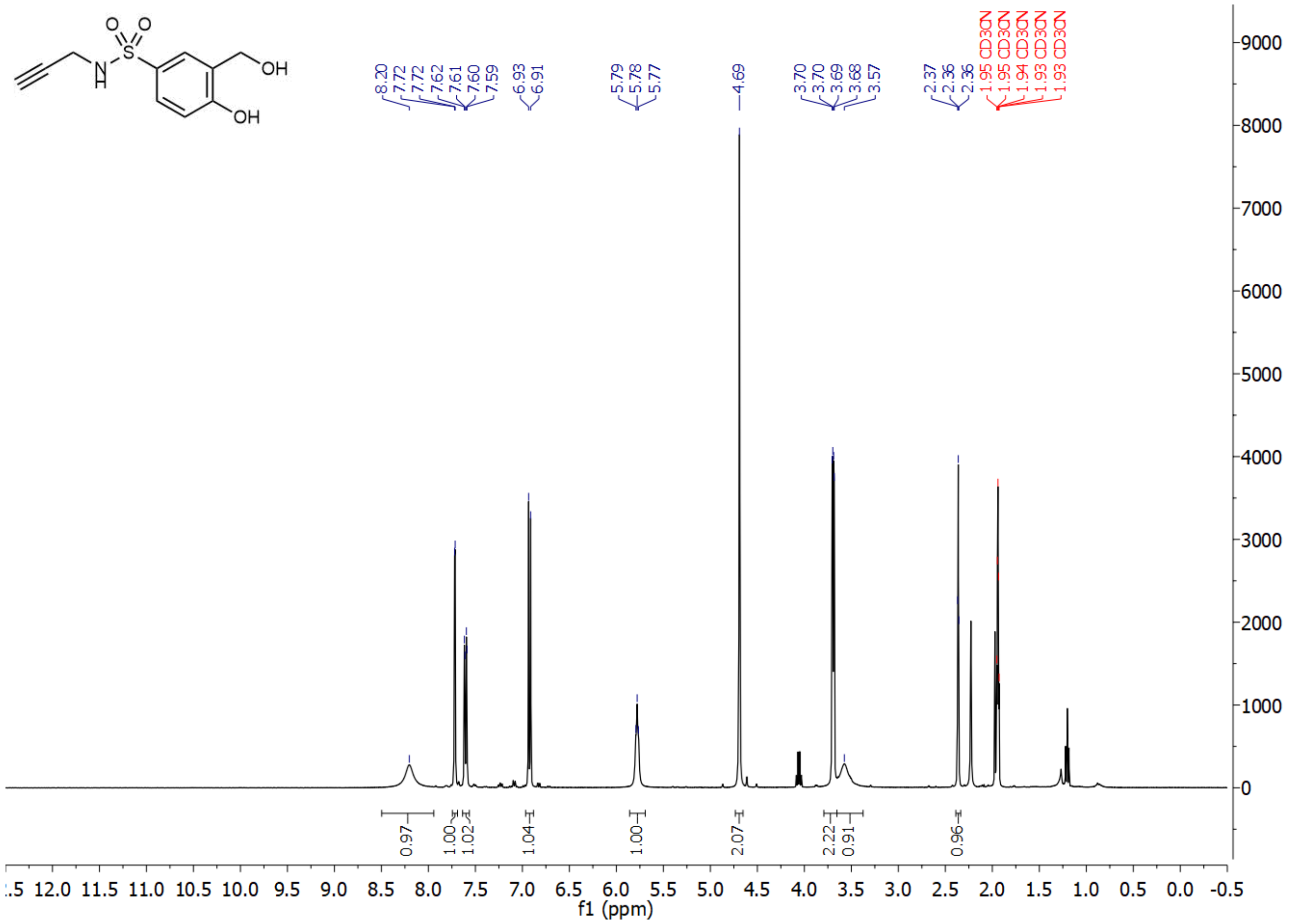

4-Hydroxy-3-(hydroxymethyl)-*N*-(prop-2-yn-1-yl)benzenesulfonamide (**HMP-alkyne**) (^{13}C, 101 MHz, CD_3CN):

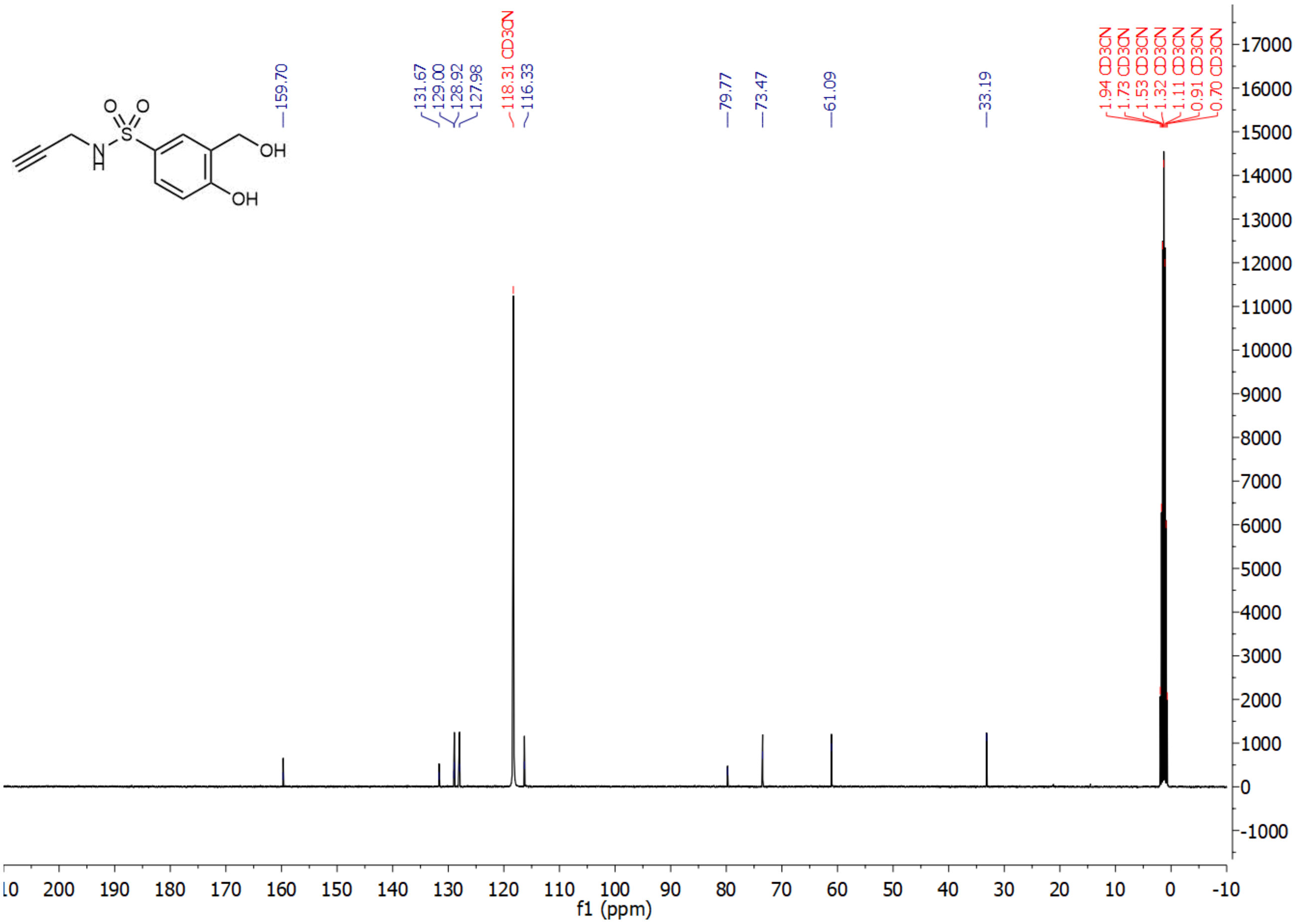

4-Hydroxy-3-(methoxymethyl)-*N*-(prop-2-yn-1-yl)benzenesulfonamide (**MMP-alkyne**) (^{1}H, 400 MHz, CD_3CN):

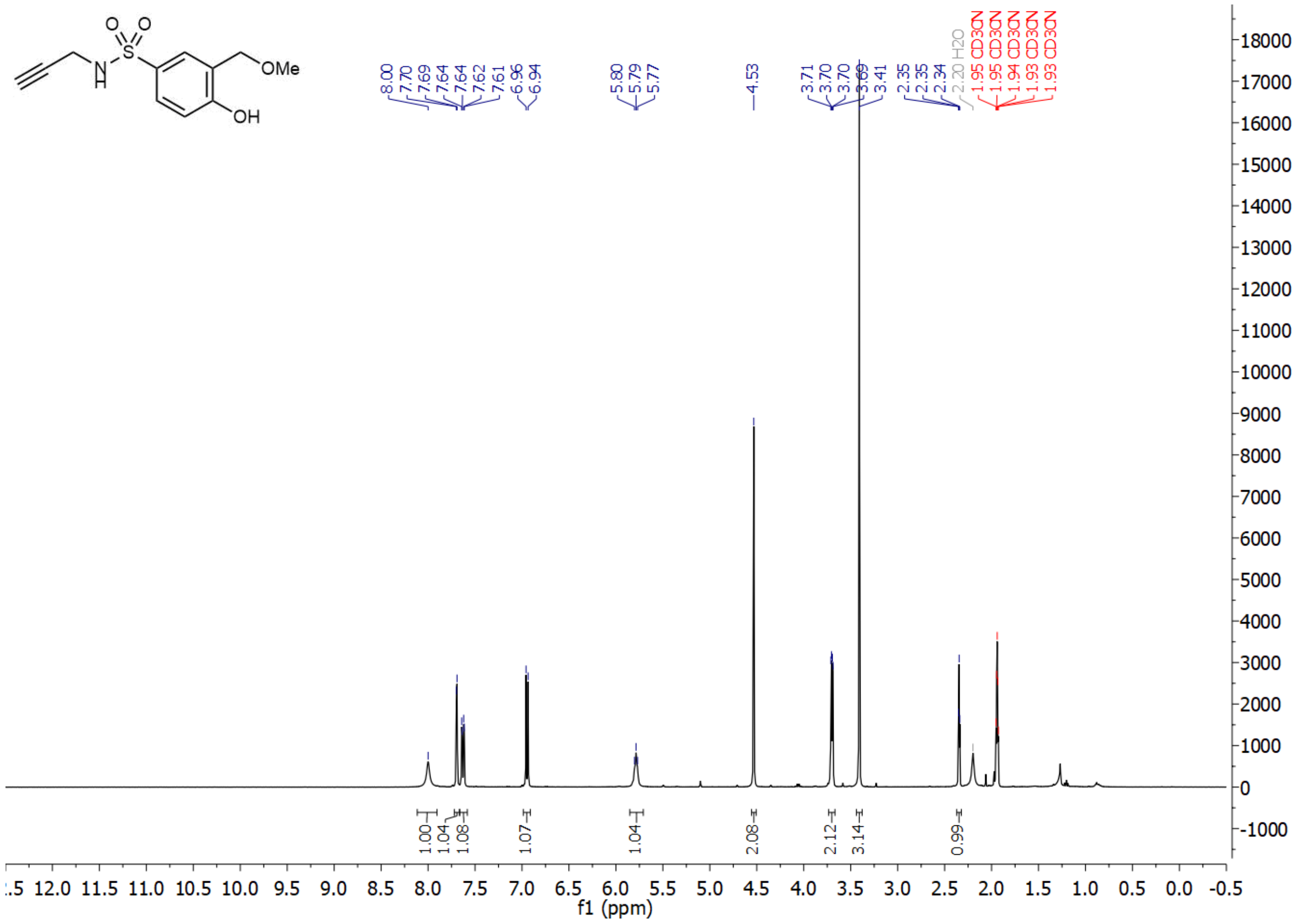

4-Hydroxy-3-(methoxymethyl)-*N*-(prop-2-yn-1-yl)benzenesulfonamide (**MMP-alkyne**) (^{13}C, 101 MHz, CD_3CN):

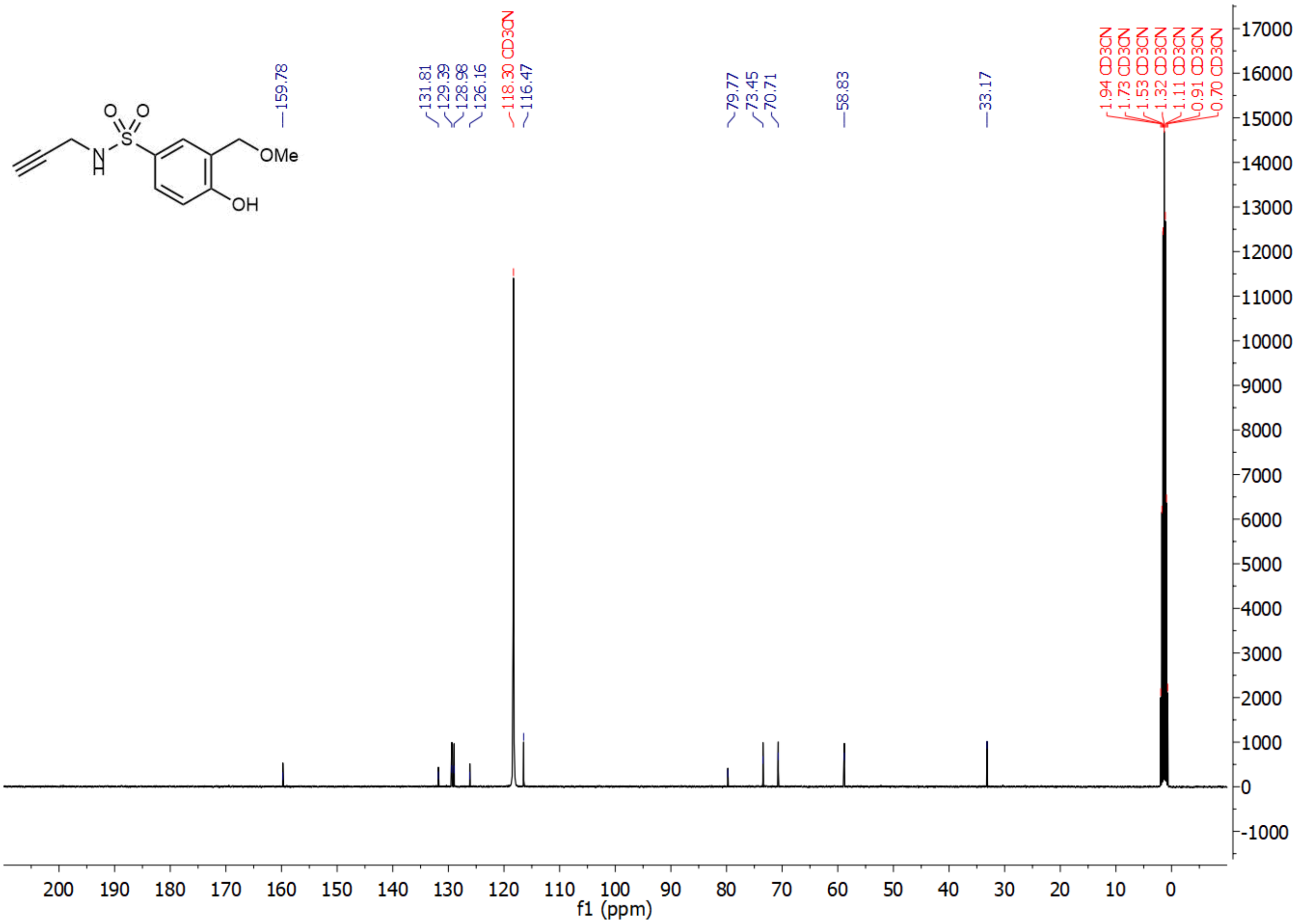

2,2-Dihydroxy-1-(4-(prop-2-yn-1-yloxy)phenyl)ethan-1-one (**PhGO-alkyne**) (^{1}H, 500 MHz, CD_3CN):

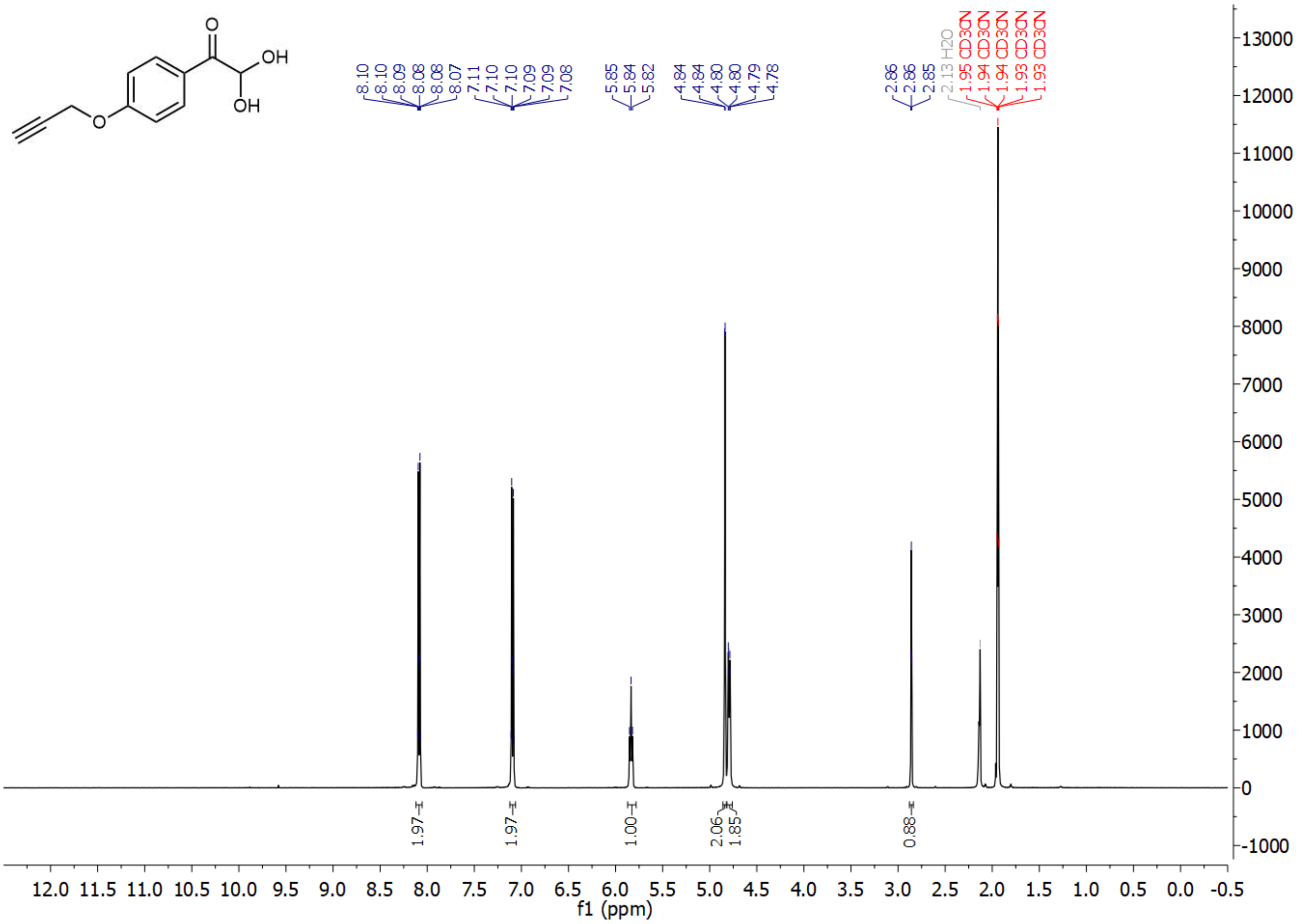

2,2-Dihydroxy-1-(4-(prop-2-yn-1-yloxy)phenyl)ethan-1-one (**PhGO-alkyne**) (^{13}C, 101 MHz, $CD_3CN:D_2O$ 1:1):

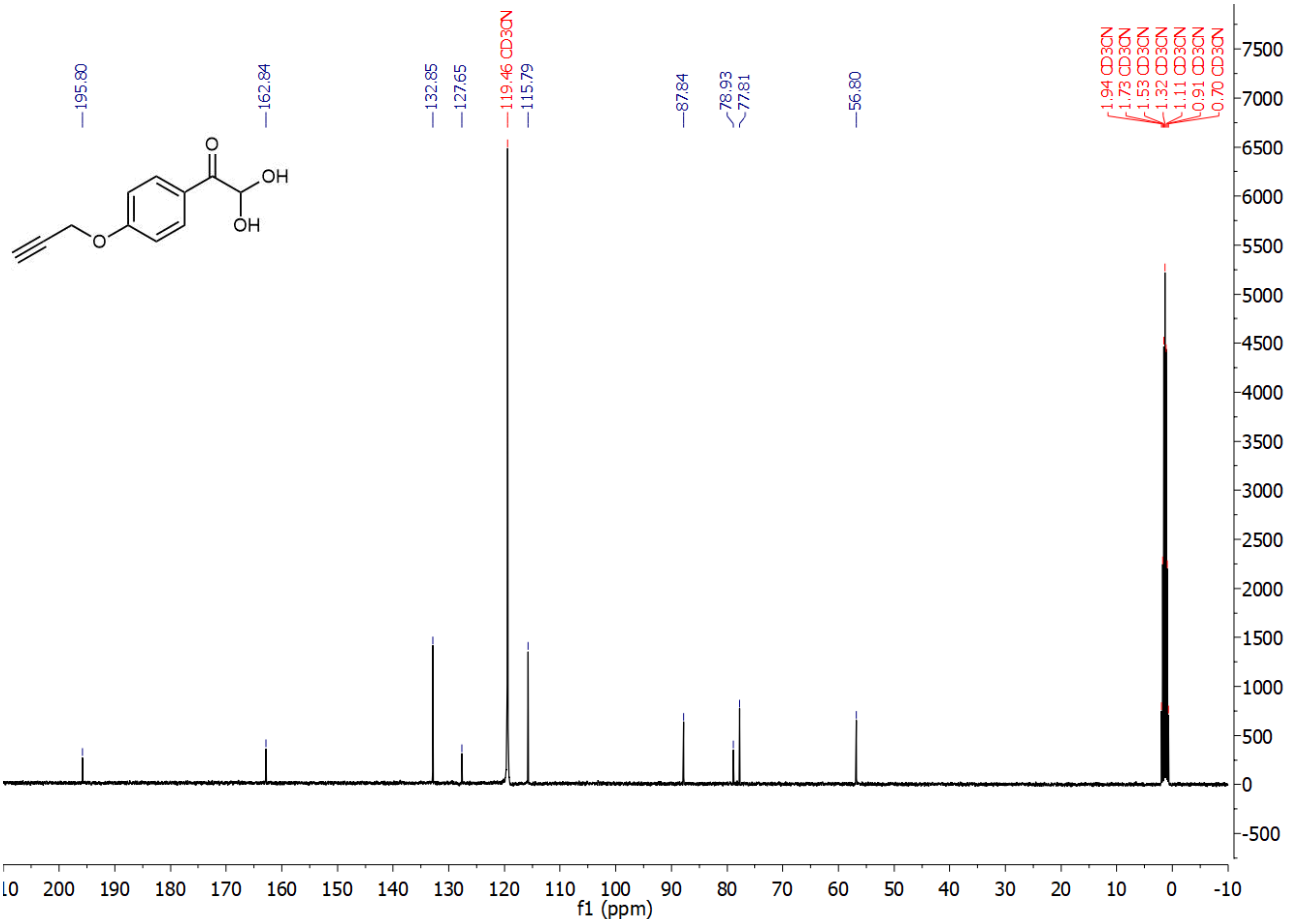

Ethyl hex-5-yn-1-ylfluorophosphonate (**FP-alkyne**) (^{1}H, 400 MHz, $CDCl_3$):

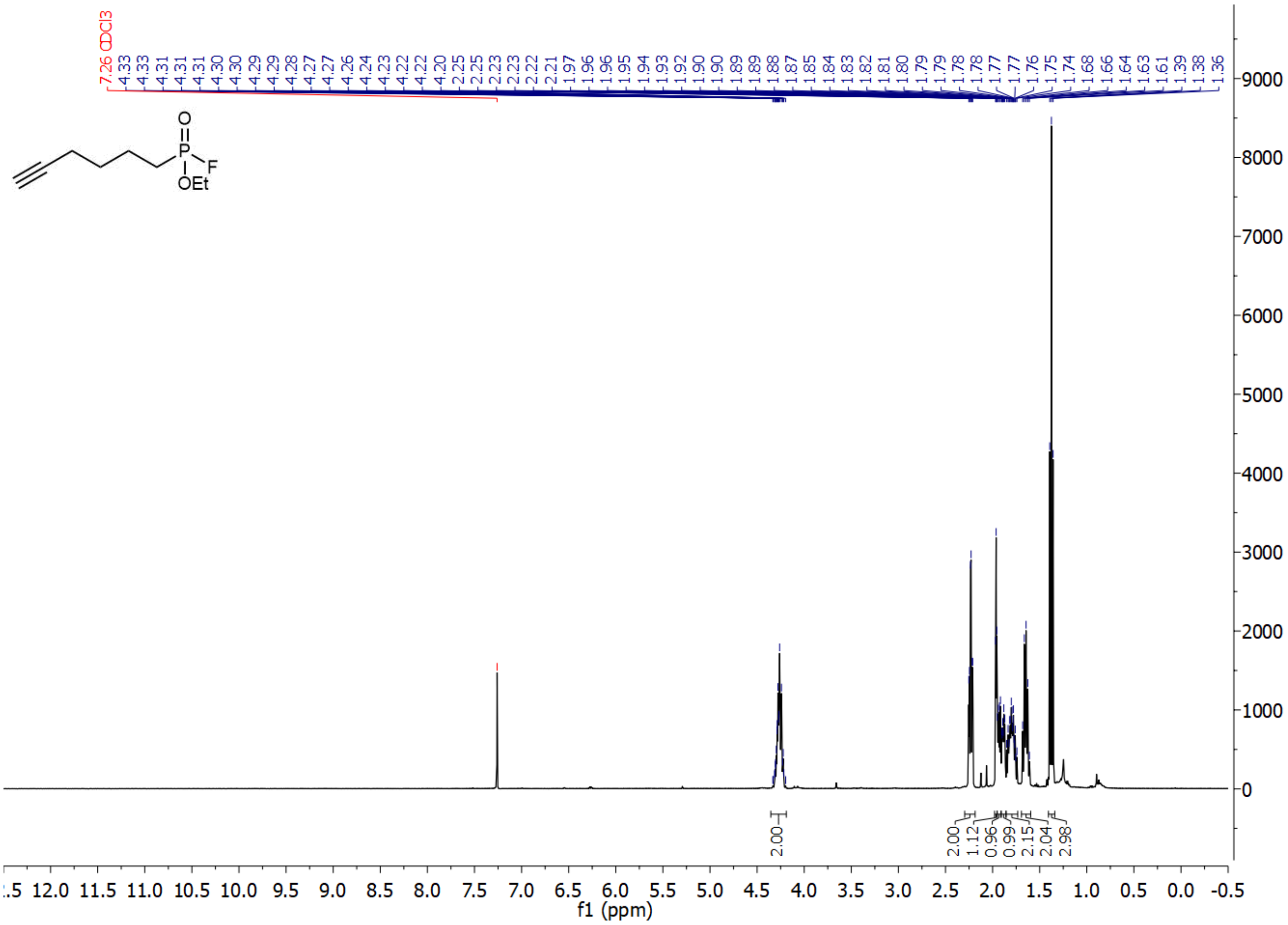

Ethyl hex-5-yn-1-ylfluorophosphonate (**FP-alkyne**) (^{13}C, 101 MHz, $CDCl_3$):

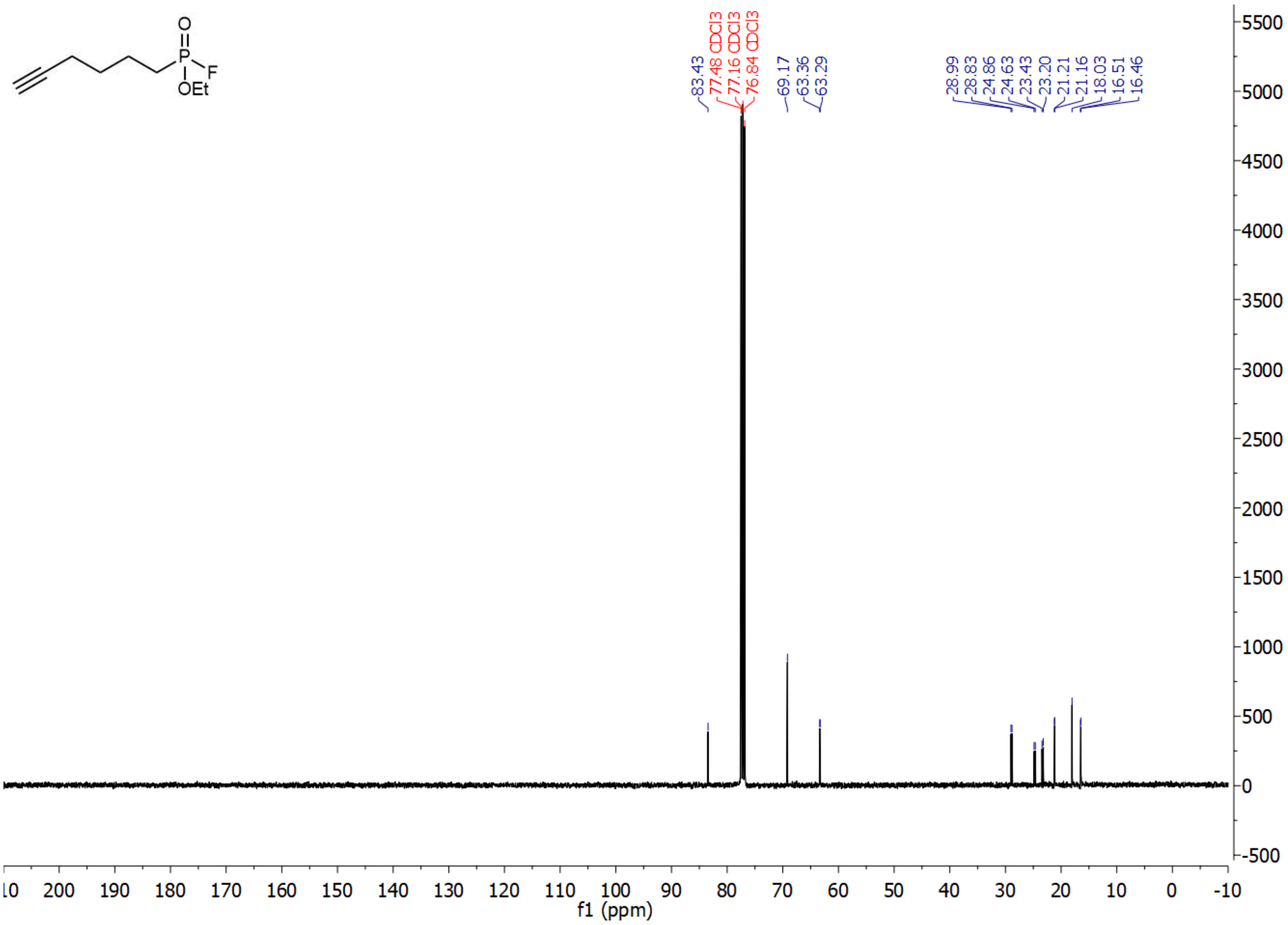

Ethyl hex-5-yn-1-ylfluorophosphonate (**FP-alkyne**) (^{19}F, 376 MHz, $CDCl_3$):

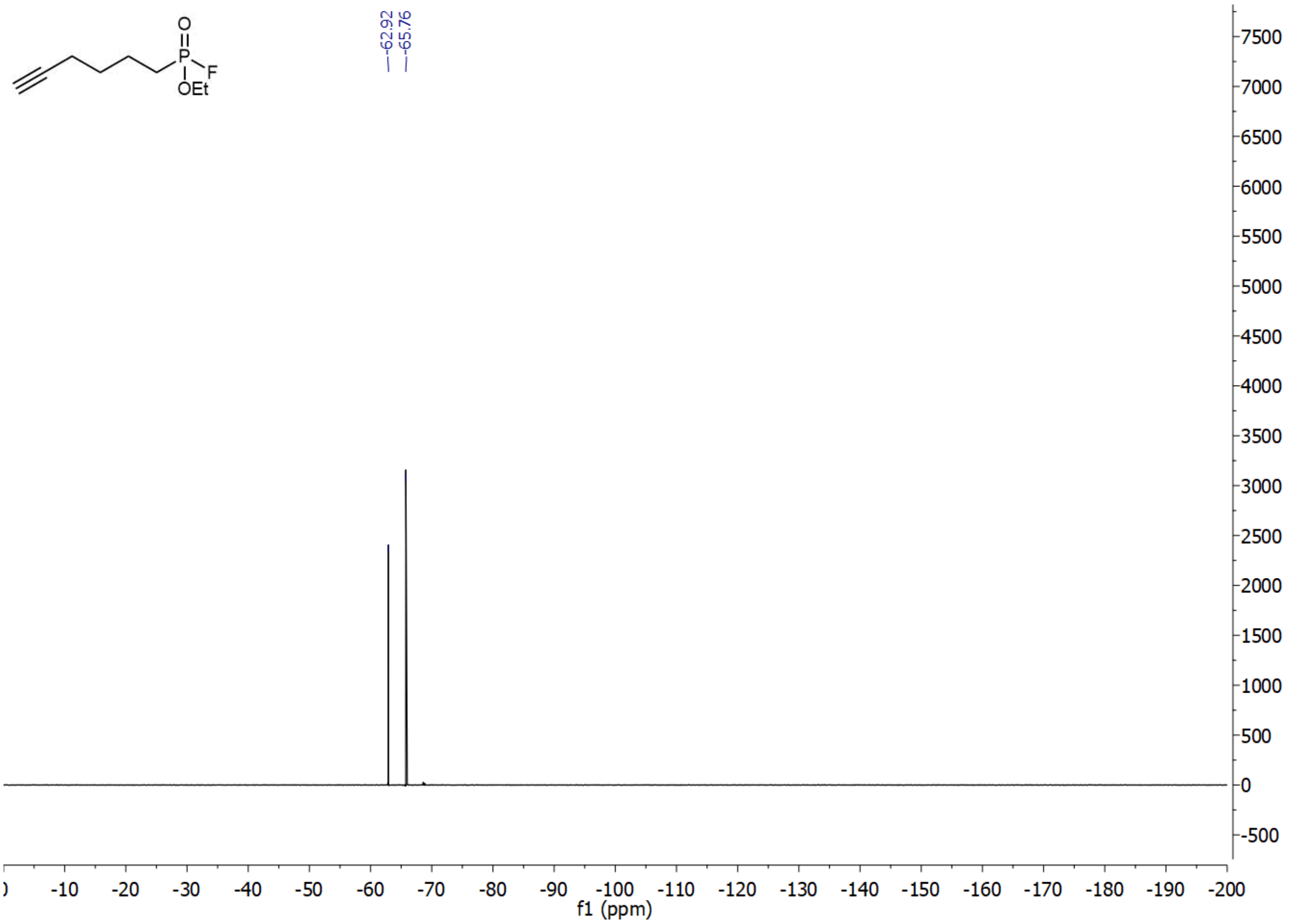

Ethyl hex-5-yn-1-ylfluorophosphonate (**FP-alkyne**) (^{31}P, 162 MHz, $CDCl_3$):

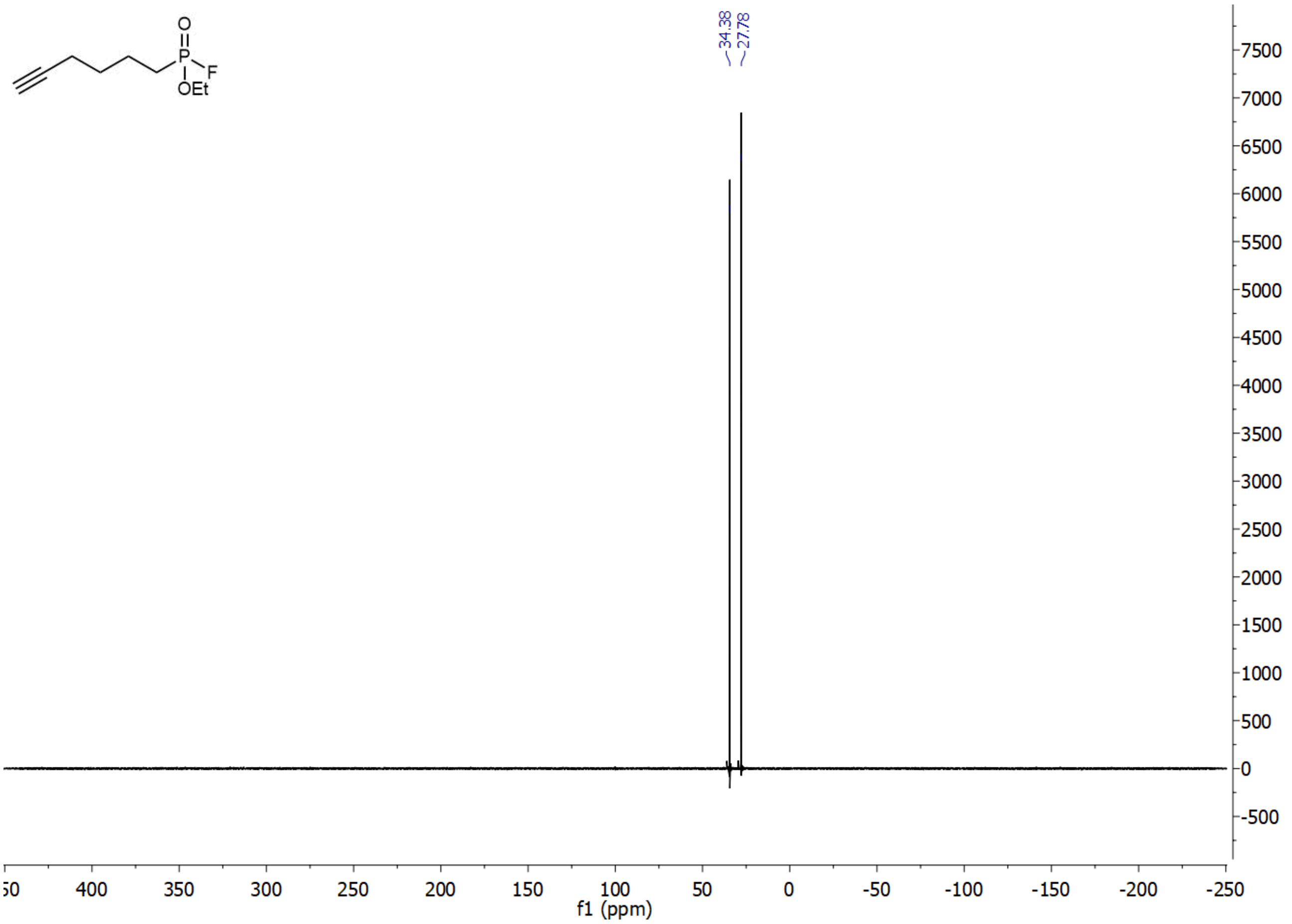

References

1. He J-X, Fei Z-C, Fu L, Tian C-P, He F-C, Chi H, Yang J. A modification-centric assessment tool for the performance of chemoproteomic probes. *Nat Chem Biol* 2022, **18**(8)**:** 904-912.
2. Mons E, Kim RQ, van Doodewaerd BR, van Veelen PA, Mulder MPC, Ovaa H. Exploring the Versatility of the Covalent Thiol–Alkyne Reaction with Substituted Propargyl Warheads: A Deciding Role for the Cysteine Protease. *J Am Chem Soc* 2021, **143**(17)**:** 6423-6433.
3. Reszka KJ, Chignell CF. One-electron reduction of arenediazonium compounds by physiological electron donors generates aryl radicals. An EPR and spin trapping investigation. *Chem-Biol Interact* 1995, **96**(3)**:** 223-234.
4. Nothling MD, Cao H, McKenzie TG, Hocking DM, Strugnell RA, Qiao GG. Bacterial Redox Potential Powers Controlled Radical Polymerization. *J Am Chem Soc* 2021, **143**(1)**:** 286-293.
5. Naveen N, Sengupta S, Chandrasekaran S. Metal-Free S-Arylation of Cysteine Using Arenediazonium Salts. *J Org Chem* 2018, **83**(7)**:** 3562-3569.
6. Fehler SK, Pratsch G, Östreicher C, Fürst MCD, Pischetsrieder M, Heinrich MR. Radical arylation of tyrosine residues in peptides. *Tetrahedron* 2016, **72**(48)**:** 7888-7893.
7. Song X, Ren X, Mei Q, Liu H, Huang H. Advancing In-Depth N-Terminomics Detection with a Cleavable 2-Pyridinecarboxyaldehyde Probe. *J Am Chem Soc* 2024, **146**(10)**:** 6487-6492.
8. Bridge HN, Leiter W, Frazier CL, Weeks AM. An N terminomics toolbox combining 2-pyridinecarboxaldehyde probes and click chemistry for profiling protease specificity. *Cell Chem Biol* 2024, **31**(3)**:** 534-549.e538.
9. Meyer B, Chiaravalli J, Gellenoncourt S, Brownridge P, Bryne DP, Daly LA*, et al.* Characterising proteolysis during SARS-CoV-2 infection identifies viral cleavage sites and cellular targets with therapeutic potential. *Nat Commun* 2021, **12**(1)**:** 5553.
10. Zanon PRA, Lewald L, Hacker SM. Isotopically Labeled Desthiobiotin Azide (isoDTB) Tags Enable Global Profiling of the Bacterial Cysteinome. *Angew Chem Int Ed* 2020, **59:** 2829-2836.
11. Kong AT, Leprevost FV, Avtonomov DM, Mellacheruvu D, Nesvizhskii AI. MSFragger: ultrafast and comprehensive peptide identification in mass spectrometry-based proteomics. *Nat Methods* 2017, **14**(5)**:** 513-520.
12. Yu F, Teo GC, Kong AT, Haynes SE, Avtonomov DM, Geiszler DJ, Nesvizhskii AI. Identification of modified peptides using localization-aware open search. *Nat Commun* 2020, **11**(1)**:** 4065.
13. Yu F, Haynes SE, Teo GC, Avtonomov DM, Polasky DA, Nesvizhskii AI. Fast Quantitative Analysis of timsTOF PASEF Data with MSFragger and IonQuant. *Mol Cell Proteom* 2020, **19**(9)**:** 1575-1585.
14. Cox J, Mann M. MaxQuant enables high peptide identification rates, individualized p.p.b.-range mass accuracies and proteome-wide protein quantification. *Nat Biotechnol* 2008, **26**(12)**:** 1367-1372.
15. Chi H, Liu C, Yang H, Zeng W-F, Wu L, Zhou W-J*, et al.* Comprehensive identification of peptides in tandem mass spectra using an efficient open search engine. *Nat Biotechnol* 2018, **36**(11)**:** 1059-1061.
16. Fu L, Li Z, Liu K, Tian C, He J, He J*, et al.* A quantitative thiol reactivity profiling platform to analyze redox and electrophile reactive cysteine proteomes. *Nat Protoc* 2020, **15**(9)**:** 2891-2919.
17. O'Shea JP, Chou MF, Quader SA, Ryan JK, Church GM, Schwartz D. pLogo: a probabilistic approach to visualizing sequence motifs. *Nat Methods* 2013, **10**(12)**:** 1211-1212.
18. Frei R, Wodrich MD, Hari DP, Borin P-A, Chauvier C, Waser J. Fast and Highly Chemoselective Alkynylation of Thiols with Hypervalent Iodine Reagents Enabled through a Low Energy Barrier Concerted Mechanism. *J Am Chem Soc* 2014, **136**(47)**:** 16563-16573.
19. Tessier R, Ceballos J, Guidotti N, Simonet-Davin R, Fierz B, Waser J. “Doubly Orthogonal” Labeling of Peptides and Proteins. *Chem* 2019, **5**(8)**:** 2243-2263.
20. Liu B, Alegre-Requena JV, Paton RS, Miyake GM. Unconventional Reactivity of Ethynylbenziodoxolone Reagents and Thiols: Scope and Mechanism. *Chem Eur J* 2020, **26**(11)**:** 2386-2394.
21. Liu B, Lim C-H, Miyake GM. Light-Driven Intermolecular Charge Transfer Induced Reactivity of Ethynylbenziodoxol(on)e and Phenols. *J Am Chem Soc* 2018, **140**(40)**:** 12829-12835.
22. Tessier R, Nandi RK, Dwyer BG, Abegg D, Sornay C, Ceballos J*, et al.* Ethynylation of Cysteine Residues: From Peptides to Proteins in Vitro and in Living Cells. *Angew Chem Int Ed* 2020, **59**(27)**:** 10961-10970.
23. Boyatzis AE, Bringans SD, Piggott MJ, Duong MN, Lipscombe RJ, Arthur PG. Limiting the Hydrolysis and Oxidation of Maleimide–Peptide Adducts Improves Detection of Protein Thiol Oxidation. *J Proteome Res* 2017, **16**(5)**:** 2004-2015.

24. Deng J-R, Lai NC-H, Kung KK-Y, Yang B, Chung S-F, Leung AS-L*, et al.* N-Terminal selective modification of peptides and proteins using 2-ethynylbenzaldehydes. *Commun Chem* 2020, **3**(1)**:** 67.
25. Zhu JS, Kraemer N, Li CJ, Haddadin MJ, Kurth MJ. Photochemical Preparation of 1,2-Dihydro-3H-indazol-3-ones in Aqueous Solvent at Room Temperature. *J Org Chem* 2018, **83**(24)**:** 15493-15498.
26. Onoda A, Inoue N, Sumiyoshi E, Hayashi T. Triazolecarbaldehyde Reagents for One-Step N-Terminal Protein Modification. *ChemBioChem* 2020, **21**(9)**:** 1274-1278.
27. MacDonald JI, Munch HK, Moore T, Francis MB. One-step site-specific modification of native proteins with 2-pyridinecarboxyaldehydes. *Nat Chem Bio* 2015, **11**(5)**:** 326-331.
28. Meier H, Heimgartner H. Intramolekulare 1,3-dipolare Cycloadditionen von Diarylnitriliminen aus 2,5-Diaryltetrazolen. *Helv Chim Acta* 1985, **68**(5)**:** 1283-1300.
29. Hegarty AF, Cashman MP, Scott FL. The kinetics of nitrilimine formation in base-catalysed hydrolysis of hydrazonyl halides. *J Chem Soc, Perkin Trans 2* 1972(1)**:** 44-52.
30. Woodward RB, Olofson RA. The reaction of isoxazolium salts with nucleophiles. *Tetrahedron* 1966, **22:** 415-440.
31. Black D, Doyle J. Direct conversion of α-halo acids into α-halo amides by reaction with 2-phenylazirine. *Aust J Chem* 1978, **31**(10)**:** 2313-2315.
32. Kaiser D, Winne JM, Ortiz-Soto ME, Seibel J, Le TA, Engels B. Mechanistical Insights into the Bioconjugation Reaction of Triazolinediones with Tyrosine. *J Org Chem* 2018, **83**(17)**:** 10248-10260.
33. Ban H, Nagano M, Gavrilyuk J, Hakamata W, Inokuma T, Barbas CF. Facile and Stabile Linkages through Tyrosine: Bioconjugation Strategies with the Tyrosine-Click Reaction. *Bioconjug Chem* 2013, **24**(4)**:** 520-532.
34. Koziakov D, Wu G, Jacobi von Wangelin A. Aromatic substitutions of arenediazonium salts via metal catalysis, single electron transfer, and weak base mediation. *Org Biomol Chem* 2018, **16**(27)**:** 4942-4953.
35. Lin S, Yang X, Jia S, Weeks AM, Hornsby M, Lee PS*, et al.* Redox-based reagents for chemoselective methionine bioconjugation. *Science* 2017, **355**(6325)**:** 597-602.
36. Tower SJ, Hetcher WJ, Myers TE, Kuehl NJ, Taylor MT. Selective Modification of Tryptophan Residues in Peptides and Proteins Using a Biomimetic Electron Transfer Process. *J Am Chem Soc* 2020, **142**(20)**:** 9112-9118.
37. Lin C, Du H-J, Zhao H, Yan D-F, Liu N-X, Sun H*, et al.* A formal intermolecular [4 + 2] cycloaddition reaction of 1,3-disubstituted indoles and alkylquinones. *Org Biomol Chem* 2017, **15**(16)**:** 3472-3478.
38. Uyanik M, Nishioka K, Kondo R, Ishihara K. Chemoselective oxidative generation of ortho-quinone methides and tandem transformations. *Nat Chem* 2020, **12**(4)**:** 353-362.
39. Thompson DA, Ng R, Dawson PE. Arginine selective reagents for ligation to peptides and proteins. *J Pept Sci* 2016, **22**(5)**:** 311-319.
40. Vantourout JC, Adusumalli SR, Knouse KW, Flood DT, Ramirez A, Padial NM*, et al.* Serine-Selective Bioconjugation. *J Am Chem Soc* 2020, **142**(41)**:** 17236-17242.
41. Hacker SM, Backus KM, Lazear MR, Forli S, Correia BE, Cravatt BF. Global profiling of lysine reactivity and ligandability in the human proteome. *Nat Chem* 2017, **9**(12)**:** 1181-1190.
42. Bach K, Beerkens BLH, Zanon PRA, Hacker SM. Light-Activatable, 2,5-Disubstituted Tetrazoles for the Proteome-Wide Profiling of Aspartates and Glutamates in Living Bacteria. *ACS Cent Sci* 2020, **6:** 546-554.
43. Hahm HS, Toroitich EK, Borne AL, Brulet JW, Libby AH, Yuan K*, et al.* Global targeting of functional tyrosines using sulfur-triazole exchange chemistry. *Nat Chem Biol* 2019, **16:** 150-159.
44. Battenberg OA, Nodwell MB, Sieber SA. Evaluation of α-Pyrones and Pyrimidones as Photoaffinity Probes for Affinity-Based Protein Profiling. *J Org Chem* 2011, **76**(15)**:** 6075-6087.
45. Nagendra Reddy C, Ye Q, Patel P, Sivendran S, Chourey S, Wang R*, et al.* Design and synthesis of affinity chromatography ligands for the purification of 5-hydroxyeicosanoid dehydrogenase. *Bioorg Med Chem* 2017, **25**(1)**:** 116-125.
46. Wu J, Tang X. Synthesis and enzymatic incorporation of photolabile dUTP analogues into DNA and their applications for DNA labeling. *Bioorg Med Chem* 2013, **21**(20)**:** 6205-6211.
47. Barendt TA, Myers WK, Cornes SP, Lebedeva MA, Porfyrakis K, Marques I*, et al.* The Green Box: An Electronically Versatile Perylene Diimide Macrocyclic Host for Fullerenes. *J Am Chem Soc* 2020, **142**(1)**:** 349-364.
48. Backus KM, Correia BE, Lum KM, Forli S, Horning BD, Gonzalez-Paez GE*, et al.* Proteome-wide covalent ligand discovery in native biological systems. *Nature* 2016, **534**(7608)**:** 570-574.

49. Harris MR, Konev MO, Jarvo ER. Enantiospecific Intramolecular Heck Reactions of Secondary Benzylic Ethers. *J Am Chem Soc* 2014, **136**(22)**:** 7825-7828.
50. Ortiz-Soto ME, Ertl J, Mut J, Adelmann J, Le TA, Shan J*, et al.* Product-oriented chemical surface modification of a levansucrase (SacB) via an ene-type reaction. *Chem Sci* 2018, **9**(24)**:** 5312-5321.
51. Motiwala HF, Kuo Y-H, Stinger BL, Palfey BA, Martin BR. Tunable Heteroaromatic Sulfones Enhance in-Cell Cysteine Profiling. *J Am Chem Soc* 2020, **142**(4)**:** 1801-1810.
52. Toda N, Asano S, Barbas Iii CF. Rapid, Stable, Chemoselective Labeling of Thiols with Julia–Kocieński-like Reagents: A Serum-Stable Alternative to Maleimide-Based Protein Conjugation. *Angew Chem Int Ed* 2013, **52**(48)**:** 12592-12596.
53. Moses JE, Ritson DJ, Zhang F, Lombardo CM, Haider S, Oldham N, Neidle S. A click chemistry approach to C3 symmetric, G-quadruplex stabilising ligands. *Org Biomol Chem* 2010, **8**(13)**:** 2926-2930.
54. Fan B, Trant JF, Wong AD, Gillies ER. Polyglyoxylates: A Versatile Class of Triggerable Self-Immolative Polymers from Readily Accessible Monomers. *J Am Chem Soc* 2014, **136**(28)**:** 10116-10123.
55. Guo A-D, Wei D, Nie H-J, Hu H, Peng C, Li S-T*, et al.* Light-induced primary amines and o-nitrobenzyl alcohols cyclization as a versatile photoclick reaction for modular conjugation. *Nat Commun* 2020, **11**(1)**:** 5472.
56. Kwok SW, Fotsing JR, Fraser RJ, Rodionov VO, Fokin VV. Transition-Metal-Free Catalytic Synthesis of 1,5-Diaryl-1,2,3-triazoles. *Org Lett* 2010, **12**(19)**:** 4217-4219.
57. Fletcher JT, Christensen JA, Villa EM. Tandem synthesis of 1-formyl-1,2,3-triazoles. *Tet Lett* 2017, **58**(47)**:** 4450-4454.
58. Qian Y, Schurmann M, Janning P, Hedberg C, Waldmann H. Activity-Based Proteome Profiling Probes Based on Woodward's Reagent K with Distinct Target Selectivity. *Angew Chem Int Ed* 2016, **55**(27)**:** 7766-7771.
59. Muralidharan S, Wang C, inventors; Trans-1,2-diphenylethylene derivatives and nanosensors made therefrom. United States patent 20100112545. 2010.
60. Ma N, Hu J, Zhang Z-M, Liu W, Huang M, Fan Y*, et al.* 2H-Azirine-Based Reagents for Chemoselective Bioconjugation at Carboxyl Residues Inside Live Cells. *J Am Chem Soc* 2020, **142**(13)**:** 6051-6059.
61. Wang R, Xu J. Selective alkylation of aminophenols. *Arkivoc* 2010**:** 293-299.
62. da Silva CM, da Silva DL, Martins CVB, de Resende MA, Dias ES, Magalhães TFF*, et al.* Synthesis of Aryl Aldimines and Their Activity against Fungi of Clinical Interest. *Chem Biol Drug Des* 2011, **78**(5)**:** 810-815.
63. Li Z, Huang R, Xu H, Chen J, Zhan Y, Zhou X*, et al.* Divinylsulfonamides as Specific Linkers for Stapling Disulfide Bonds in Peptides. *Org Lett* 2017, **19**(18)**:** 4972-4975.
64. Naik A, Alzeer J, Triemer T, Bujalska A, Luedtke NW. Chemoselective Modification of Vinyl DNA by Triazolinediones. *Angew Chem Int Ed* 2017, **56**(36)**:** 10850-10853.
65. Kekec A, Kevin NJ, Li B, Lin S, Parish CA, Tang W, inventors; Merck Sharp & Dohme Corp. (Rahway, NJ, US), assignee. Tyrosine-specific functionalized insulin and insulin analogs. United States patent 20210002345. 2021.
66. Ramanathan M, Liu S-T. Cascade annulations of aryldiazonium salts, nitriles and halo-alkynes leading to 3-haloquinolines. *Tetrahedron* 2017, **73**(30)**:** 4317-4322.
67. Evrard D, Lambert F, Policar C, Balland V, Limoges B. Electrochemical Functionalization of Carbon Surfaces by Aromatic Azide or Alkyne Molecules: A Versatile Platform for Click Chemistry. *Chem Eur J* 2008, **14**(30)**:** 9286-9291.
68. Jiang C-s, Wang X-m, Zhang S-q, Meng L-s, Zhu W-h, Xu J, Lu S-m. Discovery of 4-benzoylamino-N-(prop-2-yn-1-yl)benzamides as novel microRNA-21 inhibitors. *Bioorg Med Chem* 2015, **23**(19)**:** 6510-6519.
69. Senadi GC, Guo B-C, Hu W-P, Wang J-J. Iodine-promoted cyclization of N-propynyl amides and N-allyl amides via sulfonylation and sulfenylation. *Chem Commun* 2016, **52**(76)**:** 11410-11413.
70. Rayner PJ, O'Brien P, Horan RAJ. Preparation and Reactions of Enantiomerically Pure α-Functionalized Grignard Reagents. *J Am Chem Soc* 2013, **135**(21)**:** 8071-8077.
71. Mainolfi N, Ji N, Kluge AF, Weiss MM, Zhang Y, inventors; Kymera Therapeutics, Inc. (Cambridge, MA, US), assignee. IRAK degraders and uses thereof. United States patent 20190192668. 2019.
72. Minatti AE, Low JD, Allen JR, Chen J, Chen N, Cheng Y*, et al.*, inventors; AMGEN INC., assignee. Perfluorinated 5,6-dihydro-4H-1,3-oxazin-2-amine compounds as beta-secretase inhibitors and methods of use. United States patent 20140249104. 2014.
73. Zhao T, Grützke M, Götz KH, Druzhenko T, Huhn T. Synthesis and X-ray structure analysis of cytotoxic heptacoordinate sulfonamide salan titanium(iv)-bis-chelates. *Dalton Trans* 2015, **44**(37)**:** 16475-16485.

74. Ji H, Liu T, Mao LN, Zhao L, inventors; 1,2,3-Triazole-flavonoid compound-sophocarpidine ternary conjugate and use. Chinese patent CN104387389B. 2016.
75. Yu B, Qi P-P, Shi X-J, Huang R, Guo H, Zheng Y-C*, et al.* Efficient synthesis of new antiproliferative steroidal hybrids using the molecular hybridization approach. *Eur J Med Chem* 2016, **117:** 241-255.
76. Bomal E, Croué V, Yeo R, Scopelliti R, Frauenrath H. Hexayne Amphiphiles and Bolaamphiphiles. *Chem Eur J* 2020, **26**(41)**:** 8907-8915.
77. Kessner D, Chambers M, Burke R, Agus D, Mallick P. ProteoWizard: open source software for rapid proteomics tools development. *Bioinformatics* 2008, **24**(21)**:** 2534-2536.
78. da Veiga Leprevost F, Haynes SE, Avtonomov DM, Chang H-Y, Shanmugam AK, Mellacheruvu D*, et al.* Philosopher: a versatile toolkit for shotgun proteomics data analysis. *Nat Methods* 2020, **17**(9)**:** 869-870.
79. O'Neill AJ. Staphylococcus aureus SH1000 and 8325-4: comparative genome sequences of key laboratory strains in staphylococcal research. *Lett Appl Microbiol* 2010, **51**(3)**:** 358-361.
80. Chaudhuri RR, Allen AG, Owen PJ, Shalom G, Stone K, Harrison M*, et al.* Comprehensive identification of essential Staphylococcus aureus genes using Transposon-Mediated Differential Hybridisation (TMDH). *BMC Genom* 2009, **10:** 291.
81. Perez-Riverol Y, Csordas A, Bai J, Bernal-Llinares M, Hewapathirana S, Kundu DJ*, et al.* The PRIDE database and related tools and resources in 2019: improving support for quantification data. *Nucleic Acids Res* 2019, **47**(D1)**:** D442-D450.
