# Supplementary material for: Profiling the proteome-wide selectivity of diverse electrophiles
Source: Nat Chem. 2025 Oct 30;17(11):1712–21. doi: 10.1038/s41557-025-01902-z (PMC12580327; doi:10.1038/s41557-025-01902-z)
Supplement: Supplementary file 2 — Reporting Summary [file 41557_2025_1902_MOESM2_ESM.pdf]

nature research

Corresponding author(s): Stephan M. Hacker

Last updated by author(s): Jul 6, 2025

Reporting Summary

Nature Research wishes to improve the reproducibility of the work that we publish. This form provides structure for consistency and transparency in reporting. For further information on Nature Research policies, see our Editorial Policies and the Editorial Policy Checklist.

Statistics

For all statistical analyses, confirm that the following items are present in the figure legend, table legend, main text, or Methods section.

n/a	Confirmed	
☐	☒	The exact sample size (n) for each experimental group/condition, given as a discrete number and unit of measurement
☐	☒	A statement on whether measurements were taken from distinct samples or whether the same sample was measured repeatedly
☐	☒	The statistical test(s) used AND whether they are one- or two-sided<br>*Only common tests should be described solely by name; describe more complex techniques in the Methods section.*
☒	☐	A description of all covariates tested
☒	☐	A description of any assumptions or corrections, such as tests of normality and adjustment for multiple comparisons
☒	☐	A full description of the statistical parameters including central tendency (e.g. means) or other basic estimates (e.g. regression coefficient) AND variation (e.g. standard deviation) or associated estimates of uncertainty (e.g. confidence intervals)
☐	☒	For null hypothesis testing, the test statistic (e.g. F, t, r) with confidence intervals, effect sizes, degrees of freedom and P value noted<br>*Give P values as exact values whenever suitable.*
☒	☐	For Bayesian analysis, information on the choice of priors and Markov chain Monte Carlo settings
☒	☐	For hierarchical and complex designs, identification of the appropriate level for tests and full reporting of outcomes
☒	☐	Estimates of effect sizes (e.g. Cohen's d, Pearson's r), indicating how they were calculated

Our web collection on statistics for biologists contains articles on many of the points above.

Software and code

Policy information about availability of computer code

Data collection	Proteomics data of the LC-MS/MS analyses was collected using the Thermo Scientific Xcalibur software (version 4.1).
Data analysis	Raw data of the LC-MS/MS analyses was converted into the mzML format using the MSconvert tool (version: 3.0.19172-57d620127) of the ProteoWizard software (version: 3.0.19172 64bit). For all data analysis using MSFragger-based FragPipe, the FragPipe interface (version: 14.0) was used with MSFragger (version: 3.1.1), Philosopher (version: 3.3.10), IonQuant (version 1.4.6) and Python (version: 3.7.3). For individual analyses, pFind3 (version 3.1.56) with downstream application of a custom script (available at https://github.com/morpheusliu/Post-processing-program-for-pFind3-results) using Python (version: 3.7.3) or MaxQuant (version 1.6.17.0) were used. pLOGOs were generated at the webserver interface (https://plogo.uconn.edu/).

For manuscripts utilizing custom algorithms or software that are central to the research but not yet described in published literature, software must be made available to editors and reviewers. We strongly encourage code deposition in a community repository (e.g. GitHub). See the Nature Research guidelines for submitting code & software for further information.

Data

Policy information about availability of data

All manuscripts must include a data availability statement. This statement should provide the following information, where applicable:

- Accession codes, unique identifiers, or web links for publicly available datasets
- A list of figures that have associated raw data
- A description of any restrictions on data availability

Mass spectrometric data for all proteomic analyses, the needed FASTA databases and accessory files for FragPipe analyses (workflows, modification list etc.) have been deposited to the ProteomeXchange Consortium (http://proteomecentral.proteomexchange.org) via the PRIDE partner repository with the dataset identifier

PXD024454 and PXD065811. Raw data for Fig. 2-6 is available in the accompanying Source Data files as well as in the Supplementary Tables 2-5. All other data is available in the main text or the Supplementary Information.

Field-specific reporting

Please select the one below that is the best fit for your research. If you are not sure, read the appropriate sections before making your selection.

- [x] Life sciences
- [ ] Behavioural & social sciences
- [ ] Ecological, evolutionary & environmental sciences

For a reference copy of the document with all sections, see nature.com/documents/nr-reporting-summary-flat.pdf

Life sciences study design

All studies must disclose on these points even when the disclosure is negative.

Sample size	No sample-size calculation was performed. As we were not planning to compare replicates for the probes with a statistical test and in line with best practices in the field, it was chosen to perform the proteomic analyses in technical duplicates.
Data exclusions	No data were excluded from the analyses.
Replication	All attempts at replication were successful. All experiments were performed in technical duplicates.
Randomization	No randomization of samples was performed. Samples were not randomized as no statistical analysis across the replicates was planned and as it was deemed unlikely that the order of sample preparation and measurement would have an influence on the outcome of the study.
Blinding	No blinding of samples was performed. Samples were not blinded as no statistical analysis across the replicates was planned and as it was deemed unlikely that the knowledge of the scientist, on which sample is which, during the sample preparation and measurement would have an influence on the outcome of the study.

Reporting for specific materials, systems and methods

We require information from authors about some types of materials, experimental systems and methods used in many studies. Here, indicate whether each material, system or method listed is relevant to your study. If you are not sure if a list item applies to your research, read the appropriate section before selecting a response.

Materials & experimental systems

n/a	Involved in the study	
☒	☐	Antibodies
☐	☒	Eukaryotic cell lines
☒	☐	Palaeontology and archaeology
☒	☐	Animals and other organisms
☒	☐	Human research participants
☒	☐	Clinical data
☒	☐	Dual use research of concern

Methods

n/a	Involved in the study	
☒	☐	ChIP-seq
☒	☐	Flow cytometry
☒	☐	MRI-based neuroimaging

Eukaryotic cell lines

Policy information about cell lines

Cell line source(s)	ATCC (MDA-MB-231, catalog number HTB-26)
Authentication	The MDA-MB-231 cell line was not authenticated.
Mycoplasma contamination	The MDA-MB-231 cell line was tested negative for mycoplasma contamination.
Commonly misidentified lines (See ICLAC register)	No commonly misidentified cell lines were used.
