# Supplementary material for: Profiling the proteome-wide selectivity of diverse electrophiles
Source: Nat Chem. 2025 Oct 30;17(11):1712–21. doi: 10.1038/s41557-025-01902-z (PMC12580327; doi:10.1038/s41557-025-01902-z)
Supplement: Supplementary file 3 — Overview of the chemical mechanisms of the electrophiles used. [file 41557_2025_1902_MOESM3_ESM.pdf]

Supplementary Table 1 | Chemical reactions for all probes used in this study

Probe(s)	Comment	Reaction
**IA-alkyne**<br>**CA-alkyne**<br>**CA-nitrile**<br>**BMK-alkyne**	Reaction of cysteines with haloacetamides and halomethylketones.	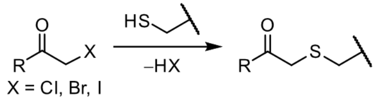
**PFPSA-alkyne**	Mechanism of cysteine-labelling using a nucleophilic aromatic substitution reaction with pentafluorophenyl sulfon-amides.	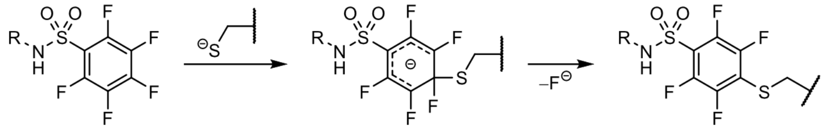
**BrBT-alkyne**<br>**MSBT-alkyne**<br>**MST-alkyne**<br>**MSOD-alkyne**	Mechanism of cysteine-labelling using a nucleophilic aromatic substitution reaction with different heteroaromatics.	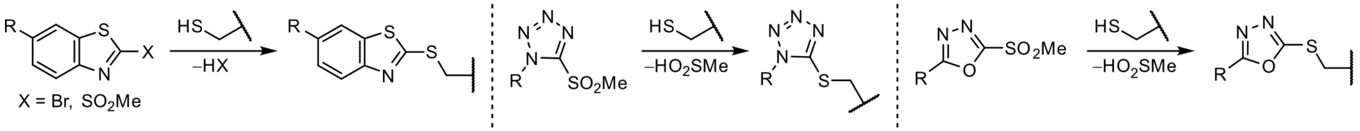
**EBX1-alkyne**	Mechanism of cysteine-labelling with hypervalent iodine reagent **EBX1-alkyne** as described by Frei *et al.*[1] After addition of cysteine, the vinylic carbanion can either undergo α-elimination followed by a 1,2-shift (**i**) to result in formal ethynylation or get protonated (**ii**) to produce a vinylbenziodoxolone (VBX),[2] which could potentially further react to the corresponding vinyl iodide.[3, 4]	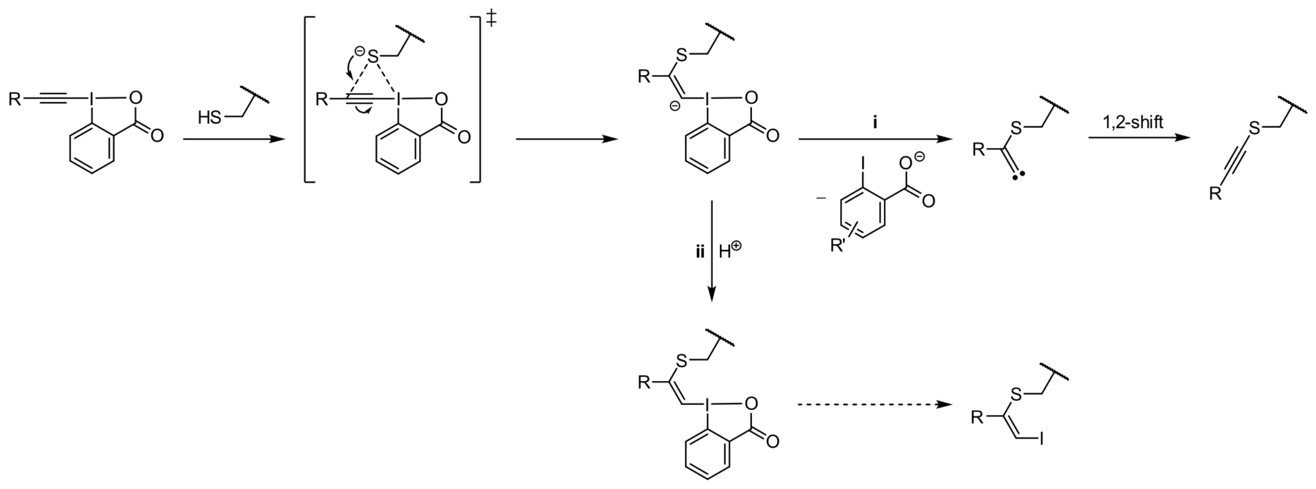

Supplementary Table 1 (continued)

Probe(s)	Comment	Reaction
**EBX2-alkyne**	Mechanism of cysteine-labelling with hypervalent iodine reagent **EBX2-alkyne** as described by Tessier *et al.*[5]	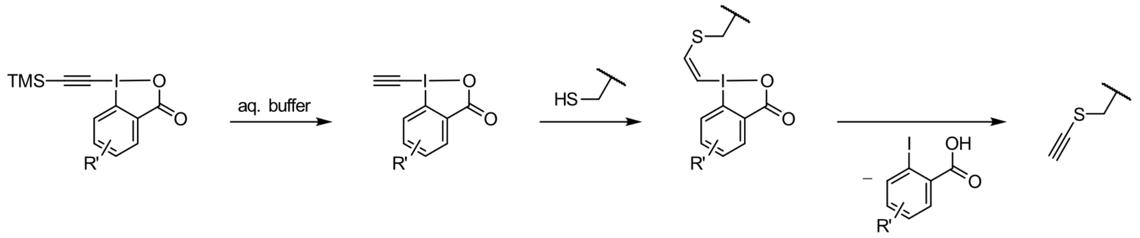
**Ep-alkyne**	Reaction of cysteines with epoxides.	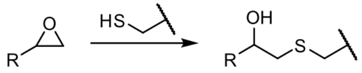
**Ts-alkyne**	Reaction of cysteines with alkyl tosylates.	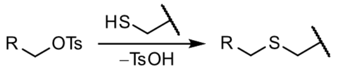
**MI-alkyne**	Reaction of cysteines with maleimides and hydrolysis of the formed adduct.[6]	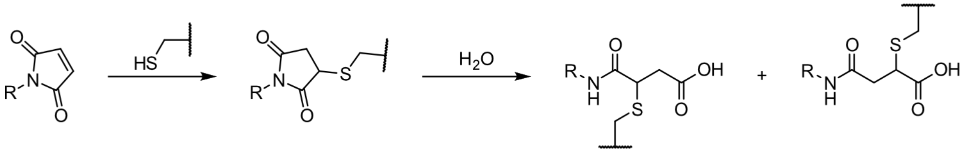
**AlkPA-alkyne**<br>**ArPA-alkyne**	Reaction of cysteines with propiolamides.	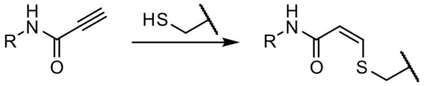
**AlkAA-alkyne**<br>**ArAA-alkyne**<br>**AlkFAA-alkyne**	Reaction of cysteines with acrylamides.	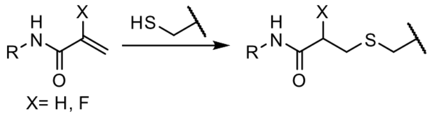
**AlkVS-alkyne**<br>**ArVS-alkyne**<br>**AlkVSA-alkyne**<br>**ArVSA-alkyne**	Reaction of cysteines with vinyl sulfones and vinyl sulfonamides.	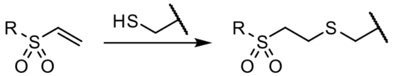
**STP-alkyne**<br>**TFP-alkyne**<br>**NHS-alkyne**<br>**ATT-alkyne**<br>**NASA-alkyne**	Reaction of lysines with activated esters.	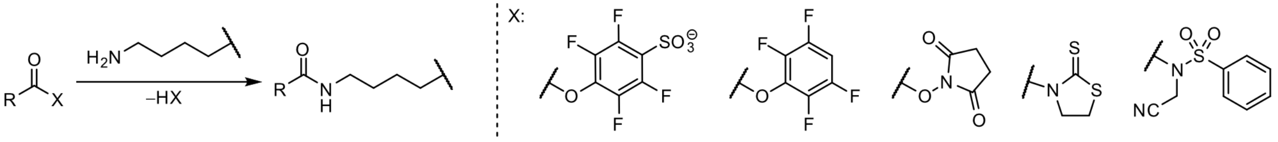
**AlkSq-alkyne**<br>**ArSq-alkyne**	Reaction of lysines with squaric acid monoester monoamides.	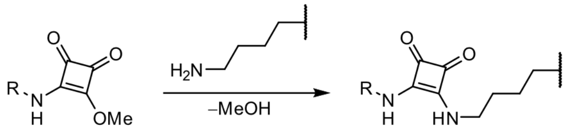

Supplementary Table 1 (continued)

Probe(s)	Comment	Reaction
**EBA-alkyne**	Mechanism of lysine-labelling with 2-ethynyl-benzaldehydes through a 6-endo-*dig* cyclisation of the formed imine as described by Deng *et al*.[7]	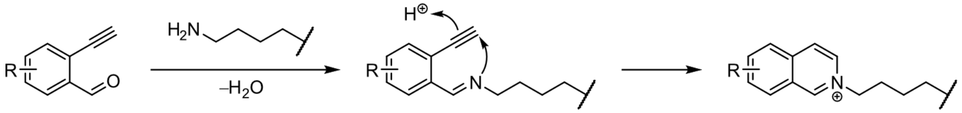
**oNBA-alkyne**	Mechanism of the photoinduced labelling of lysine with *ortho*-nitro-benzylalcohols (**oNBA alkyne**) as described by Zhu et al.[8]	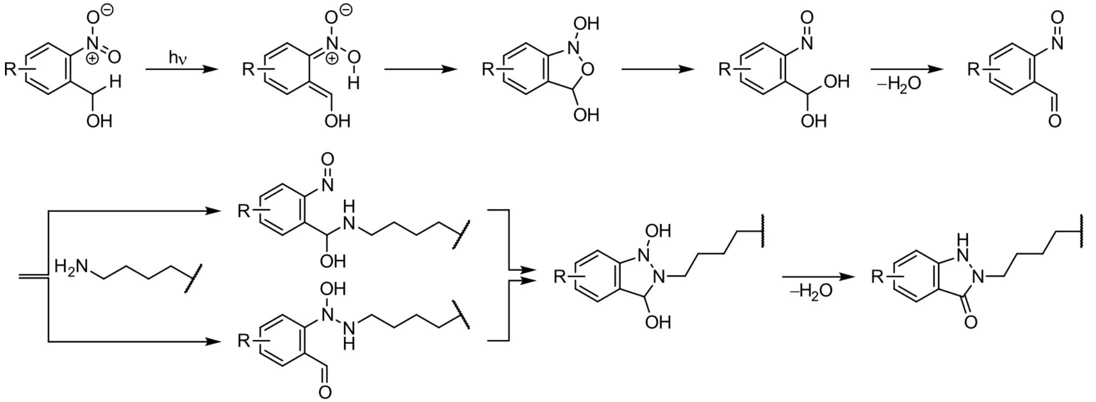
**TCA-alkyne**<br>**PCA-alkyne**	Mechanism of *N*-terminal labelling with hetero-aromatic aldehydes (**TCA-** and **PCA-alkyne**) as described by Onoda *et al.*[9] and MacDonald *et al.*,[10] respectively.	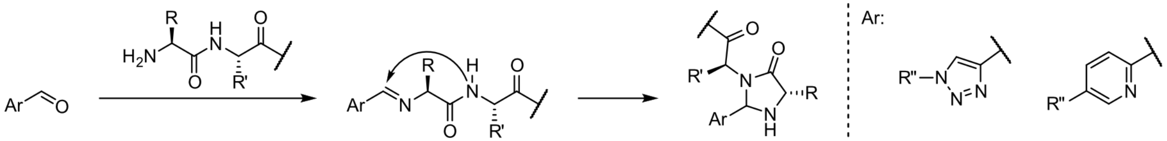

Supplementary Table 1 (continued)

Probe(s)	Comment	Reaction
**PhTet-alkyne**<br>**AmTet-alkyne**<br>**MeTet-alkyne**<br>**HC-alkyne**	Mechanism of labelling of carboxylic acids with *in situ* generated nitrilimines. These reactive inter-mediates can be generated from 2,5-disubstituted tetrazoles (**PhTet-**, **AmTet-** and **MeTet-alkyne**) *via* photolysis (**i**) as described by Meier and Heimgartner[11] or from hydrazonoyl chlorides (**HC-alkyne**) *via* elimination of HCl (**ii**) as described by Hegarty *et al.*[12] For hydrazonoyl chlorides, an addition-elimination mechanism (**iii**) is also in principle possible. The stable product is formed from the initial adduct through an *O*,*N*-acyl shift.	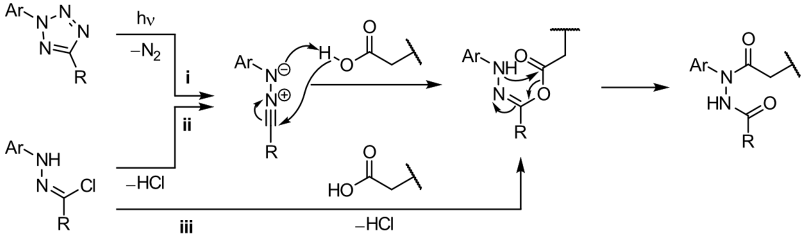
**Isx-alkyne**	Mechanism of labelling of aspartate and glutamate with isoxazolium salt **Isx-alkyne** as described by Woodward and Olofson.[13]	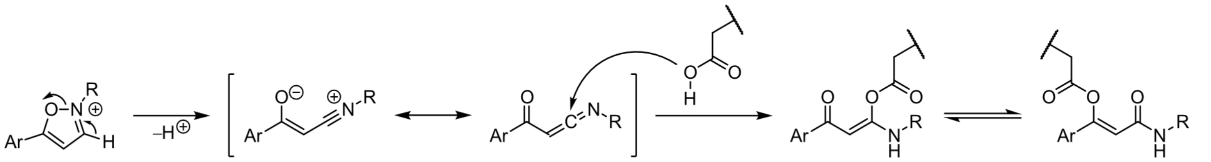

Supplementary Table 1 (continued)

Probe(s)	Comment	Reaction
**Az-alkyne**	Mechanism of labelling of aspartate and glutamate with 2*H*-azirines (**Az-alkyne**) as described by Black and Doyle (i).[14] Furthermore, a speculative reaction mechanism with nucleophiles leading to a modification that is 1 Da heavier than the expected adduct is shown (ii).	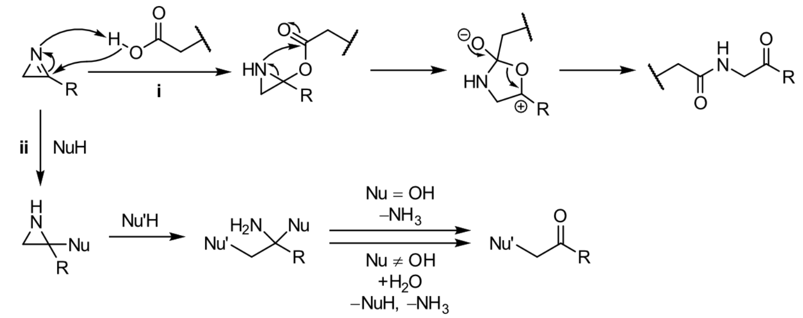
**SuFEx-alkyne**<br>**SuTEx1-alkyne**<br>**SuTEx2-alkyne**	Reaction of tyrosines with sulfonyl fluorides and sulfonyl triazoles.	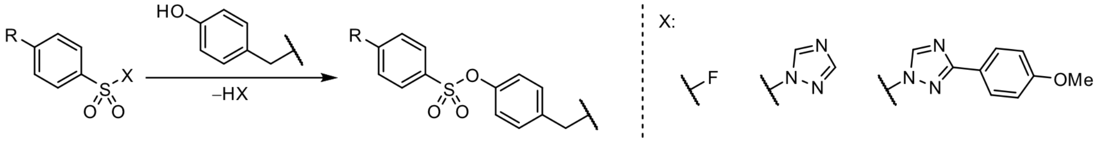
**PTAD-alkyne**	Mechanism of tyrosine-labelling with 4-substituted 3*H*-1,2,4-triazole-3,5(4*H*)-diones (**PTAD-alkyne**) through electrophilic aromatic substitution as described by Kaiser *et al.*[15] (top). Furthermore, formation of isocyanates from 4-substituted 3*H*-1,2,4-triazole-3,5(4*H*)-diones (**PTAD-alkyne**) and subsequent labelling of lysine and the *N*-terminus as described by Ban *et al.* is shown (bottom).[16]	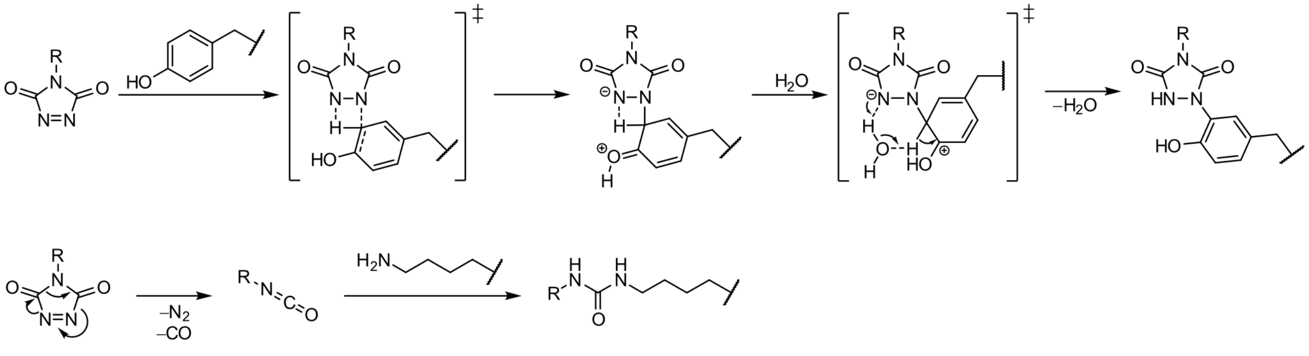

Supplementary Table 1 (continued)

Probe(s)	Comment	Reaction
**DA1-alkyne**<br>**DA2-alkyne**<br>**DA3-alkyne**	Plausible radical arylation of cysteine and aromatic amino acids after *in situ* generation of aryl radicals from diazonium ions (**DA1-**, **DA2-** and **DA3-alkyne**) as described by Naveen *et al.* for cysteine[17] and by Fehler *et al.* for tyrosine and tryptophan.[18] In the complex setting of cellular lysates, aryl radicals could be formed through either an addition-fragmentation mechanism or a single electron transfer (SET) and elimination of N_2.[19] Among others, NADH, ascorbate and glutathione have been described as reductants of diazonium ions under physiological conditions.[20, 21]	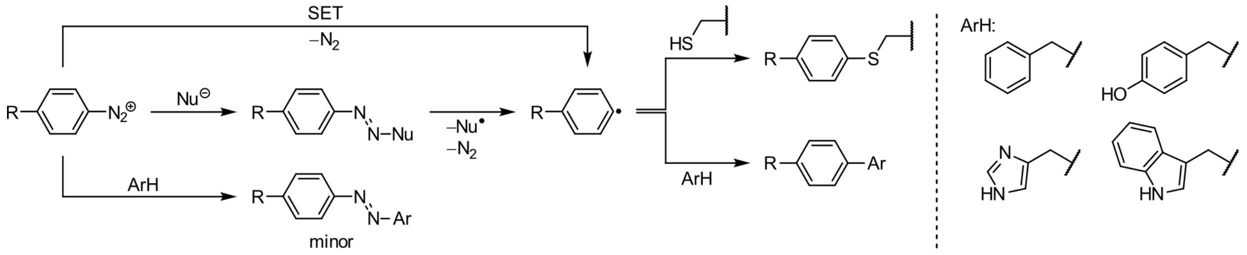
**OxMet1-alkyne**<br>**OxMet2-alkyne**	Mechanism of labelling of methionine with oxaziridines (**OxMet1-** and **OxMet2-alkyne**) as described by Lin *et al.*[22]	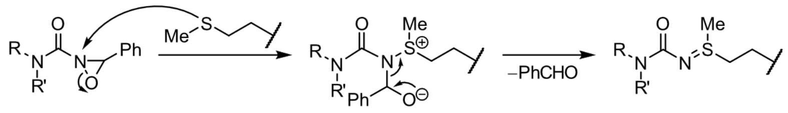

Supplementary Table 1 (continued)

Probe(s)	Comment	Reaction
**CP-alkyne**	Mechanism of tryptophan-labelling with *N*-carbamoyl-pyridinium salts (**CP-alkyne**) through photoinduced electron transfer (PET) as described by Tower *et al.*[23]	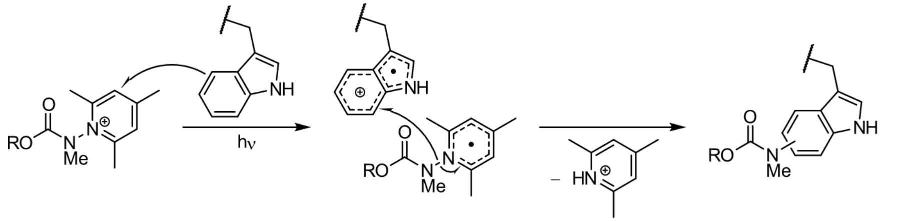
**HMN-alkyne** **HMP-alkyne** **MMP-alkyne**	Mechanism of tryptophan-labelling through reaction of the indole with *in situ* generated *ortho*-quinone methides (from **HMN-**, **HMP-** and **MMP-alkyne**) as described by Lin *et al.*[24] Alternatively, the reaction could also proceed through a concerted [4+2]-cyclo-addition as reported by Uyanik *et al.*[25]	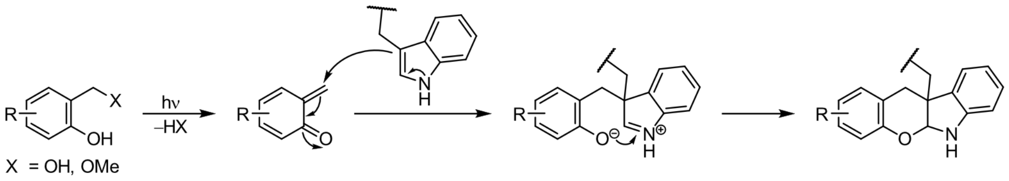
**PhGO-alkyne**	Reaction of a glyoxal (hydrate) (**PhGO-alkyne**) with arginine as described by Thompson *et al.*[26]	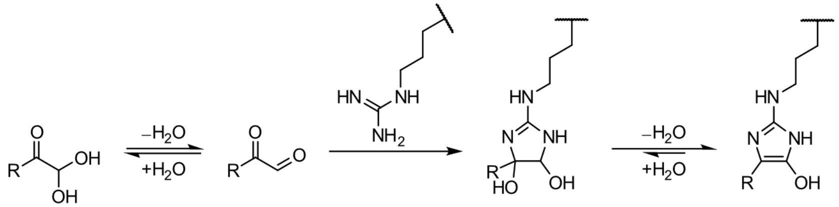
**FP-alkyne**	Reaction of serines with fluorophosphonates (**FP-alkyne**).	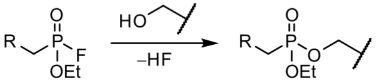
**PSI-alkyne**	Mechanism of serine-labelling with P(V) reagents (**PSI-alkyne**) as described by Vantourout *et al.*[27]	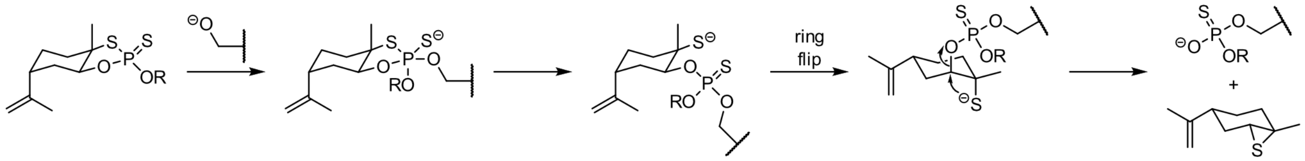

References

1. Frei R, Wodrich MD, Hari DP, Borin P-A, Chauvier C, Waser J. Fast and Highly Chemoselective Alkynylation of Thiols with Hypervalent Iodine Reagents Enabled through a Low Energy Barrier Concerted Mechanism. *J Am Chem Soc* 2014, **136**(47)**:** 16563-16573.
2. Tessier R, Ceballos J, Guidotti N, Simonet-Davin R, Fierz B, Waser J. "Doubly Orthogonal" Labeling of Peptides and Proteins. *Chem* 2019, **5**(8)**:** 2243-2263.
3. Liu B, Alegre-Requena JV, Paton RS, Miyake GM. Unconventional Reactivity of Ethynylbenziodoxolone Reagents and Thiols: Scope and Mechanism. *Chem Eur J* 2020, **26**(11)**:** 2386-2394.
4. Liu B, Lim C-H, Miyake GM. Light-Driven Intermolecular Charge Transfer Induced Reactivity of Ethynylbenziodoxol(on)e and Phenols. *J Am Chem Soc* 2018, **140**(40)**:** 12829-12835.
5. Tessier R, Nandi RK, Dwyer BG, Abegg D, Sornay C, Ceballos J*, et al.* Ethynylation of Cysteine Residues: From Peptides to Proteins in Vitro and in Living Cells. *Angew Chem Int Ed* 2020, **59**(27)**:** 10961-10970.
6. Boyatzis AE, Bringans SD, Piggott MJ, Duong MN, Lipscombe RJ, Arthur PG. Limiting the Hydrolysis and Oxidation of Maleimide–Peptide Adducts Improves Detection of Protein Thiol Oxidation. *J Proteome Res* 2017, **16**(5)**:** 2004-2015.
7. Deng J-R, Lai NC-H, Kung KK-Y, Yang B, Chung S-F, Leung AS-L*, et al.* N-Terminal selective modification of peptides and proteins using 2-ethynylbenzaldehydes. *Commun Chem* 2020, **3**(1)**:** 67.
8. Zhu JS, Kraemer N, Li CJ, Haddadin MJ, Kurth MJ. Photochemical Preparation of 1,2-Dihydro-3H-indazol-3-ones in Aqueous Solvent at Room Temperature. *J Org Chem* 2018, **83**(24)**:** 15493-15498.
9. Onoda A, Inoue N, Sumiyoshi E, Hayashi T. Triazolecarbaldehyde Reagents for One-Step N-Terminal Protein Modification. *ChemBioChem* 2020, **21**(9)**:** 1274-1278.
10. MacDonald JI, Munch HK, Moore T, Francis MB. One-step site-specific modification of native proteins with 2-pyridinecarboxyaldehydes. *Nat Chem Bio* 2015, **11**(5)**:** 326-331.
11. Meier H, Heimgartner H. Intramolekulare 1,3-dipolare Cycloadditionen von Diarylnitriliminen aus 2,5-Diaryltetrazolen. *Helv Chim Acta* 1985, **68**(5)**:** 1283-1300.
12. Hegarty AF, Cashman MP, Scott FL. The kinetics of nitrilimine formation in base-catalysed hydrolysis of hydrazonyl halides. *J Chem Soc, Perkin Trans 2* 1972(1)**:** 44-52.
13. Woodward RB, Olofson RA. The reaction of isoxazolium salts with nucleophiles. *Tetrahedron* 1966, **22:** 415-440.
14. Black D, Doyle J. Direct conversion of α-halo acids into α-halo amides by reaction with 2-phenylazirine. *Aust J Chem* 1978, **31**(10)**:** 2313-2315.
15. Kaiser D, Winne JM, Ortiz-Soto ME, Seibel J, Le TA, Engels B. Mechanistical Insights into the Bioconjugation Reaction of Triazolinediones with Tyrosine. *J Org Chem* 2018, **83**(17)**:** 10248-10260.
16. Ban H, Nagano M, Gavrilyuk J, Hakamata W, Inokuma T, Barbas CF. Facile and Stabile Linkages through Tyrosine: Bioconjugation Strategies with the Tyrosine-Click Reaction. *Bioconjug Chem* 2013, **24**(4)**:** 520-532.
17. Naveen N, Sengupta S, Chandrasekaran S. Metal-Free S-Arylation of Cysteine Using Arenediazonium Salts. *J Org Chem* 2018, **83**(7)**:** 3562-3569.
18. Fehler SK, Pratsch G, Östreicher C, Fürst MCD, Pischetsrieder M, Heinrich MR. Radical arylation of tyrosine residues in peptides. *Tetrahedron* 2016, **72**(48)**:** 7888-7893.
19. Koziakov D, Wu G, Jacobi von Wangelin A. Aromatic substitutions of arenediazonium salts via metal catalysis, single electron transfer, and weak base mediation. *Org Biomol Chem* 2018, **16**(27)**:** 4942-4953.
20. Reszka KJ, Chignell CF. One-electron reduction of arenediazonium compounds by physiological electron donors generates aryl radicals. An EPR and spin trapping investigation. *Chem-Biol Interact* 1995, **96**(3)**:** 223-234.
21. Nothling MD, Cao H, McKenzie TG, Hocking DM, Strugnell RA, Qiao GG. Bacterial Redox Potential Powers Controlled Radical Polymerization. *J Am Chem Soc* 2021, **143**(1)**:** 286-293.
22. Lin S, Yang X, Jia S, Weeks AM, Hornsby M, Lee PS*, et al.* Redox-based reagents for chemoselective methionine bioconjugation. *Science* 2017, **355**(6325)**:** 597-602.
23. Tower SJ, Hetcher WJ, Myers TE, Kuehl NJ, Taylor MT. Selective Modification of Tryptophan Residues in Peptides and Proteins Using a Biomimetic Electron Transfer Process. *J Am Chem Soc* 2020, **142**(20)**:** 9112-9118.
24. Lin C, Du H-J, Zhao H, Yan D-F, Liu N-X, Sun H*, et al.* A formal intermolecular [4 + 2] cycloaddition reaction of 1,3-disubstituted indoles and alkylquinones. *Org Biomol Chem* 2017, **15**(16)**:** 3472-3478.

25. Uyanik M, Nishioka K, Kondo R, Ishihara K. Chemoselective oxidative generation of ortho-quinone methides and tandem transformations. *Nat Chem* 2020, **12**(4)**:** 353-362.
26. Thompson DA, Ng R, Dawson PE. Arginine selective reagents for ligation to peptides and proteins. *J Pept Sci* 2016, **22**(5)**:** 311-319.
27. Vantourout JC, Adusumalli SR, Knouse KW, Flood DT, Ramirez A, Padial NM*, et al.* Serine-Selective Bioconjugation. *J Am Chem Soc* 2020, **142**(41)**:** 17236-17242.
